# Supplementary material for: The use of fast molecular descriptors and artificial neural networks approach in organochlorine compounds electron ionization mass spectra classification
Source: Environ Sci Pollut Res Int. 2019 Jul 30;26(27):28188–201. doi: 10.1007/s11356-019-05968-4 (PMC6791912; doi:10.1007/s11356-019-05968-4)
Supplement: Supplementary file 1 — (DOCX 303 kb) [file 11356_2019_5968_MOESM1_ESM.docx]

**Supplementary Material S1**

**The use of fast molecular descriptors and artificial neural networks approach** **in organochlorine compounds electron ionization mass spectra classification**

Maciej Przybyłek^a^, Waldemar Studziński^b^, Alicja Gackowska^b^ and Jerzy Gaca^b^

^a^*Chair and Department of Physical Chemistry, Pharmacy Faculty, Collegium Medicum of Bydgoszcz, Nicolaus Copernicus University in Toruń, Kurpińskiego 5, 85-950 Bydgoszcz, Poland,*

^b^*Faculty of Chemical Technology and Engineering, University of Technology and Life Science, Seminaryjna 3, 85-326 Bydgoszcz, Poland*

**Table S1** Mass spectra data of chlorine-containing organic comounds obtained from NIST Chemistry WebBook database (http://webbook.nist.gov/chemistry/)

| **Compound** | **IUPAC Standard InChIKey** | **SMILES** | **[M]** | **[M-35]** | **Criterion** | |
| --- | --- | --- | --- | --- | --- | --- |
|  |  |  |  |  | **I** | **II** |
| Cyanogen chloride (CClN) | QPJDMGCKMHUXFD-UHFFFAOYSA-N | C(#N)Cl | 9999 | 751 | 1 | 1 |
| Dichloromethane (CH2Cl2) | YMWUJEATGCHHMB-UHFFFAOYSA-N | C(Cl)Cl | 6549 | 9999 | 1 | 1 |
| Acetylene, chloro- (C2HCl) | DIWKDXFZXXCDLF-UHFFFAOYSA-N | C#CCl | 9999 | 2262 | 1 | 1 |
| Ethyl Chloride (C2H5Cl) | HRYZWHHZPQKTII-UHFFFAOYSA-N | CCCl | 9999 | 7216 | 1 | 1 |
| Ethene, chloro- (C2H3Cl) | BZHJMEDXRYGGRV-UHFFFAOYSA-N | C=CCl | 7660 | 9999 | 1 | 1 |
| Chloromethylmethyl sulfide (C2H5ClS) | JWMLCCRPDOIBAV-UHFFFAOYSA-N | CSCCl | 4404 | 9999 | 1 | 1 |
| Methyl chloromethyl ether (C2H5ClO) | XJUZRXYOEPSWMB-UHFFFAOYSA-N | COCCl | 70 | 9999 | 2 | 1 |
| Thiophosgene (CCl2S) | ZWZVWGITAAIFPS-UHFFFAOYSA-N | C(=S)(Cl)Cl | 2150 | 9999 | 1 | 1 |
| Phosgene (CCl2O) | YGYAWVDWMABLBF-UHFFFAOYSA-N | C(=O)(Cl)Cl | 679 | 9999 | 2 | 1 |
| Chloroform (CHCl3) | HEDRZPFGACZZDS-UHFFFAOYSA-N | C(Cl)(Cl)Cl | 250 | 9999 | 2 | 1 |
| Ethyne, dichloro- (C2Cl2) | ZMJOVJSTYLQINE-UHFFFAOYSA-N | C(#CCl)Cl | 9999 | 2502 | 1 | 1 |
| Chloroacetonitrile (C2H2ClN) | RENMDAKOXSCIGH-UHFFFAOYSA-N | C(C#N)Cl | 9999 | 4339 | 1 | 1 |
| 2-Chloroethanol (C2H5ClO) | SZIFAVKTNFCBPC-UHFFFAOYSA-N | C(CCl)O | 390 | 170 | 2 | 1 |
| Acetaldehyde, chloro- (C2H3ClO) | QSKPIOLLBIHNAC-UHFFFAOYSA-N | C(C=O)Cl | 4040 | 110 | 1 | 1 |
| Acetyl chloride (C2H3ClO) | WETWJCDKMRHUPV-UHFFFAOYSA-N | CC(=O)Cl | 50 | 9999 | 2 | 1 |
| 1,2-Dichloroethane (C2H4Cl2) | WSLDOOZREJYCGB-UHFFFAOYSA-N | C(CCl)Cl | 824 | 1397 | 1 | 1 |
| 1,1-Dichloroethane (C2H4Cl2) | SCYULBFZEHDVBN-UHFFFAOYSA-N | CC(Cl)Cl | 620 | 9999 | 2 | 1 |
| 3-Chloro-1-propyne (C3H3Cl) | LJZPPWWHKPGCHS-UHFFFAOYSA-N | C#CCCl | 3189 | 9999 | 1 | 1 |
| 1-Chloropropane (C3H7Cl) | SNMVRZFUUCLYTO-UHFFFAOYSA-N | CCCCl | 269 | 1369 | 2 | 1 |
| 2-Chloropropane (C3H7Cl) | ULYZAYCEDJDHCC-UHFFFAOYSA-N | CC(C)Cl | 899 | 9999 | 1 | 1 |
| 1,2-Dichloroethylene (C2H2Cl2) | KFUSEUYYWQURPO-UHFFFAOYSA-N | C(=CCl)Cl | 6916 | 9999 | 1 | 1 |
| Ethene, 1,1-dichloro- (C2H2Cl2) | LGXVIGDEPROXKC-UHFFFAOYSA-N | C=C(Cl)Cl | 6413 | 9999 | 1 | 1 |
| Allyl chloride (C3H5Cl) | OSDWBNJEKMUWAV-UHFFFAOYSA-N | C=CCCl | 3900 | 9999 | 1 | 1 |
| (Z)-1-Propene, 1-chloro (C3H5Cl) | OWXJKYNZGFSVRC-IHWYPQMZSA-N | C/C=C\Cl | 3783 | 9999 | 1 | 1 |
| (E)-1-Propene, 1-chloro (C3H5Cl) | OWXJKYNZGFSVRC-NSCUHMNNSA-N | C/C=C/Cl | 4024 | 9999 | 1 | 1 |
| Propene, 2-chloro- (C3H5Cl) | PNLQPWWBHXMFCA-UHFFFAOYSA-N | CC(=C)Cl | 4784 | 9999 | 1 | 1 |
| 1-Chloropropene (C3H5Cl) | OWXJKYNZGFSVRC-UHFFFAOYSA-N | CC=CCl | 4249 | 9999 | 1 | 1 |
| Chloromethyl thiocyanate (C2H2ClNS) | UXUCVNXUWOLPRU-UHFFFAOYSA-N | C(SC#N)Cl | 3463 | 4174 | 1 | 1 |
| Methane, chloro- (CH3Cl) | NEHMKBQYUWJMIP-UHFFFAOYSA-N | CCl | 9999 | 7237 | 1 | 1 |
| Chlorocarbonylsulfenyl chloride (CCl2OS) | MNOALXGAYUJNKX-UHFFFAOYSA-N | C(=O)(SCl)Cl | 1059 | 1789 | 1 | 1 |
| Carbonochloridothioic acid, S-methyl ester (C2H3ClOS) | YPSUCTSXOROPBS-UHFFFAOYSA-N | CSC(=O)Cl | 2400 | 9999 | 1 | 1 |
| Methyl chloroformate (C2H3ClO2) | XMJHPCRAQCTCFT-UHFFFAOYSA-N | COC(=O)Cl | 229 | 9999 | 2 | 1 |
| Bis(chloromethyl)ether (C2H4Cl2O) | HRQGCQVOJVTVLU-UHFFFAOYSA-N | C(OCCl)Cl | 0 | 9999 | 2 | 1 |
| 1,1-Dichloromethyl methyl ether (C2H4Cl2O) | GRTGGSXWHGKRSB-UHFFFAOYSA-N | COC(Cl)Cl | 0 | 9999 | 2 | 1 |
| Carbon Tetrachloride (CCl4) | VZGDMQKNWNREIO-UHFFFAOYSA-N | C(Cl)(Cl)(Cl)Cl | 0 | 9999 | 2 | 1 |
| Acetamide, 2-chloro- (C2H4ClNO) | VXIVSQZSERGHQP-UHFFFAOYSA-N | C(C(=O)N)Cl | 1419 | 249 | 1 | 1 |
| Acetic acid, chloro- (C2H3ClO2) | FOCAUTSVDIKZOP-UHFFFAOYSA-N | C(C(=O)O)Cl | 681 | 80 | 2 | 2 |
| 2-Chloroethyl methyl sulfide (C3H7ClS) | MYFKLQFBFSHBPA-UHFFFAOYSA-N | CSCCCl | 2949 | 799 | 1 | 1 |
| Ethane, 1-chloro-2-methoxy- (C3H7ClO) | XTIGGAHUZJWQMD-UHFFFAOYSA-N | COCCCl | 671 | 10 | 2 | 2 |
| Ethane, (chloromethoxy)- (C3H7ClO) | FCYRSDMGOLYDHL-UHFFFAOYSA-N | CCOCCl | 50 | 9999 | 2 | 1 |
| Acetonitrile, dichloro- (C2HCl2N) | STZZWJCGRKXEFF-UHFFFAOYSA-N | C(#N)C(Cl)Cl | 100 | 9999 | 2 | 1 |
| 2,2-Dichloroethanol (C2H4Cl2O) | IDJOCJAIQSKSOP-UHFFFAOYSA-N | C(C(Cl)Cl)O | 661 | 641 | 2 | 1 |
| Acetyl chloride, chloro- (C2H2Cl2O) | VGCXGMAHQTYDJK-UHFFFAOYSA-N | C(C(=O)Cl)Cl | 18 | 9999 | 2 | 1 |
| Dichloroacetaldehyde (C2H2Cl2O) | NWQWQKUXRJYXFH-UHFFFAOYSA-N | C(=O)C(Cl)Cl | 991 | 260 | 1 | 1 |
| 1,1,2-Trichloroethane (C2H3Cl3) | UBOXGVDOUJQMTN-UHFFFAOYSA-N | C(C(Cl)Cl)Cl | 1060 | 9999 | 1 | 1 |
| 1,1,1-Trichloroethane (C2H3Cl3) | UOCLXMDMGBRAIB-UHFFFAOYSA-N | CC(Cl)(Cl)Cl | 0 | 9999 | 2 | 1 |
| 3-Chloropropionitrile (C3H4ClN) | GNHMRTZZNHZDDM-UHFFFAOYSA-N | C(CCl)C#N | 1659 | 9999 | 1 | 1 |
| 1-Propanol, 3-chloro- (C3H7ClO) | LAMUXTNQCICZQX-UHFFFAOYSA-N | C(CO)CCl | 0 | 356 | 2 | 1 |
| CH3CHClCN (C3H4ClN) | JNAYPRPPXRWGQO-UHFFFAOYSA-N | CC(C#N)Cl | 1872 | 8678 | 1 | 1 |
| 2-Propanol, 1-chloro- (C3H7ClO) | YYTSGNJTASLUOY-UHFFFAOYSA-N | CC(CCl)O | 0 | 0 | 2 | 2 |
| 2-Chloropropanol (C3H7ClO) | VZIQXGLTRZLBEX-UHFFFAOYSA-N | CC(CO)Cl | 160 | 260 | 2 | 1 |
| Chloroacetone (C3H5ClO) | BULLHNJGPPOUOX-UHFFFAOYSA-N | CC(=O)CCl | 1091 | 200 | 1 | 1 |
| Propanoyl chloride (C3H5ClO) | RZWZRACFZGVKFM-UHFFFAOYSA-N | CCC(=O)Cl | 20 | 9909 | 2 | 1 |
| 1,3-Dichloropropane (C3H6Cl2) | YHRUOJUYPBUZOS-UHFFFAOYSA-N | C(CCl)CCl | 90 | 529 | 2 | 1 |
| 1,2-Dichloropropane (C3H6Cl2) | KNKRKFALVUDBJE-UHFFFAOYSA-N | CC(CCl)Cl | 349 | 1179 | 2 | 1 |
| 1,1-Dichloropropane (C3H6Cl2) | WIHMGGWNMISDNJ-UHFFFAOYSA-N | CCC(Cl)Cl | 58 | 9999 | 2 | 1 |
| 2,2-Dichloropropane (C3H6Cl2) | ZEOVXNVKXIPWMS-UHFFFAOYSA-N | CC(C)(Cl)Cl | 20 | 9999 | 2 | 1 |
| 2-Butyne, chloro (C4H5Cl) | OKWUYBGGPXXFLS-UHFFFAOYSA-N | CC#CCCl | 6099 | 9999 | 1 | 1 |
| 1-Chlorobutane (C4H9Cl) | VFWCMGCRMGJXDK-UHFFFAOYSA-N | CCCCCl | 40 | 570 | 2 | 1 |
| Propane, 1-chloro-2-methyl- (C4H9Cl) | QTBFPMKWQKYFLR-UHFFFAOYSA-N | CC(C)CCl | 30 | 179 | 2 | 1 |
| 2-Chlorobutane (C4H9Cl) | BSPCSKHALVHRSR-UHFFFAOYSA-N | CCC(C)Cl | 20 | 9999 | 2 | 1 |
| Propane, 2-chloro-2-methyl- (C4H9Cl) | NBRKLOOSMBRFMH-UHFFFAOYSA-N | CC(C)(C)Cl | 2 | 9999 | 2 | 1 |
| 2-Propenenitrile, 2-chloro- (C3H2ClN) | OYUNTGBISCIYPW-UHFFFAOYSA-N | C=C(C#N)Cl | 9169 | 9999 | 1 | 1 |
| 2-Chloro-2-propen-1-ol (C3H5ClO) | OSCXYTRISGREIM-UHFFFAOYSA-N | C=C(CO)Cl | 1025 | 9999 | 1 | 1 |
| 2-Propenoyl chloride (C3H3ClO) | HFBMWMNUJJDEQZ-UHFFFAOYSA-N | C=CC(=O)Cl | 50 | 9999 | 2 | 1 |
| cis-1,3-Dichloropropene (C3H4Cl2) | UOORRWUZONOOLO-UPHRSURJSA-N | C(/C=C\Cl)Cl | 1944 | 9999 | 1 | 1 |
| trans-1,3-Dichloropropene (C3H4Cl2) | UOORRWUZONOOLO-OWOJBTEDSA-N | C(/C=C/Cl)Cl | 2690 | 9999 | 1 | 1 |
| 1,3-dichloropropene (C3H4Cl2) | UOORRWUZONOOLO-UHFFFAOYSA-N | C(C=CCl)Cl | 2059 | 9999 | 1 | 1 |
| Propene, 3,3-dichloro- (C3H4Cl2) | VRTNIWBNFSHDEB-UHFFFAOYSA-N | C=CC(Cl)Cl | 261 | 9999 | 2 | 1 |
| 2,3-Dichloropropene (C3H4Cl2) | FALCMQXTWHPRIH-UHFFFAOYSA-N | C=C(CCl)Cl | 2750 | 9999 | 1 | 1 |
| 1-Propene, 1,2-dichloro- (C3H4Cl2) | PPKPKFIWDXDAGC-UHFFFAOYSA-N | CC(=CCl)Cl | 3889 | 9999 | 1 | 1 |
| 1,1-Dichloropropene (C3H4Cl2) | ZAIDIVBQUMFXEC-UHFFFAOYSA-N | CC=C(Cl)Cl | 3545 | 9999 | 1 | 1 |
| 1-Propene, 3-chloro-2-methyl- (C4H7Cl) | OHXAOPZTJOUYKM-UHFFFAOYSA-N | CC(=C)CCl | 3159 | 9999 | 1 | 1 |
| 3-Chloro-1-butene (C4H7Cl) | VZGLVCFVUREVDP-UHFFFAOYSA-N | CC(C=C)Cl | 916 | 9999 | 1 | 1 |
| 2-Butene, 1-chloro- (C4H7Cl) | YTKRILODNOEEPX-UHFFFAOYSA-N | CC=CCCl | 2499 | 9999 | 1 | 1 |
| 1-Butene, 1-chloro-, (Z)- (C4H7Cl) | DUDKKPVINWLFBI-ARJAWSKDSA-N | CC/C=C\Cl | 2988 | 9999 | 1 | 1 |
| 2-Butene, 2-chloro- (C4H7Cl) | DSDHFHLZEFQSFM-UHFFFAOYSA-N | CC=C(C)Cl | 3869 | 9999 | 1 | 1 |
| 1-Propene, 1-chloro-2-methyl- (C4H7Cl) | KWISWUFGPUHDRY-UHFFFAOYSA-N | CC(=CCl)C | 4129 | 9999 | 1 | 1 |
| Chloroprene (C4H5Cl) | YACLQRRMGMJLJV-UHFFFAOYSA-N | C=CC(=C)Cl | 4563 | 9999 | 1 | 1 |
| Epichlorohydrin (C3H5ClO) | BRLQWZUYTZBJKN-UHFFFAOYSA-N | C1C(O1)CCl | 30 | 9999 | 2 | 1 |
| Oxirane, (chloromethyl)-, (R)- (C3H5ClO) | BRLQWZUYTZBJKN-GSVOUGTGSA-N | C1[C@H](O1)CCl | 0 | 9999 | 2 | 1 |
| (Chloromethyl)cyclopropane (C4H7Cl) | ZVTQWXCKQTUVPY-UHFFFAOYSA-N | C1CC1CCl | 190 | 9999 | 2 | 1 |
| Chloromethyl sulfonylchloride (CH2Cl2O2S) | KQDDQXNVESLJNO-UHFFFAOYSA-N | C(S(=O)(=O)Cl)Cl | 0 | 475 | 2 | 1 |
| Carbamothioic chloride, dimethyl- (C3H6ClNS) | PHWISQNXPLXQRU-UHFFFAOYSA-N | CN(C)C(=S)Cl | 9999 | 9530 | 1 | 1 |
| Carbamic chloride, dimethyl- (C3H6ClNO) | YIIMEMSDCNDGTB-UHFFFAOYSA-N | CN(C)C(=O)Cl | 3230 | 9999 | 1 | 1 |
| Methanesulfenyl chloride, trichloro- (CCl4S) | RYFZYYUIAZYQLC-UHFFFAOYSA-N | C(SCl)(Cl)(Cl)Cl | 2400 | 9999 | 1 | 1 |
| 2-Chloroethyl isothiocyanate (C3H4ClNS) | ZUWFBQUHBOUPFK-UHFFFAOYSA-N | C(CCl)N=C=S | 5248 | 87 | 1 | 2 |
| Ethane, 1-chloro-2-nitro- (C2H4ClNO2) | XMZRJPITAOAPLJ-UHFFFAOYSA-N | C(CCl)[N+](=O)[O-] | 10 | 100 | 2 | 2 |
| 2-Chloro-N-methylacetamide (C3H6ClNO) | HOZLOOPIXHWKCI-UHFFFAOYSA-N | CNC(=O)CCl | 1926 | 1445 | 1 | 1 |
| methoxyacetyl chloride (C3H5ClO2) | JJKWHOSQTYYFAE-UHFFFAOYSA-N | COCC(=O)Cl | 30 | 20 | 2 | 2 |
| Methanol, chloro-, acetate (C3H5ClO2) | SMJYMSAPPGLBAR-UHFFFAOYSA-N | CC(=O)OCCl | 0 | 1131 | 2 | 1 |
| Methyl chloroacetate (C3H5ClO2) | QABLOFMHHSOFRJ-UHFFFAOYSA-N | COC(=O)CCl | 629 | 10 | 2 | 2 |
| Ethyl chloroformate (C3H5ClO2) | RIFGWPKJUGCATF-UHFFFAOYSA-N | CCOC(=O)Cl | 0 | 20 | 2 | 2 |
| Ethanamine, 2-chloro-N,N-dimethyl- (C4H10ClN) | WQMAANNAZKNUDL-UHFFFAOYSA-N | CN(C)CCCl | 439 | 749 | 2 | 1 |
| Acetamide, 2,2-dichloro- (C2H3Cl2NO) | WCGGWVOVFQNRRS-UHFFFAOYSA-N | C(C(=O)N)(Cl)Cl | 239 | 50 | 2 | 2 |
| Acetic acid, dichloro- (C2H2Cl2O2) | JXTHNDFMNIQAHM-UHFFFAOYSA-N | C(C(=O)O)(Cl)Cl | 50 | 329 | 2 | 1 |
| Oxalyl chloride (C2Cl2O2) | CTSLXHKWHWQRSH-UHFFFAOYSA-N | C(=O)(C(=O)Cl)Cl | 90 | 70 | 2 | 2 |
| 2,2-Dichloroethyl methyl ether (C3H6Cl2O) | QUCLHUUEEKVBGT-UHFFFAOYSA-N | COCC(Cl)Cl | 86 | 66 | 2 | 2 |
| Trichloroacetonitrile (C2Cl3N) | DRUIESSIVFYOMK-UHFFFAOYSA-N | C(#N)C(Cl)(Cl)Cl | 0 | 9999 | 2 | 1 |
| 2,2,2-Trichloroethanol (C2H3Cl3O) | KPWDGTGXUYRARH-UHFFFAOYSA-N | C(C(Cl)(Cl)Cl)O | 20 | 887 | 2 | 1 |
| Dichloroacetyl chloride (C2HCl3O) | FBCCMZVIWNDFMO-UHFFFAOYSA-N | C(C(=O)Cl)(Cl)Cl | 169 | 1009 | 2 | 1 |
| 1,1,1,2-Tetrachloroethane (C2H2Cl4) | QVLAWKAXOMEXPM-UHFFFAOYSA-N | C(C(Cl)(Cl)Cl)Cl | 0 | 9999 | 2 | 1 |
| Ethane, 1,1,2,2-tetrachloro- (C2H2Cl4) | QPFMBZIOSGYJDE-UHFFFAOYSA-N | C(C(Cl)Cl)(Cl)Cl | 741 | 1261 | 2 | 1 |
| 1,2-Propanediol, 3-chloro- (C3H7ClO2) | SSZWWUDQMAHNAQ-UHFFFAOYSA-N | C(C(CCl)O)O | 10 | 70 | 2 | 2 |
| 3-Chloropropionamide (C3H6ClNO) | JQDXZJYAUSVHDH-UHFFFAOYSA-N | C(CCl)C(=O)N | 659 | 7229 | 2 | 1 |
| Propanoic acid, 3-chloro- (C3H5ClO2) | QEYMMOKECZBKAC-UHFFFAOYSA-N | C(CCl)C(=O)O | 50 | 9999 | 2 | 1 |
| (S)-(+)-2-Chloro-1-propanol (C3H7ClO) | VZIQXGLTRZLBEX-GSVOUGTGSA-N | C[C@H](CO)Cl | 62 | 158 | 2 | 1 |
| 2-Chloroethyl ethyl sulfide (C4H9ClS) | GBNVXYXIRHSYEG-UHFFFAOYSA-N | CCSCCCl | 2849 | 499 | 1 | 1 |
| 1-Chloro-2-ethoxyethane (C4H9ClO) | GPTVQTPMFOLLOA-UHFFFAOYSA-N | CCOCCCl | 1039 | 40 | 1 | 2 |
| 2-Chloropropionamide (C3H6ClNO) | OEZPDHRXGCLGKB-UHFFFAOYSA-N | CC(C(=O)N)Cl | 70 | 3449 | 2 | 1 |
| (S)-(-)-2-Chloropropionic acid (C3H5ClO2) | GAWAYYRQGQZKCR-UWTATZPHSA-N | C[C@H](C(=O)O)Cl | 218 | 506 | 2 | 1 |
| Propanoic acid, 2-chloro- (C3H5ClO2) | GAWAYYRQGQZKCR-UHFFFAOYSA-N | CC(C(=O)O)Cl | 351 | 651 | 2 | 1 |
| 2,3-Dichloropropionitrile (C3H3Cl2N) | RJJDLPQZNANQDQ-UHFFFAOYSA-N | C(C(C#N)Cl)Cl | 92 | 9408 | 2 | 1 |
| 2,3-Dichloro-1-propanol (C3H6Cl2O) | ZXCYIJGIGSDJQQ-UHFFFAOYSA-N | C(C(CCl)Cl)O | 0 | 50 | 2 | 2 |
| 2-Propanol, 1,3-dichloro- (C3H6Cl2O) | DEWLEGDTCGBNGU-UHFFFAOYSA-N | C(C(CCl)O)Cl | 0 | 20 | 2 | 2 |
| Propanoyl chloride, 3-chloro- (C3H4Cl2O) | INUNLMUAPJVRME-UHFFFAOYSA-N | C(CCl)C(=O)Cl | 10 | 9999 | 2 | 1 |
| 1,3-Dichloroacetone (C3H4Cl2O) | SUNMBRGCANLOEG-UHFFFAOYSA-N | C(C(=O)CCl)Cl | 1139 | 40 | 1 | 2 |
| 2-chloropropionyl chloride (C3H4Cl2O) | JEQDSBVHLKBEIZ-UHFFFAOYSA-N | CC(C(=O)Cl)Cl | 0 | 2729 | 2 | 1 |
| 1,1-Dichloroacetone (C3H4Cl2O) | CSVFWMMPUJDVKH-UHFFFAOYSA-N | CC(=O)C(Cl)Cl | 50 | 319 | 2 | 1 |
| 1,2,3-Trichloropropane (C3H5Cl3) | CFXQEHVMCRXUSD-UHFFFAOYSA-N | C(C(CCl)Cl)Cl | 20 | 339 | 2 | 1 |
| 1,2,2-Trichloropropane (C3H5Cl3) | DAIIXVPKQATIMF-UHFFFAOYSA-N | CC(CCl)(Cl)Cl | 20 | 4424 | 2 | 1 |
| 1,1,2-Trichloropropane (C3H5Cl3) | GRSQYISVQKPZCW-UHFFFAOYSA-N | CC(C(Cl)Cl)Cl | 40 | 309 | 2 | 1 |
| Propane, 1,1,1-trichloro- (C3H5Cl3) | AVGQTJUPLKNPQP-UHFFFAOYSA-N | CCC(Cl)(Cl)Cl | 0 | 9999 | 2 | 1 |
| 2-Butyne, 1,4-dichloro- (C4H4Cl2) | RCHDLEVSZBOHOS-UHFFFAOYSA-N | C(C#CCCl)Cl | 2369 | 9999 | 1 | 1 |
| Butanenitrile, 4-chloro- (C4H6ClN) | ZFCFBWSVQWGOJJ-UHFFFAOYSA-N | C(CC#N)CCl | 10 | 219 | 2 | 1 |
| 4-Chloro-1-butanol (C4H9ClO) | HXHGULXINZUGJX-UHFFFAOYSA-N | C(CCCl)CO | 0 | 129 | 2 | 1 |
| 2-Butanone, 4-chloro- (C4H7ClO) | MAGOYBJJLVSJIC-UHFFFAOYSA-N | CC(=O)CCCl | 190 | 1188 | 2 | 1 |
| 2-Butanone, 1-chloro- (C4H7ClO) | AALRHBLMAVGWRR-UHFFFAOYSA-N | CCC(=O)CCl | 591 | 175 | 2 | 1 |
| Butanoyl chloride (C4H7ClO) | DVECBJCOGJRVPX-UHFFFAOYSA-N | CCCC(=O)Cl | 110 | 9999 | 2 | 1 |
| 2-Butanone, 3-chloro- (C4H7ClO) | OIMRLHCSLQUXLL-UHFFFAOYSA-N | CC(C(=O)C)Cl | 579 | 70 | 2 | 2 |
| isobutyryl chloride (C4H7ClO) | DGMOBVGABMBZSB-UHFFFAOYSA-N | CC(C)C(=O)Cl | 10 | 3209 | 2 | 1 |
| 1,4-Dichlorobutane (C4H8Cl2) | KJDRSWPQXHESDQ-UHFFFAOYSA-N | C(CCCl)CCl | 0 | 329 | 2 | 1 |
| 1,3-Dichlorobutane (C4H8Cl2) | QBGVARBIQGHVKR-UHFFFAOYSA-N | CC(CCCl)Cl | 10 | 549 | 2 | 1 |
| Propane, 1,3-dichloro-2-methyl- (C4H8Cl2) | VENSUTAUIFXSRD-UHFFFAOYSA-N | CC(CCl)CCl | 360 | 1001 | 2 | 1 |
| 1,2-Dichlorobutane (C4H8Cl2) | PQBOTZNYFQWRHU-UHFFFAOYSA-N | CCC(CCl)Cl | 80 | 739 | 2 | 1 |
| 1,1-Dichlorobutane (C4H8Cl2) | SEQRDAAUNCRFIT-UHFFFAOYSA-N | CCCC(Cl)Cl | 10 | 851 | 2 | 1 |
| DL-2,3-Dichlorobutane (C4H8Cl2) | RMISVOPUIFJTEO-IMJSIDKUSA-N | C[C@@H]([C@H](C)Cl)Cl | 150 | 671 | 2 | 1 |
| meso-2,3-Dichlorobutane (C4H8Cl2) | RMISVOPUIFJTEO-ZXZARUISSA-N | C[C@H]([C@H](C)Cl)Cl | 150 | 691 | 2 | 1 |
| Propane, 1,2-dichloro-2-methyl- (C4H8Cl2) | OQPNDCHKFIHPBY-UHFFFAOYSA-N | CC(C)(CCl)Cl | 3 | 1978 | 2 | 1 |
| 2,3-Dichlorobutane (C4H8Cl2) | RMISVOPUIFJTEO-UHFFFAOYSA-N | CC(C(C)Cl)Cl | 179 | 929 | 2 | 1 |
| 2,2-Dichlorobutane (C4H8Cl2) | BSRTYNDWQXVCKR-UHFFFAOYSA-N | CCC(C)(Cl)Cl | 0 | 9999 | 2 | 1 |
| 1-Chloropentane (C5H11Cl) | SQCZQTSHSZLZIQ-UHFFFAOYSA-N | CCCCCCl | 168 | 475 | 2 | 1 |
| 1-Chloro-3-methylbutane (C5H11Cl) | CZHLPWNZCJEPJB-UHFFFAOYSA-N | CC(C)CCCl | 40 | 1029 | 2 | 1 |
| 1-Chloro-2-methylbutane (C5H11Cl) | IWAKWOFEHSYKSI-UHFFFAOYSA-N | CCC(C)CCl | 44 | 66 | 2 | 2 |
| 3-Chloropentane (C5H11Cl) | CXQSCYIVCSCSES-UHFFFAOYSA-N | CCC(CC)Cl | 42 | 4309 | 2 | 1 |
| 2-Chloropentane (C5H11Cl) | NFRKUDYZEVQXTE-UHFFFAOYSA-N | CCCC(C)Cl | 14 | 1764 | 2 | 1 |
| Propane, 1-chloro-2,2-dimethyl- (C5H11Cl) | JEKYMVBQWWZVHO-UHFFFAOYSA-N | CC(C)(C)CCl | 0 | 28 | 2 | 2 |
| 2-Chloro-3-methylbutane (C5H11Cl) | JMTRCXPSDMMAGM-UHFFFAOYSA-N | CC(C)C(C)Cl | 13 | 364 | 2 | 1 |
| 2-Chloro-2-methylbutane (C5H11Cl) | CRNIHJHMEQZAAS-UHFFFAOYSA-N | CCC(C)(C)Cl | 0 | 9519 | 2 | 1 |
| Ethene, 1,2-dichloro-, (Z)- (C2H2Cl2) | KFUSEUYYWQURPO-UPHRSURJSA-N | C(=C\Cl)\Cl | 6349 | 9999 | 1 | 1 |
| Ethene, 1,2-dichloro-, (E)- (C2H2Cl2) | KFUSEUYYWQURPO-OWOJBTEDSA-N | C(=C/Cl)\Cl | 6979 | 9999 | 1 | 1 |
| Trichloroethylene (C2HCl3) | XSTXAVWGXDQKEL-UHFFFAOYSA-N | C(=C(Cl)Cl)Cl | 9999 | 9248 | 1 | 1 |
| Tetrachloroethylene (C2Cl4) | CYTYCFOTNPOANT-UHFFFAOYSA-N | C(=C(Cl)Cl)(Cl)Cl | 8077 | 7176 | 1 | 1 |
| 2-Chloroethyl vinyl sulfide (C4H7ClS) | KCCNHHACDJEDCX-UHFFFAOYSA-N | C=CSCCCl | 5104 | 730 | 1 | 1 |
| 2-Chloroethyl vinyl ether (C4H7ClO) | DNJRKFKAFWSXSE-UHFFFAOYSA-N | C=COCCCl | 2729 | 70 | 1 | 2 |
| 2-Propenoic acid, 3-chloro-, (Z)- (C3H3ClO2) | MHMUCYJKZUZMNJ-UPHRSURJSA-N | C(=C\Cl)\C(=O)O | 8399 | 6869 | 1 | 1 |
| trans-3-Chloroacrylic acid (C3H3ClO2) | MHMUCYJKZUZMNJ-OWOJBTEDSA-N | C(=C/Cl)\C(=O)O | 6378 | 6733 | 1 | 1 |
| 2-Chloroacrylic acid (C3H3ClO2) | SZTBMYHIYNGYIA-UHFFFAOYSA-N | C=C(C(=O)O)Cl | 7956 | 4981 | 1 | 1 |
| 1-Propene, 1,2,3-trichloro-, (Z)- (C3H3Cl3) | HIILBTHBHCLUER-IWQZZHSRSA-N | C(/C(=C/Cl)/Cl)Cl | 2050 | 9999 | 1 | 1 |
| trans-1,2,3-Trichloropropene (C3H3Cl3) | HIILBTHBHCLUER-HNQUOIGGSA-N | C(/C(=C\Cl)/Cl)Cl | 1910 | 9999 | 1 | 1 |
| 1,1,3-Trichloropropene (C3H3Cl3) | JFEVIPGMXQNRRF-UHFFFAOYSA-N | C(C=C(Cl)Cl)Cl | 840 | 9999 | 1 | 1 |
| 1-Propene, 1,2,3-trichloro- (C3H3Cl3) | HIILBTHBHCLUER-UHFFFAOYSA-N | C(C(=CCl)Cl)Cl | 2249 | 9999 | 1 | 1 |
| 1-Propene, 1,1,2-trichloro- (C3H3Cl3) | LIPPKMMVZOHCIF-UHFFFAOYSA-N | CC(=C(Cl)Cl)Cl | 2562 | 9999 | 1 | 1 |
| 2-Butenoyl chloride (C4H5ClO) | RJUIDDKTATZJFE-UHFFFAOYSA-N | C/C=C/C(=O)Cl | 0 | 9999 | 2 | 1 |
| Methacryloyl chloride (C4H5ClO) | VHRYZQNGTZXDNX-UHFFFAOYSA-N | CC(=C)C(=O)Cl | 20 | 9999 | 2 | 1 |
| trans-1,4-dichloro-2-butene (C4H6Cl2) | FQDIANVAWVHZIR-OWOJBTEDSA-N | C(/C=C/CCl)Cl | 2150 | 6030 | 1 | 1 |
| 1-Propene, 3-chloro-2-(chloromethyl)- (C4H6Cl2) | XJFZOSUFGSANIF-UHFFFAOYSA-N | C=C(CCl)CCl | 1702 | 6120 | 1 | 1 |
| 3,4-Dichloro-1-butene (C4H6Cl2) | XVEASTGLHPVZNA-UHFFFAOYSA-N | C=CC(CCl)Cl | 199 | 4369 | 2 | 1 |
| 1,4-Dichloro-2-butene (C4H6Cl2) | FQDIANVAWVHZIR-UHFFFAOYSA-N | C(/C=C/CCl)Cl | 1700 | 4500 | 1 | 1 |
| cis-1,3-Dichloro-2-butene (C4H6Cl2) | WLIADPFXSACYLS-UHFFFAOYSA-N | C/C(=C/CCl)/Cl | 1939 | 9999 | 1 | 1 |
| 1-Pentene, 5-chloro- (C5H9Cl) | UPOBJNRMUDPATE-UHFFFAOYSA-N | C=CCCCCl | 1104 | 1749 | 1 | 1 |
| 4-Chloro-2-methyl-1-butene (C5H9Cl) | LGMPVUDVTHHFDR-UHFFFAOYSA-N | CC(=C)CCCl | 3077 | 7070 | 1 | 1 |
| 4-Chloro-3-methyl-1-butene (C5H9Cl) | ZFXHRKNMXMGZNU-UHFFFAOYSA-N | CC(CCl)C=C | 467 | 4255 | 2 | 1 |
| 1-Chloro-3-methyl-2-butene (C5H9Cl) | JKXQKGNGJVZKFA-UHFFFAOYSA-N | CC(=CCCl)C | 3430 | 9999 | 1 | 1 |
| 1-Butene, 3-chloro-2-methyl- (C5H9Cl) | UCUDJURUNUQAHQ-UHFFFAOYSA-N | CC(C(=C)C)Cl | 2036 | 9999 | 1 | 1 |
| 2-Chloro-3-methyl-1-butene (C5H9Cl) | RBSYGFLXVMWYGD-UHFFFAOYSA-N | CC(C)C(=C)Cl | 2068 | 9999 | 1 | 1 |
| 1-Chloro-3-methyl-1-butene (C5H9Cl) | MXVSJNLRVLKAOG-ONEGZZNKSA-N | CC(C)/C=C/Cl | 2717 | 9999 | 1 | 1 |
| 2-Chloro-3-methyl-2-butene (C5H9Cl) | WIIKEBDPJPYJHF-UHFFFAOYSA-N | CC(=C(C)Cl)C | 4854 | 9560 | 1 | 1 |
| 1,3-Butadiene, 1,4-dichloro- (C4H4Cl2) | LDZSRRMBFGBOAE-ZPUQHVIOSA-N | C(=C/Cl)\C=C\Cl | 4444 | 9999 | 1 | 1 |
| 1H-1,2,4-Triazole, 3-chloro- (C2H2ClN3) | QGOUKZPSCTVYLX-UHFFFAOYSA-N | C1=NNC(=N1)Cl | 9562 | 78 | 1 | 2 |
| Cyclopropanecarboxylic acid chloride (C4H5ClO) | ZOOSILUVXHVRJE-UHFFFAOYSA-N | C1CC1C(=O)Cl | 32 | 9999 | 2 | 1 |
| Cyclopentene, 1-chloro- (C5H7Cl) | UJUIJZWQFDQKHO-UHFFFAOYSA-N | C1CC=C(C1)Cl | 2450 | 9999 | 1 | 1 |
| 3-Chlorothiophene (C4H3ClS) | QUBJDMPBDURTJT-UHFFFAOYSA-N | C1=CSC=C1Cl | 9999 | 3619 | 1 | 1 |
| Thiophene, 2-chloro- (C4H3ClS) | GSFNQBFZFXUTBN-UHFFFAOYSA-N | C1=CSC(=C1)Cl | 9999 | 7189 | 1 | 1 |
| Phosphonous dichloride, (trichloromethyl)- (CCl5P) | RWFGEVVWSNJXRX-UHFFFAOYSA-N | C(P(Cl)Cl)(Cl)(Cl)Cl | 390 | 3593 | 2 | 1 |
| Chloropicrin (CCl3NO2) | LFHISGNCFUNFFM-UHFFFAOYSA-N | C([N+](=O)[O-])(Cl)(Cl)Cl | 0 | 76 | 2 | 2 |
| Phosphonic acid, (2-chloroethyl)- (C2H6ClO3P) | UDPGUMQDCGORJQ-UHFFFAOYSA-N | C(CCl)P(=O)(O)O | 0 | 4134 | 2 | 1 |
| Carbamic acid, 2-chloroethyl ester (C3H6ClNO2) | LIJLYNWYKULUHA-UHFFFAOYSA-N | C(CCl)OC(=O)N | 0 | 1390 | 2 | 1 |
| Chloroacetyl isocyanate (C3H2ClNO2) | MOVMEFHWBOWMFU-UHFFFAOYSA-N | C(C(=O)N=C=O)Cl | 751 | 0 | 2 | 2 |
| 2-Methoxyethoxymethyl chloride (C4H9ClO2) | BIAAQBNMRITRDV-UHFFFAOYSA-N | COCCOCCl | 0 | 1131 | 2 | 1 |
| Ethane, 2-chloro-1,1-dimethoxy- (C4H9ClO2) | CRZJPEIBPQWDGJ-UHFFFAOYSA-N | COC(CCl)OC | 0 | 10 | 2 | 2 |
| Carbonochloridic acid, 2-chloroethyl ester (C3H4Cl2O2) | SVDDJQGVOFZBNX-UHFFFAOYSA-N | C(CCl)OC(=O)Cl | 0 | 159 | 2 | 1 |
| chloromethyl chloroacetate (C3H4Cl2O2) | RGXOOYDUNWUMTN-UHFFFAOYSA-N | C(C(=O)OCCl)Cl | 0 | 0 | 2 | 2 |
| Ethane, 1,1-dichloro-1-nitro- (C2H3Cl2NO2) | OQOGEOLRYAOSKO-UHFFFAOYSA-N | CC([N+](=O)[O-])(Cl)Cl | 0 | 0 | 2 | 2 |
| 1-Chloroethyl chloroformate (C3H4Cl2O2) | QOPVNWQGBQYBBP-UHFFFAOYSA-N | CC(OC(=O)Cl)Cl | 0 | 2671 | 2 | 1 |
| Methyl dichloroacetate (C3H4Cl2O2) | HKMLRUAPIDAGIE-UHFFFAOYSA-N | COC(=O)C(Cl)Cl | 0 | 30 | 2 | 2 |
| Chloral Hydrate (C2H3Cl3O2) | RNFNDJAIBTYOQL-UHFFFAOYSA-N | C(C(Cl)(Cl)Cl)(O)O | 0 | 0 | 2 | 2 |
| Acetamide, 2,2,2-trichloro- (C2H2Cl3NO) | UPQQXPKAYZYUKO-UHFFFAOYSA-N | C(=O)(C(Cl)(Cl)Cl)N | 0 | 179 | 2 | 1 |
| 2,2,2-Trichloroethanol, methyl ether (C3H5Cl3O) | NZZHHOMCJICYCD-UHFFFAOYSA-N | COCC(Cl)(Cl)Cl | 30 | 1211 | 2 | 1 |
| Trichloroacetyl chloride (C2Cl4O) | PVFOMCVHYWHZJE-UHFFFAOYSA-N | C(=O)(C(Cl)(Cl)Cl)Cl | 0 | 550 | 2 | 1 |
| Ethane, pentachloro- (C2HCl5) | BNIXVQGCZULYKV-UHFFFAOYSA-N | C(C(Cl)(Cl)Cl)(Cl)Cl | 40 | 7909 | 2 | 1 |
| 3-Chloropropyl isothiocyanate (C4H6ClNS) | ZGFOBQSQLJDLKV-UHFFFAOYSA-N | C(CN=C=S)CCl | 5328 | 1433 | 1 | 1 |
| Hemisulfur mustard (C4H9ClOS) | ZGFPMAMREQRRRB-UHFFFAOYSA-N | C(CSCCCl)O | 2262 | 581 | 1 | 1 |
| Ethanol, 2-(2-chloroethoxy)- (C4H9ClO2) | LECMBPWEOVZHKN-UHFFFAOYSA-N | C(COCCCl)O | 0 | 30 | 2 | 2 |
| Propane, 1-chloro-2-nitro- (C3H6ClNO2) | FPJNQQRSBJPGHM-UHFFFAOYSA-N | CC(CCl)[N+](=O)[O-] | 0 | 0 | 2 | 2 |
| N-(2-Chloroethyl)acetamide (C4H8ClNO) | HSKNJSHFPPHTAQ-UHFFFAOYSA-N | CC(=O)NCCCl | 1648 | 911 | 1 | 1 |
| Ethanol, 2-chloro-, acetate (C4H7ClO2) | VIRWKAJWTKAIMA-UHFFFAOYSA-N | CC(=O)OCCCl | 0 | 190 | 2 | 1 |
| Propanoic acid, 3-chloro-, methyl ester (C4H7ClO2) | GZGJIACHBCQSPC-UHFFFAOYSA-N | COC(=O)CCCl | 300 | 9999 | 2 | 1 |
| 2-Chloro-N-ethylacetamide (C4H8ClNO) | JUBORNFANZZVJL-UHFFFAOYSA-N | CCNC(=O)CCl | 949 | 4731 | 1 | 1 |
| Ethyl chloroacetate (C4H7ClO2) | VEUUMBGHMNQHGO-UHFFFAOYSA-N | CCOC(=O)CCl | 70 | 10 | 2 | 2 |
| Propyl chloroformate (C4H7ClO2) | QQKDTTWZXHEGAQ-UHFFFAOYSA-N | CCCOC(=O)Cl | 0 | 0 | 2 | 2 |
| Propane, 2-chloro-2-nitro- (C3H6ClNO2) | JQYFSFNSNVRUPY-UHFFFAOYSA-N | CC(C)([N+](=O)[O-])Cl | 0 | 0 | 2 | 2 |
| Propanoic acid, 2-chloro-, methyl ester (C4H7ClO2) | JLEJCNOTNLZCHQ-UHFFFAOYSA-N | CC(C(=O)OC)Cl | 90 | 1560 | 2 | 1 |
| Nitrogen mustard (C4H9Cl2N) | TXFLGZOGNOOEFZ-UHFFFAOYSA-N | C(CCl)NCCCl | 319 | 369 | 2 | 1 |
| Bis(2-chloroethyl) sulphide (C4H8Cl2S) | QKSKPIVNLNLAAV-UHFFFAOYSA-N | C(CCl)SCCCl | 2382 | 310 | 1 | 1 |
| Propanedinitrile, dichloro- (C3Cl2N2) | NCWGPYPDPWOJKC-UHFFFAOYSA-N | C(#N)C(C#N)(Cl)Cl | 30 | 9999 | 2 | 1 |
| Bis(2-chloroethyl) ether (C4H8Cl2O) | ZNSMNVMLTJELDZ-UHFFFAOYSA-N | C(CCl)OCCCl | 126 | 0 | 2 | 2 |
| 2,3-dichloropropionic acid (C3H4Cl2O2) | GKFWNPPZHDYVLI-UHFFFAOYSA-N | C(C(C(=O)O)Cl)Cl | 0 | 5669 | 2 | 1 |
| Ethane, 1,2-dichloro-1-ethoxy- (C4H8Cl2O) | NNBUKAPOVBEMNI-UHFFFAOYSA-N | CCOC(CCl)Cl | 0 | 2019 | 2 | 1 |
| Propanoic acid, 2,2-dichloro- (C3H4Cl2O2) | NDUPDOJHUQKPAG-UHFFFAOYSA-N | CC(C(=O)O)(Cl)Cl | 0 | 2139 | 2 | 1 |
| 2,3-dichloropropionyl chloride (C3H3Cl3O) | JQELECXPPAOSTM-UHFFFAOYSA-N | C(C(C(=O)Cl)Cl)Cl | 0 | 5195 | 2 | 1 |
| 1,1,3-Trichloroacetone (C3H3Cl3O) | ZWILTCXCTVMANU-UHFFFAOYSA-N | C(C(=O)C(Cl)Cl)Cl | 142 | 53 | 2 | 2 |
| 1,1,1-Trichloro-2-propanol (C3H5Cl3O) | HCMBPASAOZIEDZ-UHFFFAOYSA-N | CC(C(Cl)(Cl)Cl)O | 0 | 339 | 2 | 1 |
| Propanoyl chloride, 2,2-dichloro- (C3H3Cl3O) | IPKCHUGFUGHNRZ-UHFFFAOYSA-N | CC(C(=O)Cl)(Cl)Cl | 0 | 100 | 2 | 2 |
| 2-Propanone, 1,1,1-trichloro- (C3H3Cl3O) | SMZHKGXSEAGRTI-UHFFFAOYSA-N | CC(=O)C(Cl)(Cl)Cl | 0 | 1149 | 2 | 1 |
| Propane, 1,1,1,3-tetrachloro- (C3H4Cl4) | UTACNSITJSJFHA-UHFFFAOYSA-N | C(CCl)C(Cl)(Cl)Cl | 0 | 6696 | 2 | 1 |
| 1,2,2,3-Tetrachloropropane (C3H4Cl4) | UDPHJTAYHSSOQB-UHFFFAOYSA-N | C(C(CCl)(Cl)Cl)Cl | 30 | 3559 | 2 | 1 |
| Propane, 1,1,2,3-tetrachloro- (C3H4Cl4) | BUQMVYQMVLAYRU-UHFFFAOYSA-N | C(C(C(Cl)Cl)Cl)Cl | 20 | 671 | 2 | 1 |
| Propane, 1,1,2,2-tetrachloro- (C3H4Cl4) | MDCBRXYTSHYYJE-UHFFFAOYSA-N | CC(C(Cl)Cl)(Cl)Cl | 20 | 2803 | 2 | 1 |
| 1,1,1,2-Tetrachloropropane (C3H4Cl4) | FEKGWIHDBVDVSM-UHFFFAOYSA-N | CC(C(Cl)(Cl)Cl)Cl | 20 | 4949 | 2 | 1 |
| Butanoic acid, 4-chloro- (C4H7ClO2) | IPLKGJHGWCVSOG-UHFFFAOYSA-N | C(CC(=O)O)CCl | 0 | 390 | 2 | 1 |
| Propane, 1-(2-chloroethoxy)- (C5H11ClO) | BHDSGQOSIWVMJW-UHFFFAOYSA-N | CCCOCCCl | 470 | 0 | 2 | 2 |
| Butanoic acid, 3-chloro- (C4H7ClO2) | XEEMVPPCXNTVNP-UHFFFAOYSA-N | CC(CC(=O)O)Cl | 20 | 9999 | 2 | 1 |
| 2-Chlorobutyramide (C4H8ClNO) | VJIOSCMTZVXQQU-UHFFFAOYSA-N | CCC(C(=O)N)Cl | 20 | 1061 | 2 | 1 |
| Butanoic acid, 2-chloro- (C4H7ClO2) | RVBUZBPJAGZHSQ-UHFFFAOYSA-N | CCC(C(=O)O)Cl | 20 | 2519 | 2 | 1 |
| Butanoyl chloride, 4-chloro- (C4H6Cl2O) | CDIIZULDSLKBKV-UHFFFAOYSA-N | C(CC(=O)Cl)CCl | 0 | 9999 | 2 | 1 |
| Butanoyl chloride, 2-chloro- (C4H6Cl2O) | KVQJVAOMYWTLEO-UHFFFAOYSA-N | CCC(C(=O)Cl)Cl | 0 | 2192 | 2 | 1 |
| Propane, 1,1,2-trichloro-2-methyl- (C4H7Cl3) | FRRHZKFKOHHEJR-UHFFFAOYSA-N | CC(C)(C(Cl)Cl)Cl | 0 | 789 | 2 | 1 |
| 5-Chlorovaleronitrile (C5H8ClN) | JSAWFGSXRPCFSW-UHFFFAOYSA-N | C(CCCl)CC#N | 3 | 1275 | 2 | 1 |
| 2-Pentanone, 5-chloro- (C5H9ClO) | XVRIEWDDMODMGA-UHFFFAOYSA-N | CC(=O)CCCCl | 368 | 110 | 2 | 1 |
| 1-Chloro-3-pentanone (C5H9ClO) | APNSUHRNUVUCIP-UHFFFAOYSA-N | CCC(=O)CCCl | 467 | 1413 | 2 | 1 |
| Pentanoyl chloride (C5H9ClO) | XGISHOFUAFNYQF-UHFFFAOYSA-N | CCCCC(=O)Cl | 20 | 9999 | 2 | 1 |
| 3-Chloro-2,2-dimethyl-1-propanol (C5H11ClO) | CAZPRAORHCOIHC-UHFFFAOYSA-N | CC(C)(CO)CCl | 0 | 10 | 2 | 2 |
| α-Chloroisovaleronitrile (C5H8ClN) | GMPPCOCINGLLFH-UHFFFAOYSA-N | CC(C)C(C#N)Cl | 0 | 113 | 2 | 1 |
| Butane, 1-chloro-2-methyl-, (S)- (C5H11Cl) | IWAKWOFEHSYKSI-RXMQYKEDSA-N | CC[C@@H](C)CCl | 10 | 90 | 2 | 2 |
| Butanoyl chloride, 3-methyl- (C5H9ClO) | ISULZYQDGYXDFW-UHFFFAOYSA-N | CC(C)CC(=O)Cl | 0 | 5539 | 2 | 1 |
| Ethylmethylacetylchloride (C5H9ClO) | XRPVXVRWIDOORM-UHFFFAOYSA-N | CCC(C)C(=O)Cl | 10 | 1629 | 2 | 1 |
| Pivalyl chloride (C5H9ClO) | JVSFQJZRHXAUGT-UHFFFAOYSA-N | CC(C)(C)C(=O)Cl | 0 | 189 | 2 | 1 |
| 1,5-Dichloropentane (C5H10Cl2) | LBKDGROORAKTLC-UHFFFAOYSA-N | C(CCCl)CCCl | 0 | 60 | 2 | 2 |
| 1,4-Dichloropentane (C5H10Cl2) | IJZUPZAYWWVHIO-UHFFFAOYSA-N | CC(CCCCl)Cl | 0 | 630 | 2 | 1 |
| 1,4-Dichloro-2-methylbutane (C5H10Cl2) | OUSZUUNUORQHDW-UHFFFAOYSA-N | CC(CCCl)CCl | 19 | 194 | 2 | 1 |
| 1,2-Dichloropentane (C5H10Cl2) | PPLBPDUKNRCHGG-UHFFFAOYSA-N | CCCC(CCl)Cl | 0 | 0 | 2 | 2 |
| 1,1-Dichloropentane (C5H10Cl2) | PGEVTVXEERFABN-UHFFFAOYSA-N | CCCCC(Cl)Cl | 20 | 360 | 2 | 1 |
| 1,3-Dichloro-3-methylbutane (C5H10Cl2) | QGCOTJZDMVMLRQ-UHFFFAOYSA-N | CC(C)(CCCl)Cl | 0 | 2423 | 2 | 1 |
| 2,4-Dichloropentane (C5H10Cl2) | DYGOBGYJRRKFEN-UHFFFAOYSA-N | CC(CC(C)Cl)Cl | 0 | 50 | 2 | 2 |
| 1,3-Dichloro-2-methylbutane, threo (C5H10Cl2) | MCAJTVAYCDZHHF-UHFFFAOYSA-N | CC(CCl)C(C)Cl | 5 | 179 | 2 | 1 |
| 1,1-Dichloro-3-methylbutane (C5H10Cl2) | VXEMJASOVHTHLL-UHFFFAOYSA-N | CC(C)CC(Cl)Cl | 23 | 0 | 2 | 2 |
| 1,2-Dichloro-2-methylbutane (C5H10Cl2) | KVPMOKIQASUYOV-UHFFFAOYSA-N | CCC(C)(CCl)Cl | 0 | 325 | 2 | 1 |
| 2,3-Dichloropentane (C5H10Cl2) | HVFJQRZGBBKTPL-UHFFFAOYSA-N | CCC(C(C)Cl)Cl | 0 | 110 | 2 | 1 |
| 2,2-Dichloropentane (C5H10Cl2) | MNXVHBFCELLVBA-UHFFFAOYSA-N | CCCC(C)(Cl)Cl | 0 | 2720 | 2 | 1 |
| 2,3-Dichloro-2-methylbutane (C5H10Cl2) | TXTORVZCRUFBBO-UHFFFAOYSA-N | CC(C(C)(C)Cl)Cl | 3 | 923 | 2 | 1 |
| 2,2-Dichloro-3-methylbutane (C5H10Cl2) | WIQMOFSSJHPXIK-UHFFFAOYSA-N | CC(C)C(C)(Cl)Cl | 13 | 1761 | 2 | 1 |
| 1-Chlorohexane (C6H13Cl) | MLRVZFYXUZQSRU-UHFFFAOYSA-N | CCCCCCCl | 0 | 70 | 2 | 2 |
| Hexane, 2-chloro- (C6H13Cl) | GLCIPJOIEVLTPR-UHFFFAOYSA-N | CCCCC(C)Cl | 0 | 989 | 2 | 1 |
| 3-Chlorohexane (C6H13Cl) | BXSMMAVTEURRGG-UHFFFAOYSA-N | CCCC(CC)Cl | 0 | 1171 | 2 | 1 |
| Butane, 1-chloro-3,3-dimethyl- (C6H13Cl) | XGCKOSFYXBAPQM-UHFFFAOYSA-N | CC(C)(C)CCCl | 0 | 0 | 2 | 2 |
| 2-Chloro-4-methylpentane (C6H13Cl) | WIMBRKMSNRCNMP-UHFFFAOYSA-N | CC(C)CC(C)Cl | 0 | 564 | 2 | 1 |
| Pentane, 3-chloro-3-methyl- (C6H13Cl) | SGWJUIFOPCZXMR-UHFFFAOYSA-N | CCC(C)(CC)Cl | 17 | 5148 | 2 | 1 |
| 2-Chloro-2-methylpentane (C6H13Cl) | NXXHAWKBICGUCK-UHFFFAOYSA-N | CCCC(C)(C)Cl | 0 | 5107 | 2 | 1 |
| Butane, 2-chloro-2,3-dimethyl- (C6H13Cl) | HEMQRALQJLCVBR-UHFFFAOYSA-N | CC(C)C(C)(C)Cl | 10 | 2679 | 2 | 1 |
| Allyl chloroformate (C4H5ClO2) | CAEWJEXPFKNBQL-UHFFFAOYSA-N | C=CCOC(=O)Cl | 35 | 184 | 2 | 1 |
| 2-Propenoic acid, 2-chloro-, methyl ester (C4H5ClO2) | AWJZTPWDQYFQPQ-UHFFFAOYSA-N | COC(=O)C(=C)Cl | 5265 | 2682 | 1 | 1 |
| Methyl (Z)-3-chloropropenoate (C4H5ClO2) | ZLDNFLVIPPOXQL-IHWYPQMZSA-N | COC(=O)/C=C\Cl | 690 | 2232 | 2 | 1 |
| Methyl (E)-3-chloropropenoate (C4H5ClO2) | ZLDNFLVIPPOXQL-NSCUHMNNSA-N | COC(=O)/C=C/Cl | 760 | 3062 | 2 | 1 |
| 1-Propene, 1,2,3,3-tetrachloro- (C3H2Cl4) | JUGQRTGGLWOBPG-UPHRSURJSA-N | C(=C(/C(Cl)Cl)\Cl)\Cl | 1601 | 9999 | 1 | 1 |
| 1-Propene, 1,1,3-trichloro-2-methyl- (C4H5Cl3) | GVFGURPOQVIHNM-UHFFFAOYSA-N | CC(=C(Cl)Cl)CCl | 2452 | 9999 | 1 | 1 |
| 1-Propene, 3,3,3-trichloro-2-methyl- (C4H5Cl3) | AIZNASPCDNJRSP-UHFFFAOYSA-N | CC(=C)C(Cl)(Cl)Cl | 2572 | 9999 | 1 | 1 |
| 3,3-Dimethylacryloyl chloride (C5H7ClO) | BDUBTLFQHNYXPC-UHFFFAOYSA-N | CC(=CC(=O)Cl)C | 60 | 9999 | 2 | 1 |
| 2-Hexene, 1-chloro- (C6H11Cl) | YTXXOKPWZPVIFH-SNAWJCMRSA-N | CCC/C=C/CCl | 1800 | 2800 | 1 | 1 |
| 1-Hexene, 1-chloro-, (E)- (C6H11Cl) | UZIBPOIXTCIHBH-AATRIKPKSA-N | CCCC/C=C/Cl | 2512 | 243 | 1 | 1 |
| Pyrimidine, 2-chloro- (C4H3ClN2) | UNCQVRBWJWWJBF-UHFFFAOYSA-N | C1=CN=C(N=C1)Cl | 9999 | 5779 | 1 | 1 |
| Chloropyrazine (C4H3ClN2) | GELVZYOEQVJIRR-UHFFFAOYSA-N | C1=CN=C(C=N1)Cl | 9999 | 9169 | 1 | 1 |
| 2-Chloropyridine (C5H4ClN) | OKDGRDCXVWSXDC-UHFFFAOYSA-N | C1=CC=NC(=C1)Cl | 8399 | 9999 | 1 | 1 |
| 3-Chloropyridine (C5H4ClN) | PWRBCZZQRRPXAB-UHFFFAOYSA-N | C1=CC(=CN=C1)Cl | 9999 | 7199 | 1 | 1 |
| 4-Chloropyridine (C5H4ClN) | PVMNPAUTCMBOMO-UHFFFAOYSA-N | C1=CN=CC=C1Cl | 9999 | 6529 | 1 | 1 |
| Chlorobenzene (C6H5Cl) | MVPPADPHJFYWMZ-UHFFFAOYSA-N | C1=CC=C(C=C1)Cl | 9999 | 4534 | 1 | 1 |
| 4-Chloro-1,3-dioxolan-2-one (C3H3ClO3) | OYOKPDLAMOMTEE-UHFFFAOYSA-N | C1C(OC(=O)O1)Cl | 369 | 9999 | 2 | 1 |
| 1H-1,2,4-Triazole, 3-chloro-5-methyl- (C3H4ClN3) | AEDKVRCTAMEXHJ-UHFFFAOYSA-N | CC1=NC(=NN1)Cl | 9706 | 309 | 1 | 1 |
| 1,3-Dioxolane, 2-(chloromethyl)- (C4H7ClO2) | IKZOMJGRWIOEDP-UHFFFAOYSA-N | C1COC(O1)CCl | 10 | 50 | 2 | 2 |
| 3,3,3-Trichloro-2-epoxypropane (C3H3Cl3O) | VFEXYZINKMLLAK-UHFFFAOYSA-N | C1C(O1)C(Cl)(Cl)Cl | 30 | 8869 | 2 | 1 |
| Tetrahydrofurfuryl chloride (C5H9ClO) | IVJLGIMHHWKRAN-UHFFFAOYSA-N | C1CC(OC1)CCl | 80 | 0 | 2 | 2 |
| 2H-Pyran, 4-chlorotetrahydro- (C5H9ClO) | DHRSKOBIDIDMJZ-UHFFFAOYSA-N | C1COCCC1Cl | 1681 | 511 | 1 | 1 |
| Cyclobutanecarboxylic acid chloride (C5H7ClO) | JFWMYCVMQSLLOO-UHFFFAOYSA-N | C1CC(C1)C(=O)Cl | 12 | 2614 | 2 | 1 |
| Cyclopentane, 1,2-dichloro-, trans- (C5H8Cl2) | QPIRTTQWLDPXBN-WHFBIAKZSA-N | C1C[C@@H]([C@H](C1)Cl)Cl | 95 | 188 | 2 | 1 |
| Cyclopropane, 1,1-dichloro-2,2-dimethyl- (C5H8Cl2) | NNBWTSXEHTTWMR-UHFFFAOYSA-N | CC1(CC1(Cl)Cl)C | 1952 | 9999 | 1 | 1 |
| Chlorocyclohexane (C6H11Cl) | UNFUYWDGSFDHCW-UHFFFAOYSA-N | C1CCC(CC1)Cl | 199 | 2929 | 2 | 1 |
| 2-Thiazolamine, 5-chloro- (C3H3ClN2S) | SWQWTDAWUSBMGA-UHFFFAOYSA-N | C1=C(SC(=N1)N)Cl | 9999 | 2569 | 1 | 1 |
| 5-Chloro-1-methylimidazole (C4H5ClN2) | NYDGOZPYEABERA-UHFFFAOYSA-N | CN1C=NC=C1Cl | 9999 | 3337 | 1 | 1 |
| 4,5-Dichloroimidazole (C3H2Cl2N2) | CHUPRLGXGZETTE-UHFFFAOYSA-N | C1=NNC(=C1Cl)Cl | 9999 | 791 | 1 | 1 |
| Cyclopropene, tetrachloro- (C3Cl4) | BLZOHTXDDOAASQ-UHFFFAOYSA-N | C1(=C(C1(Cl)Cl)Cl)Cl | 619 | 9999 | 2 | 1 |
| 3-Chlorocyclohexene (C6H9Cl) | LNGQLHZIYFQUIR-UHFFFAOYSA-N | C1CC=CC(C1)Cl | 1488 | 9999 | 1 | 1 |
| 1-chlorocyclohex-1-ene (C6H9Cl) | BUAKPITZELZWNI-UHFFFAOYSA-N | C1CCC(=CC1)Cl | 2329 | 9999 | 1 | 1 |
| Thiophene, 2,5-dichloro- (C4H2Cl2S) | FGYBDASKYMSNCX-UHFFFAOYSA-N | C1=C(SC(=C1)Cl)Cl | 9999 | 2649 | 1 | 1 |
| Trichloromethylsulfonyl chloride (CCl4O2S) | ZCPSWAFANXCCOT-UHFFFAOYSA-N | C(S(=O)(=O)Cl)(Cl)(Cl)Cl | 0 | 0 | 2 | 2 |
| Methyl trichloroacetate (C3H3Cl3O2) | VHFUHRXYRYWELT-UHFFFAOYSA-N | COC(=O)C(Cl)(Cl)Cl | 0 | 629 | 2 | 1 |
| Hexachloroethane (C2Cl6) | VHHHONWQHHHLTI-UHFFFAOYSA-N | C(C(Cl)(Cl)Cl)(Cl)(Cl)Cl | 0 | 4350 | 2 | 1 |
| Propanoic acid, 2-chloro-, methyl ester, (S)- (C4H7ClO2) | JLEJCNOTNLZCHQ-GSVOUGTGSA-N | C[C@H](C(=O)OC)Cl | 236 | 2913 | 2 | 1 |
| Carbamic chloride, diethyl- (C5H10ClNO) | OFCCYDUUBNUJIB-UHFFFAOYSA-N | CCN(CC)C(=O)Cl | 4509 | 8439 | 1 | 1 |
| Disulfide, bis(2-chloroethyl) (C4H8Cl2S2) | XDFZUXHZXUFQOS-UHFFFAOYSA-N | C(CCl)SSCCCl | 3103 | 591 | 1 | 1 |
| Bis(β-chloroethyl) sulfoxide (C4H8Cl2OS) | NOMHBBFEJSVGSC-UHFFFAOYSA-N | C(CCl)S(=O)CCCl | 611 | 0 | 2 | 2 |
| Carbonochloridic acid, 3-chloropropyl ester (C4H6Cl2O2) | MTXMEFUEBCFWCY-UHFFFAOYSA-N | C(COC(=O)Cl)CCl | 0 | 10 | 2 | 2 |
| Bis(2-chloroethyl)methylamine (C5H11Cl2N) | HAWPXGHAZFHHAD-UHFFFAOYSA-N | CN(CCCl)CCCl | 260 | 480 | 2 | 1 |
| Propanoic acid, 2,3-dichloro-, methyl ester (C4H6Cl2O2) | OFHMODDLBXETIK-UHFFFAOYSA-N | COC(=O)C(CCl)Cl | 10 | 1539 | 2 | 1 |
| Propanoic acid, 3,3-dichloro-, methyl ester (C4H6Cl2O2) | WGVLOKHINHKFDO-UHFFFAOYSA-N | COC(=O)CC(Cl)Cl | 3800 | 9999 | 1 | 1 |
| Ethyl dichloroacetate (C4H6Cl2O2) | IWYBVQLPTCMVFO-UHFFFAOYSA-N | CCOC(=O)C(Cl)Cl | 0 | 0 | 2 | 2 |
| Propanoic acid, 2,2-dichloro-, methyl ester (C4H6Cl2O2) | RDYUJBOUMXIDPY-UHFFFAOYSA-N | CC(C(=O)OC)(Cl)Cl | 20 | 831 | 2 | 1 |
| 1,1,1,3-Tetrachloroacetone (C3H2Cl4O) | MSZQBKOLHPDFFD-UHFFFAOYSA-N | C(C(=O)C(Cl)(Cl)Cl)Cl | 30 | 420 | 2 | 1 |
| 1,1,3,3-Tetrachloroacetone (C3H2Cl4O) | DJWVKJAGMVZYFP-UHFFFAOYSA-N | C(C(=O)C(Cl)Cl)(Cl)Cl | 279 | 90 | 2 | 2 |
| 1,1,2,3,3-Pentachloropropane (C3H3Cl5) | PANVCEBTPSTUEL-UHFFFAOYSA-N | C(C(Cl)Cl)(C(Cl)Cl)Cl | 0 | 149 | 2 | 1 |
| 2-Propanol, 1-chloro-3-ethoxy- (C5H11ClO2) | XHIINWKFCZSGNY-UHFFFAOYSA-N | CCOCC(CCl)O | 4 | 42 | 2 | 2 |
| Butanoic acid, 4-chloro-, methyl ester (C5H9ClO2) | ZZUYIRISBMWFMV-UHFFFAOYSA-N | COC(=O)CCCCl | 7 | 890 | 2 | 1 |
| 1-Propanol, 3-chloro-, acetate (C5H9ClO2) | KPOHQIPNNIMWRL-UHFFFAOYSA-N | CC(=O)OCCCCl | 10 | 200 | 2 | 1 |
| Propanoic acid, 3-chloro-, ethyl ester (C5H9ClO2) | ZCLGVXACCAZJOX-UHFFFAOYSA-N | CCOC(=O)CCCl | 209 | 3699 | 2 | 1 |
| Propyl chloroacetate (C5H9ClO2) | QJZNRCWAXUGABH-UHFFFAOYSA-N | CCCOC(=O)CCl | 0 | 50 | 2 | 2 |
| Butyl chloroformate (C5H9ClO2) | NRDQFWXVTPZZAZ-UHFFFAOYSA-N | CCCCOC(=O)Cl | 0 | 0 | 2 | 2 |
| Diethyl-(2-chloro-ethyl)-amine (C6H14ClN) | YMDNODNLFSHHCV-UHFFFAOYSA-N | CCN(CC)CCCl | 1079 | 419 | 1 | 1 |
| Butanoic acid, 3-chloro-, methyl ester (C5H9ClO2) | HDQHWBXUKZBPDN-UHFFFAOYSA-N | CC(CC(=O)OC)Cl | 60 | 9999 | 2 | 1 |
| Isopropyl chloroacetate (C5H9ClO2) | VODRWDBLLGYRJT-UHFFFAOYSA-N | CC(C)OC(=O)CCl | 45 | 74 | 2 | 2 |
| isobutyl chloroformate (C5H9ClO2) | YOETUEMZNOLGDB-UHFFFAOYSA-N | CC(C)COC(=O)Cl | 0 | 0 | 2 | 2 |
| Butanoic acid, 2-chloro-, methyl ester (C5H9ClO2) | BHQQXAOBIZQEGI-UHFFFAOYSA-N | CCC(C(=O)OC)Cl | 100 | 1501 | 2 | 1 |
| ethyl 2-chloropropionate (C5H9ClO2) | JEAVBVKAYUCPAQ-UHFFFAOYSA-N | CCOC(=O)C(C)Cl | 140 | 21 | 2 | 2 |
| Propane, 1-chloro-3-((2-chloroethyl)thio)- (C5H10Cl2S) | NXRPRUPNFDEDSY-UHFFFAOYSA-N | C(CSCCCl)CCl | 2452 | 1071 | 1 | 1 |
| Pentanoic acid, 5-chloro- (C5H9ClO2) | YSXDKDWNIPOSMF-UHFFFAOYSA-N | C(CCCl)CC(=O)O | 0 | 837 | 2 | 1 |
| Propanamide, 3-chloro-2,2-dimethyl- (C5H10ClNO) | CCFNEVMWSYAQKL-UHFFFAOYSA-N | CC(C)(CCl)C(=O)N | 184 | 2977 | 2 | 1 |
| 3-Chloro-2,4-pentanedione (C5H7ClO2) | VLRGXXKFHVJQOL-UHFFFAOYSA-N | CC(=O)C(C(=O)C)Cl | 6349 | 399 | 1 | 1 |
| 2,2-Bis(chloromethyl)-1-propanol (C5H10Cl2O) | DOANJBQUOFJQHC-UHFFFAOYSA-N | CC(CO)(CCl)CCl | 0 | 0 | 2 | 2 |
| 2-Chloromethyl-1,3-dichloro-2-methylpropane (C5H9Cl3O) | BYXOMFFBGDPXHB-UHFFFAOYSA-N | CC(CCl)(CCl)CCl | 0 | 0 | 2 | 2 |
| 1-Hexanol, 6-chloro- (C6H13ClO) | JNTPTNNCGDAGEJ-UHFFFAOYSA-N | C(CCCCl)CCO | 10 | 10 | 2 | 2 |
| hexanoyl chloride (C6H11ClO) | YWGHUJQYGPDNKT-UHFFFAOYSA-N | CCCCCC(=O)Cl | 10 | 8369 | 2 | 1 |
| 2-Ethylbutiryl chloride (C6H11ClO) | SMUKODJVMQOSAB-UHFFFAOYSA-N | CCC(CC)C(=O)Cl | 0 | 1682 | 2 | 1 |
| 2-methylvaleryl chloride (C6H11ClO) | MFIQXAVMTLKUJR-UHFFFAOYSA-N | CCCC(C)C(=O)Cl | 0 | 1410 | 2 | 1 |
| 3-Chloro-4-methyl-2-pentanol (C6H13ClO) | MWICNGBDJPEOGW-UHFFFAOYSA-N | CC(C)C(C(C)O)Cl | 0 | 29 | 2 | 2 |
| 2-Butanone, 1-chloro-3,3-dimethyl- (C6H11ClO) | ULSAJQMHTGKPIY-UHFFFAOYSA-N | CC(C)(C)C(=O)CCl | 109 | 0 | 2 | 2 |
| 1,6-Dichlorohexane (C6H12Cl2) | OVISMSJCKCDOPU-UHFFFAOYSA-N | C(CCCCl)CCCl | 0 | 50 | 2 | 2 |
| 1,2-Dichlorohexane (C6H12Cl2) | ZHFXSKJYCSWRJA-UHFFFAOYSA-N | CCCCC(CCl)Cl | 0 | 0 | 2 | 2 |
| 3-Heptyne, 7-chloro- (C7H11Cl) | NFSWUGZOVBYRKR-UHFFFAOYSA-N | CCC#CCCCCl | 2046 | 7362 | 1 | 1 |
| 1-Chloroheptane (C7H15Cl) | DZMDPHNGKBEVRE-UHFFFAOYSA-N | CCCCCCCCl | 15 | 3 | 2 | 2 |
| Hexane, 1-chloro-5-methyl- (C7H15Cl) | YESHSLGUAPTMLI-UHFFFAOYSA-N | CC(C)CCCCCl | 0 | 0 | 2 | 2 |
| 2-Chloroheptane (C7H15Cl) | PTSLUOSUHFGQHV-UHFFFAOYSA-N | CCCCCC(C)Cl | 0 | 259 | 2 | 1 |
| 2-Chloro-2-methylhexane (C7H15Cl) | KBOBQLJBYKKAPN-UHFFFAOYSA-N | CCCCC(C)(C)Cl | 0 | 1178 | 2 | 1 |
| Hexane, 3-chloro-3-methyl- (C7H15Cl) | UTKDCNDVTOWUHW-UHFFFAOYSA-N | CCCC(C)(CC)Cl | 0 | 871 | 2 | 1 |
| 2-Chloro-2,4-dimethylpentane (C7H15Cl) | DQOHPSPKVODKLV-UHFFFAOYSA-N | CC(C)CC(C)(C)Cl | 3 | 5451 | 2 | 1 |
| Methyl 3,3-dichloropropenoate (C4H4Cl2O2) | YPCKXQGCSGMTIR-UHFFFAOYSA-N | COC(=O)C=C(Cl)Cl | 990 | 610 | 1 | 1 |
| 2-Propenoic acid, 2-chloroethyl ester (C5H7ClO2) | WHBAYNMEIXUTJV-UHFFFAOYSA-N | C=CC(=O)OCCCl | 0 | 1460 | 2 | 1 |
| Chloroacetic acid allyl ester (C5H7ClO2) | VMBJJCDVORDOCF-UHFFFAOYSA-N | C=CCOC(=O)CCl | 10 | 99 | 2 | 2 |
| Fumaryl chloride (C4H2Cl2O2) | ZLYYJUJDFKGVKB-OWOJBTEDSA-N | C(=C/C(=O)Cl)\C(=O)Cl | 0 | 5669 | 2 | 1 |
| 1,1,5-Trichloro-1-pentene (C5H7Cl3) | WIBRQEFYLKNRTM-UHFFFAOYSA-N | C(CC=C(Cl)Cl)CCl | 1041 | 631 | 1 | 1 |
| Pyrimidine, 4,6-dichloro- (C4H2Cl2N2) | XJPZKYIHCLDXST-UHFFFAOYSA-N | C1=C(N=CN=C1Cl)Cl | 9999 | 6856 | 1 | 1 |
| Pyrimidine, 2,4-dichloro- (C4H2Cl2N2) | BTTNYQZNBZNDOR-UHFFFAOYSA-N | C1=CN=C(N=C1Cl)Cl | 8509 | 9999 | 1 | 1 |
| 2,6-Dichloropyrazine (C4H2Cl2N2) | LSEAAPGIZCDEEH-UHFFFAOYSA-N | C1=C(N=C(C=N1)Cl)Cl | 9999 | 6919 | 1 | 1 |
| 2,3-dichloropyrazine (C4H2Cl2N2) | MLCNOCRGSBCAGH-UHFFFAOYSA-N | C1=CN=C(C(=N1)Cl)Cl | 9999 | 9045 | 1 | 1 |
| Pyridazine, 3,6-dichloro- (C4H2Cl2N2) | GUSWJGOYDXFJSI-UHFFFAOYSA-N | C1=CC(=NN=C1Cl)Cl | 8539 | 20 | 1 | 2 |
| 2-Amino-5-chloropyridine (C5H5ClN2) | MAXBVGJEFDMHNV-UHFFFAOYSA-N | C1=CC(=NC=C1Cl)N | 9999 | 1010 | 1 | 1 |
| 4-Amino-2-chloropyridine (C5H5ClN2) | BLBDTBCGPHPIJK-UHFFFAOYSA-N | C1=CN=C(C=C1N)Cl | 9999 | 6119 | 1 | 1 |
| 3-Amino-2-chloropyridine (C5H5ClN2) | MEQBJJUWDCYIAB-UHFFFAOYSA-N | C1=CC(=C(N=C1)Cl)N | 9999 | 1859 | 1 | 1 |
| 6-Chloro-2-pyridinol (C5H4ClNO) | CLNNBQDAAGDAHI-UHFFFAOYSA-N | C1=CC(=O)NC(=C1)Cl | 8986 | 9975 | 1 | 1 |
| 5-Chloro-2-pyridinol (C5H4ClNO) | SZFUWUOHDRMCKD-UHFFFAOYSA-N | C1=CC(=O)NC=C1Cl | 7237 | 631 | 1 | 1 |
| 2-Chloro-3-pyridinol (C5H4ClNO) | RSOPTYAZDFSMTN-UHFFFAOYSA-N | C1=CC(=C(N=C1)Cl)O | 9869 | 891 | 1 | 1 |
| Pyridine, 2,6-dichloro- (C5H3Cl2N) | FILKGCRCWDMBKA-UHFFFAOYSA-N | C1=CC(=NC(=C1)Cl)Cl | 8130 | 9999 | 1 | 1 |
| Pyridine, 3,5-dichloro- (C5H3Cl2N) | WPGHPGAUFIJVJF-UHFFFAOYSA-N | C1=C(C=NC=C1Cl)Cl | 9999 | 5049 | 1 | 1 |
| Benzenethiol, 4-chloro- (C6H5ClS) | VZXOZSQDJJNBRC-UHFFFAOYSA-N | C1=CC(=CC=C1S)Cl | 9999 | 5239 | 1 | 1 |
| Benzenethiol, 3-chloro- (C6H5ClS) | CQJDYPZUDYXHLM-UHFFFAOYSA-N | C1=CC(=CC(=C1)Cl)S | 9999 | 5769 | 1 | 1 |
| Benzenethiol, 2-chloro- (C6H5ClS) | PWOBDMNCYMQTCE-UHFFFAOYSA-N | C1=CC=C(C(=C1)S)Cl | 9999 | 4709 | 1 | 1 |
| 4-Chloroaniline (C6H6ClN) | QSNSCYSYFYORTR-UHFFFAOYSA-N | C1=CC(=CC=C1N)Cl | 9999 | 1860 | 1 | 1 |
| 3-Chloroaniline (C6H6ClN) | PNPCRKVUWYDDST-UHFFFAOYSA-N | C1=CC(=CC(=C1)Cl)N | 9999 | 2060 | 1 | 1 |
| 2-Chloroaniline (C6H6ClN) | AKCRQHGQIJBRMN-UHFFFAOYSA-N | C1=CC=C(C(=C1)N)Cl | 9999 | 2019 | 1 | 1 |
| 4-Chlorophenol (C6H5ClO) | WXNZTHHGJRFXKQ-UHFFFAOYSA-N | C1=CC(=CC=C1O)Cl | 9999 | 489 | 1 | 1 |
| 3-Chlorophenol (C6H5ClO) | HORNXRXVQWOLPJ-UHFFFAOYSA-N | C1=CC(=CC(=C1)Cl)O | 9999 | 482 | 1 | 1 |
| Phenol, 2-chloro- (C6H5ClO) | ISPYQTSUDJAMAB-UHFFFAOYSA-N | C1=CC=C(C(=C1)O)Cl | 9999 | 270 | 1 | 1 |
| 1,4-Dichlorobenzene (C6H4Cl2) | OCJBOOLMMGQPQU-UHFFFAOYSA-N | C1=CC(=CC=C1Cl)Cl | 9999 | 2689 | 1 | 1 |
| 1,3-Dichlorobenzene (C6H4Cl2) | ZPQOPVIELGIULI-UHFFFAOYSA-N | C1=CC(=CC(=C1)Cl)Cl | 9999 | 4510 | 1 | 1 |
| 1,2-Dichlorobenzene (C6H4Cl2) | RFFLAFLAYFXFSW-UHFFFAOYSA-N | C1=CC=C(C(=C1)Cl)Cl | 9999 | 3409 | 1 | 1 |
| 4,5-Dichloro-1,3-dioxolan-2-one (C3H2Cl2O3) | BETICXVUVYXEJX-UHFFFAOYSA-N | C1(C(OC(=O)O1)Cl)Cl | 26 | 988 | 2 | 1 |
| 2-Chloro-3-methylpyrazine (C5H5ClN2) | WZHWPZQQPWKEAV-UHFFFAOYSA-N | CC1=NC=CN=C1Cl | 9999 | 7860 | 1 | 1 |
| 3-Chloro-6-methylpyridazine (C5H5ClN2) | PRORLQAJNJMGAR-UHFFFAOYSA-N | CC1=NN=C(C=C1)Cl | 4377 | 39 | 1 | 2 |
| Pyridine, 2-chloro-6-methyl- (C6H6ClN) | GXZDYRYYNXYPMQ-UHFFFAOYSA-N | CC1=NC(=CC=C1)Cl | 9999 | 3589 | 1 | 1 |
| Benzyl chloride (C7H7Cl) | KCXMKQUNVWSEMD-UHFFFAOYSA-N | C1=CC=C(C=C1)CCl | 2489 | 9999 | 1 | 1 |
| 4-Chlorotoluene (C7H7Cl) | NPDACUSDTOMAMK-UHFFFAOYSA-N | CC1=CC=C(C=C1)Cl | 3003 | 9999 | 1 | 1 |
| 3-Chlorotoluene (C7H7Cl) | OSOUNOBYRMOXQQ-UHFFFAOYSA-N | CC1=CC(=CC=C1)Cl | 3383 | 9999 | 1 | 1 |
| 2-Chlorotoluene (C7H7Cl) | IBSQPLPBRSHTTG-UHFFFAOYSA-N | CC1=CC=CC=C1Cl | 3603 | 9999 | 1 | 1 |
| 1,4-Dioxane, 2,3-dichloro- (C4H6Cl2O2) | ZOZUXFQYIYUIND-UHFFFAOYSA-N | C1COC(C(O1)Cl)Cl | 499 | 5169 | 2 | 1 |
| Cyclopropane, pentachloro- (C3HCl5) | IACJMSLMMMSESC-UHFFFAOYSA-N | C1(C(C1(Cl)Cl)(Cl)Cl)Cl | 0 | 7770 | 2 | 1 |
| 2-(Chloromethyl)tetrahydropyran (C6H11ClO) | PPYKTTGONDVGPX-UHFFFAOYSA-N | C1CCOC(C1)CCl | 70 | 0 | 2 | 2 |
| 2,2-Dichlorocyclopropylacetonitrile (C5H5Cl2N) | METYSMJJRFRDPP-UHFFFAOYSA-N | C1C(C1(Cl)Cl)CC#N | 18 | 573 | 2 | 1 |
| 2-Chlorocyclohexanol (C6H11ClO) | NYEWDMNOXFGGDX-UHFFFAOYSA-N | C1CCC(C(C1)O)Cl | 489 | 155 | 2 | 1 |
| Cyclohexanol, 2-chloro-, cis (C6H11ClO) | NYEWDMNOXFGGDX-NTSWFWBYSA-N | C1CC[C@@H]([C@@H](C1)O)Cl | 300 | 1200 | 2 | 1 |
| Cyclohexanol, 4-chloro, trans (C6H11ClO) | HVPIAXWCSPHTAY-IZLXSQMJSA-N | C1CC(CCC1O)Cl | 140 | 110 | 2 | 1 |
| 4-Chlorocyclohexanol (C6H11ClO) | HVPIAXWCSPHTAY-UHFFFAOYSA-N | C1CC(CCC1O)Cl | 76 | 571 | 2 | 1 |
| trans-2-chlorocyclohexanol (C6H11ClO) | NYEWDMNOXFGGDX-PHDIDXHHSA-N | C1CC[C@H]([C@@H](C1)O)Cl | 2700 | 200 | 1 | 1 |
| Cyclohexanone, 2-chloro- (C6H9ClO) | CCHNWURRBFGQCD-UHFFFAOYSA-N | C1CCC(=O)C(C1)Cl | 2379 | 2471 | 1 | 1 |
| cyclopentanecarbonyl chloride (C6H9ClO) | WEPUZBYKXNKSDH-UHFFFAOYSA-N | C1CCC(C1)C(=O)Cl | 373 | 2371 | 2 | 1 |
| cis-1,2-Dichlorocyclohexane (C6H10Cl2) | GZEZIBFVJYNETN-OLQVQODUSA-N | C1CC[C@@H]([C@@H](C1)Cl)Cl | 105 | 74 | 2 | 2 |
| 1,2-Dichlorocyclohexane (C6H10Cl2) | GZEZIBFVJYNETN-UHFFFAOYSA-N | C1CCC(C(C1)Cl)Cl | 119 | 99 | 2 | 2 |
| cis-1,4-Dichlorocyclohexane (C6H10Cl2) | WQTINZDWAXJLGH-OLQVQODUSA-N | C1CC(CCC1Cl)Cl | 200 | 600 | 2 | 1 |
| trans-1,4-Dichlorocyclohexane (C6H10Cl2) | WQTINZDWAXJLGH-IZLXSQMJSA-N | C1CC(CCC1Cl)Cl | 260 | 0 | 2 | 2 |
| 1,1-Dichlorocyclohexane (C6H10Cl2) | LQRDJCBZCLUBRY-UHFFFAOYSA-N | C1CCC(CC1)(Cl)Cl | 0 | 3000 | 2 | 1 |
| trans-1,2-Dichlorocyclohexane (C6H10Cl2) | GZEZIBFVJYNETN-PHDIDXHHSA-N | C1CC[C@H]([C@@H](C1)Cl)Cl | 401 | 141 | 2 | 1 |
| 1-Chloro-4-methylcyclohexane (C7H13Cl) | KNEUJTFLMQRIFD-UHFFFAOYSA-N | CC1CCC(CC1)Cl | 106 | 665 | 2 | 1 |
| 1H-Imidazole,2-methyl,4,5-dichloro (C4H4Cl2N2) | ZWRYWVBALYOSPQ-UHFFFAOYSA-N | CC1=NC(=C(N1)Cl)Cl | 9999 | 1449 | 1 | 1 |
| Isoxazole-5-carbonyl chloride (C4H2ClNO2) | NASLINFISOTVJJ-UHFFFAOYSA-N | C1=C(ON=C1)C(=O)Cl | 1001 | 9999 | 1 | 1 |
| 5-Chloro-1,3-dimethylpyrazole (C5H7ClN2) | DDUSLFAWARYAPR-UHFFFAOYSA-N | CC1=NN(C(=C1)Cl)C | 9999 | 6589 | 1 | 1 |
| 2-Thiophenecarbonyl chloride (C5H3ClOS) | QIQITDHWZYEEPA-UHFFFAOYSA-N | C1=CSC(=C1)C(=O)Cl | 1922 | 9999 | 1 | 1 |
| 2-Furancarbonyl chloride (C5H3ClO2) | OFTKFKYVSBNYEC-UHFFFAOYSA-N | C1=COC(=C1)C(=O)Cl | 1599 | 9999 | 1 | 1 |
| 2-Thiophenecarboxaldehyde, 5-chloro- (C5H3ClOS) | VWYFITBWBRVBSW-UHFFFAOYSA-N | C1=C(SC(=C1)Cl)C=O | 8500 | 0 | 1 | 2 |
| Bicyclo[2.2.1]heptane, 2-chloro- (C7H11Cl) | PJWBUKHIZPKRJF-UHFFFAOYSA-N | C1CC2CC1CC2Cl | 846 | 2020 | 1 | 1 |
| Methyl N-chloroacetylcarbamate (C4H6ClNO3) | SYWSERLZKPGKDI-UHFFFAOYSA-N | COC(=O)NC(=O)CCl | 539 | 3916 | 2 | 1 |
| Phosphinic acid, bis(chloromethyl),- ethyl ester (C4H9Cl2O2P) | ZOQQHTTYHSLXKY-UHFFFAOYSA-N | CCOP(=O)(CCl)CCl | 817 | 0 | 1 | 2 |
| Trichloroacetyl isocyanate (C3Cl3NO2) | GRNOZCCBOFGDCL-UHFFFAOYSA-N | C(=NC(=O)C(Cl)(Cl)Cl)=O | 0 | 319 | 2 | 1 |
| 2,2,2-Trichloro-N,N-dimethylacetamide (C4H6Cl3NO) | WGBMKQSRUSKSOM-UHFFFAOYSA-N | CN(C)C(=O)C(Cl)(Cl)Cl | 87 | 24 | 2 | 2 |
| Carbonochloridic acid, 2,2,2-trichloroethyl ester (C3H2Cl4O2) | LJCZNYWLQZZIOS-UHFFFAOYSA-N | C(C(Cl)(Cl)Cl)OC(=O)Cl | 0 | 459 | 2 | 1 |
| Trisulfide, bis(2-chloroethyl) (C4H8Cl2S3) | XHODIRQQNWHUGV-UHFFFAOYSA-N | C(CCl)SSSCCCl | 5945 | 330 | 1 | 1 |
| bis-(2-Chloroethylthio)methane (C5H10Cl2S2) | RKTJTTAEKCRXNL-UHFFFAOYSA-N | C(CCl)SCSCCCl | 1621 | 0 | 1 | 2 |
| Bis(2-chloroethyl) sulfone (C4H8Cl2O2S) | LUYAMNYBNTVQJG-UHFFFAOYSA-N | C(CCl)S(=O)(=O)CCCl | 0 | 0 | 2 | 2 |
| Bis(2-chloroethoxy)methane (C5H10Cl2O2) | NLXGURFLBLRZRO-UHFFFAOYSA-N | C(CCl)OCOCCCl | 0 | 0 | 2 | 2 |
| Diglycolyl chloride (C4H4Cl2O3) | GTZXSBQCNBNWPK-UHFFFAOYSA-N | C(C(=O)Cl)OCC(=O)Cl | 0 | 0 | 2 | 2 |
| Chloroacetic anhydride (C4H4Cl2O3) | PNVPNXKRAUBJGW-UHFFFAOYSA-N | C(C(=O)OC(=O)CCl)Cl | 0 | 0 | 2 | 2 |
| Propanoic acid, 2,3,3-trichloro, methyl ester (C4H5Cl3O2) | VCJSVVVJNDJVPM-UHFFFAOYSA-N | COC(=O)C(C(Cl)Cl)Cl | 0 | 500 | 2 | 1 |
| Propanoic acid, 2,2,3-trichloro, methyl ester (C4H5Cl3O2) | SXUZAHHOLLSZRC-UHFFFAOYSA-N | COC(=O)C(CCl)(Cl)Cl | 10 | 140 | 2 | 1 |
| Ethyl trichloroacetate (C4H5Cl3O2) | SJMLNDPIJZBEKY-UHFFFAOYSA-N | CCOC(=O)C(Cl)(Cl)Cl | 0 | 669 | 2 | 1 |
| 2-Propanone, 1,1,1,3,3-pentachloro- (C3HCl5O) | RVSIFWBAGVMQKT-UHFFFAOYSA-N | C(C(=O)C(Cl)(Cl)Cl)(Cl)Cl | 33 | 122 | 2 | 1 |
| Butanoic acid, 4-chloro-3-oxo-, methyl ester (C5H7ClO3) | HFLMYYLFSNEOOT-UHFFFAOYSA-N | COC(=O)CC(=O)CCl | 769 | 40 | 2 | 2 |
| Ethane, 2-chloro-1,1-diethoxy- (C6H13ClO2) | OVXJWSYBABKZMD-UHFFFAOYSA-N | CCOC(CCl)OCC | 0 | 0 | 2 | 2 |
| Acetamide, 2-chloro-N,N-diethyl- (C6H12ClNO) | CQQUWTMMFMJEFE-UHFFFAOYSA-N | CCN(CC)C(=O)CCl | 1079 | 5409 | 1 | 1 |
| Butanoic acid, 2-chloro-3-oxo-, methyl ester (C5H7ClO3) | GYQRIAVRKLRQKP-UHFFFAOYSA-N | CC(=O)C(C(=O)OC)Cl | 169 | 0 | 2 | 2 |
| 3-chloropropyl chloroacetate (C5H8Cl2O2) | CWZVJVDIQWKNJX-UHFFFAOYSA-N | C(COC(=O)CCl)CCl | 11 | 1199 | 2 | 1 |
| Bis(2-chloroethyl)ethylamine (C6H13Cl2N) | UQZPGHOJMQTOHB-UHFFFAOYSA-N | CCN(CCCl)CCCl | 400 | 500 | 2 | 1 |
| 2,3-Dichloropropyl acetate (C5H8Cl2O2) | BVXPMFQVOWRQKD-UHFFFAOYSA-N | CC(=O)OCC(CCl)Cl | 0 | 30 | 2 | 2 |
| 2,4-Dichlorobutanoic acid, methyl ester (C5H8Cl2O2) | MIXXUSQBRXDJHD-UHFFFAOYSA-N | COC(=O)C(CCCl)Cl | 0 | 600 | 2 | 1 |
| 4,4-Dichlorobutanoic acid, methyl ester (C5H8Cl2O2) | FLTUWIKFBYMPIV-UHFFFAOYSA-N | COC(=O)CCC(Cl)Cl | 0 | 600 | 2 | 1 |
| Butanoic acid, 3,4-dichloro-, methyl ester (C5H8Cl2O2) | DVXYQNCRDDPGKM-UHFFFAOYSA-N | COC(=O)CC(CCl)Cl | 0 | 4233 | 2 | 1 |
| Ethyl-2,3-dichloropropionate (C5H8Cl2O2) | RNZPQAZETZXKCQ-UHFFFAOYSA-N | CCOC(=O)C(CCl)Cl | 0 | 340 | 2 | 1 |
| Propyl dichloroacetate (C5H8Cl2O2) | AHRFMMRLDGAHBM-UHFFFAOYSA-N | CCCOC(=O)C(Cl)Cl | 0 | 0 | 2 | 2 |
| Methyl threo-2,3-dichlorobutanoate (C5H8Cl2O2) | KJZMAXQPXRBYDC-UHFFFAOYSA-N | CC(C(C(=O)OC)Cl)Cl | 0 | 330 | 2 | 1 |
| Butanoic acid, 2,2-dichloro-, methyl ester (C5H8Cl2O2) | VAPJFYJBCCPOAV-UHFFFAOYSA-N | CCC(C(=O)OC)(Cl)Cl | 0 | 250 | 2 | 1 |
| 2-Propanol, 1-chloro-3-propoxy- (C6H13ClO2) | BJKFQZKSICPHSR-UHFFFAOYSA-N | CCCOCC(CCl)O | 0 | 30 | 2 | 2 |
| Pentanoic acid, 5-chloro-, methyl ester (C6H11ClO2) | JAVHFVJOWIQHII-UHFFFAOYSA-N | COC(=O)CCCCCl | 30 | 4974 | 2 | 1 |
| Ethyl 4-chlorobutanoate (C6H11ClO2) | OPXNFHAILOHHFO-UHFFFAOYSA-N | CCOC(=O)CCCCl | 30 | 851 | 2 | 1 |
| Pentyl chloroformate (C6H11ClO2) | XHRRYUDVWPPWIP-UHFFFAOYSA-N | CCCCCOC(=O)Cl | 0 | 0 | 2 | 2 |
| Butyl chloroacetate (C6H11ClO2) | YJRGMUWRPCPLNH-UHFFFAOYSA-N | CCCCOC(=O)CCl | 0 | 30 | 2 | 2 |
| Pentanoic acid, chloromethyl ester (C6H11ClO2) | ZYDSTVMBNBPPLQ-UHFFFAOYSA-N | CCCCC(=O)OCCl | 0 | 0 | 2 | 2 |
| 2-Propanol, 1-chloro-3-isopropoxy- (C6H13ClO2) | GQPJSBQMFFGZAU-UHFFFAOYSA-N | CC(C)OCC(CCl)O | 0 | 20 | 2 | 2 |
| Propanoic acid, 3-chloro-, 1-methylethyl ester (C6H11ClO2) | YPAMSUOJRFMSIA-UHFFFAOYSA-N | CC(C)OC(=O)CCCl | 0 | 20 | 2 | 2 |
| Isobutyl chloroacetate (C6H11ClO2) | QSGCJQBBHYWZHS-UHFFFAOYSA-N | CC(C)COC(=O)CCl | 0 | 10 | 2 | 2 |
| Monochloromethyl isopentanoate (C6H11ClO2) | GDUQVLASZBFABN-UHFFFAOYSA-N | CC(C)CC(=O)OCCl | 1101 | 0 | 1 | 2 |
| Acetic acid, chloro-, 1-methylpropyl ester (C6H11ClO2) | MCUHSYTVMNEJFT-UHFFFAOYSA-N | CCC(C)OC(=O)CCl | 20 | 70 | 2 | 2 |
| Propanoic acid, 2-chloro, propyl ester (C6H11ClO2) | XVDQGLUGZORASO-UHFFFAOYSA-N | CCCOC(=O)C(C)Cl | 355 | 0 | 2 | 2 |
| Methyl chloropivalate (C6H11ClO2) | CKYBSQGDQJXNSB-UHFFFAOYSA-N | CC(C)(CCl)C(=O)OC | 10 | 3353 | 2 | 1 |
| Acetic acid, chloro-, 1,1-dimethylethyl ester (C6H11ClO2) | KUYMVWXKHQSIAS-UHFFFAOYSA-N | CC(C)(C)OC(=O)CCl | 0 | 0 | 2 | 2 |
| Chloromethyl pivalate (C6H11ClO2) | GGRHYQCXXYLUTL-UHFFFAOYSA-N | CC(C)(C)C(=O)OCCl | 60 | 0 | 2 | 2 |
| Propane, 1,1'-thiobis[3-chloro- (C6H12Cl2S) | VDTHWBLOSZIMMN-UHFFFAOYSA-N | C(CSCCCCl)CCl | 1090 | 115 | 1 | 1 |
| Propane, 1,1'-oxybis[3-chloro- (C6H12Cl2O) | SMANNJALMIGASX-UHFFFAOYSA-N | C(COCCCCl)CCl | 10 | 10 | 2 | 2 |
| Pentanedioyl dichloride (C5H6Cl2O2) | YVOFTMXWTWHRBH-UHFFFAOYSA-N | C(CC(=O)Cl)CC(=O)Cl | 0 | 7760 | 2 | 1 |
| Bis(2-chloropropyl) sulfide (C6H12Cl2S) | AQHTWLYIOSPNMG-UHFFFAOYSA-N | CC(CSCC(C)Cl)Cl | 2913 | 1381 | 1 | 1 |
| bis(2-chloro-1-methylethyl) ether (C6H12Cl2O) | QCFYJCYNJLBDRT-UHFFFAOYSA-N | CC(CCl)OC(C)CCl | 0 | 10 | 2 | 2 |
| Dimethylmalonyl chloride (C5H6Cl2O2) | CJXQAYQWVNXIQE-UHFFFAOYSA-N | CC(C)(C(=O)Cl)C(=O)Cl | 0 | 5515 | 2 | 1 |
| Bis(2-chloroisopropyl) ether (C6H12Cl2O) | BULHJTXRZFEUDQ-UHFFFAOYSA-N | CC(C)(OC(C)(C)Cl)Cl | 0 | 0 | 2 | 2 |
| Pentane, 1,1,1,5-tetrachloro- (C5H8Cl4) | VZEWJVRACUZHQR-UHFFFAOYSA-N | C(CCCl)CC(Cl)(Cl)Cl | 0 | 1461 | 2 | 1 |
| Pentaerythrityl tetrachloride (C5H8Cl4) | KPZGRMZPZLOPBS-UHFFFAOYSA-N | C(C(CCl)(CCl)CCl)Cl | 44 | 62 | 2 | 2 |
| 6-Chlorohexanoyl chloride (C6H10Cl2O) | WZILXAPNPKMOSA-UHFFFAOYSA-N | C(CCC(=O)Cl)CCCl | 0 | 2962 | 2 | 1 |
| 2-Butanone, 1,1-dichloro-3,3-dimethyl- (C6H10Cl2O) | UDWZXMQIEHAAQT-UHFFFAOYSA-N | CC(C)(C)C(=O)C(Cl)Cl | 0 | 0 | 2 | 2 |
| Heptanoyl chloride (C7H13ClO) | UCVODTZQZHMTPN-UHFFFAOYSA-N | CCCCCCC(=O)Cl | 10 | 4449 | 2 | 1 |
| 2,2-Dimethylvaleroyl chloride (C7H13ClO) | NUBKYCCXDNYHLQ-UHFFFAOYSA-N | CCCC(C)(C)C(=O)Cl | 0 | 58 | 2 | 2 |
| 1,7-Dichloroheptane (C7H14Cl2) | PSEMXLIZFGUOGC-UHFFFAOYSA-N | C(CCCCl)CCCCl | 0 | 0 | 2 | 2 |
| 1-Chlorooctane (C8H17Cl) | CNDHHGUSRIZDSL-UHFFFAOYSA-N | CCCCCCCCCl | 0 | 10 | 2 | 2 |
| 2-Chlorooctane (C8H17Cl) | HKDCIIMOALDWHF-UHFFFAOYSA-N | CCCCCCC(C)Cl | 0 | 117 | 2 | 1 |
| 3-Chlorooctane (C8H17Cl) | FYNJWLOOBINARS-UHFFFAOYSA-N | CCCCCC(CC)Cl | 0 | 162 | 2 | 1 |
| Heptane, 3-(chloromethyl)- (C8H17Cl) | WLVCBAMXYMWGLJ-UHFFFAOYSA-N | CCCCC(CC)CCl | 4 | 9 | 2 | 2 |
| 4-Chlorooctane (C8H17Cl) | SRGMFDQMGOZQPO-UHFFFAOYSA-N | CCCCC(CCC)Cl | 0 | 152 | 2 | 1 |
| Heptane, 3-chloro-3-methyl- (C8H17Cl) | XGAHNVWATDXDPG-UHFFFAOYSA-N | CCCCC(C)(CC)Cl | 0 | 343 | 2 | 1 |
| Hexane, 2-chloro-2,5-dimethyl- (C8H17Cl) | SVRDRJZPEYTXKX-UHFFFAOYSA-N | CC(C)CCC(C)(C)Cl | 0 | 628 | 2 | 1 |
| 4-Chloro-2,4-dimethylhexane (C8H17Cl) | SYKUKUWLGOVGGT-UHFFFAOYSA-N | CCC(C)(CC(C)C)Cl | 0 | 627 | 2 | 1 |
| trans-2,3-dichloropropenoic acid, methyl ester (C4H4Cl2O2) | DHANPGDOODFCAZ-NSCUHMNNSA-N | COC(=O)/C(=C\Cl)/Cl | 1891 | 8447 | 1 | 1 |
| 2,3,3-trichloropropenoic acid, methyl ester (C4H3Cl3O2) | LUAABZPMDXDUBN-UHFFFAOYSA-N | COC(=O)C(=C(Cl)Cl)Cl | 2001 | 6115 | 1 | 1 |
| Hexachloropropene (C3Cl6) | VFDYKPARTDCDCU-UHFFFAOYSA-N | C(=C(Cl)Cl)(C(Cl)(Cl)Cl)Cl | 533 | 7399 | 2 | 1 |
| 2-Propen-1-ol, dichloroacetate (C5H6Cl2O2) | UJUPTENXDPFFDP-UHFFFAOYSA-N | C=CCOC(=O)C(Cl)Cl | 0 | 70 | 2 | 2 |
| 2-Propenoic acid, 2-methyl-, 2-chloroethyl ester (C6H9ClO2) | GPOGMJLHWQHEGF-UHFFFAOYSA-N | CC(=C)C(=O)OCCCl | 80 | 1852 | 2 | 1 |
| Propanoic acid, 2-chloro, 2-propenyl ester (C6H9ClO2) | FDCTVCOKDZZPRP-UHFFFAOYSA-N | CC(C(=O)OCC=C)Cl | 241 | 207 | 2 | 1 |
| Ethchlorvynol (C7H9ClO) | ZEHYJZXQEQOSON-AATRIKPKSA-N | CCC(/C=C/Cl)(C#C)O | 0 | 1832 | 2 | 1 |
| Desethyl-desisopropyl-atrazine (C3H4ClN5) | FVFVNNKYKYZTJU-UHFFFAOYSA-N | C1(=NC(=NC(=N1)Cl)N)N | 9999 | 3069 | 1 | 1 |
| 4-Chloro-2,6-diaminopyrimidine (C4H5ClN4) | QJIUMVUZDYPQRT-UHFFFAOYSA-N | C1=C(N=C(N=C1Cl)N)N | 9999 | 6629 | 1 | 1 |
| 5-Chloro-2-methoxypyrimidine (C5H5ClN2O) | GJNGJHZIOYFEOP-UHFFFAOYSA-N | COC1=NC=C(C=N1)Cl | 8190 | 120 | 1 | 1 |
| 5-Amino-4,6-dichloropyrimidine (C4H3Cl2N3) | NIGDWBHWHVHOAD-UHFFFAOYSA-N | C1=NC(=C(C(=N1)Cl)N)Cl | 9999 | 4724 | 1 | 1 |
| Pyrimidine, 2,4,6-trichloro- (C4HCl3N2) | DPVIABCMTHHTGB-UHFFFAOYSA-N | C1=C(N=C(N=C1Cl)Cl)Cl | 9999 | 6869 | 1 | 1 |
| Pyridazine, 3-chloro-6-methoxy- (C5H5ClN2O) | XBJLKXOOHLLTPG-UHFFFAOYSA-N | COC1=NN=C(C=C1)Cl | 6058 | 1756 | 1 | 1 |
| 2-Chloro-6-methoxypyridine (C6H6ClNO) | VAVGOGHLNAJECD-UHFFFAOYSA-N | COC1=NC(=CC=C1)Cl | 8369 | 1119 | 1 | 1 |
| 3,4,5-Trichloropyridine (C5H2Cl3N) | KKWRVUBDCJQHBZ-UHFFFAOYSA-N | C1=C(C(=C(C=N1)Cl)Cl)Cl | 9999 | 4994 | 1 | 1 |
| p-Chlorophenylhydroxylamine (C6H6ClNO) | VVQDMDRAUXFEND-UHFFFAOYSA-N | C1=CC(=CC=C1NO)Cl | 5363 | 258 | 1 | 1 |
| Benzene, 1-chloro-4-nitroso- (C6H4ClNO) | IEYSGPZUULPJPQ-UHFFFAOYSA-N | C1=CC(=CC=C1N=O)Cl | 5900 | 0 | 1 | 2 |
| 4-Chloro-meta-benzenedithiol (C6H5ClS2) | HCDLDZCJYMCKQH-UHFFFAOYSA-N | C1=CC(=C(C=C1S)S)Cl | 9999 | 2019 | 1 | 1 |
| 5-Chloro-1,3-phenylenediamine (C6H7ClN2) | VZNUCJOYPXKLTA-UHFFFAOYSA-N | C1=C(C=C(C=C1N)Cl)N | 9999 | 1199 | 1 | 1 |
| 1,3-Benzenediamine, 4-chloro- (C6H7ClN2) | ZWUBBMDHSZDNTA-UHFFFAOYSA-N | C1=CC(=C(C=C1N)N)Cl | 9999 | 1000 | 1 | 1 |
| 1,2-Benzenediamine, 4-chloro- (C6H7ClN2) | BXIXXXYDDJVHDL-UHFFFAOYSA-N | C1=CC(=C(C=C1Cl)N)N | 9999 | 1640 | 1 | 1 |
| 2-amino-4-chlorophenol (C6H6ClNO) | SWFNPENEBHAHEB-UHFFFAOYSA-N | C1=CC(=C(C=C1Cl)N)O | 9999 | 170 | 1 | 1 |
| 1,4-Benzenediol, 2-chloro- (C6H5ClO2) | AJPXTSMULZANCB-UHFFFAOYSA-N | C1=CC(=C(C=C1O)Cl)O | 9999 | 119 | 1 | 1 |
| 1,3-Benzenediol, 4-chloro- (C6H5ClO2) | JQVAPEJNIZULEK-UHFFFAOYSA-N | C1=CC(=C(C=C1O)O)Cl | 9999 | 219 | 1 | 1 |
| 3-Chlorothioanisole (C7H7ClS) | PTGSDZVASWKUHK-UHFFFAOYSA-N | CSC1=CC(=CC=C1)Cl | 9999 | 897 | 1 | 1 |
| Benzenamine, 3-chloro-N-methyl- (C7H8ClN) | WFGYSQDPURFIFL-UHFFFAOYSA-N | CNC1=CC(=CC=C1)Cl | 6726 | 360 | 1 | 1 |
| Benzenamine, 2-chloro-N-methyl- (C7H8ClN) | WGNNILPYHCKCFF-UHFFFAOYSA-N | CNC1=CC=CC=C1Cl | 6906 | 170 | 1 | 1 |
| Benzenamine, 4-chloro-N-methyl- (C7H8ClN) | XCEYKKJMLOFDSS-UHFFFAOYSA-N | CNC1=CC=C(C=C1)Cl | 8879 | 279 | 1 | 1 |
| 3-Chloroanisole (C7H7ClO) | YUKILTJWFRTXGB-UHFFFAOYSA-N | COC1=CC(=CC=C1)Cl | 9999 | 909 | 1 | 1 |
| 4-Chloroanisole (C7H7ClO) | YRGAYAGBVIXNAQ-UHFFFAOYSA-N | COC1=CC=C(C=C1)Cl | 9999 | 580 | 1 | 1 |
| 2-Chloroanisole (C7H7ClO) | QGRPVMLBTFGQDQ-UHFFFAOYSA-N | COC1=CC=CC=C1Cl | 9999 | 407 | 1 | 1 |
| 2,3-Dichlorobenzenethiol (C6H4Cl2S) | QGRKONUHHGBHRB-UHFFFAOYSA-N | C1=CC(=C(C(=C1)Cl)Cl)S | 9759 | 9999 | 1 | 1 |
| Benzenethiol, 2,5-dichloro- (C6H4Cl2S) | QIULLHZMZMGGFH-UHFFFAOYSA-N | C1=CC(=C(C=C1Cl)S)Cl | 9999 | 5779 | 1 | 1 |
| 2,4-Dichloroaniline (C6H5Cl2N) | KQCMTOWTPBNWDB-UHFFFAOYSA-N | C1=CC(=C(C=C1Cl)Cl)N | 9999 | 1747 | 1 | 1 |
| 2,3-Dichloroaniline (C6H5Cl2N) | BRPSAOUFIJSKOT-UHFFFAOYSA-N | C1=CC(=C(C(=C1)Cl)Cl)N | 9999 | 1149 | 1 | 1 |
| 2,6-Dichloroaniline (C6H5Cl2N) | JDMFXJULNGEPOI-UHFFFAOYSA-N | C1=CC(=C(C(=C1)Cl)N)Cl | 9999 | 1259 | 1 | 1 |
| 3,4-Dichloroaniline (C6H5Cl2N) | SDYWXFYBZPNOFX-UHFFFAOYSA-N | C1=CC(=C(C=C1N)Cl)Cl | 9999 | 1430 | 1 | 1 |
| 2,5-Dichloroaniline (C6H5Cl2N) | AVYGCQXNNJPXSS-UHFFFAOYSA-N | C1=CC(=C(C=C1Cl)N)Cl | 9999 | 1269 | 1 | 1 |
| 2,4-Dichlorophenol (C6H4Cl2O) | HFZWRUODUSTPEG-UHFFFAOYSA-N | C1=CC(=C(C=C1Cl)Cl)O | 9999 | 169 | 1 | 1 |
| Phenol, 2,3-dichloro- (C6H4Cl2O) | UMPSXRYVXUPCOS-UHFFFAOYSA-N | C1=CC(=C(C(=C1)Cl)Cl)O | 9999 | 510 | 1 | 1 |
| Phenol, 2,5-dichloro- (C6H4Cl2O) | RANCECPPZPIPNO-UHFFFAOYSA-N | C1=CC(=C(C=C1Cl)O)Cl | 9999 | 210 | 1 | 1 |
| Phenol, 3,5-dichloro- (C6H4Cl2O) | VPOMSPZBQMDLTM-UHFFFAOYSA-N | C1=C(C=C(C=C1Cl)Cl)O | 9999 | 409 | 1 | 1 |
| 2,6-Dichlorophenol (C6H4Cl2O) | HOLHYSJJBXSLMV-UHFFFAOYSA-N | C1=CC(=C(C(=C1)Cl)O)Cl | 9999 | 217 | 1 | 1 |
| Phenol, 3,4-dichloro- (C6H4Cl2O) | WDNBURPWRNALGP-UHFFFAOYSA-N | C1=CC(=C(C=C1O)Cl)Cl | 9999 | 360 | 1 | 1 |
| 1,3,5-Trichlorobenzene (C6H3Cl3) | XKEFYDZQGKAQCN-UHFFFAOYSA-N | C1=C(C=C(C=C1Cl)Cl)Cl | 9999 | 3210 | 1 | 1 |
| 1,2,4-Trichlorobenzene (C6H3Cl3) | PBKONEOXTCPAFI-UHFFFAOYSA-N | C1=CC(=C(C=C1Cl)Cl)Cl | 9999 | 4490 | 1 | 1 |
| 1,2,3-Trichlorobenzene (C6H3Cl3) | RELMFMZEBKVZJC-UHFFFAOYSA-N | C1=CC(=C(C(=C1)Cl)Cl)Cl | 9999 | 3800 | 1 | 1 |
| 2-Pyrimidinamine, 4-chloro-6-methyl- (C5H6ClN3) | NPTGVVKPLWFPPX-UHFFFAOYSA-N | CC1=CC(=NC(=N1)N)Cl | 9999 | 7119 | 1 | 1 |
| Pyridazine, 3,6-dichloro-4-methyl- (C5H4Cl2N2) | ROYHWGZNGMXQEU-UHFFFAOYSA-N | CC1=CC(=NN=C1Cl)Cl | 2600 | 0 | 1 | 2 |
| 4-Chlorobenzyl mercaptan (C7H7ClS) | GKQXPTHQTXCXEV-UHFFFAOYSA-N | C1=CC(=CC=C1CS)Cl | 2009 | 80 | 1 | 2 |
| Benzonitrile, 4-chloro- (C7H4ClN) | GJNGXPDXRVXSEH-UHFFFAOYSA-N | C1=CC(=CC=C1C#N)Cl | 9999 | 3300 | 1 | 1 |
| Benzonitrile, 3-chloro- (C7H4ClN) | WBUOVKBZJOIOAE-UHFFFAOYSA-N | C1=CC(=CC(=C1)Cl)C#N | 9999 | 3021 | 1 | 1 |
| 2-Chlorobenzonitrile (C7H4ClN) | NHWQMJMIYICNBP-UHFFFAOYSA-N | C1=CC=C(C(=C1)C#N)Cl | 9999 | 3620 | 1 | 1 |
| 4-chlorobenzylamine (C7H8ClN) | YMVFJGSXZNNUDW-UHFFFAOYSA-N | C1=CC(=CC=C1CN)Cl | 1079 | 9999 | 1 | 1 |
| Benzenemethanamine, 3-chloro- (C7H8ClN) | BJFPYGGTDAYECS-UHFFFAOYSA-N | C1=CC(=CC(=C1)Cl)CN | 869 | 9999 | 1 | 1 |
| Benzenemethanamine, 2-chloro- (C7H8ClN) | KDDNKZCVYQDGKE-UHFFFAOYSA-N | C1=CC=C(C(=C1)CN)Cl | 769 | 9999 | 2 | 1 |
| Benzenemethanol, 2-chloro- (C7H7ClO) | MBYQPPXEXWRMQC-UHFFFAOYSA-N | C1=CC=C(C(=C1)CO)Cl | 4329 | 7179 | 1 | 1 |
| 3-Chlorobenzyl alcohol (C7H7ClO) | ZSRDNPVYGSFUMD-UHFFFAOYSA-N | C1=CC(=CC(=C1)Cl)CO | 4694 | 5525 | 1 | 1 |
| 4-Chlorobenzyl alcohol (C7H7ClO) | PTHGDVCPCZKZKR-UHFFFAOYSA-N | C1=CC(=CC=C1CO)Cl | 5915 | 7577 | 1 | 1 |
| Benzoyl chloride (C7H5ClO) | PASDCCFISLVPSO-UHFFFAOYSA-N | C1=CC=C(C=C1)C(=O)Cl | 951 | 9999 | 1 | 1 |
| Benzaldehyde, 4-chloro- (C7H5ClO) | AVPYQKSLYISFPO-UHFFFAOYSA-N | C1=CC(=CC=C1C=O)Cl | 7239 | 20 | 1 | 2 |
| m-Chlorobenzaldehyde (C7H5ClO) | SRWILAKSARHZPR-UHFFFAOYSA-N | C1=CC(=CC(=C1)Cl)C=O | 8219 | 30 | 1 | 2 |
| 2-Chlorobenzaldehyde (C7H5ClO) | FPYUJUBAXZAQNL-UHFFFAOYSA-N | C1=CC=C(C(=C1)C=O)Cl | 7569 | 50 | 1 | 2 |
| 4-Chloro-2-methylthiophenol (C7H7ClS) | YIUPEFSJLWXHJR-UHFFFAOYSA-N | CC1=C(C=CC(=C1)Cl)S | 9999 | 6190 | 1 | 1 |
| 2-Chloro-4-methylaniline (C7H8ClN) | XGYLSRFSXKAYCR-UHFFFAOYSA-N | CC1=CC(=C(C=C1)N)Cl | 7349 | 9999 | 1 | 1 |
| m-Toluidine, 4-chloro- (C7H8ClN) | HIHCTGNZNHSZPP-UHFFFAOYSA-N | CC1=C(C=CC(=C1)N)Cl | 9021 | 9999 | 1 | 1 |
| o-Toluidine, 3-chloro- (C7H8ClN) | ZUVPLKVDZNDZCM-UHFFFAOYSA-N | CC1=C(C=CC=C1Cl)N | 9999 | 7249 | 1 | 1 |
| o-Toluidine, 6-chloro- (C7H8ClN) | WFNLHDJJZSJARK-UHFFFAOYSA-N | CC1=C(C(=CC=C1)Cl)N | 6050 | 9999 | 1 | 1 |
| o-Toluidine, 4-chloro- (C7H8ClN) | CXNVOWPRHWWCQR-UHFFFAOYSA-N | CC1=C(C=CC(=C1)Cl)N | 9999 | 8409 | 1 | 1 |
| p-Toluidine, 3-chloro- (C7H8ClN) | RQKFYFNZSHWXAW-UHFFFAOYSA-N | CC1=C(C=C(C=C1)N)Cl | 9859 | 9029 | 1 | 1 |
| o-Toluidine, 5-chloro- (C7H8ClN) | WRZOMWDJOLIVQP-UHFFFAOYSA-N | CC1=C(C=C(C=C1)Cl)N | 9999 | 7489 | 1 | 1 |
| m-Toluidine, 6-chloro (C7H8ClN) | HPSCXFOQUFPEPE-UHFFFAOYSA-N | CC1=CC(=C(C=C1)Cl)N | 9585 | 9999 | 1 | 1 |
| Phenol, 4-chloro-2-methyl- (C7H7ClO) | RHPUJHQBPORFGV-UHFFFAOYSA-N | CC1=C(C=CC(=C1)Cl)O | 9999 | 9579 | 1 | 1 |
| Phenol, 5-chloro-2-methyl- (C7H7ClO) | KKFPXGXMSBBNJI-UHFFFAOYSA-N | CC1=C(C=C(C=C1)Cl)O | 4812 | 9999 | 1 | 1 |
| Phenol, 3-chloro-4-methyl- (C7H7ClO) | VQZRLBWPEHFGCD-UHFFFAOYSA-N | CC1=C(C=C(C=C1)O)Cl | 4165 | 9999 | 1 | 1 |
| Phenol, 2-chloro-5-methyl- (C7H7ClO) | SMFHPCZZAAMJJO-UHFFFAOYSA-N | CC1=CC(=C(C=C1)Cl)O | 6370 | 9999 | 1 | 1 |
| Phenol, 2-chloro-4-methyl- (C7H7ClO) | AQJFATAFTQCRGC-UHFFFAOYSA-N | CC1=CC(=C(C=C1)O)Cl | 3694 | 9999 | 1 | 1 |
| Phenol, 2-chloro-6-methyl- (C7H7ClO) | YPNZJHFXFVLXSE-UHFFFAOYSA-N | CC1=C(C(=CC=C1)Cl)O | 5318 | 9999 | 1 | 1 |
| Benzene, 1-chloro-4-(chloromethyl)- (C7H6Cl2) | JQZAEUFPPSRDOP-UHFFFAOYSA-N | C1=CC(=CC=C1CCl)Cl | 1929 | 9999 | 1 | 1 |
| Benzene, 1-chloro-2-(chloromethyl)- (C7H6Cl2) | BASMANVIUSSIIM-UHFFFAOYSA-N | C1=CC=C(C(=C1)CCl)Cl | 1889 | 9999 | 1 | 1 |
| Benzene, 1-chloro-3-(chloromethyl)- (C7H6Cl2) | DDGRAFHHXYIQQR-UHFFFAOYSA-N | C1=CC(=CC(=C1)Cl)CCl | 2189 | 9999 | 1 | 1 |
| Benzal chloride (C7H6Cl2) | CAHQGWAXKLQREW-UHFFFAOYSA-N | C1=CC=C(C=C1)C(Cl)Cl | 990 | 9999 | 1 | 1 |
| 2,6-Dichlorotoluene (C7H6Cl2) | DMEDNTFWIHCBRK-UHFFFAOYSA-N | CC1=C(C=CC=C1Cl)Cl | 4719 | 9999 | 1 | 1 |
| 2,5-Dichlorotoluene (C7H6Cl2) | KFAKZJUYBOYVKA-UHFFFAOYSA-N | CC1=C(C=CC(=C1)Cl)Cl | 6689 | 9999 | 1 | 1 |
| Benzene, 1,3-dichloro-5-methyl- (C7H6Cl2) | RYMMNSVHOKXTNN-UHFFFAOYSA-N | CC1=CC(=CC(=C1)Cl)Cl | 5210 | 9999 | 1 | 1 |
| Benzene, 1,2-dichloro-3-methyl- (C7H6Cl2) | GWLKCPXYBLCEKC-UHFFFAOYSA-N | CC1=C(C(=CC=C1)Cl)Cl | 4169 | 9999 | 1 | 1 |
| 2,4-Dichlorotoluene (C7H6Cl2) | FUNUTBJJKQIVSY-UHFFFAOYSA-N | CC1=C(C=C(C=C1)Cl)Cl | 4249 | 9999 | 1 | 1 |
| 3,4-Dichlorotoluene (C7H6Cl2) | WYUIWKFIFOJVKW-UHFFFAOYSA-N | CC1=CC(=C(C=C1)Cl)Cl | 4829 | 9999 | 1 | 1 |
| Benzene,(chloroethynyl)- (C8H5Cl) | GDWZLADUGAKASM-UHFFFAOYSA-N | C1=CC=C(C=C1)C#CCl | 9999 | 2632 | 1 | 1 |
| Benzene, 1-chloro-4-ethynyl- (C8H5Cl) | LFZJRTMTKGYJRS-UHFFFAOYSA-N | C#CC1=CC=C(C=C1)Cl | 9999 | 1982 | 1 | 1 |
| 2-Chloro-4,6-dimethylpyrimidine (C6H7ClN2) | RZVPFDOTMFYQHR-UHFFFAOYSA-N | CC1=CC(=NC(=N1)Cl)C | 9999 | 2340 | 1 | 1 |
| 2-Chloro-3,6-dimethylpyrazine (C6H7ClN2) | NNBALVIZMGWZHS-UHFFFAOYSA-N | CC1=CN=C(C(=N1)Cl)C | 9999 | 9568 | 1 | 1 |
| Benzene, (2-chloroethyl)- (C8H9Cl) | MNNZINNZIQVULG-UHFFFAOYSA-N | C1=CC=C(C=C1)CCCl | 1881 | 290 | 1 | 1 |
| Benzene, 1-(chloromethyl)-4-methyl- (C8H9Cl) | DMHZDOTYAVHSEH-UHFFFAOYSA-N | CC1=CC=C(C=C1)CCl | 2542 | 9999 | 1 | 1 |
| Benzene, 1-(chloromethyl)-2-methyl- (C8H9Cl) | VQRBXYBBGHOGFT-UHFFFAOYSA-N | CC1=CC=CC=C1CCl | 2236 | 9999 | 1 | 1 |
| Benzene, 1-(chloromethyl)-3-methyl- (C8H9Cl) | LZBOHNCMCCSTJX-UHFFFAOYSA-N | CC1=CC(=CC=C1)CCl | 2207 | 9999 | 1 | 1 |
| Benzene, (1-chloroethyl)- (C8H9Cl) | GTLWADFFABIGAE-UHFFFAOYSA-N | CC(C1=CC=CC=C1)Cl | 1179 | 9999 | 1 | 1 |
| Benzene, 1-chloro-3-ethyl- (C8H9Cl) | LOXUEGMPESDGBQ-UHFFFAOYSA-N | CCC1=CC(=CC=C1)Cl | 5215 | 8818 | 1 | 1 |
| Benzene, 1-chloro-4-ethyl- (C8H9Cl) | GPOFSFLJOIAMSA-UHFFFAOYSA-N | CCC1=CC=C(C=C1)Cl | 3633 | 4694 | 1 | 1 |
| Benzene, 1-chloro-2-ethyl- (C8H9Cl) | CVGAWKYSRYXQOI-UHFFFAOYSA-N | CCC1=CC=CC=C1Cl | 4104 | 5645 | 1 | 1 |
| o-Xylene, 4-chloro- (C8H9Cl) | HNQLMBJUMVLFCF-UHFFFAOYSA-N | CC1=C(C=C(C=C1)Cl)C | 4634 | 9999 | 1 | 1 |
| m-Xylene, 2-chloro- (C8H9Cl) | VDXLAYAQGYCQEO-UHFFFAOYSA-N | CC1=C(C(=CC=C1)C)Cl | 4419 | 9999 | 1 | 1 |
| m-Xylene, 4-chloro- (C8H9Cl) | UIEVCEQLNUHDIF-UHFFFAOYSA-N | CC1=CC(=C(C=C1)Cl)C | 5035 | 9999 | 1 | 1 |
| Benzene, 2-chloro-1,4-dimethyl- (C8H9Cl) | KZNRNQGTVRTDPN-UHFFFAOYSA-N | CC1=CC(=C(C=C1)C)Cl | 5216 | 9999 | 1 | 1 |
| Thiophene, 3,4-dichlorotetrahydro-, 1,1-dioxide (C4H6Cl2O2S) | JCUQWSIALJCIEE-UHFFFAOYSA-N | C1C(C(CS1(=O)=O)Cl)Cl | 140 | 30 | 2 | 2 |
| 1,3-Dioxolane, 4-(chloromethyl)-2,2-dimethyl- (C6H11ClO2) | BNPOTXLWPZOESZ-UHFFFAOYSA-N | CC1(OCC(O1)CCl)C | 0 | 0 | 2 | 2 |
| Cyclohexanecarbonyl chloride (C7H11ClO) | RVOJTCZRIKWHDX-UHFFFAOYSA-N | C1CCC(CC1)C(=O)Cl | 80 | 1039 | 2 | 1 |
| Cyclohexanone,2-chloro-2-methyl- (C7H11ClO) | IDRWHLQDVSLCBJ-UHFFFAOYSA-N | CC1(CCCCC1=O)Cl | 1700 | 400 | 1 | 1 |
| 6-Chlorouracil (C4H3ClN2O2) | PKUFNWPSFCOSLU-UHFFFAOYSA-N | C1=C(NC(=O)NC1=O)Cl | 3170 | 144 | 1 | 1 |
| 4,5-Dichloro-2-methyl-4-isothiazolin-3-one (C4H3Cl2NOS) | CVZDIUZSWUDGOP-UHFFFAOYSA-N | CN1C(=O)C(=C(S1)Cl)Cl | 5382 | 127 | 1 | 1 |
| 4-chlorostyrene (C8H7Cl) | KTZVZZJJVJQZHV-UHFFFAOYSA-N | C=CC1=CC=C(C=C1)Cl | 9999 | 4964 | 1 | 1 |
| Benzene, 1-chloro-3-ethenyl- (C8H7Cl) | BOVQCIDBZXNFEJ-UHFFFAOYSA-N | C=CC1=CC(=CC=C1)Cl | 9999 | 7529 | 1 | 1 |
| 2-Chlorostyrene (C8H7Cl) | ISRGONDNXBCDBM-UHFFFAOYSA-N | C=CC1=CC=CC=C1Cl | 8177 | 9999 | 1 | 1 |
| Benzene, (1-chloroethenyl)- (C8H7Cl) | XHAFIUUYXQFJEW-UHFFFAOYSA-N | C=C(C1=CC=CC=C1)Cl | 3569 | 9999 | 1 | 1 |
| β-Chlorostyrene (C8H7Cl) | SBYMUDUGTIKLCR-VOTSOKGWSA-N | C1=CC=C(C=C1)/C=C/Cl | 5866 | 9999 | 1 | 1 |
| 2,5-Furandione, 3,4-dichloro- (C4Cl2O3) | AGULWIQIYWWFBJ-UHFFFAOYSA-N | C1(=C(C(=O)OC1=O)Cl)Cl | 6529 | 30 | 1 | 2 |
| Isoxazole, 4-(chloromethyl)-3,5-dimethyl- (C6H8ClNO) | NIFAUKBQIAURIM-UHFFFAOYSA-N | CC1=C(C(=NO1)C)CCl | 1589 | 9999 | 1 | 1 |
| 3-Cyclohexenecarbonyl chloride (C7H9ClO) | CXWMHIRIXVNVQN-UHFFFAOYSA-N | C1CC(CC=C1)C(=O)Cl | 100 | 1442 | 2 | 1 |
| Tetrachlorothiophene (C4Cl4S) | WZXXZHONLFRKGG-UHFFFAOYSA-N | C1(=C(SC(=C1Cl)Cl)Cl)Cl | 7440 | 4060 | 1 | 1 |
| 5-Chloro-2-thiophenecarboxylic acid (C5H3ClO2S) | QZLSBOVWPHXCLT-UHFFFAOYSA-N | C1=C(SC(=C1)Cl)C(=O)O | 6360 | 46 | 1 | 2 |
| 2(1H)-Pyridinone, 6-chloro-1-methyl- (C6H6ClNO) | WTMIJNWKTCXRBK-UHFFFAOYSA-N | CN1C(=O)C=CC=C1Cl | 4958 | 9999 | 1 | 1 |
| 3,5-Dichloro-2-pyridone (C5H3Cl2NO) | ZICOPWJJZSJEDL-UHFFFAOYSA-N | C1=C(C(=O)NC=C1Cl)Cl | 8180 | 875 | 1 | 1 |
| 2-Acetyl-5-chlorothiophene (C6H5ClOS) | HTZGPEHWQCRXGZ-UHFFFAOYSA-N | CC(=O)C1=CC=C(S1)Cl | 4113 | 0 | 1 | 2 |
| 2,5-Cyclohexadiene-1,4-dione, 2-chloro- (C6H3ClO2) | WOGWYSWDBYCVDY-UHFFFAOYSA-N | C1=CC(=O)C(=CC1=O)Cl | 9999 | 300 | 1 | 1 |
| 4-Chloro-1-azabicyclo[2.2.2]octane (C7H12ClN) | CHAXYLNTCZVHBJ-UHFFFAOYSA-N | C1CN2CCC1(CC2)Cl | 80 | 9999 | 2 | 1 |
| 3-Chloro-1-azabicyclo[2.2.2]octane (C7H12ClN) | NOLVABYTFMDREN-UHFFFAOYSA-N | C1CN2CCC1C(C2)Cl | 3039 | 8099 | 1 | 1 |
| 3-Chloro-2-norbornanone (C7H9ClO) | PQRKEKMZLKKQOP-UHFFFAOYSA-N | C1CC2CC1C(C2=O)Cl | 2606 | 746 | 1 | 1 |
| Bicyclo[2.2.2]octane, 2-chloro- (C8H13Cl) | PTVXDQZENDCNDA-UHFFFAOYSA-N | C1CC2CCC1CC2Cl | 387 | 2793 | 2 | 1 |
| Bicyclo[3.2.1]oct-2-ene, 3-chloro- (C8H11Cl) | XLVAWKKCOIWHBT-UHFFFAOYSA-N | C1CC2CC1CC(=C2)Cl | 2802 | 2065 | 1 | 1 |
| (1-Hydroxy-2,2,2-trichloroethyl)formamide (C3H4Cl3NO2) | ACZVWYLTJHGUCP-UHFFFAOYSA-N | C(=O)NC(C(Cl)(Cl)Cl)O | 0 | 770 | 2 | 1 |
| 2-Chloroethyl carbonate (C5H8Cl2O3) | WQULVXNWEYLDJY-UHFFFAOYSA-N | C(CCl)OC(=O)OCCCl | 0 | 199 | 2 | 1 |
| Propanoic acid, 2,2,3,3-tetrachloro, methyl ester (C4H4Cl4O2) | UQYYMXYJSOOLLA-UHFFFAOYSA-N | COC(=O)C(C(Cl)Cl)(Cl)Cl | 0 | 230 | 2 | 1 |
| Propanoic acid, 2,3,3,3-tetrachloro, methyl ester (C4H4Cl4O2) | LFHGTEYJIKRWFF-UHFFFAOYSA-N | COC(=O)C(C(Cl)(Cl)Cl)Cl | 30 | 100 | 2 | 2 |
| 2-Propanone, 1,1,1,3,3,3-hexachloro- (C3Cl6O) | DOJXGHGHTWFZHK-UHFFFAOYSA-N | C(=O)(C(Cl)(Cl)Cl)C(Cl)(Cl)Cl | 10 | 109 | 2 | 1 |
| 1,1,1,2,3,3,3-Heptachloropropane (C3HCl7) | LZOUTMTXULWVSU-UHFFFAOYSA-N | C(C(Cl)(Cl)Cl)(C(Cl)(Cl)Cl)Cl | 0 | 539 | 2 | 1 |
| 1,1,1,2,2,3,3-Heptachloropropane (C3HCl7) | YFIIENAGGCUHIQ-UHFFFAOYSA-N | C(C(C(Cl)(Cl)Cl)(Cl)Cl)(Cl)Cl | 0 | 189 | 2 | 1 |
| Ethanol, 2-[2-(2-chloroethoxy)ethoxy]- (C6H13ClO3) | KECMLGZOQMJIBM-UHFFFAOYSA-N | C(COCCOCCCl)O | 0 | 0 | 2 | 2 |
| Sesquimustard (C6H12Cl2S2) | AMGNHZVUZWILSB-UHFFFAOYSA-N | C(CSCCCl)SCCCl | 1231 | 160 | 1 | 1 |
| 1,2-bis-(2-Chloroethyl)ethane (C6H12Cl2O2) | AGYUOJIYYGGHKV-UHFFFAOYSA-N | C(COCCCl)OCCCl | 0 | 0 | 2 | 2 |
| Ethane, 1,1-dichloro-2,2-diethoxy- (C6H12Cl2O2) | CDHLQZJRWKQATP-UHFFFAOYSA-N | CCOC(C(Cl)Cl)OCC | 0 | 0 | 2 | 2 |
| HN3 (C6H12Cl3N) | FDAYLTPAFBGXAB-UHFFFAOYSA-N | C(CCl)N(CCCl)CCCl | 120 | 350 | 2 | 1 |
| Propyl trichloroacetate (C5H7Cl3O2) | QWWBZHDIGCDTLY-UHFFFAOYSA-N | CCCOC(=O)C(Cl)(Cl)Cl | 0 | 140 | 2 | 1 |
| Isopropyl trichloroacetate (C5H7Cl3O2) | JYXIYFNAIFVCAN-UHFFFAOYSA-N | CC(C)OC(=O)C(Cl)(Cl)Cl | 0 | 220 | 2 | 1 |
| Butanoic acid, 4-chloro-4-oxo-, ethyl ester (C6H9ClO3) | IXZFDJXHLQQSGQ-UHFFFAOYSA-N | CCOC(=O)CCC(=O)Cl | 0 | 1640 | 2 | 1 |
| Butanoic acid, 4-chloro-3-oxo-, ethyl ester (C6H9ClO3) | OHLRLMWUFVDREV-UHFFFAOYSA-N | CCOC(=O)CC(=O)CCl | 520 | 20 | 2 | 2 |
| 3-Chloropropionaldehyde diethyl acetal (C7H15ClO2) | NXHONHDWVLPPCS-UHFFFAOYSA-N | CCOC(CCCl)OCC | 0 | 0 | 2 | 2 |
| Butanoic acid, 2-chloro-3-oxo-, ethyl ester (C6H9ClO3) | RDULEYWUGKOCMR-UHFFFAOYSA-N | CCOC(=O)C(C(=O)C)Cl | 179 | 10 | 2 | 2 |
| 2-Acetoxyisobutyryl chloride (C6H9ClO3) | RBTCRFLJLUNCLL-UHFFFAOYSA-N | CC(=O)OC(C)(C)C(=O)Cl | 0 | 10 | 2 | 2 |
| 5-Chloropentanoic acid, chloromethyl ester (C6H10Cl2O2) | VQDRONCNGCJOGI-UHFFFAOYSA-N | C(CCCl)CC(=O)OCCl | 0 | 390 | 2 | 1 |
| 4-Chloropentanoic acid, chloromethyl ester (C6H10Cl2O2) | BTDLLBKQZJSWTP-UHFFFAOYSA-N | CC(CCC(=O)OCCl)Cl | 0 | 0 | 2 | 2 |
| 2-Chloropentanoic acid, chloromethyl ester (C6H10Cl2O2) | YVZRATJRHVPWJR-UHFFFAOYSA-N | CCCC(C(=O)OCCl)Cl | 0 | 0 | 2 | 2 |
| Butyl dichloroacetate (C6H10Cl2O2) | MASXONVJEAXEIV-UHFFFAOYSA-N | CCCCOC(=O)C(Cl)Cl | 0 | 0 | 2 | 2 |
| Isopropyl 2,3-dichloropropanoate (C6H10Cl2O2) | DQNKZNXCBULFCV-UHFFFAOYSA-N | CC(C)OC(=O)C(CCl)Cl | 20 | 40 | 2 | 2 |
| Acetic acid, dichloro, isobutyl ester (C6H10Cl2O2) | XLKJPQKUATYFQJ-UHFFFAOYSA-N | CC(C)COC(=O)C(Cl)Cl | 0 | 0 | 2 | 2 |
| Acetic acid, dichloro, 1-methylpropyl ester (C6H10Cl2O2) | IQYVZHJFEAIITM-UHFFFAOYSA-N | CCC(C)OC(=O)C(Cl)Cl | 0 | 20 | 2 | 2 |
| Propanoic acid, 2,2-dichloro-, 1-methylethyl ester (C6H10Cl2O2) | QVZCJUSIWAGBDX-UHFFFAOYSA-N | CC(C)OC(=O)C(C)(Cl)Cl | 10 | 40 | 2 | 2 |
| 1-Pentanol, 5-chloro-, acetate (C7H13ClO2) | ZCYVIAZIVJNAMO-UHFFFAOYSA-N | CC(=O)OCCCCCCl | 0 | 0 | 2 | 2 |
| Pentanoic acid, 5-chloro-, ethyl ester (C7H13ClO2) | JHPBGAORCOXWNO-UHFFFAOYSA-N | CCOC(=O)CCCCCl | 90 | 4154 | 2 | 1 |
| hexyl chloroformate (C7H13ClO2) | KIWBRXCOTCXSSZ-UHFFFAOYSA-N | CCCCCCOC(=O)Cl | 0 | 10 | 2 | 2 |
| Propanoic acid, 3-chloro-, butyl ester (C7H13ClO2) | KRZKQEQBZXWCDJ-UHFFFAOYSA-N | CCCCOC(=O)CCCl | 0 | 0 | 2 | 2 |
| Pentyl chloroacetate (C7H13ClO2) | SAOZOMQLKLWJAN-UHFFFAOYSA-N | CCCCCOC(=O)CCl | 0 | 0 | 2 | 2 |
| Hexanoic acid, chloromethyl ester (C7H13ClO2) | HNUBMUSOXKDQIN-UHFFFAOYSA-N | CCCCCC(=O)OCCl | 0 | 0 | 2 | 2 |
| Acetic acid, chloro-, 3-methylbutyl ester (C7H13ClO2) | UZQBACINTKFBSX-UHFFFAOYSA-N | CC(C)CCOC(=O)CCl | 0 | 0 | 2 | 2 |
| Propanoic acid, 3-chloro-, 2-methylpropyl ester (C7H13ClO2) | NLGHCSSXCMOGKD-UHFFFAOYSA-N | CC(C)COC(=O)CCCl | 0 | 0 | 2 | 2 |
| Propanoic acid, 3-chloro, 1-methylpropyl ester (C7H13ClO2) | XLIOZHNKYDPTAJ-UHFFFAOYSA-N | CCC(C)OC(=O)CCCl | 0 | 0 | 2 | 2 |
| Acetic acid, chloro-, 1-methylbutyl ester (C7H13ClO2) | IYMXUCSCPWQCFH-UHFFFAOYSA-N | CCCC(C)OC(=O)CCl | 0 | 20 | 2 | 2 |
| Propanoic acid, 2-chloro-, butyl ester (C7H13ClO2) | KATNUXHENWPMQJ-UHFFFAOYSA-N | CCCCOC(=O)C(C)Cl | 93 | 21 | 2 | 2 |
| Propanoic acid, 2-chloro, 1,1-dimethylethyl ester (C7H13ClO2) | YXYWJZNXZGYLNO-UHFFFAOYSA-N | CC(C(=O)OC(C)(C)C)Cl | 63 | 235 | 2 | 1 |
| Hexanedioyl dichloride (C6H8Cl2O2) | PWAXUOGZOSVGBO-UHFFFAOYSA-N | C(CCC(=O)Cl)CC(=O)Cl | 0 | 3819 | 2 | 1 |
| 8-Chloro-1-octanol (C8H17ClO) | YDFAJMDFCCJZSI-UHFFFAOYSA-N | C(CCCCCl)CCCO | 0 | 0 | 2 | 2 |
| Octanoyl chloride (C8H15ClO) | REEZZSHJLXOIHL-UHFFFAOYSA-N | CCCCCCCC(=O)Cl | 0 | 3140 | 2 | 1 |
| 2-ethylhexanoyl chloride (C8H15ClO) | WFSGQBNCVASPMW-UHFFFAOYSA-N | CCCCC(CC)C(=O)Cl | 0 | 309 | 2 | 1 |
| 1,8-Dichlorooctane (C8H16Cl2) | WXYMNDFVLNUAIA-UHFFFAOYSA-N | C(CCCCCl)CCCCl | 0 | 0 | 2 | 2 |
| 1-Chlorononane (C9H19Cl) | RKAMCQVGHFRILV-UHFFFAOYSA-N | CCCCCCCCCCl | 10 | 0 | 2 | 2 |
| Mucochloric acid (C4H2Cl2O3) | LUMLZKVIXLWTCI-NSCUHMNNSA-N | C(=O)/C(=C(/C(=O)O)\Cl)/Cl | 2950 | 9999 | 1 | 1 |
| Trichloroacetic acid 2-propenyl ester (C5H5Cl3O2) | LJQCONXCOYBYIE-UHFFFAOYSA-N | C=CCOC(=O)C(Cl)(Cl)Cl | 50 | 90 | 2 | 2 |
| 3-Chloropropionic acid, 3-chloroprop-2-enyl ester (C6H8Cl2O2) | CSADUUPDAFPXAX-HNQUOIGGSA-N | C(CCl)C(=O)OC/C=C/Cl | 60 | 1842 | 2 | 1 |
| Butanoic acid, 3-chloroprop-2-enyl ester (C7H11ClO2) | RVCJBAWEOOBVCN-HWKANZROSA-N | CCCC(=O)OC/C=C/Cl | 60 | 2963 | 2 | 1 |
| Hexachlorobutadiene (C4Cl6) | RWNKSTSCBHKHTB-UHFFFAOYSA-N | C(=C(Cl)Cl)(C(=C(Cl)Cl)Cl)Cl | 1700 | 6439 | 1 | 1 |
| 4-Pyrimidinamine, 6-chloro-2-(methylthio)- (C5H6ClN3S) | ISUXMAHVLFRZQU-UHFFFAOYSA-N | CSC1=NC(=CC(=N1)Cl)N | 9999 | 289 | 1 | 1 |
| Pyrimidine, 4,6-dichloro-2-(methylthio)- (C5H4Cl2N2S) | FCMLONIWOAGZJX-UHFFFAOYSA-N | CSC1=NC(=CC(=N1)Cl)Cl | 9999 | 3049 | 1 | 1 |
| 2-Chloro-5-nitropyridine (C5H3ClN2O2) | BAZVFQBTJPBRTJ-UHFFFAOYSA-N | C1=CC(=NC=C1[N+](=O)[O-])Cl | 5939 | 10 | 1 | 2 |
| 2-Chloro-3-nitropyridine (C5H3ClN2O2) | UUOLETYDNTVQDY-UHFFFAOYSA-N | C1=CC(=C(N=C1)Cl)[N+](=O)[O-] | 5291 | 0 | 1 | 2 |
| 2-Hydroxy-3,5,6-trichloropyridine (C5H2Cl3NO) | WCYYAQFQZQEUEN-UHFFFAOYSA-N | C1=C(C(=O)NC(=C1Cl)Cl)Cl | 9145 | 1505 | 1 | 1 |
| Benzenamine, 4-chloro-N-sulfinyl- (C6H4ClNOS) | GTKDDSPQJMLGOM-UHFFFAOYSA-N | C1=CC(=CC=C1N=S=O)Cl | 5400 | 9999 | 1 | 1 |
| 4-Chlorophenyl isothiocyanate (C7H4ClNS) | MZZVFXMTZTVUFO-UHFFFAOYSA-N | C1=CC(=CC=C1N=C=S)Cl | 9999 | 825 | 1 | 1 |
| 3-Chlorophenyl isothiocyanate (C7H4ClNS) | WGXCKFMVBAOIFH-UHFFFAOYSA-N | C1=CC(=CC(=C1)Cl)N=C=S | 9999 | 993 | 1 | 1 |
| 2-Chlorophenyl isothiocyanate (C7H4ClNS) | DASSPOJBUMBXLU-UHFFFAOYSA-N | C1=CC=C(C(=C1)N=C=S)Cl | 9999 | 1696 | 1 | 1 |
| 1-Chloro-4-Nitrobenzene (C6H4ClNO2) | CZGCEKJOLUNIFY-UHFFFAOYSA-N | C1=CC(=CC=C1[N+](=O)[O-])Cl | 8749 | 10 | 1 | 2 |
| 1-Chloro-3-nitrobenzene (C6H4ClNO2) | KMAQZIILEGKYQZ-UHFFFAOYSA-N | C1=CC(=CC(=C1)Cl)[N+](=O)[O-] | 6259 | 0 | 1 | 2 |
| Benzene, 1-chloro-2-nitro- (C6H4ClNO2) | BFCFYVKQTRLZHA-UHFFFAOYSA-N | C1=CC=C(C(=C1)[N+](=O)[O-])Cl | 8849 | 0 | 1 | 2 |
| 1-Chloro-4-isocyanatobenzene (C7H4ClNO) | ADAKRBAJFHTIEW-UHFFFAOYSA-N | C1=CC(=CC=C1N=C=O)Cl | 9999 | 10 | 1 | 2 |
| Formamide, N-(2-chlorophenyl)- (C7H6ClNO) | DGRDTMLQUWBPSM-UHFFFAOYSA-N | C1=CC=C(C(=C1)NC=O)Cl | 4549 | 9999 | 1 | 1 |
| 1-Chloro-3-isocyanatobenzene (C7H4ClNO) | HHIRBXHEYVDUAM-UHFFFAOYSA-N | C1=CC(=CC(=C1)Cl)N=C=O | 9999 | 30 | 1 | 2 |
| 1-Chloro-2-isocyanatobenzene (C7H4ClNO) | NOHQUGRVHSJYMR-UHFFFAOYSA-N | C1=CC=C(C(=C1)N=C=O)Cl | 9999 | 0 | 1 | 2 |
| Phenyl chloroformate (C7H5ClO2) | AHWALFGBDFAJAI-UHFFFAOYSA-N | C1=CC=C(C=C1)OC(=O)Cl | 4250 | 750 | 1 | 1 |
| 4-Chloro-2-methoxyphenol (C7H7ClO2) | FVZQMMMRFNURSH-UHFFFAOYSA-N | COC1=C(C=CC(=C1)Cl)O | 9088 | 70 | 1 | 2 |
| 2-Chloro-4-methoxyphenol (C7H7ClO2) | GNVRRKLFFYSLGT-UHFFFAOYSA-N | COC1=CC(=C(C=C1)O)Cl | 7267 | 40 | 1 | 2 |
| 2-Methoxy-3-chloro-phenol (C7H7ClO2) | JMRPFKGSKYTUSD-UHFFFAOYSA-N | COC1=C(C=CC=C1Cl)O | 6015 | 100 | 1 | 2 |
| m-Chloro-N,N-dimethylaniline (C8H10ClN) | CHHCCYVOJBBCIY-UHFFFAOYSA-N | CN(C)C1=CC(=CC=C1)Cl | 6946 | 130 | 1 | 1 |
| 2,6-Dichloronitrosobenzene (C6H3Cl2NO) | ICYPOGVRNGUWNP-UHFFFAOYSA-N | C1=CC(=C(C(=C1)Cl)N=O)Cl | 6825 | 0 | 1 | 2 |
| Hydrazine, (2,5-dichlorophenyl)- (C6H6Cl2N2) | LZKWWERBNXLGLI-UHFFFAOYSA-N | C1=CC(=C(C=C1Cl)NN)Cl | 9999 | 2589 | 1 | 1 |
| Benzene, 1-chloro-4-[(chloromethyl)thio]- (C7H6Cl2S) | XPJUCMIJGVAEGF-UHFFFAOYSA-N | C1=CC(=CC=C1SCCl)Cl | 1100 | 9999 | 1 | 1 |
| 4,5-Dichloro-ortho-phenylenediamine (C6H6Cl2N2) | IWFHBRFJOHTIPU-UHFFFAOYSA-N | C1=C(C(=CC(=C1Cl)Cl)N)N | 9999 | 1655 | 1 | 1 |
| α,para-Dichloroanisole (C7H6Cl2O) | PJLGIZXAYTZSIZ-UHFFFAOYSA-N | C1=CC(=CC=C1OCCl)Cl | 6639 | 9999 | 1 | 1 |
| Phenol, 4-amino-2,6-dichloro- (C6H5Cl2NO) | KGEXISHTCZHGFT-UHFFFAOYSA-N | C1=C(C=C(C(=C1Cl)O)Cl)N | 9999 | 250 | 1 | 1 |
| 1,3-Benzenediol, 4,6-dichloro- (C6H4Cl2O2) | GRLQBYQELUWBIO-UHFFFAOYSA-N | C1=C(C(=CC(=C1O)Cl)Cl)O | 9999 | 240 | 1 | 1 |
| 1,4-Benzenediol, 2,5-dichloro- (C6H4Cl2O2) | AYNPIRVEWMUJDE-UHFFFAOYSA-N | C1=C(C(=CC(=C1Cl)O)Cl)O | 9999 | 220 | 1 | 1 |
| Benzene, 1,4-dichloro-2-methoxy- (C7H6Cl2O) | QKMNFFSBZRGHDJ-UHFFFAOYSA-N | COC1=C(C=CC(=C1)Cl)Cl | 9999 | 470 | 1 | 1 |
| 2,3-Dichloroanisole (C7H6Cl2O) | HFEASCCDHUVYKU-UHFFFAOYSA-N | COC1=C(C(=CC=C1)Cl)Cl | 9999 | 269 | 1 | 1 |
| 2,6-Dichloroanisole (C7H6Cl2O) | KZLMCDNAVVJKPX-UHFFFAOYSA-N | COC1=C(C=CC=C1Cl)Cl | 9999 | 170 | 1 | 1 |
| 3,5-Dichloroanisole (C7H6Cl2O) | SSNXYMVLSOMJLU-UHFFFAOYSA-N | COC1=CC(=CC(=C1)Cl)Cl | 9999 | 259 | 1 | 1 |
| 3,4-Dichloroanisole (C7H6Cl2O) | VISJRVXHPNMYRH-UHFFFAOYSA-N | COC1=CC(=C(C=C1)Cl)Cl | 9999 | 751 | 1 | 1 |
| 2,4-Dichloroanisole (C7H6Cl2O) | CICQUFBZCADHHX-UHFFFAOYSA-N | COC1=C(C=C(C=C1)Cl)Cl | 9999 | 115 | 1 | 1 |
| Benzenamine, 2,3,4-trichloro- (C6H4Cl3N) | RRJUYQOFOMFVQS-UHFFFAOYSA-N | C1=CC(=C(C(=C1N)Cl)Cl)Cl | 9999 | 799 | 1 | 1 |
| 3,4,5-Trichloroaniline (C6H4Cl3N) | XOGYQVITULCUGU-UHFFFAOYSA-N | C1=C(C=C(C(=C1Cl)Cl)Cl)N | 9999 | 899 | 1 | 1 |
| 2,4,6-Trichloroaniline (C6H4Cl3N) | NATVSFWWYVJTAZ-UHFFFAOYSA-N | C1=C(C=C(C(=C1Cl)N)Cl)Cl | 9999 | 969 | 1 | 1 |
| Aniline, 2,4,5-trichloro- (C6H4Cl3N) | GUMCAKKKNKYFEB-UHFFFAOYSA-N | C1=C(C(=CC(=C1Cl)Cl)Cl)N | 9999 | 599 | 1 | 1 |
| Phenol, 2,3,4-trichloro- (C6H3Cl3O) | HSQFVBWFPBKHEB-UHFFFAOYSA-N | C1=CC(=C(C(=C1O)Cl)Cl)Cl | 9929 | 289 | 1 | 1 |
| Phenol, 3,4,5-trichloro- (C6H3Cl3O) | GBNHEBQXJVDXSW-UHFFFAOYSA-N | C1=C(C=C(C(=C1Cl)Cl)Cl)O | 9999 | 189 | 1 | 1 |
| 2,4,6-Trichlorophenol (C6H3Cl3O) | LINPIYWFGCPVIE-UHFFFAOYSA-N | C1=C(C=C(C(=C1Cl)O)Cl)Cl | 9999 | 119 | 1 | 1 |
| Phenol, 2,3,6-trichloro- (C6H3Cl3O) | XGCHAIDDPMFRLJ-UHFFFAOYSA-N | C1=CC(=C(C(=C1Cl)O)Cl)Cl | 9999 | 189 | 1 | 1 |
| Phenol, 2,3,5-trichloro- (C6H3Cl3O) | WWGQHTJIFOQAOC-UHFFFAOYSA-N | C1=C(C=C(C(=C1Cl)Cl)O)Cl | 9999 | 279 | 1 | 1 |
| Phenol, 2,4,5-trichloro- (C6H3Cl3O) | LHJGJYXLEPZJPM-UHFFFAOYSA-N | C1=C(C(=CC(=C1Cl)Cl)Cl)O | 9999 | 149 | 1 | 1 |
| 1,2,3,4-Tetrachlorobenzene (C6H2Cl4) | GBDZXPJXOMHESU-UHFFFAOYSA-N | C1=CC(=C(C(=C1Cl)Cl)Cl)Cl | 7359 | 2069 | 1 | 1 |
| 1,2,3,5-Tetrachlorobenzene (C6H2Cl4) | QZYNWJQFTJXIRN-UHFFFAOYSA-N | C1=C(C=C(C(=C1Cl)Cl)Cl)Cl | 7809 | 1849 | 1 | 1 |
| 1,2,4,5-Tetrachlorobenzene (C6H2Cl4) | JHBKHLUZVFWLAG-UHFFFAOYSA-N | C1=C(C(=CC(=C1Cl)Cl)Cl)Cl | 7889 | 1699 | 1 | 1 |
| 6-Chloronicotinic acid (C6H4ClNO2) | UAWMVMPAYRWUFX-UHFFFAOYSA-N | C1=CC(=NC=C1C(=O)O)Cl | 9999 | 519 | 1 | 1 |
| 2-Chloro-6-ethoxypyridine (C7H8ClNO) | AMSLPXHLKHZWBJ-UHFFFAOYSA-N | CCOC1=NC(=CC=C1)Cl | 3819 | 459 | 1 | 1 |
| 2-Chlorothiobenzamide (C7H6ClNS) | FLQYOORLPNYQEV-UHFFFAOYSA-N | C1=CC=C(C(=C1)C(=S)N)Cl | 7667 | 9999 | 1 | 1 |
| 4-Chlorothiobenzamide (C7H6ClNS) | OKPUICCJRDBRJT-UHFFFAOYSA-N | C1=CC(=CC=C1C(=S)N)Cl | 5375 | 0 | 1 | 2 |
| 4-Amino-3-chlorobenzonitrile (C7H5ClN2) | OREVCMGFYSUYPX-UHFFFAOYSA-N | C1=CC(=C(C=C1C#N)Cl)N | 9999 | 1429 | 1 | 1 |
| 6-Chloroanthranilonitrile (C7H5ClN2) | MEJVTQKBWPYBFG-UHFFFAOYSA-N | C1=CC(=C(C(=C1)Cl)C#N)N | 9999 | 1395 | 1 | 1 |
| 2-Chloroethyl phenyl ether (C8H9ClO) | VQUYNUJARXBNPK-UHFFFAOYSA-N | C1=CC=C(C=C1)OCCCl | 3609 | 149 | 1 | 1 |
| 3-Chlorobenzamide (C7H6ClNO) | MJTGQALMWUUPQM-UHFFFAOYSA-N | C1=CC(=CC(=C1)Cl)C(=O)N | 7229 | 10 | 1 | 2 |
| Benzamide, 4-chloro- (C7H6ClNO) | BLNVISNJTIRAHF-UHFFFAOYSA-N | C1=CC(=CC=C1C(=O)N)Cl | 4942 | 0 | 1 | 2 |
| Benzoic acid, 2-chloro- (C7H5ClO2) | IKCLCGXPQILATA-UHFFFAOYSA-N | C1=CC=C(C(=C1)C(=O)O)Cl | 7079 | 119 | 1 | 1 |
| Benzoic acid, 3-chloro- (C7H5ClO2) | LULAYUGMBFYYEX-UHFFFAOYSA-N | C1=CC(=CC(=C1)Cl)C(=O)O | 9999 | 0 | 1 | 2 |
| Benzoic acid, 4-chloro- (C7H5ClO2) | XRHGYUZYPHTUJZ-UHFFFAOYSA-N | C1=CC(=CC=C1C(=O)O)Cl | 8529 | 0 | 1 | 2 |
| 2-Chloro-4-hydroxybenzaldehyde (C7H5ClO2) | ZMOMCILMBYEGLD-UHFFFAOYSA-N | C1=CC(=C(C=C1O)Cl)C=O | 6196 | 0 | 1 | 2 |
| Benzaldehyde, 5-chloro-2-hydroxy- (C7H5ClO2) | FUGKCSRLAQKUHG-UHFFFAOYSA-N | C1=CC(=C(C=C1Cl)C=O)O | 9999 | 70 | 1 | 2 |
| Benzene, 1-(chloromethyl)-2-methoxy- (C8H9ClO) | UAWVMPOAIVZWFQ-UHFFFAOYSA-N | COC1=CC=CC=C1CCl | 2477 | 8369 | 1 | 1 |
| p-(chloromethyl)anisole (C8H9ClO) | MOHYOXXOKFQHDC-UHFFFAOYSA-N | COC1=CC=C(C=C1)CCl | 1200 | 9999 | 1 | 1 |
| Benzene, 1-(chloromethyl)-3-methoxy- (C8H9ClO) | VGISFWWEOGVMED-UHFFFAOYSA-N | COC1=CC=CC(=C1)CCl | 3909 | 9999 | 1 | 1 |
| Benzene, 1-chloro-3-ethoxy- (C8H9ClO) | HNMYSCABMYNGTP-UHFFFAOYSA-N | CCOC1=CC(=CC=C1)Cl | 2813 | 50 | 1 | 2 |
| Benzene, 1-chloro-2-ethoxy- (C8H9ClO) | IRYSAAMKXPLGAM-UHFFFAOYSA-N | CCOC1=CC=CC=C1Cl | 3053 | 10 | 1 | 2 |
| p-Chlorophenetole (C8H9ClO) | IXLSVQMYQRAMEW-UHFFFAOYSA-N | CCOC1=CC=C(C=C1)Cl | 3109 | 20 | 1 | 2 |
| Benzene, 1-chloro-2-methyl-4-methoxy (C8H9ClO) | SDGMUBWPXBSKCT-UHFFFAOYSA-N | CC1=C(C=CC(=C1)OC)Cl | 9999 | 3373 | 1 | 1 |
| 3-Chloro-4-methoxytoluene (C8H9ClO) | VUZBRBKYGIQXMP-UHFFFAOYSA-N | CC1=CC(=C(C=C1)OC)Cl | 5155 | 3833 | 1 | 1 |
| 2,4-Dichlorobenzyl mercaptan (C7H6Cl2S) | CEGBRSQPRQXALB-UHFFFAOYSA-N | C1=CC(=C(C=C1CS)Cl)Cl | 2504 | 168 | 1 | 1 |
| 3,4-Dichlorobenzyl mercaptan (C7H6Cl2S) | ZSPXTTVUJDSRNJ-UHFFFAOYSA-N | C1=CC(=C(C=C1Cl)Cl)CS | 2462 | 140 | 1 | 1 |
| Dichlobenil (C7H3Cl2N) | YOYAIZYFCNQIRF-UHFFFAOYSA-N | C1=CC(=C(C(=C1)Cl)C#N)Cl | 9999 | 2089 | 1 | 1 |
| 2,5-Dichlorobenzonitrile (C7H3Cl2N) | LNGWRTKJZCBXGT-UHFFFAOYSA-N | C1=CC(=C(C=C1Cl)C#N)Cl | 9999 | 2004 | 1 | 1 |
| 2,4-Dichlorobenzonitrile (C7H3Cl2N) | GRUHREVRSOOQJG-UHFFFAOYSA-N | C1=CC(=C(C=C1Cl)Cl)C#N | 9999 | 2152 | 1 | 1 |
| 3,4-Dichlorobenzonitrile (C7H3Cl2N) | KUWBYWUSERRVQP-UHFFFAOYSA-N | C1=CC(=C(C=C1C#N)Cl)Cl | 9999 | 2028 | 1 | 1 |
| 3,5-Dichlorobenzonitrile (C7H3Cl2N) | PUJSUOGJGIECFQ-UHFFFAOYSA-N | C1=C(C=C(C=C1Cl)Cl)C#N | 9999 | 1839 | 1 | 1 |
| 2,5-Dichlorobenzylamine (C7H7Cl2N) | AKGJLIXNRPNPCH-UHFFFAOYSA-N | C1=CC(=C(C=C1Cl)CN)Cl | 1372 | 9999 | 1 | 1 |
| 3,5-Dichlorobenzylamine (C7H7Cl2N) | ICIJWOWQUHHETJ-UHFFFAOYSA-N | C1=C(C=C(C=C1Cl)Cl)CN | 581 | 6259 | 2 | 1 |
| 2,4-Dichlorobenzylamine (C7H7Cl2N) | SJUKJZSTBBSGHF-UHFFFAOYSA-N | C1=CC(=C(C=C1Cl)Cl)CN | 710 | 9999 | 2 | 1 |
| 2,6-dichlorobenzyl alcohol (C7H6Cl2O) | WKKHCCZLKYKUDN-UHFFFAOYSA-N | C1=CC(=C(C(=C1)Cl)CO)Cl | 2845 | 4864 | 1 | 1 |
| 3,4-Dichlorobenzyl alcohol (C7H6Cl2O) | FVJIUQSKXOYFKG-UHFFFAOYSA-N | C1=CC(=C(C=C1CO)Cl)Cl | 6599 | 9669 | 1 | 1 |
| 2,5-dichlorobenzylic alcohol (C7H6Cl2O) | LCEIGNVIDJNUGF-UHFFFAOYSA-N | C1=CC(=C(C=C1Cl)CO)Cl | 5845 | 9999 | 1 | 1 |
| 3,5-dichlorobenzylic alcohol (C7H6Cl2O) | VSNNLLQKDRCKCB-UHFFFAOYSA-N | C1=C(C=C(C=C1Cl)Cl)CO | 3610 | 4594 | 1 | 1 |
| Benzoyl chloride, 4-chloro- (C7H4Cl2O) | RKIDDEGICSMIJA-UHFFFAOYSA-N | C1=CC(=CC=C1C(=O)Cl)Cl | 641 | 9999 | 2 | 1 |
| Benzoyl chloride, 2-chloro- (C7H4Cl2O) | ONIKNECPXCLUHT-UHFFFAOYSA-N | C1=CC=C(C(=C1)C(=O)Cl)Cl | 399 | 9999 | 2 | 1 |
| Benzoyl chloride, 3-chloro- (C7H4Cl2O) | WHIHIKVIWVIIER-UHFFFAOYSA-N | C1=CC(=CC(=C1)Cl)C(=O)Cl | 1079 | 9999 | 1 | 1 |
| 3,5-Dichlorobenzaldehyde (C7H4Cl2O) | CASRSOJWLARCRX-UHFFFAOYSA-N | C1=C(C=C(C=C1Cl)Cl)C=O | 7849 | 60 | 1 | 2 |
| Benzaldehyde, 3,4-dichloro- (C7H4Cl2O) | ZWUSBSHBFFPRNE-UHFFFAOYSA-N | C1=CC(=C(C=C1C=O)Cl)Cl | 6469 | 20 | 1 | 2 |
| Benzaldehyde, 2,6-dichloro- (C7H4Cl2O) | DMIYKWPEFRFTPY-UHFFFAOYSA-N | C1=CC(=C(C(=C1)Cl)C=O)Cl | 6139 | 50 | 1 | 2 |
| Phenol, 2,4-dichloro-6-methyl- (C7H6Cl2O) | WJQZZLQMLJPKQH-UHFFFAOYSA-N | CC1=CC(=CC(=C1O)Cl)Cl | 7049 | 9999 | 1 | 1 |
| α,3,4-Trichlorotoluene (C7H5Cl3) | YZIFVWOCPGPNHB-UHFFFAOYSA-N | C1=CC(=C(C=C1CCl)Cl)Cl | 1869 | 9999 | 1 | 1 |
| Benzene, 2,6-dichloro-1-(chloromethyl) (C7H5Cl3) | LBOBESSDSGODDD-UHFFFAOYSA-N | C1=CC(=C(C(=C1)Cl)CCl)Cl | 1869 | 9999 | 1 | 1 |
| Benzene, 1,3-dichloro-5-(chloromethyl)- (C7H5Cl3) | ZFLRKAMKGYNFPH-UHFFFAOYSA-N | C1=C(C=C(C=C1Cl)Cl)CCl | 1785 | 9999 | 1 | 1 |
| Benzene, 2,4-dichloro-1-(chloromethyl)- (C7H5Cl3) | IRSVDHPYXFLLDS-UHFFFAOYSA-N | C1=CC(=C(C=C1Cl)Cl)CCl | 1486 | 9999 | 1 | 1 |
| Benzene, (trichloromethyl)- (C7H5Cl3) | XEMRAKSQROQPBR-UHFFFAOYSA-N | C1=CC=C(C=C1)C(Cl)(Cl)Cl | 300 | 9999 | 2 | 1 |
| Toluene, 2,3,6-trichloro- (C7H5Cl3) | UZYYBZNZSSNYSA-UHFFFAOYSA-N | CC1=C(C=CC(=C1Cl)Cl)Cl | 4290 | 9999 | 1 | 1 |
| 2,4,5-Trichlorotoluene (C7H5Cl3) | ZCXHZKNWIYVQNC-UHFFFAOYSA-N | CC1=CC(=C(C=C1Cl)Cl)Cl | 5295 | 9999 | 1 | 1 |
| Benzene, 1,2,3-trichloro-4-methyl- (C7H5Cl3) | LHOGNQZQKDZOBP-UHFFFAOYSA-N | CC1=C(C(=C(C=C1)Cl)Cl)Cl | 3456 | 9999 | 1 | 1 |
| 2-Chloro-3-cyano-6-methylpyridine (C7H5ClN2) | YSBNBAYNISAUIT-UHFFFAOYSA-N | CC1=NC(=C(C=C1)C#N)Cl | 6788 | 1790 | 1 | 1 |
| Benzeneacetonitrile, 4-chloro- (C8H6ClN) | IVYMIRMKXZAHRV-UHFFFAOYSA-N | C1=CC(=CC=C1CC#N)Cl | 2689 | 9999 | 1 | 1 |
| Benzeneacetonitrile, 3-chloro- (C8H6ClN) | GTIKLPYCSAMPNG-UHFFFAOYSA-N | C1=CC(=CC(=C1)Cl)CC#N | 3179 | 9999 | 1 | 1 |
| 2-Chlorobenzyl cyanide (C8H6ClN) | MRDUURPIPLIGQX-UHFFFAOYSA-N | C1=CC=C(C(=C1)CC#N)Cl | 3563 | 9999 | 1 | 1 |
| Benzonitrile, 4-(chloromethyl)- (C8H6ClN) | LOQLDQJTSMKBJU-UHFFFAOYSA-N | C1=CC(=CC=C1CCl)C#N | 340 | 9999 | 2 | 1 |
| 2-(3-Chlorophenyl)ethylamine (C8H10ClN) | NRHVNPYOTNGECT-UHFFFAOYSA-N | C1=CC(=CC(=C1)Cl)CCN | 101 | 7 | 2 | 2 |
| Benzeneethanamine, 2-chloro- (C8H10ClN) | RZBOMSOHMOVUES-UHFFFAOYSA-N | C1=CC=C(C(=C1)CCN)Cl | 0 | 589 | 2 | 1 |
| 4-Chlorophenyl methyl carbinol (C8H9ClO) | HZFRKZWBVUJYDA-UHFFFAOYSA-N | C1=CC(=CC=C1CCO)Cl | 2692 | 40 | 1 | 2 |
| o-Chlorophenethyl alcohol (C8H9ClO) | IWNHTCBFRSCBQK-UHFFFAOYSA-N | C1=CC=C(C(=C1)CCO)Cl | 3163 | 90 | 1 | 2 |
| Benzeneethanol, 3-chloro- (C8H9ClO) | NDWAVJKRSASRPH-UHFFFAOYSA-N | C1=CC(=CC(=C1)Cl)CCO | 4109 | 30 | 1 | 2 |
| Benzeneacetyl chloride (C8H7ClO) | VMZCDNSFRSVYKQ-UHFFFAOYSA-N | C1=CC=C(C=C1)CC(=O)Cl | 1289 | 1019 | 1 | 1 |
| CN (C8H7ClO) | IMACFCSSMIZSPP-UHFFFAOYSA-N | C1=CC=C(C=C1)C(=O)CCl | 99 | 0 | 2 | 2 |
| 3-Chloro-4-methylbenzonitrile (C8H6ClN) | INEMHABDFCKBID-UHFFFAOYSA-N | CC1=C(C=C(C=C1)C#N)Cl | 4569 | 9999 | 1 | 1 |
| 4-Chloro-2-methylbenzonitrile (C8H6ClN) | ZZAJFWXXFZTTLH-UHFFFAOYSA-N | CC1=C(C=CC(=C1)Cl)C#N | 4563 | 9999 | 1 | 1 |
| 5-Chloro-2-methylbenzonitrile (C8H6ClN) | HFBZPKYQUFLCEL-UHFFFAOYSA-N | CC1=C(C=C(C=C1)Cl)C#N | 3813 | 9999 | 1 | 1 |
| 3-Chloro-2-methylbenzonitrile (C8H6ClN) | FKFZTNLSUJCIMG-UHFFFAOYSA-N | CC1=C(C=CC=C1Cl)C#N | 4306 | 9999 | 1 | 1 |
| Benzonitrile, 2-chloro-6-methyl- (C8H6ClN) | WQWQHJNUHQEGTN-UHFFFAOYSA-N | CC1=C(C(=CC=C1)Cl)C#N | 5093 | 9999 | 1 | 1 |
| 2-Chloro-6-methylbenzylamine (C8H10ClN) | WPQIAYXZZULKMV-UHFFFAOYSA-N | CC1=C(C(=CC=C1)Cl)CN | 896 | 1289 | 1 | 1 |
| Benzenemethanol, 2-chloro-α-methyl- (C8H9ClO) | DDUBOVLGCYUYFX-UHFFFAOYSA-N | CC(C1=CC=CC=C1Cl)O | 1679 | 359 | 1 | 1 |
| p-Chloro-α-methylbenzyl alcohol (C8H9ClO) | MVOSNPUNXINWAD-UHFFFAOYSA-N | CC(C1=CC=C(C=C1)Cl)O | 1962 | 1181 | 1 | 1 |
| 3-Chloro-4-methylbenzyl alcohol (C8H9ClO) | NLRJZTGNCBMNKS-UHFFFAOYSA-N | CC1=C(C=C(C=C1)CO)Cl | 4968 | 4629 | 1 | 1 |
| Benzoyl chloride, 3-methyl- (C8H7ClO) | YHOYYHYBFSYOSQ-UHFFFAOYSA-N | CC1=CC=CC(=C1)C(=O)Cl | 739 | 9999 | 2 | 1 |
| Benzoyl chloride, 4-methyl- (C8H7ClO) | NQUVCRCCRXRJCK-UHFFFAOYSA-N | CC1=CC=C(C=C1)C(=O)Cl | 429 | 9999 | 2 | 1 |
| Benzoyl chloride, 2-methyl- (C8H7ClO) | GPZXFICWCMCQPF-UHFFFAOYSA-N | CC1=CC=CC=C1C(=O)Cl | 449 | 9999 | 2 | 1 |
| o-Chloroacetophenone (C8H7ClO) | ZDOYHCIRUPHUHN-UHFFFAOYSA-N | CC(=O)C1=CC=CC=C1Cl | 1879 | 0 | 1 | 2 |
| Acetophenone, 3'-chloro- (C8H7ClO) | UUWJBXKHMMQDED-UHFFFAOYSA-N | CC(=O)C1=CC(=CC=C1)Cl | 3469 | 10 | 1 | 2 |
| Acetophenone, 4'-chloro- (C8H7ClO) | BUZYGTVTZYSBCU-UHFFFAOYSA-N | CC(=O)C1=CC=C(C=C1)Cl | 2669 | 0 | 1 | 2 |
| Phenol, 4-chloro-2,6-dimethyl- (C8H9ClO) | VWYKSJIPZHRLNO-UHFFFAOYSA-N | CC1=CC(=CC(=C1O)C)Cl | 7216 | 9999 | 1 | 1 |
| Phenol, 2-chloro-4,5-dimethyl- (C8H9ClO) | PSOJLBXHRBFLLQ-UHFFFAOYSA-N | CC1=CC(=C(C=C1C)Cl)O | 5405 | 9999 | 1 | 1 |
| 4-chloro-3,5-dimethylphenol (C8H9ClO) | OSDLLIBGSJNGJE-UHFFFAOYSA-N | CC1=CC(=CC(=C1Cl)C)O | 5870 | 9999 | 1 | 1 |
| Benzene, (1,2-dichloroethyl)- (C8H8Cl2) | GCXHSBQTVXCWBK-UHFFFAOYSA-N | C1=CC=C(C=C1)C(CCl)Cl | 1839 | 2179 | 1 | 1 |
| Benzene, 1,2-bis(chloromethyl)- (C8H8Cl2) | FMGGHNGKHRCJLL-UHFFFAOYSA-N | C1=CC=C(C(=C1)CCl)CCl | 2659 | 9999 | 1 | 1 |
| Benzene, 1,4-bis(chloromethyl)- (C8H8Cl2) | ZZHIDJWUJRKHGX-UHFFFAOYSA-N | C1=CC(=CC=C1CCl)CCl | 2079 | 9999 | 1 | 1 |
| Benzene, 1,3-bis(chloromethyl)- (C8H8Cl2) | GRJWOKACBGZOKT-UHFFFAOYSA-N | C1=CC(=CC(=C1)CCl)CCl | 2089 | 9999 | 1 | 1 |
| Benzene, 1-(dichloromethyl)-4-methyl- (C8H8Cl2) | RGDYIHSZBVIIND-UHFFFAOYSA-N | CC1=CC=C(C=C1)C(Cl)Cl | 1661 | 9999 | 1 | 1 |
| 3-Chloro-1-(Chloromethyl)-4-methylbenzene (C8H8Cl2) | JTMYLQKKQFLIGV-UHFFFAOYSA-N | CC1=C(C=C(C=C1)CCl)Cl | 1806 | 9999 | 1 | 1 |
| Benzene, 1,3-dichloro-2-ethyl- (C8H8Cl2) | NUDVJQOVBFONPG-UHFFFAOYSA-N | CCC1=C(C=CC=C1Cl)Cl | 2893 | 1421 | 1 | 1 |
| Benzene, 1,2-dichloro-3-ethyl- (C8H8Cl2) | KEBKSRNCCOFIBI-UHFFFAOYSA-N | CCC1=C(C(=CC=C1)Cl)Cl | 4244 | 6166 | 1 | 1 |
| 2,4-Dichloroethylbenzene (C8H8Cl2) | MJTYPPBFTMKLGA-UHFFFAOYSA-N | CCC1=C(C=C(C=C1)Cl)Cl | 3894 | 4564 | 1 | 1 |
| Benzene, 2,4-dichloro-4-ethyl (C8H8Cl2) | PZPSDDYNMXBZOA-UHFFFAOYSA-N | CCC1=C(C=CC(=C1)Cl)Cl | 4464 | 5225 | 1 | 1 |
| Benzene, 1,2-dichloro-4-ethyl- (C8H8Cl2) | LAGWZWJDSQHXQZ-UHFFFAOYSA-N | CCC1=CC(=C(C=C1)Cl)Cl | 4044 | 4844 | 1 | 1 |
| p-Xylene, 2,5-dichloro- (C8H8Cl2) | UTGSRNVBAFCOEU-UHFFFAOYSA-N | CC1=CC(=C(C=C1Cl)C)Cl | 5739 | 9999 | 1 | 1 |
| Chloroacetic acid, morpholide (C6H10ClNO2) | YMQRPXBBBOXHNZ-UHFFFAOYSA-N | C1COCCN1C(=O)CCl | 3793 | 9999 | 1 | 1 |
| Benzene, (3-chloropropyl)- (C9H11Cl) | XZBXAYCCBFTQHH-UHFFFAOYSA-N | C1=CC=C(C=C1)CCCCl | 2697 | 262 | 1 | 1 |
| Benzene, [1-(chloromethyl)ethyl] (C9H11Cl) | SXVRSCIZJBGJGB-UHFFFAOYSA-N | CC(CCl)C1=CC=CC=C1 | 1551 | 70 | 1 | 2 |
| 4-(Chloromethyl)-1-ethylbenzene (C9H11Cl) | DUBCVXSYZVTCOC-UHFFFAOYSA-N | CCC1=CC=C(C=C1)CCl | 3194 | 9999 | 1 | 1 |
| o-Chloropropylbenzene (C9H11Cl) | WRZSIHUUDJFHNY-UHFFFAOYSA-N | CCCC1=CC=CC=C1Cl | 2392 | 1001 | 1 | 1 |
| 1,2-Dimethyl-4-(chloromethyl)benzene (C9H11Cl) | UBQRAAXAHIKWRI-UHFFFAOYSA-N | CC1=C(C=C(C=C1)CCl)C | 2410 | 9999 | 1 | 1 |
| 3,5-Dimethylbenzyl chloride (C9H11Cl) | FYNVRRYQTHUESZ-UHFFFAOYSA-N | CC1=CC(=CC(=C1)CCl)C | 2100 | 9999 | 1 | 1 |
| Benzene, 2-(chloromethyl)-1,4-dimethyl- (C9H11Cl) | PECXPZGFZFGDRD-UHFFFAOYSA-N | CC1=CC(=C(C=C1)C)CCl | 1942 | 9999 | 1 | 1 |
| 2,4-Dimethylbenzyl chloride (C9H11Cl) | BETNPSBTDMBHCZ-UHFFFAOYSA-N | CC1=CC(=C(C=C1)CCl)C | 1651 | 9999 | 1 | 1 |
| Benzene, 1-chloro-2-(1-methylethyl)- (C9H11Cl) | RNEMUWDQJSRDMQ-UHFFFAOYSA-N | CC(C)C1=CC=CC=C1Cl | 2973 | 561 | 1 | 1 |
| Benzene, 1-chloro-4-(1-methylethyl)- (C9H11Cl) | FHBSIIZALGOVLM-UHFFFAOYSA-N | CC(C)C1=CC=C(C=C1)Cl | 7206 | 1982 | 1 | 1 |
| Benzene, 2-chloro-1,3,5-trimethyl- (C9H11Cl) | WDZACGWEPQLKOM-UHFFFAOYSA-N | CC1=CC(=C(C(=C1)C)Cl)C | 5359 | 9999 | 1 | 1 |
| 1,3-Dioxolane, 2-(3-chloropropyl)-2-methyl- (C7H13ClO2) | OFERIRWCHSOJJT-UHFFFAOYSA-N | CC1(OCCO1)CCCCl | 0 | 10 | 2 | 2 |
| Cyclopropanecarboxylic acid, 2,2-dichloro-1-methyl-, methyl ester (C6H8Cl2O2) | JGCAZFUFORGMFB-UHFFFAOYSA-N | CC1(CC1(Cl)Cl)C(=O)OC | 1709 | 4044 | 1 | 1 |
| 1H-Imidazole, 5-chloro-1-methyl-4-nitro- (C4H4ClN3O2) | OSJUNMSWBBOTQU-UHFFFAOYSA-N | CN1C=NC(=C1Cl)[N+](=O)[O-] | 5500 | 0 | 1 | 2 |
| Benzene, 1,4-dichloro-2-ethenyl- (C8H6Cl2) | IZMZREOTRMMCCB-UHFFFAOYSA-N | C=CC1=C(C=CC(=C1)Cl)Cl | 9999 | 5555 | 1 | 1 |
| 3,4-Dichlorostyrene (C8H6Cl2) | BJQFWAQRPATHTR-UHFFFAOYSA-N | C=CC1=CC(=C(C=C1)Cl)Cl | 9999 | 3477 | 1 | 1 |
| 2,6-Dichlorostyrene (C8H6Cl2) | YJCVRMIJBXTMNR-UHFFFAOYSA-N | C=CC1=C(C=CC=C1Cl)Cl | 8378 | 9999 | 1 | 1 |
| 4,5-Dichloro-2-methylpyridazin-3-one (C5H4Cl2N2O) | ACKBTCUMGAHRIE-UHFFFAOYSA-N | CN1C(=O)C(=C(C=N1)Cl)Cl | 6861 | 791 | 1 | 1 |
| 3-Phenyl-2-propenyl chloride (C9H9Cl) | IWTYTFSSTWXZFU-QPJJXVBHSA-N | C1=CC=C(C=C1)/C=C/CCl | 1989 | 9999 | 1 | 1 |
| Benzene, (3-chloroallyl)- (C9H9Cl) | JJTUJRVKTPSEFZ-XBXARRHUSA-N | C1=CC=C(C=C1)C/C=C/Cl | 2482 | 9999 | 1 | 1 |
| Benzene, 1-chloro-4-(1-methylethenyl)- (C9H9Cl) | WQDGTJOEMPEHHL-UHFFFAOYSA-N | CC(=C)C1=CC=C(C=C1)Cl | 8863 | 9999 | 1 | 1 |
| Cyclopropanecarboxylic acid, 3-chloroprop-2-enyl ester (C7H9ClO2) | QAJSCZWJFSRULT-DAFODLJHSA-N | C1CC1C(=O)OC/C=C/Cl | 0 | 2563 | 2 | 1 |
| 3-Chloro-5,5-dimethylcyclohex-2-enone (C8H11ClO) | PJYTYJGMJDIKEJ-UHFFFAOYSA-N | CC1(CC(=CC(=O)C1)Cl)C | 1678 | 43 | 1 | 2 |
| 3-Acetyl-2,5-dichlorothiophene (C6H4Cl2OS) | GYFDNIRENHZKGR-UHFFFAOYSA-N | CC(=O)C1=C(SC(=C1)Cl)Cl | 3553 | 0 | 1 | 2 |
| 2,5-Cyclohexadiene-1,4-dione, 2,5-dichloro- (C6H2Cl2O2) | LNXVNZRYYHFMEY-UHFFFAOYSA-N | C1=C(C(=O)C=C(C1=O)Cl)Cl | 8799 | 369 | 1 | 1 |
| 2,5-Cyclohexadiene-1,4-dione, 2,6-dichloro- (C6H2Cl2O2) | JCARTGJGWCGSSU-UHFFFAOYSA-N | C1=C(C(=O)C(=CC1=O)Cl)Cl | 9999 | 2329 | 1 | 1 |
| 6-Chloropurine (C5H3ClN4) | ZKBQDFAWXLTYKS-UHFFFAOYSA-N | C1=NC2=C(N1)C(=NC=N2)Cl | 9999 | 5882 | 1 | 1 |
| 2-Chlorobenzimidazole (C7H5ClN2) | AYPSHJCKSDNETA-UHFFFAOYSA-N | C1=CC=C2C(=C1)NC(=N2)Cl | 9999 | 821 | 1 | 1 |
| Benzothiazole, 2-chloro- (C7H4ClNS) | BSQLQMLFTHJVKS-UHFFFAOYSA-N | C1=CC=C2C(=C1)N=C(S2)Cl | 9999 | 1409 | 1 | 1 |
| Benzoxazole, 2-chloro- (C7H4ClNO) | BBVQDWDBTWSGHQ-UHFFFAOYSA-N | C1=CC=C2C(=C1)N=C(O2)Cl | 9999 | 459 | 1 | 1 |
| 5-Chloro-1,3-benzodioxole (C7H5ClO2) | ODQPZHOXLYATLC-UHFFFAOYSA-N | C1OC2=C(O1)C=C(C=C2)Cl | 7259 | 141 | 1 | 1 |
| 4-chloroepoxystyrene (C8H7ClO) | IBWLXNDOMYKTAD-UHFFFAOYSA-N | C1C(O1)C2=CC=C(C=C2)Cl | 3939 | 6499 | 1 | 1 |
| 2-Norbornane carbonyl chloride (C8H11ClO) | GDBUZLAZTFSUEL-UHFFFAOYSA-N | C1CC2CC1CC2C(=O)Cl | 24 | 1619 | 2 | 1 |
| 6-Chloroindole (C8H6ClN) | YTYIMDRWPTUAHP-UHFFFAOYSA-N | C1=CC(=CC2=C1C=CN2)Cl | 9999 | 1539 | 1 | 1 |
| 1H-Indole, 4-chloro- (C8H6ClN) | SVLZRCRXNHITBY-UHFFFAOYSA-N | C1=CC2=C(C=CN2)C(=C1)Cl | 9999 | 1669 | 1 | 1 |
| 5-Norbornene-2-carbonyl chloride (C8H9ClO) | HXYXVFUUHSZSNV-UHFFFAOYSA-N | C1C2CC(C1C=C2)C(=O)Cl | 301 | 533 | 2 | 1 |
| Methane, chlorotrinitro- (CClN3O6) | GNZCDDDPGSFZFG-UHFFFAOYSA-N | C([N+](=O)[O-])([N+](=O)[O-])([N+](=O)[O-])Cl | 0 | 173 | 2 | 1 |
| Chlormephos (C5H12ClO2PS2) | QGTYWWGEWOBMAK-UHFFFAOYSA-N | CCOP(=S)(OCC)SCCl | 3590 | 190 | 1 | 1 |
| Dichloroacetic anhydride (C4H2Cl4O3) | RQHMQURGSQBBJY-UHFFFAOYSA-N | C(C(=O)OC(=O)C(Cl)Cl)(Cl)Cl | 0 | 0 | 2 | 2 |
| Propanoic acid, pentachloro-, methyl ester (C4H3Cl5O2) | YSCHNGJMUJYQJG-UHFFFAOYSA-N | COC(=O)C(C(Cl)(Cl)Cl)(Cl)Cl | 0 | 0 | 2 | 2 |
| Octachloropropane (C3Cl8) | QQAHAGNPDBPSJP-UHFFFAOYSA-N | C(C(Cl)(Cl)Cl)(C(Cl)(Cl)Cl)(Cl)Cl | 0 | 10 | 2 | 2 |
| Phosphonic acid, (2-chloroethyl)-, diethyl ester (C6H14ClO3P) | GMDLEOVIACJWTD-UHFFFAOYSA-N | CCOP(=O)(CCCl)OCC | 0 | 7725 | 2 | 1 |
| Trichloroacetic acid, but-3-yn-2-yl ester (C6H5Cl3O2) | KEBRGWUCOLOSMN-UHFFFAOYSA-N | CC(C#C)OC(=O)C(Cl)(Cl)Cl | 0 | 130 | 2 | 1 |
| 1,3-bis-(2-Chloroethylthio)propane (C7H14Cl2S2) | YHRGRBPJIRKFND-UHFFFAOYSA-N | C(CSCCCl)CSCCCl | 1111 | 0 | 1 | 2 |
| Butyl trichloroacetate (C6H9Cl3O2) | SECVZLDDYUWJAC-UHFFFAOYSA-N | CCCCOC(=O)C(Cl)(Cl)Cl | 0 | 0 | 2 | 2 |
| Isobutyl trichloroacetate (C6H9Cl3O2) | LRASRNYGUIRRSW-UHFFFAOYSA-N | CC(C)COC(=O)C(Cl)(Cl)Cl | 0 | 110 | 2 | 1 |
| Acetic acid, trichloro, 1-methylpropyl ester (C6H9Cl3O2) | CJTKROHEGUUXOZ-UHFFFAOYSA-N | CCC(C)OC(=O)C(Cl)(Cl)Cl | 0 | 100 | 2 | 2 |
| Hexanedioic acid monochloride monomethyl ester (C7H11ClO3) | HDLGIEZOMYJKAK-UHFFFAOYSA-N | COC(=O)CCCCC(=O)Cl | 20 | 1729 | 2 | 1 |
| 6-Chlorohexanoic acid, chloromethyl ester (C7H12Cl2O2) | NFZSDYANLAMKQW-UHFFFAOYSA-N | C(CCC(=O)OCCl)CCCl | 0 | 0 | 2 | 2 |
| 5-Chlorohexanoic acid, chloromethyl ester (C7H12Cl2O2) | PZVNORNNHRGBRP-UHFFFAOYSA-N | CC(CCCC(=O)OCCl)Cl | 0 | 0 | 2 | 2 |
| 4-Chlorohexanoic acid, chloromethyl ester (C7H12Cl2O2) | SKALGZTWKDDAHB-UHFFFAOYSA-N | CCC(CCC(=O)OCCl)Cl | 0 | 0 | 2 | 2 |
| 3-Chlorohexanoic acid, chloromethyl ester (C7H12Cl2O2) | FWVZTAJOBMVDDT-UHFFFAOYSA-N | CCCC(CC(=O)OCCl)Cl | 0 | 290 | 2 | 1 |
| Pentyl dichloroacetate (C7H12Cl2O2) | UIFCHGLUUASAAI-UHFFFAOYSA-N | CCCCCOC(=O)C(Cl)Cl | 0 | 0 | 2 | 2 |
| 2-Chlorohexanoic acid, chloromethyl ester (C7H12Cl2O2) | SLCVMKYCEWYVKS-UHFFFAOYSA-N | CCCCC(C(=O)OCCl)Cl | 0 | 0 | 2 | 2 |
| Acetic acid, dichloro, 3-methylbutyl ester (C7H12Cl2O2) | UQQSGXRKRFDREY-UHFFFAOYSA-N | CC(C)CCOC(=O)C(Cl)Cl | 0 | 0 | 2 | 2 |
| Acetic acid, dichloro, 1-methylbutyl ester (C7H12Cl2O2) | BSBLGRPLLFXELO-UHFFFAOYSA-N | CCCC(C)OC(=O)C(Cl)Cl | 0 | 20 | 2 | 2 |
| Propanoic acid, 2,2-dichloro, butyl ester (C7H12Cl2O2) | INWCHAUMYGSQAZ-UHFFFAOYSA-N | CCCCOC(=O)C(C)(Cl)Cl | 40 | 40 | 2 | 2 |
| Hexyl chloroacetate (C8H15ClO2) | OJGRZJILAIHWIY-UHFFFAOYSA-N | CCCCCCOC(=O)CCl | 0 | 0 | 2 | 2 |
| Propanoic acid, 3-chloro, pentyl ester (C8H15ClO2) | VSUKVMJYRSUJJS-UHFFFAOYSA-N | CCCCCOC(=O)CCCl | 0 | 0 | 2 | 2 |
| Heptanoic acid, chloromethyl ester (C8H15ClO2) | JUBQFNSRJOLWED-UHFFFAOYSA-N | CCCCCCC(=O)OCCl | 0 | 0 | 2 | 2 |
| Propanoic acid, 3-chloro-, 3-methylbutyl ester (C8H15ClO2) | ZFNWFRKLGAIOME-UHFFFAOYSA-N | CC(C)CCOC(=O)CCCl | 0 | 30 | 2 | 2 |
| Propanoic acid, 3-chloro, 1-methylbutyl ester (C8H15ClO2) | ZODXJJOAYWVAGY-UHFFFAOYSA-N | CCCC(C)OC(=O)CCCl | 0 | 0 | 2 | 2 |
| Propanoic acid, 2-chloro, pentyl ester (C8H15ClO2) | ASYLXRAVZWKEBQ-UHFFFAOYSA-N | CCCCCOC(=O)C(C)Cl | 35 | 10 | 2 | 2 |
| Propanoic acid, 2-chloro, 1-methylbutyl ester (C8H15ClO2) | QTQYTMIPKPUVCH-UHFFFAOYSA-N | CCCC(C)OC(=O)C(C)Cl | 90 | 0 | 2 | 2 |
| Butane, 1,1'-oxybis[4-chloro- (C8H16Cl2O) | PVBMXMKIKMJQRK-UHFFFAOYSA-N | C(CCCl)COCCCCCl | 99 | 10 | 2 | 2 |
| Pimeloylchloride (C7H10Cl2O2) | LVIMBOHJGMDKEJ-UHFFFAOYSA-N | C(CCC(=O)Cl)CCC(=O)Cl | 0 | 5136 | 2 | 1 |
| 8-Chloro-1-octanol, methyl ether (C9H19ClO) | AGXFBKIMLRSQTG-UHFFFAOYSA-N | COCCCCCCCCCl | 0 | 0 | 2 | 2 |
| Nonanoyl chloride (C9H17ClO) | NTQYXUJLILNTFH-UHFFFAOYSA-N | CCCCCCCCC(=O)Cl | 0 | 620 | 2 | 1 |
| 1,9-Dichlorononane (C9H18Cl2) | JMGRNJZUQCEJDB-UHFFFAOYSA-N | C(CCCCCl)CCCCCl | 0 | 0 | 2 | 2 |
| 1-Chloro-4-decyne (C10H17Cl) | WQROTKWHQIGFTO-UHFFFAOYSA-N | CCCCCC#CCCCCl | 0 | 554 | 2 | 1 |
| 1-Chlorodecane (C10H21Cl) | ZTEHOZMYMCEYRM-UHFFFAOYSA-N | CCCCCCCCCCCl | 10 | 0 | 2 | 2 |
| 3-Chlorodecane (C10H21Cl) | SMVZPOXWOUHGQI-UHFFFAOYSA-N | CCCCCCCC(CC)Cl | 0 | 622 | 2 | 1 |
| 2-Chloro-2-methylnonane (C10H21Cl) | QDJXQCKHBZEIJM-UHFFFAOYSA-N | CCCCCCCC(C)(C)Cl | 0 | 183 | 2 | 1 |
| 5-Chloro-5-methylnonane (C10H21Cl) | FYIAPWZQRBQNSP-UHFFFAOYSA-N | CCCCC(C)(CCCC)Cl | 0 | 243 | 2 | 1 |
| Dichlorvos (C4H7Cl2O4P) | OEBRKCOSUFCWJD-UHFFFAOYSA-N | COP(=O)(OC)OC=C(Cl)Cl | 522 | 2791 | 2 | 1 |
| Trichloroacetic acid, 3-chloroprop-2-enyl ester (C5H4Cl4O2) | KPJDOQORQYKCEB-OWOJBTEDSA-N | C(/C=C/Cl)OC(=O)C(Cl)(Cl)Cl | 120 | 240 | 2 | 1 |
| Dichloroacetic acid, pent-2-en-4-ynyl ester (C7H6Cl2O2) | NNFFABBXUJXUSY-UHFFFAOYSA-N | C#CC=CCOC(=O)C(Cl)Cl | 120 | 360 | 2 | 1 |
| 3-Chloropropionic acid, pent-2-en-4-ynyl ester (C8H9ClO2) | JBIDPFGDHSPIBB-UHFFFAOYSA-N | C#CC=CCOC(=O)CCCl | 160 | 0 | 2 | 2 |
| valeric acid, 3-chloroprop-2-enyl ester (C8H13ClO2) | OLCICKDOYCBKFY-GQCTYLIASA-N | CCCCC(=O)OC/C=C/Cl | 70 | 2492 | 2 | 1 |
| 3-Chloropropionic acid, 3-methylbut-2-enyl ester (C8H13ClO2) | JQSINDIMIDHUQE-UHFFFAOYSA-N | CC(=CCOC(=O)CCCl)C | 60 | 0 | 2 | 2 |
| Methyl 4-chloro-2,2-dimethyl-4-pentenoate (C8H13ClO2) | NMIYZQVNXZOOPF-UHFFFAOYSA-N | CC(C)(CC(=C)Cl)C(=O)OC | 0 | 9999 | 2 | 1 |
| 2,2-Dimethylpropanoic acid, 3-chloroprop-2-enyl ester (C8H13ClO2) | HEINMRDBMAQWSG-SNAWJCMRSA-N | CC(C)(C)C(=O)OC/C=C/Cl | 120 | 3904 | 2 | 1 |
| CDAA (C8H12ClNO) | MDBGGTQNNUOQRC-UHFFFAOYSA-N | C=CCN(CC=C)C(=O)CCl | 700 | 3700 | 2 | 1 |
| 3-Methyl-2-butenoic acid, 3-chloroprop-2-enyl ester (C8H11ClO2) | UHEWJISEFLFVKJ-ONEGZZNKSA-N | CC(=CC(=O)OC/C=C/Cl)C | 0 | 2743 | 2 | 1 |
| Pyrimidine, 4,6-dichloro-5-nitro (C4HCl2N3O2) | HCTISZQLTGAYOX-UHFFFAOYSA-N | C1=NC(=C(C(=N1)Cl)[N+](=O)[O-])Cl | 8969 | 0 | 1 | 2 |
| Pyridine, pentachloro- (C5Cl5N) | DNDPLEAVNVOOQZ-UHFFFAOYSA-N | C1(=C(C(=NC(=C1Cl)Cl)Cl)Cl)Cl | 6135 | 2492 | 1 | 1 |
| 3-Chlorobenzenesulfonamide (C6H6ClNO2S) | WSYQJNPRQUFCGL-UHFFFAOYSA-N | C1=CC(=CC(=C1)Cl)S(=O)(=O)N | 4191 | 0 | 1 | 2 |
| 3-Chlorobenzenesulfonyl chloride (C6H4Cl2O2S) | OINWZUJVEXUHCC-UHFFFAOYSA-N | C1=CC(=CC(=C1)Cl)S(=O)(=O)Cl | 1647 | 4573 | 1 | 1 |
| 2-Chlorobenzenesulfonyl chloride (C6H4Cl2O2S) | KMVZDSQHLDGKGV-UHFFFAOYSA-N | C1=CC=C(C(=C1)S(=O)(=O)Cl)Cl | 2000 | 6518 | 1 | 1 |
| 2-Chlorobenzenesulfonamide (C6H6ClNO2S) | JCCBZCMSYUSCFM-UHFFFAOYSA-N | C1=CC=C(C(=C1)S(=O)(=O)N)Cl | 5037 | 680 | 1 | 1 |
| Benzenesulfonyl chloride, 4-chloro- (C6H4Cl2O2S) | ZLYBFBAHAQEEQQ-UHFFFAOYSA-N | C1=CC(=CC=C1S(=O)(=O)Cl)Cl | 1559 | 6529 | 1 | 1 |
| Benzenesulfonamide, 4-chloro- (C6H6ClNO2S) | HHHDJHHNEURCNV-UHFFFAOYSA-N | C1=CC(=CC=C1S(=O)(=O)N)Cl | 5039 | 0 | 1 | 2 |
| 4-Chlorobenzenesulfonic acid (C6H5ClO3S) | RJWBTWIBUIGANW-UHFFFAOYSA-N | C1=CC(=CC=C1S(=O)(=O)O)Cl | 2381 | 0 | 1 | 2 |
| Thiourea, (2-chlorophenyl)- (C7H7ClN2S) | YZUKKTCDYSIWKJ-UHFFFAOYSA-N | C1=CC=C(C(=C1)NC(=S)N)Cl | 0 | 0 | 2 | 2 |
| Benzenamine, 2-chloro-4-nitro- (C6H5ClN2O2) | LOCWBQIWHWIRGN-UHFFFAOYSA-N | C1=CC(=C(C=C1[N+](=O)[O-])Cl)N | 9999 | 70 | 1 | 2 |
| Benzenamine, 2-chloro-5-nitro- (C6H5ClN2O2) | KWIXNFOTNVKIGM-UHFFFAOYSA-N | C1=CC(=C(C=C1[N+](=O)[O-])N)Cl | 9649 | 30 | 1 | 2 |
| Benzenamine, 4-chloro-3-nitro- (C6H5ClN2O2) | FOHHWGVAOVDVLP-UHFFFAOYSA-N | C1=CC(=C(C=C1N)[N+](=O)[O-])Cl | 9999 | 509 | 1 | 1 |
| Aniline, 4-chloro-2-nitro- (C6H5ClN2O2) | PBGKNXWGYQPUJK-UHFFFAOYSA-N | C1=CC(=C(C=C1Cl)[N+](=O)[O-])N | 9999 | 0 | 1 | 2 |
| Phenol, 5-chloro-2-nitro- (C6H4ClNO3) | MZDBQSFPAMTTIS-UHFFFAOYSA-N | C1=CC(=C(C=C1Cl)O)[N+](=O)[O-] | 9999 | 0 | 1 | 2 |
| Phenol, 2-chloro-4-nitro- (C6H4ClNO3) | BOFRXDMCQRTGII-UHFFFAOYSA-N | C1=CC(=C(C=C1[N+](=O)[O-])Cl)O | 9920 | 30 | 1 | 2 |
| Urea, (4-chlorophenyl)- (C7H7ClN2O) | RECCURWJDVZHIH-UHFFFAOYSA-N | C1=CC(=CC=C1NC(=O)N)Cl | 1320 | 0 | 1 | 2 |
| Carbamic chloride, methylphenyl- (C8H8ClNO) | CPGWSLFYXMRNDV-UHFFFAOYSA-N | CN(C1=CC=CC=C1)C(=O)Cl | 2550 | 9999 | 1 | 1 |
| Benzene, 4-chloro-1,2-dimethoxy (C8H9ClO2) | RXJCXGJKJZARAW-UHFFFAOYSA-N | COC1=C(C=C(C=C1)Cl)OC | 9999 | 30 | 1 | 2 |
| 2-Chloro-4-methoxyphenol, methyl ether (C8H9ClO2) | QMXZSRVFIWACJH-UHFFFAOYSA-N | COC1=CC(=C(C=C1)OC)Cl | 5915 | 0 | 1 | 2 |
| 1-Chloro-2,4-dimethoxybenzene (C8H9ClO2) | OACCRGFGCIQFNR-UHFFFAOYSA-N | COC1=CC(=C(C=C1)Cl)OC | 9999 | 552 | 1 | 1 |
| Benzene, 3-chloro-1,2-dimethoxy (C8H9ClO2) | JVCXXMPHTZGGNV-UHFFFAOYSA-N | COC1=C(C(=CC=C1)Cl)OC | 9999 | 10 | 1 | 2 |
| 2,5-Dichlorophenyl isothiocyanate (C7H3Cl2NS) | JHTPBGFVWWSHDL-UHFFFAOYSA-N | C1=CC(=C(C=C1Cl)N=C=S)Cl | 9999 | 1388 | 1 | 1 |
| 3,5-Dichlorophenyl isothiocyanate (C7H3Cl2NS) | JLEMKZDHFGCHLO-UHFFFAOYSA-N | C1=C(C=C(C=C1Cl)Cl)N=C=S | 9999 | 1345 | 1 | 1 |
| 3,4-Dichlorophenyl isothiocyanate (C7H3Cl2NS) | OSBIEFWIIINTNJ-UHFFFAOYSA-N | C1=CC(=C(C=C1N=C=S)Cl)Cl | 9999 | 1171 | 1 | 1 |
| 2,6-Dichlorophenyl isothiocyanate (C7H3Cl2NS) | SUCGVQHNGIQXGD-UHFFFAOYSA-N | C1=CC(=C(C(=C1)Cl)N=C=S)Cl | 9999 | 1255 | 1 | 1 |
| 2,4-Dichlorophenyl isothiocyanate (C7H3Cl2NS) | WVBNZZHGECFCSH-UHFFFAOYSA-N | C1=CC(=C(C=C1Cl)Cl)N=C=S | 9999 | 1254 | 1 | 1 |
| Benzene, 1,2-dichloro-3-nitro- (C6H3Cl2NO2) | CMVQZRLQEOAYSW-UHFFFAOYSA-N | C1=CC(=C(C(=C1)Cl)Cl)[N+](=O)[O-] | 9420 | 46 | 1 | 2 |
| Benzene, 2,4-dichloro-1-nitro- (C6H3Cl2NO2) | QUIMTLZDMCNYGY-UHFFFAOYSA-N | C1=CC(=C(C=C1Cl)Cl)[N+](=O)[O-] | 9199 | 0 | 1 | 2 |
| 3,5-Dichloro-1-nitrobenzene (C6H3Cl2NO2) | RNABGKOKSBUFHW-UHFFFAOYSA-N | C1=C(C=C(C=C1Cl)Cl)[N+](=O)[O-] | 4854 | 50 | 1 | 2 |
| Benzene, 1,4-dichloro-2-nitro- (C6H3Cl2NO2) | RZKKOBGFCAHLCZ-UHFFFAOYSA-N | C1=CC(=C(C=C1Cl)[N+](=O)[O-])Cl | 6519 | 80 | 1 | 2 |
| Benzene, 1,2-dichloro-4-nitro- (C6H3Cl2NO2) | NTBYINQTYWZXLH-UHFFFAOYSA-N | C1=CC(=C(C=C1[N+](=O)[O-])Cl)Cl | 7379 | 10 | 1 | 2 |
| 3,4-Dichlorphenylisocyanate (C7H3Cl2NO) | MFUVCHZWGSJKEQ-UHFFFAOYSA-N | C1=CC(=C(C=C1N=C=O)Cl)Cl | 9999 | 13 | 1 | 2 |
| 3,5-Dichlorophenyl isocyanate (C7H3Cl2NO) | XEFUJGURFLOFAN-UHFFFAOYSA-N | C1=C(C=C(C=C1Cl)Cl)N=C=O | 9999 | 0 | 1 | 2 |
| Benzene, 1,4-dichloro-2-isocyanato- (C7H3Cl2NO) | PEQMJVGRHNZPAM-UHFFFAOYSA-N | C1=CC(=C(C=C1Cl)N=C=O)Cl | 9999 | 0 | 1 | 2 |
| 4-Chlorophenyl chloroformate (C7H4Cl2O2) | RYWGPCLTVXMMHO-UHFFFAOYSA-N | C1=CC(=CC=C1OC(=O)Cl)Cl | 7508 | 1288 | 1 | 1 |
| Phenol, 2,5-dichloro-4-methoxy- (C7H6Cl2O2) | JXKFAPUUGWQXHX-UHFFFAOYSA-N | COC1=C(C=C(C(=C1)Cl)O)Cl | 5670 | 1 | 1 | 2 |
| 2-Methoxy-3,6-dichloro-phenol (C7H6Cl2O2) | OBRQSFBOZCMSTK-UHFFFAOYSA-N | COC1=C(C=CC(=C1O)Cl)Cl | 6605 | 250 | 1 | 1 |
| (2,4,6-Trichlorophenyl)hydrazine (C6H5Cl3N2) | MULHANRBCQBHII-UHFFFAOYSA-N | C1=C(C=C(C(=C1Cl)NN)Cl)Cl | 8359 | 179 | 1 | 1 |
| 2,3,6-Trichloroanisole (C7H5Cl3O) | OTFNCXLUCRUNCH-UHFFFAOYSA-N | COC1=C(C=CC(=C1Cl)Cl)Cl | 9919 | 300 | 1 | 1 |
| 2,3,5-Trichloroanisole (C7H5Cl3O) | VGGPULNMTUAGOK-UHFFFAOYSA-N | COC1=CC(=CC(=C1Cl)Cl)Cl | 9999 | 600 | 1 | 1 |
| Benzene, 1,2,4-trichloro-5-methoxy- (C7H5Cl3O) | SXKBHOQOOGRFJF-UHFFFAOYSA-N | COC1=CC(=C(C=C1Cl)Cl)Cl | 9999 | 240 | 1 | 1 |
| 2,4,6-Trichloroanisole (C7H5Cl3O) | WCVOGSZTONGSQY-UHFFFAOYSA-N | COC1=C(C=C(C=C1Cl)Cl)Cl | 6776 | 130 | 1 | 1 |
| Benzenamine, 2,3,5,6-tetrachloro- (C6H3Cl4N) | YTDHEFNWWHSXSU-UHFFFAOYSA-N | C1=C(C(=C(C(=C1Cl)Cl)N)Cl)Cl | 7859 | 750 | 1 | 1 |
| Benzenamine, 2,3,4,5-tetrachloro- (C6H3Cl4N) | GBKZRUCVLTWAML-UHFFFAOYSA-N | C1=C(C(=C(C(=C1Cl)Cl)Cl)Cl)N | 7839 | 669 | 1 | 1 |
| Phenol, 2,3,4,5-tetrachloro- (C6H2Cl4O) | RULKYXXCCZZKDZ-UHFFFAOYSA-N | C1=C(C(=C(C(=C1Cl)Cl)Cl)Cl)O | 8109 | 209 | 1 | 1 |
| Phenol, 2,3,4,6-tetrachloro- (C6H2Cl4O) | VGVRPFIJEJYOFN-UHFFFAOYSA-N | C1=C(C(=C(C(=C1Cl)Cl)Cl)O)Cl | 7877 | 160 | 1 | 1 |
| Phenol, 2,3,5,6-tetrachloro- (C6H2Cl4O) | KEWNKZNZRIAIAK-UHFFFAOYSA-N | C1=C(C(=C(C(=C1Cl)Cl)O)Cl)Cl | 7789 | 149 | 1 | 1 |
| Pentachlorobenzene (C6HCl5) | CEOCDNVZRAIOQZ-UHFFFAOYSA-N | C1=C(C(=C(C(=C1Cl)Cl)Cl)Cl)Cl | 6779 | 1960 | 1 | 1 |
| Desisopropylatrazine (C5H8ClN5) | IVENSCMCQBJAKW-UHFFFAOYSA-N | CCNC1=NC(=NC(=N1)N)Cl | 5005 | 0 | 1 | 2 |
| 1,3,5-Triazin-2-amine, 4,6-dichloro-N-ethyl- (C5H6Cl2N4) | ACAHVXOSWOUZAB-UHFFFAOYSA-N | CCNC1=NC(=NC(=N1)Cl)Cl | 4804 | 0 | 1 | 2 |
| 2-Amino-5-chloro-4-pyrimidinecarboxylic acid (C5H4ClN3O2) | PDLZOTZLGNZOFS-UHFFFAOYSA-N | C1=C(C(=NC(=N1)N)C(=O)O)Cl | 0 | 0 | 2 | 2 |
| Methyl 6-chloronicotinate (C7H6ClNO2) | RMEDXVIWDFLGES-UHFFFAOYSA-N | COC(=O)C1=CN=C(C=C1)Cl | 2837 | 2498 | 1 | 1 |
| 4-Pyridinecarboxylic acid, 2,6-dichloro- (C6H3Cl2NO2) | SQSYNRCXIZHKAI-UHFFFAOYSA-N | C1=C(C=C(N=C1Cl)Cl)C(=O)O | 9999 | 1569 | 1 | 1 |
| Nitrapyrin (C6H3Cl4N) | DCUJJWWUNKIJPH-UHFFFAOYSA-N | C1=CC(=NC(=C1)Cl)C(Cl)(Cl)Cl | 360 | 9999 | 2 | 1 |
| Benzene, 1-chloro-2-(isothiocyanatomethyl)- (C8H6ClNS) | RMVDNJDSLXQPAV-UHFFFAOYSA-N | C1=CC=C(C(=C1)CN=C=S)Cl | 1795 | 22 | 1 | 2 |
| Benzene, 1-chloro-4-(isothiocyanatomethyl)- (C8H6ClNS) | DEHXIHUIYSXZNH-UHFFFAOYSA-N | C1=CC(=CC=C1CN=C=S)Cl | 1104 | 45 | 1 | 2 |
| Isothiocyanic acid, m-chlorobenzyl ester (C8H6ClNS) | GGNLZOLEJMSOKU-UHFFFAOYSA-N | C1=CC(=CC(=C1)Cl)CN=C=S | 2303 | 37 | 1 | 2 |
| Benzene, 1-(chloromethyl)-4-nitro- (C7H6ClNO2) | KGCNHWXDPDPSBV-UHFFFAOYSA-N | C1=CC(=CC=C1CCl)[N+](=O)[O-] | 4410 | 9999 | 1 | 1 |
| Benzene, 1-(chloromethyl)-2-nitro- (C7H6ClNO2) | BXCBUWKTXLWPSB-UHFFFAOYSA-N | C1=CC=C(C(=C1)CCl)[N+](=O)[O-] | 10 | 5369 | 2 | 1 |
| Benzene, 1-(chloromethyl)-3-nitro- (C7H6ClNO2) | APGGSERFJKEWFG-UHFFFAOYSA-N | C1=CC(=CC(=C1)[N+](=O)[O-])CCl | 2979 | 9999 | 1 | 1 |
| 2-Chlorophenoxyacetonitrile (C8H6ClNO) | JWRJGSQLKHGQOT-UHFFFAOYSA-N | C1=CC=C(C(=C1)OCC#N)Cl | 2963 | 311 | 1 | 1 |
| 2-(2-Chlorophenoxy)ethanol (C8H9ClO2) | FDQGMCQSIVZGHW-UHFFFAOYSA-N | C1=CC=C(C(=C1)OCCO)Cl | 1942 | 10 | 1 | 2 |
| Ethanol, 2-(4-chlorophenoxy)- (C8H9ClO2) | GEGSSUSEWOHAFE-UHFFFAOYSA-N | C1=CC(=CC=C1OCCO)Cl | 2709 | 20 | 1 | 2 |
| 3-Chlorobenzhydrazide (C7H7ClN2O) | PHRDZSRVSVNQRN-UHFFFAOYSA-N | C1=CC(=CC(=C1)Cl)C(=O)NN | 1571 | 0 | 1 | 2 |
| 4-Chlorobenzoic acid hydrazide (C7H7ClN2O) | PKBGHORNUFQAAW-UHFFFAOYSA-N | C1=CC(=CC=C1C(=O)NN)Cl | 1960 | 0 | 1 | 2 |
| 2-Chlorobenzoic acid hydrazide (C7H7ClN2O) | KPPNLSKVTKSSTG-UHFFFAOYSA-N | C1=CC=C(C(=C1)C(=O)NN)Cl | 1186 | 95 | 1 | 2 |
| 2-Amino-5-chlorobenzamide (C7H7ClN2O) | DNRVZOZGQHHDAT-UHFFFAOYSA-N | C1=CC(=C(C=C1Cl)C(=O)N)N | 5205 | 0 | 1 | 2 |
| Chloroformic acid, phenylmethyl ester (C8H7ClO2) | HSDAJNMJOMSNEV-UHFFFAOYSA-N | C1=CC=C(C=C1)COC(=O)Cl | 634 | 0 | 2 | 2 |
| Acetyl chloride, phenoxy- (C8H7ClO2) | PKUPAJQAJXVUEK-UHFFFAOYSA-N | C1=CC=C(C=C1)OCC(=O)Cl | 3569 | 0 | 1 | 2 |
| Acetic acid, chloro-, phenyl ester (C8H7ClO2) | AGUWUIVKDXDKBT-UHFFFAOYSA-N | C1=CC=C(C=C1)OC(=O)CCl | 881 | 0 | 1 | 2 |
| 2-Amino-6-chlorobenzoic acid (C7H6ClNO2) | SZCPTRGBOVXVCA-UHFFFAOYSA-N | C1=CC(=C(C(=C1)Cl)C(=O)O)N | 5471 | 0 | 1 | 2 |
| Benzoic acid, 4-amino-2-chloro- (C7H6ClNO2) | MBDUKNCPOPMRJQ-UHFFFAOYSA-N | C1=CC(=C(C=C1N)Cl)C(=O)O | 9099 | 60 | 1 | 2 |
| 3-Amino-4-chlorobenzoic acid (C7H6ClNO2) | DMGFVJVLVZOSOE-UHFFFAOYSA-N | C1=CC(=C(C=C1C(=O)O)N)Cl | 9999 | 100 | 1 | 2 |
| Benzoic acid, 2-amino-5-chloro- (C7H6ClNO2) | IFXKXCLVKQVVDI-UHFFFAOYSA-N | C1=CC(=C(C=C1Cl)C(=O)O)N | 5989 | 60 | 1 | 2 |
| Benzoic acid, 3-chloro-2-hydroxy (C7H5ClO3) | PPINMMULCRBDOS-UHFFFAOYSA-N | C1=CC(=C(C(=C1)Cl)O)C(=O)O | 4409 | 0 | 1 | 2 |
| Benzoic acid, 5-chloro-2-hydroxy (C7H5ClO3) | NKBASRXWGAGQDP-UHFFFAOYSA-N | C1=CC(=C(C=C1Cl)C(=O)O)O | 3653 | 0 | 1 | 2 |
| Benzoic acid, 4-chloro-3-hydroxy- (C7H5ClO3) | SCPUNJAMWFAYED-UHFFFAOYSA-N | C1=CC(=C(C=C1C(=O)O)O)Cl | 8482 | 0 | 1 | 2 |
| 3-Chloro-4-hydroxybenzoic acid (C7H5ClO3) | QGNLHMKIGMZKJX-UHFFFAOYSA-N | C1=CC(=C(C=C1C(=O)O)Cl)O | 5929 | 38 | 1 | 2 |
| Isothiocyanic acid, 5-chloro-o-tolyl ester (C8H6ClNS) | VDBDGAPNWNWUSA-UHFFFAOYSA-N | CC1=C(C=C(C=C1)Cl)N=C=S | 8518 | 9999 | 1 | 1 |
| Benzene, 2-chloro-4-isothiocyanato-1-methyl- (C8H6ClNS) | PQLHTYDGCDDPNU-UHFFFAOYSA-N | CC1=C(C=C(C=C1)N=C=S)Cl | 9999 | 8570 | 1 | 1 |
| Benzene, 4-chloro-1-isothiocyanato-2-methyl- (C8H6ClNS) | XTYLRVPBHHRTMS-UHFFFAOYSA-N | CC1=C(C=CC(=C1)Cl)N=C=S | 7985 | 9999 | 1 | 1 |
| 2-Chloro-4-methylphenyl isothiocyanate (C8H6ClNS) | UIZZXGXTVVWRED-UHFFFAOYSA-N | CC1=CC(=C(C=C1)N=C=S)Cl | 9999 | 8994 | 1 | 1 |
| 2-Chloro-4-nitrotoluene (C7H6ClNO2) | LLYXJBROWQDVMI-UHFFFAOYSA-N | CC1=C(C=C(C=C1)[N+](=O)[O-])Cl | 9999 | 60 | 1 | 2 |
| Benzene, 1-chloro-2-methyl-4-nitro- (C7H6ClNO2) | BGDCQZFFNFXYQC-UHFFFAOYSA-N | CC1=C(C=CC(=C1)[N+](=O)[O-])Cl | 9999 | 70 | 1 | 2 |
| 6-Chloro-2-nitrotoluene (C7H6ClNO2) | XCSNRORTQRKCHB-UHFFFAOYSA-N | CC1=C(C=CC=C1Cl)[N+](=O)[O-] | 2229 | 10 | 1 | 2 |
| Toluene, 4-chloro-2-nitro- (C7H6ClNO2) | SQFLFRQWPBEDHM-UHFFFAOYSA-N | CC1=C(C=C(C=C1)Cl)[N+](=O)[O-] | 3443 | 10 | 1 | 2 |
| 4-Chloro-3-nitrotoluene (C7H6ClNO2) | NWESJZZPAJGHRZ-UHFFFAOYSA-N | CC1=CC(=C(C=C1)Cl)[N+](=O)[O-] | 9469 | 149 | 1 | 1 |
| 4-Chloro-o-tolyl isocyanate (C8H6ClNO) | FTZJLXIATZSKIL-UHFFFAOYSA-N | CC1=C(C=CC(=C1)Cl)N=C=O | 9999 | 6854 | 1 | 1 |
| o-Chloroacetanilide (C8H8ClNO) | KNVQTRVKSOEHPU-UHFFFAOYSA-N | CC(=O)NC1=CC=CC=C1Cl | 1149 | 2669 | 1 | 1 |
| Acetamide, N-(4-chlorophenyl)- (C8H8ClNO) | GGUOCFNAWIODMF-UHFFFAOYSA-N | CC(=O)NC1=CC=C(C=C1)Cl | 2339 | 0 | 1 | 2 |
| Acetamide, N-(3-chlorophenyl)- (C8H8ClNO) | MUUQHCOAOLLHIL-UHFFFAOYSA-N | CC(=O)NC1=CC(=CC=C1)Cl | 2329 | 20 | 1 | 2 |
| anisoyl chloride (C8H7ClO2) | MXMOTZIXVICDSD-UHFFFAOYSA-N | COC1=CC=C(C=C1)C(=O)Cl | 540 | 9999 | 2 | 1 |
| Benzoyl chloride, 3-methoxy- (C8H7ClO2) | RUQIUASLAXJZIE-UHFFFAOYSA-N | COC1=CC=CC(=C1)C(=O)Cl | 2039 | 9999 | 1 | 1 |
| Benzoyl chloride, 2-methoxy- (C8H7ClO2) | RZNHSEZOLFEFGB-UHFFFAOYSA-N | COC1=CC=CC=C1C(=O)Cl | 529 | 9999 | 2 | 1 |
| Benzoic acid, 4-chloro-, methyl ester (C8H7ClO2) | LXNFVVDCCWUUKC-UHFFFAOYSA-N | COC(=O)C1=CC=C(C=C1)Cl | 3864 | 191 | 1 | 1 |
| Acetic acid, 3-chlorophenyl ester (C8H7ClO2) | GQTKYLQYHPTULY-UHFFFAOYSA-N | CC(=O)OC1=CC(=CC=C1)Cl | 2072 | 0 | 1 | 2 |
| Benzoic acid, 3-chloro-, methyl ester (C8H7ClO2) | XRDRKVPNHIWTBX-UHFFFAOYSA-N | COC(=O)C1=CC(=CC=C1)Cl | 5155 | 340 | 1 | 1 |
| Acetic acid, 2-chlorophenyl ester (C8H7ClO2) | CJPVPOYTTALCNX-UHFFFAOYSA-N | CC(=O)OC1=CC=CC=C1Cl | 839 | 0 | 1 | 2 |
| Benzoic acid, 2-chloro-, methyl ester (C8H7ClO2) | JAVRNIFMYIJXIE-UHFFFAOYSA-N | COC(=O)C1=CC=CC=C1Cl | 2793 | 40 | 1 | 2 |
| Acetic acid, 4-chlorophenyl ester (C8H7ClO2) | KEUPLGRNURQXAR-UHFFFAOYSA-N | CC(=O)OC1=CC=C(C=C1)Cl | 859 | 0 | 1 | 2 |
| 2,6-Dichlorobenzaldoxime (C7H5Cl2NO) | YBSXDWIAUZOFFV-UHFFFAOYSA-N | C1=CC(=C(C(=C1)Cl)C=NO)Cl | 9524 | 8591 | 1 | 1 |
| 3,4-Dichlorobenzaldoxime (C7H5Cl2NO) | ROBIUDOANJUDHD-UHFFFAOYSA-N | C1=CC(=C(C=C1C=NO)Cl)Cl | 8823 | 925 | 1 | 1 |
| chlorthiamid (C7H5Cl2NS) | KGKGSIUWJCAFPX-UHFFFAOYSA-N | C1=CC(=C(C(=C1)Cl)C(=S)N)Cl | 4190 | 9999 | 1 | 1 |
| Benzonitrile, 4-amino-3,5-dichloro- (C7H4Cl2N2) | COFNCCWGWXFACE-UHFFFAOYSA-N | C1=C(C=C(C(=C1Cl)N)Cl)C#N | 9999 | 935 | 1 | 1 |
| 3,5-Dichloro-4-hydroxybenzonitrile (C7H3Cl2NO) | YRSSHOVRSMQULE-UHFFFAOYSA-N | C1=C(C=C(C(=C1Cl)O)Cl)C#N | 9999 | 279 | 1 | 1 |
| Benzaldehyde, 2,4-dichloro- (C7H4Cl2O) | YSFBEAASFUWWHU-UHFFFAOYSA-N | C1=CC(=C(C=C1Cl)Cl)C=O | 6129 | 40 | 1 | 2 |
| Benzamide, 2,6-dichloro- (C7H5Cl2NO) | JHSPCUHPSIUQRB-UHFFFAOYSA-N | C1=CC(=C(C(=C1)Cl)C(=O)N)Cl | 3523 | 0 | 1 | 2 |
| 3,4-Dichlorobenzamide (C7H5Cl2NO) | XURBWYCGQQXTHJ-UHFFFAOYSA-N | C1=CC(=C(C=C1C(=O)N)Cl)Cl | 5505 | 0 | 1 | 2 |
| Benzoic acid, 2,6-dichloro- (C7H4Cl2O2) | MRUDNSFOFOQZDA-UHFFFAOYSA-N | C1=CC(=C(C(=C1)Cl)C(=O)O)Cl | 5262 | 230 | 1 | 1 |
| Benzoic acid, 2,5-dichloro- (C7H4Cl2O2) | QVTQYSFCFOGITD-UHFFFAOYSA-N | C1=CC(=C(C=C1Cl)C(=O)O)Cl | 9999 | 469 | 1 | 1 |
| Benzoic acid, 2,4-dichloro- (C7H4Cl2O2) | ATCRIUVQKHMXSH-UHFFFAOYSA-N | C1=CC(=C(C=C1Cl)Cl)C(=O)O | 6049 | 60 | 1 | 2 |
| 3,5-Dichlorobenzoic acid (C7H4Cl2O2) | CXKCZFDUOYMOOP-UHFFFAOYSA-N | C1=C(C=C(C=C1Cl)Cl)C(=O)O | 9999 | 302 | 1 | 1 |
| Benzoic acid, 3,4-dichloro- (C7H4Cl2O2) | VPHHJAOJUJHJKD-UHFFFAOYSA-N | C1=CC(=C(C=C1C(=O)O)Cl)Cl | 9999 | 60 | 1 | 2 |
| Benzaldehyde, 3,5-dichloro-2-hydroxy- (C7H4Cl2O2) | FABVMBDCVAJXMB-UHFFFAOYSA-N | C1=C(C=C(C(=C1Cl)O)C=O)Cl | 9999 | 70 | 1 | 2 |
| 1,3-dichloro-2-(methoxymethyl)benzene (C8H8Cl2O) | QBKBHXIQLAMKOB-UHFFFAOYSA-N | COCC1=C(C=CC=C1Cl)Cl | 1401 | 9999 | 1 | 1 |
| 2,6-Dichlorophenyl ethyl sulphide (C8H8Cl2S) | OCLSIUYYVUQKDI-UHFFFAOYSA-N | CCSC1=C(C=CC=C1Cl)Cl | 7205 | 87 | 1 | 2 |
| 2,4,6-Trichlorobenzonitrile (C7H2Cl3N) | PGODHCIOIPODFE-UHFFFAOYSA-N | C1=C(C=C(C(=C1Cl)C#N)Cl)Cl | 9999 | 1780 | 1 | 1 |
| 3,5-Dichlorobenzoyl chloride (C7H3Cl3O) | GGHLXLVPNZMBQR-UHFFFAOYSA-N | C1=C(C=C(C=C1Cl)Cl)C(=O)Cl | 1491 | 9999 | 1 | 1 |
| Benzoyl chloride, 3,4-dichloro- (C7H3Cl3O) | VTXNOVCTHUBABW-UHFFFAOYSA-N | C1=CC(=C(C=C1C(=O)Cl)Cl)Cl | 989 | 9999 | 1 | 1 |
| 2,6-Dichlorobenzoyl chloride (C7H3Cl3O) | JBLIDPPHFGWTKU-UHFFFAOYSA-N | C1=CC(=C(C(=C1)Cl)C(=O)Cl)Cl | 781 | 9999 | 2 | 1 |
| Benzoyl chloride, 2,4-dichloro- (C7H3Cl3O) | CEOCVKWBUWKBKA-UHFFFAOYSA-N | C1=CC(=C(C=C1Cl)Cl)C(=O)Cl | 269 | 9999 | 2 | 1 |
| 2,3,6-Trichlorobenzaldehyde (C7H3Cl3O) | AURSMWWOMOVHBM-UHFFFAOYSA-N | C1=CC(=C(C(=C1Cl)C=O)Cl)Cl | 5872 | 62 | 1 | 2 |
| o-Cresol, 3,4,6-trichloro- (C7H5Cl3O) | IAGVLEMVUDJCJS-UHFFFAOYSA-N | CC1=C(C(=CC(=C1Cl)Cl)Cl)O | 8678 | 9999 | 1 | 1 |
| α,α,α-2-Tetrachlorotoluene (C7H4Cl4) | MFHPYLFZSCSNST-UHFFFAOYSA-N | C1=CC=C(C(=C1)C(Cl)(Cl)Cl)Cl | 679 | 9999 | 2 | 1 |
| α,α,α-4-Tetrachlorotoluene (C7H4Cl4) | LVZPKYYPPLUECL-UHFFFAOYSA-N | C1=CC(=CC=C1C(Cl)(Cl)Cl)Cl | 430 | 9999 | 2 | 1 |
| Benzene, 2,6-dichloro-1-(dichloromethyl) (C7H4Cl4) | QQPXXHAEIGVZKQ-UHFFFAOYSA-N | C1=CC(=C(C(=C1)Cl)C(Cl)Cl)Cl | 1242 | 9999 | 1 | 1 |
| 2-Chloroethyl benzyl sulfide (C9H11ClS) | UQSMMTFBNJUPGW-UHFFFAOYSA-N | C1=CC=C(C=C1)CSCCCl | 1151 | 35 | 1 | 2 |
| 4-(Chloromethyl)benzoic acid (C8H7ClO2) | OITNBJHJJGMFBN-UHFFFAOYSA-N | C1=CC(=CC=C1CCl)C(=O)O | 3349 | 9999 | 1 | 1 |
| m-Chlorophenylacetic acid (C8H7ClO2) | WFPMUFXQDKMVCO-UHFFFAOYSA-N | C1=CC(=CC(=C1)Cl)CC(=O)O | 3929 | 30 | 1 | 2 |
| Benzeneacetic acid, 4-chloro- (C8H7ClO2) | CDPKJZJVTHSESZ-UHFFFAOYSA-N | C1=CC(=CC=C1CC(=O)O)Cl | 2703 | 16 | 1 | 2 |
| Benzaldehyde, 5-(chloromethyl)-2-hydroxy- (C8H7ClO2) | WFACWTZLXIFJCM-UHFFFAOYSA-N | C1=CC(=C(C=C1CCl)C=O)O | 1441 | 9999 | 1 | 1 |
| 4-Amino-3-chloro-5-methyl benzonitrile (C8H7ClN2) | NDTNVCCDQAOBSZ-UHFFFAOYSA-N | CC1=C(C(=CC(=C1)C#N)Cl)N | 8708 | 9999 | 1 | 1 |
| 2-Chloro-3-methylbenzoic acid (C8H7ClO2) | LWOKLXMNGXXORN-UHFFFAOYSA-N | CC1=CC=CC(=C1Cl)C(=O)O | 9999 | 5717 | 1 | 1 |
| p-Toluic acid, 3-chloro- (C8H7ClO2) | SDKUOEOJAXGCLU-UHFFFAOYSA-N | CC1=C(C=C(C=C1)C(=O)O)Cl | 9999 | 4867 | 1 | 1 |
| Ethanone, 1-(5-chloro-2-hydroxyphenyl)- (C8H7ClO2) | XTGCUDZCCIRWHL-UHFFFAOYSA-N | CC(=O)C1=C(C=CC(=C1)Cl)O | 5039 | 0 | 1 | 2 |
| 4-Methoxy-3-methylbenzyl chloride (C9H11ClO) | BHEHNICAPZVKRH-UHFFFAOYSA-N | CC1=C(C=CC(=C1)CCl)OC | 1572 | 9999 | 1 | 1 |
| Acetonitrile, (2,6-dichlorophenyl)- (C8H5Cl2N) | AOEJUUCUKRUCEF-UHFFFAOYSA-N | C1=CC(=C(C(=C1)Cl)CC#N)Cl | 6719 | 9999 | 1 | 1 |
| 3,4-Dichlorophenylacetonitrile (C8H5Cl2N) | QWZNCAFWRZZJMA-UHFFFAOYSA-N | C1=CC(=C(C=C1CC#N)Cl)Cl | 2532 | 9999 | 1 | 1 |
| Benzeneacetonitrile, 2,4-dichloro- (C8H5Cl2N) | VJARIBGMDPJLCL-UHFFFAOYSA-N | C1=CC(=C(C=C1Cl)Cl)CC#N | 4119 | 9999 | 1 | 1 |
| 2,4-Dichlorophenethylamine (C8H9Cl2N) | VHJKDOLGYMULOP-UHFFFAOYSA-N | C1=CC(=C(C=C1Cl)Cl)CCN | 0 | 187 | 2 | 1 |
| p-Chlorophenylacetyl chloride (C8H6Cl2O) | UMQUIRYNOVNYPA-UHFFFAOYSA-N | C1=CC(=CC=C1CC(=O)Cl)Cl | 686 | 539 | 2 | 1 |
| α-Chlorophenylacetyl chloride (C8H6Cl2O) | FGEAOSXMQZWHIQ-UHFFFAOYSA-N | C1=CC=C(C=C1)C(C(=O)Cl)Cl | 520 | 10 | 2 | 2 |
| 2,2-Dichloroacetophenone (C8H6Cl2O) | CERJZAHSUZVMCH-UHFFFAOYSA-N | C1=CC=C(C=C1)C(=O)C(Cl)Cl | 0 | 0 | 2 | 2 |
| Benzenemethanol, 3,4-dichloro-α-methyl- (C8H8Cl2O) | VZTGSONNNMGQNQ-UHFFFAOYSA-N | CC(C1=CC(=C(C=C1)Cl)Cl)O | 3733 | 2292 | 1 | 1 |
| 2,5-Dichloro-α-methylbenzyl alcohol (C8H8Cl2O) | RDMKUSDLLGKMCK-UHFFFAOYSA-N | CC(C1=C(C=CC(=C1)Cl)Cl)O | 3033 | 480 | 1 | 1 |
| Benzenemethanol, 2,6-dichloro-α-methyl- (C8H8Cl2O) | VUSOJMQVQGKPNN-UHFFFAOYSA-N | CC(C1=C(C=CC=C1Cl)Cl)O | 811 | 30 | 1 | 2 |
| 2,6-Dichloroacetophenone (C8H6Cl2O) | HYBDSXBLGCQKRE-UHFFFAOYSA-N | CC(=O)C1=C(C=CC=C1Cl)Cl | 1550 | 0 | 1 | 2 |
| Ethanone, 1-(2,4-dichlorophenyl)- (C8H6Cl2O) | XMCRWEBERCXJCH-UHFFFAOYSA-N | CC(=O)C1=C(C=C(C=C1)Cl)Cl | 1609 | 0 | 1 | 2 |
| Ethanone, 1-(2,5-dichlorophenyl)- (C8H6Cl2O) | CYNFEPKQDJHIMV-UHFFFAOYSA-N | CC(=O)C1=C(C=CC(=C1)Cl)Cl | 2798 | 9 | 1 | 2 |
| Ethanone, 1-(3,4-dichlorophenyl)- (C8H6Cl2O) | WBPAOUHWPONFEQ-UHFFFAOYSA-N | CC(=O)C1=CC(=C(C=C1)Cl)Cl | 3603 | 0 | 1 | 2 |
| Benzene, (2,2,2-trichloroethyl)- (C8H7Cl3) | XFEKIQFBJSDMQB-UHFFFAOYSA-N | C1=CC=C(C=C1)CC(Cl)(Cl)Cl | 611 | 486 | 2 | 1 |
| Dichloroacetic acid, morpholide (C6H9Cl2NO2) | SPDHGKQMFGYCHN-UHFFFAOYSA-N | C1COCCN1C(=O)C(Cl)Cl | 1231 | 851 | 1 | 1 |
| Propanenitrile, 3-(2-chlorophenyl) (C9H8ClN) | MMTXIUJWFHJGBA-UHFFFAOYSA-N | C1=CC=C(C(=C1)CCC#N)Cl | 1951 | 61 | 1 | 2 |
| Benzenepropanoyl chloride (C9H9ClO) | MFEILWXBDBCWKF-UHFFFAOYSA-N | C1=CC=C(C=C1)CCC(=O)Cl | 2619 | 1729 | 1 | 1 |
| 1-Phenyl-3-chloro-1-propanone (C9H9ClO) | KTJRGPZVSKWRTJ-UHFFFAOYSA-N | C1=CC=C(C=C1)C(=O)CCCl | 249 | 479 | 2 | 1 |
| p-(Chlorophenyl)acetone (C9H9ClO) | WEJRYKSUUFKMBC-UHFFFAOYSA-N | CC(=O)CC1=CC=C(C=C1)Cl | 573 | 0 | 2 | 2 |
| 1-(2-Chlorophenyl)propan-2-one (C9H9ClO) | LWGNDIMNCPMZOF-UHFFFAOYSA-N | CC(=O)CC1=CC=CC=C1Cl | 244 | 1450 | 2 | 1 |
| 4-Ethylbenzoyl chloride (C9H9ClO) | AVTLLLZVYYPGFX-UHFFFAOYSA-N | CCC1=CC=C(C=C1)C(=O)Cl | 414 | 9999 | 2 | 1 |
| 1-Propanone, 1-(4-chlorophenyl)- (C9H9ClO) | ADCYRBXQAJXJTD-UHFFFAOYSA-N | CCC(=O)C1=CC=C(C=C1)Cl | 773 | 51 | 2 | 2 |
| Benzene, 1,3-bis-(chloromethyl)-4-methyl (C9H10Cl2) | HKCWYAWNOIYUAS-UHFFFAOYSA-N | CC1=C(C=C(C=C1)CCl)CCl | 2519 | 9999 | 1 | 1 |
| Benzene, 1-(1-chloroethyl)-4-(chloromethyl)- (C9H10Cl2) | OPVAKHQEJWPSQB-UHFFFAOYSA-N | CC(C1=CC=C(C=C1)CCl)Cl | 1571 | 9999 | 1 | 1 |
| 3-Chloropropionic acid, morpholide (C7H12ClNO2) | RHPBOUGBOGWTDW-UHFFFAOYSA-N | C1COCCN1C(=O)CCCl | 5475 | 9999 | 1 | 1 |
| 2-Chloropropanoic acid, morpholide (C7H12ClNO2) | QAGATHCAVJHHGR-UHFFFAOYSA-N | CC(C(=O)N1CCOCC1)Cl | 1772 | 3253 | 1 | 1 |
| 2-chloro-3-isobutylpyrazine (C8H11ClN2) | ZWVNFQUBQJXJOP-UHFFFAOYSA-N | CC(C)CC1=NC=CN=C1Cl | 40 | 160 | 2 | 1 |
| Benzene, (4-chlorobutyl)- (C10H13Cl) | FLLZCZIHURYEQP-UHFFFAOYSA-N | C1=CC=C(C=C1)CCCCCl | 1108 | 168 | 1 | 1 |
| 1-Chloro-2-methyl-2-phenylpropane (C10H13Cl) | DNXXUUPUQXSUFH-UHFFFAOYSA-N | CC(C)(CCl)C1=CC=CC=C1 | 530 | 10 | 2 | 2 |
| Benzene, 1-chloromethyl-4-(1-methylethyl) (C10H13Cl) | CYAKWEQUWJAHLW-UHFFFAOYSA-N | CC(C)C1=CC=C(C=C1)CCl | 4184 | 5885 | 1 | 1 |
| 1-Chloro-4-(1,1-dimethylethyl)benzene (C10H13Cl) | XRTANKYQJQXSFP-UHFFFAOYSA-N | CC(C)(C)C1=CC=C(C=C1)Cl | 1781 | 0 | 1 | 2 |
| p-Cymene, 2-chloro- (C10H13Cl) | JVIGKRUGGYKFSL-UHFFFAOYSA-N | CC1=C(C=C(C=C1)C(C)C)Cl | 2973 | 1021 | 1 | 1 |
| Acetamide, N-tetrahydrofurfuryl-2-chloro- (C7H12ClNO2) | OINOJHHVQJESIB-UHFFFAOYSA-N | C1CC(OC1)CNC(=O)CCl | 0 | 90 | 2 | 2 |
| 2,2,6,6-Tetrachlorocyclohexanol (C6H8Cl4O) | RORBTKDJFQCFMD-UHFFFAOYSA-N | C1CC(C(C(C1)(Cl)Cl)O)(Cl)Cl | 70 | 901 | 2 | 1 |
| 2-Chlorocyclooctanone oxime (C8H14ClNO) | CLUUAPBIIZAOJL-UHFFFAOYSA-N | C1CCCC(=NO)C(CC1)Cl | 2302 | 4704 | 1 | 1 |
| Cyclopropane, 1,1-dichloro-2-methyl-2-(3-methylbutyl) (C9H16Cl2) | SZDCIZLZSRDCSY-UHFFFAOYSA-N | CC(C)CCC1(CC1(Cl)Cl)C | 50 | 0 | 2 | 2 |
| Cyclopropane, 1,1-dichloro-2,2,3-triethyl- (C9H16Cl2) | HZZCFNUFGQXPQC-UHFFFAOYSA-N | CCC1C(C1(Cl)Cl)(CC)CC | 300 | 1401 | 2 | 1 |
| Cyclohexane, 2-chloro-4-methyl-1-(1-methylethyl)-, [1S-(1α,2β,4β)]- (C10H19Cl) | OMLOJNNKKPNVKN-UHFFFAOYSA-N | CC1CCC(C(C1)Cl)C(C)C | 0 | 489 | 2 | 1 |
| 6-Chloro-2,4-dihydroxy-1,3-dimethylpyrimidine (C6H7ClN2O2) | VATQPUHLFQHDBD-UHFFFAOYSA-N | CN1C(=CC(=O)N(C1=O)C)Cl | 2592 | 20 | 1 | 2 |
| 5-Chloro-1,3-dimethyl-4-nitropyrazole (C5H6ClN3O2) | WBMBDRIOUHCVAS-UHFFFAOYSA-N | CC1=NN(C(=C1[N+](=O)[O-])Cl)C | 4090 | 0 | 1 | 2 |
| Phenol, 2-allyl-6-chloro-, (C9H9ClO) | UPEBXJHGPMUFKU-UHFFFAOYSA-N | C=CCC1=C(C(=CC=C1)Cl)O | 9597 | 9999 | 1 | 1 |
| α-Chlorocinnamaldehyde (C9H7ClO) | SARRRAKOHPKFBW-TWGQIWQCSA-N | C1=CC=C(C=C1)/C=C(/C=O)\Cl | 6656 | 1281 | 1 | 1 |
| Cyclobutanecarboxylic acid, 3-chloroprop-2-enyl ester (C8H11ClO2) | PWMURWCEFOHIHN-GORDUTHDSA-N | C1CC(C1)C(=O)OC/C=C/Cl | 50 | 3423 | 2 | 1 |
| 2,3-Dichlorothiophene-5-sulfonyl chloride (C4HCl3O2S2) | IVTWLTRKVRJPNG-UHFFFAOYSA-N | C1=C(SC(=C1Cl)Cl)S(=O)(=O)Cl | 3969 | 9999 | 1 | 1 |
| 2,5-Dichlorothiophene-3-sulfonyl chloride (C4HCl3O2S2) | JJKSHSHZJOWSEC-UHFFFAOYSA-N | C1=C(SC(=C1S(=O)(=O)Cl)Cl)Cl | 5145 | 9999 | 1 | 1 |
| 5-Nitro-2-furoyl chloride (C5H2ClNO4) | OLEFNFXYGGTROA-UHFFFAOYSA-N | C1=C(OC(=C1)[N+](=O)[O-])C(=O)Cl | 1442 | 9999 | 1 | 1 |
| Methyl 2,5-dichlorothiophene-3-carboxylate (C6H4Cl2O2S) | VCXPHMKCDRPIDG-UHFFFAOYSA-N | COC(=O)C1=C(SC(=C1)Cl)Cl | 3086 | 347 | 1 | 1 |
| Ethyl 5-chlorothiophene-2-carboxylate (C7H7ClO2S) | BMOKMXXTOZFEIZ-UHFFFAOYSA-N | CCOC(=O)C1=CC=C(S1)Cl | 1911 | 20 | 1 | 2 |
| 1,3-Cyclopentadiene, 1,2,3,4,5,5-hexachloro- (C5Cl6) | VUNCWTMEJYMOOR-UHFFFAOYSA-N | C1(=C(C(C(=C1Cl)Cl)(Cl)Cl)Cl)Cl | 919 | 6309 | 1 | 1 |
| 2,5-Cyclohexadien-1-one, 2,6-dichloro-4-(chloroimino)- (C6H2Cl3NO) | YHUMTHWQGWPJOQ-UHFFFAOYSA-N | C1=C(C(=O)C(=CC1=NCl)Cl)Cl | 9999 | 2419 | 1 | 1 |
| γ-Chloro-2-butyrothienone (C8H9ClOS) | NPFQPHILVMHTKP-UHFFFAOYSA-N | C1=CSC(=C1)C(=O)CCCCl | 510 | 50 | 2 | 2 |
| 4-Dichloromethyl-4-methyl-2,5-cyclohexadien-1-one (C8H8Cl2O) | MJRRCEHODULPAV-UHFFFAOYSA-N | CC1(C=CC(=O)C=C1)C(Cl)Cl | 704 | 3948 | 2 | 1 |
| 2-Amino-6-chlorobenzothiazole (C7H5ClN2S) | VMNXKIDUTPOHPO-UHFFFAOYSA-N | C1=CC2=C(C=C1Cl)SC(=N2)N | 9999 | 1039 | 1 | 1 |
| Zoxazolamine (C7H5ClN2O) | YGCODSQDUUUKIV-UHFFFAOYSA-N | C1=CC2=C(C=C1Cl)N=C(O2)N | 9999 | 50 | 1 | 2 |
| 2(3H)-Benzoxazolone, 6-chloro- (C7H4ClNO2) | MATCZHXABVLZIE-UHFFFAOYSA-N | C1=CC2=C(C=C1Cl)OC(=O)N2 | 5666 | 141 | 1 | 1 |
| Chloroxazone (C7H4ClNO2) | TZFWDZFKRBELIQ-UHFFFAOYSA-N | C1=CC2=C(C=C1Cl)NC(=O)O2 | 9999 | 20 | 1 | 2 |
| Benzothiazole, 2,6-dichloro- (C7H3Cl2NS) | QDZGJGWDGLHVNK-UHFFFAOYSA-N | C1=CC2=C(C=C1Cl)SC(=N2)Cl | 9999 | 1854 | 1 | 1 |
| 8-Chloroquinoline (C9H6ClN) | RUSMDMDNFUYZTM-UHFFFAOYSA-N | C1=CC2=C(C(=C1)Cl)N=CC=C2 | 9999 | 2589 | 1 | 1 |
| 4-Chloroquinoline (C9H6ClN) | KNDOFJFSHZCKGT-UHFFFAOYSA-N | C1=CC=C2C(=C1)C(=CC=N2)Cl | 9999 | 7216 | 1 | 1 |
| Quinoline, 6-chloro- (C9H6ClN) | GKJSZXGYFJBYRQ-UHFFFAOYSA-N | C1=CC2=C(C=CC(=C2)Cl)N=C1 | 9999 | 2829 | 1 | 1 |
| Quinoline, 2-chloro- (C9H6ClN) | OFUFXTHGZWIDDB-UHFFFAOYSA-N | C1=CC=C2C(=C1)C=CC(=N2)Cl | 8989 | 9999 | 1 | 1 |
| Quinoline, 5-chloro- (C9H6ClN) | HJSRGOVAIOPERP-UHFFFAOYSA-N | C1=CC2=C(C=CC=N2)C(=C1)Cl | 9999 | 3763 | 1 | 1 |
| 1-Chloronaphthalene (C10H7Cl) | JTPNRXUCIXHOKM-UHFFFAOYSA-N | C1=CC=C2C(=C1)C=CC=C2Cl | 9999 | 3279 | 1 | 1 |
| 2-Chloronaphthalene (C10H7Cl) | CGYGETOMCSJHJU-UHFFFAOYSA-N | C1=CC=C2C=C(C=CC2=C1)Cl | 9999 | 3279 | 1 | 1 |
| 1,3-Benzodioxole, 5-(chloromethyl)- (C8H7ClO2) | DWSUJONSJJTODA-UHFFFAOYSA-N | C1OC2=C(O1)C=C(C=C2)CCl | 2485 | 9999 | 1 | 1 |
| Benzothiazole, 5-chloro-2-methyl- (C8H6ClNS) | XCALAYIRFYALSX-UHFFFAOYSA-N | CC1=NC2=C(S1)C=CC(=C2)Cl | 9999 | 3863 | 1 | 1 |
| 5-Chloro-1-indanone (C9H7ClO) | MEDSHTHCZIOVPU-UHFFFAOYSA-N | C1CC(=O)C2=C1C=C(C=C2)Cl | 9999 | 1006 | 1 | 1 |
| Cyclopropane, 1,1-dichloro, 2-phenyl (C9H8Cl2) | WLWFQGXZIDYWQF-UHFFFAOYSA-N | C1C(C1(Cl)Cl)C2=CC=CC=C2 | 550 | 2332 | 2 | 1 |
| Bornyl chloride (C10H17Cl) | XXZAOMJCZBZKPV-UHFFFAOYSA-N | CC1(C2CCC1(C(C2)Cl)C)C | 0 | 709 | 2 | 1 |
| 5-Chloro-beznofurazan oxide (C6H3ClN2O2) | DHPQXIQZZCNOLI-UHFFFAOYSA-N | C1=CC2=[N+](ON=C2C=C1Cl)[O-] | 9999 | 0 | 1 | 2 |
| 1-Chloroadamantane (C10H15Cl) | OZNXTQSXSHODFR-UHFFFAOYSA-N | C1C2CC3CC1CC(C2)(C3)Cl | 200 | 9999 | 2 | 1 |
| Trichlorfon (C4H8Cl3O4P) | NFACJZMKEDPNKN-UHFFFAOYSA-N | COP(=O)(C(C(Cl)(Cl)Cl)O)OC | 0 | 1049 | 2 | 1 |
| Ethyleneglycol bischloro acetate (C6H8Cl2O4) | HIIBHBNRMVLLKH-UHFFFAOYSA-N | C(COC(=O)CCl)OC(=O)CCl | 0 | 0 | 2 | 2 |
| 2-Chloro-N,N-diethylacetoacetamide (C8H14ClNO2) | SEWWCLPPLUYJOT-UHFFFAOYSA-N | CCN(CC)C(=O)C(C(=O)C)Cl | 50 | 1176 | 2 | 1 |
| 1,4-Bis (2-Chloroethylthio)butane (C8H16Cl2S2) | AYSIRJGVBLMLAS-UHFFFAOYSA-N | C(CCSCCCl)CSCCCl | 130 | 90 | 2 | 2 |
| Succinic acid, monochloride, 2-chloropropyl ester (C7H10Cl2O3) | CBFQBJGKZBRWKX-UHFFFAOYSA-N | CC(COC(=O)CCC(=O)Cl)Cl | 0 | 30 | 2 | 2 |
| Pentyl trichloroacetate (C7H11Cl3O2) | LZJOVWVTJGLPFN-UHFFFAOYSA-N | CCCCCOC(=O)C(Cl)(Cl)Cl | 0 | 0 | 2 | 2 |
| Acetic acid, trichloro-, 3-methylbutyl ester (C7H11Cl3O2) | HBNLTLLITHPMDZ-UHFFFAOYSA-N | CC(C)CCOC(=O)C(Cl)(Cl)Cl | 0 | 40 | 2 | 2 |
| Acetic acid, trichloro, 1-methylbutyl ester (C7H11Cl3O2) | GKGUCQJRCNVBMU-UHFFFAOYSA-N | CCCC(C)OC(=O)C(Cl)(Cl)Cl | 0 | 210 | 2 | 1 |
| Dichloroacetic acid, hex-4-yn-3-yl ester (C8H10Cl2O2) | HNJCYURZVMLJAK-UHFFFAOYSA-N | CCC(C#CC)OC(=O)C(Cl)Cl | 0 | 4675 | 2 | 1 |
| 7-Chloroheptanoic acid, chloromethyl ester (C8H14Cl2O2) | MWONBSQQAQAREF-UHFFFAOYSA-N | C(CCCCl)CCC(=O)OCCl | 0 | 0 | 2 | 2 |
| 6-chlorohexyl chloroacetate (C8H14Cl2O2) | VNGMYNDYUJRSGC-UHFFFAOYSA-N | C(CCCCl)CCOC(=O)CCl | 0 | 0 | 2 | 2 |
| 6-Chloroheptanoic acid, chloromethyl ester (C8H14Cl2O2) | GKEBLUUVNFJTHF-UHFFFAOYSA-N | CC(CCCCC(=O)OCCl)Cl | 0 | 0 | 2 | 2 |
| 4-Chloroheptanoic acid, chloromethyl ester (C8H14Cl2O2) | ZEZMVWNYGQAVFR-UHFFFAOYSA-N | CCCC(CCC(=O)OCCl)Cl | 0 | 0 | 2 | 2 |
| Hexyl dichloroacetate (C8H14Cl2O2) | WZSTYEOGVNOGKU-UHFFFAOYSA-N | CCCCCCOC(=O)C(Cl)Cl | 0 | 0 | 2 | 2 |
| 2-Chloroheptanoic acid, chloromethyl ester (C8H14Cl2O2) | OEONOJHNBIZMIO-UHFFFAOYSA-N | CCCCCC(C(=O)OCCl)Cl | 0 | 0 | 2 | 2 |
| 3-Chloroheptanoic acid, chloromethyl ester (C8H14Cl2O2) | HJUPGERNLBGVLC-UHFFFAOYSA-N | CCCCC(CC(=O)OCCl)Cl | 0 | 990 | 2 | 1 |
| Propanoic acid, 2,2-dichloro, pentyl ester (C8H14Cl2O2) | ZCPOSLAFHCNHBE-UHFFFAOYSA-N | CCCCCOC(=O)C(C)(Cl)Cl | 0 | 0 | 2 | 2 |
| 5-chlorovaleric acid, but-3-yn-2-yl ester (C9H13ClO2) | DKDCXIDSBYMDQQ-UHFFFAOYSA-N | CC(C#C)OC(=O)CCCCCl | 70 | 60 | 2 | 2 |
| 3-Chloropropionic acid, hex-4-yn-3-yl ester (C9H13ClO2) | GEBZZBIUKLVCOU-UHFFFAOYSA-N | CCC(C#CC)OC(=O)CCCl | 511 | 120 | 2 | 1 |
| Heptyl chloroacetate (C9H17ClO2) | JUFPTDUONWPUBM-UHFFFAOYSA-N | CCCCCCCOC(=O)CCl | 17 | 79 | 2 | 2 |
| Octanoic acid, chloromethyl ester (C9H17ClO2) | JRCUJOMLYAQHDP-UHFFFAOYSA-N | CCCCCCCC(=O)OCCl | 50 | 0 | 2 | 2 |
| Propanoic acid, 3-chloro, hexyl ester (C9H17ClO2) | PYOAHWWMEFRKLM-UHFFFAOYSA-N | CCCCCCOC(=O)CCCl | 0 | 0 | 2 | 2 |
| 2-Chlorooctanoic acid, methyl ester (C9H17ClO2) | ABYDNTFEQUKFPQ-UHFFFAOYSA-N | CCCCCCC(C(=O)OC)Cl | 130 | 1211 | 2 | 1 |
| 10-Chloro-1-decanol (C10H21ClO) | OTUSESJECXGMIV-UHFFFAOYSA-N | C(CCCCCCl)CCCCO | 0 | 0 | 2 | 2 |
| Decanoyl chloride (C10H19ClO) | IPIVAXLHTVNRBS-UHFFFAOYSA-N | CCCCCCCCCC(=O)Cl | 0 | 4624 | 2 | 1 |
| 1,10-Dichlorodecane (C10H20Cl2) | RBBNTRDPSVZESY-UHFFFAOYSA-N | C(CCCCCCl)CCCCCl | 0 | 0 | 2 | 2 |
| 1-Chloroundecane (C11H23Cl) | ZHKKNUKCXPWZOP-UHFFFAOYSA-N | CCCCCCCCCCCCl | 20 | 0 | 2 | 2 |
| Acetoxyacetic acid, 3-chloroprop-2-enyl ester (C7H9ClO4) | MATWRJSMXKVOPA-NSCUHMNNSA-N | CC(=O)OCC(=O)OC/C=C/Cl | 0 | 330 | 2 | 1 |
| CDEC (C8H14ClNS2) | XJCLWVXTCRQIDI-UHFFFAOYSA-N | CCN(CC)C(=S)SCC(=C)Cl | 350 | 9999 | 2 | 1 |
| Trichloroacetic acid, pent-2-en-4-ynyl ester (C7H5Cl3O2) | GRCGKUPJKHXUAF-UHFFFAOYSA-N | C#CC=CCOC(=O)C(Cl)(Cl)Cl | 50 | 230 | 2 | 1 |
| Trichloroacetic acid, 3-methylbut-2-enyl ester (C7H9Cl3O2) | NYRNTCPRCWEVKU-UHFFFAOYSA-N | CC(=CCOC(=O)C(Cl)(Cl)Cl)C | 90 | 180 | 2 | 1 |
| 5-chlorovaleric acid, 3-chloroprop-2-enyl ester (C8H12Cl2O2) | JSKLDLBTXFSIRC-ZZXKWVIFSA-N | C(CCCl)CC(=O)OC/C=C/Cl | 60 | 1892 | 2 | 1 |
| Hexanoic acid, 3-chloroprop-2-enyl ester (C9H15ClO2) | SYXYWMBFAHQABF-FNORWQNLSA-N | CCCCCC(=O)OC/C=C/Cl | 110 | 2412 | 2 | 1 |
| 2-Chlorophenyl dichlorophosphate (C6H4Cl3O2P) | VLDPXPPHXDGHEW-UHFFFAOYSA-N | C1=CC=C(C(=C1)OP(=O)(Cl)Cl)Cl | 1799 | 9999 | 1 | 1 |
| 4-Chlorophenyl dichlorophosphate (C6H4Cl3O2P) | CCZMQYGSXWZFKI-UHFFFAOYSA-N | C1=CC(=CC=C1OP(=O)(Cl)Cl)Cl | 7026 | 1171 | 1 | 1 |
| Benzenamine, 2-chloro-4-(methylsulfonyl)- (C7H8ClNO2S) | VLMRGLCBIFWPGL-UHFFFAOYSA-N | CS(=O)(=O)C1=CC(=C(C=C1)N)Cl | 8008 | 0 | 1 | 2 |
| Benzene, 2-chloro-1-methoxy-4-nitro- (C7H6ClNO3) | DLJPNXLHWMRQIQ-UHFFFAOYSA-N | COC1=C(C=C(C=C1)[N+](=O)[O-])Cl | 9999 | 0 | 1 | 2 |
| Urea, N-(2-chlorophenyl)-N'-methyl- (C8H9ClN2O) | NFUCGJBURDOBPQ-UHFFFAOYSA-N | CNC(=O)NC1=CC=CC=C1Cl | 1500 | 1500 | 1 | 1 |
| Carbamic acid, 4-chlorophenyl, methyl ester (C8H8ClNO2) | CRGWHGISCDFEJY-UHFFFAOYSA-N | COC(=O)NC1=CC=C(C=C1)Cl | 9999 | 0 | 1 | 2 |
| Carbamic acid, 3-chlorophenyl, methyl ester (C8H8ClNO2) | SEPMCAVKHUPSMA-UHFFFAOYSA-N | COC(=O)NC1=CC(=CC=C1)Cl | 9999 | 30 | 1 | 2 |
| Formamidine, 3,3-dimethyl-1-(4-chlorophenyl) (C9H11ClN2) | ZPTXBCJETBDOAT-UHFFFAOYSA-N | CN(C)C=NC1=CC=C(C=C1)Cl | 9999 | 0 | 1 | 2 |
| N1N1-dimethyl-N2-ortho-chlorophenylformamidine (C9H11ClN2) | APKRGIKHQYEHGV-UHFFFAOYSA-N | CN(C)C=NC1=CC=CC=C1Cl | 8696 | 9999 | 1 | 1 |
| N'-(3-chloro-phenyl)-N,N-dimethyl-formamidine (C9H11ClN2) | BZBSXSKRUISGJM-UHFFFAOYSA-N | CN(C)C=NC1=CC(=CC=C1)Cl | 9999 | 90 | 1 | 2 |
| Benzenamine, 4-chloro-2,5-dimethoxy- (C8H10ClNO2) | YGUFQYGSBVXPMC-UHFFFAOYSA-N | COC1=CC(=C(C=C1N)OC)Cl | 4290 | 0 | 1 | 2 |
| Benzenesulfonyl chloride, 2,4-dichloro- (C6H3Cl3O2S) | FDTPBIKNYWQLAE-UHFFFAOYSA-N | C1=CC(=C(C=C1Cl)Cl)S(=O)(=O)Cl | 2572 | 9999 | 1 | 1 |
| 2,6-Dichlorobenzenesulfonyl chloride (C6H3Cl3O2S) | WGGKQIKICKLWGN-UHFFFAOYSA-N | C1=CC(=C(C(=C1)Cl)S(=O)(=O)Cl)Cl | 2751 | 9469 | 1 | 1 |
| 3,5-Dichlorobenzenesulfonyl chloride (C6H3Cl3O2S) | RJSQINMKOSOUGT-UHFFFAOYSA-N | C1=C(C=C(C=C1Cl)Cl)S(=O)(=O)Cl | 1861 | 3679 | 1 | 1 |
| Benzenesulfonyl chloride, 3,4-dichloro- (C6H3Cl3O2S) | NYIBPWGZGSXURD-UHFFFAOYSA-N | C1=CC(=C(C=C1S(=O)(=O)Cl)Cl)Cl | 1820 | 6550 | 1 | 1 |
| 2,4-Dichloro-6-nitroaniline (C6H4Cl2N2O2) | IZEZAMILKKYOPW-UHFFFAOYSA-N | C1=C(C=C(C(=C1Cl)N)[N+](=O)[O-])Cl | 9999 | 0 | 1 | 2 |
| 4,5-Dichloro-2-nitroaniline (C6H4Cl2N2O2) | FSGTULQLEVAYRS-UHFFFAOYSA-N | C1=C(C(=CC(=C1Cl)Cl)[N+](=O)[O-])N | 9999 | 0 | 1 | 2 |
| Dichloran (C6H4Cl2N2O2) | BIXZHMJUSMUDOQ-UHFFFAOYSA-N | C1=C(C=C(C(=C1Cl)N)Cl)[N+](=O)[O-] | 9999 | 53 | 1 | 2 |
| Phenol, 2,4-dichloro-6-nitro- (C6H3Cl2NO3) | LYPMXMBQPXPNIQ-UHFFFAOYSA-N | C1=C(C=C(C(=C1Cl)O)[N+](=O)[O-])Cl | 9999 | 0 | 1 | 2 |
| Chloroneb (C8H8Cl2O2) | PFIADAMVCJPXSF-UHFFFAOYSA-N | COC1=CC(=C(C=C1Cl)OC)Cl | 5298 | 19 | 1 | 2 |
| Benzene, 4,5-dichloro-1,2-dimethoxy (C8H8Cl2O2) | RJYXLZQZBLGBOM-UHFFFAOYSA-N | COC1=CC(=C(C=C1OC)Cl)Cl | 9999 | 0 | 1 | 2 |
| Benzene, 3,4-dichloro-1,2-dimethoxy (C8H8Cl2O2) | WEMHPBMPYVGSJI-UHFFFAOYSA-N | COC1=C(C(=C(C=C1)Cl)Cl)OC | 9999 | 0 | 1 | 2 |
| Benzene, 3,5-dichloro-1,2-dimethoxy (C8H8Cl2O2) | BCWABYVHGXOWHB-UHFFFAOYSA-N | COC1=CC(=CC(=C1OC)Cl)Cl | 8617 | 0 | 1 | 2 |
| Benzene, 3,6-dichloro-1,2-dimethoxy (C8H8Cl2O2) | CBYMFVMZRAOHOY-UHFFFAOYSA-N | COC1=C(C=CC(=C1OC)Cl)Cl | 9999 | 0 | 1 | 2 |
| 2,3,4-Trichlorophenyl isothiocyanate (C7H2Cl3NS) | NUAGIDXAYVLNEG-UHFFFAOYSA-N | C1=CC(=C(C(=C1N=C=S)Cl)Cl)Cl | 9652 | 992 | 1 | 1 |
| Benzene, 1,3,5-trichloro-2-isothiocyanato- (C7H2Cl3NS) | DSXVZIOQROMOEF-UHFFFAOYSA-N | C1=C(C=C(C(=C1Cl)N=C=S)Cl)Cl | 9845 | 825 | 1 | 1 |
| Benzene, 1,2,4-trichloro-5-isothiocyanato- (C7H2Cl3NS) | PJLRSYLEFZNICX-UHFFFAOYSA-N | C1=C(C(=CC(=C1Cl)Cl)Cl)N=C=S | 9499 | 1087 | 1 | 1 |
| 1,2,3-Trichloro-4-nitrobenzene (C6H2Cl3NO2) | BGKIECJVXXHLDP-UHFFFAOYSA-N | C1=CC(=C(C(=C1[N+](=O)[O-])Cl)Cl)Cl | 9999 | 0 | 1 | 2 |
| Benzene, 1,3,5-trichloro-2-nitro- (C6H2Cl3NO2) | AEBJDOTVYMITIA-UHFFFAOYSA-N | C1=C(C=C(C(=C1Cl)[N+](=O)[O-])Cl)Cl | 9159 | 0 | 1 | 2 |
| Benzene, 1,2,3-trichloro-5-nitro- (C6H2Cl3NO2) | HHLCSFGOTLUREE-UHFFFAOYSA-N | C1=C(C=C(C(=C1Cl)Cl)Cl)[N+](=O)[O-] | 9209 | 20 | 1 | 2 |
| Benzene, 1,2,4-trichloro-5-nitro- (C6H2Cl3NO2) | IBRBMZRLVYKVRF-UHFFFAOYSA-N | C1=C(C(=CC(=C1Cl)Cl)Cl)[N+](=O)[O-] | 9999 | 20 | 1 | 2 |
| Hydroquinone, tetrachloro- (C6H2Cl4O2) | STOSPPMGXZPHKP-UHFFFAOYSA-N | C1(=C(C(=C(C(=C1Cl)Cl)O)Cl)Cl)O | 8000 | 420 | 1 | 1 |
| 2,3,5,6-Tetrachloroanisole (C7H4Cl4O) | WMMFIDNWZNCBCT-UHFFFAOYSA-N | COC1=C(C(=CC(=C1Cl)Cl)Cl)Cl | 8300 | 500 | 1 | 1 |
| Benzene, 1,2,3,5-tetrachloro-4-methoxy- (C7H4Cl4O) | ITXDBGLYYSJNPK-UHFFFAOYSA-N | COC1=C(C(=C(C=C1Cl)Cl)Cl)Cl | 6306 | 200 | 1 | 1 |
| Benzenethiol, pentachloro- (C6HCl5S) | LLMLGZUZTFMXSA-UHFFFAOYSA-N | C1(=C(C(=C(C(=C1Cl)Cl)Cl)Cl)Cl)S | 6259 | 4739 | 1 | 1 |
| HCB (C6Cl6) | CKAPSXZOOQJIBF-UHFFFAOYSA-N | C1(=C(C(=C(C(=C1Cl)Cl)Cl)Cl)Cl)Cl | 5339 | 1800 | 1 | 1 |
| Isonicotinic acid, 2-amino-6-chloro-, hydrazide (C6H7ClN4O) | OAOMXEJFYBPNEX-UHFFFAOYSA-N | C1=C(C=C(N=C1N)Cl)C(=O)NN | 5691 | 81 | 1 | 2 |
| Clopyralid acid, methyl ester (C7H5Cl2NO2) | HQTUEAOWLVWJLF-UHFFFAOYSA-N | COC(=O)C1=C(C=CC(=N1)Cl)Cl | 1756 | 0 | 1 | 2 |
| Benzonitrile, 5-chloro-2-nitro- (C7H3ClN2O2) | HPWJUEZFOUOUEO-UHFFFAOYSA-N | C1=CC(=C(C=C1Cl)C#N)[N+](=O)[O-] | 9889 | 0 | 1 | 2 |
| 2-(4-Chlorophenoxy)thioacetamide (C8H8ClNOS) | AIOQDHOVIDONNW-UHFFFAOYSA-N | C1=CC(=CC=C1OCC(=S)N)Cl | 1511 | 0 | 1 | 2 |
| 2-(2-Chlorophenoxy)thioacetamide (C8H8ClNOS) | YVAJKXSZBMTZMX-UHFFFAOYSA-N | C1=CC=C(C(=C1)OCC(=S)N)Cl | 25 | 4509 | 2 | 1 |
| 2-(3-Chlorophenoxy)thioacetamide (C8H8ClNOS) | RPAOLVIADVQKNA-UHFFFAOYSA-N | C1=CC(=CC(=C1)Cl)OCC(=S)N | 847 | 0 | 1 | 2 |
| 4-Chlorobenzoyl isothiocyanate (C8H4ClNOS) | OTZBZZNWOAIAEN-UHFFFAOYSA-N | C1=CC(=CC=C1C(=O)N=C=S)Cl | 70 | 0 | 2 | 2 |
| 2-Chlorobenzoyl isothiocyanate (C8H4ClNOS) | XYPZDEZBMUGRRY-UHFFFAOYSA-N | C1=CC=C(C(=C1)C(=O)N=C=S)Cl | 30 | 0 | 2 | 2 |
| 3-Chlorobenzoyl isothiocyanate (C8H4ClNOS) | ZUISLKTWVWIULT-UHFFFAOYSA-N | C1=CC(=CC(=C1)Cl)C(=O)N=C=S | 170 | 0 | 2 | 2 |
| 4-Nitrobenzoyl chloride (C7H4ClNO3) | SKDHHIUENRGTHK-UHFFFAOYSA-N | C1=CC(=CC=C1C(=O)Cl)[N+](=O)[O-] | 90 | 9999 | 2 | 1 |
| o-Nitrobenzoyl chloride (C7H4ClNO3) | BWWHTIHDQBHTHP-UHFFFAOYSA-N | C1=CC=C(C(=C1)C(=O)Cl)[N+](=O)[O-] | 0 | 9999 | 2 | 1 |
| 4-Chloro-3-nitrobenzaldehyde (C7H4ClNO3) | HETBKLHJEWXWBM-UHFFFAOYSA-N | C1=CC(=C(C=C1C=O)[N+](=O)[O-])Cl | 8731 | 0 | 1 | 2 |
| Acetic acid, (4-chlorophenoxy)- (C8H7ClO3) | SODPIMGUZLOIPE-UHFFFAOYSA-N | C1=CC(=CC=C1OCC(=O)O)Cl | 9999 | 30 | 1 | 2 |
| o-Chlorophenoxyacetic acid (C8H7ClO3) | OPQYFNRLWBWCST-UHFFFAOYSA-N | C1=CC=C(C(=C1)OCC(=O)O)Cl | 8129 | 2519 | 1 | 1 |
| 3-Chloro-2-Methylbenzenesulphonyl chloride (C7H6Cl2O2S) | ZSIYKAQPQRTBPF-UHFFFAOYSA-N | CC1=C(C=CC=C1Cl)S(=O)(=O)Cl | 3093 | 3801 | 1 | 1 |
| p-Methylsulfonylbenzyl chloride (C8H9ClO2S) | LXPRVXKHIXWBJZ-UHFFFAOYSA-N | CS(=O)(=O)C1=CC=C(C=C1)CCl | 3552 | 1898 | 1 | 1 |
| Benzoic acid, 2-amino-5-chloro-, methyl ester (C8H8ClNO2) | IGHVUURTQGBABT-UHFFFAOYSA-N | COC(=O)C1=C(C=CC(=C1)Cl)N | 5945 | 74 | 1 | 2 |
| 4-Chloro-ortho-anisic acid (C8H7ClO3) | UFEYMXHWIHFRBX-UHFFFAOYSA-N | COC1=C(C=CC(=C1)Cl)C(=O)O | 5617 | 206 | 1 | 1 |
| 2,4-Dichlorobenzyl isothiocyanate (C8H5Cl2NS) | JZHPXEMPVDMDFB-UHFFFAOYSA-N | C1=CC(=C(C=C1Cl)Cl)CN=C=S | 1190 | 36 | 1 | 2 |
| 3,5-Dichlorophenoxyacetonitrile (C8H5Cl2NO) | PNROREDTZJCOHF-UHFFFAOYSA-N | C1=C(C=C(C=C1Cl)Cl)OCC#N | 6360 | 7011 | 1 | 1 |
| 2,4-Dichlorophenoxyacetonitrile (C8H5Cl2NO) | QSDMPLNZSJLFRM-UHFFFAOYSA-N | C1=CC(=C(C=C1Cl)Cl)OCC#N | 3974 | 100 | 1 | 2 |
| Ethanol, 2-(2,4-dichlorophenoxy)- (C8H8Cl2O2) | PCCMNBRZMKANQD-UHFFFAOYSA-N | C1=CC(=C(C=C1Cl)Cl)OCCO | 1541 | 0 | 1 | 2 |
| 2,4-Dichlorobenzhydrazide (C7H6Cl2N2O) | QOJQHOGSXXSMKX-UHFFFAOYSA-N | C1=CC(=C(C=C1Cl)Cl)C(=O)NN | 1001 | 0 | 1 | 2 |
| 2,5-Dichlorobenzhydrazide (C7H6Cl2N2O) | XECBCXNWGBDIMG-UHFFFAOYSA-N | C1=CC(=C(C=C1Cl)C(=O)NN)Cl | 1191 | 568 | 1 | 1 |
| Acetamide, N-(3-chlorophenyl)-2-chloro- (C8H7Cl2NO) | KNVBYGNINQITJC-UHFFFAOYSA-N | C1=CC(=CC(=C1)Cl)NC(=O)CCl | 4714 | 180 | 1 | 1 |
| Chloroacetic acid, 4-chlorophenyl ester (C8H6Cl2O2) | GEQGYTKAVRDKNR-UHFFFAOYSA-N | C1=CC(=CC=C1OC(=O)CCl)Cl | 711 | 0 | 2 | 2 |
| Benzoic acid, 3-amino-2,5-dichloro- (C7H5Cl2NO2) | HSSBORCLYSCBJR-UHFFFAOYSA-N | C1=C(C=C(C(=C1N)Cl)C(=O)O)Cl | 9999 | 320 | 1 | 1 |
| 1(4H)-Pyridineacetic acid, 3,5-dichloro-4-oxo- (C7H5Cl2NO3) | UHXYYTSWBYTDPD-UHFFFAOYSA-N | C1=C(C=C(C(=C1Cl)N)Cl)C(=O)O | 9999 | 0 | 1 | 2 |
| Benzoic acid, 3,5-dichloro-2-hydroxy (C7H4Cl2O3) | CNJGWCQEGROXEE-UHFFFAOYSA-N | C1=C(C=C(C(=C1Cl)O)C(=O)O)Cl | 3229 | 10 | 1 | 2 |
| 3,5-Dichloro-4-hydroxybenzoic acid (C7H4Cl2O3) | AULKDLUOQCUNOK-UHFFFAOYSA-N | C1=C(C=C(C(=C1Cl)O)Cl)C(=O)O | 9999 | 30 | 1 | 2 |
| 2,6-Dichloro-3-nitrotoluene (C7H5Cl2NO2) | WBNZUUIFTPNYRN-UHFFFAOYSA-N | CC1=C(C=CC(=C1Cl)[N+](=O)[O-])Cl | 7617 | 0 | 1 | 2 |
| Acetamide, N-(2,6-dichlorophenyl)- (C8H7Cl2NO) | DWVWVSLAIJHBBG-UHFFFAOYSA-N | CC(=O)NC1=C(C=CC=C1Cl)Cl | 40 | 3093 | 2 | 1 |
| 3',4'-Dichloroacetanilide (C8H7Cl2NO) | SCYGGCAQZFJGRF-UHFFFAOYSA-N | CC(=O)NC1=CC(=C(C=C1)Cl)Cl | 2449 | 0 | 1 | 2 |
| Acetamide, N-(2,4-dichlorophenyl)- (C8H7Cl2NO) | GZSGTFDLLISMMA-UHFFFAOYSA-N | CC(=O)NC1=C(C=C(C=C1)Cl)Cl | 1879 | 979 | 1 | 1 |
| Phenol, 3,4-dichloro-, acetate (C8H6Cl2O2) | OSKGYRIYNMZFSJ-UHFFFAOYSA-N | CC(=O)OC1=CC(=C(C=C1)Cl)Cl | 861 | 0 | 1 | 2 |
| Phenol, 2,6-dichloro-, acetate (C8H6Cl2O2) | YNSMUWSJRXDNLN-UHFFFAOYSA-N | CC(=O)OC1=C(C=CC=C1Cl)Cl | 1882 | 10 | 1 | 2 |
| Benzoic acid, 3,5-dichloro-, methyl ester (C8H6Cl2O2) | BTEVDFJXGLQUDS-UHFFFAOYSA-N | COC(=O)C1=CC(=CC(=C1)Cl)Cl | 3639 | 203 | 1 | 1 |
| Phenol, 2,5-dichloro-, acetate (C8H6Cl2O2) | XSJDGJRHRDWQOR-UHFFFAOYSA-N | CC(=O)OC1=C(C=CC(=C1)Cl)Cl | 1041 | 0 | 1 | 2 |
| Methyl 2,4-dichlorobenzoate (C8H6Cl2O2) | VCRWILYAWSRHBN-UHFFFAOYSA-N | COC(=O)C1=C(C=C(C=C1)Cl)Cl | 2132 | 60 | 1 | 2 |
| Phenol, 2,3-dichloro-, acetate (C8H6Cl2O2) | UGURLMCNLGTRCL-UHFFFAOYSA-N | CC(=O)OC1=C(C(=CC=C1)Cl)Cl | 1051 | 10 | 1 | 2 |
| Phenol, 2,4-dichloro-, acetate (C8H6Cl2O2) | KGXUYVOKSRCTEK-UHFFFAOYSA-N | CC(=O)OC1=C(C=C(C=C1)Cl)Cl | 691 | 0 | 2 | 2 |
| Benzoic acid, 2,4,6-trichloro- (C7H3Cl3O2) | RAFFVQBMVYYTQS-UHFFFAOYSA-N | C1=C(C=C(C(=C1Cl)C(=O)O)Cl)Cl | 6949 | 90 | 1 | 2 |
| Benzene, 2,4,5-trichloro-1-ethoxy (C8H7Cl3O) | XMLUZDBYNTZUSI-UHFFFAOYSA-N | CCOC1=CC(=C(C=C1Cl)Cl)Cl | 2372 | 20 | 1 | 2 |
| Benzene, 2,4-dichloro-1-(trichloromethyl)- (C7H3Cl5) | KZSNBJMYJWDVTK-UHFFFAOYSA-N | C1=CC(=C(C=C1Cl)Cl)C(Cl)(Cl)Cl | 239 | 7879 | 2 | 1 |
| α,α,α-3,4-Pentachlorotoluene (C7H3Cl5) | ATYLRBXENHNROH-UHFFFAOYSA-N | C1=CC(=C(C=C1C(Cl)(Cl)Cl)Cl)Cl | 400 | 8039 | 2 | 1 |
| Benzene, pentachloromethyl- (C7H3Cl5) | AVSIMRGRHWKCAY-UHFFFAOYSA-N | CC1=C(C(=C(C(=C1Cl)Cl)Cl)Cl)Cl | 6389 | 7839 | 1 | 1 |
| 3-(2,6-Dichlorophenoxy)prop-1-yne (C9H6Cl2O) | HCGOBPUSIBTNRV-UHFFFAOYSA-N | C#CCOC1=C(C=CC=C1Cl)Cl | 3974 | 9489 | 1 | 1 |
| Etridiazole (C5H5Cl3N2OS) | KQTVWCSONPJJPE-UHFFFAOYSA-N | CCOC1=NC(=NS1)C(Cl)(Cl)Cl | 1497 | 9999 | 1 | 1 |
| 2-(4-Chlorophenethyl) isothiocyanate (C9H8ClNS) | MRJJYUJULSZFDV-UHFFFAOYSA-N | C1=CC(=CC=C1CCN=C=S)Cl | 2666 | 38 | 1 | 2 |
| N-(2-Chloroethyl)benzamide (C9H10ClNO) | FYQJUYCGPLFWQR-UHFFFAOYSA-N | C1=CC=C(C=C1)C(=O)NCCCl | 2039 | 559 | 1 | 1 |
| Phenyl β-chloropropionate (C9H9ClO2) | RAFRTSDUWORDLA-UHFFFAOYSA-N | C1=CC=C(C=C1)OC(=O)CCCl | 409 | 20 | 2 | 2 |
| 2-Chloroethyl benzoate (C9H9ClO2) | ANPPGQUFDXLAGY-UHFFFAOYSA-N | C1=CC=C(C=C1)C(=O)OCCCl | 380 | 20 | 2 | 2 |
| p-(2-Chloroethoxy)benzaldehyde (C9H9ClO2) | HBHHMVNKQWECIS-UHFFFAOYSA-N | C1=CC(=CC=C1C=O)OCCCl | 5480 | 74 | 1 | 2 |
| 1-(p-Chlorophenylthio)-2-propanol (C9H11ClOS) | CHHYHUZLMAKWAS-UHFFFAOYSA-N | CC(CSC1=CC=C(C=C1)Cl)O | 2043 | 0 | 1 | 2 |
| Acetamide, N-(3-methylphenyl)-2-chloro- (C9H10ClNO) | GYMACIQGUHXSLT-UHFFFAOYSA-N | CC1=CC(=CC=C1)NC(=O)CCl | 8127 | 440 | 1 | 1 |
| Methyl 2-(4-chlorophenyl) acetate (C9H9ClO2) | WWIYGBWRUXQDND-UHFFFAOYSA-N | COC(=O)CC1=CC=C(C=C1)Cl | 2282 | 30 | 1 | 2 |
| Methyl α-chlorophenylacetate (C9H9ClO2) | XOIOYHPJZJLTGK-UHFFFAOYSA-N | COC(=O)C(C1=CC=CC=C1)Cl | 1955 | 275 | 1 | 1 |
| Chloroacetic acid, 3-methylphenyl ester (C9H9ClO2) | CEYGBRILXUZJEF-UHFFFAOYSA-N | CC1=CC(=CC=C1)OC(=O)CCl | 1281 | 0 | 1 | 2 |
| Propanamide, N-(4-chlorophenyl)- (C9H10ClNO) | FFHGCIYKPNISDY-UHFFFAOYSA-N | CCC(=O)NC1=CC=C(C=C1)Cl | 1300 | 0 | 1 | 2 |
| 2-Chloropropionanilide (C9H10ClNO) | VCVUMBWLSGNGFA-UHFFFAOYSA-N | CCC(=O)NC1=CC=CC=C1Cl | 2100 | 2000 | 1 | 1 |
| 4-Ethoxybenzoyl chloride (C9H9ClO2) | XLWQUESMILVIPR-UHFFFAOYSA-N | CCOC1=CC=C(C=C1)C(=O)Cl | 1058 | 9999 | 1 | 1 |
| Benzoic acid, 3-chloro-, ethyl ester (C9H9ClO2) | LVFRSNCBCHABAM-UHFFFAOYSA-N | CCOC(=O)C1=CC(=CC=C1)Cl | 1952 | 200 | 1 | 1 |
| Ethyl-2-chlorobenzoate (C9H9ClO2) | RETLCWPMLJPOTP-UHFFFAOYSA-N | CCOC(=O)C1=CC=CC=C1Cl | 1311 | 150 | 1 | 1 |
| Ethyl-4-chlorobenzoate (C9H9ClO2) | RWBYCMPOFNRISR-UHFFFAOYSA-N | CCOC(=O)C1=CC=C(C=C1)Cl | 1702 | 270 | 1 | 1 |
| Ethanone, 1-(3-chloro-4-methoxyphenyl)- (C9H9ClO2) | QILWOKAXHOAFOF-UHFFFAOYSA-N | CC(=O)C1=CC(=C(C=C1)OC)Cl | 3373 | 0 | 1 | 2 |
| Phenol, 4-chloro-3-methyl-, acetate (C9H9ClO2) | LKKDOFSIQKPJLX-UHFFFAOYSA-N | CC1=C(C=CC(=C1)OC(=O)C)Cl | 931 | 0 | 1 | 2 |
| Methyl 4-chloro-3-methylbenzoate (C9H9ClO2) | QOTGNXMPQNGYDM-UHFFFAOYSA-N | CC1=C(C=CC(=C1)C(=O)OC)Cl | 2873 | 160 | 1 | 1 |
| 2,4-Dichlorophenylacetic acid (C8H6Cl2O2) | GXMWLJKTGBZMBH-UHFFFAOYSA-N | C1=CC(=C(C=C1Cl)Cl)CC(=O)O | 2701 | 1814 | 1 | 1 |
| 3,4-Dichlorophenylacetic acid (C8H6Cl2O2) | ZOUPGSMSNQLUNW-UHFFFAOYSA-N | C1=CC(=C(C=C1CC(=O)O)Cl)Cl | 3023 | 50 | 1 | 2 |
| 1,2-Benzenedicarbonyl dichloride (C8H4Cl2O2) | FYXKZNLBZKRYSS-UHFFFAOYSA-N | C1=CC=C(C(=C1)C(=O)Cl)C(=O)Cl | 0 | 9999 | 2 | 1 |
| 1,3-Benzenedicarbonyl dichloride (C8H4Cl2O2) | FDQSRULYDNDXQB-UHFFFAOYSA-N | C1=CC(=CC(=C1)C(=O)Cl)C(=O)Cl | 84 | 9999 | 2 | 1 |
| 3,5-Dichloro-2-hydroxyacetophenone (C8H6Cl2O2) | CJFYGRLJDKWMDI-UHFFFAOYSA-N | CC(=O)C1=CC(=CC(=C1O)Cl)Cl | 3669 | 0 | 1 | 2 |
| Benzeneacetonitrile, 2,3,6-trichloro- (C8H4Cl3N) | ZUZLGSONCFESPE-UHFFFAOYSA-N | C1=CC(=C(C(=C1Cl)CC#N)Cl)Cl | 6236 | 9999 | 1 | 1 |
| Benzenemethanol, 2,4-dichloro-α-(chloromethyl)- (C8H7Cl3O) | XHEPANNURIQWRM-UHFFFAOYSA-N | C1=CC(=C(C=C1Cl)Cl)C(CCl)O | 326 | 4 | 2 | 2 |
| 2,2,2-Trichloro-1-phenylethanol (C8H7Cl3O) | ABFRBTDJEKZSRM-UHFFFAOYSA-N | C1=CC=C(C=C1)C(C(Cl)(Cl)Cl)O | 10 | 0 | 2 | 2 |
| 1-(2,4,5-Trichlorophenyl)ethanol (C8H7Cl3O) | CZTWWYRPCFIOMY-UHFFFAOYSA-N | CC(C1=CC(=C(C=C1Cl)Cl)Cl)O | 2650 | 350 | 1 | 1 |
| Ethanone, 1-(2,3,4-trichlorophenyl)- (C8H5Cl3O) | BXJZZJYNVIDEKG-UHFFFAOYSA-N | CC(=O)C1=C(C(=C(C=C1)Cl)Cl)Cl | 2709 | 0 | 1 | 2 |
| α,α,α',α'-Tetrachloro-o-xylene (C8H6Cl4) | UFJYKWQUTDGGPV-UHFFFAOYSA-N | C1=CC=C(C(=C1)C(Cl)Cl)C(Cl)Cl | 1459 | 9999 | 1 | 1 |
| Benzene, 1,2,3,5-tetrachloro-4,6-dimethyl- (C8H6Cl4) | NTUBJKOTTSFEEV-UHFFFAOYSA-N | CC1=C(C(=C(C(=C1Cl)Cl)Cl)C)Cl | 5899 | 9999 | 1 | 1 |
| Trichloroacetic acid, morpholide (C6H8Cl3NO2) | GDPGGCADYVWIBK-UHFFFAOYSA-N | C1COCCN1C(=O)C(Cl)(Cl)Cl | 761 | 240 | 2 | 1 |
| Benzene, (4-chlorobutoxy)- (C10H13ClO) | JKXCPAVECBFBOC-UHFFFAOYSA-N | C1=CC=C(C=C1)OCCCCCl | 909 | 10 | 1 | 2 |
| 4-Acetamidobenzyl chloride (C9H10ClNO) | DDKYWWMTJDDHKO-UHFFFAOYSA-N | C1=CC(=CC=C1CC(=O)N)CCl | 1384 | 1744 | 1 | 1 |
| 3-Chlorobenzoylacetonitrile (C9H6ClNO) | IUDFNNHFARLIPF-UHFFFAOYSA-N | C1=CC(=CC(=C1)Cl)C(=O)CC#N | 1181 | 0 | 1 | 2 |
| 4-Chlorobenzoylacetonitrile (C9H6ClNO) | JYOUFPNYTOFCSJ-UHFFFAOYSA-N | C1=CC(=CC=C1C(=O)CC#N)Cl | 821 | 0 | 1 | 2 |
| 3-(2-Chlorophenyl)propionic acid (C9H9ClO2) | KZMDFTFGWIVSNQ-UHFFFAOYSA-N | C1=CC=C(C(=C1)CCC(=O)O)Cl | 1495 | 9623 | 1 | 1 |
| 3-(4-Chlorophenyl)propionic acid (C9H9ClO2) | BBSLOKZINKEUCR-UHFFFAOYSA-N | C1=CC(=CC=C1CCC(=O)O)Cl | 2597 | 201 | 1 | 1 |
| 2-(4-Chlorophenyl)propanoic acid (C9H9ClO2) | YOZILQVNIWNPFP-UHFFFAOYSA-N | CC(C1=CC=C(C=C1)Cl)C(=O)O | 2292 | 10 | 1 | 2 |
| Benzene, 1,3,5-trichloro-2,4,6-trimethyl- (C9H9Cl3) | VMNISWKTOHUZQN-UHFFFAOYSA-N | CC1=C(C(=C(C(=C1Cl)C)Cl)C)Cl | 6425 | 9999 | 1 | 1 |
| 4-Chlorobutyrophenone (C10H11ClO) | GHEFQKHLHFXSBR-UHFFFAOYSA-N | C1=CC=C(C=C1)C(=O)CCCCl | 497 | 220 | 2 | 1 |
| 4'-(2-Chloroethyl)acetophenone (C10H11ClO) | RGVUACHOYCYNMB-UHFFFAOYSA-N | CC(=O)C1=CC=C(C=C1)CCCl | 1789 | 129 | 1 | 1 |
| 2-phenylbutyryl chloride (C10H11ClO) | QGXMHCMPIAYMGT-UHFFFAOYSA-N | CCC(C1=CC=CC=C1)C(=O)Cl | 781 | 260 | 2 | 1 |
| Benzoyl chloride, 4-propyl- (C10H11ClO) | NZYPCJXREKMMCJ-UHFFFAOYSA-N | CCCC1=CC=C(C=C1)C(=O)Cl | 289 | 9999 | 2 | 1 |
| 1-[4-(chloromethyl)phenyl]-1-butanone (C10H11ClO) | XLCJPQYALLFIPW-UHFFFAOYSA-N | CCCC(=O)C1=CC=C(C=C1)Cl | 99 | 1306 | 2 | 1 |
| Clotermine (C10H14ClN) | HXCXASJHZQXCKK-UHFFFAOYSA-N | CC(C)(CC1=CC=CC=C1Cl)N | 0 | 0 | 2 | 2 |
| Chlorphentermine (C10H14ClN) | ZCKAMNXUHHNZLN-UHFFFAOYSA-N | CC(C)(CC1=CC=C(C=C1)Cl)N | 0 | 0 | 2 | 2 |
| p-Chloro-α,α-dimethylphenethyl alcohol (C10H13ClO) | WAAJRPRSQXYYAA-UHFFFAOYSA-N | CC(C)(CC1=CC=C(C=C1)Cl)O | 30 | 20 | 2 | 2 |
| Phenol, 2-chloro-6-(1,1-dimethylethyl)- (C10H13ClO) | UBHJDCKSYYGMRI-UHFFFAOYSA-N | CC(C)(C)C1=C(C(=CC=C1)Cl)O | 3403 | 90 | 1 | 2 |
| phenol, 2-chloro, 4-t-butyl (C10H13ClO) | PRLINSMUYJWPBL-UHFFFAOYSA-N | CC(C)(C)C1=CC(=C(C=C1)O)Cl | 2179 | 50 | 1 | 2 |
| Chlorothymol (C10H13ClO) | KFZXVMNBUMVKLN-UHFFFAOYSA-N | CC1=CC(=C(C=C1Cl)C(C)C)O | 2989 | 349 | 1 | 1 |
| 2,4,6-Trimethylbenzoyl chloride (C10H11ClO) | UKRQMDIFLKHCRO-UHFFFAOYSA-N | CC1=CC(=C(C(=C1)C)C(=O)Cl)C | 220 | 9999 | 2 | 1 |
| 1,3-Dimethyl-4,6-bis(chloromethyl)benzene (C10H12Cl2) | JALHTPIEJQBDEN-UHFFFAOYSA-N | CC1=CC(=C(C=C1CCl)CCl)C | 2142 | 9999 | 1 | 1 |
| Benzene, 1,2-bis-(chloromethyl)-4,5-dimethyl (C10H12Cl2) | UIMFHDVFMPUGMO-UHFFFAOYSA-N | CC1=CC(=C(C=C1C)CCl)CCl | 1709 | 9999 | 1 | 1 |
| Benzene, 1,4-bis-(chloromethyl)-2,5-dimethyl (C10H12Cl2) | UYRPOMMBPQHVMN-UHFFFAOYSA-N | CC1=CC(=C(C=C1CCl)C)CCl | 1903 | 9999 | 1 | 1 |
| Acetamide, N-tetrahydrofurfuryl-2,2-dichloro- (C7H11Cl2NO2) | JUNUKZRYFJPFGM-UHFFFAOYSA-N | C1CC(OC1)CNC(=O)C(Cl)Cl | 180 | 0 | 2 | 2 |
| Piperidine, 1-trichloroacetyl-, (C7H10Cl3NO) | COHUYGSEUCDFQT-UHFFFAOYSA-N | C1CCN(CC1)C(=O)C(Cl)(Cl)Cl | 283 | 580 | 2 | 1 |
| Benzene, (5-chloropentyl)- (C11H15Cl) | UXJLBVYYDZDPBV-UHFFFAOYSA-N | C1=CC=C(C=C1)CCCCCCl | 895 | 16 | 1 | 2 |
| Benzene, [3-chloro-3-methylbutyl] (C11H15Cl) | GCAZETCXAVCJCN-UHFFFAOYSA-N | CC(C)(CCC1=CC=CC=C1)Cl | 100 | 400 | 2 | 1 |
| Benzene, 1-(chloromethyl)-4-(1,1-dimethylethyl) (C11H15Cl) | WAXIFMGAKWIFDQ-UHFFFAOYSA-N | CC(C)(C)C1=CC=C(C=C1)CCl | 2349 | 1099 | 1 | 1 |
| 1-Chloromethyl-3-(1,1-dimethylethyl)benzene (C11H15Cl) | QZWCABOLGIVZCP-UHFFFAOYSA-N | CC(C)(C)C1=CC=CC(=C1)CCl | 1300 | 470 | 1 | 1 |
| 1,2,4,5-Tetramethyl-3-(chloromethyl)benzene (C11H15Cl) | UGAPPXGBBWAIGT-UHFFFAOYSA-N | CC1=CC(=C(C(=C1C)CCl)C)C | 1531 | 9999 | 1 | 1 |
| Propanamide, N-tetrahydrofurfuryl-3-chloro- (C8H14ClNO2) | OAWKSFOYJKEQCH-UHFFFAOYSA-N | C1CC(OC1)CNC(=O)CCCl | 0 | 30 | 2 | 2 |
| Propanamide, N-tetrahydrofurfuryl-2-chloro- (C8H14ClNO2) | CCYFCLVMSOMMEF-UHFFFAOYSA-N | CC(C(=O)NCC1CCCO1)Cl | 50 | 100 | 2 | 2 |
| β-BHC (C6H6Cl6) | JLYXXMFPNIAWKQ-UHFFFAOYSA-N | C1(C(C(C(C(C1Cl)Cl)Cl)Cl)Cl)Cl | 279 | 159 | 2 | 1 |
| Lindane (C6H6Cl6) | JLYXXMFPNIAWKQ-GNIYUCBRSA-N | C1(C(C(C(C(C1Cl)Cl)Cl)Cl)Cl)Cl | 399 | 119 | 2 | 1 |
| BHC (C6H6Cl6) | JLYXXMFPNIAWKQ-UHFFFAOYSA-N | C1(C(C(C(C(C1Cl)Cl)Cl)Cl)Cl)Cl | 300 | 110 | 2 | 1 |
| 5-chlorovaleric acid, cyclobutyl ester (C9H15ClO2) | IJPXXYJIZRATCH-UHFFFAOYSA-N | C1CC(C1)OC(=O)CCCCCl | 0 | 260 | 2 | 1 |
| p-Chlorocinnamic acid (C9H7ClO2) | GXLIFJYFGMHYDY-ZZXKWVIFSA-N | C1=CC(=CC=C1/C=C/C(=O)O)Cl | 9999 | 1549 | 1 | 1 |
| m-Chlorocinnamic acid (C9H7ClO2) | FFKGOJWPSXRALK-SNAWJCMRSA-N | C1=CC(=CC(=C1)Cl)/C=C/C(=O)O | 9999 | 4049 | 1 | 1 |
| 2-Chlorocinnamic acid (C9H7ClO2) | KJRRTHHNKJBVBO-AATRIKPKSA-N | C1=CC=C(C(=C1)/C=C/C(=O)O)Cl | 1109 | 9999 | 1 | 1 |
| 3-Buten-2-one, 4-(2-chlorophenyl)- (C10H9ClO) | FHDSETHROOWFCQ-VOTSOKGWSA-N | CC(=O)/C=C/C1=CC=CC=C1Cl | 1231 | 9999 | 1 | 1 |
| 3-Buten-2-one, 4-(4-chlorophenyl)- (C10H9ClO) | UUKRKWJGNHNTRG-NSCUHMNNSA-N | CC(=O)/C=C/C1=CC=C(C=C1)Cl | 5459 | 2849 | 1 | 1 |
| 2,5-Cyclohexadiene-1,4-dione, 2,5-dichloro-3,6-dihydroxy- (C6H2Cl2O4) | IPPWILKGXFOXHO-UHFFFAOYSA-N | C1(=C(C(=O)C(=C(C1=O)Cl)O)Cl)O | 9999 | 1239 | 1 | 1 |
| p-Benzoquinone, 2,3,5,6-tetrachloro- (C6Cl4O2) | UGNWTBMOAKPKBL-UHFFFAOYSA-N | C1(=C(C(=O)C(=C(C1=O)Cl)Cl)Cl)Cl | 4899 | 4569 | 1 | 1 |
| Tetrachloro-o-benzoquinone (C6Cl4O2) | VRGCYEIGVVTZCC-UHFFFAOYSA-N | C1(=C(C(=O)C(=O)C(=C1Cl)Cl)Cl)Cl | 1109 | 1779 | 1 | 1 |
| 2-Furoic acid, 3-chloroprop-2-enyl ester (C8H7ClO3) | IXYKCWJROBXCPM-DUXPYHPUSA-N | C1=COC(=C1)C(=O)OC/C=C/Cl | 150 | 3584 | 2 | 1 |
| 2-Chloro-5-methoxybenzimidazole (C8H7ClN2O) | FMDGYQOERIOABX-UHFFFAOYSA-N | COC1=CC2=C(C=C1)N=C(N2)Cl | 7443 | 68 | 1 | 2 |
| Cloxyquin (C9H6ClNO) | CTQMJYWDVABFRZ-UHFFFAOYSA-N | C1=CC2=C(C=CC(=C2N=C1)O)Cl | 9999 | 30 | 1 | 2 |
| Quinoline, 4,7-dichloro- (C9H5Cl2N) | HXEWMTXDBOQQKO-UHFFFAOYSA-N | C1=CC2=C(C=CN=C2C=C1Cl)Cl | 9999 | 5909 | 1 | 1 |
| Naphthalene, 1,5-dichloro- (C10H6Cl2) | ZBQZXTBAGBTUAD-UHFFFAOYSA-N | C1=CC2=C(C=CC=C2Cl)C(=C1)Cl | 9999 | 1100 | 1 | 1 |
| Naphthalene, 1,4-dichloro- (C10H6Cl2) | JDPKCYMVSKDOGS-UHFFFAOYSA-N | C1=CC=C2C(=C1)C(=CC=C2Cl)Cl | 9999 | 2019 | 1 | 1 |
| Naphthalene, 1,2-dichloro- (C10H6Cl2) | MOXLHAPKZWTHEX-UHFFFAOYSA-N | C1=CC=C2C(=C1)C=CC(=C2Cl)Cl | 9999 | 1100 | 1 | 1 |
| Naphthalene, 1,8-dichloro- (C10H6Cl2) | ADRYPAGQXFMVFP-UHFFFAOYSA-N | C1=CC2=C(C(=C1)Cl)C(=CC=C2)Cl | 9999 | 1300 | 1 | 1 |
| Naphthalene, 2,3-dichloro- (C10H6Cl2) | SKGXUFZRYNGFJS-UHFFFAOYSA-N | C1=CC=C2C=C(C(=CC2=C1)Cl)Cl | 9999 | 2300 | 1 | 1 |
| Naphthalene, 2,7-dichloro- (C10H6Cl2) | DWBQZSYTSNYEEJ-UHFFFAOYSA-N | C1=CC(=CC2=C1C=CC(=C2)Cl)Cl | 9999 | 1807 | 1 | 1 |
| 6-Chloropiperonal (C8H5ClO3) | VRNADRCOROWLJC-UHFFFAOYSA-N | C1OC2=C(O1)C=C(C(=C2)C=O)Cl | 7497 | 0 | 1 | 2 |
| 6-Chloropiperonyl chloride (C8H6Cl2O2) | APJKOQPCHGXQBI-UHFFFAOYSA-N | C1OC2=C(O1)C=C(C(=C2)CCl)Cl | 2202 | 9999 | 1 | 1 |
| 8-Chloro-2-methylquinoline (C10H8ClN) | VVLYDFPOGMTMFJ-UHFFFAOYSA-N | CC1=NC2=C(C=CC=C2Cl)C=C1 | 9999 | 1414 | 1 | 1 |
| 4-Chloroquinaldine (C10H8ClN) | HQAIROMRVBVWSK-UHFFFAOYSA-N | CC1=NC2=CC=CC=C2C(=C1)Cl | 9999 | 4259 | 1 | 1 |
| Quinoline, 7-chloro-2-methyl- (C10H8ClN) | WQZQFYRSYLXBGP-UHFFFAOYSA-N | CC1=NC2=C(C=C1)C=CC(=C2)Cl | 9999 | 2266 | 1 | 1 |
| Quinoline, 2-chloro-4-methyl- (C10H8ClN) | PFEIMKNQOIFKSW-UHFFFAOYSA-N | CC1=CC(=NC2=CC=CC=C12)Cl | 9999 | 5849 | 1 | 1 |
| 6-Chloro-2-methylquinoline (C10H8ClN) | OCCIBGIEIBQGAJ-UHFFFAOYSA-N | CC1=NC2=C(C=C1)C=C(C=C2)Cl | 9999 | 2758 | 1 | 1 |
| 1-(Chloromethyl)naphthalene (C11H9Cl) | XMWGTKZEDLCVIG-UHFFFAOYSA-N | C1=CC=C2C(=C1)C=CC=C2CCl | 2189 | 9999 | 1 | 1 |
| p-Chlorophenyl 2,3-epoxypropyl ether (C9H9ClO2) | KSLSZOOZWRMSAP-UHFFFAOYSA-N | C1C(O1)COC2=CC=C(C=C2)Cl | 7149 | 10 | 1 | 2 |
| 6-Chloro-4-chromanone (C12H16) | LLTDYHFVIVSQPJ-UHFFFAOYSA-N | C1COC2=C(C1=O)C=C(C=C2)Cl | 4891 | 39 | 1 | 2 |
| 4-Chlorophenyl cyclopropyl ketone (C10H9ClO) | OPSFCTBBDIDFJM-UHFFFAOYSA-N | C1CC1C(=O)C2=CC=C(C=C2)Cl | 1896 | 288 | 1 | 1 |
| trans-2-phenylcyclopropanecarbonyl chloride (C10H9ClO) | SODZBMGDYKEZJG-DTWKUNHWSA-N | C1[C@H]([C@@H]1C(=O)Cl)C2=CC=CC=C2 | 100 | 3033 | 2 | 1 |
| Benzene, (2,2-dichloro-1-methylcyclopropyl)- (C10H10Cl2) | NXYPRVQXSWVEOB-UHFFFAOYSA-N | CC1(CC1(Cl)Cl)C2=CC=CC=C2 | 688 | 4971 | 2 | 1 |
| 1-(2-Chlorophenyl)imidazole (C9H7ClN2) | ZGGZGKAVJNFVHE-UHFFFAOYSA-N | C1=CC=C(C(=C1)N2C=CN=C2)Cl | 9999 | 1808 | 1 | 1 |
| Imidazole, 1-(m-chlorophenyl)- (C9H7ClN2) | LEKTXVRARNYCNV-UHFFFAOYSA-N | C1=CC(=CC(=C1)Cl)N2C=CN=C2 | 9999 | 457 | 1 | 1 |
| 1-(4-Chlorophenyl)imidazole (C9H7ClN2) | BARLRKAYTDVUIS-UHFFFAOYSA-N | C1=CC(=CC=C1N2C=CN=C2)Cl | 9999 | 327 | 1 | 1 |
| Thianaphthene-2-carbonyl chloride (C9H5ClOS) | DNGLRCHMGDDHNC-UHFFFAOYSA-N | C1=CC=C2C(=C1)C=C(S2)C(=O)Cl | 2000 | 9999 | 1 | 1 |
| 7,7-Dichlorobicyclo(3,2,0)hept-2-en-6-one (C7H6Cl2O) | JBPBARAOHIDZPU-UHFFFAOYSA-N | C1C=CC2C1C(=O)C2(Cl)Cl | 70 | 240 | 2 | 1 |
| Trichloroacetic anhydride (C4Cl6O3) | MEFKFJOEVLUFAY-UHFFFAOYSA-N | C(=O)(C(Cl)(Cl)Cl)OC(=O)C(Cl)(Cl)Cl | 0 | 0 | 2 | 2 |
| Bis-2-chloroethyl-2-chloroethylphosphonate (C6H12Cl3O3P) | XXIDKSWYSYEFAG-UHFFFAOYSA-N | C(CCl)OP(=O)(CCCl)OCCCl | 0 | 6719 | 2 | 1 |
| Tris-(2-chloroethyl)orthoformate (C7H13Cl3O3) | RKHRVKJTCSHZCR-UHFFFAOYSA-N | C(CCl)OC(OCCCl)OCCCl | 20 | 0 | 2 | 2 |
| O-Mustard (C8H16Cl2OS2) | FWVCSXWHVOOTFJ-UHFFFAOYSA-N | C(CSCCCl)OCCSCCCl | 0 | 70 | 2 | 2 |
| Acetic acid, chloro-, 1-methyl-1,2-ethanediyl ester (C7H10Cl2O4) | RVKVLMMCZLSUGU-UHFFFAOYSA-N | CC(COC(=O)CCl)OC(=O)CCl | 40 | 0 | 2 | 2 |
| Malonic acid, 2,2-dichloroethyl ethyl ester (C7H10Cl2O4) | VTIZVXMFTSBZRS-UHFFFAOYSA-N | CCOC(=O)CC(=O)OCC(Cl)Cl | 0 | 30 | 2 | 2 |
| Glycine, N-(5-chlorovaleryl)-, methyl ester (C8H14ClNO3) | DSJFHGNKSIAIHB-UHFFFAOYSA-N | COC(=O)CNC(=O)CCCCCl | 140 | 9999 | 2 | 1 |
| Malonic acid, 2-chloropropyl ethyl ester (C8H13ClO4) | LNNJMHLJYAMEAG-UHFFFAOYSA-N | CCOC(=O)CC(=O)OCC(C)Cl | 0 | 50 | 2 | 2 |
| Trichloroacetic acid, hex-4-yn-3-yl ester (C8H9Cl3O2) | ALHKHMGMXWSIDK-UHFFFAOYSA-N | CCC(C#CC)OC(=O)C(Cl)(Cl)Cl | 0 | 5655 | 2 | 1 |
| 4-chlorobutanoic anhydride (C8H12Cl2O3) | VAXCCWFPTAPDMK-UHFFFAOYSA-N | C(CC(=O)OC(=O)CCCCl)CCl | 0 | 0 | 2 | 2 |
| Hexyl trichloroacetate (C8H13Cl3O2) | UJGLOWVVUOBEJU-UHFFFAOYSA-N | CCCCCCOC(=O)C(Cl)(Cl)Cl | 0 | 0 | 2 | 2 |
| Succinic acid, monochloride, 3-methylbutyl ester (C9H15ClO3) | NVHUMKDRBJJLIC-UHFFFAOYSA-N | CC(C)CCOC(=O)CCC(=O)Cl | 0 | 0 | 2 | 2 |
| 8-Chlorooctanoic acid, chloromethyl ester (C9H16Cl2O2) | YEDAJMGEJCGDOV-UHFFFAOYSA-N | C(CCCC(=O)OCCl)CCCCl | 0 | 0 | 2 | 2 |
| 7-Chlorooctanoic acid, chloromethyl ester (C9H16Cl2O2) | RVCBGSJHWXKROV-UHFFFAOYSA-N | CC(CCCCCC(=O)OCCl)Cl | 0 | 0 | 2 | 2 |
| 6-Chlorooctanoic acid, chloromethyl ester (C9H16Cl2O2) | XCSMASOBOZXNSF-UHFFFAOYSA-N | CCC(CCCCC(=O)OCCl)Cl | 0 | 0 | 2 | 2 |
| 5-Chlorooctanoic acid, chloromethyl ester (C9H16Cl2O2) | JUVOKRBZTUYKPF-UHFFFAOYSA-N | CCCC(CCCC(=O)OCCl)Cl | 0 | 0 | 2 | 2 |
| 2-Chlorooctanoic acid, chloromethyl ester (C9H16Cl2O2) | KZYDWRFXCQZHHV-UHFFFAOYSA-N | CCCCCCC(C(=O)OCCl)Cl | 0 | 0 | 2 | 2 |
| 3-Chlorooctanoic acid, chloromethyl ester (C9H16Cl2O2) | QVLVXAKSGCMENB-UHFFFAOYSA-N | CCCCCC(CC(=O)OCCl)Cl | 0 | 3593 | 2 | 1 |
| 4-Chlorooctanoic acid, chloromethyl ester (C9H16Cl2O2) | DFWMUPOBAPRFLT-UHFFFAOYSA-N | CCCCC(CCC(=O)OCCl)Cl | 0 | 0 | 2 | 2 |
| Heptyl dichloroacetate (C9H16Cl2O2) | DABKLGRTFPUJHU-UHFFFAOYSA-N | CCCCCCCOC(=O)C(Cl)Cl | 0 | 0 | 2 | 2 |
| octyl chloroacetate (C10H19ClO2) | NNXMXUSTTDFBDE-UHFFFAOYSA-N | CCCCCCCCOC(=O)CCl | 0 | 0 | 2 | 2 |
| 5-Chlorovaleric acid, pentyl ester (C10H19ClO2) | LAYFAERBACNODJ-UHFFFAOYSA-N | CCCCCOC(=O)CCCCCl | 0 | 300 | 2 | 1 |
| Propanoic acid, 3-chloro, heptyl ester (C10H19ClO2) | BQZQUWKYBJWQNL-UHFFFAOYSA-N | CCCCCCCOC(=O)CCCl | 0 | 0 | 2 | 2 |
| nonanoic acid, chloromethyl ester (C10H19ClO2) | NNVIRQBZFULPEI-UHFFFAOYSA-N | CCCCCCCCC(=O)OCCl | 120 | 0 | 2 | 2 |
| 2-chloroethyl octanoate (C10H19ClO2) | XBVCXCNGNQZCES-UHFFFAOYSA-N | CCCCCCCC(=O)OCCCl | 130 | 60 | 2 | 2 |
| Azelaoyl chloride (C9H14Cl2O2) | HGEVGSTXQGZPCL-UHFFFAOYSA-N | C(CCCC(=O)Cl)CCCC(=O)Cl | 0 | 809 | 2 | 1 |
| 5-Dodecyne, 12-chloro- (C12H21Cl) | DJJGBABLSKSRGV-UHFFFAOYSA-N | CCCCC#CCCCCCCCl | 0 | 0 | 2 | 2 |
| Dodecane, 1-chloro- (C12H25Cl) | YAYNEUUHHLGGAH-UHFFFAOYSA-N | CCCCCCCCCCCCCl | 60 | 0 | 2 | 2 |
| 5-chlorovaleric acid, pent-2-en-4-ynyl ester (C10H13ClO2) | ZDYVJHVEGPSABA-ZZXKWVIFSA-N | C#C/C=C/COC(=O)CCCCCl | 180 | 0 | 2 | 2 |
| 5-chlorovaleric acid, 3-methylbut-2-enyl ester (C10H17ClO2) | VWQQVHCGEXKURD-UHFFFAOYSA-N | CC(=CCOC(=O)CCCCCl)C | 130 | 0 | 2 | 2 |
| Fumaric acid, monochloride, pent-4-en-2-yl chloride (C9H11ClO3) | WBAFEBVXXKAFFY-AATRIKPKSA-N | CC(CC=C)OC(=O)/C=C/C(=O)Cl | 0 | 821 | 2 | 1 |
| 4-Chloro-3-nitrophenyl isothiocyanate (C7H3ClN2O2S) | ZXGZBHIDSJXKLE-UHFFFAOYSA-N | C1=CC(=C(C=C1N=C=S)[N+](=O)[O-])Cl | 8589 | 126 | 1 | 1 |
| Benzene, 1-chloro-2-isothiocyanato-4-nitro- (C7H3ClN2O2S) | QSSZYQDXSYYXKK-UHFFFAOYSA-N | C1=CC(=C(C=C1[N+](=O)[O-])N=C=S)Cl | 9999 | 0 | 1 | 2 |
| 1-Chloro-2,6-dinitrobenzene (C6H3ClN2O4) | BPPMIQPXQVIZNJ-UHFFFAOYSA-N | C1=CC(=C(C(=C1)[N+](=O)[O-])Cl)[N+](=O)[O-] | 6454 | 0 | 1 | 2 |
| Benzene, 4-chloro-1,2-dinitro- (C6H3ClN2O4) | QVQSOXMXXFZAKU-UHFFFAOYSA-N | C1=CC(=C(C=C1Cl)[N+](=O)[O-])[N+](=O)[O-] | 9999 | 0 | 1 | 2 |
| 1-Chloro-2,4-dinitrobenzene (C6H3ClN2O4) | VYZAHLCBVHPDDF-UHFFFAOYSA-N | C1=CC(=C(C=C1[N+](=O)[O-])[N+](=O)[O-])Cl | 5469 | 0 | 1 | 2 |
| 4-Chlorobenzenesulfonyl isocyanate (C7H4ClNO3S) | JGHDVROWMPBQSR-UHFFFAOYSA-N | C1=CC(=CC=C1S(=O)(=O)N=C=O)Cl | 1879 | 0 | 1 | 2 |
| 4-Chloro-3-nitrophenyl isocyanate (C7H3ClN2O3) | ZCPHLSPLEGBTCZ-UHFFFAOYSA-N | C1=CC(=C(C=C1N=C=O)[N+](=O)[O-])Cl | 7302 | 357 | 1 | 1 |
| Carbonochloridic acid, 4-nitrophenyl ester (C7H4ClNO4) | NXLNNXIXOYSCMB-UHFFFAOYSA-N | C1=CC(=CC=C1[N+](=O)[O-])OC(=O)Cl | 3609 | 529 | 1 | 1 |
| 3-(4-Chlorophenyl)-1,1-dimethyl-2-thiourea (C9H11ClN2S) | FWGDAHLBEXSAPT-UHFFFAOYSA-N | CN(C)C(=S)NC1=CC=C(C=C1)Cl | 4149 | 10 | 1 | 2 |
| 2-Chloro-N,N-dimethyl-4-nitroaniline (C8H9ClN2O2) | OZKAWTHGBGLZKC-UHFFFAOYSA-N | CN(C)C1=C(C=C(C=C1)[N+](=O)[O-])Cl | 9999 | 60 | 1 | 2 |
| Monuron (C9H11ClN2O) | BMLIZLVNXIYGCK-UHFFFAOYSA-N | CN(C)C(=O)NC1=CC=C(C=C1)Cl | 2349 | 30 | 1 | 2 |
| Urea, N'-(3-chlorophenyl)-N,N-dimethyl- (C9H11ClN2O) | QLQDWOFJALEHSP-UHFFFAOYSA-N | CN(C)C(=O)NC1=CC(=CC=C1)Cl | 2500 | 0 | 1 | 2 |
| 3,5-Dichlorosulfanilamide (C6H6Cl2N2O2S) | DVZMRTJKNJKEGV-UHFFFAOYSA-N | C1=C(C=C(C(=C1Cl)N)Cl)S(=O)(=O)N | 4649 | 70 | 1 | 2 |
| Swep (C8H7Cl2NO) | WOZQBERUBLYCEG-UHFFFAOYSA-N | COC(=O)NC1=CC(=C(C=C1)Cl)Cl | 5609 | 60 | 1 | 2 |
| Benzenesulfonyl chloride, 2,4,5-trichloro- (C6H2Cl4O2S) | WNVVRCKTQSCPAC-UHFFFAOYSA-N | C1=C(C(=CC(=C1Cl)Cl)Cl)S(=O)(=O)Cl | 2889 | 9919 | 1 | 1 |
| 2,3,4-Trichloro benzene sulfonyl chloride (C6H2Cl4O2S) | JDAJYNHGBUXIKS-UHFFFAOYSA-N | C1=CC(=C(C(=C1S(=O)(=O)Cl)Cl)Cl)Cl | 3472 | 9999 | 1 | 1 |
| 2,4,6-Trichlorobenzenesulfonyl chloride (C6H2Cl4O2S) | WHJAQKAAIOHCGN-UHFFFAOYSA-N | C1=C(C=C(C(=C1Cl)S(=O)(=O)Cl)Cl)Cl | 3571 | 9999 | 1 | 1 |
| Phenol, 2-nitro-3,4,6-trichloro- (C6H2Cl3NO3) | XWLBYVXDCGYXGY-UHFFFAOYSA-N | C1=C(C(=C(C(=C1Cl)Cl)[N+](=O)[O-])O)Cl | 3500 | 0 | 1 | 2 |
| Benzene, 3,4,5-trichloro-1,2-dimethoxy (C8H7Cl3O2) | VKNITLPENCJQOP-UHFFFAOYSA-N | COC1=CC(=C(C(=C1OC)Cl)Cl)Cl | 8000 | 0 | 1 | 2 |
| Benzene, 3,4,6-trichloro-1,2-dimethoxy (C8H7Cl3O2) | BGWJOOFPIQFVOT-UHFFFAOYSA-N | COC1=C(C(=C(C=C1Cl)Cl)Cl)OC | 9999 | 0 | 1 | 2 |
| Benzene, 1,2,4,5-tetrachloro-3-nitro- (C6HCl4NO2) | XQTLDIFVVHJORV-UHFFFAOYSA-N | C1=C(C(=C(C(=C1Cl)Cl)[N+](=O)[O-])Cl)Cl | 5009 | 190 | 1 | 1 |
| Benzene, 1,2,3,4-tetrachloro-5-nitro- (C6HCl4NO2) | MTBYTWZDRVOMBR-UHFFFAOYSA-N | C1=C(C(=C(C(=C1Cl)Cl)Cl)Cl)[N+](=O)[O-] | 3101 | 203 | 1 | 1 |
| Phenol, 2,3,5,6-tetrachloro-4-methoxy- (C7H4Cl4O2) | XIWJLPHQDBDOAN-UHFFFAOYSA-N | COC1=C(C(=C(C(=C1Cl)Cl)O)Cl)Cl | 4471 | 97 | 1 | 2 |
| Pentachlorothioanisole (C7H3Cl5S) | LGZZJTIUEJNNKV-UHFFFAOYSA-N | CSC1=C(C(=C(C(=C1Cl)Cl)Cl)Cl)Cl | 6159 | 679 | 1 | 1 |
| Pentachloroanisole (C7H3Cl5O) | BBABSCYTNHOKOG-UHFFFAOYSA-N | COC1=C(C(=C(C(=C1Cl)Cl)Cl)Cl)Cl | 6025 | 397 | 1 | 1 |
| 2-Pyridinecarboxylic acid, 4-amino-3,5,6-trichloro- (C6H3Cl3N2O2) | NQQVFXUMIDALNH-UHFFFAOYSA-N | C1(=C(C(=NC(=C1Cl)Cl)C(=O)O)Cl)N | 4349 | 50 | 1 | 2 |
| Pyridin-4-amine, 3,5-dichloro-2-trichloromethyl- (C6H3Cl5N2) | WEYZLTAYNCBWFI-UHFFFAOYSA-N | C1=C(C(=C(C(=N1)C(Cl)(Cl)Cl)Cl)N)Cl | 1099 | 7973 | 1 | 1 |
| Aklomide (C7H5ClN2O3) | GFGSZUNNBQXGMK-UHFFFAOYSA-N | C1=CC(=C(C=C1[N+](=O)[O-])Cl)C(=O)N | 6141 | 0 | 1 | 2 |
| 5-Chloro-2-nitrobenzoic acid (C7H4ClNO4) | ZKUYSJHXBFFGPU-UHFFFAOYSA-N | C1=CC(=C(C=C1Cl)C(=O)O)[N+](=O)[O-] | 9999 | 0 | 1 | 2 |
| 2-Chloro-5-nitrobenzoic acid (C7H4ClNO4) | QUEKGYQTRJVEQC-UHFFFAOYSA-N | C1=CC(=C(C=C1[N+](=O)[O-])C(=O)O)Cl | 9999 | 0 | 1 | 2 |
| Benzoic acid, 3-chloro-4-nitro- (C7H4ClNO4) | TZPGGFYKIOBMCN-UHFFFAOYSA-N | C1=CC(=C(C=C1C(=O)O)Cl)[N+](=O)[O-] | 9999 | 0 | 1 | 2 |
| 4-Chloro-2-nitrobenzoic acid (C7H4ClNO4) | JAHIPDTWWVYVRV-UHFFFAOYSA-N | C1=CC(=C(C=C1Cl)[N+](=O)[O-])C(=O)O | 9999 | 0 | 1 | 2 |
| Benzoic acid, 4-chloro-3-nitro- (C7H4ClNO4) | DFXQXFGFOLXAPO-UHFFFAOYSA-N | C1=CC(=C(C=C1C(=O)O)[N+](=O)[O-])Cl | 9999 | 0 | 1 | 2 |
| Acetamide, N-(3-chlorophenyl)-2-methoxy- (C9H10ClNO2) | WFOKHEMHRLYTDD-UHFFFAOYSA-N | COCC(=O)NC1=CC(=CC=C1)Cl | 6636 | 0 | 1 | 2 |
| Chloroacetic acid, 4-methoxyphenyl ester (C9H9ClO3) | CWZUOZZTNYZVSQ-UHFFFAOYSA-N | COC1=CC=C(C=C1)OC(=O)CCl | 1271 | 0 | 1 | 2 |
| Carbamic acid, 3-chlorophenyl, ethyl ester (C9H10ClNO2) | LBVUCHGNHUEOFH-UHFFFAOYSA-N | CCOC(=O)NC1=CC(=CC=C1)Cl | 7056 | 0 | 1 | 2 |
| Carbamic acid, 4-chlorophenyl, ethyl ester (C9H10ClNO2) | WSKXXIMERYQVGJ-UHFFFAOYSA-N | CCOC(=O)NC1=CC=C(C=C1)Cl | 9999 | 0 | 1 | 2 |
| Carbamic acid, 2-chlorophenyl, ethyl ester (C9H10ClNO2) | SUMRDHQNSILLDV-UHFFFAOYSA-N | CCOC(=O)NC1=CC=CC=C1Cl | 6069 | 4540 | 1 | 1 |
| Methyl 3-chloro-4-methylcarbanilate (C9H10ClNO2) | QURFKMPDWGGSOK-UHFFFAOYSA-N | CC1=C(C=C(C=C1)NC(=O)OC)Cl | 9232 | 352 | 1 | 1 |
| 3,5-Dimethoxybenzoyl chloride (C9H9ClO3) | FTHPLWDYWAKYCY-UHFFFAOYSA-N | COC1=CC(=CC(=C1)C(=O)Cl)OC | 2032 | 9999 | 1 | 1 |
| 3,4-Dimethoxybenzoyl chloride (C9H9ClO3) | VIOBGCWEHLRBEP-UHFFFAOYSA-N | COC1=C(C=C(C=C1)C(=O)Cl)OC | 1201 | 9999 | 1 | 1 |
| Methyl 3-chloro-4-methoxybenzoate (C9H9ClO3) | PINQDVFQCCFACD-UHFFFAOYSA-N | COC1=C(C=C(C=C1)C(=O)OC)Cl | 2943 | 110 | 1 | 1 |
| 4-Chloro-2-methoxyphenol, acetate (C9H9ClO3) | QGTPWFVCCNXPON-UHFFFAOYSA-N | CC(=O)OC1=C(C=C(C=C1)Cl)OC | 360 | 0 | 2 | 2 |
| 2-Chloro-4-methoxyphenol, acetate (C9H9ClO3) | OZFXOOSAUSDUCK-UHFFFAOYSA-N | CC(=O)OC1=C(C=C(C=C1)OC)Cl | 350 | 20 | 2 | 2 |
| Chlordimeform (C10H13ClN2) | STUSTWKEFDQFFZ-UHFFFAOYSA-N | CC1=C(C=CC(=C1)Cl)N=CN(C)C | 9999 | 110 | 1 | 1 |
| 2-Chloro-5-nitrobenzoyl chloride (C7H3Cl2NO3) | OGLKKYALUKXVPQ-UHFFFAOYSA-N | C1=CC(=C(C=C1[N+](=O)[O-])C(=O)Cl)Cl | 216 | 9999 | 2 | 1 |
| 4-Chloro-3-nitrobenzoyl chloride (C7H3Cl2NO3) | IWLGXPWQZDOMSB-UHFFFAOYSA-N | C1=CC(=C(C=C1C(=O)Cl)[N+](=O)[O-])Cl | 370 | 9999 | 2 | 1 |
| 2-Chloro-4-nitrobenzoyl chloride (C7H3Cl2NO3) | KTHNITVDTYAHFF-UHFFFAOYSA-N | C1=CC(=C(C=C1[N+](=O)[O-])Cl)C(=O)Cl | 159 | 9999 | 2 | 1 |
| Chloramben, methyl ester (C8H7Cl2NO2) | DTSSCQVCVYZGSI-UHFFFAOYSA-N | COC(=O)C1=CC(=CC(=C1Cl)N)Cl | 7241 | 0 | 1 | 2 |
| Benzoic acid, 3,6-dichloro-2-methoxy- (C8H6Cl2O3) | IWEDIXLBFLAXBO-UHFFFAOYSA-N | COC1=C(C=CC(=C1C(=O)O)Cl)Cl | 7769 | 314 | 1 | 1 |
| Acetamide, N-(3-chlorophenyl)-2,2-dichloro- (C8H6Cl3NO) | LCMVVAIGAFVRAL-UHFFFAOYSA-N | C1=CC(=CC(=C1)Cl)NC(=O)C(Cl)Cl | 2642 | 80 | 1 | 2 |
| Acetyl chloride, (2,4-dichlorophenoxy)- (C8H5Cl3O2) | FUJSJWRORKKPAI-UHFFFAOYSA-N | C1=CC(=C(C=C1Cl)Cl)OCC(=O)Cl | 3056 | 30 | 1 | 2 |
| Dichloroacetic acid, 4-chlorophenyl ester (C8H5Cl3O2) | XBXQIOFECZDDPP-UHFFFAOYSA-N | C1=CC(=CC=C1OC(=O)C(Cl)Cl)Cl | 1521 | 0 | 1 | 2 |
| Chloroacetic acid, 3,4-dichlorophenyl ester (C8H5Cl3O2) | CNALHVZXVPTLGY-UHFFFAOYSA-N | C1=CC(=C(C=C1OC(=O)CCl)Cl)Cl | 791 | 0 | 2 | 2 |
| 2',4',5'-Trichloroacetanilide (C8H6Cl3NO) | VUSLTUGEJURGLB-UHFFFAOYSA-N | CC(=O)NC1=CC(=C(C=C1Cl)Cl)Cl | 961 | 1481 | 1 | 1 |
| Phenol, 2,4,6-trichloro-, acetate (C8H5Cl3O2) | RFOCPJZGJNKBOI-UHFFFAOYSA-N | CC(=O)OC1=C(C=C(C=C1Cl)Cl)Cl | 330 | 20 | 2 | 2 |
| Benzoic acid, 2,3,6-trichloro-, methyl ester (C8H5Cl3O2) | OAWDHDKEHFFNQQ-UHFFFAOYSA-N | COC(=O)C1=C(C=CC(=C1Cl)Cl)Cl | 2670 | 0 | 1 | 2 |
| Benzene, 1,2,3,5-tetrachloro-4-ethoxy- (C8H6Cl4O) | OMGPLLBLNOWRAA-UHFFFAOYSA-N | CCOC1=C(C(=C(C=C1Cl)Cl)Cl)Cl | 1348 | 0 | 1 | 2 |
| Benzonitrile, pentachloro- (C7Cl5N) | INICGXSKJYKEIV-UHFFFAOYSA-N | C(#N)C1=C(C(=C(C(=C1Cl)Cl)Cl)Cl)Cl | 7247 | 1631 | 1 | 1 |
| Simazine (C7H12ClN5) | ODCWYMIRDDJXKW-UHFFFAOYSA-N | CCNC1=NC(=NC(=N1)Cl)NCC | 9999 | 357 | 1 | 1 |
| Norazine (C7H12ClN5) | KLJYRZOFJHYOCO-UHFFFAOYSA-N | CC(C)NC1=NC(=NC(=N1)NC)Cl | 1099 | 0 | 1 | 2 |
| 4-Pyridinecarboxlylic acid, 2-amino-6-chloro-, ethyl ester (C8H9ClN2O2) | ZFTAEJJVVQTXKD-UHFFFAOYSA-N | CCOC(=O)C1=CC(=NC(=C1)Cl)N | 9999 | 57 | 1 | 2 |
| Benzyl 2-chloroethyl sulfone (C9H11ClO2S) | CKNUUVURUUCDGT-UHFFFAOYSA-N | C1=CC=C(C=C1)CS(=O)(=O)CCCl | 137 | 0 | 2 | 2 |
| Chloroacetic acid, 4-cyanophenyl ester (C9H6ClNO2) | APQLVUNYVFVTPC-UHFFFAOYSA-N | C1=CC(=CC=C1C#N)OC(=O)CCl | 901 | 0 | 1 | 2 |
| 4-Methyl-3-nitrobenzoyl chloride (C8H6ClNO3) | DXMHBBURYDVYAI-UHFFFAOYSA-N | CC1=C(C=C(C=C1)C(=O)Cl)[N+](=O)[O-] | 272 | 9999 | 2 | 1 |
| Propanoic acid, 2-(2-chlorophenoxy)- (C9H9ClO3) | ZGWNXHRVUJVMCP-UHFFFAOYSA-N | CC(C(=O)O)OC1=CC=CC=C1Cl | 2699 | 50 | 1 | 2 |
| [(4-Chloro-o-tolyl)oxy]acetic acid (C9H9ClO3) | WHKUVVPPKQRRBV-UHFFFAOYSA-N | CC1=C(C=CC(=C1)Cl)OCC(=O)O | 7756 | 92 | 1 | 2 |
| Acetylsalicyloyl chloride (C9H7ClO3) | DSGKWFGEUBCEIE-UHFFFAOYSA-N | CC(=O)OC1=CC=CC=C1C(=O)Cl | 100 | 721 | 2 | 1 |
| Aniline mustard (C10H13Cl2N) | ROSJKFFLIXTTAW-UHFFFAOYSA-N | C1=CC=C(C=C1)N(CCCl)CCCl | 1129 | 189 | 1 | 1 |
| Propanamide, N-(3-chlorophenyl)-3-chloro- (C9H9Cl2NO) | PPDCJGYLZPSGAW-UHFFFAOYSA-N | C1=CC(=CC(=C1)Cl)NC(=O)CCCl | 2082 | 60 | 1 | 2 |
| Benzoic acid, 4-chloro, 2-chloroethyl ester (C9H8Cl2O2) | AJIDYZYFPNXVGW-UHFFFAOYSA-N | C1=CC(=CC=C1C(=O)OCCCl)Cl | 731 | 0 | 2 | 2 |
| Benzoic acid, 2-chloro, 2-chloroethyl ester (C9H8Cl2O2) | OPAWQQDDCKSJAC-UHFFFAOYSA-N | C1=CC=C(C(=C1)C(=O)OCCCl)Cl | 921 | 30 | 1 | 2 |
| Acetamide, N-(3-methylphenyl)-2,2-dichloro- (C9H9Cl2NO) | JUXKBLADHIZLJE-UHFFFAOYSA-N | CC1=CC(=CC=C1)NC(=O)C(Cl)Cl | 4024 | 150 | 1 | 1 |
| Propanamide, N-(3-chlorophenyl)-2-chloro- (C9H9Cl2NO) | RMICMLBMAQRUJI-UHFFFAOYSA-N | CC(C(=O)NC1=CC(=CC=C1)Cl)Cl | 6546 | 360 | 1 | 1 |
| Dichloroacetic acid, 3-methylphenyl ester (C9H8Cl2O2) | PIRXAVOHMRJPRX-UHFFFAOYSA-N | CC1=CC(=CC=C1)OC(=O)C(Cl)Cl | 2022 | 0 | 1 | 2 |
| propanil (C9H9Cl2NO) | LFULEKSKNZEWOE-UHFFFAOYSA-N | CCC(=O)NC1=CC(=C(C=C1)Cl)Cl | 1996 | 22 | 1 | 2 |
| Ethyl-2,5-dichlorobenzoate (C9H8Cl2O2) | JSZYWIKNIZKJAN-UHFFFAOYSA-N | CCOC(=O)C1=C(C=CC(=C1)Cl)Cl | 2302 | 631 | 1 | 1 |
| Ethyl 2,4-dichlorobenzoate (C9H8Cl2O2) | ZBBGAUHWTZKKQQ-UHFFFAOYSA-N | CCOC(=O)C1=C(C=C(C=C1)Cl)Cl | 1306 | 86 | 1 | 2 |
| Chlorfenac (C8H5Cl3O2) | QZXCCPZJCKEPSA-UHFFFAOYSA-N | C1=CC(=C(C(=C1Cl)CC(=O)O)Cl)Cl | 4461 | 9999 | 1 | 1 |
| 1-(Pentachloroethyl)benzene (C8H5Cl5) | AXVLZOYLQOEKAO-UHFFFAOYSA-N | C1=CC=C(C=C1)C(C(Cl)(Cl)Cl)(Cl)Cl | 1290 | 400 | 1 | 1 |
| Benzoic acid, 2-chloro, 2-propynyl ester (C10H7ClO2) | KPSJNVKFAPXVOC-UHFFFAOYSA-N | C#CCOC(=O)C1=CC=CC=C1Cl | 841 | 350 | 1 | 1 |
| Terbuthylazine M (des-ethyl) (C7H12ClN5) | LMKQNTMFZLAJDV-UHFFFAOYSA-N | CC(C)(C)NC1=NC(=NC(=N1)N)Cl | 1780 | 0 | 1 | 2 |
| Beclamide (C10H12ClNO) | JPYQFYIEOUVJDU-UHFFFAOYSA-N | C1=CC=C(C=C1)CNC(=O)CCCl | 5905 | 3103 | 1 | 1 |
| Propanamide, N-(3-methylphenyl)-3-chloro- (C10H12ClNO) | XZPLSUAKDONHQY-UHFFFAOYSA-N | CC1=CC(=CC=C1)NC(=O)CCCl | 2793 | 70 | 1 | 2 |
| Butanamide,N-(2-chlorophenyl)- (C10H12ClNO) | SUINBJHPZPYMTI-UHFFFAOYSA-N | CCCC(=O)NC1=CC=CC=C1Cl | 1900 | 1200 | 1 | 1 |
| Butanamide, N-(3-chlorophenyl)- (C10H12ClNO) | QOKSOJARIYYAAO-UHFFFAOYSA-N | CCCC(=O)NC1=CC(=CC=C1)Cl | 1832 | 0 | 1 | 2 |
| Benzoic acid, 4-chloro, propyl ester (C10H11ClO2) | BLEFFSGNRQPNCA-UHFFFAOYSA-N | CCCOC(=O)C1=CC=C(C=C1)Cl | 110 | 0 | 2 | 2 |
| Butyric acid, 4-chlorophenyl ester (C10H11ClO2) | CLXDRKZHOMSWBH-UHFFFAOYSA-N | CCCC(=O)OC1=CC=C(C=C1)Cl | 1541 | 0 | 1 | 2 |
| 2-Chloro-2',6'-acetoxylidide (C10H12ClNO) | FPQQSNUTBWFFLB-UHFFFAOYSA-N | CC1=C(C(=CC=C1)C)NC(=O)CCl | 2385 | 194 | 1 | 1 |
| Propanamide, N-(3-methylphenyl)-2-chloro- (C10H12ClNO) | VOVIUZVPOZODGY-UHFFFAOYSA-N | CC1=CC(=CC=C1)NC(=O)C(C)Cl | 7056 | 621 | 1 | 1 |
| Chloroacetic acid, 3,5-dimethylphenyl ester (C10H11ClO2) | WQVBITVXKZGBJJ-UHFFFAOYSA-N | CC1=CC(=CC(=C1)OC(=O)CCl)C | 1541 | 0 | 1 | 2 |
| 2-Chloropropionic acid, 3-methylphenyl ester (C10H11ClO2) | PAAIIYQEPLSEAS-UHFFFAOYSA-N | CC1=CC(=CC=C1)OC(=O)C(C)Cl | 1431 | 0 | 1 | 2 |
| Benzoic acid, 4-chloro, isopropyl ester (C10H11ClO2) | VOLPFZRDHTWKTD-UHFFFAOYSA-N | CC(C)OC(=O)C1=CC=C(C=C1)Cl | 811 | 330 | 1 | 1 |
| Benzoic acid, 2-chloro, 1-methylethyl ester (C10H11ClO2) | ZQVYNQNVFVRHMT-UHFFFAOYSA-N | CC(C)OC(=O)C1=CC=CC=C1Cl | 1051 | 90 | 1 | 2 |
| Propanamide, N-(3-chlorophenyl)-2-methyl- (C10H12ClNO) | XJJWNMFVLXRGNJ-UHFFFAOYSA-N | CC(C)C(=O)NC1=CC(=CC=C1)Cl | 2873 | 0 | 1 | 2 |
| 2-Methylpropionic acid, 4-chlorophenyl ester (C10H11ClO2) | WPCAYDFRIDVTNI-UHFFFAOYSA-N | CC(C)C(=O)OC1=CC=C(C=C1)Cl | 2442 | 0 | 1 | 2 |
| 3,4-Dichlorobenzoylacetonitrile (C9H5Cl2NO) | HUEULXLHYYYTTP-UHFFFAOYSA-N | C1=CC(=C(C=C1C(=O)CC#N)Cl)Cl | 1561 | 90 | 1 | 2 |
| 3-(3,4-Dichlorophenyl)propionic acid (C9H8Cl2O2) | NHYJRLYFKZYPMO-UHFFFAOYSA-N | C1=CC(=C(C=C1CCC(=O)O)Cl)Cl | 2845 | 163 | 1 | 1 |
| Benzene, 1,3,3,3-tetrachloropropyl (C9H8Cl4) | JJRCOEWDFJHOFK-UHFFFAOYSA-N | C1=CC=C(C=C1)C(CC(Cl)(Cl)Cl)Cl | 400 | 300 | 2 | 1 |
| 2-(p-Chlorophenyl)-2-methylpropionic acid (C10H11ClO2) | SSFDAZXGUKDEAH-UHFFFAOYSA-N | CC(C)(C1=CC=C(C=C1)Cl)C(=O)O | 1460 | 0 | 1 | 2 |
| Benzenepropanenitrile, α,3-dichloro-2-methyl- (C10H9Cl2N) | YYJUXSGXHHPBTK-UHFFFAOYSA-N | CC1=C(C=CC=C1Cl)CC(C#N)Cl | 1496 | 291 | 1 | 1 |
| Phenol, 2,6-dichloro-4-(1-methylpropyl)- (C10H12Cl2O) | DZKJAGRVOGNQGW-UHFFFAOYSA-N | CCC(C)C1=CC(=C(C(=C1)Cl)O)Cl | 2042 | 20 | 1 | 2 |
| Acetamide, N-tetrahydrofurfuryl-2,2,2-trichloro- (C7H10Cl3NO2) | BYQNTQKJMWQRBO-UHFFFAOYSA-N | C1CC(OC1)CNC(=O)C(Cl)(Cl)Cl | 0 | 50 | 2 | 2 |
| Benzoyl chloride, 4-butyl- (C11H13ClO) | OUOWCSJYDCPVDM-UHFFFAOYSA-N | CCCCC1=CC=C(C=C1)C(=O)Cl | 220 | 9999 | 2 | 1 |
| 2-(p-Chlorophenyl)-3-methylbutyronitrile (C11H12ClN) | RBGSZIRWNWQDOK-UHFFFAOYSA-N | CC(C)C(C#N)C1=CC=C(C=C1)Cl | 705 | 0 | 2 | 2 |
| 4-tert-butylbenzoyl chloride (C11H13ClO) | WNLMYNASWOULQY-UHFFFAOYSA-N | CC(C)(C)C1=CC=C(C=C1)C(=O)Cl | 319 | 9999 | 2 | 1 |
| 5-Chlorovaleric acid, morpholide (C9H16ClNO2) | WVBRKFINNLXIAL-UHFFFAOYSA-N | C1COCCN1C(=O)CCCCCl | 370 | 9999 | 2 | 1 |
| 1-Chloro-6-phenylhexane (C12H17Cl) | RCVNHNOEUMFFDY-UHFFFAOYSA-N | C1=CC=C(C=C1)CCCCCCCl | 859 | 11 | 1 | 2 |
| 2,4-Dichloro-β-nitrostyrene (C8H5Cl2NO2) | LIWIJBBAMBDXME-ONEGZZNKSA-N | C1=CC(=C(C=C1Cl)Cl)/C=C/[N+](=O)[O-] | 2516 | 6603 | 1 | 1 |
| Benzoic acid, 4-chloro, 2-propenyl ester (C10H9ClO2) | BFQCHDDTURWEGT-UHFFFAOYSA-N | C=CCOC(=O)C1=CC=C(C=C1)Cl | 661 | 20 | 2 | 2 |
| Benzoic acid, 2-chloro, 2-propenyl ester (C10H9ClO2) | QAPRPPQBBCRDKG-UHFFFAOYSA-N | C=CCOC(=O)C1=CC=CC=C1Cl | 761 | 0 | 2 | 2 |
| 2,6-Dichlorocinnamic acid (C9H6Cl2O2) | OIPVGRCXMFBNAN-SNAWJCMRSA-N | C1=CC(=C(C(=C1)Cl)/C=C/C(=O)O)Cl | 499 | 9999 | 2 | 1 |
| Malonodinitrile, 4-chlorobenzylidene (C10H5ClN2) | FQSXBLOWLYPURG-UHFFFAOYSA-N | C1=CC(=CC=C1C=C(C#N)C#N)Cl | 5961 | 9999 | 1 | 1 |
| CS (C10H5ClN2) | JJNZXLAFIPKXIG-UHFFFAOYSA-N | C1=CC=C(C(=C1)C=C(C#N)C#N)Cl | 3980 | 9999 | 1 | 1 |
| Octachlorocyclopentene (C5Cl8) | DMZRCHJVWAKCAX-UHFFFAOYSA-N | C1(=C(C(C(C1(Cl)Cl)(Cl)Cl)(Cl)Cl)Cl)Cl | 169 | 3869 | 2 | 1 |
| Thiophene, 2,5-dichloro-3,4-dinitro- (C4Cl2N2O4S) | UNRSLHGBQKYYSF-UHFFFAOYSA-N | C1(=C(SC(=C1[N+](=O)[O-])Cl)Cl)[N+](=O)[O-] | 1388 | 0 | 1 | 2 |
| 1,3-Cyclopentadiene, 1,2,3,4-tetrachloro-5,5-dimethoxy- (C7H6Cl4O2) | UHSMEJQTFMHABA-UHFFFAOYSA-N | COC1(C(=C(C(=C1Cl)Cl)Cl)Cl)OC | 3819 | 9999 | 1 | 1 |
| 9H-Purine, 6-chloro-9-(methoxymethyl)- (C7H7ClN4O) | SZLMRHFSYVCXQX-UHFFFAOYSA-N | COCN1C=NC2=C1N=CN=C2Cl | 930 | 0 | 1 | 2 |
| Chloroxine (C9H5Cl2NO) | WDFKMLRRRCGAKS-UHFFFAOYSA-N | C1=CC2=C(C(=C(C=C2Cl)Cl)O)N=C1 | 9999 | 330 | 1 | 1 |
| Naphthalene, 1,3,7-trichloro- (C10H5Cl3) | CFEUGIGSIREATC-UHFFFAOYSA-N | C1=CC(=CC2=C(C=C(C=C21)Cl)Cl)Cl | 9999 | 1316 | 1 | 1 |
| Naphthalene, 2,3,6-trichloro- (C10H5Cl3) | ZYTLBFKRYKUCMJ-UHFFFAOYSA-N | C1=CC(=CC2=CC(=C(C=C21)Cl)Cl)Cl | 9999 | 1351 | 1 | 1 |
| 2-Naphthalenecarbonyl chloride (C11H7ClO) | XNLBCXGRQWUJLU-UHFFFAOYSA-N | C1=CC=C2C=C(C=CC2=C1)C(=O)Cl | 2229 | 9999 | 1 | 1 |
| 1-Naphthalenecarbonyl chloride (C11H7ClO) | NSNPSJGHTQIXDO-UHFFFAOYSA-N | C1=CC=C2C(=C1)C=CC=C2C(=O)Cl | 1659 | 9999 | 1 | 1 |
| PCB2 (C12H9Cl) | NMWSKOLWZZWHPL-UHFFFAOYSA-N | C1=CC=C(C=C1)C2=CC(=CC=C2)Cl | 9999 | 2491 | 1 | 1 |
| PCB 3 (C12H9Cl) | FPWNLURCHDRMHC-UHFFFAOYSA-N | C1=CC=C(C=C1)C2=CC=C(C=C2)Cl | 9999 | 2499 | 1 | 1 |
| N-(Chloromethyl)phthalimide (C9H6ClNO2) | JKGLRGGCGUQNEX-UHFFFAOYSA-N | C1=CC=C2C(=C1)C(=O)N(C2=O)CCl | 299 | 9999 | 2 | 1 |
| 1-Methyl-4-(chloromethyl)naphthalene (C12H11Cl) | QHXSBKTZBDHBKF-UHFFFAOYSA-N | CC1=CC=C(C2=CC=CC=C12)CCl | 2017 | 9999 | 1 | 1 |
| Naphthalene, 1-(chloromethyl)-2-methyl- (C12H11Cl) | STBYRSZXHDPASK-UHFFFAOYSA-N | CC1=C(C2=CC=CC=C2C=C1)CCl | 2521 | 9999 | 1 | 1 |
| 2H-1,3-Oxazine, 2-(4-chlorophenyl)tetrahydro- (C10H12ClNO) | IUSGGJOQPVRUFH-UHFFFAOYSA-N | C1CNC(OC1)C2=CC=C(C=C2)Cl | 1210 | 110 | 1 | 1 |
| Cyclopropanecarboxamide, N-(3-chlorophenyl)- (C10H10ClNO) | QOGAQGLPMQVRTR-UHFFFAOYSA-N | C1CC1C(=O)NC2=CC(=CC=C2)Cl | 3823 | 0 | 1 | 2 |
| Cyclopropanecarboxylic acid, 4-chlorophenyl ester (C10H9ClO2) | VDWGAQSMDCDWER-UHFFFAOYSA-N | C1CC1C(=O)OC2=CC=C(C=C2)Cl | 741 | 0 | 2 | 2 |
| 1-(2-Chlorophenyl)imidazoline-2-thione (C9H7ClN2S) | XOCLHGYTLWVXQB-UHFFFAOYSA-N | C1=CC=C(C(=C1)N2C=CNC2=S)Cl | 1727 | 9999 | 1 | 1 |
| 3-Chlorobenzo(b)thiophene-2-carboxylic acid (C9H5ClO2S) | HJTMIYKPPPYDRJ-UHFFFAOYSA-N | C1=CC=C2C(=C1)C(=C(S2)C(=O)O)Cl | 9999 | 135 | 1 | 1 |
| 3-Chlorobenzo[b]thiophene-2-carbonyl chloride (C9H4Cl2OS) | GWKSSMDJEWPKCM-UHFFFAOYSA-N | C1=CC=C2C(=C1)C(=C(S2)C(=O)Cl)Cl | 1756 | 9999 | 1 | 1 |
| 2-Chlorobenzo[b]thiophene-3-acetonitrile (C10H6ClNS) | CBWNFQCLFYLDJQ-UHFFFAOYSA-N | C1=CC=C2C(=C1)C(=C(S2)Cl)CC#N | 3937 | 9999 | 1 | 1 |
| Hexachloronorbornadiene (C7H2Cl6) | IHAXIFZOERJWDF-UHFFFAOYSA-N | C1=CC2(C(=C(C1(C2(Cl)Cl)Cl)Cl)Cl)Cl | 170 | 6696 | 2 | 1 |
| 1-Adamantanecarboxylic acid chloride (C11H15ClO) | MIBQYWIOHFTKHD-UHFFFAOYSA-N | C1C2CC3CC1CC(C2)(C3)C(=O)Cl | 10 | 50 | 2 | 2 |
| Clorethate (C5H4Cl6O3) | IEPBPSSCIZTJIF-UHFFFAOYSA-N | C(C(Cl)(Cl)Cl)OC(=O)OCC(Cl)(Cl)Cl | 0 | 0 | 2 | 2 |
| Tris(2-chloroethyl) phosphate (C6H12Cl3O4P) | HQUQLFOMPYWACS-UHFFFAOYSA-N | C(CCl)OP(=O)(OCCCl)OCCCl | 0 | 7359 | 2 | 1 |
| Succinic acid, 2,2-dichloroethyl ethyl ester (C8H12Cl2O4) | PFNFXPLZQOXXMK-UHFFFAOYSA-N | CCOC(=O)CCC(=O)OCC(Cl)Cl | 0 | 0 | 2 | 2 |
| Malonic acid, 2,2-dichloroethyl propyl ester (C8H12Cl2O4) | OSKMQXOYMHLDJW-UHFFFAOYSA-N | CCCOC(=O)CC(=O)OCC(Cl)Cl | 0 | 0 | 2 | 2 |
| L-Alanine, N-(5-chlorovaleryl)-, methyl ester (C9H16ClNO3) | XARKWCNFPJCPGN-UHFFFAOYSA-N | CC(C(=O)OC)NC(=O)CCCCCl | 0 | 400 | 2 | 1 |
| Succinic acid, 2-chloropropyl ethyl ester (C9H15ClO4) | JBPBSXHYWSBZCF-UHFFFAOYSA-N | CCOC(=O)CCC(=O)OCC(C)Cl | 0 | 20 | 2 | 2 |
| Malonic acid, 2-chloropropyl propyl ester (C9H15ClO4) | IQLRLUZXLPNYMG-UHFFFAOYSA-N | CCCOC(=O)CC(=O)OCC(C)Cl | 0 | 30 | 2 | 2 |
| 6-chlorohexyl trichloroacetate (C8H12Cl4O2) | SCWOHXIWHLOZJV-UHFFFAOYSA-N | C(CCCCl)CCOC(=O)C(Cl)(Cl)Cl | 0 | 0 | 2 | 2 |
| Dichloroacetamide, N,N-dibutyl- (C10H19Cl2NO) | ADUKNAPXUVHFPK-UHFFFAOYSA-N | CCCCN(CCCC)C(=O)C(Cl)Cl | 0 | 2582 | 2 | 1 |
| Heptyl trichloroacetate (C9H15Cl3O2) | RESXQXYZQVQEDT-UHFFFAOYSA-N | CCCCCCCOC(=O)C(Cl)(Cl)Cl | 0 | 0 | 2 | 2 |
| Propanamide, N,N-dibutyl-3-chloro- (C11H22ClNO) | ALAPRJIGPQQKKY-UHFFFAOYSA-N | CCCCN(CCCC)C(=O)CCCl | 160 | 2873 | 2 | 1 |
| Succinic acid, monochloride 2-ethylbutyl ester (C10H17ClO3) | LHYZWFBBDKPSSG-UHFFFAOYSA-N | CCC(CC)COC(=O)CCC(=O)Cl | 0 | 0 | 2 | 2 |
| Succinic acid, monochloride, 2-hexyl ester (C10H17ClO3) | RUIXFGUNJBUFDS-UHFFFAOYSA-N | CCCCC(C)OC(=O)CCC(=O)Cl | 0 | 0 | 2 | 2 |
| Succinic acid, monochloride 3-hexyl ester (C10H17ClO3) | YBFFUKFZMKXXSD-UHFFFAOYSA-N | CCCC(CC)OC(=O)CCC(=O)Cl | 0 | 0 | 2 | 2 |
| Propanamide, N,N-dibutyl-2-chloro- (C11H22ClNO) | AMBSMXGFOZRXNX-UHFFFAOYSA-N | CCCCN(CCCC)C(=O)C(C)Cl | 400 | 3934 | 2 | 1 |
| Succinic acid, monochloride, 4-methylpent-2-yl ester (C10H17ClO3) | OEDRJAVMOAYALV-UHFFFAOYSA-N | CC(C)CC(C)OC(=O)CCC(=O)Cl | 0 | 0 | 2 | 2 |
| Succinic acid, monochloride 2-methylpent-3-yl ester (C10H17ClO3) | LPYUZXWODZHGIK-UHFFFAOYSA-N | CCC(C(C)C)OC(=O)CCC(=O)Cl | 0 | 0 | 2 | 2 |
| Succinic acid, monochloride, 3,3-dimethylbut-2-yl ester (C10H17ClO3) | PUJBLZZZGPXNRQ-UHFFFAOYSA-N | CC(C(C)(C)C)OC(=O)CCC(=O)Cl | 0 | 0 | 2 | 2 |
| 9-Chlorononanoic acid, chloromethyl ester (C10H18Cl2O2) | AAVUFTKCHHFPHZ-UHFFFAOYSA-N | C(CCCCCl)CCCC(=O)OCCl | 0 | 0 | 2 | 2 |
| 8-Chlorononanoic acid, chloromethyl ester (C10H18Cl2O2) | XRIQYYVDKSLECK-UHFFFAOYSA-N | CC(CCCCCCC(=O)OCCl)Cl | 0 | 0 | 2 | 2 |
| 7-Chlorononanoic acid, chloromethyl ester (C10H18Cl2O2) | PYUVKWQQSVOCEA-UHFFFAOYSA-N | CCC(CCCCCC(=O)OCCl)Cl | 0 | 0 | 2 | 2 |
| 6-Chlorononanoic acid, chloromethyl ester (C10H18Cl2O2) | AGAUMIHMEOWJDE-UHFFFAOYSA-N | CCCC(CCCCC(=O)OCCl)Cl | 0 | 0 | 2 | 2 |
| 2-Chlorononanoic acid, chloromethyl ester (C10H18Cl2O2) | ZFVRVFPFKKNKCD-UHFFFAOYSA-N | CCCCCCCC(C(=O)OCCl)Cl | 0 | 0 | 2 | 2 |
| 3-Chlorononanoic acid, chloromethyl ester (C10H18Cl2O2) | QADYLIOQVFEZKX-UHFFFAOYSA-N | CCCCCCC(CC(=O)OCCl)Cl | 0 | 3202 | 2 | 1 |
| 4-Chlorononanoic acid, chloromethyl ester (C10H18Cl2O2) | COADCZUXYCMGLU-UHFFFAOYSA-N | CCCCCC(CCC(=O)OCCl)Cl | 0 | 0 | 2 | 2 |
| 5-Chlorononanoic acid, chloromethyl ester (C10H18Cl2O2) | MJVJQTXCHDQYKX-UHFFFAOYSA-N | CCCCC(CCCC(=O)OCCl)Cl | 0 | 0 | 2 | 2 |
| 5-chlorovaleric acid, hex-4-yn-3-yl ester (C11H17ClO2) | PVSFHUUTAKAGCC-UHFFFAOYSA-N | CCC(C#CC)OC(=O)CCCCCl | 891 | 240 | 1 | 1 |
| Decyl chloroformate (C11H21ClO2) | AZZCHVHSWUYCQA-UHFFFAOYSA-N | CCCCCCCCCCOC(=O)Cl | 0 | 100 | 2 | 2 |
| 5-Chlorovaleric acid, hexyl ester (C11H21ClO2) | ZFVGXJLTIIOCHW-UHFFFAOYSA-N | CCCCCCOC(=O)CCCCCl | 0 | 230 | 2 | 1 |
| Nonyl chloroacetate (C11H21ClO2) | OKWGMWNSHCOYJE-UHFFFAOYSA-N | CCCCCCCCCOC(=O)CCl | 0 | 0 | 2 | 2 |
| Propanoic acid, 3-chloro, octyl ester (C11H21ClO2) | APSFWUHAPFXKHS-UHFFFAOYSA-N | CCCCCCCCOC(=O)CCCl | 0 | 20 | 2 | 2 |
| Decanoic acid, chloromethyl ester (C11H21ClO2) | UXAJBSZQOZOHEI-UHFFFAOYSA-N | CCCCCCCCCC(=O)OCCl | 590 | 0 | 2 | 2 |
| Decanedioyl dichloride (C10H16Cl2O2) | WMPOZLHMGVKUEJ-UHFFFAOYSA-N | C(CCCCC(=O)Cl)CCCC(=O)Cl | 0 | 1489 | 2 | 1 |
| Dodecanoyl chloride (C12H23ClO) | NQGIJDNPUZEBRU-UHFFFAOYSA-N | CCCCCCCCCCCC(=O)Cl | 0 | 1399 | 2 | 1 |
| Octanoic acid, 3-chloroprop-2-enyl ester (C11H19ClO2) | DQDAHPGZXVTTSA-VQHVLOKHSA-N | CCCCCCCC(=O)OC/C=C/Cl | 180 | 2342 | 2 | 1 |
| Benzenesulfonyl chloride, 4-chloro-3-nitro- (C6H3Cl2NO4S) | SEWNAJIUKSTYOP-UHFFFAOYSA-N | C1=CC(=C(C=C1S(=O)(=O)Cl)[N+](=O)[O-])Cl | 1751 | 9999 | 1 | 1 |
| 5-Chloro-2,4-dimethoxyphenyl isothiocyanate (C9H8ClNO2S) | BXJOFCAXBGIDIR-UHFFFAOYSA-N | COC1=CC(=C(C=C1N=C=S)Cl)OC | 9999 | 0 | 1 | 2 |
| Monolinuron (C9H11ClN2O2) | LKJPSUCKSLORMF-UHFFFAOYSA-N | CN(C(=O)NC1=CC=C(C=C1)Cl)OC | 1250 | 0 | 1 | 2 |
| 5-Chloro-2,4-dimethoxyphenyl isocyanate (C9H8ClNO3) | COSVEXSXJCUYOT-UHFFFAOYSA-N | COC1=CC(=C(C=C1N=C=O)Cl)OC | 9999 | 0 | 1 | 2 |
| 1,3-Dichloro-4,6-dinitrobenzene (C6H2Cl2N2O4) | ZPXDNSYFDIHPOJ-UHFFFAOYSA-N | C1=C(C(=CC(=C1[N+](=O)[O-])Cl)Cl)[N+](=O)[O-] | 1702 | 0 | 1 | 2 |
| 3-(2,3-Dichlorophenyl)-1,1-dimethylurea (C9H10Cl2N2O) | ZVPQMESWKIQHGS-UHFFFAOYSA-N | CN(C)C(=O)NC1=C(C(=CC=C1)Cl)Cl | 330 | 2970 | 2 | 1 |
| Diuron (C9H10Cl2N2O) | XMTQQYYKAHVGBJ-UHFFFAOYSA-N | CN(C)C(=O)NC1=CC(=C(C=C1)Cl)Cl | 3757 | 27 | 1 | 2 |
| Carbamic acid, dimethyl-, 2,4-dichlorophenyl ester (C9H9Cl2NO2) | ZYYOAHAWVWGMJY-UHFFFAOYSA-N | CN(C)C(=O)OC1=C(C=C(C=C1)Cl)Cl | 6 | 508 | 2 | 1 |
| Benzene, 1,2,3,4-tetrachloro-5,6-dimethoxy- (C8H6Cl4O2) | NCYHCGGUQGDEQW-UHFFFAOYSA-N | COC1=C(C(=C(C(=C1Cl)Cl)Cl)Cl)OC | 7529 | 0 | 1 | 2 |
| Benzene, 1,2,4,5-tetrachloro-3,6-dimethoxy- (C8H6Cl4O2) | HICARXIPJINIRA-UHFFFAOYSA-N | COC1=C(C(=C(C(=C1Cl)Cl)OC)Cl)Cl | 5357 | 23 | 1 | 2 |
| Benzene, pentachloronitro- (C6Cl5NO2) | LKPLKUMXSAEKID-UHFFFAOYSA-N | C1(=C(C(=C(C(=C1Cl)Cl)Cl)Cl)Cl)[N+](=O)[O-] | 5041 | 38 | 1 | 2 |
| Acetic acid, [(3,5,6-trichloro-2-pyridinyl)oxy]- (C7H4Cl3NO3) | REEQLXCGVXDJSQ-UHFFFAOYSA-N | C1=C(C(=NC(=C1Cl)Cl)OCC(=O)O)Cl | 2961 | 38 | 1 | 2 |
| Picloram-methyl ester (C7H5Cl3N2O2) | RJQUHEYNLDNJLN-UHFFFAOYSA-N | COC(=O)C1=NC(=C(C(=C1Cl)N)Cl)Cl | 2292 | 0 | 1 | 2 |
| Acetanilide, 2-chloro-4'-nitro- (C8H7ClN2O3) | AZURFBCEYQYATI-UHFFFAOYSA-N | C1=CC(=CC=C1NC(=O)CCl)[N+](=O)[O-] | 6574 | 191 | 1 | 1 |
| 2-Chloro-4-nitrobenzhydrazide (C7H6ClN3O3) | XLDHQBNLMQYVBC-UHFFFAOYSA-N | C1=CC(=C(C=C1[N+](=O)[O-])Cl)C(=O)NN | 2026 | 68 | 1 | 2 |
| Acetamide, N-(3-nitrophenyl)-2-chloro- (C8H7ClN2O3) | UCBHRCPNMDOUMV-UHFFFAOYSA-N | C1=CC(=CC(=C1)[N+](=O)[O-])NC(=O)CCl | 5575 | 130 | 1 | 1 |
| Carbonochloridic acid, (4-nitrophenyl)methyl ester (C8H6ClNO4) | MHSGOABISYIYKP-UHFFFAOYSA-N | C1=CC(=CC=C1COC(=O)Cl)[N+](=O)[O-] | 3369 | 20 | 1 | 2 |
| Benzene, 2-chloro-5-methyl-1,3-dinitro- (C7H5ClN2O4) | JMDVARRGYWIJGZ-UHFFFAOYSA-N | CC1=CC(=C(C(=C1)[N+](=O)[O-])Cl)[N+](=O)[O-] | 6021 | 0 | 1 | 2 |
| Methyl 2-chloro-4-nitrobenzoate (C8H6ClNO4) | PICNSXCJRMYANX-UHFFFAOYSA-N | COC(=O)C1=C(C=C(C=C1)[N+](=O)[O-])Cl | 2812 | 83 | 1 | 2 |
| 3,4,5-Trimethoxybenzyl chloride (C10H13ClO3) | XXRUQNNAKXZSOS-UHFFFAOYSA-N | COC1=CC(=CC(=C1OC)OC)CCl | 2242 | 9999 | 1 | 1 |
| Chlorotoluron (C10H13ClN2O) | JXCGFZXSOMJFOA-UHFFFAOYSA-N | CC1=C(C=C(C=C1)NC(=O)N(C)C)Cl | 2000 | 0 | 1 | 2 |
| 3,4-Dichlorobenzyl N-methylcarbamate (C9H9Cl2NO2) | DSVOTYIOPGIVPP-UHFFFAOYSA-N | CNC(=O)OCC1=CC(=C(C=C1)Cl)Cl | 160 | 0 | 2 | 2 |
| 2,3-Dichlorobenzyl-N-methylcarbamate (C9H9Cl2NO2) | SESJCOBCXSACPH-UHFFFAOYSA-N | CNC(=O)OCC1=C(C(=CC=C1)Cl)Cl | 960 | 5425 | 1 | 1 |
| Acetamide, N-(4-methoxyphenyl)-2,2-dichloro- (C9H9Cl2NO2) | IKDULUPZZPGFFJ-UHFFFAOYSA-N | COC1=CC=C(C=C1)NC(=O)C(Cl)Cl | 5875 | 190 | 1 | 1 |
| 2,4-D methyl ester (C9H8Cl2O3) | HWIGZMADSFQMOI-UHFFFAOYSA-N | COC(=O)COC1=C(C=C(C=C1)Cl)Cl | 4762 | 9999 | 1 | 1 |
| Dichloroacetic acid, 4-methoxyphenyl ester (C9H8Cl2O3) | FYSUBKYBGWIHKT-UHFFFAOYSA-N | COC1=CC=C(C=C1)OC(=O)C(Cl)Cl | 1732 | 0 | 1 | 2 |
| Benzoic acid, 2,5-dichloro-3-hydroxy-6-methoxy- (C8H6Cl2O4) | XYHBJALHMANCCC-UHFFFAOYSA-N | COC1=C(C=C(C(=C1C(=O)O)Cl)O)Cl | 1857 | 13 | 1 | 2 |
| Dicamba methyl ester (C8H8Cl2O3) | AWSBKDYHGOOSML-UHFFFAOYSA-N | COC1=C(C=CC(=C1C(=O)OC)Cl)Cl | 2027 | 77 | 1 | 2 |
| Acetic acid, (2,4,5-trichlorophenoxy)- (C8H5Cl3O3) | SMYMJHWAQXWPDB-UHFFFAOYSA-N | C1=C(C(=CC(=C1Cl)Cl)Cl)OCC(=O)O | 4980 | 634 | 1 | 1 |
| 2,2',4',5'-Tetrachloroacetanilide (C8H5Cl4NO) | GQRWKOPRUXSOBA-UHFFFAOYSA-N | C1=C(C(=CC(=C1Cl)Cl)Cl)NC(=O)CCl | 2102 | 8624 | 1 | 1 |
| Acetamide, N-(3-chlorophenyl)-2,2,2-trichloro- (C8H5Cl4NO) | ZCNFWVGKVHRKCD-UHFFFAOYSA-N | C1=CC(=CC(=C1)Cl)NC(=O)C(Cl)(Cl)Cl | 1631 | 210 | 1 | 1 |
| Dichloroacetic acid, 3,4-dichlorophenyl ester (C8H4Cl4O2) | VEVXIXJKVRAVCE-UHFFFAOYSA-N | C1=CC(=C(C=C1OC(=O)C(Cl)Cl)Cl)Cl | 961 | 0 | 1 | 2 |
| 2,3,4,6-Tetrachlorophenyl acetate (C8H4Cl4O2) | RHZFLXLMXCNOHX-UHFFFAOYSA-N | CC(=O)OC1=C(C(=C(C=C1Cl)Cl)Cl)Cl | 350 | 50 | 2 | 2 |
| Benzene, pentachloroethoxy- (C8H5Cl5O) | YXNDWTIYDLVODL-UHFFFAOYSA-N | CCOC1=C(C(=C(C(=C1Cl)Cl)Cl)Cl)Cl | 1005 | 0 | 1 | 2 |
| Ethanol, 2,2'-((m-chlorophenyl)imino)di- (C10H14ClNO2) | MVQUJEUCFOGFJU-UHFFFAOYSA-N | C1=CC(=CC(=C1)Cl)N(CCO)CCO | 1228 | 51 | 1 | 2 |
| 4-Chloro-3-nitrophenylacetamide (C8H7ClN2O3) | FPLQCHARENUDSW-UHFFFAOYSA-N | C1=CC(=C(C=C1CC(=O)N)[N+](=O)[O-])Cl | 1268 | 0 | 1 | 2 |
| Ethanol, 2-chloro-, 4-methylbenzenesulfonate (C9H11ClO3S) | ZXNMIUJDTOMBPV-UHFFFAOYSA-N | CC1=CC=C(C=C1)S(=O)(=O)OCCCl | 1519 | 0 | 1 | 2 |
| Benzenesulfonic acid, 2-amino-5-chloro-4-ethyl- (C8H10ClNO3S) | DJOIZOKQHNHZPN-UHFFFAOYSA-N | CCC1=CC(=C(C=C1Cl)S(=O)(=O)O)N | 7679 | 0 | 1 | 2 |
| 4-Chlorophenylsulfonylacetone (C9H9ClO3S) | BRDBHPZILGTBFY-UHFFFAOYSA-N | CC(=O)CS(=O)(=O)C1=CC=C(C=C1)Cl | 277 | 0 | 2 | 2 |
| Propanamide, N-(4-methoxyphenyl)-3-chloro- (C10H12ClNO2) | ZVNNQFDBJXKWOE-UHFFFAOYSA-N | COC1=CC=C(C=C1)NC(=O)CCCl | 5185 | 110 | 1 | 1 |
| 2-Acetamido-6-chlorobenzoic acid (C9H8ClNO3) | VFHSJTHAMJFUCK-UHFFFAOYSA-N | CC(=O)NC1=C(C(=CC=C1)Cl)C(=O)O | 2889 | 0 | 1 | 2 |
| Propanamide, N-(4-methoxyphenyl)-2-chloro- (C10H12ClNO2) | DGAWNIKUBVWNHS-UHFFFAOYSA-N | CC(C(=O)NC1=CC=C(C=C1)OC)Cl | 9038 | 781 | 1 | 1 |
| MCPA Methyl ester (C10H11ClO3) | VWERIRLJUWTNDA-UHFFFAOYSA-N | CC1=C(C=CC(=C1)Cl)OCC(=O)OC | 7873 | 177 | 1 | 1 |
| 2-Chloropropionic acid, 4-methoxyphenyl ester (C10H11ClO3) | LHPTYGDRPPSKNI-UHFFFAOYSA-N | CC(C(=O)OC1=CC=C(C=C1)OC)Cl | 1441 | 0 | 1 | 2 |
| Isopropyl m-chloro carbanilate (C10H12ClNO2) | CWJSHJJYOPWUGX-UHFFFAOYSA-N | CC(C)OC(=O)NC1=CC(=CC=C1)Cl | 3094 | 11 | 1 | 2 |
| Ethyl 3-Chloro-4-methylthiolcarbanilate (C10H12ClNOS) | PCXVSKLBAGHKHW-UHFFFAOYSA-N | CCSC(=O)NC1=CC(=C(C=C1)C)Cl | 1075 | 0 | 1 | 2 |
| Ethyl N-(3-chloro-4-methylphenyl)carbamate (C10H12ClNO2) | KJEXITNRKHKKBH-UHFFFAOYSA-N | CCOC(=O)NC1=CC(=C(C=C1)C)Cl | 3648 | 0 | 1 | 2 |
| Carbanolate (C10H12ClNO2) | QRTXZGIQTYDABO-UHFFFAOYSA-N | CC1=CC(=C(C=C1C)Cl)OC(=O)NC | 500 | 0 | 2 | 2 |
| Dichloroacetic acid, 4-cyanophenyl ester (C9H5Cl2NO2) | FZXOVVSGYFFISB-UHFFFAOYSA-N | C1=CC(=CC=C1C#N)OC(=O)C(Cl)Cl | 1742 | 0 | 1 | 2 |
| Dichlorprop (C9H8Cl2O3) | MZHCENGPTKEIGP-UHFFFAOYSA-N | CC(C(=O)O)OC1=C(C=C(C=C1)Cl)Cl | 3509 | 10 | 1 | 2 |
| Benzyl 2,2,2-trichloroacetimidate (C9H8Cl3NO) | HUZCTWYDQIQZPM-UHFFFAOYSA-N | C1=CC=C(C=C1)COC(=N)C(Cl)(Cl)Cl | 120 | 30 | 2 | 2 |
| Benzoic acid, 2,2,2-trichloroethyl ester (C9H7Cl3O2) | WGXLSZQUOYSATB-UHFFFAOYSA-N | C1=CC=C(C=C1)C(=O)OCC(Cl)(Cl)Cl | 611 | 220 | 2 | 1 |
| Acetamide, N-(3-methylphenyl)-2,2,2-trichloro- (C9H8Cl3NO) | WJTMDWHORSRZQN-UHFFFAOYSA-N | CC1=CC(=CC=C1)NC(=O)C(Cl)(Cl)Cl | 2322 | 320 | 1 | 1 |
| Trichloroacetic acid, 3-methylphenyl ester (C9H7Cl3O2) | WEXLTVMJHWNNTN-UHFFFAOYSA-N | CC1=CC(=CC=C1)OC(=O)C(Cl)(Cl)Cl | 2642 | 100 | 1 | 2 |
| 2-Chloropropionic acid, 3,4-dichlorophenyl ester (C9H7Cl3O2) | YFMJDVKPXPSLOQ-UHFFFAOYSA-N | CC(C(=O)OC1=CC(=C(C=C1)Cl)Cl)Cl | 1281 | 0 | 1 | 2 |
| Chlorothalonil (C8Cl4N2) | CRQQGFGUEAVUIL-UHFFFAOYSA-N | C(#N)C1=C(C(=C(C(=C1Cl)Cl)Cl)C#N)Cl | 8149 | 1099 | 1 | 1 |
| 1,2-Benzenedicarbonitrile, 3,4,5,6-tetrachloro- (C8Cl4N2) | OQHXZZGZASQSOB-UHFFFAOYSA-N | C(#N)C1=C(C(=C(C(=C1Cl)Cl)Cl)Cl)C#N | 7954 | 1228 | 1 | 1 |
| Benzene, 1,2,4,5-tetrachloro-3,6-bis(chloromethyl)- (C8H4Cl6) | IYGDLOMSJZQSGY-UHFFFAOYSA-N | C(C1=C(C(=C(C(=C1Cl)Cl)CCl)Cl)Cl)Cl | 1799 | 6099 | 1 | 1 |
| Benzene, 1,4-bis(trichloromethyl)- (C8H4Cl6) | OTEKOJQFKOIXMU-UHFFFAOYSA-N | C1=CC(=CC=C1C(Cl)(Cl)Cl)C(Cl)(Cl)Cl | 389 | 6249 | 2 | 1 |
| 4-(p-Chlorophenoxy)butyric acid (C10H11ClO3) | SIYAHZSHQIPQLY-UHFFFAOYSA-N | C1=CC(=CC=C1OCCCC(=O)O)Cl | 516 | 0 | 2 | 2 |
| p-([2-Chloroethyl]ethylamino)benzaldehyde (C11H14ClNO) | MFUFJCIFNHFEDW-UHFFFAOYSA-N | CCN(CCCl)C1=CC=C(C=C1)C=O | 1250 | 184 | 1 | 1 |
| Butanamide, N-(4-chlorophenyl)-3-oxo- (C10H10ClNO2) | JMRJWEJJUKUBEA-UHFFFAOYSA-N | CC(=O)CC(=O)NC1=CC=C(C=C1)Cl | 3289 | 0 | 1 | 2 |
| Propanoic acid, 2-methyl, 2-(4-chlorophenyloxy) (C10H11ClO3) | TXCGAZHTZHNUAI-UHFFFAOYSA-N | CC(C)(C(=O)O)OC1=CC=C(C=C1)Cl | 650 | 0 | 2 | 2 |
| Propanoic acid, 2-(4-chloro-2-methylphenoxy)- (C9H11ClO3) | WNTGYJSOUMFZEP-UHFFFAOYSA-N | CC1=C(C=CC(=C1)Cl)OC(C)C(=O)O | 3064 | 0 | 1 | 2 |
| propachlor (C11H14ClNO) | MFOUDYKPLGXPGO-UHFFFAOYSA-N | CC(C)N(C1=CC=CC=C1)C(=O)CCl | 810 | 3712 | 1 | 1 |
| Chloroacetamide, N-ethyl-N-(3-methylphenyl)- (C11H14ClNO) | HDVSUKKVAMUTGV-UHFFFAOYSA-N | CCN(C1=CC=CC(=C1)C)C(=O)CCl | 4664 | 5355 | 1 | 1 |
| 4-Chlorobutyric acid, 4-chlorophenyl ester (C10H10Cl2O2) | BZXFHJFAMAYMNE-UHFFFAOYSA-N | C1=CC(=CC=C1OC(=O)CCCCl)Cl | 791 | 0 | 2 | 2 |
| Chlorfenprop-methyl (C10H10Cl2O2) | YJKIALIXRCSISK-UHFFFAOYSA-N | COC(=O)C(CC1=CC=C(C=C1)Cl)Cl | 80 | 3750 | 2 | 1 |
| Butyric acid, 3,4-dichlorophenyl ester (C10H10Cl2O2) | GVUWGECOKPZWDC-UHFFFAOYSA-N | CCCC(=O)OC1=CC(=C(C=C1)Cl)Cl | 1121 | 0 | 1 | 2 |
| Dichloroacetic acid, 3,5-dimethylphenyl ester (C10H10Cl2O2) | NGJFMOALFGIQLY-UHFFFAOYSA-N | CC1=CC(=CC(=C1)OC(=O)C(Cl)Cl)C | 1742 | 0 | 1 | 2 |
| 2-Methylpropionic acid, 3,4-dichlorophenyl ester (C10H10Cl2O2) | ZCWLUOCBBNEMPS-UHFFFAOYSA-N | CC(C)C(=O)OC1=CC(=C(C=C1)Cl)Cl | 1251 | 0 | 1 | 2 |
| 2-Chlorobenzoic acid, but-3-yn-2-yl ester (C11H9ClO2) | NEARZUNSJNFJKJ-UHFFFAOYSA-N | CC(C#C)OC(=O)C1=CC=CC=C1Cl | 721 | 120 | 2 | 1 |
| 4-Chlorobenzoic acid, but-3-yn-2-yl ester (C11H9ClO2) | LRAXWWFDHMSJAF-UHFFFAOYSA-N | CC(C#C)OC(=O)C1=CC=C(C=C1)Cl | 1091 | 90 | 1 | 2 |
| Cyclophosphamide (C7H15Cl2N2O2P) | CMSMOCZEIVJLDB-UHFFFAOYSA-N | C1CNP(=O)(OC1)N(CCCl)CCCl | 189 | 159 | 2 | 1 |
| 4-Chlorobutyl benzoate (C11H13ClO2) | XFFQVVCNZAYQSJ-UHFFFAOYSA-N | C1=CC=C(C=C1)C(=O)OCCCCCl | 259 | 949 | 2 | 1 |
| Baclofen (C10H12ClNO2) | KPYSYYIEGFHWSV-UHFFFAOYSA-N | C1=CC(=CC=C1C(CC(=O)O)CN)Cl | 0 | 0 | 2 | 2 |
| Pentanamide, N-(3-chlorophenyl)- (C11H14ClNO) | LOQKNLXBLMIZPG-UHFFFAOYSA-N | CCCCC(=O)NC1=CC(=CC=C1)Cl | 1271 | 0 | 1 | 2 |
| Butyl 4-chlorobenzoate (C11H13ClO2) | PBPVJPWUWLGOEC-UHFFFAOYSA-N | CCCCOC(=O)C1=CC=C(C=C1)Cl | 90 | 0 | 2 | 2 |
| Valeric acid, 4-chlorophenyl ester (C11H13ClO2) | BEZFOOVQBIWCMU-UHFFFAOYSA-N | CCCCC(=O)OC1=CC=C(C=C1)Cl | 1822 | 0 | 1 | 2 |
| Clorprenaline (C11H16ClNO) | SSMSBSWKLKKXGG-UHFFFAOYSA-N | CC(C)NCC(C1=CC=CC=C1Cl)O | 0 | 0 | 2 | 2 |
| Benzoic acid, 4-chloro, isobutyl ester (C11H13ClO2) | BWLDEQAGSZRTIV-UHFFFAOYSA-N | CC(C)COC(=O)C1=CC=C(C=C1)Cl | 40 | 0 | 2 | 2 |
| Benzoic acid, 2-chloro, 2-methylpropyl ester (C11H13ClO2) | BESGEXPDWNNBJA-UHFFFAOYSA-N | CC(C)COC(=O)C1=CC=CC=C1Cl | 0 | 0 | 2 | 2 |
| Benzoic acid, 4-chloro, 1-methylpropyl ester (C11H13ClO2) | XQYCLIPXEPYRON-UHFFFAOYSA-N | CCC(C)OC(=O)C1=CC=C(C=C1)Cl | 50 | 60 | 2 | 2 |
| 2-Chloropropionic acid, 3,5-dimethylphenyl ester (C11H13ClO2) | SJELLOBJQJSXPZ-UHFFFAOYSA-N | CC1=CC(=CC(=C1)OC(=O)C(C)Cl)C | 921 | 0 | 1 | 2 |
| Propanamide, N-(3-chlorophenyl)-2,2-dimethyl- (C11H14ClNO) | OGOQXGKPTWSHPS-UHFFFAOYSA-N | CC(C)(C)C(=O)NC1=CC(=CC=C1)Cl | 5485 | 0 | 1 | 2 |
| 2,2-Dimethylpropanoic acid, 4-chlorophenyl ester (C11H13ClO2) | OYQBAUOEQCWQJN-UHFFFAOYSA-N | CC(C)(C)C(=O)OC1=CC=C(C=C1)Cl | 1291 | 0 | 1 | 2 |
| Mefenorex (C12H18ClN) | XXVROGAVTTXONC-UHFFFAOYSA-N | CC(CC1=CC=CC=C1)NCCCCl | 10 | 0 | 2 | 2 |
| 2-(p-Chlorophenyl)-3-methylbutyric acid (C11H13ClO2) | VTJMSIIXXKNIDJ-UHFFFAOYSA-N | CC(C)C(C1=CC=C(C=C1)Cl)C(=O)O | 1854 | 0 | 1 | 2 |
| 1-Propanone, 2-chloro-1-(4-ethylphenyl)-2-methyl- (C12H15ClO) | JRCYBVHLGNUGDA-UHFFFAOYSA-N | CCC1=CC=C(C=C1)C(=O)C(C)(C)Cl | 52 | 24 | 2 | 2 |
| 1-Propanone, 2-chloro-1-(2,5-dimethylphenyl)-2-methyl- (C12H15ClO) | VXXAHSALZMCFQS-UHFFFAOYSA-N | CC1=CC(=C(C=C1)C)C(=O)C(C)(C)Cl | 70 | 100 | 2 | 2 |
| 1-Propanone, 2-chloro-1-(2,4-dimethylphenyl)-2-methyl- (C12H15ClO) | FRXUQBIZUYXNKL-UHFFFAOYSA-N | CC1=CC(=C(C=C1)C(=O)C(C)(C)Cl)C | 30 | 20 | 2 | 2 |
| 1,2,4,5-Tetramethyl-3,6-bis(chloromethyl)benzene (C12H16Cl2) | PGFAKOSRZYDFLR-UHFFFAOYSA-N | CC1=C(C(=C(C(=C1CCl)C)C)CCl)C | 1630 | 9999 | 1 | 1 |
| 7-Phenyl-1-heptyl chloride (C13H19Cl) | PNUBJDFTMKBZJK-UHFFFAOYSA-N | C1=CC=C(C=C1)CCCCCCCCl | 794 | 7 | 2 | 2 |
| Pentanamide, N-tetrahydrofurfuryl-5-chloro- (C10H18ClNO2) | LGSZKARSGCPNGG-UHFFFAOYSA-N | C1CC(OC1)CNC(=O)CCCCCl | 0 | 190 | 2 | 1 |
| 2-Chlorobenzoic acid, 3-chloroprop-2-enyl ester (C10H8Cl2O2) | GIMRXOTXLJWYLM-ZZXKWVIFSA-N | C1=CC=C(C(=C1)C(=O)OC/C=C/Cl)Cl | 180 | 1441 | 2 | 1 |
| 4-Chlorobenzoic acid, 3-chloroprop-2-enyl ester (C10H8Cl2O2) | KYSAXPAUQIXXNR-LZCJLJQNSA-N | C1=CC(=CC=C1C(=O)OC/C=C/Cl)Cl | 50 | 1832 | 2 | 1 |
| 2-Propenamide, N-(3,4-dichlorophenyl)-2-methyl- (C10H9Cl2NO) | VCBRBUKGTWLJOB-UHFFFAOYSA-N | CC(=C)C(=O)NC1=CC(=C(C=C1)Cl)Cl | 1111 | 270 | 1 | 1 |
| phenylacetic acid, 3-chloroprop-2-enyl ester (C11H11ClO2) | PBULRLQIJUOCDV-QPJJXVBHSA-N | C1=CC=C(C=C1)CC(=O)OC/C=C/Cl | 911 | 1041 | 1 | 1 |
| m-toluylic acid, 3-chloroprop-2-enyl ester (C11H11ClO2) | MBSMOYXJNIUOJL-ZZXKWVIFSA-N | CC1=CC=CC(=C1)C(=O)OC/C=C/Cl | 200 | 2853 | 2 | 1 |
| p-toluylic acid, 3-chloroprop-2-enyl ester (C11H11ClO2) | AKHNPGGATKIWFI-FARCUNLSSA-N | CC1=CC=C(C=C1)C(=O)OC/C=C/Cl | 130 | 2502 | 2 | 1 |
| o-Toluylic acid, 3-chloropen-2-enyl ester (C11H11ClO2) | MKXZDOUAKCYRJS-QPJJXVBHSA-N | CC1=CC=CC=C1C(=O)OC/C=C/Cl | 280 | 2482 | 2 | 1 |
| 2-Butenamide, N-(3-chlorophenyl)-3-methyl- (C11H12ClNO) | DYNVGFUQFOJQAY-UHFFFAOYSA-N | CC(=CC(=O)NC1=CC(=CC=C1)Cl)C | 2272 | 0 | 1 | 2 |
| 3-Methylbut-2-enoic acid, 4-chlorophenyl ester (C11H11ClO2) | CFKIJEHSEFGFHA-UHFFFAOYSA-N | CC(=CC(=O)OC1=CC=C(C=C1)Cl)C | 280 | 0 | 2 | 2 |
| Terbacil (C8H13ClN2O2) | NBQCNZYJJMBDKY-UHFFFAOYSA-N | CC1=C(C(=O)N(C(=O)N1)C(C)(C)C)Cl | 379 | 0 | 2 | 2 |
| Cyclopropanecarboxylic acid, 3-(2,2-dichloroethenyl)-2,2-dimethyl-, ethyl ester (C10H14Cl2O2) | QPTWKDNRYCGMJM-UHFFFAOYSA-N | CCOC(=O)C1C(C1(C)C)C=C(Cl)Cl | 379 | 2879 | 2 | 1 |
| 3-cyclopentylpropionic acid, 3-chloroprop-2-enyl ester (C11H17ClO2) | QXWNSXGSUGCIHO-FPYGCLRLSA-N | C1CCC(C1)CCC(=O)OC/C=C/Cl | 190 | 2983 | 2 | 1 |
| 1,2-Dichloro-4,5-dicyano-p-benzoquinone (C8Cl2N2O2) | HZNVUJQVZSTENZ-UHFFFAOYSA-N | C(#N)C1=C(C(=O)C(=C(C1=O)Cl)Cl)C#N | 5189 | 889 | 1 | 1 |
| Naphthalene, 1,2,3,4-tetrachloro- (C10H4Cl4) | NAQWICRLNQSPPW-UHFFFAOYSA-N | C1=CC=C2C(=C1)C(=C(C(=C2Cl)Cl)Cl)Cl | 8029 | 1410 | 1 | 1 |
| Naphthalene, 1,3,5,7-tetrachloro- (C10H4Cl4) | OTTCXKPQKOLSJN-UHFFFAOYSA-N | C1=C(C=C(C2=CC(=CC(=C21)Cl)Cl)Cl)Cl | 7837 | 896 | 1 | 1 |
| Naphthalene, 1,4,6,7-tetrachloro- (C10H4Cl4) | VJZRCIYSYVGDMU-UHFFFAOYSA-N | C1=CC(=C2C=C(C(=CC2=C1Cl)Cl)Cl)Cl | 7713 | 886 | 1 | 1 |
| 3-Chlorodiphenylamine (C12H10ClN) | OHHIBZKYXJDQEU-UHFFFAOYSA-N | C1=CC=C(C=C1)NC2=CC(=CC=C2)Cl | 9999 | 1559 | 1 | 1 |
| Benzene, 1-chloro-4-phenoxy- (C12H9ClO) | PGPNJCAMHOJTEF-UHFFFAOYSA-N | C1=CC=C(C=C1)OC2=CC=C(C=C2)Cl | 9999 | 741 | 1 | 1 |
| Clonidine (C9H9Cl2N3) | GJSURZIOUXUGAL-UHFFFAOYSA-N | C1CN=C(N1)NC2=C(C=CC=C2Cl)Cl | 9999 | 2819 | 1 | 1 |
| chloro quinaldol (C10H7Cl2NO) | GPTXWRGISTZRIO-UHFFFAOYSA-N | CC1=NC2=C(C=C1)C(=CC(=C2O)Cl)Cl | 9999 | 360 | 1 | 1 |
| [1,1'-Biphenyl-3-ol], 6-chloro- (C12H9ClO) | UGRROGFBORDDFR-UHFFFAOYSA-N | C1=CC=C(C=C1)C2=C(C=CC(=C2)O)Cl | 9999 | 77 | 1 | 2 |
| [1,1'-Biphenyl-4-ol], 2-chloro- (C12H9ClO) | MXORDJXBRHNWBE-UHFFFAOYSA-N | C1=CC=C(C=C1)C2=C(C=C(C=C2)O)Cl | 9999 | 470 | 1 | 1 |
| [1,1'-Biphenyl]-4-ol, 4'-chloro- (C12H9ClO) | ICVFJPSNAUMFCW-UHFFFAOYSA-N | C1=CC(=CC=C1C2=CC=C(C=C2)Cl)O | 9999 | 289 | 1 | 1 |
| [1,1'-Biphenyl]-2-ol, 5-chloro- (C12H9ClO) | DSQWWSVOIGUHAE-UHFFFAOYSA-N | C1=CC=C(C=C1)C2=C(C=CC(=C2)Cl)O | 9999 | 1254 | 1 | 1 |
| [1,1'-Biphenyl]-2-ol, 3-chloro- (C12H9ClO) | XBILVINOJVKEHG-UHFFFAOYSA-N | C1=CC=C(C=C1)C2=C(C(=CC=C2)Cl)O | 9999 | 2267 | 1 | 1 |
| [1,1'-Biphenyl]-4-ol, 3-chloro- (C12H9ClO) | BZWMYDJJDBFAPE-UHFFFAOYSA-N | C1=CC=C(C=C1)C2=CC(=C(C=C2)O)Cl | 9999 | 229 | 1 | 1 |
| PCB 4 (C12H8Cl2) | JAYCNKDKIKZTAF-UHFFFAOYSA-N | C1=CC=C(C(=C1)C2=CC=CC=C2Cl)Cl | 5909 | 4250 | 1 | 1 |
| PCB 5 (C12H8Cl2) | XOMKZKJEJBZBJJ-UHFFFAOYSA-N | C1=CC=C(C=C1)C2=C(C(=CC=C2)Cl)Cl | 9999 | 270 | 1 | 1 |
| PCB 11 (C12H8Cl2) | KTXUOWUHFLBZPW-UHFFFAOYSA-N | C1=CC(=CC(=C1)Cl)C2=CC(=CC=C2)Cl | 9999 | 170 | 1 | 1 |
| PCB 15 (C12H8Cl2) | YTBRNEUEFCNVHC-UHFFFAOYSA-N | C1=CC(=CC=C1C2=CC=C(C=C2)Cl)Cl | 9999 | 202 | 1 | 1 |
| PCB 6 (C12H8Cl2) | ZHBBDTRJIVXKEX-UHFFFAOYSA-N | C1=CC=C(C(=C1)C2=CC(=CC=C2)Cl)Cl | 9999 | 359 | 1 | 1 |
| PCB 13 (C12H8Cl2) | CJDNEKOMKXLSBN-UHFFFAOYSA-N | C1=CC(=CC(=C1)Cl)C2=CC=C(C=C2)Cl | 9999 | 227 | 1 | 1 |
| PCB 12 (C12H8Cl2) | ZGHQUYZPMWMLBM-UHFFFAOYSA-N | C1=CC=C(C=C1)C2=CC(=C(C=C2)Cl)Cl | 9802 | 241 | 1 | 1 |
| PCB 10 (C12H8Cl2) | IYZWUWBAFUBNCH-UHFFFAOYSA-N | C1=CC=C(C=C1)C2=C(C=CC=C2Cl)Cl | 8487 | 563 | 1 | 1 |
| PCB 7 (C12H8Cl2) | WEJZHZJJXPXXMU-UHFFFAOYSA-N | C1=CC=C(C=C1)C2=C(C=C(C=C2)Cl)Cl | 9999 | 220 | 1 | 1 |
| PCB 9 (C12H8Cl2) | KKQWHYGECTYFIA-UHFFFAOYSA-N | C1=CC=C(C=C1)C2=C(C=CC(=C2)Cl)Cl | 7705 | 329 | 1 | 1 |
| PCB 14 (C12H8Cl2) | QHZSDTDMQZPUKC-UHFFFAOYSA-N | C1=CC=C(C=C1)C2=CC(=CC(=C2)Cl)Cl | 9234 | 266 | 1 | 1 |
| PCB 8 (C12H8Cl2) | UFNIBRDIUNVOMX-UHFFFAOYSA-N | C1=CC=C(C(=C1)C2=CC=C(C=C2)Cl)Cl | 8497 | 256 | 1 | 1 |
| Phthalide (C8H2Cl4O2) | NMWKWBPNKPGATC-UHFFFAOYSA-N | C1C2=C(C(=C(C(=C2Cl)Cl)Cl)Cl)C(=O)O1 | 1960 | 30 | 1 | 2 |
| 2-[(4-Chlorophenyl)methyl]pyridine (C12H10ClN) | XSVWMIMFDMJQRL-UHFFFAOYSA-N | C1=CC=NC(=C1)CC2=CC=C(C=C2)Cl | 3173 | 1922 | 1 | 1 |
| 4-(para-Chlorobenzyl)-pyridine (C12H10ClN) | OHKBVLWPESSWKC-UHFFFAOYSA-N | C1=CC(=CC=C1CC2=CC=NC=C2)Cl | 6836 | 9999 | 1 | 1 |
| 1,1'-Biphenyl, 4-(chloromethyl)- (C13H11Cl) | HLQZCRVEEQKNMS-UHFFFAOYSA-N | C1=CC=C(C=C1)C2=CC=C(C=C2)CCl | 2709 | 9999 | 1 | 1 |
| Diphenylmethane, 2-chloro (C13H11Cl) | IKKSPFNZXBWDQA-UHFFFAOYSA-N | C1=CC=C(C=C1)CC2=CC=CC=C2Cl | 3744 | 9999 | 1 | 1 |
| 5-Isobenzofurancarbonyl chloride, 1,3-dihydro-1,3-dioxo- (C9H3ClO4) | NJMOHBDCGXJLNJ-UHFFFAOYSA-N | C1=CC2=C(C=C1C(=O)Cl)C(=O)OC2=O | 0 | 9999 | 2 | 1 |
| 1-(3-Chlorophenyl)-3-methyl-2-pyrazolin-5-one (C10H9ClN2O) | RIOMUJXIGYZENC-UHFFFAOYSA-N | CC1=NN(C(=O)C1)C2=CC(=CC=C2)Cl | 9999 | 0 | 1 | 2 |
| Oxirane, 2,2-dicyano-3-(2-chlorophenyl) (C10H5ClN2O) | MVPVSDDVDXPOOP-UHFFFAOYSA-N | C1=CC=C(C(=C1)C2C(O2)(C#N)C#N)Cl | 6005 | 2092 | 1 | 1 |
| Cypromid (C10H9Cl2NO) | PLQDLOBGKJCDSZ-UHFFFAOYSA-N | C1CC1C(=O)NC2=CC(=C(C=C2)Cl)Cl | 1531 | 20 | 1 | 2 |
| Cyclopropanecarboxylic acid, 3,4-dichlorophenyl ester (C10H8Cl2O2) | IPQDLGFOBIGXHR-UHFFFAOYSA-N | C1CC1C(=O)OC2=CC(=C(C=C2)Cl)Cl | 300 | 0 | 2 | 2 |
| 4-(para-Chlorophenyl)-4-hydroxypiperidine (C11H14ClNO) | LZAYOZUFUAMFLD-UHFFFAOYSA-N | C1CNCCC1(C2=CC=C(C=C2)Cl)O | 1048 | 0 | 1 | 2 |
| 2-chlorobenzoic acid, cyclobutyl ester (C11H11ClO2) | CDMUMZPSCNDTJY-UHFFFAOYSA-N | C1CC(C1)OC(=O)C2=CC=CC=C2Cl | 0 | 60 | 2 | 2 |
| Cyclobutanecarboxamide, N-(3-chlorophenyl)- (C11H12ClNO) | NKTPEKDVZVAYNU-UHFFFAOYSA-N | C1CC(C1)C(=O)NC2=CC(=CC=C2)Cl | 2813 | 0 | 1 | 2 |
| Cyclobutanecarboxylic acid, 4-chlorophenyl ester (C11H11ClO2) | KPTHQNDVUAFMRH-UHFFFAOYSA-N | C1CC(C1)C(=O)OC2=CC=C(C=C2)Cl | 1401 | 0 | 1 | 2 |
| 4-Chloro-2-cyclohexylphenol (C12H15ClO) | XRUHXAQEOJDPEG-UHFFFAOYSA-N | C1CCC(CC1)C2=C(C=CC(=C2)Cl)O | 5075 | 771 | 1 | 1 |
| Phenol, 2-chloro-4-cyclohexyl- (C12H15ClO) | VVMQAIJCWNJCMK-UHFFFAOYSA-N | C1CCC(CC1)C2=CC(=C(C=C2)O)Cl | 8117 | 530 | 1 | 1 |
| o-Chlorophenyl cyclopentyl ketone (C12H13ClO) | QIJMMRNZBJHXRI-UHFFFAOYSA-N | C1CCC(C1)C(=O)C2=CC=CC=C2Cl | 489 | 491 | 2 | 1 |
| N-(2-Chlorophenyl)-maleimide (C10H6ClNO2) | KPQOXMCRYWDRSB-UHFFFAOYSA-N | C1=CC=C(C(=C1)N2C(=O)C=CC2=O)Cl | 2029 | 9999 | 1 | 1 |
| 5-Chlorobenzo[b]thiophene-3-acetic acid (C10H7ClO2S) | QQKKTOPRRGBBCT-UHFFFAOYSA-N | C1=CC2=C(C=C1Cl)C(=CS2)CC(=O)O | 3494 | 0 | 1 | 2 |
| ACN (C10H6ClNO2) | OBLNWSCLAYSJJR-UHFFFAOYSA-N | C1=CC=C2C(=C1)C(=O)C(=C(C2=O)Cl)N | 8778 | 9999 | 1 | 1 |
| Dichlone (C10H4Cl2O2) | SVPKNMBRVBMTLB-UHFFFAOYSA-N | C1=CC=C2C(=C1)C(=O)C(=C(C2=O)Cl)Cl | 7639 | 9999 | 1 | 1 |
| 2-Chloro-3-methyl-1,4-naphthoquinone (C11H7ClO2) | WYIOQBYLZGEHCV-UHFFFAOYSA-N | CC1=C(C(=O)C2=CC=CC=C2C1=O)Cl | 9850 | 9999 | 1 | 1 |
| 2-Norbornene, 1,2,3,4,5,7,7-heptachloro (C7H3Cl7) | FCMVPUGRXPEIQX-UHFFFAOYSA-N | C1C(C2(C(=C(C1(C2(Cl)Cl)Cl)Cl)Cl)Cl)Cl | 350 | 1450 | 2 | 1 |
| 2-Chlorofluorene (C13H9Cl) | FCPAQNZCCWBDSY-UHFFFAOYSA-N | C1C2=CC=CC=C2C3=C1C=C(C=C3)Cl | 2519 | 9999 | 1 | 1 |
| Malonic acid, di(2,2-dichloroethyl) ester (C7H8Cl4O4) | RYOQIENTSXLUNE-UHFFFAOYSA-N | C(C(Cl)Cl)OC(=O)CC(=O)OCC(Cl)Cl | 0 | 20 | 2 | 2 |
| Octachlorodipropyl ether (C6H6Cl8O) | LNJXZKBHJZAIKQ-UHFFFAOYSA-N | C(C(C(Cl)(Cl)Cl)Cl)OCC(C(Cl)(Cl)Cl)Cl | 0 | 0 | 2 | 2 |
| Succinic acid, ethyl 2,2,2-trichloroethyl ester (C8H11Cl3O4) | KTANCHZRFSHKEZ-UHFFFAOYSA-N | CCOC(=O)CCC(=O)OCC(Cl)(Cl)Cl | 0 | 0 | 2 | 2 |
| Succinic acid, 2,2-dichloroethyl propyl ester (C9H14Cl2O4) | FJMPKWLUBAMQEB-UHFFFAOYSA-N | CCCOC(=O)CCC(=O)OCC(Cl)Cl | 0 | 0 | 2 | 2 |
| Malonic acid, butyl 2,2-dichloroethyl ester (C9H14Cl2O4) | OXMJTLDSJUOPRS-UHFFFAOYSA-N | CCCCOC(=O)CC(=O)OCC(Cl)Cl | 0 | 60 | 2 | 2 |
| Malonic acid, di(2-chloropropyl) ester (C9H14Cl2O4) | HOHKYAJHKKSVSS-UHFFFAOYSA-N | CC(COC(=O)CC(=O)OCC(C)Cl)Cl | 0 | 20 | 2 | 2 |
| Malonic acid, 2,2-dichloroethyl isobutyl ester (C9H14Cl2O4) | ZAUJNOGWWYTMRR-UHFFFAOYSA-N | CC(C)COC(=O)CC(=O)OCC(Cl)Cl | 0 | 30 | 2 | 2 |
| Trichloroacetamide, N,N-dibutyl- (C10H18Cl3NO) | XAPJKGWLPPCTDF-UHFFFAOYSA-N | CCCCN(CCCC)C(=O)C(Cl)(Cl)Cl | 0 | 811 | 2 | 1 |
| Octyl trichloroacetate (C10H17Cl3O2) | GNKXBEJZRKOBKI-UHFFFAOYSA-N | CCCCCCCCOC(=O)C(Cl)(Cl)Cl | 0 | 0 | 2 | 2 |
| Dichloroacetic acid, 2-methyloct-5-yn-4-yl ester (C11H16Cl2O2) | AHCSRIQXLAZANZ-UHFFFAOYSA-N | CCC#CC(CC(C)C)OC(=O)C(Cl)Cl | 0 | 1902 | 2 | 1 |
| Succinic acid, monochloride, 3-heptyl ester (C11H19ClO3) | TTZXRGGNPFEDIS-UHFFFAOYSA-N | CCCCC(CC)OC(=O)CCC(=O)Cl | 0 | 0 | 2 | 2 |
| Succinic acid, monochloride, 4-heptyl ester (C11H19ClO3) | GMHUSZRKKXFXSG-UHFFFAOYSA-N | CCCC(CCC)OC(=O)CCC(=O)Cl | 0 | 0 | 2 | 2 |
| Succinic acid, monochloride, 2,4-dimethylpent-3-yl ester (C11H19ClO3) | LQAKEJMUFGKWGB-UHFFFAOYSA-N | CC(C)C(C(C)C)OC(=O)CCC(=O)Cl | 0 | 0 | 2 | 2 |
| 10-Chlorodecanoic acid, chloromethyl ester (C11H20Cl2O2) | ZOBAMFUTBJXGTK-UHFFFAOYSA-N | C(CCCCC(=O)OCCl)CCCCCl | 0 | 0 | 2 | 2 |
| 9-Chlorodecanoic acid, chloromethyl ester (C11H20Cl2O2) | KPSTVHFMJIDEGU-UHFFFAOYSA-N | CC(CCCCCCCC(=O)OCCl)Cl | 0 | 0 | 2 | 2 |
| 8-Chlorodecanoic acid, chloromethyl ester (C11H20Cl2O2) | ZMBCSGUZVMJJFT-UHFFFAOYSA-N | CCC(CCCCCCC(=O)OCCl)Cl | 0 | 0 | 2 | 2 |
| 7-Chlorodecanoic acid, chloromethyl ester (C11H20Cl2O2) | WOXCLOSQVCNINZ-UHFFFAOYSA-N | CCCC(CCCCCC(=O)OCCl)Cl | 0 | 0 | 2 | 2 |
| 2-Chlorodecanoic acid, chloromethyl ester (C11H20Cl2O2) | ZBWKSSOXFUXWPK-UHFFFAOYSA-N | CCCCCCCCC(C(=O)OCCl)Cl | 0 | 0 | 2 | 2 |
| 4-Chlorodecanoic acid, chloromethyl ester (C11H20Cl2O2) | LDNMSCDSUKAAMZ-UHFFFAOYSA-N | CCCCCCC(CCC(=O)OCCl)Cl | 0 | 0 | 2 | 2 |
| 5-Chlorodecanoic acid, chloromethyl ester (C11H20Cl2O2) | XIOMXKGHIDUICH-UHFFFAOYSA-N | CCCCCC(CCCC(=O)OCCl)Cl | 0 | 0 | 2 | 2 |
| Nonyl dichloroacetate (C11H20Cl2O2) | XKMMRMJBZDCIHI-UHFFFAOYSA-N | CCCCCCCCCOC(=O)C(Cl)Cl | 0 | 0 | 2 | 2 |
| 3-Chloropropionic acid, 2-methyloct-5-yn-4-yl ester (C12H19ClO2) | UZTJDQYZKQSKGR-UHFFFAOYSA-N | CCC#CC(CC(C)C)OC(=O)CCCl | 50 | 0 | 2 | 2 |
| 2-Chloroethyl decanoate (C12H23ClO2) | WRIOEJKKXQEEFB-UHFFFAOYSA-N | CCCCCCCCCC(=O)OCCCl | 300 | 0 | 2 | 2 |
| 5-Chlorovaleric acid, heptyl ester (C12H23ClO2) | PSTOZFWGSOOMNV-UHFFFAOYSA-N | CCCCCCCOC(=O)CCCCCl | 0 | 280 | 2 | 1 |
| Decyl chloroacetate (C12H23ClO2) | WLAYVQKPZABXSY-UHFFFAOYSA-N | CCCCCCCCCCOC(=O)CCl | 0 | 0 | 2 | 2 |
| Propanoic acid, 3-chloro, nonyl ester (C12H23ClO2) | TVEXZMIDIHNANR-UHFFFAOYSA-N | CCCCCCCCCOC(=O)CCCl | 0 | 70 | 2 | 2 |
| Chloromethyl undecanoate (C12H23ClO2) | BDZBWJLRACXOET-UHFFFAOYSA-N | CCCCCCCCCCC(=O)OCCl | 1191 | 0 | 1 | 2 |
| Metoxuron (C10H13ClN2O2) | DSRNRYQBBJQVCW-UHFFFAOYSA-N | CN(C)C(=O)NC1=CC(=C(C=C1)OC)Cl | 2312 | 10 | 1 | 2 |
| Linuron (C9H10Cl2N2O2) | XKJMBINCVNINCA-UHFFFAOYSA-N | CN(C(=O)NC1=CC(=C(C=C1)Cl)Cl)OC | 1059 | 0 | 1 | 2 |
| Triclopyr methyl ester (C8H6Cl3NO3) | MNYBZEHWPRTNJY-UHFFFAOYSA-N | COC(=O)COC1=NC(=C(C=C1Cl)Cl)Cl | 2993 | 370 | 1 | 1 |
| 3,5-dinitrobenzoyl chloride (C7H3ClN2O5) | NNOHXABAQAGKRZ-UHFFFAOYSA-N | C1=C(C=C(C=C1[N+](=O)[O-])[N+](=O)[O-])C(=O)Cl | 0 | 9999 | 2 | 1 |
| Acetamide, N-(2,5-dimethoxyphenyl)-2-chloro- (C10H12ClNO3) | BDDQUHPZDJEQRL-UHFFFAOYSA-N | COC1=CC(=C(C=C1)OC)NC(=O)CCl | 7267 | 70 | 1 | 2 |
| 3,4,5-Trimethoxybenzoyl chloride (C10H11ClO4) | BUHYMJLFRZAFBF-UHFFFAOYSA-N | COC1=CC(=CC(=C1OC)OC)C(=O)Cl | 2310 | 9999 | 1 | 1 |
| Acetamide, N-(3-nitrophenyl)-2,2-dichloro- (C8H6Cl2N2O3) | MJSNGWQBPBCMJV-UHFFFAOYSA-N | C1=CC(=CC(=C1)[N+](=O)[O-])NC(=O)C(Cl)Cl | 1421 | 0 | 1 | 2 |
| Dichloroacetic acid, 4-nitrophenyl ester (C8H5Cl2NO4) | PXRGBMOGEXCISN-UHFFFAOYSA-N | C1=CC(=CC=C1[N+](=O)[O-])OC(=O)C(Cl)Cl | 1121 | 0 | 1 | 2 |
| 2',5'-Dichloro-4'-nitroacetanilide (C8H6Cl2N2O3) | YOKGZPNQGIYATO-UHFFFAOYSA-N | CC(=O)NC1=CC(=C(C=C1Cl)[N+](=O)[O-])Cl | 435 | 1414 | 2 | 1 |
| Acetamide, N-(4-methoxyphenyl)-2,2,2-trichloro- (C9H8Cl3NO2) | IFSVHMNTIRKVCD-UHFFFAOYSA-N | COC1=CC=C(C=C1)NC(=O)C(Cl)(Cl)Cl | 2122 | 260 | 1 | 1 |
| 2,4,5-T Methyl ester (C9H7Cl3O3) | JUCNGUOYQGHBJC-UHFFFAOYSA-N | COC(=O)COC1=CC(=C(C=C1Cl)Cl)Cl | 4535 | 9999 | 1 | 1 |
| Trichloroacetic acid, 4-methoxyphenyl ester (C9H7Cl3O3) | MMIXCSCJXHTNAC-UHFFFAOYSA-N | COC1=CC=C(C=C1)OC(=O)C(Cl)(Cl)Cl | 1441 | 110 | 1 | 1 |
| Pentachlorophenyl acetate (C8H3Cl5O2) | RRYATXLRCBOQTJ-UHFFFAOYSA-N | CC(=O)OC1=C(C(=C(C(=C1Cl)Cl)Cl)Cl)Cl | 290 | 50 | 2 | 2 |
| Ethanimidamide, N-[(6-chloro-3-pyridinyl)methyl]-N'-cyano-N-methyl-, (1E)- (C10H11ClN4) | WCXDHFDTOYPNIE-UHFFFAOYSA-N | CC(=NC#N)N(C)CC1=CN=C(C=C1)Cl | 500 | 581 | 2 | 1 |
| Propanamide, N-(3-nitrophenyl)-2-chloro- (C9H9ClN2O3) | DKFBWJTZPPJWFC-UHFFFAOYSA-N | CC(C(=O)NC1=CC(=CC=C1)[N+](=O)[O-])Cl | 5335 | 190 | 1 | 1 |
| Glycine, N-(2-chlorobenzoyl)-, methyl ester (C10H10ClNO3) | NVWHFQJYAXGYIN-UHFFFAOYSA-N | COC(=O)CNC(=O)C1=CC=CC=C1Cl | 801 | 0 | 1 | 2 |
| Acetamide, N-(3-chlorophenyl)-2-acetoxy- (C10H10ClNO3) | ZTOFIGAKFYAJPE-UHFFFAOYSA-N | CC(=O)OCC(=O)NC1=CC(=CC=C1)Cl | 3673 | 0 | 1 | 2 |
| Acetoxyacetic acid, 4-chlorophenyl ester (C10H9ClO4) | XDEZQKYHBPNTOE-UHFFFAOYSA-N | CC(=O)OCC(=O)OC1=CC=C(C=C1)Cl | 561 | 0 | 2 | 2 |
| 1,4-Benzenedicarboxylic acid, 2-chloro-, dimethyl ester (C10H9ClO4) | FUFFCPIFRICMFH-UHFFFAOYSA-N | COC(=O)C1=CC(=C(C=C1)C(=O)OC)Cl | 2355 | 71 | 1 | 2 |
| Dimethyl 5-chloroisophthalate (C10H9ClO4) | CMMPMNSOVLQGMJ-UHFFFAOYSA-N | COC(=O)C1=CC(=CC(=C1)Cl)C(=O)OC | 3358 | 80 | 1 | 2 |
| 2,4-D ethyl ester (C10H10Cl2O3) | JSLBZIVMVVHMDJ-UHFFFAOYSA-N | CCOC(=O)COC1=C(C=C(C=C1)Cl)Cl | 4987 | 2079 | 1 | 1 |
| Dichlorprop, methyl ester (C10H10Cl2O3) | SCHCPDWDIOTCMJ-UHFFFAOYSA-N | CC(C(=O)OC)OC1=C(C=C(C=C1)Cl)Cl | 3669 | 276 | 1 | 1 |
| Chlorthalonil, 4-methoxy (C9H3Cl3N2O) | UXQSHXVXPDJKJU-UHFFFAOYSA-N | COC1=C(C(=C(C(=C1Cl)Cl)C#N)Cl)C#N | 9999 | 0 | 1 | 2 |
| Silvex (C9H7Cl3O3) | ZLSWBLPERHFHIS-UHFFFAOYSA-N | CC(C(=O)O)OC1=CC(=C(C=C1Cl)Cl)Cl | 1885 | 0 | 1 | 2 |
| Chlorbufam (C11H10ClNO2) | ULBXWWGWDPVHAO-UHFFFAOYSA-N | CC(C#C)OC(=O)NC1=CC(=CC=C1)Cl | 1331 | 0 | 1 | 2 |
| Trietazine (C9H16ClN5) | HFBWPRKWDIRYNX-UHFFFAOYSA-N | CCNC1=NC(=NC(=N1)Cl)N(CC)CC | 4320 | 250 | 1 | 1 |
| Methyl 4-(p-chlorophenoxy)butyrate (C11H13ClO3) | NKQYQRFVXNXTBS-UHFFFAOYSA-N | COC(=O)CCCOC1=CC=C(C=C1)Cl | 270 | 0 | 2 | 2 |
| 4-Chlorobutyric acid, 4-methoxyphenyl ester (C11H13ClO3) | JBOBRZWEFOHJFK-UHFFFAOYSA-N | COC1=CC=C(C=C1)OC(=O)CCCCl | 430 | 0 | 2 | 2 |
| Methyl 3-(4-chlorophenylthio)-2-methylpropionate (C11H13ClO2S) | LQTPIEYMBJPFQS-UHFFFAOYSA-N | CC(CSC1=CC=C(C=C1)Cl)C(=O)OC | 5313 | 0 | 1 | 2 |
| 3-Chlorophenol, isoBOC (C11H13ClO3) | RHVHALPDQOPUPP-UHFFFAOYSA-N | CC(C)COC(=O)OC1=CC(=CC=C1)Cl | 70 | 0 | 2 | 2 |
| ethyl 4-chloro-o-tolyloxyacetate (C11H13ClO3) | OUYDEKFRLSFDMU-UHFFFAOYSA-N | CCOC(=O)COC1=C(C=C(C=C1)Cl)C | 9999 | 30 | 1 | 2 |
| CPIB methyl ester (C11H13ClO3) | UXIVINXAZVEIMC-UHFFFAOYSA-N | CC(C)(C(=O)OC)OC1=CC=C(C=C1)Cl | 851 | 0 | 1 | 2 |
| 4-Bis(2-chloroethyl)aminobenzaldehyde (C11H13Cl2NO) | PXUFHXLGUJLBMI-UHFFFAOYSA-N | C1=CC(=CC=C1C=O)N(CCCl)CCCl | 992 | 116 | 1 | 1 |
| Butanoic acid, 4-(2,4-dichlorophenoxy)- (C10H10Cl2O3) | YIVXMZJTEQBPQO-UHFFFAOYSA-N | C1=CC(=C(C=C1Cl)Cl)OCCCC(=O)O | 589 | 0 | 2 | 2 |
| Dichloroacetamide, N-ethyl-N-(3-methylphenyl)- (C11H13Cl2NO) | SAURIVDRNUMSSC-UHFFFAOYSA-N | CCN(C1=CC=CC(=C1)C)C(=O)C(Cl)Cl | 1772 | 611 | 1 | 1 |
| 1,3,5-Benzenetricarbonyl trichloride (C9H3Cl3O3) | UWCPYKQBIPYOLX-UHFFFAOYSA-N | C1=C(C=C(C=C1C(=O)Cl)C(=O)Cl)C(=O)Cl | 0 | 9999 | 2 | 1 |
| Rose acetate (C10H9Cl3O2) | JKRWZLOCPLZZEI-UHFFFAOYSA-N | CC(=O)OC(C1=CC=CC=C1)C(Cl)(Cl)Cl | 0 | 0 | 2 | 2 |
| Trichloroacetic acid, 3,5-dimethylphenyl ester (C10H9Cl3O2) | RKKOPRPERWQZIH-UHFFFAOYSA-N | CC1=CC(=CC(=C1)OC(=O)C(Cl)(Cl)Cl)C | 1511 | 130 | 1 | 1 |
| 1,3,5-Triazine-2,4-diamine, 6-chloro-N-ethyl-N'-(1-methylpropyl)- (C9H16ClN5) | BZRUVKZGXNSXMB-UHFFFAOYSA-N | CCC(C)NC1=NC(=NC(=N1)NCC)Cl | 1249 | 0 | 1 | 2 |
| Propazine (C9H16ClN5) | WJNRPILHGGKWCK-UHFFFAOYSA-N | CC(C)NC1=NC(=NC(=N1)Cl)NC(C)C | 5268 | 55 | 1 | 2 |
| Terbuthylazine (C9H16ClN5) | FZXISNSWEXTPMF-UHFFFAOYSA-N | CCNC1=NC(=NC(=N1)Cl)NC(C)(C)C | 2679 | 35 | 1 | 2 |
| Butanoic acid, 4-(4-chloro-2-methylphenoxy)- (C10H13ClO3) | LLWADFLAOKUBDR-UHFFFAOYSA-N | CC1=C(C=CC(=C1)Cl)OCCCC(=O)O | 1419 | 0 | 1 | 2 |
| Propanamide, N-ethyl-N-(3-methylphenyl)-3-chloro- (C12H16ClNO) | CBHNNONZIXWDRN-UHFFFAOYSA-N | CCN(C1=CC=CC(=C1)C)C(=O)CCCl | 2682 | 1601 | 1 | 1 |
| Propanamide, N-ethyl-N-(3-methylphenyl)-2-chloro- (C12H16ClNO) | UQTVJWBAWLRDEE-UHFFFAOYSA-N | CCN(C1=CC=CC(=C1)C)C(=O)C(C)Cl | 7257 | 3853 | 1 | 1 |
| Pentanamide, N-(3-chlorophenyl)-5-chloro- (C11H13Cl2NO) | GYILROAJGQKZID-UHFFFAOYSA-N | C1=CC(=CC(=C1)Cl)NC(=O)CCCCCl | 1011 | 400 | 1 | 1 |
| 5-Chlorovaleric acid, 4-chlorophenyl ester (C11H12Cl2O2) | ZSYKPXRQYIMAOB-UHFFFAOYSA-N | C1=CC(=CC=C1OC(=O)CCCCCl)Cl | 861 | 0 | 1 | 2 |
| Valeric acid, 3,4-dichlorophenyl ester (C11H12Cl2O2) | YNABTDZJUAFPIV-UHFFFAOYSA-N | CCCCC(=O)OC1=CC(=C(C=C1)Cl)Cl | 669 | 0 | 2 | 2 |
| 2,6-Bis(chloromethyl)-p-tolyl acetate (C11H12Cl2O2) | KUMMZJSZVNWQLZ-UHFFFAOYSA-N | CC1=CC(=C(C(=C1)CCl)OC(=O)C)CCl | 129 | 233 | 2 | 1 |
| 3',4'-Dichloropivalanilide (C11H13Cl2NO) | WMFDYXPRRHDSQS-UHFFFAOYSA-N | CC(C)(C)C(=O)NC1=CC(=C(C=C1)Cl)Cl | 765 | 11 | 2 | 2 |
| 2,2-Dimethylpropanoic acid, 3,4-dichlorophenyl ester (C11H12Cl2O2) | XIRSVOKAKYHVOX-UHFFFAOYSA-N | CC(C)(C)C(=O)OC1=CC(=C(C=C1)Cl)Cl | 160 | 0 | 2 | 2 |
| 5-Chloropentyl benzoate (C12H15ClO2) | XIOZPPYPTAEPGJ-UHFFFAOYSA-N | C1=CC=C(C=C1)C(=O)OCCCCCCl | 64 | 85 | 2 | 2 |
| Pentanamide, N-(3-methylphenyl)-5-chloro- (C12H16ClNO) | KXBWQDPILGCNTC-UHFFFAOYSA-N | CC1=CC(=CC=C1)NC(=O)CCCCCl | 1381 | 420 | 1 | 1 |
| 5-Chlorovaleric acid, 3-methylphenyl ester (C12H15ClO2) | RDYAPVUPQWREBE-UHFFFAOYSA-N | CC1=CC(=CC=C1)OC(=O)CCCCCl | 220 | 0 | 2 | 2 |
| Hexanamide, N-(3-chlorophenyl)- (C12H16ClNO) | BAUYPJBSRHYPFK-UHFFFAOYSA-N | CCCCCC(=O)NC1=CC(=CC=C1)Cl | 961 | 0 | 1 | 2 |
| Benzoyl chloride, 4-(pentyloxy)- (C12H15ClO2) | IBQDPNHVFRFCFK-UHFFFAOYSA-N | CCCCCOC1=CC=C(C=C1)C(=O)Cl | 500 | 6620 | 2 | 1 |
| Benzoic acid, 2-chloro, pentyl ester (C12H15ClO2) | SUCIUJIXAHNYGX-UHFFFAOYSA-N | CCCCCOC(=O)C1=CC=CC=C1Cl | 260 | 0 | 2 | 2 |
| Pentyl 4-chlorobenzoate (C12H15ClO2) | APVFEMBQOQUREJ-UHFFFAOYSA-N | CCCCCOC(=O)C1=CC=C(C=C1)Cl | 50 | 0 | 2 | 2 |
| Benzoic acid, 4-chloro, 3-methylbutyl ester (C12H15ClO2) | YBRVJNBUZZRJDV-UHFFFAOYSA-N | CC(C)CCOC(=O)C1=CC=C(C=C1)Cl | 0 | 0 | 2 | 2 |
| Bennzoic acid, 2-chloro, 3-methylbutyl ester (C12H15ClO2) | MPUGTFANXARAQE-UHFFFAOYSA-N | CC(C)CCOC(=O)C1=CC=CC=C1Cl | 0 | 0 | 2 | 2 |
| 2-chloro-2',6'-diethylacetanilide (C12H16ClNO) | LBJVHMAYBNQJBK-UHFFFAOYSA-N | CCC1=C(C(=CC=C1)CC)NC(=O)CCl | 1301 | 60 | 1 | 2 |
| Benzoic acid, 2-chloro, 1-methylbutyl ester (C12H15ClO2) | RMHHIFFWPDEYER-UHFFFAOYSA-N | CCCC(C)OC(=O)C1=CC=CC=C1Cl | 50 | 0 | 2 | 2 |
| Benzoic acid, 4-chloro, 1-methylbutyl ester (C12H15ClO2) | YAOLJHAAVJEVSU-UHFFFAOYSA-N | CCCC(C)OC(=O)C1=CC=C(C=C1)Cl | 30 | 20 | 2 | 2 |
| 8-Phenyl-1-octyl chloride (C14H21Cl) | GVDYDOFBAKCSGC-UHFFFAOYSA-N | C1=CC=C(C=C1)CCCCCCCCCl | 744 | 0 | 2 | 2 |
| 2-Chlorocyclododecanone oxime (C12H22ClNO) | PMQJQOIUPPQMCK-UHFFFAOYSA-N | C1CCCCCC(=NO)C(CCCC1)Cl | 4704 | 9999 | 1 | 1 |
| (Phenylthio)acetic acid, 3-chloroprop-2-enyl ester (C11H11ClO2S) | RUHVHMBFVMISGJ-QPJJXVBHSA-N | C1=CC=C(C=C1)SCC(=O)OC/C=C/Cl | 4575 | 70 | 1 | 2 |
| o-anisic acid, 3-chloroprop-2-enyl ester (C11H11ClO3) | WBWAKPXHPTXRTF-QPJJXVBHSA-N | COC1=CC=CC=C1C(=O)OC/C=C/Cl | 611 | 1381 | 2 | 1 |
| p-Anisic acid, 3-chloroprop-2-enyl ester (C11H11ClO3) | TXUYHADQUJYBNC-FARCUNLSSA-N | COC1=CC=C(C=C1)C(=O)OC/C=C/Cl | 380 | 1732 | 2 | 1 |
| Uracil Mustard (C8H11Cl2N3O2) | IDPUKCWIGUEADI-UHFFFAOYSA-N | C1=C(C(=O)NC(=O)N1)N(CCCl)CCCl | 1290 | 1155 | 1 | 1 |
| 4-Cyanobenzoic acid, 3-chloroprop-2-enyl ester (C11H8ClNO2) | XVSJUDNOFHITQB-LZCJLJQNSA-N | C1=CC(=CC=C1C#N)C(=O)OC/C=C/Cl | 0 | 1431 | 2 | 1 |
| 4-Allyloxy-3-chlorophenylacetic acid (C11H11ClO3) | ARHWPKZXBHOEEE-UHFFFAOYSA-N | C=CCOC1=C(C=C(C=C1)CC(=O)O)Cl | 2689 | 560 | 1 | 1 |
| 3-Methylbut-2-enoic acid, 3,4-dichlorophenyl ester (C11H10Cl2O2) | ZFPKWERGBZSOTQ-UHFFFAOYSA-N | CC(=CC(=O)OC1=CC(=C(C=C1)Cl)Cl)C | 100 | 0 | 2 | 2 |
| 2-Chlorobenzoic acid, pent-2-en-4-ynyl ester (C12H9ClO2) | OYJLGUJTTGZWJG-UHFFFAOYSA-N | C#CC=CCOC(=O)C1=CC=CC=C1Cl | 160 | 40 | 2 | 2 |
| 4-Chlorobenzoic acid, pent-2-en-4-ynyl ester (C12H9ClO2) | BTORAKBXDGGVSR-UHFFFAOYSA-N | C#CC=CCOC(=O)C1=CC=C(C=C1)Cl | 190 | 0 | 2 | 2 |
| 4-ethylbenzoic acid, 3-chloroprop-2-enyl ester (C12H13ClO2) | SJAFWAMUNZHDSD-FPYGCLRLSA-N | CCC1=CC=C(C=C1)C(=O)OC/C=C/Cl | 150 | 2232 | 2 | 1 |
| 4-Chlorobenzoic acid, 3-methylbut-2-enyl ester (C12H13ClO2) | SLMPDOANKJZGFW-UHFFFAOYSA-N | CC(=CCOC(=O)C1=CC=C(C=C1)Cl)C | 280 | 30 | 2 | 2 |
| Diazene, (4-chlorophenyl)phenyl- (C12H9ClN2) | NJFDMENHTAYHMA-UHFFFAOYSA-N | C1=CC=C(C=C1)N=NC2=CC=C(C=C2)Cl | 4474 | 0 | 1 | 2 |
| Diazene, (4-chlorophenyl)phenyl-, (E)- (C12H9ClN2) | NJFDMENHTAYHMA-UHFFFAOYSA-N | C1=CC=C(C=C1)N=NC2=CC=C(C=C2)Cl | 3586 | 0 | 1 | 2 |
| N-(4-Chlorophenyl)-1,2-phenylenediamine (C12H11ClN2) | WEUBIWJPIRTWDF-UHFFFAOYSA-N | C1=CC=C(C(=C1)N)NC2=CC=C(C=C2)Cl | 9999 | 5985 | 1 | 1 |
| 4,4'-Dichlorodiphenylsulphide (C12H8Cl2S) | MJEPOVIWHVRBIT-UHFFFAOYSA-N | C1=CC(=CC=C1SC2=CC=C(C=C2)Cl)Cl | 9999 | 1137 | 1 | 1 |
| Benzene, 1,1'-oxybis[4-chloro- (C12H8Cl2O) | URUJZHZLCCIILC-UHFFFAOYSA-N | C1=CC(=CC=C1OC2=CC=C(C=C2)Cl)Cl | 9999 | 230 | 1 | 1 |
| Benzene, 2,4-dichloro-1-phenoxy- (C12H8Cl2O) | KXIPYLZZJZMMPD-UHFFFAOYSA-N | C1=CC=C(C=C1)OC2=C(C=C(C=C2)Cl)Cl | 9999 | 5265 | 1 | 1 |
| Acetamide, N-(1-naphthyl)-2-chloro- (C12H10ClNO) | CVRUANQADYCNLO-UHFFFAOYSA-N | C1=CC=C2C(=C1)C=CC=C2NC(=O)CCl | 7156 | 280 | 1 | 1 |
| Chloroacetic acid, 2-naphthyl ester (C12H9ClO2) | DQHZHTWQJQJIKE-UHFFFAOYSA-N | C1=CC=C2C=C(C=CC2=C1)OC(=O)CCl | 1491 | 0 | 1 | 2 |
| Benzene, 1-(chloromethyl)-3-phenoxy- (C13H11ClO) | QUYVTGFWFHQVRO-UHFFFAOYSA-N | C1=CC=C(C=C1)OC2=CC=CC(=C2)CCl | 7102 | 9999 | 1 | 1 |
| 1,1'-Biphenyl,3-chloro-4-methoxy- (C13H11ClO) | SLBYCCHSURAIIK-UHFFFAOYSA-N | COC1=C(C=C(C=C1)C2=CC=CC=C2)Cl | 9139 | 0 | 1 | 2 |
| 1,1'-Biphenyl-4-ol, 3,5-dichloro (C12H8Cl2O) | HHFXDKAXCKUSIV-UHFFFAOYSA-N | C1=CC=C(C=C1)C2=CC(=C(C(=C2)Cl)O)Cl | 9999 | 50 | 1 | 2 |
| [1,1'-Biphenyl]-2-ol, 3,5-dichloro- (C12H8Cl2O) | AFXPPGXJWHJULD-UHFFFAOYSA-N | C1=CC=C(C=C1)C2=CC(=CC(=C2O)Cl)Cl | 9999 | 550 | 1 | 1 |
| 1,1'-Biphenyl-4-ol, 2',5'-dichloro (C12H8Cl2O) | BTORSXCJJIWNIS-UHFFFAOYSA-N | C1=CC(=CC=C1C2=C(C=CC(=C2)Cl)Cl)O | 9999 | 50 | 1 | 2 |
| 1,1'-Biphenyl-3-ol, 2',5'-dichloro (C12H8Cl2O) | MRRLNQOPCMALNK-UHFFFAOYSA-N | C1=CC(=CC(=C1)O)C2=C(C=CC(=C2)Cl)Cl | 9999 | 170 | 1 | 1 |
| 1,1'-Biphenyl-2-ol, 2',5'-dichloro (C12H8Cl2O) | LKDWVOYFEVHMEJ-UHFFFAOYSA-N | C1=CC=C(C(=C1)C2=C(C=CC(=C2)Cl)Cl)O | 9999 | 2710 | 1 | 1 |
| [1,1'-Biphenyl]-4-ol, 3,4'-dichloro- (C12H8Cl2O) | UHKLHHYQCUBQDI-UHFFFAOYSA-N | C1=CC(=CC=C1C2=CC(=C(C=C2)O)Cl)Cl | 9999 | 40 | 1 | 2 |
| PCB 29 (C12H7Cl3) | VGVIKVCCUATMNG-UHFFFAOYSA-N | C1=CC=C(C=C1)C2=CC(=C(C=C2Cl)Cl)Cl | 9999 | 167 | 1 | 1 |
| 2,4',5-Trichlorobiphenyl (C12H7Cl3) | VAHKBZSAUKPEOV-UHFFFAOYSA-N | C1=CC(=CC=C1C2=C(C=CC(=C2)Cl)Cl)Cl | 8307 | 178 | 1 | 1 |
| PCB 30 (C12H7Cl3) | MTLMVEWEYZFYTH-UHFFFAOYSA-N | C1=CC=C(C=C1)C2=C(C=C(C=C2Cl)Cl)Cl | 9999 | 389 | 1 | 1 |
| PCB 18 (C12H7Cl3) | DCMURXAZTZQAFB-UHFFFAOYSA-N | C1=CC=C(C(=C1)C2=C(C=CC(=C2)Cl)Cl)Cl | 4974 | 4113 | 1 | 1 |
| PCB 17 (C12H7Cl3) | YKKYCYQDUUXNLN-UHFFFAOYSA-N | C1=CC=C(C(=C1)C2=C(C=C(C=C2)Cl)Cl)Cl | 9293 | 3493 | 1 | 1 |
| PCB 34 (C12H7Cl3) | GXVMAQACUOSFJF-UHFFFAOYSA-N | C1=CC=C(C(=C1)C2=CC(=CC(=C2)Cl)Cl)Cl | 9999 | 233 | 1 | 1 |
| PCB 35 (C12H7Cl3) | JHBVPKZLIBDTJR-UHFFFAOYSA-N | C1=CC(=CC(=C1)Cl)C2=CC(=C(C=C2)Cl)Cl | 9999 | 183 | 1 | 1 |
| PCB 19 (C12H7Cl3) | MVXIJRBBCDLNLX-UHFFFAOYSA-N | C1=CC=C(C(=C1)C2=C(C=CC=C2Cl)Cl)Cl | 9892 | 6009 | 1 | 1 |
| PCB 32 (C12H7Cl3) | IHIDFKLAWYPTKB-UHFFFAOYSA-N | C1=CC(=C(C(=C1)Cl)C2=CC=C(C=C2)Cl)Cl | 9471 | 214 | 1 | 1 |
| PCB 16 (C12H7Cl3) | XVIZMMSINIOIQP-UHFFFAOYSA-N | C1=CC=C(C(=C1)C2=C(C(=CC=C2)Cl)Cl)Cl | 9999 | 5945 | 1 | 1 |
| PCB 26 (C12H7Cl3) | ONNCPBRWFSKDMQ-UHFFFAOYSA-N | C1=CC(=CC(=C1)Cl)C2=C(C=CC(=C2)Cl)Cl | 9999 | 196 | 1 | 1 |
| PCB 20 (C12H7Cl3) | SXHLTVKPNQVZGL-UHFFFAOYSA-N | C1=CC(=CC(=C1)Cl)C2=C(C(=CC=C2)Cl)Cl | 9492 | 314 | 1 | 1 |
| PCB 22 (C12H7Cl3) | ZMHWQAHZKUPENF-UHFFFAOYSA-N | C1=CC(=C(C(=C1)Cl)Cl)C2=CC=C(C=C2)Cl | 9999 | 195 | 1 | 1 |
| PCB 33 (C12H7Cl3) | RIMXLXBUOQMDHV-UHFFFAOYSA-N | C1=CC=C(C(=C1)C2=CC(=C(C=C2)Cl)Cl)Cl | 9838 | 195 | 1 | 1 |
| PCB 36 (C12H7Cl3) | RIBGNAJQTOXRDK-UHFFFAOYSA-N | C1=CC(=CC(=C1)Cl)C2=CC(=CC(=C2)Cl)Cl | 9620 | 315 | 1 | 1 |
| PCB 39 (C12H7Cl3) | SYSBNFJJSJLZMM-UHFFFAOYSA-N | C1=CC(=CC=C1C2=CC(=CC(=C2)Cl)Cl)Cl | 9999 | 123 | 1 | 1 |
| PCB 37 (C12H7Cl3) | YZANRISAORXTHU-UHFFFAOYSA-N | C1=CC(=CC=C1C2=CC(=C(C=C2)Cl)Cl)Cl | 9999 | 118 | 1 | 1 |
| PCB 38 (C12H7Cl3) | BSFZSQRJGZHMMV-UHFFFAOYSA-N | C1=CC=C(C=C1)C2=CC(=C(C(=C2)Cl)Cl)Cl | 9999 | 245 | 1 | 1 |
| PCB 24 (C12H7Cl3) | LVROLHVSYNLFBE-UHFFFAOYSA-N | C1=CC=C(C=C1)C2=C(C=CC(=C2Cl)Cl)Cl | 9999 | 390 | 1 | 1 |
| PCB 21 (C12H7Cl3) | IUYHQGMDSZOPDZ-UHFFFAOYSA-N | C1=CC=C(C=C1)C2=C(C(=C(C=C2)Cl)Cl)Cl | 9999 | 203 | 1 | 1 |
| PCB 25 (C12H7Cl3) | XBBZAULFUPBZSP-UHFFFAOYSA-N | C1=CC(=CC(=C1)Cl)C2=C(C=C(C=C2)Cl)Cl | 9655 | 271 | 1 | 1 |
| PCB 23 (C12H7Cl3) | GBUCDGDROYMOAN-UHFFFAOYSA-N | C1=CC=C(C=C1)C2=CC(=CC(=C2Cl)Cl)Cl | 9999 | 229 | 1 | 1 |
| Phthalic anhydride, tetrachloro- (C8Cl4O3) | AUHHYELHRWCWEZ-UHFFFAOYSA-N | C12=C(C(=C(C(=C1Cl)Cl)Cl)Cl)C(=O)OC2=O | 5905 | 0 | 1 | 2 |
| Methanone,(2-chlorophenyl)-2-pyridinyl- (C12H8ClNO) | XSPNJXJVXUZKPE-UHFFFAOYSA-N | C1=CC=C(C(=C1)C(=O)C2=CC=CC=N2)Cl | 290 | 9999 | 2 | 1 |
| Benzenemethanamine, 4-chloro-α-phenyl- (C13H12ClN) | XAFODXGEQUOEKN-UHFFFAOYSA-N | C1=CC=C(C=C1)C(C2=CC=C(C=C2)Cl)N | 2899 | 549 | 1 | 1 |
| Phenyl-(4-chlorophenyl)carbinol (C13H11ClO) | AJYOOHCNOXWTKJ-UHFFFAOYSA-N | C1=CC=C(C=C1)C(C2=CC=C(C=C2)Cl)O | 3169 | 1069 | 1 | 1 |
| o-Benzyl-p-chlorophenol (C13H11ClO) | NCKMMSIFQUPKCK-UHFFFAOYSA-N | C1=CC=C(C=C1)CC2=C(C=CC(=C2)Cl)O | 9999 | 6999 | 1 | 1 |
| Methanone, (3-chlorophenyl)phenyl- (C13H9ClO) | CPLWKNRPZVNELG-UHFFFAOYSA-N | C1=CC=C(C=C1)C(=O)C2=CC(=CC=C2)Cl | 6606 | 1001 | 1 | 1 |
| Hydroxyzine (chloro-benzophenone) (C13H9ClO) | UGVRJVHOJNYEHR-UHFFFAOYSA-N | C1=CC=C(C=C1)C(=O)C2=CC=C(C=C2)Cl | 5049 | 1329 | 1 | 1 |
| Methanone, (2-chlorophenyl)phenyl- (C13H9ClO) | VMHYWKBKHMYRNF-UHFFFAOYSA-N | C1=CC=C(C=C1)C(=O)C2=CC=CC=C2Cl | 3699 | 439 | 1 | 1 |
| Chloro(p-chlorophenyl)phenylmethane (C13H10Cl2) | ALKWTKGPKKAZMN-UHFFFAOYSA-N | C1=CC=C(C=C1)C(C2=CC=C(C=C2)Cl)Cl | 109 | 9769 | 2 | 1 |
| Benzene, 1,1'-(dichloromethylene)bis- (C13H10Cl2) | OPTDDWCXQQYKGU-UHFFFAOYSA-N | C1=CC=C(C=C1)C(C2=CC=CC=C2)(Cl)Cl | 50 | 9999 | 2 | 1 |
| 2-Chlorobenzoic acid, morpholide (C11H12ClNO2) | ZRGHSGNYLSCFJY-UHFFFAOYSA-N | C1COCCN1C(=O)C2=CC=CC=C2Cl | 601 | 530 | 2 | 1 |
| 4-Chlorobenzoic acid, morpholide (C11H12ClNO2) | BGRFQNTYHMHVFJ-UHFFFAOYSA-N | C1COCCN1C(=O)C2=CC=C(C=C2)Cl | 1621 | 50 | 1 | 2 |
| Bibenzyl, 3-chloro- (C14H13Cl) | NWVGKFZMBBPAJL-UHFFFAOYSA-N | C1=CC=C(C=C1)CCC2=CC(=CC=C2)Cl | 1451 | 0 | 1 | 2 |
| Cyprazine (C9H12ClN5) | OOHIAOSLOGDBCE-UHFFFAOYSA-N | CC(C)NC1=NC(=NC(=N1)NC2CC2)Cl | 4710 | 500 | 1 | 1 |
| Cyclobutanecarboxylic acid, 3,4-dichlorophenyl ester (C11H10Cl2O2) | APPODQFYNHYRBW-UHFFFAOYSA-N | C1CC(C1)C(=O)OC2=CC(=C(C=C2)Cl)Cl | 641 | 0 | 2 | 2 |
| Cyclopentanecarboxamide, N-(3-chlorophenyl)- (C12H14ClNO) | YOKCBEZMIXZJTA-UHFFFAOYSA-N | C1CCC(C1)C(=O)NC2=CC(=CC=C2)Cl | 2212 | 0 | 1 | 2 |
| Cyclopentanecarboxylic acid, 4-chlorophenyl ester (C12H13ClO2) | ZMLQNIGUNYBKJO-UHFFFAOYSA-N | C1CCC(C1)C(=O)OC2=CC=C(C=C2)Cl | 731 | 0 | 2 | 2 |
| 8-Chlorotheophylline (C7H7ClN4O2) | RYIGNEOBDRVTHA-UHFFFAOYSA-N | CN1C2=C(C(=O)N(C1=O)C)NC(=N2)Cl | 9999 | 0 | 1 | 2 |
| chloridazon (C10H8ClN3O) | WYKYKTKDBLFHCY-UHFFFAOYSA-N | C1=CC=C(C=C1)N2C(=O)C(=C(C=N2)N)Cl | 7767 | 0 | 1 | 2 |
| 1,1-Diphenyl-2-chloroethylene (C14H11Cl) | DLIRODSKOPSWFS-UHFFFAOYSA-N | C1=CC=C(C=C1)C(=CCl)C2=CC=CC=C2 | 9205 | 9756 | 1 | 1 |
| Methyl 4-chloroindolyl-3-acetate (C11H10ClNO2) | SYPGJEURLIGNPE-UHFFFAOYSA-N | COC(=O)CC1=CNC2=C1C(=CC=C2)Cl | 2350 | 0 | 1 | 2 |
| 6,8-Dichlorochromone-3-carboxaldehyde (C10H4Cl2O3) | IHCCHRKNCOFDAJ-UHFFFAOYSA-N | C1=C(C=C2C(=C1Cl)OC=C(C2=O)C=O)Cl | 358 | 0 | 2 | 2 |
| Thiophene-2-carboxamide, N-(3-chlorophenyl)- (C11H8ClNOS) | FHTIWIPMVNMNHO-UHFFFAOYSA-N | C1=CC(=CC(=C1)Cl)NC(=O)C2=CC=CS2 | 2702 | 0 | 1 | 2 |
| 2-Furancarboxamide, N-(3-chlorophenyl)- (C11H8ClNO2) | GXPIOIIKCASZKA-UHFFFAOYSA-N | C1=CC(=CC(=C1)Cl)NC(=O)C2=CC=CO2 | 3883 | 30 | 1 | 2 |
| 2-Thiophenecarboxylic acid, 4-chlorophenyl ester (C11H7ClO2S) | RHDSCYYKAZNOCO-UHFFFAOYSA-N | C1=CSC(=C1)C(=O)OC2=CC=C(C=C2)Cl | 731 | 0 | 2 | 2 |
| 2-Furoic acid, 4-chlorophenyl ester (C11H7ClO3) | ZGZHQUGYCJLSQM-UHFFFAOYSA-N | C1=COC(=C1)C(=O)OC2=CC=C(C=C2)Cl | 1942 | 0 | 1 | 2 |
| Fenpiclonil (C11H6Cl2N2) | FKLFBQCQQYDUAM-UHFFFAOYSA-N | C1=CC(=C(C(=C1)Cl)Cl)C2=CNC=C2C#N | 9999 | 3573 | 1 | 1 |
| Heptenophos (C9H12ClO4P) | GBAWQJNHVWMTLU-UHFFFAOYSA-N | COP(=O)(OC)OC1=C(C2C1CC=C2)Cl | 940 | 1310 | 1 | 1 |
| 5-Chloro-1,10-phenanthroline (C12H7ClN2) | XDUUQOQFSWSZSM-UHFFFAOYSA-N | C1=CC2=CC(=C3C=CC=NC3=C2N=C1)Cl | 9999 | 1419 | 1 | 1 |
| Phenoxathiin, 2-chloro- (C12H7ClOS) | QQGJRVZIBGEUGZ-UHFFFAOYSA-N | C1=CC=C2C(=C1)OC3=C(S2)C=C(C=C3)Cl | 9999 | 2759 | 1 | 1 |
| Dibenzo[b,e][1,4]dioxin, 1-chloro- (C12H7ClO2) | VGGGRWRBGXENKI-UHFFFAOYSA-N | C1=CC=C2C(=C1)OC3=C(O2)C(=CC=C3)Cl | 9999 | 207 | 1 | 1 |
| Dibenzo-p-dioxin, 2-chloro (C12H7ClO2) | GIUGGRUEPHPVNR-UHFFFAOYSA-N | C1=CC=C2C(=C1)OC3=C(O2)C=C(C=C3)Cl | 9999 | 966 | 1 | 1 |
| Acridine, 9-chloro- (C13H8ClN) | BPXINCHFOLVVSG-UHFFFAOYSA-N | C1=CC=C2C(=C1)C(=C3C=CC=CC3=N2)Cl | 9999 | 2570 | 1 | 1 |
| Anthracene, 2-chloro- (C14H9Cl) | OWFINXQLBMJDJQ-UHFFFAOYSA-N | C1=CC=C2C=C3C=C(C=CC3=CC2=C1)Cl | 9999 | 1299 | 1 | 1 |
| Anthracene, 1-chloro- (C14H9Cl) | SRIHSAFSOOUEGL-UHFFFAOYSA-N | C1=CC=C2C=C3C(=CC2=C1)C=CC=C3Cl | 9999 | 1129 | 1 | 1 |
| 9-Chloroanthracene (C14H9Cl) | KULLJOPUZUWTMF-UHFFFAOYSA-N | C1=CC=C2C(=C1)C=C3C=CC=CC3=C2Cl | 9999 | 1483 | 1 | 1 |
| 9-Chlorophenanthrene (C14H9Cl) | CJWHZQNUDAJJSB-UHFFFAOYSA-N | C1=CC=C2C(=C1)C=C(C3=CC=CC=C23)Cl | 9999 | 1857 | 1 | 1 |
| Dibenzofuran, 2,8-dichloro- (C12H6Cl2O) | IVVRJIDVYSPKFZ-UHFFFAOYSA-N | C1=CC2=C(C=C1Cl)C3=C(O2)C=CC(=C3)Cl | 9999 | 79 | 1 | 2 |
| Succinic acid, di(2,2-dichloroethyl) ester (C8H10Cl4O4) | APJYUYGGYUZQET-UHFFFAOYSA-N | C(CC(=O)OCC(Cl)Cl)C(=O)OCC(Cl)Cl | 0 | 0 | 2 | 2 |
| Propanoic acid, 2,2-dichloro-, 1,2-ethanediyl ester (C8H10Cl4O4) | HDVMFCLSSJBSOM-UHFFFAOYSA-N | CC(C(=O)OCCOC(=O)C(C)(Cl)Cl)(Cl)Cl | 0 | 0 | 2 | 2 |
| Succinic acid, propyl 2,2,2-trichloroethyl ester (C9H13Cl3O4) | XDAKQGGVMSYSGN-UHFFFAOYSA-N | CCCOC(=O)CCC(=O)OCC(Cl)(Cl)Cl | 0 | 0 | 2 | 2 |
| Succinic acid, butyl 2,2-dichloroethyl ester (C10H16Cl2O4) | CBJUDFLVVQADHN-UHFFFAOYSA-N | CCCCOC(=O)CCC(=O)OCC(Cl)Cl | 0 | 0 | 2 | 2 |
| Succinic acid, di(2-chloropropyl) ester (C10H16Cl2O4) | RKTVUISFJBUYMU-UHFFFAOYSA-N | CC(COC(=O)CCC(=O)OCC(C)Cl)Cl | 0 | 0 | 2 | 2 |
| Succinic acid, 2,2-dichloroethyl isobutyl ester (C10H16Cl2O4) | JRIJGNIUJAIFKV-UHFFFAOYSA-N | CC(C)COC(=O)CCC(=O)OCC(Cl)Cl | 0 | 0 | 2 | 2 |
| Succinic acid, butyl 2-chloropropyl ester (C11H19ClO4) | TUQSZQVARNKNCQ-UHFFFAOYSA-N | CCCCOC(=O)CCC(=O)OCC(C)Cl | 0 | 0 | 2 | 2 |
| L-Valine, N-(5-chlrovaleryl)-, methyl ester (C11H20ClNO3) | ZDCLUYAODLSBMX-UHFFFAOYSA-N | CC(C)C(C(=O)OC)NC(=O)CCCCCl | 0 | 380 | 2 | 1 |
| Succinic acid, 2-chloropropyl isobutyl ester (C11H19ClO4) | SLQQAJCFLZYRKR-UHFFFAOYSA-N | CC(C)COC(=O)CCC(=O)OCC(C)Cl | 0 | 0 | 2 | 2 |
| Trichloroacetic acid, 2-methyloct-5-yn-4-yl ester (C11H15Cl3O2) | BFHVFJFRDWWLLF-UHFFFAOYSA-N | CCC#CC(CC(C)C)OC(=O)C(Cl)(Cl)Cl | 0 | 190 | 2 | 1 |
| Nonyl trichloroacetate (C11H19Cl3O2) | KZFXYGVHWVJDHZ-UHFFFAOYSA-N | CCCCCCCCCOC(=O)C(Cl)(Cl)Cl | 0 | 0 | 2 | 2 |
| 11-Chloroundecanoic acid, chloromethyl ester (C12H22Cl2O2) | ZLIBDGLPYRUFEI-UHFFFAOYSA-N | C(CCCCCCl)CCCCC(=O)OCCl | 0 | 0 | 2 | 2 |
| 10-Chloroundecanoic acid, chloromethyl ester (C12H22Cl2O2) | PBAUXMNCDJRVRG-UHFFFAOYSA-N | CC(CCCCCCCCC(=O)OCCl)Cl | 0 | 0 | 2 | 2 |
| 9-Chloroundecanoic acid, chloromethyl ester (C12H22Cl2O2) | ADXXJEWPDOIYQC-UHFFFAOYSA-N | CCC(CCCCCCCC(=O)OCCl)Cl | 0 | 0 | 2 | 2 |
| 8-Chloroundecanoic acid, chloromethyl ester (C12H22Cl2O2) | APKARRRMGNWDTO-UHFFFAOYSA-N | CCCC(CCCCCCC(=O)OCCl)Cl | 0 | 0 | 2 | 2 |
| 2-Chloroundecanoic acid, chloromethyl ester (C12H22Cl2O2) | LSNQBMHSPMRKRR-UHFFFAOYSA-N | CCCCCCCCCC(C(=O)OCCl)Cl | 0 | 0 | 2 | 2 |
| 5-Chloroundecanoic acid, chloromethyl ester (C12H22Cl2O2) | YEZLUIOASUOLRC-UHFFFAOYSA-N | CCCCCCC(CCCC(=O)OCCl)Cl | 0 | 0 | 2 | 2 |
| 6-Chloroundecanoic acid, chloromethyl ester (C12H22Cl2O2) | NHURGYSRXYAHKJ-UHFFFAOYSA-N | CCCCCC(CCCCC(=O)OCCl)Cl | 0 | 0 | 2 | 2 |
| 7-Chloroundecanoic acid, chloromethyl ester (C12H22Cl2O2) | PDOUDFSWPSGPMS-UHFFFAOYSA-N | CCCCC(CCCCCC(=O)OCCl)Cl | 0 | 0 | 2 | 2 |
| Decyl dichloroacetate (C12H22Cl2O2) | ZMLAQDPXQKQOGC-UHFFFAOYSA-N | CCCCCCCCCCOC(=O)C(Cl)Cl | 0 | 0 | 2 | 2 |
| Dodecanoic acid, chloromethyl ester (C13H25ClO2) | XCHDYWCWXAASER-UHFFFAOYSA-N | CCCCCCCCCCCC(=O)OCCl | 1251 | 0 | 1 | 2 |
| Propanoic acid, 3-chloro, decyl ester (C13H25ClO2) | HOFHJGXZUUNNDQ-UHFFFAOYSA-N | CCCCCCCCCCOC(=O)CCCl | 0 | 70 | 2 | 2 |
| 5-Chlorovaleric acid, octyl ester (C13H25ClO2) | KWLSFBAAGRONDK-UHFFFAOYSA-N | CCCCCCCCOC(=O)CCCCCl | 0 | 210 | 2 | 1 |
| 2-Chlorododecanoic acid, methyl ester (C13H25ClO2) | LVITUWGEPWZWRD-UHFFFAOYSA-N | CCCCCCCCCCC(C(=O)OC)Cl | 0 | 440 | 2 | 1 |
| Propanoic acid, 2-chloro, decyl ester (C13H25ClO2) | ZULQXFOAUSXCIJ-UHFFFAOYSA-N | CCCCCCCCCCOC(=O)C(C)Cl | 0 | 0 | 2 | 2 |
| Myristoyl chloride (C14H27ClO) | LPWCRLGKYWVLHQ-UHFFFAOYSA-N | CCCCCCCCCCCCCC(=O)Cl | 0 | 1864 | 2 | 1 |
| Fumaric acid, di(2,2-dichloroethyl) ester (C8H8Cl4O4) | FSRFBNQOJOZBHW-OWOJBTEDSA-N | C(C(Cl)Cl)OC(=O)/C=C/C(=O)OCC(Cl)Cl | 0 | 60 | 2 | 2 |
| Fumaric acid, propyl 2,2,2-trichloroethyl ester (C9H11Cl3O4) | GQQOPVNABGMTHV-ONEGZZNKSA-N | CCCOC(=O)/C=C/C(=O)OCC(Cl)(Cl)Cl | 0 | 1451 | 2 | 1 |
| Triallate (C10H16Cl3NOS) | MWBPRDONLNQCFV-UHFFFAOYSA-N | CC(C)N(C(C)C)C(=O)SCC(=C(Cl)Cl)Cl | 11 | 2313 | 2 | 1 |
| Fumaric acid, butyl 2,2-dichloroethyl ester (C10H14Cl2O4) | DHVYZRRCEDWZEQ-SNAWJCMRSA-N | CCCCOC(=O)/C=C/C(=O)OCC(Cl)Cl | 0 | 80 | 2 | 2 |
| Fumaric acid, di(2-chloropropyl) ester (C10H14Cl2O4) | XTVLIWMGYKFLMA-ONEGZZNKSA-N | CC(COC(=O)/C=C/C(=O)OCC(C)Cl)Cl | 0 | 0 | 2 | 2 |
| Fumaric acid, 2,2-dichloroethyl isobutyl ester (C10H14Cl2O4) | IKJKPEUGXREZPB-ONEGZZNKSA-N | CC(C)COC(=O)/C=C/C(=O)OCC(Cl)Cl | 0 | 70 | 2 | 2 |
| Fumaric acid, butyl 2-chloropropyl ester (C11H17ClO4) | DGWPOGSZJVYMBF-AATRIKPKSA-N | CCCCOC(=O)/C=C/C(=O)OCC(C)Cl | 0 | 30 | 2 | 2 |
| Dichloroacetic acid, 2,7-dimethyloct-7-en-5-yn-4-yl (C12H16Cl2O2) | VSXCBHKTNKYJJP-UHFFFAOYSA-N | CC(C)CC(C#CC(=C)C)OC(=O)C(Cl)Cl | 330 | 841 | 2 | 1 |
| 3-Cloropropionic acid, 2,7-dimethyloct-7-en-5-yn-4-yl ester (C13H19ClO2) | NJMIWJNQZFQLFM-UHFFFAOYSA-N | CC(C)CC(C#CC(=C)C)OC(=O)CCCl | 921 | 290 | 1 | 1 |
| 5-chlorovaleric acid, oct-3-en-2-yl ester (C13H23ClO2) | WLTIAUVTCDQKPT-RMKNXTFCSA-N | CCCC/C=C/C(C)OC(=O)CCCCCl | 70 | 130 | 2 | 1 |
| Benzene, 2-chloro-1,3,5-trinitro- (C6H2ClN3O6) | HJRJRUMKQCMYDL-UHFFFAOYSA-N | C1=C(C=C(C(=C1[N+](=O)[O-])Cl)[N+](=O)[O-])[N+](=O)[O-] | 2779 | 0 | 1 | 2 |
| Methyl trithion (C9H12ClO2PS3) | OUCCVXVYGFBXSV-UHFFFAOYSA-N | COP(=S)(OC)SCSC1=CC=C(C=C1)Cl | 3667 | 0 | 1 | 2 |
| Dichlorphenamide (C6H6Cl2N2O4S2) | GJQPMPFPNINLKP-UHFFFAOYSA-N | C1=C(C=C(C(=C1S(=O)(=O)N)Cl)Cl)S(=O)(=O)N | 9999 | 1261 | 1 | 1 |
| Ronnel (C8H8Cl3O3PS) | JHJOOSLFWRRSGU-UHFFFAOYSA-N | COP(=S)(OC)OC1=CC(=C(C=C1Cl)Cl)Cl | 789 | 9999 | 2 | 1 |
| O,O-Dimethyl O-(2,4,5-trichloro)phenyl phosphate (C8H8Cl3O4P) | XWMMHXRGYYPFAV-UHFFFAOYSA-N | COP(=O)(OC)OC1=CC(=C(C=C1Cl)Cl)Cl | 338 | 5042 | 2 | 1 |
| 2-Chloro-3,5-dinitrobenzoic acid (C7H3ClN2O6) | ADTKEYLCJYYHHH-UHFFFAOYSA-N | C1=C(C=C(C(=C1[N+](=O)[O-])Cl)C(=O)O)[N+](=O)[O-] | 9999 | 0 | 1 | 2 |
| Acetamide, N-(2,5-dimethoxyphenyl)-2,2-dichloro- (C10H11Cl2NO3) | MRFYBJFXENFYEJ-UHFFFAOYSA-N | COC1=CC(=C(C=C1)OC)NC(=O)C(Cl)Cl | 9999 | 430 | 1 | 1 |
| O-(2,6-dichloro-p-tolyl) O,O-dimethyl thiophosphate (C9H11Cl2O3PS) | OBZIQQJJIKNWNO-UHFFFAOYSA-N | CC1=CC(=C(C(=C1)Cl)OP(=S)(OC)OC)Cl | 12 | 9999 | 2 | 1 |
| Diethyl 3-chlorophenyl phosphate (C10H14ClO4P) | OXUXCXDESUJMFE-UHFFFAOYSA-N | CCOP(=O)(OCC)OC1=CC(=CC=C1)Cl | 3493 | 0 | 1 | 2 |
| Propanamide, N-(2,5-dimethoxyphenyl)-3-chloro- (C11H14ClNO3) | QLZAYTWEEVKOOY-UHFFFAOYSA-N | COC1=CC(=C(C=C1)OC)NC(=O)CCCl | 4714 | 0 | 1 | 2 |
| Propanamide, N-(2,5-dimethoxyphenyl)-2-chloro- (C11H14ClNO3) | RMRDVDAALOHFAM-UHFFFAOYSA-N | CC(C(=O)NC1=C(C=CC(=C1)OC)OC)Cl | 6736 | 390 | 1 | 1 |
| Acetoxyacetic acid, 3,4-dichlorophenyl ester (C10H8Cl2O4) | ISETXWLPWNBHGV-UHFFFAOYSA-N | CC(=O)OCC(=O)OC1=CC(=C(C=C1)Cl)Cl | 230 | 0 | 2 | 2 |
| Silvex, methyl ester (C10H9Cl3O3) | YTAXYXOJOYIQQO-UHFFFAOYSA-N | CC(C(=O)OC)OC1=CC(=C(C=C1Cl)Cl)Cl | 3022 | 236 | 1 | 1 |
| Barban (C11H9Cl2NO2) | MCOQHIWZJUDQIC-UHFFFAOYSA-N | C1=CC(=CC(=C1)Cl)NC(=O)OCC#CCCl | 2306 | 9999 | 1 | 1 |
| Acetoxyacetic acid, (4-chlorophenyl)methyl ester (C11H11ClO4) | IUAPHMNPPYPAAC-UHFFFAOYSA-N | CC(=O)OCC(=O)OCC1=CC=C(C=C1)Cl | 100 | 0 | 2 | 2 |
| Thiobencarb (C12H16ClNOS) | QHTQREMOGMZHJV-UHFFFAOYSA-N | CCN(CC)C(=O)SCC1=CC=C(C=C1)Cl | 1594 | 0 | 1 | 2 |
| Orbencarb (C12H16ClNOS) | LLLFASISUZUJEQ-UHFFFAOYSA-N | CCN(CC)C(=O)SCC1=CC=CC=C1Cl | 100 | 3250 | 2 | 1 |
| L-Alanine, N-(2-chlorobenzoyl)-, methyl ester (C11H12ClNO3) | IYECSIXZMPHUOA-UHFFFAOYSA-N | CC(C(=O)OC)NC(=O)C1=CC=CC=C1Cl | 130 | 0 | 2 | 2 |
| p-N,N-Bis(2-chloroethyl)aminobenzoic acid (C11H13Cl2NO2) | PCLQMXVMRDPVKX-UHFFFAOYSA-N | C1=CC(=CC=C1C(=O)O)N(CCCl)CCCl | 1195 | 103 | 1 | 1 |
| 2,4-DB methyl ester (C11H12Cl2O3) | NKXSNMCGZZWMMW-UHFFFAOYSA-N | COC(=O)CCCOC1=C(C=C(C=C1)Cl)Cl | 231 | 0 | 2 | 2 |
| 2,4-D isopropyl ester (C11H12Cl2O3) | WHOKDONDRZNCBC-UHFFFAOYSA-N | CC(C)OC(=O)COC1=C(C=C(C=C1)Cl)Cl | 1611 | 60 | 1 | 2 |
| 2,3-Dichlorophenol, isoBOC (C11H12Cl2O3) | SJOZPBMTTPQALN-UHFFFAOYSA-N | CC(C)COC(=O)OC1=C(C(=CC=C1)Cl)Cl | 0 | 0 | 2 | 2 |
| Butanoic acid, 4-(2,4,5-trichlorophenoxy)- (C10H9Cl3O3) | RTWCZQFXFMXXKP-UHFFFAOYSA-N | C1=C(C(=CC(=C1Cl)Cl)Cl)OCCCC(=O)O | 1681 | 0 | 1 | 2 |
| Trichloroacetamide, N-ethyl-N-(3-methylphenyl)- (C11H12Cl3NO) | DEQXVMATOARFGZ-UHFFFAOYSA-N | CCN(C1=CC=CC(=C1)C)C(=O)C(Cl)(Cl)Cl | 1371 | 300 | 1 | 1 |
| cyanazine (C9H13ClN6) | MZZBPDKVEFVLFF-UHFFFAOYSA-N | CCNC1=NC(=NC(=N1)Cl)NC(C)(C)C#N | 4582 | 417 | 1 | 1 |
| Ipazine (C10H18ClN5) | OWYWGLHRNBIFJP-UHFFFAOYSA-N | CCN(CC)C1=NC(=NC(=N1)NC(C)C)Cl | 7729 | 450 | 1 | 1 |
| Pentanamide, N-(4-methoxyphenyl)-5-chloro- (C12H16ClNO2) | FBJIABNNCRFUEM-UHFFFAOYSA-N | COC1=CC=C(C=C1)NC(=O)CCCCCl | 2582 | 340 | 1 | 1 |
| 5-Chlorovaleric acid, 4-methoxyphenyl ester (C12H15ClO3) | KGAVHPXSHLZLNB-UHFFFAOYSA-N | COC1=CC=C(C=C1)OC(=O)CCCCCl | 280 | 0 | 2 | 2 |
| MCPB methyl ester (C12H15ClO3) | FWDQLSHRVKQKBS-UHFFFAOYSA-N | CC1=C(C=CC(=C1)Cl)OCCCC(=O)OC | 470 | 0 | 2 | 2 |
| Clofibrate (C12H15ClO3) | KNHUKKLJHYUCFP-UHFFFAOYSA-N | CCOC(=O)C(C)(C)OC1=CC=C(C=C1)Cl | 819 | 0 | 1 | 2 |
| 4-N,N-Bis(2-chloroethyl)amino-2-tolualdehyde (C12H15Cl2NO) | ZQIAXDULHBLZJE-UHFFFAOYSA-N | CC1=C(C=CC(=C1)N(CCCl)CCCl)C=O | 1144 | 148 | 1 | 1 |
| 5-Chlorovaleric acid, 3,4-dichlorophenyl ester (C11H11Cl3O2) | BYNHRZMXLGSQAV-UHFFFAOYSA-N | C1=CC(=C(C=C1OC(=O)CCCCCl)Cl)Cl | 140 | 0 | 2 | 2 |
| Propyzamide (C12H11Cl2NO) | PHNUZKMIPFFYSO-UHFFFAOYSA-N | CC(C)(C#C)NC(=O)C1=CC(=CC(=C1)Cl)Cl | 2742 | 0 | 1 | 2 |
| 5-Chlorovaleric acid, 4-cyanophenyl ester (C12H12ClNO2) | ZNTCEUKZWJLZDV-UHFFFAOYSA-N | C1=CC(=CC=C1C#N)OC(=O)CCCCCl | 50 | 0 | 2 | 2 |
| 2-Chlorobenzoic acid, hex-4-yn-3-yl ester (C13H13ClO2) | KNDBMTMCFPSMSH-UHFFFAOYSA-N | CCC(C#CC)OC(=O)C1=CC=CC=C1Cl | 120 | 210 | 2 | 1 |
| 4-Chlorobenzoic acid, hex-4-yn-3-yl ester (C13H13ClO2) | AZSLZDAEKLKXKB-UHFFFAOYSA-N | CCC(C#CC)OC(=O)C1=CC=C(C=C1)Cl | 120 | 0 | 2 | 2 |
| 6-Chlorohexanoic acid, 3-methylphenyl ester (C13H17ClO2) | ZRXIFYCSKIEIGD-UHFFFAOYSA-N | CC1=CC(=CC=C1)OC(=O)CCCCCCl | 641 | 0 | 2 | 2 |
| Hexyl 4-chlorobenzoate (C13H17ClO2) | WNIDDGDBCDWAIE-UHFFFAOYSA-N | CCCCCCOC(=O)C1=CC=C(C=C1)Cl | 0 | 0 | 2 | 2 |
| Benzoic acid, 2-chloro, hexyl ester (C13H17ClO2) | LWXVSNHMEABLCY-UHFFFAOYSA-N | CCCCCCOC(=O)C1=CC=CC=C1Cl | 180 | 0 | 2 | 2 |
| 5-Chlorovaleric acid, 3,5-dimethylphenyl ester (C13H17ClO2) | ZZLQVNHJJPBAPS-UHFFFAOYSA-N | CC1=CC(=CC(=C1)OC(=O)CCCCCl)C | 200 | 0 | 2 | 2 |
| Monalide (C13H18ClNO) | KXGYBSNVFXBPNO-UHFFFAOYSA-N | CCCC(C)(C)C(=O)NC1=CC=C(C=C1)Cl | 670 | 10 | 2 | 2 |
| Pentanochlor (C13H18ClNO) | WGVWLKXZBUVUAM-UHFFFAOYSA-N | CCCC(C)C(=O)NC1=CC(=C(C=C1)C)Cl | 1470 | 40 | 1 | 2 |
| Semustine (C10H18ClN3O2) | FVLVBPDQNARYJU-UHFFFAOYSA-N | CC1CCC(CC1)NC(=O)N(CCCl)N=O | 58 | 0 | 2 | 2 |
| L-Proline, N-(5-chlorovaleryl)-, methyl ester (C11H18ClNO3) | OBAXQHFPWSIRJV-UHFFFAOYSA-N | COC(=O)C1CCCN1C(=O)CCCCCl | 70 | 521 | 2 | 1 |
| 1-Chloro-9-phenylnonane (C15H23Cl) | KFJDHWCTDWNJTP-UHFFFAOYSA-N | C1=CC=C(C=C1)CCCCCCCCCCl | 746 | 0 | 2 | 2 |
| 1-Chloromethyl-3,5-bis(1,1-dimethylethyl)benzene (C15H23Cl) | UNRGFCVSCXJGCL-UHFFFAOYSA-N | CC(C)(C)C1=CC(=CC(=C1)CCl)C(C)(C)C | 1700 | 570 | 1 | 1 |
| Octachlorostyrene (C8Cl8) | RUYUCCQRWINUHE-UHFFFAOYSA-N | C1(=C(C(=C(C(=C1Cl)Cl)Cl)Cl)Cl)C(=C(Cl)Cl)Cl | 2751 | 3254 | 1 | 1 |
| 4-Nitrobenzoic acid, 3-chloroprop-2-enyl ester (C10H8ClNO4) | LIVUUKNGTUNKGJ-LZCJLJQNSA-N | C1=CC(=CC=C1C(=O)OC/C=C/Cl)[N+](=O)[O-] | 0 | 2082 | 2 | 1 |
| Methazole (C9H6Cl2N2O3) | LRUUNMYPIBZBQH-UHFFFAOYSA-N | CN1C(=O)N(OC1=O)C2=CC(=C(C=C2)Cl)Cl | 7129 | 0 | 1 | 2 |
| Anilazine (C9H5Cl3N4) | IMHBYKMAHXWHRP-UHFFFAOYSA-N | C1=CC=C(C(=C1)NC2=NC(=NC(=N2)Cl)Cl)Cl | 940 | 9999 | 1 | 1 |
| 4-Amino-2-chloro-6,7-dimethoxyquinazoline (C10H10ClN3O2) | HWIIAAVGRHKSOJ-UHFFFAOYSA-N | COC1=C(C=C2C(=C1)C(=NC(=N2)Cl)N)OC | 9999 | 782 | 1 | 1 |
| 2,4-Dichloro-6,7-dimethoxyquinazoline (C10H8Cl2N2O2) | DGHKCBSVAZXEPP-UHFFFAOYSA-N | COC1=C(C=C2C(=C1)C(=NC(=N2)Cl)Cl)OC | 9999 | 254 | 1 | 1 |
| Naphthalene, 1,2,3,6,7,8-hexachloro (C10H2Cl6) | WJYZNPLWZGYFIE-UHFFFAOYSA-N | C1=C2C=C(C(=C(C2=C(C(=C1Cl)Cl)Cl)Cl)Cl)Cl | 297 | 0 | 2 | 2 |
| Sulphenone (C12H9ClO2S) | OFCFYWOKHPOXKF-UHFFFAOYSA-N | C1=CC=C(C=C1)S(=O)(=O)C2=CC=C(C=C2)Cl | 2959 | 0 | 1 | 2 |
| Carbamic chloride, diphenyl- (C13H10ClNO) | XNBKKRFABABBPM-UHFFFAOYSA-N | C1=CC=C(C=C1)N(C2=CC=CC=C2)C(=O)Cl | 6619 | 9999 | 1 | 1 |
| Disulfide, bis(4-chlorophenyl) (C12H8Cl2S2) | ZIXXRXGPBFMPFD-UHFFFAOYSA-N | C1=CC(=CC=C1SSC2=CC=C(C=C2)Cl)Cl | 7626 | 104 | 1 | 1 |
| Sulfoxide, bis(p-chlorophenyl) (C12H8Cl2OS) | KJGYFISADIZFEL-UHFFFAOYSA-N | C1=CC(=CC=C1S(=O)C2=CC=C(C=C2)Cl)Cl | 4369 | 259 | 1 | 1 |
| Diazene, bis(2-chlorophenyl)- (C12H8Cl2N2) | FIQUJBRQBIUISO-UHFFFAOYSA-N | C1=CC=C(C(=C1)N=NC2=CC=CC=C2Cl)Cl | 3899 | 0 | 1 | 2 |
| Nicotinamide, N-(3-chlorophenyl)- (C12H9ClN2O) | XQPPPWLPXGVYAJ-UHFFFAOYSA-N | C1=CC(=CC(=C1)Cl)NC(=O)C2=CN=CC=C2 | 3723 | 0 | 1 | 2 |
| Acetamide, N-(1-naphthyl)-2,2-dichloro- (C12H9Cl2NO) | SJVSQQFAACIPOJ-UHFFFAOYSA-N | C1=CC=C2C(=C1)C=CC=C2NC(=O)C(Cl)Cl | 8037 | 430 | 1 | 1 |
| Dichloroacetic acid, 2-naphthyl ester (C12H8Cl2O2) | BINBEEOLDVVDPS-UHFFFAOYSA-N | C1=CC=C2C=C(C=CC2=C1)OC(=O)C(Cl)Cl | 1792 | 0 | 1 | 2 |
| Benzoic acid, 4-chlorophenyl ester (C13H9ClO2) | JKSIXXOEIXUYFW-UHFFFAOYSA-N | C1=CC=C(C=C1)C(=O)OC2=CC=C(C=C2)Cl | 220 | 0 | 2 | 2 |
| Benzoic acid, 2-chloro-, phenyl ester (C13H9ClO2) | RBKABBZAJGDBFA-UHFFFAOYSA-N | C1=CC=C(C=C1)OC(=O)C2=CC=CC=C2Cl | 350 | 0 | 2 | 2 |
| Chlorbenzide (C13H10Cl2S) | ZHLKXBJTJHRTTE-UHFFFAOYSA-N | C1=CC(=CC=C1CSC2=CC=C(C=C2)Cl)Cl | 1317 | 0 | 1 | 2 |
| 3,3'-Dichlorobenzidine (C12H10Cl2N2) | HUWXDEQWWKGHRV-UHFFFAOYSA-N | C1=CC(=C(C=C1C2=CC(=C(C=C2)N)Cl)Cl)N | 9999 | 420 | 1 | 1 |
| PCB 54 (C12H6Cl4) | PXAGFNRKXSYIHU-UHFFFAOYSA-N | C1=CC(=C(C(=C1)Cl)C2=C(C=CC=C2Cl)Cl)Cl | 8076 | 0 | 1 | 2 |
| PCB 47 (C12H6Cl4) | QORAVNMWUNPXAO-UHFFFAOYSA-N | C1=CC(=C(C=C1Cl)Cl)C2=C(C=C(C=C2)Cl)Cl | 7959 | 1690 | 1 | 1 |
| PCB 66 (C12H6Cl4) | RKLLTEAEZIJBAU-UHFFFAOYSA-N | C1=CC(=C(C=C1C2=C(C=C(C=C2)Cl)Cl)Cl)Cl | 7827 | 78 | 1 | 2 |
| PCB 70 (C12H6Cl4) | KENZYIHFBRWMOD-UHFFFAOYSA-N | C1=CC(=C(C=C1C2=C(C=CC(=C2)Cl)Cl)Cl)Cl | 7790 | 74 | 1 | 2 |
| PCB 75 (C12H6Cl4) | RZFZBHKDGHISSH-UHFFFAOYSA-N | C1=CC(=CC=C1C2=C(C=C(C=C2Cl)Cl)Cl)Cl | 8080 | 201 | 1 | 1 |
| PCB 77 (C12H6Cl4) | UQMGJOKDKOLIDP-UHFFFAOYSA-N | C1=CC(=C(C=C1C2=CC(=C(C=C2)Cl)Cl)Cl)Cl | 7470 | 99 | 1 | 2 |
| PCB 74 (C12H6Cl4) | TULCXSBAPHCWCF-UHFFFAOYSA-N | C1=CC(=CC=C1C2=CC(=C(C=C2Cl)Cl)Cl)Cl | 7942 | 127 | 1 | 1 |
| PCB 60 (C12H6Cl4) | XLDBTRJKXLKYTC-UHFFFAOYSA-N | C1=CC(=CC=C1C2=C(C(=C(C=C2)Cl)Cl)Cl)Cl | 7383 | 127 | 1 | 1 |
| PCB 80 (C12H6Cl4) | UTMWFJSRHLYRPY-UHFFFAOYSA-N | C1=C(C=C(C=C1Cl)Cl)C2=CC(=CC(=C2)Cl)Cl | 8250 | 53 | 1 | 2 |
| PCB 61 (C12H6Cl4) | HLQDGCWIOSOMDP-UHFFFAOYSA-N | C1=CC=C(C=C1)C2=CC(=C(C(=C2Cl)Cl)Cl)Cl | 8062 | 41 | 1 | 2 |
| PCB 65 (C12H6Cl4) | BLAYIQLVUNIICD-UHFFFAOYSA-N | C1=CC=C(C=C1)C2=C(C(=CC(=C2Cl)Cl)Cl)Cl | 7991 | 227 | 1 | 1 |
| PCB 42 (C12H6Cl4) | ALFHIHDQSYXSGP-UHFFFAOYSA-N | C1=CC(=C(C(=C1)Cl)Cl)C2=C(C=C(C=C2)Cl)Cl | 8029 | 2478 | 1 | 1 |
| PCB 40 (C12H6Cl4) | VTLYHLREPCPDKX-UHFFFAOYSA-N | C1=CC(=C(C(=C1)Cl)Cl)C2=C(C(=CC=C2)Cl)Cl | 6552 | 5099 | 1 | 1 |
| PCB 44 (C12H6Cl4) | ALDJIKXAHSDLLB-UHFFFAOYSA-N | C1=CC(=C(C(=C1)Cl)Cl)C2=C(C=CC(=C2)Cl)Cl | 7793 | 3338 | 1 | 1 |
| PCB 49 (C12H6Cl4) | ZWPVHELAQPIZHO-UHFFFAOYSA-N | C1=CC(=C(C=C1Cl)Cl)C2=C(C=CC(=C2)Cl)Cl | 6330 | 3610 | 1 | 1 |
| PCB 53 (C12H6Cl4) | SFTUSTXGTCCSHX-UHFFFAOYSA-N | C1=CC(=C(C(=C1)Cl)C2=C(C=CC(=C2)Cl)Cl)Cl | 8736 | 3800 | 1 | 1 |
| PCB 72 (C12H6Cl4) | WBTMFEPLVQOWFI-UHFFFAOYSA-N | C1=CC(=C(C=C1Cl)C2=CC(=CC(=C2)Cl)Cl)Cl | 7765 | 200 | 1 | 1 |
| PCB 56 (C12H6Cl4) | UNCGJRRROFURDV-UHFFFAOYSA-N | C1=CC(=C(C(=C1)Cl)Cl)C2=CC(=C(C=C2)Cl)Cl | 9837 | 133 | 1 | 1 |
| PCB 71 (C12H6Cl4) | WYVBETQIUHPLFO-UHFFFAOYSA-N | C1=CC(=C(C(=C1)Cl)C2=CC(=C(C=C2)Cl)Cl)Cl | 6974 | 166 | 1 | 1 |
| PCB 46 (C12H6Cl4) | CUGLICQCTXWQNF-UHFFFAOYSA-N | C1=CC(=C(C(=C1)Cl)Cl)C2=C(C=CC=C2Cl)Cl | 5630 | 5420 | 1 | 1 |
| PCB 79 (C12H6Cl4) | QLCTXEMDCZGPCG-UHFFFAOYSA-N | C1=CC(=C(C=C1C2=CC(=CC(=C2)Cl)Cl)Cl)Cl | 8008 | 54 | 1 | 2 |
| PCB 58 (C12H6Cl4) | IOPBNBSKOPJKEG-UHFFFAOYSA-N | C1=CC(=C(C(=C1)Cl)Cl)C2=CC(=CC(=C2)Cl)Cl | 7731 | 136 | 1 | 1 |
| PCB 64 (C12H6Cl4) | FXRXQYZZALWWGA-UHFFFAOYSA-N | C1=CC(=CC=C1C2=C(C=CC(=C2Cl)Cl)Cl)Cl | 7751 | 99 | 1 | 2 |
| PCB 41 (C12H6Cl4) | SEWHDNLIHDBVDZ-UHFFFAOYSA-N | C1=CC=C(C(=C1)C2=C(C(=C(C=C2)Cl)Cl)Cl)Cl | 8709 | 3746 | 1 | 1 |
| PCB 69 (C12H6Cl4) | CKUBKYSLNCKBOI-UHFFFAOYSA-N | C1=CC(=CC(=C1)Cl)C2=C(C=C(C=C2Cl)Cl)Cl | 7890 | 138 | 1 | 1 |
| PCB 50 (C12H6Cl4) | VLLVVZDKBSYMCG-UHFFFAOYSA-N | C1=CC=C(C(=C1)C2=C(C=C(C=C2Cl)Cl)Cl)Cl | 6100 | 3535 | 1 | 1 |
| PCB 51 (C12H6Cl4) | WVHNUGRFECMVLQ-UHFFFAOYSA-N | C1=CC(=C(C(=C1)Cl)C2=C(C=C(C=C2)Cl)Cl)Cl | 7108 | 2663 | 1 | 1 |
| 1,1'-Biphenyl, 2,2',3,6-tetrachloro (C12H6Cl4) | VHGHHZZTMJLTJX-UHFFFAOYSA-N | C1=CC=C(C(=C1)C2=C(C=CC(=C2Cl)Cl)Cl)Cl | 7300 | 3604 | 1 | 1 |
| PCB 43 (C12H6Cl4) | NRBNBYFPJCCKTO-UHFFFAOYSA-N | C1=CC=C(C(=C1)C2=CC(=CC(=C2Cl)Cl)Cl)Cl | 7405 | 3797 | 1 | 1 |
| PCB 81 (C12H6Cl4) | BHWVLZJTVIYLIV-UHFFFAOYSA-N | C1=CC(=CC=C1C2=CC(=C(C(=C2)Cl)Cl)Cl)Cl | 8515 | 189 | 1 | 1 |
| PCB 55 (C12H6Cl4) | ZKGSEEWIVLAUNH-UHFFFAOYSA-N | C1=CC(=CC(=C1)Cl)C2=C(C(=C(C=C2)Cl)Cl)Cl | 7921 | 119 | 1 | 1 |
| Morpholine, 4-(4-chloro-2-nitrophenyl) (C10H11ClN2O3) | KJISHWYSNGQNFM-UHFFFAOYSA-N | C1COCCN1C2=C(C=C(C=C2)Cl)[N+](=O)[O-] | 8679 | 1809 | 1 | 1 |
| Folpet (C9H4Cl3NO2S) | HKIOYBQGHSTUDB-UHFFFAOYSA-N | C1=CC=C2C(=C1)C(=O)N(C2=O)SC(Cl)(Cl)Cl | 2040 | 9999 | 1 | 1 |
| 2-Propanol, 1-chloro-3-(1-naphthalenyloxy)- (C13H13ClO2) | ZVVJOJMCXYDDEW-UHFFFAOYSA-N | C1=CC=C2C(=C1)C=CC=C2OCC(CCl)O | 1491 | 0 | 1 | 2 |
| Propanamide, N-(1-naphthyl)-3-chloro- (C13H12ClNO) | ISEAPHXDUWABSS-UHFFFAOYSA-N | C1=CC=C2C(=C1)C=CC=C2NC(=O)CCCl | 2742 | 50 | 1 | 2 |
| Propanamide, N-(1-naphthyl)-2-chloro- (C13H12ClNO) | HZEXEFBFZFWVOD-UHFFFAOYSA-N | CC(C(=O)NC1=CC=CC2=CC=CC=C21)Cl | 5525 | 500 | 1 | 1 |
| Benzophenone, 2-amino-5-chloro- (C13H10ClNO) | ZUWXHHBROGLWNH-UHFFFAOYSA-N | C1=CC=C(C=C1)C(=O)C2=C(C=CC(=C2)Cl)N | 6686 | 320 | 1 | 1 |
| Methanone, (4-chlorophenyl)(4-hydroxyphenyl)- (C13H9ClO2) | RUETVLNXAGWCDS-UHFFFAOYSA-N | C1=CC(=CC=C1C(=O)C2=CC=C(C=C2)Cl)O | 3669 | 869 | 1 | 1 |
| 4,4'-Dichlorobenzhydrol (C13H10Cl2O) | PHUYGURFBULKPA-UHFFFAOYSA-N | C1=CC(=CC=C1C(C2=CC=C(C=C2)Cl)O)Cl | 990 | 890 | 1 | 1 |
| Methanone, (3-chlorophenyl)(4-chlorophenyl)- (C13H8Cl2O) | XFTXWDDQIXYAGY-UHFFFAOYSA-N | C1=CC(=CC(=C1)Cl)C(=O)C2=CC=C(C=C2)Cl | 2813 | 100 | 1 | 2 |
| 2,4'-Dichlorobenzophenone (C13H8Cl2O) | YXMYPHLWXBXNFF-UHFFFAOYSA-N | C1=CC=C(C(=C1)C(=O)C2=CC=C(C=C2)Cl)Cl | 2139 | 209 | 1 | 1 |
| Benzophenone, 4,4'-dichloro- (C13H8Cl2O) | OKISUZLXOYGIFP-UHFFFAOYSA-N | C1=CC(=CC=C1C(=O)C2=CC=C(C=C2)Cl)Cl | 3693 | 1061 | 1 | 1 |
| Drazoxolon (C10H8ClN3O2) | OOTHTARUZHONSW-UHFFFAOYSA-N | CC1=NOC(=O)C1=NNC2=CC=CC=C2Cl | 1880 | 0 | 1 | 2 |
| Benoxacor (C11H11Cl2NO2) | PFJJMJDEVDLPNE-UHFFFAOYSA-N | CC1COC2=CC=CC=C2N1C(=O)C(Cl)Cl | 2270 | 310 | 1 | 1 |
| Diphenylacetyl chloride (C14H11ClO) | MSYLETHDEIJMAF-UHFFFAOYSA-N | C1=CC=C(C=C1)C(C2=CC=CC=C2)C(=O)Cl | 0 | 500 | 2 | 1 |
| Ethane, 1,1-bis(p-chlorophenyl)- (C14H12Cl2) | KTEARTXATWOYDB-UHFFFAOYSA-N | CC(C1=CC=C(C=C1)Cl)C2=CC=C(C=C2)Cl | 3700 | 1600 | 1 | 1 |
| Tridiphane (C10H7Cl5O) | IBZHOAONZVJLOB-UHFFFAOYSA-N | C1C(O1)(CC(Cl)(Cl)Cl)C2=CC(=CC(=C2)Cl)Cl | 1059 | 1877 | 1 | 1 |
| Benzamide, N-tetrahydrofurfuryl-2-chloro- (C12H14ClNO2) | HOJRGAVTPWHWKA-UHFFFAOYSA-N | C1CC(OC1)CNC(=O)C2=CC=CC=C2Cl | 100 | 50 | 2 | 2 |
| Benzamide, N-tetrahydrofurfuryl-4-chloro- (C12H14ClNO2) | ZMMAVVGZVPYQOX-UHFFFAOYSA-N | C1CC(OC1)CNC(=O)C2=CC=C(C=C2)Cl | 130 | 0 | 2 | 2 |
| Clomazone (C12H14ClNO2) | KIEDNEWSYUYDSN-UHFFFAOYSA-N | CC1(CON(C1=O)CC2=CC=CC=C2Cl)C | 77 | 6735 | 2 | 1 |
| Cyclopentanecarboxylic acid, 3,4-dichlorophenyl ester (C12H12Cl2O2) | CIKRVIKIBNQLQZ-UHFFFAOYSA-N | C1CCC(C1)C(=O)OC2=CC(=C(C=C2)Cl)Cl | 380 | 0 | 2 | 2 |
| Cyclohexanecarboxamide, N-(3-chlorophenyl)- (C13H16ClNO) | WGUWTOYIQWKLDD-UHFFFAOYSA-N | C1CCC(CC1)C(=O)NC2=CC(=CC=C2)Cl | 3013 | 0 | 1 | 2 |
| 1-(4-Chlorophenyl)-1-cyclohexane-carboxylic acid (C13H15ClO2) | UPNXUJXIIZGXLQ-UHFFFAOYSA-N | C1CCC(CC1)(C2=CC=C(C=C2)Cl)C(=O)O | 1411 | 0 | 1 | 2 |
| Tranid (C10H12ClN3O2) | QCQPGRMMDFIQMB-UHFFFAOYSA-N | CNC(=O)ON=C1C2CC(C1Cl)CC2C#N | 0 | 0 | 2 | 2 |
| 1,1-Bis(4-chlorophenyl)ethylene (C14H10Cl2) | IEAUXBMXWDAYID-UHFFFAOYSA-N | C=C(C1=CC=C(C=C1)Cl)C2=CC=C(C=C2)Cl | 3800 | 3000 | 1 | 1 |
| Diclomezine (C11H8Cl2N2O) | UWQMKVBQKFHLCE-UHFFFAOYSA-N | CC1=C(C=C(C=C1Cl)C2=NNC(=O)C=C2)Cl | 9999 | 2142 | 1 | 1 |
| Captan (C9H8Cl3NO2S) | LDVVMCZRFWMZSG-UHFFFAOYSA-N | C1C=CCC2C1C(=O)N(C2=O)SC(Cl)(Cl)Cl | 30 | 420 | 2 | 1 |
| 5,5,6-exo-8,9,10-hexachlorocamphene (C10H10Cl6) | UCEQMZNYZROTLW-UQCOIBPSSA-N | C1C2C(C(C1/C(=C/Cl)/C2(CCl)CCl)(Cl)Cl)Cl | 1084 | 1377 | 1 | 1 |
| 2-Thiophenecarboxylic acid, 3,4-dichlorophenyl ester (C11H6Cl2O2S) | BTWJLTDNAKWSDS-UHFFFAOYSA-N | C1=CSC(=C1)C(=O)OC2=CC(=C(C=C2)Cl)Cl | 380 | 0 | 2 | 2 |
| Acetamide, N-(3-chlorophenyl)-2-(2-thienyl)- (C12H10ClNOS) | RTZYPCPHOITSSY-UHFFFAOYSA-N | C1=CC(=CC(=C1)Cl)NC(=O)CC2=CC=CS2 | 4724 | 0 | 1 | 2 |
| 2-Thiopheneacetic acid, 4-chlorophenyl ester (C12H9ClO2S) | JKMNFPKKWDGILK-UHFFFAOYSA-N | C1=CSC(=C1)CC(=O)OC2=CC=C(C=C2)Cl | 240 | 0 | 2 | 2 |
| Dibenzo[b,e][1,4]dioxin, 2,3-dichloro- (C12H6Cl2O2) | YCIYTXRUZSDMRZ-UHFFFAOYSA-N | C1=CC=C2C(=C1)OC3=CC(=C(C=C3O2)Cl)Cl | 9999 | 596 | 1 | 1 |
| Dibenzo[b,E][1,4]dioxin, 2,7-dichloro- (C12H6Cl2O2) | NBFMTHWVRBOVPE-UHFFFAOYSA-N | C1=CC2=C(C=C1Cl)OC3=C(O2)C=C(C=C3)Cl | 9999 | 984 | 1 | 1 |
| Anthracene, 9,10-dichloro- (C14H8Cl2) | FKDIWXZNKAZCBY-UHFFFAOYSA-N | C1=CC=C2C(=C1)C(=C3C=CC=CC3=C2Cl)Cl | 9999 | 859 | 1 | 1 |
| 9-(Chloromethyl)anthracene (C15H11Cl) | PCVRSXXPGXRVEZ-UHFFFAOYSA-N | C1=CC=C2C(=C1)C=C3C=CC=CC3=C2CCl | 1823 | 9999 | 1 | 1 |
| 4-Chloro-1,8-naphthalic anhydride (C12H5ClO3) | UJEUBSWHCGDJQU-UHFFFAOYSA-N | C1=CC2=C(C=CC3=C2C(=C1)C(=O)OC3=O)Cl | 6636 | 80 | 1 | 2 |
| Chlordene (C10H6Cl6) | XCJXQCUJXDUNDN-UHFFFAOYSA-N | C1C=CC2C1C3(C(=C(C2(C3(Cl)Cl)Cl)Cl)Cl)Cl | 237 | 124 | 2 | 1 |
| α-Chlordene (C10H6Cl6) | GSNLXLNDMLYEEK-UHFFFAOYSA-N | C1C2C3C(C1Cl)C(=C(C2(C(=C3Cl)Cl)Cl)Cl)Cl | 1879 | 5941 | 1 | 1 |
| β-Chlordene (C10H6Cl6) | OSFPUJNCRLXHDW-UHFFFAOYSA-N | C1C2C3C(C1C(=C(C3(C(=C2Cl)Cl)Cl)Cl)Cl)Cl | 1721 | 2942 | 1 | 1 |
| γ-Chlordene (C10H6Cl6) | SVGYVZYRIHEIML-UHFFFAOYSA-N | C1C2C(C3C1C(C(=C2Cl)Cl)(C(=C3Cl)Cl)Cl)Cl | 4128 | 6105 | 1 | 1 |
| 2,1-Benzisoxazole, 5-chloro-3-phenyl- (C13H8ClNO) | MUHJZJKVEQASGY-UHFFFAOYSA-N | C1=CC=C(C=C1)C2=C3C=C(C=CC3=NO2)Cl | 8521 | 7708 | 1 | 1 |
| Tris(2-chloro-1-methylethyl) phosphate (C9H18Cl3O4P) | KVMPUXDNESXNOH-UHFFFAOYSA-N | CC(CCl)OP(=O)(OC(C)CCl)OC(C)CCl | 0 | 370 | 2 | 1 |
| L-Methionine, N-(5-chlorovaleryl)-, methyl ester (C11H20ClNO3S) | IQLBEONWSITFOW-UHFFFAOYSA-N | COC(=O)C(CCSC)NC(=O)CCCCCl | 0 | 120 | 2 | 1 |
| Succinic acid, butyl 2,2,2-trichloroethyl ester (C10H15Cl3O4) | RBGALIXBFKCRQF-UHFFFAOYSA-N | CCCCOC(=O)CCC(=O)OCC(Cl)(Cl)Cl | 0 | 0 | 2 | 2 |
| Succinic acid, isobutyl 2,2,2-trichloroethyl ester (C10H15Cl3O4) | MGZOIMXKEQGDAC-UHFFFAOYSA-N | CC(C)COC(=O)CCC(=O)OCC(Cl)(Cl)Cl | 0 | 0 | 2 | 2 |
| D-Alanine, N-(5-chlorovaleryl)-, butyl ester (C12H22ClNO3) | FSJRFDUSDJCEDT-UHFFFAOYSA-N | CCCCOC(=O)C(C)NC(=O)CCCCCl | 0 | 400 | 2 | 1 |
| Malonic acid, 2-chloropropyl hexyl ester (C12H21ClO4) | XHGYTOCDBVKYCB-UHFFFAOYSA-N | CCCCCCOC(=O)CC(=O)OCC(C)Cl | 0 | 0 | 2 | 2 |
| L-Leucine, N-(5-chlorovaleryl)-, methyl ester (C12H22ClNO3) | DWVUQKRUDKYQKT-UHFFFAOYSA-N | CC(C)CC(C(=O)OC)NC(=O)CCCCCl | 0 | 310 | 2 | 1 |
| D-Alanine, N-(5-chlorovaleryl)-, isobutyl ester (C12H22ClNO3) | ZYJVGFIIKFSXSY-UHFFFAOYSA-N | CC(C)COC(=O)C(C)NC(=O)CCCCCl | 0 | 420 | 2 | 1 |
| Decyl trichloroacetate (C12H21Cl3O2) | JVOWHDCGJSEUMO-UHFFFAOYSA-N | CCCCCCCCCCOC(=O)C(Cl)(Cl)Cl | 0 | 0 | 2 | 2 |
| Propanamide, N-decyl-N-methyl-3-chloro- (C14H28ClNO) | VFWVCQWSWNBFSU-UHFFFAOYSA-N | CCCCCCCCCCN(C)C(=O)CCCl | 50 | 4154 | 2 | 1 |
| Chloroacetamide, N,N-dihexyl- (C14H28ClNO) | ZGQVQRKCUZEQLW-UHFFFAOYSA-N | CCCCCCN(CCCCCC)C(=O)CCl | 60 | 5305 | 2 | 1 |
| Hexanamide, N,N-dibutyl-6-chloro- (C14H28ClNO) | DLKRTZPVPJCJBW-UHFFFAOYSA-N | CCCCN(CCCC)C(=O)CCCCCCl | 100 | 490 | 2 | 1 |
| Propanamide, N-decyl-N-methyl-2-chloro- (C14H28ClNO) | LJUWACZMRIHSHY-UHFFFAOYSA-N | CCCCCCCCCCN(C)C(=O)C(C)Cl | 170 | 5445 | 2 | 1 |
| 11-Chlorododecanoic acid, chloromethyl ester (C13H24Cl2O2) | SWJDHWAMRFKAJU-UHFFFAOYSA-N | CC(CCCCCCCCCC(=O)OCCl)Cl | 0 | 0 | 2 | 2 |
| 10-Chlorododecanoic acid, chloromethyl ester (C13H24Cl2O2) | CKRGKKRODIRREL-UHFFFAOYSA-N | CCC(CCCCCCCCC(=O)OCCl)Cl | 0 | 0 | 2 | 2 |
| 9-Chlorododecanoic acid, chloromethyl ester (C13H24Cl2O2) | NXKVVDSCGLSSDG-UHFFFAOYSA-N | CCCC(CCCCCCCC(=O)OCCl)Cl | 0 | 0 | 2 | 2 |
| 3-Chlorododecanoic acid, chloromethyl ester (C13H24Cl2O2) | GQHFPKLBEGPYQL-UHFFFAOYSA-N | CCCCCCCCCC(CC(=O)OCCl)Cl | 0 | 5685 | 2 | 1 |
| 4-Chlorododecanoic acid, chloromethyl ester (C13H24Cl2O2) | ZXYVRYZSZYAFKI-UHFFFAOYSA-N | CCCCCCCCC(CCC(=O)OCCl)Cl | 0 | 0 | 2 | 2 |
| 5-Chlorododecanoic acid, chloromethyl ester (C13H24Cl2O2) | NHSXKNLOSXLLRZ-UHFFFAOYSA-N | CCCCCCCC(CCCC(=O)OCCl)Cl | 0 | 0 | 2 | 2 |
| 6-Chlorododecanoic acid, chloromethyl ester (C13H24Cl2O2) | JPUSQOKDHBAWPK-UHFFFAOYSA-N | CCCCCCC(CCCCC(=O)OCCl)Cl | 0 | 0 | 2 | 2 |
| 7-Chlorododecanoic acid, chloromethyl ester (C13H24Cl2O2) | FTELUGWCTDTFNA-UHFFFAOYSA-N | CCCCCC(CCCCCC(=O)OCCl)Cl | 0 | 0 | 2 | 2 |
| 8-Chlorododecanoic acid, chloromethyl ester (C13H24Cl2O2) | JVKMUBVORYFQKQ-UHFFFAOYSA-N | CCCCC(CCCCCCC(=O)OCCl)Cl | 0 | 0 | 2 | 2 |
| 5-chlorovaleric acid, 2-methyloct-5-yn-4-yl ester (C14H23ClO2) | LGSSOHRMKNDPBM-UHFFFAOYSA-N | CCC#CC(CC(C)C)OC(=O)CCCCCl | 210 | 150 | 2 | 1 |
| 5-chlorovaleric acid, nonyl ester (C14H27ClO2) | ZMLMYLLZBXHXHL-UHFFFAOYSA-N | CCCCCCCCCOC(=O)CCCCCl | 0 | 0 | 2 | 2 |
| Dodecyl chloroacetate (C14H27ClO2) | JPPYCWJDINILKY-UHFFFAOYSA-N | CCCCCCCCCCCCOC(=O)CCl | 0 | 0 | 2 | 2 |
| 2-chloroethyl dodecanoate (C14H27ClO2) | PPRUSMUBWUQYRY-UHFFFAOYSA-N | CCCCCCCCCCCC(=O)OCCCl | 450 | 0 | 2 | 2 |
| Propanoic acid, 3-chloro, undecyl ester (C14H27ClO2) | MEBJHQDVCFHKMD-UHFFFAOYSA-N | CCCCCCCCCCCOC(=O)CCCl | 0 | 480 | 2 | 1 |
| 1-Chlorohexadecane (C16H33Cl) | CLWAXFZCVYJLLM-UHFFFAOYSA-N | CCCCCCCCCCCCCCCCCl | 129 | 20 | 2 | 2 |
| Fumaric acid, butyl 2,2,2-trichloroethyl ester (C10H13Cl3O4) | ZAVMYKKEQJIFPH-SNAWJCMRSA-N | CCCCOC(=O)/C=C/C(=O)OCC(Cl)(Cl)Cl | 0 | 490 | 2 | 1 |
| Fumaric acid, isobutyl 2,2,2-trichloroethyl ester (C10H13Cl3O4) | VYCVEXWSKPJYKF-ONEGZZNKSA-N | CC(C)COC(=O)/C=C/C(=O)OCC(Cl)(Cl)Cl | 0 | 1111 | 2 | 1 |
| Fumaric acid, 2,2-dichloroethyl pentyl ester (C11H16Cl2O4) | XMQDRXJXGJNOAC-AATRIKPKSA-N | CCCCCOC(=O)/C=C/C(=O)OCC(Cl)Cl | 0 | 90 | 2 | 2 |
| Trichloroacetic acid, 2,7-dimethyloct-7-en-5-yn-4-yl ester (C12H15Cl3O2) | XXRBSODQKFUODU-UHFFFAOYSA-N | CC(C)CC(C#CC(=C)C)OC(=O)C(Cl)(Cl)Cl | 270 | 1902 | 2 | 1 |
| Dichloroacetic acid, 2,6-dimethylnon-1-en-3-yn-5-yl ester (C13H18Cl2O2) | WQHIJAZVGMYLTQ-UHFFFAOYSA-N | CCCC(C)C(C#CC(=C)C)OC(=O)C(Cl)Cl | 0 | 400 | 2 | 1 |
| Dichloroacetic acid, undec-2-enyl ester (C13H22Cl2O2) | CWGSMVDQCFKHOS-MDZDMXLPSA-N | CCCCCCCC/C=C/COC(=O)C(Cl)Cl | 30 | 80 | 2 | 2 |
| 3-Chloropropionic acid, 2,6-dimethylnon-1-en-3-yn-5-yl ester (C14H21ClO2) | NOIASPFJFXMZDP-UHFFFAOYSA-N | CCCC(C)C(C#CC(=C)C)OC(=O)CCCl | 80 | 30 | 2 | 2 |
| 3-Chloropropionic acid, undec-2-enyl ester (C14H25ClO2) | SRHPHYGOUXFJTB-MDZDMXLPSA-N | CCCCCCCC/C=C/COC(=O)CCCl | 0 | 300 | 2 | 1 |
| Dicapthon (C8H9ClNO5PS) | OTKXWJHPGBRXCR-UHFFFAOYSA-N | COP(=S)(OC)OC1=C(C=C(C=C1)[N+](=O)[O-])Cl | 0 | 9999 | 2 | 1 |
| Chlorthion (C8H9ClNO5PS) | NZNRRXXETLSZRO-UHFFFAOYSA-N | COP(=S)(OC)OC1=CC(=C(C=C1)[N+](=O)[O-])Cl | 4013 | 0 | 1 | 2 |
| Acetamide, N-(2,5-dimethoxyphenyl)-2,2,2-trichloro- (C10H10Cl3NO3) | MXINBMRGIGQXDX-UHFFFAOYSA-N | COC1=CC(=C(C=C1)OC)NC(=O)C(Cl)(Cl)Cl | 4764 | 691 | 1 | 1 |
| Chlorpropamide (C10H13ClN2O3S) | RKWGIWYCVPQPMF-UHFFFAOYSA-N | CCCNC(=O)NS(=O)(=O)C1=CC=C(C=C1)Cl | 40 | 0 | 2 | 2 |
| Meclofenoxate (C12H16ClNO3) | XZTYGFHCIAKPGJ-UHFFFAOYSA-N | CN(C)CCOC(=O)COC1=CC=C(C=C1)Cl | 50 | 0 | 2 | 2 |
| Dichlofenthion (C10H13Cl2O3PS) | WGOWCPGHOCIHBW-UHFFFAOYSA-N | CCOP(=S)(OCC)OC1=C(C=C(C=C1)Cl)Cl | 176 | 9999 | 2 | 1 |
| Zytron (C10H14Cl2NO2PS) | PJFGPJQBWSEWKX-UHFFFAOYSA-N | CC(C)NP(=S)(OC)OC1=C(C=C(C=C1)Cl)Cl | 0 | 6499 | 2 | 1 |
| Trichloronate (C10H12Cl3O2PS) | ANIAQSUBRGXWLS-UHFFFAOYSA-N | CCOP(=S)(CC)OC1=CC(=C(C=C1Cl)Cl)Cl | 0 | 5384 | 2 | 1 |
| Monomethyl 2,3,5,6-tetrachloroterephthalate (C9H4Cl4O4) | SXINVWXSZUQKSW-UHFFFAOYSA-N | COC(=O)C1=C(C(=C(C(=C1Cl)Cl)C(=O)O)Cl)Cl | 1962 | 45 | 1 | 2 |
| Sarcosine, N-(2-chlorobenzoyl)-, ethyl ester (C12H14ClNO3) | RBBWFAMCKUUJGX-UHFFFAOYSA-N | CCOC(=O)CN(C)C(=O)C1=CC=CC=C1Cl | 1672 | 110 | 1 | 1 |
| Sarcosine, N-(4-chlorobenzoyl)-, ethyl ester (C12H14ClNO3) | CSDYVZTVOGIXFK-UHFFFAOYSA-N | CCOC(=O)CN(C)C(=O)C1=CC=C(C=C1)Cl | 1061 | 0 | 1 | 2 |
| N,N-Bis(2-chloroethyl)-p-toluenesulfonamide (C11H15Cl2NO2S) | PTVBBIMKLOMGSY-UHFFFAOYSA-N | CC1=CC=C(C=C1)S(=O)(=O)N(CCCl)CCCl | 30 | 10 | 2 | 2 |
| Neburon (C12H16Cl2N2O) | CCGPUGMWYLICGL-UHFFFAOYSA-N | CCCCN(C)C(=O)NC1=CC(=C(C=C1)Cl)Cl | 671 | 15 | 2 | 2 |
| 2,4,5-TB methyl ester (C11H11Cl3O3) | OTBMAATXBNOQHO-UHFFFAOYSA-N | COC(=O)CCCOC1=CC(=C(C=C1Cl)Cl)Cl | 40 | 0 | 2 | 2 |
| 2,4,6-Trichlorophenol, isoBOC (C11H11Cl3O3) | UDQGCNQSIVYSON-UHFFFAOYSA-N | CC(C)COC(=O)OC1=C(C=C(C=C1Cl)Cl)Cl | 0 | 0 | 2 | 2 |
| tert-Butyl 2,4,5-trichlorophenyl carbonate (C11H11Cl3O3) | LJFPGBIHDPZHIN-UHFFFAOYSA-N | CC(C)(C)OC(=O)OC1=CC(=C(C=C1Cl)Cl)Cl | 0 | 0 | 2 | 2 |
| Chlorazine (C11H20ClN5) | QHXDTLYEHWXDSO-UHFFFAOYSA-N | CCN(CC)C1=NC(=NC(=N1)Cl)N(CC)CC | 3679 | 229 | 1 | 1 |
| 5-Chlorovaleric acid, 4-nitrophenyl ester (C11H12ClNO4) | XBBBWKBYQVWSPV-UHFFFAOYSA-N | C1=CC(=CC=C1[N+](=O)[O-])OC(=O)CCCCCl | 0 | 0 | 2 | 2 |
| Chloroacetyl-L-tyrosine (C11H12ClNO4) | GDOGSOZOUAVIFX-SECBINFHSA-N | C1=CC(=CC=C1C[C@H](C(=O)O)NC(=O)CCl)O | 20 | 10 | 2 | 2 |
| β-Alanine, N-(2-chlorobenzoyl)-, ethyl ester (C12H14ClNO3) | SLITWDDTDLXMJU-UHFFFAOYSA-N | CCOC(=O)CCNC(=O)C1=CC=CC=C1Cl | 721 | 140 | 2 | 1 |
| β-Alanine, N-(4-chlorobenzoyl)-, ethyl ester (C12H14ClNO3) | OFYIJZQIJRBKNG-UHFFFAOYSA-N | CCOC(=O)CCNC(=O)C1=CC=C(C=C1)Cl | 941 | 0 | 1 | 2 |
| 2-Chloro-4-nitro-N,N-dipropylaniline (C12H17ClN2O2) | OYJJPFCGGNTTTO-UHFFFAOYSA-N | CCCN(CCC)C1=C(C=C(C=C1)[N+](=O)[O-])Cl | 900 | 15 | 1 | 2 |
| Dimethachlor (C13H18ClNO2) | SCCDDNKJYDZXMM-UHFFFAOYSA-N | CC1=C(C(=CC=C1)C)N(CCOC)C(=O)CCl | 135 | 15 | 2 | 2 |
| 2,4-D butyl ester (C12H14Cl2O3) | UQMRAFJOBWOFNS-UHFFFAOYSA-N | CCCCOC(=O)COC1=C(C=C(C=C1)Cl)Cl | 7506 | 0 | 1 | 2 |
| 2,4-D isobutyl ester (C12H14Cl2O3) | GPSGZZSRFJXXBA-UHFFFAOYSA-N | CC(C)COC(=O)COC1=C(C=C(C=C1)Cl)Cl | 680 | 0 | 2 | 2 |
| Urea, N-(2-chloroethyl)-N'-(2,6-dioxo-3-piperidinyl)-N-nitroso- (C8H11ClN4O4) | KHWIRCOLWPNBJP-UHFFFAOYSA-N | C1CC(=O)NC(=O)C1NC(=O)N(CCCl)N=O | 156 | 0 | 2 | 2 |
| 6-Chlorohexanoic acid, 4-methoxyphenyl ester (C13H17ClO3) | WIUNAWMQMZCBAR-UHFFFAOYSA-N | COC1=CC=C(C=C1)OC(=O)CCCCCCl | 260 | 0 | 2 | 2 |
| Butanilicaine (C13H19ClN2O) | VWYQKFLLGRBICZ-UHFFFAOYSA-N | CCCCNCC(=O)NC1=C(C=CC=C1Cl)C | 120 | 930 | 2 | 1 |
| 5'-Chloro-2'-methoxy-2-methylvaleranilide (C13H18ClNO2) | YFWAXWYCHZLBET-UHFFFAOYSA-N | CCCC(C)C(=O)NC1=C(C=CC(=C1)Cl)OC | 1501 | 19 | 1 | 2 |
| 6-Chlorohexanoic acid, 3,4-dichlorophenyl ester (C12H13Cl3O2) | IBFRHXSHKRZPDS-UHFFFAOYSA-N | C1=CC(=C(C=C1OC(=O)CCCCCCl)Cl)Cl | 180 | 0 | 2 | 2 |
| 6-Chlorohexanoic acid, 4-cyanophenyl ester (C13H14ClNO2) | SDRUVBBNRXNYRO-UHFFFAOYSA-N | C1=CC(=CC=C1C#N)OC(=O)CCCCCCl | 0 | 0 | 2 | 2 |
| Octanamide, N-(3-chlorophenyl)- (C14H20ClNO) | FWIHIQJEUPYAPG-UHFFFAOYSA-N | CCCCCCCC(=O)NC1=CC(=CC=C1)Cl | 601 | 0 | 2 | 2 |
| Benzoic acid, 2-chloro, heptyl ester (C14H19ClO2) | AZDUVJGZVLGFLA-UHFFFAOYSA-N | CCCCCCCOC(=O)C1=CC=CC=C1Cl | 140 | 0 | 2 | 2 |
| Benzoic acid, 4-chloro, heptyl ester (C14H19ClO2) | ZGRCQXNQOQBZGF-UHFFFAOYSA-N | CCCCCCCOC(=O)C1=CC=C(C=C1)Cl | 30 | 0 | 2 | 2 |
| Octanoic acid, 4-chlorophenyl ester (C14H19ClO2) | FSEXRXSPQYSFBC-UHFFFAOYSA-N | CCCCCCCC(=O)OC1=CC=C(C=C1)Cl | 1331 | 0 | 1 | 2 |
| 4-butylbenzoic acid, 3-chloroprop-2-enyl ester (C14H17ClO2) | FDTZLBPFIUPWST-ONNFQVAWSA-N | CCCCC1=CC=C(C=C1)C(=O)OC/C=C/Cl | 150 | 1551 | 2 | 1 |
| dimethenamid (C12H18ClNO2S) | JLYFCTQDENRSOL-UHFFFAOYSA-N | CC1=CSC(=C1N(C(C)COC)C(=O)CCl)C | 110 | 20 | 2 | 2 |
| Hydrochlorothiazide (C7H8ClN3O4S2) | JZUFKLXOESDKRF-UHFFFAOYSA-N | C1NC2=CC(=C(C=C2S(=O)(=O)N1)S(=O)(=O)N)Cl | 4070 | 0 | 1 | 2 |
| Chlorothiazide (C7H6ClN3O4S2) | JBMKAUGHUNFTOL-UHFFFAOYSA-N | C1=C2C(=CC(=C1Cl)S(=O)(=O)N)S(=O)(=O)N=CN2 | 9999 | 30 | 1 | 2 |
| Benzenesulfonothioic acid, 4-chloro-, S-phenyl ester (C12H9ClO2S2) | OZBQHMNNLSUKDP-UHFFFAOYSA-N | C1=CC=C(C=C1)SS(=O)(=O)C2=CC=C(C=C2)Cl | 4022 | 0 | 1 | 2 |
| Fenson (C12H9ClO2S) | SPJOZZSIXXJYBT-UHFFFAOYSA-N | C1=CC=C(C=C1)S(=O)(=O)OC2=CC=C(C=C2)Cl | 2215 | 0 | 1 | 2 |
| 4-Chloro-4'-nitrodiphenyl sulfide (C12H8ClNO2S) | CPUBRDUBQIVBGM-UHFFFAOYSA-N | C1=CC(=CC=C1[N+](=O)[O-])SC2=CC=C(C=C2)Cl | 9999 | 59 | 1 | 2 |
| Benzene, 1-chloro-4-(4-nitrophenoxy)- (C12H8ClNO3) | GDEZSMXXDMVYHT-UHFFFAOYSA-N | C1=CC(=CC=C1[N+](=O)[O-])OC2=CC=C(C=C2)Cl | 9999 | 0 | 1 | 2 |
| Diazene, bis(3-chlorophenyl)-, 1-oxide (C12H8Cl2N2O) | SXCDGVDBGGCCLV-UHFFFAOYSA-N | C1=CC(=CC(=C1)Cl)N=[N+](C2=CC(=CC=C2)Cl)[O-] | 860 | 473 | 1 | 1 |
| Bis(4-chlorophenylthio)methane (C13H10Cl2S2) | NTZBITFHYUVXFR-UHFFFAOYSA-N | C1=CC(=CC=C1SCSC2=CC=C(C=C2)Cl)Cl | 1677 | 0 | 1 | 2 |
| Diazene, bis(4-chlorophenyl)-, 1-oxide (C12H8Cl2N2O) | NMAZIJPSESMWSA-UHFFFAOYSA-N | C1=CC(=CC=C1N=[N+](C2=CC=C(C=C2)Cl)[O-])Cl | 8768 | 0 | 1 | 2 |
| bis(4-chlorophenyl) sulphone (C12H8Cl2O2S) | GPAPPPVRLPGFEQ-UHFFFAOYSA-N | C1=CC(=CC=C1S(=O)(=O)C2=CC=C(C=C2)Cl)Cl | 2132 | 10 | 1 | 2 |
| Fenticlor (C12H8Cl2O2S) | ANUSOIHIIPAHJV-UHFFFAOYSA-N | C1=CC(=C(C=C1Cl)SC2=C(C=CC(=C2)Cl)O)O | 4039 | 129 | 1 | 1 |
| Tetrasul (C12H6Cl4S) | QUWSDLYBOVGOCW-UHFFFAOYSA-N | C1=CC(=CC=C1SC2=CC(=C(C=C2Cl)Cl)Cl)Cl | 5815 | 290 | 1 | 1 |
| Nicotinic acid, 3,4-dichlorophenyl ester (C12H7Cl2NO2) | GYEMZNAXCNKADB-UHFFFAOYSA-N | C1=CC(=CN=C1)C(=O)OC2=CC(=C(C=C2)Cl)Cl | 731 | 0 | 2 | 2 |
| Acetamide, N-(1-naphthyl)-2,2,2-trichloro- (C12H8Cl3NO) | FEXNJZJFISBCHC-UHFFFAOYSA-N | C1=CC=C2C(=C1)C=CC=C2NC(=O)C(Cl)(Cl)Cl | 3263 | 330 | 1 | 1 |
| Benzamide, N-(3-chlorophenyl)-2-chloro- (C13H9Cl2NO) | HWDAIZBRWARIMB-UHFFFAOYSA-N | C1=CC=C(C(=C1)C(=O)NC2=CC(=CC=C2)Cl)Cl | 1962 | 120 | 1 | 1 |
| Benzamide, N-(3-chlorophenyl)-4-chloro- (C13H9Cl2NO) | ACBJKFUXTGAULL-UHFFFAOYSA-N | C1=CC(=CC(=C1)Cl)NC(=O)C2=CC=C(C=C2)Cl | 2152 | 0 | 1 | 2 |
| 4-Chlorobenzoic acid, 4-chlorophenyl ester (C13H8Cl2O2) | BJLVHBDHEMFVIE-UHFFFAOYSA-N | C1=CC(=CC=C1C(=O)OC2=CC=C(C=C2)Cl)Cl | 440 | 0 | 2 | 2 |
| 2-Chlorobenzoic acid, 4-chlorophenyl ester (C13H8Cl2O2) | FAWUTQLFEATJSE-UHFFFAOYSA-N | C1=CC=C(C(=C1)C(=O)OC2=CC=C(C=C2)Cl)Cl | 250 | 0 | 2 | 2 |
| m-(3,4-Dichlorophenoxy)benzaldehyde (C13H8Cl2O2) | ABQHJSHFFLAGHF-UHFFFAOYSA-N | C1=CC(=CC(=C1)OC2=CC(=C(C=C2)Cl)Cl)C=O | 9999 | 1581 | 1 | 1 |
| PCB 116 (C12H5Cl5) | GGMPTLAAIUQMIE-UHFFFAOYSA-N | C1=CC=C(C=C1)C2=C(C(=C(C(=C2Cl)Cl)Cl)Cl)Cl | 6415 | 178 | 1 | 1 |
| PCB 118 (C12H5Cl5) | IUTPYMGCWINGEY-UHFFFAOYSA-N | C1=CC(=C(C=C1C2=CC(=C(C=C2Cl)Cl)Cl)Cl)Cl | 6012 | 32 | 1 | 2 |
| PCB 105 (C12H5Cl5) | WIDHRBRBACOVOY-UHFFFAOYSA-N | C1=CC(=C(C=C1C2=C(C(=C(C=C2)Cl)Cl)Cl)Cl)Cl | 6057 | 62 | 1 | 2 |
| PCB 101 (C12H5Cl5) | LAHWLEDBADHJGA-UHFFFAOYSA-N | C1=CC(=C(C=C1Cl)C2=CC(=C(C=C2Cl)Cl)Cl)Cl | 6347 | 1362 | 1 | 1 |
| PCB 95 (C12H5Cl5) | GXNNLIMMEXHBKV-UHFFFAOYSA-N | C1=CC(=C(C=C1Cl)C2=C(C=CC(=C2Cl)Cl)Cl)Cl | 5754 | 2284 | 1 | 1 |
| PCB 99 (C12H5Cl5) | LMQJBFRGXHMNOX-UHFFFAOYSA-N | C1=CC(=C(C=C1Cl)Cl)C2=CC(=C(C=C2Cl)Cl)Cl | 6123 | 750 | 1 | 1 |
| PCB 87 (C12H5Cl5) | OPKYDBFRKPQCBS-UHFFFAOYSA-N | C1=CC(=C(C=C1Cl)C2=C(C(=C(C=C2)Cl)Cl)Cl)Cl | 6431 | 2621 | 1 | 1 |
| PCB 110 (C12H5Cl5) | ARXHIJMGSIYYRZ-UHFFFAOYSA-N | C1=CC(=C(C=C1C2=C(C=CC(=C2Cl)Cl)Cl)Cl)Cl | 5928 | 91 | 1 | 2 |
| PCB 100 (C12H5Cl5) | RKUAZJIXKHPFRK-UHFFFAOYSA-N | C1=CC(=C(C=C1Cl)Cl)C2=C(C=C(C=C2Cl)Cl)Cl | 6122 | 1707 | 1 | 1 |
| PCB 97 (C12H5Cl5) | JTUSORDQZVOEAZ-UHFFFAOYSA-N | C1=CC(=C(C(=C1)Cl)Cl)C2=CC(=C(C=C2Cl)Cl)Cl | 6289 | 1825 | 1 | 1 |
| PCB 84 (C12H5Cl5) | QVWUJLANSDKRAH-UHFFFAOYSA-N | C1=CC(=C(C(=C1)Cl)Cl)C2=C(C=CC(=C2Cl)Cl)Cl | 5723 | 2889 | 1 | 1 |
| PCB 92 (C12H5Cl5) | CRCBRZBVCDKPGA-UHFFFAOYSA-N | C1=CC(=C(C=C1Cl)C2=CC(=CC(=C2Cl)Cl)Cl)Cl | 5848 | 1470 | 1 | 1 |
| PCB 82 (C12H5Cl5) | AUGNBQPSMWGAJE-UHFFFAOYSA-N | C1=CC(=C(C(=C1)Cl)Cl)C2=C(C(=C(C=C2)Cl)Cl)Cl | 6190 | 1582 | 1 | 1 |
| PCB 88 (C12H5Cl5) | QGDKRLQRLFUJPP-UHFFFAOYSA-N | C1=CC=C(C(=C1)C2=C(C(=C(C=C2Cl)Cl)Cl)Cl)Cl | 6025 | 2277 | 1 | 1 |
| PCB 86 (C12H5Cl5) | AIURIRUDHVDRFQ-UHFFFAOYSA-N | C1=CC=C(C(=C1)C2=CC(=C(C(=C2Cl)Cl)Cl)Cl)Cl | 6241 | 2241 | 1 | 1 |
| PCB 104 (C12H5Cl5) | MTCPZNVSDFCBBE-UHFFFAOYSA-N | C1=CC(=C(C(=C1)Cl)C2=C(C=C(C=C2Cl)Cl)Cl)Cl | 5867 | 343 | 1 | 1 |
| PCB 119 (C12H5Cl5) | OAEQTHQGPZKTQP-UHFFFAOYSA-N | C1=CC(=C(C=C1C2=C(C=C(C=C2Cl)Cl)Cl)Cl)Cl | 6189 | 267 | 1 | 1 |
| PCB 121 (C12H5Cl5) | XBVSGJGMWSKAKL-UHFFFAOYSA-N | C1=C(C=C(C=C1Cl)Cl)C2=C(C=C(C=C2Cl)Cl)Cl | 6328 | 179 | 1 | 1 |
| PCB 126 (C12H5Cl5) | REHONNLQRWTIFF-UHFFFAOYSA-N | C1=CC(=C(C=C1C2=CC(=C(C(=C2)Cl)Cl)Cl)Cl)Cl | 6077 | 27 | 1 | 2 |
| PCB 83 (C12H5Cl5) | SUBRHHYLRGOTHL-UHFFFAOYSA-N | C1=CC(=C(C(=C1)Cl)Cl)C2=CC(=CC(=C2Cl)Cl)Cl | 6341 | 3178 | 1 | 1 |
| 1,1'-Biphenyl, 2,2',4,5',6-Pentachloro- (C12H5Cl5) | PQHZWWBJPCNNGI-UHFFFAOYSA-N | C1=CC(=C(C=C1Cl)C2=C(C=C(C=C2Cl)Cl)Cl)Cl | 6387 | 2004 | 1 | 1 |
| PCB 98 (C12H5Cl5) | GOFFZTAPOOICFT-UHFFFAOYSA-N | C1=CC(=C(C(=C1)Cl)Cl)C2=C(C=C(C=C2Cl)Cl)Cl | 6026 | 2753 | 1 | 1 |
| PCB 123 (C12H5Cl5) | YAHNWSSFXMVPOU-UHFFFAOYSA-N | C1=CC(=C(C=C1Cl)Cl)C2=CC(=C(C(=C2)Cl)Cl)Cl | 6138 | 0 | 1 | 2 |
| PCB 85 (C12H5Cl5) | LACXVZHAJMVESG-UHFFFAOYSA-N | C1=CC(=C(C=C1Cl)Cl)C2=C(C(=C(C=C2)Cl)Cl)Cl | 6256 | 1518 | 1 | 1 |
| PCB 91 (C12H5Cl5) | CXKIGWXPPVZSQK-UHFFFAOYSA-N | C1=CC(=C(C=C1Cl)Cl)C2=C(C=CC(=C2Cl)Cl)Cl | 5912 | 1867 | 1 | 1 |
| PCB 102 (C12H5Cl5) | BWWVXHRLMPBDCK-UHFFFAOYSA-N | C1=CC(=C(C(=C1)Cl)C2=CC(=C(C=C2Cl)Cl)Cl)Cl | 6190 | 1582 | 1 | 1 |
| PCB 113 (C12H5Cl5) | YDGFMDPEJCJZEV-UHFFFAOYSA-N | C1=CC(=C(C(=C1Cl)C2=CC(=CC(=C2)Cl)Cl)Cl)Cl | 5614 | 166 | 1 | 1 |
| PCB 117 (C12H5Cl5) | ZDDZPDTVCZLFFC-UHFFFAOYSA-N | C1=CC(=CC=C1C2=C(C(=CC(=C2Cl)Cl)Cl)Cl)Cl | 6412 | 97 | 1 | 2 |
| PCB 120 (C12H5Cl5) | ZLGYJAIAVPVCNF-UHFFFAOYSA-N | C1=C(C=C(C=C1Cl)Cl)C2=CC(=C(C=C2Cl)Cl)Cl | 6393 | 0 | 1 | 2 |
| PCB 124 (C12H5Cl5) | PIVBPZFQXKMHBD-UHFFFAOYSA-N | C1=CC(=C(C=C1Cl)C2=CC(=C(C(=C2)Cl)Cl)Cl)Cl | 6093 | 79 | 1 | 2 |
| PCB 93 (C12H5Cl5) | BMXRLHMJGHJGLR-UHFFFAOYSA-N | C1=CC=C(C(=C1)C2=C(C(=CC(=C2Cl)Cl)Cl)Cl)Cl | 6174 | 2673 | 1 | 1 |
| PCB 109 (C12H5Cl5) | XGQBSVVYMVILEL-UHFFFAOYSA-N | C1=CC(=CC(=C1)Cl)C2=C(C(=C(C=C2Cl)Cl)Cl)Cl | 6404 | 188 | 1 | 1 |
| PCB 114 (C12H5Cl5) | SXZSFWHOSHAKMN-UHFFFAOYSA-N | C1=CC(=CC=C1C2=CC(=C(C(=C2Cl)Cl)Cl)Cl)Cl | 6285 | 34 | 1 | 2 |
| PCB 115 (C12H5Cl5) | IOVARPVVZDOPGQ-UHFFFAOYSA-N | C1=CC(=CC=C1C2=C(C(=C(C=C2Cl)Cl)Cl)Cl)Cl | 6370 | 52 | 1 | 2 |
| Isonicotinic acid, (4-chlorophenyl)methyl ester (C13H10ClNO2) | KFNLUWVOHPCBPL-UHFFFAOYSA-N | C1=CC(=CC=C1COC(=O)C2=CC=NC=C2)Cl | 3253 | 190 | 1 | 1 |
| Nicotinic acid, (4-chlorophenyl)methyl ester (C13H10ClNO2) | FLUZHFKASOGKOP-UHFFFAOYSA-N | C1=CC(=CN=C1)C(=O)OCC2=CC=C(C=C2)Cl | 7847 | 190 | 1 | 1 |
| Benzonitrile, 2-chloro-6-(phenylmethoxy)- (C14H10ClNO) | CEFUICQUABSEPM-UHFFFAOYSA-N | C1=CC=C(C=C1)COC2=C(C(=CC=C2)Cl)C#N | 424 | 0 | 2 | 2 |
| Acetamide, N-(3-chlorophenyl)-2-phenyl- (C14H12ClNO) | GAWITMMQHFGOMV-UHFFFAOYSA-N | C1=CC=C(C=C1)CC(=O)NC2=CC(=CC=C2)Cl | 5895 | 0 | 1 | 2 |
| 2,5-Diamino-2'-chlorobenzophenone (C13H11ClN2O) | HMTGFGCXWOUKTO-UHFFFAOYSA-N | C1=CC=C(C(=C1)C(=O)C2=C(C=CC(=C2)N)N)Cl | 9999 | 4200 | 1 | 1 |
| 4-Chlorobenzyl benzoate (C14H11ClO2) | ARLTXMAKDGVKNK-UHFFFAOYSA-N | C1=CC=C(C=C1)C(=O)OCC2=CC=C(C=C2)Cl | 1832 | 140 | 1 | 1 |
| Benzyl 4-chlorobenzoate (C14H11ClO2) | VBIOKGVOQMIBHO-UHFFFAOYSA-N | C1=CC=C(C=C1)COC(=O)C2=CC=C(C=C2)Cl | 4854 | 911 | 1 | 1 |
| Benzyl 2-chlorobenzoate (C14H11ClO2) | IJIVXOPMPSQRSF-UHFFFAOYSA-N | C1=CC=C(C=C1)COC(=O)C2=CC=CC=C2Cl | 1541 | 420 | 1 | 1 |
| Phenylacetic acid, 2-chlorophenyl ester (C14H11ClO2) | KQBWWQRVHFNTSK-UHFFFAOYSA-N | C1=CC=C(C=C1)CC(=O)OC2=CC=CC=C2Cl | 0 | 0 | 2 | 2 |
| Phenylacetic acid, 4-chlorophenyl ester (C14H11ClO2) | WQHZGEHSVIOHHW-UHFFFAOYSA-N | C1=CC=C(C=C1)CC(=O)OC2=CC=C(C=C2)Cl | 50 | 0 | 2 | 2 |
| Benzamide, N-(3-chlorophenyl)-4-methyl- (C14H12ClNO) | RUYDNAMXAAGBCA-UHFFFAOYSA-N | CC1=CC=C(C=C1)C(=O)NC2=CC(=CC=C2)Cl | 1692 | 0 | 1 | 2 |
| Benzamide, N-(3-methylphenyl)-2-chloro- (C14H12ClNO) | QMUDBMHUUBUXHG-UHFFFAOYSA-N | CC1=CC(=CC=C1)NC(=O)C2=CC=CC=C2Cl | 3693 | 450 | 1 | 1 |
| Benzamide, N-(3-chlorophenyl)-2-methyl- (C14H12ClNO) | ZNBNLMVCJLGUKS-UHFFFAOYSA-N | CC1=CC=CC=C1C(=O)NC2=CC(=CC=C2)Cl | 1441 | 0 | 1 | 2 |
| Benzamide, N-(3-chlorophenyl)-3-methyl- (C14H12ClNO) | PXBPMCNEAZKAKS-UHFFFAOYSA-N | CC1=CC=CC(=C1)C(=O)NC2=CC(=CC=C2)Cl | 2252 | 0 | 1 | 2 |
| Benzamide, N-(3-methylphenyl)-4-chloro- (C14H12ClNO) | ZGNNVNCFQUJRAG-UHFFFAOYSA-N | CC1=CC(=CC=C1)NC(=O)C2=CC=C(C=C2)Cl | 3543 | 0 | 1 | 2 |
| 2-Methylamino-5-chlorobenzophenone (C14H12ClNO) | WPNMLCMTDCANOZ-UHFFFAOYSA-N | CNC1=C(C=C(C=C1)Cl)C(=O)C2=CC=CC=C2 | 9999 | 324 | 1 | 1 |
| p-Toluylic acid, 2-chlorophenyl ester (C14H11ClO2) | SHDNIUHQTKMTGG-UHFFFAOYSA-N | CC1=CC=C(C=C1)C(=O)OC2=CC=CC=C2Cl | 110 | 0 | 2 | 2 |
| o-Toluylic acid, 4-chlorophenyl ester (C14H11ClO2) | GINBDDAROBWNDR-UHFFFAOYSA-N | CC1=CC=CC=C1C(=O)OC2=CC=C(C=C2)Cl | 70 | 0 | 2 | 2 |
| m-Toluylic acid, 4-chlorophenyl ester (C14H11ClO2) | AFMWNHRHTYUYLU-UHFFFAOYSA-N | CC1=CC=CC(=C1)C(=O)OC2=CC=C(C=C2)Cl | 280 | 0 | 2 | 2 |
| 4-Chlorobenzoic acid, 3-methylphenyl ester (C14H11ClO2) | GAUJZVXSFTUKEY-UHFFFAOYSA-N | CC1=CC(=CC=C1)OC(=O)C2=CC=C(C=C2)Cl | 1191 | 0 | 1 | 2 |
| p-Toluylic acid, 4-chlorophenyl ester (C14H11ClO2) | WFAGYTKJEMEMDZ-UHFFFAOYSA-N | CC1=CC=C(C=C1)C(=O)OC2=CC=C(C=C2)Cl | 220 | 0 | 2 | 2 |
| 2-Chlorobenzoic acid, 3-methylphenyl ester (C14H11ClO2) | XTOVEDGKBJLUIA-UHFFFAOYSA-N | CC1=CC(=CC=C1)OC(=O)C2=CC=CC=C2Cl | 791 | 0 | 2 | 2 |
| 4,4'-Methylene-bis-(2-chloroaniline) (C13H12Cl2N2) | IBOFVQJTBBUKMU-UHFFFAOYSA-N | C1=CC(=C(C=C1CC2=CC(=C(C=C2)N)Cl)Cl)N | 6549 | 9999 | 1 | 1 |
| Dichlorophen (C13H10Cl2O2) | MDNWOSOZYLHTCG-UHFFFAOYSA-N | C1=CC(=C(C=C1Cl)CC2=C(C=CC(=C2)Cl)O)O | 3260 | 1150 | 1 | 1 |
| Benzophenone, 2-amino-2',5-dichloro- (C13H9Cl2NO) | KWZYIAJRFJVQDO-UHFFFAOYSA-N | C1=CC=C(C(=C1)C(=O)C2=C(C=CC(=C2)Cl)N)Cl | 5639 | 9999 | 1 | 1 |
| Chlormezanone (C11H12ClNO3S) | WEQAYVWKMWHEJO-UHFFFAOYSA-N | CN1C(S(=O)(=O)CCC1=O)C2=CC=C(C=C2)Cl | 0 | 0 | 2 | 2 |
| Pyrimethamine (C12H13ClN4) | WKSAUQYGYAYLPV-UHFFFAOYSA-N | CCC1=C(C(=NC(=N1)N)N)C2=CC=C(C=C2)Cl | 4860 | 350 | 1 | 1 |
| 4-Chlorobutyric acid, 2-naphthyl ester (C14H13ClO2) | KNSGPNGISXLZHO-UHFFFAOYSA-N | C1=CC=C2C=C(C=CC2=C1)OC(=O)CCCCl | 601 | 0 | 2 | 2 |
| Benzhydryl 2-chloroethyl ether (C15H15ClO) | ZNVASENTCOLNJT-UHFFFAOYSA-N | C1=CC=C(C=C1)C(C2=CC=CC=C2)OCCCl | 3275 | 0 | 1 | 2 |
| Benzophenone, 2-methylamino-5-chloro- (C14H12ClNO) | YHSCBLSYYDIOFJ-UHFFFAOYSA-N | C1=CC=C(C=C1)C(=O)C2=C(C=CC(=C2)Cl)CN | 9999 | 301 | 1 | 1 |
| 4-Chlorodibenzoyl (C14H9ClO2) | QDCKVAZDINMMHO-UHFFFAOYSA-N | C1=CC=C(C=C1)C(=O)C(=O)C2=CC=C(C=C2)Cl | 79 | 16 | 2 | 2 |
| Ethanol, 2,2-bis-(4-chlorophenyl) (C14H12Cl2O) | ZVIDYKRNLNAXFT-UHFFFAOYSA-N | C1=CC(=CC=C1C(CO)C2=CC=C(C=C2)Cl)Cl | 770 | 240 | 2 | 1 |
| Chlorfenethol (C14H12Cl2O) | URYAFVKLYSEINW-UHFFFAOYSA-N | CC(C1=CC=C(C=C1)Cl)(C2=CC=C(C=C2)Cl)O | 970 | 120 | 1 | 1 |
| 1-Chloro-2,2-Bis(p-chlorophenyl)ethane (C14H11Cl3) | CHBOSHOWERDCMH-UHFFFAOYSA-N | C1=CC(=CC=C1C(CCl)C2=CC=C(C=C2)Cl)Cl | 600 | 0 | 2 | 2 |
| Procyazine (C10H13ClN6) | WUZNHSBFPPFULJ-UHFFFAOYSA-N | CC(C)(C#N)NC1=NC(=NC(=N1)NC2CC2)Cl | 5000 | 310 | 1 | 1 |
| 3',4'-Dichlorocyclohexanecarboxanilide (C13H15Cl2NO) | QNLPPYRKEBGGHV-UHFFFAOYSA-N | C1CCC(CC1)C(=O)NC2=CC(=C(C=C2)Cl)Cl | 800 | 17 | 2 | 2 |
| Cyclohexanecarboxylic acid, 3,4-dichlorophenyl ester (C13H14Cl2O2) | JMJTVAZCYQSBSD-UHFFFAOYSA-N | C1CCC(CC1)C(=O)OC2=CC(=C(C=C2)Cl)Cl | 604 | 0 | 2 | 2 |
| 3-Cyclopentylpropionic acid, 4-chlorophenyl ester (C14H17ClO2) | XVFXNEMYRISWTL-UHFFFAOYSA-N | C1CCC(C1)CCC(=O)OC2=CC=C(C=C2)Cl | 1762 | 0 | 1 | 2 |
| 1-Naphthoic acid, 3-chloroprop-2-enyl ester (C14H11ClO2) | OAUXYJNCYQYCJW-RUDMXATFSA-N | C1=CC=C2C(=C1)C=CC=C2C(=O)OC/C=C/Cl | 2923 | 370 | 1 | 1 |
| p,p'-DME (C14H9Cl3) | LNKQQZFLNUVWQQ-UHFFFAOYSA-N | C1=CC(=CC=C1C(=CCl)C2=CC=C(C=C2)Cl)Cl | 9677 | 1911 | 1 | 1 |
| Benzene, 1-chloro-4-(2-cyano-2-phenylethenyl) (C15H10ClN) | WHZUHCZQGFNGNH-GXDHUFHOSA-N | C1=CC=C(C=C1)/C(=C/C2=CC=C(C=C2)Cl)/C#N | 5451 | 9999 | 1 | 1 |
| α-(p-Chlorophenyl)cinnamonitrile (C15H10ClN) | JJGLLZWUWIKTAG-GXDHUFHOSA-N | C1=CC=C(C=C1)/C=C(\C#N)/C2=CC=C(C=C2)Cl | 4864 | 9999 | 1 | 1 |
| 3-(2,6-Dichlorophenyl)-5-methyl isoxazole-4-carbonyl chloride (C11H6Cl3NO2) | IZQGELJKDARDMZ-UHFFFAOYSA-N | CC1=C(C(=NO1)C2=C(C=CC=C2Cl)Cl)C(=O)Cl | 90 | 1862 | 2 | 1 |
| 1-Cyclohexenyl-5-chloro-2-methylaminophenyl ketone (C14H16ClNO) | CZSVMGRCLAQSME-UHFFFAOYSA-N | CNC1=C(C=C(C=C1)Cl)C(=O)C2=CCCCC2 | 5535 | 0 | 1 | 2 |
| Chlorbicyclen (C9H6Cl8) | FUZORIOHZSVKAW-UHFFFAOYSA-N | C(C1C(C2(C(=C(C1(C2(Cl)Cl)Cl)Cl)Cl)Cl)CCl)Cl | 220 | 1580 | 2 | 1 |
| 2-Thiopheneacetic acid, 3,4-dichlorophenyl ester (C12H8Cl2O2S) | BTHYDFCWZORZBJ-UHFFFAOYSA-N | C1=CSC(=C1)CC(=O)OC2=CC(=C(C=C2)Cl)Cl | 460 | 0 | 2 | 2 |
| Phenothiazine-10-carbonyl chloride (C13H8ClNOS) | MJRIZSDRKPPHTK-UHFFFAOYSA-N | C1=CC=C2C(=C1)N(C3=CC=CC=C3S2)C(=O)Cl | 2184 | 25 | 1 | 2 |
| Dibenzo[b,e][1,4]dioxin, 1,2,4-trichloro- (C12H5Cl3O2) | HRVUKLBFRPWXPJ-UHFFFAOYSA-N | C1=CC=C2C(=C1)OC3=C(O2)C(=C(C=C3Cl)Cl)Cl | 9999 | 316 | 1 | 1 |
| 2H-1,3-Benzoxazine, 6-chloro-3,4-dihydro-3-phenyl- (C14H12ClNO) | ANDMOXODDHVMFN-UHFFFAOYSA-N | C1C2=C(C=CC(=C2)Cl)OCN1C3=CC=CC=C3 | 136 | 0 | 2 | 2 |
| 2,3,7,8-Tetrachlorodibenzofuran (C12H4Cl4O) | KSMVNVHUTQZITP-UHFFFAOYSA-N | C1=C2C3=CC(=C(C=C3OC2=CC(=C1Cl)Cl)Cl)Cl | 7619 | 440 | 1 | 1 |
| Am-ex-ol (C14H9ClN2) | OBHKONRNYCDRKM-UHFFFAOYSA-N | C1=CC=C(C=C1)C2=NC3=CC=CC=C3C(=N2)Cl | 3673 | 9999 | 1 | 1 |
| 2-Chloroanthraquinone (C14H7ClO2) | FPKCTSIVDAWGFA-UHFFFAOYSA-N | C1=CC=C2C(=C1)C(=O)C3=C(C2=O)C=C(C=C3)Cl | 9999 | 2590 | 1 | 1 |
| Ticlopidine (C14H14ClNS) | PHWBOXQYWZNQIN-UHFFFAOYSA-N | C1CN(CC2=C1SC=C2)CC3=CC=CC=C3Cl | 4233 | 440 | 1 | 1 |
| 1-Hydroxychlordene (C10H6Cl6O) | YQWCIPIEEBVRNY-UHFFFAOYSA-N | C1=CC(C2C1C3(C(=C(C2(C3(Cl)Cl)Cl)Cl)Cl)Cl)O | 0 | 990 | 2 | 1 |
| Heptachlor (C10H5Cl7) | FRCCEHPWNOQAEU-UHFFFAOYSA-N | C1=CC(C2C1C3(C(=C(C2(C3(Cl)Cl)Cl)Cl)Cl)Cl)Cl | 342 | 898 | 2 | 1 |
| Chlordene epoxide (C10H6Cl6O) | VMNNMBZKONGDDQ-UHFFFAOYSA-N | C1C2C(C3C1O3)C4(C(=C(C2(C4(Cl)Cl)Cl)Cl)Cl)Cl | 571 | 3583 | 2 | 1 |
| 1-Chloropyrene (C16H9Cl) | WNYHOOQHJMHHQW-UHFFFAOYSA-N | C1=CC2=C3C(=C1)C=CC4=C(C=CC(=C43)C=C2)Cl | 9999 | 3143 | 1 | 1 |
| Succinic acid, di(2,2,2-trichloroethyl) ester (C8H8Cl6O4) | KUKFKXHVFOUECA-UHFFFAOYSA-N | C(CC(=O)OCC(Cl)(Cl)Cl)C(=O)OCC(Cl)(Cl)Cl | 0 | 0 | 2 | 2 |
| Succinic acid, pentyl 2,2,2-trichloroethyl ester (C11H17Cl3O4) | SOSGHDULTDVXJY-UHFFFAOYSA-N | CCCCCOC(=O)CCC(=O)OCC(Cl)(Cl)Cl | 0 | 0 | 2 | 2 |
| Malonic acid, 2,2-dichloroethyl heptyl ester (C12H20Cl2O4) | UPSFVOVWXAXHHD-UHFFFAOYSA-N | CCCCCCCOC(=O)CC(=O)OCC(Cl)Cl | 0 | 70 | 2 | 2 |
| Malonic acid, 8-chlorooctyl ethyl ester (C13H23ClO4) | QVPHPCBYJIWUMA-UHFFFAOYSA-N | CCOC(=O)CC(=O)OCCCCCCCCCl | 0 | 0 | 2 | 2 |
| D-Alanine, N-(5-chlorovaleryl)-, pentyl ester (C13H24ClNO3) | FSKCEGKRZVKHRS-UHFFFAOYSA-N | CCCCCOC(=O)C(C)NC(=O)CCCCCl | 0 | 370 | 2 | 1 |
| Malonic acid, 2-chloropropyl heptyl ester (C13H23ClO4) | IEQDMFOGLIMBKT-UHFFFAOYSA-N | CCCCCCCOC(=O)CC(=O)OCC(C)Cl | 0 | 20 | 2 | 2 |
| Succinic acid, 2-chloropropyl hexyl ester (C13H23ClO4) | VSDFQIDHPWYVAS-UHFFFAOYSA-N | CCCCCCOC(=O)CCC(=O)OCC(C)Cl | 0 | 0 | 2 | 2 |
| Succinic acid, 2-chloropropyl isohexyl ester (C13H23ClO4) | WCTQPSDQUVPTMD-UHFFFAOYSA-N | CC(C)CCCOC(=O)CCC(=O)OCC(C)Cl | 0 | 0 | 2 | 2 |
| Trichloroacetamide, N-decyl-N-methyl- (C13H24Cl3NO) | SJOGSVVCAXLHSK-UHFFFAOYSA-N | CCCCCCCCCCN(C)C(=O)C(Cl)(Cl)Cl | 0 | 1391 | 2 | 1 |
| Dichloroacetamide, N,N-dihexyl- (C14H27Cl2NO) | TXPVLEZUMVIIBX-UHFFFAOYSA-N | CCCCCCN(CCCCCC)C(=O)C(Cl)Cl | 0 | 2973 | 2 | 1 |
| Trichloroacetic acid, undecyl ester (C13H23Cl3O2) | ZKEYPXQCWNXAQG-UHFFFAOYSA-N | CCCCCCCCCCCOC(=O)C(Cl)(Cl)Cl | 0 | 0 | 2 | 2 |
| Propanamide, N,N-dihexyl-3-chloro- (C15H30ClNO) | SIHRUMLFKNAMRI-UHFFFAOYSA-N | CCCCCCN(CCCCCC)C(=O)CCCl | 70 | 3853 | 2 | 1 |
| Propanamide, N,N-dihexyl-2-chloro- (C15H30ClNO) | CPIHTZXGVWMATK-UHFFFAOYSA-N | CCCCCCN(CCCCCC)C(=O)C(C)Cl | 150 | 5575 | 2 | 1 |
| Dodecyl dichloroacetate (C14H26Cl2O2) | XOWWGPRKFWEEDR-UHFFFAOYSA-N | CCCCCCCCCCCCOC(=O)C(Cl)Cl | 0 | 0 | 2 | 2 |
| 5-Chlorovaleric acid, decyl ester (C15H29ClO2) | GDDOSYXFHTVPTR-UHFFFAOYSA-N | CCCCCCCCCCOC(=O)CCCCCl | 0 | 270 | 2 | 1 |
| Propanoic acid, 3-chloro, dodecyl ester (C15H29ClO2) | NCUROSDPAOGNHW-UHFFFAOYSA-N | CCCCCCCCCCCCOC(=O)CCCl | 0 | 240 | 2 | 1 |
| Palmitoyl chloride (C16H31ClO) | ARBOVOVUTSQWSS-UHFFFAOYSA-N | CCCCCCCCCCCCCCCC(=O)Cl | 0 | 1802 | 2 | 1 |
| Phosphamidon (C10H19ClNO5P) | RGCLLPNLLBQHPF-HJWRWDBZSA-N | CCN(CC)C(=O)/C(=C(\C)/OP(=O)(OC)OC)/Cl | 0 | 4629 | 2 | 1 |
| Phosphamidon, Z (C10H19ClNO5P) | RGCLLPNLLBQHPF-HJWRWDBZSA-N | CCN(CC)C(=O)/C(=C(\C)/OP(=O)(OC)OC)/Cl | 0 | 4629 | 2 | 1 |
| (E)-Phosphamidon (C10H19ClNO5P) | RGCLLPNLLBQHPF-CMDGGOBGSA-N | CCN(CC)C(=O)/C(=C(/C)\OP(=O)(OC)OC)/Cl | 0 | 3223 | 2 | 1 |
| Fumaric acid, di(2,2,2-trichloroethyl) ester (C8H6Cl6O4) | WFTACGQPAJJUOO-OWOJBTEDSA-N | C(C(Cl)(Cl)Cl)OC(=O)/C=C/C(=O)OCC(Cl)(Cl)Cl | 0 | 470 | 2 | 1 |
| Fumaric acid, pentyl 2,2,2-trichloroethyl ester (C11H15Cl3O4) | CPZRBRIYEOEULK-AATRIKPKSA-N | CCCCCOC(=O)/C=C/C(=O)OCC(Cl)(Cl)Cl | 0 | 1161 | 2 | 1 |
| Fumaric acid, 2-chloropropyl hexyl ester (C13H21ClO4) | IXVHEHRRSGIADG-BQYQJAHWSA-N | CCCCCCOC(=O)/C=C/C(=O)OCC(C)Cl | 0 | 30 | 2 | 2 |
| Trichloroacetic acid, 2,6-dimethylnon-1-en-3-yn-5-yl ester (C13H17Cl3O2) | QUQXVSNIXBWVAX-UHFFFAOYSA-N | CCCC(C)C(C#CC(=C)C)OC(=O)C(Cl)(Cl)Cl | 0 | 400 | 2 | 1 |
| Trichloroacetic acid, undec-2-enyl ester (C13H21Cl3O2) | HPCFGWRDJUTDBB-MDZDMXLPSA-N | CCCCCCCC/C=C/COC(=O)C(Cl)(Cl)Cl | 0 | 40 | 2 | 2 |
| 5-chlorovaleric acid, 2,7-dimethylocty-7-en-5-yn-4-yl ester (C15H23ClO2) | MABVZJMLUHQMDS-UHFFFAOYSA-N | CC(C)CC(C#CC(=C)C)OC(=O)CCCCCl | 430 | 30 | 2 | 2 |
| Phenol, pentachloro-, trichloroacetate (C8Cl8O2) | WMUFBMFZOHGUPA-UHFFFAOYSA-N | C1(=C(C(=C(C(=C1Cl)Cl)Cl)Cl)Cl)OC(=O)C(Cl)(Cl)Cl | 60 | 20 | 2 | 2 |
| Chlorpyriphos (C9H11Cl3NO3PS) | SBPBAQFWLVIOKP-UHFFFAOYSA-N | CCOP(=S)(OCC)OC1=NC(=C(C=C1Cl)Cl)Cl | 651 | 4744 | 2 | 1 |
| Chlorpyrifos (C9H11Cl3NO4P) | OTMOUPHCTWPNSL-UHFFFAOYSA-N | CCOP(=O)(OCC)OC1=NC(=C(C=C1Cl)Cl)Cl | 929 | 5242 | 1 | 1 |
| Carbophenothion O-analog (C11H16ClO3PS2) | ZSAZGCBSZUURAX-UHFFFAOYSA-N | CCOP(=O)(OCC)SCSC1=CC=C(C=C1)Cl | 490 | 0 | 2 | 2 |
| Carbophenothion (C11H16ClO2PS3) | VEDTXTNSFWUXGQ-UHFFFAOYSA-N | CCOP(=S)(OCC)SCSC1=CC=C(C=C1)Cl | 7523 | 0 | 1 | 2 |
| Chlorproguanil (C11H15Cl2N5) | ISZNZKHCRKXXAU-UHFFFAOYSA-N | CC(C)N=C(N)/N=C(\N)/NC1=CC(=C(C=C1)Cl)Cl | 2808 | 0 | 1 | 2 |
| chlorthal-dimethyl (C10H6Cl4O4) | NPOJQCVWMSKXDN-UHFFFAOYSA-N | COC(=O)C1=C(C(=C(C(=C1Cl)Cl)C(=O)OC)Cl)Cl | 2506 | 108 | 1 | 1 |
| Phosphorodithioic acid, O-(2,4-dichlorophenyl) O-ethyl S-propyl ester (C11H15Cl2O2PS2) | FITIWKDOCAUBQD-UHFFFAOYSA-N | CCCSP(=S)(OCC)OC1=C(C=C(C=C1)Cl)Cl | 65 | 4234 | 2 | 1 |
| Phosphorothioic acid, O-[5-chloro-1-(1-methylethyl)-1H-1,2,4-triazol-3-yl] O,O-diethyl ester (C9H17ClN3O3PS) | XRHGWAGWAHHFLF-UHFFFAOYSA-N | CCOP(=S)(OCC)OC1=NN(C(=N1)Cl)C(C)C | 860 | 620 | 1 | 1 |
| Pentanamide, N-(2,5-dimethoxyphenyl)-5-chloro- (C13H18ClNO3) | RUIMICHAILIILK-UHFFFAOYSA-N | COC1=CC(=C(C=C1)OC)NC(=O)CCCCCl | 4614 | 130 | 1 | 1 |
| Chloroprocaine (C13H19ClN2O2) | VDANGULDQQJODZ-UHFFFAOYSA-N | CCN(CC)CCOC(=O)C1=C(C=C(C=C1)N)Cl | 0 | 0 | 2 | 2 |
| Sarcosine, N-(2-chlorobenzoyl)-, propyl ester (C13H16ClNO3) | MIFLCFDAVSUCKU-UHFFFAOYSA-N | CCCOC(=O)CN(C)C(=O)C1=CC=CC=C1Cl | 1531 | 100 | 1 | 2 |
| Sarcosine, N-(4-chlorobenzoyl)-, propyl ester (C13H16ClNO3) | IHSZLSTUMANNCN-UHFFFAOYSA-N | CCCOC(=O)CN(C)C(=O)C1=CC=C(C=C1)Cl | 1001 | 0 | 1 | 2 |
| Crufomate (C12H19ClNO3P) | BOFHKBLZOYVHSI-UHFFFAOYSA-N | CC(C)(C)C1=CC(=C(C=C1)OP(=O)(NC)OC)Cl | 1911 | 9999 | 1 | 1 |
| Succinic acid, 3,5-dichlorophenyl ethyl ester (C12H12Cl2O4) | JAUNYACIFVMSCA-UHFFFAOYSA-N | CCOC(=O)CCC(=O)OC1=CC(=CC(=C1)Cl)Cl | 0 | 0 | 2 | 2 |
| 2,4,5-T Butyl ester (C12H13Cl3O3) | FNEXNZUHBCBQTI-UHFFFAOYSA-N | CCCCOC(=O)COC1=CC(=C(C=C1Cl)Cl)Cl | 2502 | 0 | 1 | 2 |
| 2,4,5-t Isobutyl ester (C12H13Cl3O3) | KIIVFWSIMRWBKW-UHFFFAOYSA-N | CC(C)COC(=O)COC1=CC(=C(C=C1Cl)Cl)Cl | 480 | 0 | 2 | 2 |
| 6-Chlorohexanoic acid, 4-nitrophenyl ester (C12H14ClNO4) | ILPNRCAJUCEDJY-UHFFFAOYSA-N | C1=CC(=CC=C1[N+](=O)[O-])OC(=O)CCCCCCl | 0 | 0 | 2 | 2 |
| β-Alanine, N-(2-chlorobenzoyl)-, propyl ester (C13H16ClNO3) | ZLAMXWWURXSZNM-UHFFFAOYSA-N | CCCOC(=O)CCNC(=O)C1=CC=CC=C1Cl | 721 | 100 | 2 | 2 |
| β-Alanine, N-(4-chlorobenzoyl)-, propyl ester (C13H16ClNO3) | MRMFHSQHERDFJF-UHFFFAOYSA-N | CCCOC(=O)CCNC(=O)C1=CC=C(C=C1)Cl | 881 | 0 | 1 | 2 |
| Alachlor (C14H20ClNO2) | XCSGPAVHZFQHGE-UHFFFAOYSA-N | CCC1=C(C(=CC=C1)CC)N(COC)C(=O)CCl | 876 | 359 | 1 | 1 |
| Acetochlor (C14H20ClNO2) | VTNQPKFIQCLBDU-UHFFFAOYSA-N | CCC1=CC=CC(=C1N(COCC)C(=O)CCl)C | 580 | 1610 | 2 | 1 |
| L-Valine, N-(2-chlorobenzoyl)-, methyl ester (C13H16ClNO3) | FUBQWOIKCWKZGE-UHFFFAOYSA-N | CC(C)C(C(=O)OC)NC(=O)C1=CC=CC=C1Cl | 40 | 0 | 2 | 2 |
| Hexanamide, N-ethyl-N-(3-methylphenyl)-6-chloro- (C15H22ClNO) | XOQPJEQXXHERBD-UHFFFAOYSA-N | CCN(C1=CC=CC(=C1)C)C(=O)CCCCCCl | 751 | 671 | 2 | 1 |
| Benzamide, N,N-dibutyl-4-chloro- (C15H22ClNO) | HBSQBZKONFNLBZ-UHFFFAOYSA-N | CCCCN(CCCC)C(=O)C1=CC=C(C=C1)Cl | 520 | 0 | 2 | 2 |
| Octanoic acid, 3,4-dichlorophenyl ester (C14H18Cl2O2) | XRHJWXJKQKYIBL-UHFFFAOYSA-N | CCCCCCCC(=O)OC1=CC(=C(C=C1)Cl)Cl | 631 | 0 | 2 | 2 |
| Octyl 4-chlorobenzoate (C15H21ClO2) | JIAKTPQRGQDKAH-UHFFFAOYSA-N | CCCCCCCCOC(=O)C1=CC=C(C=C1)Cl | 30 | 0 | 2 | 2 |
| Benzoic acid, 2-chloro, octyl ester (C15H21ClO2) | MBYHWDNNJFEZEI-UHFFFAOYSA-N | CCCCCCCCOC(=O)C1=CC=CC=C1Cl | 110 | 0 | 2 | 2 |
| 2,4,6-Triisopropylbenzoyl chloride (C16H23ClO) | OSKNTKJPGKHDHV-UHFFFAOYSA-N | CC(C)C1=CC(=C(C(=C1)C(C)C)C(=O)Cl)C(C)C | 20 | 9999 | 2 | 1 |
| Fumaric acid, 3,5-dichlorophenyl ethyl ester (C12H10Cl2O4) | NTLLZAXEGZZMPE-ONEGZZNKSA-N | CCOC(=O)/C=C/C(=O)OC1=CC(=CC(=C1)Cl)Cl | 80 | 0 | 2 | 2 |
| Fumaric acid, 2-chloro-5-methylphenyl ethyl ester (C13H13ClO4) | DHFVGVUEZZSWGO-VOTSOKGWSA-N | CCOC(=O)/C=C/C(=O)OC1=C(C=CC(=C1)C)Cl | 340 | 60 | 2 | 2 |
| 2-Chlorobenzoic acid, oct-3-en-2-yl ester (C15H19ClO2) | FDHVCFIINZXAOR-RMKNXTFCSA-N | CCCC/C=C/C(C)OC(=O)C1=CC=CC=C1Cl | 40 | 0 | 2 | 2 |
| 4-Chlorobenzoic acid, oct-3-en-2-yl ester (C15H19ClO2) | CKYPWBNNZZHCOB-VOTSOKGWSA-N | CCCC/C=C/C(C)OC(=O)C1=CC=C(C=C1)Cl | 40 | 0 | 2 | 2 |
| Octachloronaphthalene (C10Cl8) | RTNLUFLDZOAXIC-UHFFFAOYSA-N | C12=C(C(=C(C(=C1Cl)Cl)Cl)Cl)C(=C(C(=C2Cl)Cl)Cl)Cl | 3278 | 0 | 1 | 2 |
| Ovex (C12H8Cl2O3S) | RZXLPPRPEOUENN-UHFFFAOYSA-N | C1=CC(=CC=C1OS(=O)(=O)C2=CC=C(C=C2)Cl)Cl | 3413 | 0 | 1 | 2 |
| Genite (C12H8Cl2O3S) | OZFAFGSSMRRTDW-UHFFFAOYSA-N | C1=CC=C(C=C1)S(=O)(=O)OC2=C(C=C(C=C2)Cl)Cl | 2122 | 0 | 1 | 2 |
| nitrofen (C12H7Cl2NO3) | XITQUSLLOSKDTB-UHFFFAOYSA-N | C1=CC(=CC=C1[N+](=O)[O-])OC2=C(C=C(C=C2)Cl)Cl | 9999 | 0 | 1 | 2 |
| Tetrasul sulfoxide (C12H6Cl4OS) | IEPUYXNXNXZXRP-UHFFFAOYSA-N | C1=CC(=CC=C1S(=O)C2=CC(=C(C=C2Cl)Cl)Cl)Cl | 820 | 100 | 1 | 2 |
| Acetamide, N-(3-chlorophenyl)-2-phenylthio- (C14H12ClNOS) | OTOSJZUVCFFPHN-UHFFFAOYSA-N | C1=CC=C(C=C1)SCC(=O)NC2=CC(=CC=C2)Cl | 9999 | 0 | 1 | 2 |
| Phenylthioacetic acid, 4-chlorophenyl ester (C14H11ClO2S) | RODRARCUWYZGJA-UHFFFAOYSA-N | C1=CC=C(C=C1)SCC(=O)OC2=CC=C(C=C2)Cl | 2112 | 0 | 1 | 2 |
| Benzamide, N-(4-methoxyphenyl)-4-chloro- (C14H12ClNO2) | UVGPQLMHIRNFEE-UHFFFAOYSA-N | COC1=CC=C(C=C1)NC(=O)C2=CC=C(C=C2)Cl | 5195 | 0 | 1 | 2 |
| Benzamide, N-(3-chlorophenyl)-3-methoxy- (C14H12ClNO2) | ZJTFEWWZCZJCIL-UHFFFAOYSA-N | COC1=CC=CC(=C1)C(=O)NC2=CC(=CC=C2)Cl | 2382 | 0 | 1 | 2 |
| Benzamide, N-(3-chlorophenyl)-2-methoxy- (C14H12ClNO2) | RLDGBZWAIDMLAE-UHFFFAOYSA-N | COC1=CC=CC=C1C(=O)NC2=CC(=CC=C2)Cl | 1491 | 0 | 1 | 2 |
| Benzamide, N-(3-chlorophenyl)-4-methoxy- (C14H12ClNO2) | SPKOXUOZUKBVLV-UHFFFAOYSA-N | COC1=CC=C(C=C1)C(=O)NC2=CC(=CC=C2)Cl | 1041 | 0 | 1 | 2 |
| m-Methoxybenzoic acid, 2-chlorophenyl ester (C14H11ClO3) | WWVXQWHJNRHRHI-UHFFFAOYSA-N | COC1=CC=CC(=C1)C(=O)OC2=CC=CC=C2Cl | 400 | 0 | 2 | 2 |
| 4-Chlorobenzoic acid, 4-methoxyphenyl ester (C14H11ClO3) | MSZPFWFDFWYVOE-UHFFFAOYSA-N | COC1=CC=C(C=C1)OC(=O)C2=CC=C(C=C2)Cl | 2012 | 0 | 1 | 2 |
| p-Methoxybenzoic acid, 2-chlorophenyl ester (C14H11ClO3) | GQOTVEHMQKIPGS-UHFFFAOYSA-N | COC1=CC=C(C=C1)C(=O)OC2=CC=CC=C2Cl | 70 | 0 | 2 | 2 |
| o-Methoxybenzoic acid, 2-chlorophenyl ester (C14H11ClO3) | SSEUCRYVDZRERD-UHFFFAOYSA-N | COC1=CC=CC=C1C(=O)OC2=CC=CC=C2Cl | 0 | 0 | 2 | 2 |
| p-Anisic acid, 4-chlorophenyl ester (C14H11ClO3) | NUUGTSDUQSAQIU-UHFFFAOYSA-N | COC1=CC=C(C=C1)C(=O)OC2=CC=C(C=C2)Cl | 70 | 0 | 2 | 2 |
| o-Anisic acid, 4-chlorophenyl ester (C14H11ClO3) | BELPBVCDOMAVAK-UHFFFAOYSA-N | COC1=CC=CC=C1C(=O)OC2=CC=C(C=C2)Cl | 30 | 0 | 2 | 2 |
| m-Anisic acid, 4-chlorophenyl ester (C14H11ClO3) | NSYNJRZBHCVYKP-UHFFFAOYSA-N | COC1=CC=CC(=C1)C(=O)OC2=CC=C(C=C2)Cl | 180 | 0 | 2 | 2 |
| 2-Chlorobenzoic acid, 4-methoxyphenyl ester (C14H11ClO3) | LSSGHRSKHZXKOD-UHFFFAOYSA-N | COC1=CC=C(C=C1)OC(=O)C2=CC=CC=C2Cl | 1561 | 0 | 1 | 2 |
| 4-Chlorobenzoic acid, 3,4-dichlorophenyl ester (C13H7Cl3O2) | ANAWVRPOZLXVTH-UHFFFAOYSA-N | C1=CC(=CC=C1C(=O)OC2=CC(=C(C=C2)Cl)Cl)Cl | 210 | 0 | 2 | 2 |
| 2-Chlorobenzoic acid, 3,4-dichlorophenyl ester (C13H7Cl3O2) | MAYPBCZANOCTLF-UHFFFAOYSA-N | C1=CC=C(C(=C1)C(=O)OC2=CC(=C(C=C2)Cl)Cl)Cl | 120 | 0 | 2 | 2 |
| PCB 169 (C12H4Cl6) | ZHLICBPIXDOFFG-UHFFFAOYSA-N | C1=C(C=C(C(=C1Cl)Cl)Cl)C2=CC(=C(C(=C2)Cl)Cl)Cl | 4660 | 125 | 1 | 1 |
| PCB 155 (C12H4Cl6) | ICOAEPDGFWLUTI-UHFFFAOYSA-N | C1=C(C=C(C(=C1Cl)C2=C(C=C(C=C2Cl)Cl)Cl)Cl)Cl | 4903 | 314 | 1 | 1 |
| PCB 153 (C12H4Cl6) | MVWHGTYKUMDIHL-UHFFFAOYSA-N | C1=C(C(=CC(=C1Cl)Cl)Cl)C2=CC(=C(C=C2Cl)Cl)Cl | 7057 | 877 | 1 | 1 |
| PCB 138 (C12H4Cl6) | RPUMZMSNLZHIGZ-UHFFFAOYSA-N | C1=CC(=C(C(=C1C2=CC(=C(C=C2Cl)Cl)Cl)Cl)Cl)Cl | 4954 | 1521 | 1 | 1 |
| PCB 133 (C12H4Cl6) | AJKLKINFZLWHQE-UHFFFAOYSA-N | C1=C(C=C(C(=C1Cl)Cl)C2=CC(=CC(=C2Cl)Cl)Cl)Cl | 5131 | 2629 | 1 | 1 |
| PCB 137 (C12H4Cl6) | CKLLRBPBZLTGDJ-UHFFFAOYSA-N | C1=CC(=C(C=C1Cl)Cl)C2=CC(=C(C(=C2Cl)Cl)Cl)Cl | 4710 | 1180 | 1 | 1 |
| PCB 149 (C12H4Cl6) | LKHLFUVHHXCNJH-UHFFFAOYSA-N | C1=CC(=C(C(=C1Cl)C2=CC(=C(C=C2Cl)Cl)Cl)Cl)Cl | 5006 | 1624 | 1 | 1 |
| PCB 132 (C12H4Cl6) | OKBJVIVEFXPEOU-UHFFFAOYSA-N | C1=CC(=C(C(=C1C2=C(C=CC(=C2Cl)Cl)Cl)Cl)Cl)Cl | 4754 | 2261 | 1 | 1 |
| PCB 128 (C12H4Cl6) | BTAGRXWGMYTPBY-UHFFFAOYSA-N | C1=CC(=C(C(=C1C2=C(C(=C(C=C2)Cl)Cl)Cl)Cl)Cl)Cl | 5359 | 2039 | 1 | 1 |
| PCB 156 (C12H4Cl6) | LCXMEXLGMKFLQO-UHFFFAOYSA-N | C1=CC(=C(C=C1C2=CC(=C(C(=C2Cl)Cl)Cl)Cl)Cl)Cl | 5024 | 48 | 1 | 2 |
| PCB 136 (C12H4Cl6) | FZFUUSROAHKTTF-UHFFFAOYSA-N | C1=CC(=C(C(=C1Cl)C2=C(C=CC(=C2Cl)Cl)Cl)Cl)Cl | 4839 | 259 | 1 | 1 |
| PCB 160 (C12H4Cl6) | JHJMZCXLJXRCHK-UHFFFAOYSA-N | C1=CC(=CC(=C1)Cl)C2=C(C(=C(C(=C2Cl)Cl)Cl)Cl)Cl | 5483 | 143 | 1 | 1 |
| PCB 146 (C12H4Cl6) | BQHCQAQLTCQFJZ-UHFFFAOYSA-N | C1=C(C=C(C(=C1Cl)Cl)C2=CC(=C(C=C2Cl)Cl)Cl)Cl | 4329 | 1164 | 1 | 1 |
| PCB 151 (C12H4Cl6) | UHCLFIWDCYOTOL-UHFFFAOYSA-N | C1=CC(=C(C=C1Cl)C2=C(C(=CC(=C2Cl)Cl)Cl)Cl)Cl | 4905 | 1796 | 1 | 1 |
| PCB 130 (C12H4Cl6) | YFSLABAYQDPWPF-UHFFFAOYSA-N | C1=CC(=C(C(=C1C2=CC(=CC(=C2Cl)Cl)Cl)Cl)Cl)Cl | 5142 | 2619 | 1 | 1 |
| PCB 167 (C12H4Cl6) | AZXHAWRMEPZSSV-UHFFFAOYSA-N | C1=C(C=C(C(=C1Cl)Cl)Cl)C2=CC(=C(C=C2Cl)Cl)Cl | 4825 | 33 | 1 | 2 |
| PCB 134 (C12H4Cl6) | RVWLHPJFOKUPNM-UHFFFAOYSA-N | C1=CC(=C(C(=C1)Cl)Cl)C2=C(C(=CC(=C2Cl)Cl)Cl)Cl | 4640 | 2798 | 1 | 1 |
| PCB 141 (C12H4Cl6) | UCLKLGIYGBLTSM-UHFFFAOYSA-N | C1=CC(=C(C=C1Cl)C2=CC(=C(C(=C2Cl)Cl)Cl)Cl)Cl | 4911 | 1619 | 1 | 1 |
| PCB 135 (C12H4Cl6) | UUTNFLRSJBQQJM-UHFFFAOYSA-N | C1=CC(=C(C(=C1Cl)C2=CC(=CC(=C2Cl)Cl)Cl)Cl)Cl | 4792 | 2701 | 1 | 1 |
| PCB 129 (C12H4Cl6) | VQQKIXKPMJTUMP-UHFFFAOYSA-N | C1=CC(=C(C(=C1)Cl)Cl)C2=CC(=C(C(=C2Cl)Cl)Cl)Cl | 4497 | 2044 | 1 | 1 |
| PCB 139 (C12H4Cl6) | SPOPSCCFZQFGDL-UHFFFAOYSA-N | C1=CC(=C(C=C1Cl)Cl)C2=C(C(=C(C=C2Cl)Cl)Cl)Cl | 4919 | 1276 | 1 | 1 |
| PCB 154 (C12H4Cl6) | QXZHEJWDLVUFFB-UHFFFAOYSA-N | C1=C(C=C(C(=C1Cl)C2=CC(=C(C=C2Cl)Cl)Cl)Cl)Cl | 5247 | 936 | 1 | 1 |
| PCB 131 (C12H4Cl6) | WDLTVNWWEZJMPF-UHFFFAOYSA-N | C1=CC(=C(C(=C1)Cl)Cl)C2=C(C(=C(C=C2Cl)Cl)Cl)Cl | 4949 | 2496 | 1 | 1 |
| PCB 150 (C12H4Cl6) | RPPNJBZNXQNKNM-UHFFFAOYSA-N | C1=CC(=C(C(=C1Cl)C2=C(C=C(C=C2Cl)Cl)Cl)Cl)Cl | 4480 | 211 | 1 | 1 |
| PCB 147 (C12H4Cl6) | AQONCPKMJSBHQT-UHFFFAOYSA-N | C1=CC(=C(C=C1Cl)Cl)C2=C(C(=CC(=C2Cl)Cl)Cl)Cl | 5343 | 2213 | 1 | 1 |
| PCB 143 (C12H4Cl6) | UQPQKLGBEKEZBV-UHFFFAOYSA-N | C1=CC(=C(C(=C1)Cl)C2=CC(=C(C(=C2Cl)Cl)Cl)Cl)Cl | 5122 | 1884 | 1 | 1 |
| PCB 157 (C12H4Cl6) | YTWXDQVNPCIEOX-UHFFFAOYSA-N | C1=CC(=C(C(=C1C2=CC(=C(C(=C2)Cl)Cl)Cl)Cl)Cl)Cl | 4395 | 0 | 1 | 2 |
| 4-Chloro-3-nitrobenzophenone (C13H8ClNO3) | YBDBYPQFIMSFJW-UHFFFAOYSA-N | C1=CC=C(C=C1)C(=O)C2=CC(=C(C=C2)Cl)[N+](=O)[O-] | 2856 | 173 | 1 | 1 |
| 4-Cyanobenzoic acid, 2-chlorophenyl ester (C14H8ClNO2) | WNVUMPBLXYWKLI-UHFFFAOYSA-N | C1=CC=C(C(=C1)OC(=O)C2=CC=C(C=C2)C#N)Cl | 380 | 140 | 2 | 1 |
| 4-Chlorobenzoic acid, 4-cyanophenyl ester (C14H8ClNO2) | PGPBOUBXPMLQLM-UHFFFAOYSA-N | C1=CC(=CC=C1C#N)OC(=O)C2=CC=C(C=C2)Cl | 30 | 0 | 2 | 2 |
| 2-Chlorobenzoic acid, 4-cyanophenyl ester (C14H8ClNO2) | FBJQDKRQROMXNN-UHFFFAOYSA-N | C1=CC=C(C(=C1)C(=O)OC2=CC=C(C=C2)C#N)Cl | 0 | 0 | 2 | 2 |
| N,N-Bis(4-chlorobenzyl)hydroxylamine (C14H13Cl2NO) | LJISSWQMTWDSGS-UHFFFAOYSA-N | C1=CC(=CC=C1CN(CC2=CC=C(C=C2)Cl)O)Cl | 0 | 0 | 2 | 2 |
| 3',4'-Dichlorophenylacetanilide (C14H11Cl2NO) | WOLDJAKASQLODM-UHFFFAOYSA-N | C1=CC=C(C=C1)CC(=O)NC2=CC(=C(C=C2)Cl)Cl | 1628 | 0 | 1 | 2 |
| Phenylacetic acid, 3,4-dichlorophenyl ester (C14H10Cl2O2) | FUOMZHNWKYXFPG-UHFFFAOYSA-N | C1=CC=C(C=C1)CC(=O)OC2=CC(=C(C=C2)Cl)Cl | 30 | 0 | 2 | 2 |
| 2-Methylamino-5,2'-dichlorobenzophenone (C14H11Cl2NO) | DQYHYPVCQHTLRO-UHFFFAOYSA-N | CNC1=C(C=C(C=C1)Cl)C(=O)C2=CC=CC=C2Cl | 9999 | 9030 | 1 | 1 |
| o-Toluylic acid, 3,4-dichlorophenyl ester (C14H10Cl2O2) | KJYZJTRREBNGNX-UHFFFAOYSA-N | CC1=CC=CC=C1C(=O)OC2=CC(=C(C=C2)Cl)Cl | 20 | 0 | 2 | 2 |
| m-Toluylic acid, 3,4-dichlorophenyl ester (C14H10Cl2O2) | YVULESXWFJKJNZ-UHFFFAOYSA-N | CC1=CC=CC(=C1)C(=O)OC2=CC(=C(C=C2)Cl)Cl | 130 | 0 | 2 | 2 |
| p-Toluylic acid, 3,4-dichlorophenyl ester (C14H10Cl2O2) | AVIKUTDZLPGICI-UHFFFAOYSA-N | CC1=CC=C(C=C1)C(=O)OC2=CC(=C(C=C2)Cl)Cl | 90 | 0 | 2 | 2 |
| 1-(2'-Chloro-5'-sulfophenyl)-3-methyl-5-pyrazolone (C10H9ClN2O4S) | UWLNKHDLVZEYKQ-UHFFFAOYSA-N | CC1=NN(C(=O)C1)C2=C(C=CC(=C2)S(=O)(=O)O)Cl | 7919 | 9999 | 1 | 1 |
| Propanamide, N-(3-chlorophenyl)-3-phenyl- (C15H14ClNO) | WNWRTBIACAYQKW-UHFFFAOYSA-N | C1=CC=C(C=C1)CCC(=O)NC2=CC(=CC=C2)Cl | 4214 | 0 | 1 | 2 |
| 2-Phenylethyl 2-chlorobenzoate (C15H13ClO2) | FANZVPUMGJHASY-UHFFFAOYSA-N | C1=CC=C(C=C1)CCOC(=O)C2=CC=CC=C2Cl | 0 | 0 | 2 | 2 |
| 3-Phenylpropionic acid, 4-chlorophenyl ester (C15H13ClO2) | KYCIZZNPNUORSI-UHFFFAOYSA-N | C1=CC=C(C=C1)CCC(=O)OC2=CC=C(C=C2)Cl | 1141 | 0 | 1 | 2 |
| 2-Phenylethyl 4-chlorobenzoate (C15H13ClO2) | CLORGXVHZOTBAZ-UHFFFAOYSA-N | C1=CC=C(C=C1)CCOC(=O)C2=CC=C(C=C2)Cl | 0 | 0 | 2 | 2 |
| Benzamide, N-(3-chlorophenyl)-4-ethyl- (C15H14ClNO) | QUCAEGXNFCWNHP-UHFFFAOYSA-N | CCC1=CC=C(C=C1)C(=O)NC2=CC(=CC=C2)Cl | 1351 | 0 | 1 | 2 |
| 4-Ethylbenzoic acid, 4-chlorophenyl ester (C15H13ClO2) | YSLISJQPFLHFPN-UHFFFAOYSA-N | CCC1=CC=C(C=C1)C(=O)OC2=CC=C(C=C2)Cl | 120 | 0 | 2 | 2 |
| 4-Chlorobenzoic acid, 3,5-dimethylphenyl ester (C15H13ClO2) | QAEMEZNLFVUKMH-UHFFFAOYSA-N | CC1=CC(=CC(=C1)OC(=O)C2=CC=C(C=C2)Cl)C | 1191 | 0 | 1 | 2 |
| 2-Chlorobenzoic acid, 3,5-dimethylphenyl ester (C15H13ClO2) | HHSLGRLKQCSCBW-UHFFFAOYSA-N | CC1=CC(=CC(=C1)OC(=O)C2=CC=CC=C2Cl)C | 1021 | 0 | 1 | 2 |
| Acetic acid, bis-(4-chlorophenyl) (C14H10Cl2O2) | YIOCIFXUGBYCJR-UHFFFAOYSA-N | C1=CC(=CC=C1C(C2=CC=C(C=C2)Cl)C(=O)O)Cl | 1946 | 0 | 1 | 2 |
| 2,2'-Dichlorobenzil (C14H8Cl2O2) | VOSNNSVWVJFJCR-UHFFFAOYSA-N | C1=CC=C(C(=C1)C(=O)C(=O)C2=CC=CC=C2Cl)Cl | 20 | 70 | 2 | 2 |
| o,p'-DDD (C14H10Cl4) | JWBOIMRXGHLCPP-UHFFFAOYSA-N | C1=CC=C(C(=C1)C(C2=CC=C(C=C2)Cl)C(Cl)Cl)Cl | 330 | 68 | 2 | 2 |
| p,p'-DDD (C14H10Cl4) | AHJKRLASYNVKDZ-UHFFFAOYSA-N | C1=CC(=CC=C1C(C2=CC=C(C=C2)Cl)C(Cl)Cl)Cl | 333 | 83 | 2 | 2 |
| Moclobemide (C13H17ClN2O2) | YHXISWVBGDMDLQ-UHFFFAOYSA-N | C1COCCN1CCNC(=O)C2=CC=C(C=C2)Cl | 10 | 0 | 2 | 2 |
| Pentanamide, N-(1-naphthyl)-5-chloro- (C15H16ClNO) | ICQIPXNEVQEEDC-UHFFFAOYSA-N | C1=CC=C2C(=C1)C=CC=C2NC(=O)CCCCCl | 1261 | 120 | 1 | 1 |
| 5-Chlorovaleric acid, 2-naphthyl ester (C15H15ClO2) | ZCUGJMUORSUDHD-UHFFFAOYSA-N | C1=CC=C2C=C(C=CC2=C1)OC(=O)CCCCCl | 370 | 0 | 2 | 2 |
| Clobenzorex (C16H18ClN) | LRXXRIXDSAEIOR-UHFFFAOYSA-N | CC(CC1=CC=CC=C1)NCC2=CC=CC=C2Cl | 10 | 0 | 2 | 2 |
| 3-Cyclopentylpropionic acid, 3,4-dichlorophenyl ester (C14H16Cl2O2) | JPOVKVUFJRAFMA-UHFFFAOYSA-N | C1CCC(C1)CCC(=O)OC2=CC(=C(C=C2)Cl)Cl | 661 | 0 | 2 | 2 |
| o,p'-DDE (C14H8Cl4) | ZDYJWDIWLRZXDB-UHFFFAOYSA-N | C1=CC=C(C(=C1)C(=C(Cl)Cl)C2=CC=C(C=C2)Cl)Cl | 3238 | 532 | 1 | 1 |
| p,p'-DDE (C14H8Cl4) | UCNVFOCBFJOQAL-UHFFFAOYSA-N | C1=CC(=CC=C1C(=C(Cl)Cl)C2=CC=C(C=C2)Cl)Cl | 5477 | 791 | 1 | 1 |
| Vinclozolin (C12H9Cl2NO3) | FSCWZHGZWWDELK-UHFFFAOYSA-N | CC1(C(=O)N(C(=O)O1)C2=CC(=CC(=C2)Cl)Cl)C=C | 9906 | 0 | 1 | 2 |
| Captafol (C10H9Cl4NO2S) | JHRWWRDRBPCWTF-UHFFFAOYSA-N | C1C=CCC2C1C(=O)N(C2=O)SC(C(Cl)Cl)(Cl)Cl | 467 | 849 | 2 | 1 |
| 2,3,7,8-Tetrachlorodibenzo-p-dioxin (C12H4Cl4O2) | HGUFODBRKLSHSI-UHFFFAOYSA-N | C1=C2C(=CC(=C1Cl)Cl)OC3=CC(=C(C=C3O2)Cl)Cl | 8007 | 700 | 1 | 1 |
| Dibenzo[b,e][1,4]dioxin, 1,2,3,4-tetrachloro- (C12H4Cl4O2) | DJHHDLMTUOLVHY-UHFFFAOYSA-N | C1=CC=C2C(=C1)OC3=C(O2)C(=C(C(=C3Cl)Cl)Cl)Cl | 7343 | 260 | 1 | 1 |
| 1,3,6,8-Tetrachlorodibenzo-p-dioxin (C12H4Cl4O2) | OTQFXRBLGNEOGH-UHFFFAOYSA-N | C1=C(C=C2C(=C1Cl)OC3=CC(=CC(=C3O2)Cl)Cl)Cl | 7550 | 1350 | 1 | 1 |
| 1,3,7,8-Tetrachlorodibenzo-p-dioxin (C12H4Cl4O2) | VPTDIAYLYJBYQG-UHFFFAOYSA-N | C1=C(C=C2C(=C1Cl)OC3=CC(=C(C=C3O2)Cl)Cl)Cl | 7830 | 950 | 1 | 1 |
| 1,3,7,9-Tetrachlorodibenzo-p-dioxin (C12H4Cl4O2) | JMGYHLJVDHUACM-UHFFFAOYSA-N | C1=C(C=C2C(=C1Cl)OC3=C(C=C(C=C3O2)Cl)Cl)Cl | 7830 | 1030 | 1 | 1 |
| 7H-Purine, 7-benzyl-2,6-dichloro- (C12H8Cl2N4) | CUYCHULDTCBWLI-UHFFFAOYSA-N | C1=CC=C(C=C1)CN2C=NC3=C2N=C(N=C3Cl)Cl | 1288 | 0 | 1 | 2 |
| 1,2,3,7,8-Pentachlorodibenzofuran (C12H3Cl5O) | SBMIVUVRFPGOEB-UHFFFAOYSA-N | C1=C2C(=CC(=C1Cl)Cl)OC3=CC(=C(C(=C23)Cl)Cl)Cl | 6299 | 210 | 1 | 1 |
| 1H-Isoindole-1,3(2H)-dione, 2-(2-chlorophenyl)- (C14H8ClNO2) | HZJTXHXNUXBSFD-UHFFFAOYSA-N | C1=CC=C2C(=C1)C(=O)N(C2=O)C3=CC=CC=C3Cl | 300 | 9999 | 2 | 1 |
| N-(2-Chloroethyl)-1,8-naphthalimide (C14H10ClNO2) | AWVXDWQRGJLTBI-UHFFFAOYSA-N | C1=CC2=C3C(=C1)C(=O)N(C(=O)C3=CC=C2)CCCl | 9652 | 7318 | 1 | 1 |
| Desmethylmedazepam (C15H13ClN2) | JZWOKDTXYPEJEW-UHFFFAOYSA-N | C1CN=C(C2=C(N1)C=CC(=C2)Cl)C3=CC=CC=C3 | 7307 | 0 | 1 | 2 |
| 9,10-Anthracenedione, 1-amino-5-chloro- (C14H8ClNO2) | QIHMGEKACAOTPE-UHFFFAOYSA-N | C1=CC2=C(C(=C1)N)C(=O)C3=C(C2=O)C(=CC=C3)Cl | 9999 | 210 | 1 | 1 |
| 9,10-Anthracenedione, 2-amino-3-chloro- (C14H8ClNO2) | VMOJFUJVEWWUAV-UHFFFAOYSA-N | C1=CC=C2C(=C1)C(=O)C3=CC(=C(C=C3C2=O)Cl)N | 9999 | 0 | 1 | 2 |
| 9,10-Anthracenedione, 1,8-dichloro- (C14H6Cl2O2) | VBQNYYXVDQUKIU-UHFFFAOYSA-N | C1=CC2=C(C(=C1)Cl)C(=O)C3=C(C2=O)C=CC=C3Cl | 9999 | 4379 | 1 | 1 |
| 9,10-Anthracenedione, 1,5-dichloro- (C14H6Cl2O2) | MQIUMARJCOGCIM-UHFFFAOYSA-N | C1=CC2=C(C(=C1)Cl)C(=O)C3=C(C2=O)C(=CC=C3)Cl | 9999 | 3019 | 1 | 1 |
| Cyclopropanecarbonitrile, 1-(p-chlorophenyl)-2-phenyl- (C16H12ClN) | WNUOJHANAJTNCJ-UHFFFAOYSA-N | C1C(C1(C#N)C2=CC=C(C=C2)Cl)C3=CC=CC=C3 | 6005 | 9999 | 1 | 1 |
| 3-(3,5-dichlorophenyl)-1,5-dimethyl-3-azabicyclo[3.1.0]hexane-2,4-dione (C14H11Cl2NO2) | QXJKBPAVAHBARF-UHFFFAOYSA-N | CC12CC1(C(=O)N(C2=O)C3=CC(=CC(=C3)Cl)Cl)C | 9999 | 35 | 1 | 2 |
| 2H-1,3-Benzoxazine, 6-chloro-3-cyclohexyl-3,4-dihydro-8-methyl- (C15H20ClNO) | HLFBMVRLQWNGLY-UHFFFAOYSA-N | CC1=C2C(=CC(=C1)Cl)CN(CO2)C3CCCCC3 | 0 | 0 | 2 | 2 |
| 5-chlorovaleric acid, 2-adamantyl ester (C15H23ClO2) | XEDXKFLTKHZQND-UHFFFAOYSA-N | C1C2CC3CC1CC(C2)C3OC(=O)CCCCCl | 60 | 0 | 2 | 2 |
| N-(p-Chlorophenyl)-4-cyclohexene-1,2-dicarboxamide (C14H12ClNO2) | LVBNTQRVWHUJOP-UHFFFAOYSA-N | C1C=CCC2C1C(=O)N(C2=O)C3=CC=C(C=C3)Cl | 4203 | 0 | 1 | 2 |
| Chlorendic anhydride (C9H2Cl6O3) | FLBJFXNAEMSXGL-UHFFFAOYSA-N | C12C(C(=O)OC1=O)C3(C(=C(C2(C3(Cl)Cl)Cl)Cl)Cl)Cl | 420 | 670 | 2 | 1 |
| Isobenzan (C9H4Cl8O) | LRWHHSXTGZSMSN-UHFFFAOYSA-N | C12C(C(OC1Cl)Cl)C3(C(=C(C2(C3(Cl)Cl)Cl)Cl)Cl)Cl | 103 | 747 | 2 | 1 |
| Chlordane (C10H6Cl8) | BIWJNBZANLAXMG-UHFFFAOYSA-N | C1C2C(C(C1Cl)Cl)C3(C(=C(C2(C3(Cl)Cl)Cl)Cl)Cl)Cl | 257 | 5050 | 2 | 1 |
| heptachlor epoxide (C10H5Cl7O) | ZXFXBSWRVIQKOD-UHFFFAOYSA-N | C12C(C(C3C1O3)Cl)C4(C(=C(C2(C4(Cl)Cl)Cl)Cl)Cl)Cl | 408 | 4837 | 2 | 1 |
| Succinic acid, isohexyl 2,2,2-trichloroethyl ester (C12H19Cl3O4) | ZFKJXSGRVYKTJB-UHFFFAOYSA-N | CC(C)CCCOC(=O)CCC(=O)OCC(Cl)(Cl)Cl | 0 | 0 | 2 | 2 |
| Malonic acid, 2,2-dichloroethyl octyl ester (C13H22Cl2O4) | PLAYDBGXBJBHEE-UHFFFAOYSA-N | CCCCCCCCOC(=O)CC(=O)OCC(Cl)Cl | 0 | 80 | 2 | 2 |
| Succinic acid, 2,2-dichloroethyl heptyl ester (C13H22Cl2O4) | XYHOOJUYXZQYME-UHFFFAOYSA-N | CCCCCCCOC(=O)CCC(=O)OCC(Cl)Cl | 0 | 0 | 2 | 2 |
| Succinic acid, 8-chloroctyl ethyl ester (C14H25ClO4) | SJXVGPNAVQCMJO-UHFFFAOYSA-N | CCOC(=O)CCC(=O)OCCCCCCCCCl | 0 | 20 | 2 | 2 |
| Malonic acid, 8-chlorooctyl propyl ester (C14H25ClO4) | CSXNAOIJZYGGFP-UHFFFAOYSA-N | CCCOC(=O)CC(=O)OCCCCCCCCCl | 0 | 0 | 2 | 2 |
| D-Alanine, N-(5-chlorovaleryl)-, hexyl ester (C14H26ClNO3) | JAYPLEGBRSWVSE-UHFFFAOYSA-N | CCCCCCOC(=O)C(C)NC(=O)CCCCCl | 0 | 300 | 2 | 1 |
| Malonic acid, 2-chloropropyl octyl ester (C14H25ClO4) | DFJKJUMOXRTFSC-UHFFFAOYSA-N | CCCCCCCCOC(=O)CC(=O)OCC(C)Cl | 0 | 0 | 2 | 2 |
| Succinic acid, 2-chloropropyl heptyl ester (C14H25ClO4) | RUGSBADMELSWSF-UHFFFAOYSA-N | CCCCCCCOC(=O)CCC(=O)OCC(C)Cl | 0 | 0 | 2 | 2 |
| D-Alanine, N-(5-chlorovaleryl)-, isohexyl ester (C14H26ClNO3) | GNZLWQRTBQAPBZ-UHFFFAOYSA-N | CC(C)CCCOC(=O)C(C)NC(=O)CCCCCl | 0 | 340 | 2 | 1 |
| Trichloroacetamide, N,N-dihexyl- (C14H26Cl3NO) | KUPGOOKIYUHFCE-UHFFFAOYSA-N | CCCCCCN(CCCCCC)C(=O)C(Cl)(Cl)Cl | 0 | 2172 | 2 | 1 |
| Dodecyl trichloroacetate (C14H25Cl3O2) | LFBVFMYQESVNJY-UHFFFAOYSA-N | CCCCCCCCCCCCOC(=O)C(Cl)(Cl)Cl | 0 | 0 | 2 | 2 |
| Dichloroacetic acid, tridec-2-ynyl ester (C15H24Cl2O2) | DVLQUHLPIXUEAP-UHFFFAOYSA-N | CCCCCCCCCCC#CCOC(=O)C(Cl)Cl | 0 | 30 | 2 | 2 |
| Chloroacetamide, N,N-diheptyl- (C16H32ClNO) | ZOFPQFIAAZHFAY-UHFFFAOYSA-N | CCCCCCCN(CCCCCCC)C(=O)CCl | 60 | 6526 | 2 | 1 |
| 3-Chloropropionic acid, tridec-2-ynyl ester (C16H27ClO2) | FWHBIDZGLNVDFD-UHFFFAOYSA-N | CCCCCCCCCCC#CCOC(=O)CCCl | 0 | 150 | 2 | 1 |
| Tetradecyl chloroacetate (C16H31ClO2) | INPWKHSGGJNIIM-UHFFFAOYSA-N | CCCCCCCCCCCCCCOC(=O)CCl | 0 | 0 | 2 | 2 |
| 2-chloroethyl tetradecanoate (C16H31ClO2) | MPNMNLLETRXDEX-UHFFFAOYSA-N | CCCCCCCCCCCCCC(=O)OCCCl | 1489 | 60 | 1 | 2 |
| 5-chlorovaleric acid, undecyl ester (C16H31ClO2) | MZLDFDLDFGBNEV-UHFFFAOYSA-N | CCCCCCCCCCCOC(=O)CCCCCl | 0 | 190 | 2 | 1 |
| 1-Chlorooctadecane (C18H37Cl) | VUQPJRPDRDVQMN-UHFFFAOYSA-N | CCCCCCCCCCCCCCCCCCCl | 209 | 20 | 2 | 2 |
| Fumaric acid, 8-chlorooctyl ethyl ester (C14H23ClO4) | PZWWEXQGKBYUIG-MDZDMXLPSA-N | CCOC(=O)/C=C/C(=O)OCCCCCCCCCl | 0 | 30 | 2 | 2 |
| Fumaric acid, 2-chloropropyl heptyl ester (C14H23ClO4) | KFSJHJRXOISCGF-CMDGGOBGSA-N | CCCCCCCOC(=O)/C=C/C(=O)OCC(C)Cl | 0 | 30 | 2 | 2 |
| 5-chlorovaleric acid, 2,6-dimethylnon-1-en-3-yn-5-yl ester (C16H25ClO2) | QPMAFXVSIZPLAK-UHFFFAOYSA-N | CCCC(C)C(C#CC(=C)C)OC(=O)CCCCCl | 270 | 40 | 2 | 2 |
| 5-chlorovaleric acid, undec-2-enyl ester (C16H29ClO2) | MMBYQPWSFIURFR-FMIVXFBMSA-N | CCCCCCCC/C=C/COC(=O)CCCCCl | 90 | 40 | 2 | 2 |
| Carbophenothion O-analog sulfoxide (C11H16ClO4PS2) | XQKGIZXBDUMZBY-UHFFFAOYSA-N | CCOP(=O)(OCC)SCS(=O)C1=CC=C(C=C1)Cl | 0 | 0 | 2 | 2 |
| Carbophenothion sulfoxide (C11H16ClO3PS3) | LAHNDTNSNGVHPJ-UHFFFAOYSA-N | CCOP(=S)(OCC)SCS(=O)C1=CC=C(C=C1)Cl | 0 | 0 | 2 | 2 |
| Erbon (C11H9Cl5O3) | KMHZPJNVPCAUMN-UHFFFAOYSA-N | CC(C(=O)OCCOC1=CC(=C(C=C1Cl)Cl)Cl)(Cl)Cl | 283 | 0 | 2 | 2 |
| L-Methionine, N-(2-chlorobenzoyl)-, methyl ester (C13H16ClNO3S) | VTGMWNUUHTXXLY-UHFFFAOYSA-N | COC(=O)C(CCSC)NC(=O)C1=CC=CC=C1Cl | 280 | 0 | 2 | 2 |
| Succinic acid, ethyl 2,3,5-trichlorophenyl ester (C12H11Cl3O4) | DMGJAPZJPKIMQE-UHFFFAOYSA-N | CCOC(=O)CCC(=O)OC1=CC(=CC(=C1Cl)Cl)Cl | 0 | 0 | 2 | 2 |
| Succinic acid, ethyl 2,3,6-trichlorophenyl ester (C12H11Cl3O4) | VQAWDMHUBWVGEG-UHFFFAOYSA-N | CCOC(=O)CCC(=O)OC1=C(C=CC(=C1Cl)Cl)Cl | 0 | 0 | 2 | 2 |
| Sarcosine, N-(2-chlorobenzoyl)-, butyl ester (C14H18ClNO3) | XKYYSPFBLCOTSW-UHFFFAOYSA-N | CCCCOC(=O)CN(C)C(=O)C1=CC=CC=C1Cl | 1191 | 80 | 1 | 2 |
| Sarcosine, N-(4-chlorobenzoyl)-, butyl ester (C14H18ClNO3) | SRIOEDMSFBSNRU-UHFFFAOYSA-N | CCCCOC(=O)CN(C)C(=O)C1=CC=C(C=C1)Cl | 961 | 0 | 1 | 2 |
| Sarcosine, N-(4-chlorobenzoyl)-, isobutyl ester (C14H18ClNO3) | BIRRTJZKUPXLRD-UHFFFAOYSA-N | CC(C)COC(=O)CN(C)C(=O)C1=CC=C(C=C1)Cl | 1141 | 0 | 1 | 2 |
| Succinic acid, 3,5-dichlorophenyl propyl ester (C13H14Cl2O4) | GPPPPAWRSWZRLL-UHFFFAOYSA-N | CCCOC(=O)CCC(=O)OC1=CC(=CC(=C1)Cl)Cl | 0 | 0 | 2 | 2 |
| β-Alanine, N-(2-chlorobenzoyl)-, butyl ester (C14H18ClNO3) | VUQQMELHTPWSLV-UHFFFAOYSA-N | CCCCOC(=O)CCNC(=O)C1=CC=CC=C1Cl | 831 | 100 | 1 | 2 |
| β-Alanine, N-(4-chlorobenzoyl)-, butyl ester (C14H18ClNO3) | GQSWAGGIXFHPKC-UHFFFAOYSA-N | CCCCOC(=O)CCNC(=O)C1=CC=C(C=C1)Cl | 931 | 0 | 1 | 2 |
| 2-chloro-2'-ethyl-N-(2-methoxy-1-methylethyl)-6'-methylacetanilide (C15H22ClNO2) | WVQBLGZPHOPPFO-UHFFFAOYSA-N | CCC1=CC=CC(=C1N(C(C)COC)C(=O)CCl)C | 18 | 0 | 2 | 2 |
| Chlorambucil (C14H19Cl2NO2) | JCKYGMPEJWAADB-UHFFFAOYSA-N | C1=CC(=CC=C1CCCC(=O)O)N(CCCl)CCCl | 958 | 169 | 1 | 1 |
| 2,4-DB, butyl ester (C14H18Cl2O3) | IXXKVXJYFVAQBI-UHFFFAOYSA-N | CCCCOC(=O)CCCOC1=C(C=C(C=C1)Cl)Cl | 40 | 0 | 2 | 2 |
| 2-Chlorobenzoic acid, 2-methyloct-5-yn-4-yl ester (C16H19ClO2) | OYBLJDLPLOGLEE-UHFFFAOYSA-N | CCC#CC(CC(C)C)OC(=O)C1=CC=CC=C1Cl | 0 | 70 | 2 | 2 |
| 4-Chlorobenzoic acid, 2-methyloct-5-yn-4-yl ester (C16H19ClO2) | HTRZUGYDEBUERV-UHFFFAOYSA-N | CCC#CC(CC(C)C)OC(=O)C1=CC=C(C=C1)Cl | 0 | 0 | 2 | 2 |
| Benzoic acid, 2-chloro, nonyl ester (C16H23ClO2) | DTONOUJAHINTOZ-UHFFFAOYSA-N | CCCCCCCCCOC(=O)C1=CC=CC=C1Cl | 60 | 0 | 2 | 2 |
| Nonyl 4-chlorobenzoate (C16H23ClO2) | ZGGCOLIJJWJZNM-UHFFFAOYSA-N | CCCCCCCCCOC(=O)C1=CC=C(C=C1)Cl | 80 | 0 | 2 | 2 |
| cis-Tetrachlorvinphos (C10H9Cl4O4P) | UBCKGWBNUIFUST-YHYXMXQVSA-N | COP(=O)(OC)O/C(=C\Cl)/C1=CC(=C(C=C1Cl)Cl)Cl | 73 | 9840 | 2 | 1 |
| Fumaric acid, ethyl 2,4,5-trichlorophenyl ester (C12H9Cl3O4) | BAHRNZCHZSZBBX-ONEGZZNKSA-N | CCOC(=O)/C=C/C(=O)OC1=CC(=C(C=C1Cl)Cl)Cl | 80 | 0 | 2 | 2 |
| Fumaric acid, ethyl 2,3,5-trichlorophenyl ester (C12H9Cl3O4) | RWERCTSWXBEGQR-ONEGZZNKSA-N | CCOC(=O)/C=C/C(=O)OC1=CC(=CC(=C1Cl)Cl)Cl | 100 | 0 | 2 | 2 |
| Fumaric acid, ethyl 3,4,5-trichlorophenyl ester (C12H9Cl3O4) | NPYGPOLDZDOIEX-ONEGZZNKSA-N | CCOC(=O)/C=C/C(=O)OC1=CC(=C(C(=C1)Cl)Cl)Cl | 70 | 0 | 2 | 2 |
| Fumaric acid, ethyl 2,3,6-trichlorophenyl ester (C12H9Cl3O4) | VXWLCYIYNUHENI-AATRIKPKSA-N | CCOC(=O)/C=C/C(=O)OC1=C(C=CC(=C1Cl)Cl)Cl | 230 | 0 | 2 | 2 |
| Fumaric acid, ethyl 2,4,6-trichlorophenyl ester (C12H9Cl3O4) | ZQUKPRSELNYMAD-ONEGZZNKSA-N | CCOC(=O)/C=C/C(=O)OC1=C(C=C(C=C1Cl)Cl)Cl | 70 | 0 | 2 | 2 |
| Fumaric acid, 3,5-dichlorophenyl propyl ester (C13H12Cl2O4) | GQDYWAPQBXUEDV-ONEGZZNKSA-N | CCCOC(=O)/C=C/C(=O)OC1=CC(=CC(=C1)Cl)Cl | 50 | 0 | 2 | 2 |
| Fumaric acid, 2-chloro-5-methylphenyl propyl ester (C14H15ClO4) | QOJOFIGGVFJBIJ-VOTSOKGWSA-N | CCCOC(=O)/C=C/C(=O)OC1=C(C=CC(=C1)C)Cl | 280 | 60 | 2 | 2 |
| Azamethiphos (C9H10ClN2O5PS) | VNKBTWQZTQIWDV-UHFFFAOYSA-N | COP(=O)(OC)SCN1C2=NC=C(C=C2OC1=O)Cl | 900 | 0 | 1 | 2 |
| Bis(2-chlorophenyl)phosphorochloridate (C12H8Cl3O3P) | ZLSGEMKKBUVQOM-UHFFFAOYSA-N | C1=CC=C(C(=C1)OP(=O)(OC2=CC=CC=C2Cl)Cl)Cl | 2082 | 9999 | 1 | 1 |
| Chlornitrofen (C12H6Cl3NO3) | XQNAUQUKWRBODG-UHFFFAOYSA-N | C1=CC(=CC=C1[N+](=O)[O-])OC2=C(C=C(C=C2Cl)Cl)Cl | 1821 | 10 | 1 | 2 |
| Triclocarban (C13H9Cl3N2O) | ICUTUKXCWQYESQ-UHFFFAOYSA-N | C1=CC(=CC=C1NC(=O)NC2=CC(=C(C=C2)Cl)Cl)Cl | 2009 | 30 | 1 | 2 |
| Bis(3,4-dichlorophenylthio)methane (C13H8Cl4S2) | PVURINFQLWSBRK-UHFFFAOYSA-N | C1=CC(=C(C=C1SCSC2=CC(=C(C=C2)Cl)Cl)Cl)Cl | 1391 | 0 | 1 | 2 |
| Tetradifon (C12H6Cl4O2S) | MLGCXEBRWGEOQX-UHFFFAOYSA-N | C1=CC(=CC=C1S(=O)(=O)C2=CC(=C(C=C2Cl)Cl)Cl)Cl | 3070 | 0 | 1 | 2 |
| Bithionol (C12H6Cl4O2S) | JFIOVJDNOJYLKP-UHFFFAOYSA-N | C1=C(C=C(C(=C1Cl)O)SC2=CC(=CC(=C2O)Cl)Cl)Cl | 1989 | 70 | 1 | 2 |
| 4-Chloro-3-nitrobenzanilide (C13H9ClN2O3) | MPSDMWLZVDJFGW-UHFFFAOYSA-N | C1=CC=C(C=C1)NC(=O)C2=CC(=C(C=C2)Cl)[N+](=O)[O-] | 3235 | 30 | 1 | 2 |
| Benzamide, N-(3-nitrophenyl)-2-chloro- (C13H9ClN2O3) | XJCZQYCXLFPBAM-UHFFFAOYSA-N | C1=CC=C(C(=C1)C(=O)NC2=CC(=CC=C2)[N+](=O)[O-])Cl | 1191 | 0 | 1 | 2 |
| Benzamide, N-(3-chlorophenyl)-4-nitro- (C13H9ClN2O3) | KVVNRXNSICWUSG-UHFFFAOYSA-N | C1=CC(=CC(=C1)Cl)NC(=O)C2=CC=C(C=C2)[N+](=O)[O-] | 3613 | 0 | 1 | 2 |
| 4-Nitrobenzoic acid, 2-chlorophenyl ester (C13H8ClNO4) | OFIWZIJKZNBSHV-UHFFFAOYSA-N | C1=CC=C(C(=C1)OC(=O)C2=CC=C(C=C2)[N+](=O)[O-])Cl | 480 | 150 | 2 | 1 |
| 4-Chlorobenzoic acid, 4-nitrophenyl ester (C13H8ClNO4) | PCOGDBDOULFSKE-UHFFFAOYSA-N | C1=CC(=CC=C1C(=O)OC2=CC=C(C=C2)[N+](=O)[O-])Cl | 0 | 0 | 2 | 2 |
| 2-Chlorobenzoic acid, 4-nitrophenyl ester (C13H8ClNO4) | LYQCUKYZFNQYEG-UHFFFAOYSA-N | C1=CC=C(C(=C1)C(=O)OC2=CC=C(C=C2)[N+](=O)[O-])Cl | 0 | 0 | 2 | 2 |
| 4-Nitrobenzoic acid, 4-chlorophenyl ester (C13H8ClNO4) | FYLVXSFYEBNIKC-UHFFFAOYSA-N | C1=CC(=CC=C1C(=O)OC2=CC=C(C=C2)Cl)[N+](=O)[O-] | 831 | 0 | 1 | 2 |
| Benzoic acid, 4-[(4-chlorophenyl)sulfonyl]- (C13H9ClO4S) | MIVYLPYNSKNBPW-UHFFFAOYSA-N | C1=CC(=CC=C1C(=O)O)S(=O)(=O)C2=CC=C(C=C2)Cl | 2083 | 0 | 1 | 2 |
| Phenylthioacetic acid, 3,4-dichlorophenyl ester (C14H10Cl2O2S) | BDFGZZWWXMDBOI-UHFFFAOYSA-N | C1=CC=C(C=C1)SCC(=O)OC2=CC(=C(C=C2)Cl)Cl | 1872 | 0 | 1 | 2 |
| p-Anisic acid, 3,4-dichlorophenyl ester (C14H10Cl2O3) | FRVXSRVZQSAALV-UHFFFAOYSA-N | COC1=CC=C(C=C1)C(=O)OC2=CC(=C(C=C2)Cl)Cl | 30 | 0 | 2 | 2 |
| o-Anisic acid, 3,4-dichlorophenyl ester (C14H10Cl2O3) | YFVNVFGDQAIOLM-UHFFFAOYSA-N | COC1=CC=CC=C1C(=O)OC2=CC(=C(C=C2)Cl)Cl | 0 | 0 | 2 | 2 |
| m-Anisic acid, 3,4-dichlorophenyl ester (C14H10Cl2O3) | NKZQQSMRSOXSET-UHFFFAOYSA-N | COC1=CC=CC(=C1)C(=O)OC2=CC(=C(C=C2)Cl)Cl | 250 | 0 | 2 | 2 |
| Benzoic acid, 2-(2,4,6-trichlorophenyl)hydrazide (C13H9Cl3N2O) | FWASGTVTBTYRAD-UHFFFAOYSA-N | C1=CC=C(C=C1)C(=O)NNC2=C(C=C(C=C2Cl)Cl)Cl | 2462 | 290 | 1 | 1 |
| PCB 180 (C12H3Cl7) | WBHQEUPUMONIKF-UHFFFAOYSA-N | C1=C(C(=CC(=C1Cl)Cl)Cl)C2=CC(=C(C(=C2Cl)Cl)Cl)Cl | 4378 | 815 | 1 | 1 |
| PCB 170 (C12H3Cl7) | RMPWIIKNWPVWNG-UHFFFAOYSA-N | C1=CC(=C(C(=C1C2=CC(=C(C(=C2Cl)Cl)Cl)Cl)Cl)Cl)Cl | 4473 | 1307 | 1 | 1 |
| PCB 174 (C12H3Cl7) | ZDLMBNHYTPHDLF-UHFFFAOYSA-N | C1=CC(=C(C(=C1Cl)C2=CC(=C(C(=C2Cl)Cl)Cl)Cl)Cl)Cl | 4150 | 1594 | 1 | 1 |
| PCB 189 (C12H3Cl7) | XUAWBXBYHDRROL-UHFFFAOYSA-N | C1=C(C=C(C(=C1Cl)Cl)Cl)C2=CC(=C(C(=C2Cl)Cl)Cl)Cl | 3585 | 0 | 1 | 2 |
| PCB 175 (C12H3Cl7) | KJBDZJFSYQUNJT-UHFFFAOYSA-N | C1=C(C=C(C(=C1Cl)Cl)C2=C(C(=C(C=C2Cl)Cl)Cl)Cl)Cl | 4424 | 1693 | 1 | 1 |
| PCB 190 (C12H3Cl7) | TYEDCFVCFDKSBK-UHFFFAOYSA-N | C1=CC(=C(C=C1C2=C(C(=C(C(=C2Cl)Cl)Cl)Cl)Cl)Cl)Cl | 4485 | 46 | 1 | 2 |
| PCB 179 (C12H3Cl7) | XYHVYEUZLSYHDP-UHFFFAOYSA-N | C1=CC(=C(C(=C1Cl)C2=C(C(=CC(=C2Cl)Cl)Cl)Cl)Cl)Cl | 4641 | 171 | 1 | 1 |
| PCB 176 (C12H3Cl7) | HGMYRFJAJNYBRX-UHFFFAOYSA-N | C1=CC(=C(C(=C1Cl)C2=C(C(=C(C=C2Cl)Cl)Cl)Cl)Cl)Cl | 4620 | 0 | 1 | 2 |
| PCB 178 (C12H3Cl7) | WCIBKXHMIXUQHK-UHFFFAOYSA-N | C1=C(C=C(C(=C1Cl)Cl)C2=C(C(=CC(=C2Cl)Cl)Cl)Cl)Cl | 3938 | 1991 | 1 | 1 |
| PCB 187 (C12H3Cl7) | UDMZPLROONOSEF-UHFFFAOYSA-N | C1=C(C(=CC(=C1Cl)Cl)Cl)C2=C(C(=CC(=C2Cl)Cl)Cl)Cl | 4437 | 1208 | 1 | 1 |
| PCB 183 (C12H3Cl7) | KQBFUDNJKCZEDQ-UHFFFAOYSA-N | C1=C(C(=CC(=C1Cl)Cl)Cl)C2=C(C(=C(C=C2Cl)Cl)Cl)Cl | 4206 | 812 | 1 | 1 |
| PCB 177 (C12H3Cl7) | CXOYNJAHPUASHN-UHFFFAOYSA-N | C1=CC(=C(C(=C1C2=C(C(=CC(=C2Cl)Cl)Cl)Cl)Cl)Cl)Cl | 4319 | 2020 | 1 | 1 |
| PCB 171 (C12H3Cl7) | TZMHVHLTPWKZCI-UHFFFAOYSA-N | C1=CC(=C(C(=C1C2=C(C(=C(C=C2Cl)Cl)Cl)Cl)Cl)Cl)Cl | 4194 | 1907 | 1 | 1 |
| PCB 172 (C12H3Cl7) | HOPMUCXYRNOABF-UHFFFAOYSA-N | C1=C(C=C(C(=C1Cl)Cl)C2=CC(=C(C(=C2Cl)Cl)Cl)Cl)Cl | 4367 | 2069 | 1 | 1 |
| PCB 185 (C12H3Cl7) | PYZHTHZEHQHHEN-UHFFFAOYSA-N | C1=CC(=C(C=C1Cl)C2=C(C(=C(C(=C2Cl)Cl)Cl)Cl)Cl)Cl | 4442 | 1244 | 1 | 1 |
| PCB 182 (C12H3Cl7) | RXRLRYZUMSYVLS-UHFFFAOYSA-N | C1=C(C=C(C(=C1Cl)C2=CC(=C(C(=C2Cl)Cl)Cl)Cl)Cl)Cl | 4389 | 1522 | 1 | 1 |
| PCB 193 (C12H3Cl7) | SSTJUBQGYXNFFP-UHFFFAOYSA-N | C1=C(C=C(C(=C1Cl)Cl)Cl)C2=C(C(=CC(=C2Cl)Cl)Cl)Cl | 4833 | 130 | 1 | 1 |
| PCB 181 (C12H3Cl7) | DJEUXBQAKBLKPO-UHFFFAOYSA-N | C1=CC(=C(C=C1Cl)Cl)C2=C(C(=C(C(=C2Cl)Cl)Cl)Cl)Cl | 4527 | 374 | 1 | 1 |
| PCB 186 (C12H3Cl7) | FGDPOTMRBQHPJK-UHFFFAOYSA-N | C1=CC(=C(C(=C1)Cl)C2=C(C(=C(C(=C2Cl)Cl)Cl)Cl)Cl)Cl | 4425 | 168 | 1 | 1 |
| PCB 192 (C12H3Cl7) | ZUTDUGMNROUBOX-UHFFFAOYSA-N | C1=C(C=C(C=C1Cl)Cl)C2=C(C(=C(C(=C2Cl)Cl)Cl)Cl)Cl | 4641 | 149 | 1 | 1 |
| (Phenylthio)acetic acid, (4-chlorophenyl)methyl ester (C15H13ClO2S) | ZHIZABUTNCVGJX-UHFFFAOYSA-N | C1=CC=C(C=C1)SCC(=O)OCC2=CC=C(C=C2)Cl | 4854 | 0 | 1 | 2 |
| Tolfenamic acid, methyl deriv. (C15H14ClNO2) | CNIPMLJEWLMTCO-UHFFFAOYSA-N | CC1=C(C=CC=C1Cl)NC2=CC=CC=C2C(=O)OC | 4434 | 0 | 1 | 2 |
| Benzene, 1,1'-[sulfonylbis(methylene)]bis[4-chloro- (C14H12Cl2O2S) | JEVYVLLSVHNREX-UHFFFAOYSA-N | C1=CC(=CC=C1CS(=O)(=O)CC2=CC=C(C=C2)Cl)Cl | 195 | 0 | 2 | 2 |
| Diclofenac (C14H11Cl2NO2) | DCOPUUMXTXDBNB-UHFFFAOYSA-N | C1=CC=C(C(=C1)CC(=O)O)NC2=C(C=CC=C2Cl)Cl | 4044 | 10 | 1 | 2 |
| 3-chlorobenzoic anhydride (C14H8Cl2O3) | NIHKFGMYMWGERR-UHFFFAOYSA-N | C1=CC(=CC(=C1)Cl)C(=O)OC(=O)C2=CC(=CC=C2)Cl | 1829 | 10 | 1 | 2 |
| 2,6-Dichlorobenzyl ether (C14H10Cl4O) | NWYHVMDKERUNLM-UHFFFAOYSA-N | C1=CC(=C(C=C1Cl)Cl)COCC2=C(C=C(C=C2)Cl)Cl | 120 | 20 | 2 | 2 |
| Pyrifenox (C14H12Cl2N2O) | CKPCAYZTYMHQEX-UHFFFAOYSA-N | CON=C(CC1=CN=CC=C1)C2=C(C=C(C=C2)Cl)Cl | 1702 | 981 | 1 | 1 |
| 2-amino-5-chlorobenzophenone, acetylated (C15H12ClNO2) | NHAUKYAYIYDFST-UHFFFAOYSA-N | CC(=O)NC1=C(C=C(C=C1)Cl)C(=O)C2=CC=CC=C2 | 1170 | 11 | 1 | 2 |
| Benzamide, N-ethyl-N-(3-methylphenyl)-4-chloro- (C16H16ClNO) | MHIIVAZRXRPBPI-UHFFFAOYSA-N | CCN(C1=CC=CC(=C1)C)C(=O)C2=CC=C(C=C2)Cl | 2422 | 0 | 1 | 2 |
| 3-Phenylpropionic acid, 3,4-dichlorophenyl ester (C15H12Cl2O2) | FQOCOCXQBBVPNZ-UHFFFAOYSA-N | C1=CC=C(C=C1)CCC(=O)OC2=CC(=C(C=C2)Cl)Cl | 601 | 0 | 2 | 2 |
| 4-Ethylbenzoic acid, 3,4-dichlorophenyl ester (C15H12Cl2O2) | UHVGMXBIDBHYKS-UHFFFAOYSA-N | CCC1=CC=C(C=C1)C(=O)OC2=CC(=C(C=C2)Cl)Cl | 40 | 0 | 2 | 2 |
| p,p'-DDT (C14H9Cl5) | YVGGHNCTFXOJCH-UHFFFAOYSA-N | C1=CC(=CC=C1C(C2=CC=C(C=C2)Cl)C(Cl)(Cl)Cl)Cl | 247 | 256 | 2 | 1 |
| o,p'-DDT (C14H9Cl5) | CVUGPAFCQJIYDT-UHFFFAOYSA-N | C1=CC=C(C(=C1)C(C2=CC=C(C=C2)Cl)C(Cl)(Cl)Cl)Cl | 279 | 149 | 2 | 1 |
| n.-Propanamine, 3-(4-chlorophenyl)-3-(2-pyridyl)-N,N-dimethyl- (C16H19ClN2) | SOYKEARSMXGVTM-UHFFFAOYSA-N | CN(C)CCC(C1=CC=C(C=C1)Cl)C2=CC=CC=N2 | 10 | 0 | 2 | 2 |
| Dexchlorpheniramine (C16H19ClN2) | SOYKEARSMXGVTM-UHFFFAOYSA-N | CN(C)CCC(C1=CC=C(C=C1)Cl)C2=CC=CC=N2 | 10 | 0 | 2 | 2 |
| 6-Chlorohexanoic acid, 2-naphthyl ester (C16H17ClO2) | BPYZYGDDNGIIMV-UHFFFAOYSA-N | C1=CC=C2C=C(C=CC2=C1)OC(=O)CCCCCCl | 340 | 0 | 2 | 2 |
| 2-chloro-N-(2,6-dimethylphenyl)-N-(tetrahydro-2-oxo-3-furyl)acetamide (C14H16ClNO3) | OWDLFBLNMPCXSD-UHFFFAOYSA-N | CC1=C(C(=CC=C1)C)N(C2CCOC2=O)C(=O)CCl | 3063 | 230 | 1 | 1 |
| Metazachlor (C14H16ClN3O) | STEPQTYSZVCJPV-UHFFFAOYSA-N | CC1=C(C(=CC=C1)C)N(CN2C=CC=N2)C(=O)CCl | 680 | 65 | 2 | 2 |
| Bicyclo[2.2.1]hept-5-ene-2,3-dicarboxylic acid, 1,4,5,6,7,7-hexachloro- (C9H4Cl6O4) | DJKGDNKYTKCJKD-UHFFFAOYSA-N | C1(C(C2(C(=C(C1(C2(Cl)Cl)Cl)Cl)Cl)Cl)C(=O)O)C(=O)O | 0 | 0 | 2 | 2 |
| Chlorothen (C14H18ClN3S) | XAEXSWVTEJHRMH-UHFFFAOYSA-N | CN(C)CCN(CC1=CC=C(S1)Cl)C2=CC=CC=N2 | 630 | 10 | 2 | 2 |
| Imazalil (C14H14Cl2N2O) | PZBPKYOVPCNPJY-UHFFFAOYSA-N | C=CCOC(CN1C=CN=C1)C2=C(C=C(C=C2)Cl)Cl | 219 | 120 | 2 | 1 |
| 1,2,3,7,8-Pentachlorodibenzodioxin (C12H3Cl5O2) | FSPZPQQWDODWAU-UHFFFAOYSA-N | C1=C2C(=CC(=C1Cl)Cl)OC3=C(C(=C(C=C3O2)Cl)Cl)Cl | 6190 | 600 | 1 | 1 |
| 1,2,4,7,8-Pentachlorodibenzo-p-dioxin (C12H3Cl5O2) | QUPLGUUISJOUPJ-UHFFFAOYSA-N | C1=C2C(=CC(=C1Cl)Cl)OC3=C(O2)C(=CC(=C3Cl)Cl)Cl | 6260 | 680 | 1 | 1 |
| 2,3,4,6,7,8-Hexachlorodibenzofuran (C12H2Cl6O) | XTAHLACQOVXINQ-UHFFFAOYSA-N | C1=C2C3=CC(=C(C(=C3OC2=C(C(=C1Cl)Cl)Cl)Cl)Cl)Cl | 5239 | 79 | 1 | 2 |
| Mecloqualone (C15H11ClN2O) | SFITWQDBYUMAPS-UHFFFAOYSA-N | CC1=NC2=CC=CC=C2C(=O)N1C3=CC=CC=C3Cl | 671 | 9999 | 2 | 1 |
| Norchlordiazepoxide (C15H11ClN2O) | YIXUNVZJCJYWIW-UHFFFAOYSA-N | C1C=NC2=C(C=C(C=C2)Cl)C(=[N+]1[O-])C3=CC=CC=C3 | 4564 | 0 | 1 | 2 |
| Nordiazepam (C15H11ClN2O) | AKPLHCDWDRPJGD-UHFFFAOYSA-N | C1C(=O)NC2=C(C=C(C=C2)Cl)C(=N1)C3=CC=CC=C3 | 9999 | 2769 | 1 | 1 |
| Medazepam (C16H15ClN2) | YLCXGBZIZBEVPZ-UHFFFAOYSA-N | CN1CCN=C(C2=C1C=CC(=C2)Cl)C3=CC=CC=C3 | 3309 | 40 | 1 | 2 |
| Chlorflurenol, methyl ester (C15H11ClO3) | LINPVWIEWJTEEJ-UHFFFAOYSA-N | COC(=O)C1(C2=CC=CC=C2C3=C1C=C(C=C3)Cl)O | 1111 | 0 | 1 | 2 |
| proclonol (C16H14Cl2O) | BKAYSPSVVJBHHK-UHFFFAOYSA-N | C1CC1C(C2=CC=C(C=C2)Cl)(C3=CC=C(C=C3)Cl)O | 303 | 273 | 2 | 1 |
| Cyprofuram (C14H14ClNO3) | KRZUZYJEQBXUIN-UHFFFAOYSA-N | C1CC1C(=O)N(C2CCOC2=O)C3=CC(=CC=C3)Cl | 930 | 0 | 1 | 2 |
| 5-chlorovaleric acid, 1-adamantylmethyl ester (C16H25ClO2) | VEQDGEGLHTXBCJ-UHFFFAOYSA-N | C1C2CC3CC1CC(C2)(C3)COC(=O)CCCCCl | 0 | 0 | 2 | 2 |
| Quinoline, 2-[2-(4-chlorophenyl)ethenyl]- (C17H12ClN) | PWZHLFSCFYMSMR-YRNVUSSQSA-N | C1=CC=C2C(=C1)C=CC(=N2)/C=C/C3=CC=C(C=C3)Cl | 4333 | 570 | 1 | 1 |
| Nor-tetrazepam (C15H15ClN2O) | FDRMSENAXZDFTN-UHFFFAOYSA-N | C1CCC(=CC1)C2=NCC(=O)NC3=C2C=C(C=C3)Cl | 4492 | 9999 | 1 | 1 |
| Endosulfan (C9H6Cl6O3S) | RDYMFSUJUZBWLH-UHFFFAOYSA-N | C1C2C(COS(=O)O1)C3(C(=C(C2(C3(Cl)Cl)Cl)Cl)Cl)Cl | 284 | 0 | 2 | 2 |
| Nonachlor (C10H5Cl9) | OCHOKXCPKDPNQU-UHFFFAOYSA-N | C12C(C(C(C1Cl)Cl)Cl)C3(C(=C(C2(C3(Cl)Cl)Cl)Cl)Cl)Cl | 125 | 3495 | 2 | 1 |
| Oxychlordane (C10H4Cl8O) | VWGNQYSIWFHEQU-UHFFFAOYSA-N | C12C(C(C3(C1O3)Cl)Cl)C4(C(=C(C2(C4(Cl)Cl)Cl)Cl)Cl)Cl | 0 | 2085 | 2 | 1 |
| Endrin ketone (C12H8Cl6O) | IZHZFAQWVKBTSL-UHFFFAOYSA-N | C1C2C3C4C1C(=O)C2C5(C3(C(C4(C5Cl)Cl)(Cl)Cl)Cl)Cl | 70 | 860 | 2 | 1 |
| Endrin aldehyde (C12H8Cl6O) | HCTWZIFNBBCVGM-UHFFFAOYSA-N | C1C(C2C3C1C4C5(C2(C(C3(C45Cl)Cl)(Cl)Cl)Cl)Cl)C=O | 0 | 2569 | 2 | 1 |
| Photodieldrin (C12H8Cl6O) | LOVDWMTZZYUFMA-UHFFFAOYSA-N | C12C3C(C4C1C5(C(C3(C4(C5(Cl)Cl)Cl)Cl)Cl)Cl)C6C2O6 | 30 | 50 | 2 | 2 |
| Tris(1,3-dichioro-2-propyl) phosphate (C9H15Cl6O4P) | ASLWPAWFJZFCKF-UHFFFAOYSA-N | C(C(CCl)OP(=O)(OC(CCl)CCl)OC(CCl)CCl)Cl | 0 | 40 | 2 | 2 |
| Phosphoric acid, bis[2-chloro-1-(chloromethyl)ethyl] 2,3-dichloropropyl ester (C9H15Cl6O4P) | PLUXGFREPRMQHY-UHFFFAOYSA-N | C(C(CCl)Cl)OP(=O)(OC(CCl)CCl)OC(CCl)CCl | 0 | 901 | 2 | 1 |
| Tris(2,3-dichloropropyl) phosphate (C9H15Cl6O4P) | JZZBTMVTLBHJHL-UHFFFAOYSA-N | C(C(CCl)Cl)OP(=O)(OCC(CCl)Cl)OCC(CCl)Cl | 0 | 10 | 2 | 2 |
| Succinic acid, heptyl 2,2,2-trichloroethyl ester (C13H21Cl3O4) | NAQVIHHSOMSWFF-UHFFFAOYSA-N | CCCCCCCOC(=O)CCC(=O)OCC(Cl)(Cl)Cl | 0 | 0 | 2 | 2 |
| Malonic acid, 2,2-dichloroethyl nonyl ester (C14H24Cl2O4) | ZXSUKHKMKVFXIJ-UHFFFAOYSA-N | CCCCCCCCCOC(=O)CC(=O)OCC(Cl)Cl | 0 | 90 | 2 | 2 |
| Succinic acid, 2,2-dichloroethyl octyl ester (C14H24Cl2O4) | QFTBHEJIAVIDBU-UHFFFAOYSA-N | CCCCCCCCOC(=O)CCC(=O)OCC(Cl)Cl | 0 | 0 | 2 | 2 |
| Malonic acid, 10-chlorodecyl ethyl ester (C15H27ClO4) | CYTQLGHFERDHGV-UHFFFAOYSA-N | CCOC(=O)CC(=O)OCCCCCCCCCCCl | 0 | 0 | 2 | 2 |
| Succinic acid, 8-chloroctyl propyl ester (C15H27ClO4) | CTESHHZPTSHUSY-UHFFFAOYSA-N | CCCOC(=O)CCC(=O)OCCCCCCCCCl | 0 | 20 | 2 | 2 |
| Malonic acid, butyl 8-chlorooctyl ester (C15H27ClO4) | BPVJFODNWKIIMW-UHFFFAOYSA-N | CCCCOC(=O)CC(=O)OCCCCCCCCCl | 0 | 0 | 2 | 2 |
| Malonic acid, 8-chlorooctyl isobutyl ester (C15H27ClO4) | HSJUCFNMXKNWJC-UHFFFAOYSA-N | CC(C)COC(=O)CC(=O)OCCCCCCCCCl | 0 | 0 | 2 | 2 |
| D-Alanine, N-(5-chlorovaleryl)-, heptyl ester (C15H28ClNO3) | KOFVZRFXZTVRON-UHFFFAOYSA-N | CCCCCCCOC(=O)C(C)NC(=O)CCCCCl | 0 | 460 | 2 | 1 |
| Malonic acid, 2-chloropropyl nonyl ester (C15H27ClO4) | VVUNFCIBQWZLJJ-UHFFFAOYSA-N | CCCCCCCCCOC(=O)CC(=O)OCC(C)Cl | 0 | 0 | 2 | 2 |
| Succinic acid, 2-chloropropyl octyl ester (C15H27ClO4) | GZLBTHIHJKOQHC-UHFFFAOYSA-N | CCCCCCCCOC(=O)CCC(=O)OCC(C)Cl | 0 | 0 | 2 | 2 |
| Trichloroacetic acid, tridec-2-ynyl ester (C15H23Cl3O2) | IKJIABATPGGYLS-UHFFFAOYSA-N | CCCCCCCCCCC#CCOC(=O)C(Cl)(Cl)Cl | 0 | 0 | 2 | 2 |
| Dichloroacetamide, N,N-diheptyl- (C16H31Cl2NO) | DTFJMKGUYWLTPF-UHFFFAOYSA-N | CCCCCCCN(CCCCCCC)C(=O)C(Cl)Cl | 0 | 3603 | 2 | 1 |
| Tridecyl trichloroacetate (C15H27Cl3O2) | IVESCNMAOKRZNG-UHFFFAOYSA-N | CCCCCCCCCCCCCOC(=O)C(Cl)(Cl)Cl | 0 | 0 | 2 | 2 |
| Chloroacetamide, N-heptyl-N-octyl- (C17H34ClNO) | HFLONKJUBWGIHD-UHFFFAOYSA-N | CCCCCCCCN(CCCCCCC)C(=O)CCl | 50 | 6376 | 2 | 1 |
| Propanamide, N,N-diheptyl-3-chloro- (C17H34ClNO) | HMZMBEDMMYANJV-UHFFFAOYSA-N | CCCCCCCN(CCCCCCC)C(=O)CCCl | 70 | 4904 | 2 | 1 |
| Propanamide, N,N-diheptyl-2-chloro- (C17H34ClNO) | UYMKAUWTCKPFKN-UHFFFAOYSA-N | CCCCCCCN(CCCCCCC)C(=O)C(C)Cl | 300 | 9999 | 2 | 1 |
| Tetradecyl dichloroacetate (C16H30Cl2O2) | JFGNOMDHPRPBJP-UHFFFAOYSA-N | CCCCCCCCCCCCCCOC(=O)C(Cl)Cl | 0 | 0 | 2 | 2 |
| 5-chlorovaleric acid, dodec-9-ynyl ester (C17H29ClO2) | RUPFATUHMIZGOQ-UHFFFAOYSA-N | CCC#CCCCCCCCCOC(=O)CCCCCl | 0 | 0 | 2 | 2 |
| Stearic acid chloride (C18H35ClO) | WTBAHSZERDXKKZ-UHFFFAOYSA-N | CCCCCCCCCCCCCCCCCC(=O)Cl | 0 | 1559 | 2 | 1 |
| Propanoic acid, 3-chloro, tetradecyl ester (C17H33ClO2) | KUZUNHDCZIJWAO-UHFFFAOYSA-N | CCCCCCCCCCCCCCOC(=O)CCCl | 0 | 250 | 2 | 1 |
| 2-Chlorohexadecanoic acid, methyl ester (C17H33ClO2) | NYTGXUXRZXHXQK-UHFFFAOYSA-N | CCCCCCCCCCCCCCC(C(=O)OC)Cl | 210 | 440 | 2 | 1 |
| Fumaric acid, 8-chlorooctyl propyl ester (C15H25ClO4) | MGHZNNGUVBDAGD-MDZDMXLPSA-N | CCCOC(=O)/C=C/C(=O)OCCCCCCCCCl | 0 | 30 | 2 | 2 |
| Fumaric acid, 2-chloropropyl octyl ester (C15H25ClO4) | FJYBULSVGOZXRU-MDZDMXLPSA-N | CCCCCCCCOC(=O)/C=C/C(=O)OCC(C)Cl | 0 | 50 | 2 | 2 |
| Dichlorphenamide, tetramethyl deriv. (C10H14Cl2N2O4S2) | NJXHBEXVYIBIEK-UHFFFAOYSA-N | CN(C)S(=O)(=O)C1=CC(=C(C(=C1)Cl)Cl)S(=O)(=O)N(C)C | 1096 | 384 | 1 | 1 |
| Phosphorodiamidic acid, tetramethyl-, pentachlorophenyl ester (C10H12Cl5N2O2P) | RBBJXBKKZWSEHR-UHFFFAOYSA-N | CN(C)P(=O)(N(C)C)OC1=C(C(=C(C(=C1Cl)Cl)Cl)Cl)Cl | 0 | 99 | 2 | 2 |
| Carbophenothion sulfone (C11H16ClO4PS3) | CBXRYOZUHFXMRF-UHFFFAOYSA-N | CCOP(=S)(OCC)SCS(=O)(=O)C1=CC=C(C=C1)Cl | 40 | 0 | 2 | 2 |
| Carbophenothion O-analog sulfone (C11H16ClO5PS2) | AQORIFUYBMSEHZ-UHFFFAOYSA-N | CCOP(=O)(OCC)SCS(=O)(=O)C1=CC=C(C=C1)Cl | 0 | 0 | 2 | 2 |
| Chlornidine (C11H13Cl2N3O4) | XKUWFOYPQIVFMM-UHFFFAOYSA-N | CC1=CC(=C(C(=C1)[N+](=O)[O-])N(CCCl)CCCl)[N+](=O)[O-] | 176 | 0 | 2 | 2 |
| Chloramphenicol (C11H12Cl2N2O5) | WIIZWVCIJKGZOK-IUCAKERBSA-N | C1=CC(=CC=C1[C@@H]([C@H](CO)NC(=O)C(Cl)Cl)O)[N+](=O)[O-] | 0 | 0 | 2 | 2 |
| Succinic acid, ethyl 2,3,4,6-tetrachlorophenyl ester (C12H10Cl4O4) | JDEADFKPUSQYMO-UHFFFAOYSA-N | CCOC(=O)CCC(=O)OC1=C(C(=C(C=C1Cl)Cl)Cl)Cl | 0 | 0 | 2 | 2 |
| 2,4-D Butoxyethyl ester (C14H18Cl2O4) | ZMWGIGHRZQTQRE-UHFFFAOYSA-N | CCCCOCCOC(=O)COC1=C(C=C(C=C1)Cl)Cl | 1290 | 10 | 1 | 2 |
| Trichlamide (C13H16Cl3NO3) | NHTFLYKPEGXOAN-UHFFFAOYSA-N | CCCCOC(C(Cl)(Cl)Cl)NC(=O)C1=CC=CC=C1O | 510 | 10 | 2 | 2 |
| Succinic acid, propyl 2,3,5-trichlorophenyl ester (C13H13Cl3O4) | BCWDFFKPKYQWFC-UHFFFAOYSA-N | CCCOC(=O)CCC(=O)OC1=CC(=CC(=C1Cl)Cl)Cl | 0 | 0 | 2 | 2 |
| Succinic acid, propyl 2,3,6-trichlorophenyl ester (C13H13Cl3O4) | ULGNTESKJOCJGR-UHFFFAOYSA-N | CCCOC(=O)CCC(=O)OC1=C(C=CC(=C1Cl)Cl)Cl | 0 | 0 | 2 | 2 |
| Sarcosine, N-(2-chlorobenzoyl)-, pentyl ester (C15H20ClNO3) | KELZJYIYVCWDRO-UHFFFAOYSA-N | CCCCCOC(=O)CN(C)C(=O)C1=CC=CC=C1Cl | 1672 | 100 | 1 | 2 |
| Sarcosine, N-(4-chlorobenzoyl)-, pentyl ester (C15H20ClNO3) | SUFPIRCEMKSABB-UHFFFAOYSA-N | CCCCCOC(=O)CN(C)C(=O)C1=CC=C(C=C1)Cl | 571 | 0 | 2 | 2 |
| Succinic acid, butyl 3,5-dichlorophenyl ester (C14H16Cl2O4) | OBFINFKDECSTNA-UHFFFAOYSA-N | CCCCOC(=O)CCC(=O)OC1=CC(=CC(=C1)Cl)Cl | 0 | 0 | 2 | 2 |
| Succinic acid, 3,5-dichlorophenyl isobutyl ester (C14H16Cl2O4) | KJLLZEAVRZFQCY-UHFFFAOYSA-N | CC(C)COC(=O)CCC(=O)OC1=CC(=CC(=C1)Cl)Cl | 0 | 0 | 2 | 2 |
| L-Phenylalanine, N-(5-hlrovaleryl)-, methyl ester (C15H20ClNO3) | JMOWXKQIYNSZQF-UHFFFAOYSA-N | COC(=O)C(CC1=CC=CC=C1)NC(=O)CCCCCl | 40 | 50 | 2 | 2 |
| β-Alanine, N-(2-chlorobenzoyl)-, pentyl ester (C15H20ClNO3) | MMWDKJCCDUDHPZ-UHFFFAOYSA-N | CCCCCOC(=O)CCNC(=O)C1=CC=CC=C1Cl | 761 | 30 | 2 | 2 |
| β-Alanine, N-(4-chlorobenzoyl)-, pentyl ester (C15H20ClNO3) | AEPLOPPDIMQBKI-UHFFFAOYSA-N | CCCCCOC(=O)CCNC(=O)C1=CC=C(C=C1)Cl | 1161 | 0 | 1 | 2 |
| Decyl 4-chlorobenzoate (C17H25ClO2) | ASTUITNORCWJGQ-UHFFFAOYSA-N | CCCCCCCCCCOC(=O)C1=CC=C(C=C1)Cl | 0 | 0 | 2 | 2 |
| Benzoic acid, 2-chloro, decyl ester (C17H25ClO2) | XWNLBSXKHJVMLC-UHFFFAOYSA-N | CCCCCCCCCCOC(=O)C1=CC=CC=C1Cl | 80 | 0 | 2 | 2 |
| trans-Chlorfenvinphos (C12H14Cl3O4P) | FSAVDKDHPDSCTO-XYOKQWHBSA-N | CCOP(=O)(OCC)O/C(=C/Cl)/C1=C(C=C(C=C1)Cl)Cl | 30 | 5149 | 2 | 1 |
| chlorfenvinphos(cis) (C12H14Cl3O4P) | FSAVDKDHPDSCTO-WQLSENKSSA-N | CCOP(=O)(OCC)O/C(=C\Cl)/C1=C(C=C(C=C1)Cl)Cl | 60 | 5729 | 2 | 1 |
| Chlorfenvinphos (C12H14Cl3O4P) | FSAVDKDHPDSCTO-XYOKQWHBSA-N | CCOP(=O)(OCC)O/C(=C/Cl)/C1=C(C=C(C=C1)Cl)Cl | 42 | 5949 | 2 | 1 |
| Fumaric acid, ethyl 2,3,4,6-tetrachlorophenyl ester (C12H8Cl4O4) | VOYOQIXZKUBDBK-ONEGZZNKSA-N | CCOC(=O)/C=C/C(=O)OC1=C(C(=C(C=C1Cl)Cl)Cl)Cl | 80 | 0 | 2 | 2 |
| Fumaric acid, ethyl 2,3,5,6-tetrachlorophenyl ester (C12H8Cl4O4) | SCTMAYQQQLGGGL-ONEGZZNKSA-N | CCOC(=O)/C=C/C(=O)OC1=C(C(=CC(=C1Cl)Cl)Cl)Cl | 120 | 0 | 2 | 2 |
| Fumaric acid, propyl 2,4,5-trichlorophenyl ester (C13H11Cl3O4) | CFQPOUXSYAHDSJ-ONEGZZNKSA-N | CCCOC(=O)/C=C/C(=O)OC1=CC(=C(C=C1Cl)Cl)Cl | 60 | 0 | 2 | 2 |
| Fumaric acid, propyl 2,3,5-trichlorophenyl ester (C13H11Cl3O4) | PSHHEPLAPIWQBF-ONEGZZNKSA-N | CCCOC(=O)/C=C/C(=O)OC1=CC(=CC(=C1Cl)Cl)Cl | 60 | 0 | 2 | 2 |
| Fumaric acid, propyl 3,4,5-trichlorophenyl ester (C13H11Cl3O4) | QFIHIVQNZBYJBU-ONEGZZNKSA-N | CCCOC(=O)/C=C/C(=O)OC1=CC(=C(C(=C1)Cl)Cl)Cl | 60 | 0 | 2 | 2 |
| Fumaric acid, propyl 2,3,6-trichlorophenyl ester (C13H11Cl3O4) | HXALDMWGQWGCOL-AATRIKPKSA-N | CCCOC(=O)/C=C/C(=O)OC1=C(C=CC(=C1Cl)Cl)Cl | 150 | 0 | 2 | 2 |
| Fumaric acid, propyl 2,4,6-trichlorophenyl ester (C13H11Cl3O4) | QOWQPXIRHJLGBC-ONEGZZNKSA-N | CCCOC(=O)/C=C/C(=O)OC1=C(C=C(C=C1Cl)Cl)Cl | 40 | 0 | 2 | 2 |
| Fumaric acid, butyl 3,5-dichlorophenyl ester (C14H14Cl2O4) | OWQDNEANEOIMEX-SNAWJCMRSA-N | CCCCOC(=O)/C=C/C(=O)OC1=CC(=CC(=C1)Cl)Cl | 30 | 0 | 2 | 2 |
| Fumaric acid, 3,5-dichlorophenyl isobutyl ester (C14H14Cl2O4) | HLGHAOURNLIAIB-ONEGZZNKSA-N | CC(C)COC(=O)/C=C/C(=O)OC1=CC(=CC(=C1)Cl)Cl | 50 | 0 | 2 | 2 |
| Ethacrynic acid, methyl ester (C14H14Cl2O4) | XFNMYPXAINMYAC-UHFFFAOYSA-N | CCC(=C)C(=O)C1=C(C(=C(C=C1)OCC(=O)OC)Cl)Cl | 1480 | 1030 | 1 | 1 |
| Fumaric acid, butyl 2-chloro-5-methylphenyl ester (C15H17ClO4) | FNERJLZUNQETKU-BQYQJAHWSA-N | CCCCOC(=O)/C=C/C(=O)OC1=C(C=CC(=C1)C)Cl | 250 | 70 | 2 | 2 |
| Fumaric acid, 2-chloro-5-methylphenyl isobutyl ester (C15H17ClO4) | QMXUCWZBIUTUSC-VOTSOKGWSA-N | CC1=CC(=C(C=C1)Cl)OC(=O)/C=C/C(=O)OCC(C)C | 390 | 110 | 2 | 1 |
| 2-Chlorobenzoic acid, 2,7-dimethyloct-7-en-5-yn-4-yl ester (C17H19ClO2) | KNPYXCMBMIIPQA-UHFFFAOYSA-N | CC(C)CC(C#CC(=C)C)OC(=O)C1=CC=CC=C1Cl | 80 | 0 | 2 | 2 |
| 4-Chlorobenzoic acid, 2,7-dimethyloct-7-en-5-yn-4-yl ester (C17H19ClO2) | COCOCAKCCLBCEG-UHFFFAOYSA-N | CC(C)CC(C#CC(=C)C)OC(=O)C1=CC=C(C=C1)Cl | 0 | 0 | 2 | 2 |
| Chloroxuron (C15H15ClN2O2) | IVUXTESCPZUGJC-UHFFFAOYSA-N | CN(C)C(=O)NC1=CC=C(C=C1)OC2=CC=C(C=C2)Cl | 5975 | 0 | 1 | 2 |
| Trichlormethiazide (C8H8Cl3N3O4S2) | LMJSLTNSBFUCMU-UHFFFAOYSA-N | C1=C2C(=CC(=C1Cl)S(=O)(=O)N)S(=O)(=O)NC(N2)C(Cl)Cl | 229 | 0 | 2 | 2 |
| Benzamide, N-(2,5-dimethoxyphenyl)-2-chloro- (C15H14ClNO3) | RLHACPNMKPCNMS-UHFFFAOYSA-N | COC1=CC(=C(C=C1)OC)NC(=O)C2=CC=CC=C2Cl | 6716 | 110 | 1 | 1 |
| Benzamide, N-(2,5-dimethoxyphenyl)-4-chloro- (C15H14ClNO3) | HCVYUDNPZQUVRH-UHFFFAOYSA-N | COC1=CC(=C(C=C1)OC)NC(=O)C2=CC=C(C=C2)Cl | 5225 | 20 | 1 | 2 |
| 4-Nitrobenzoic acid, 3,4-dichlorophenyl ester (C13H7Cl2NO4) | LXEDUJYAENAZGC-UHFFFAOYSA-N | C1=CC(=CC=C1C(=O)OC2=CC(=C(C=C2)Cl)Cl)[N+](=O)[O-] | 480 | 0 | 2 | 2 |
| Bis(4-chlorothiophenyl)oxalate (C14H8Cl2O2S2) | NDEFWMFSVXHDCR-UHFFFAOYSA-N | C1=CC(=CC=C1SC(=O)C(=O)SC2=CC=C(C=C2)Cl)Cl | 196 | 0 | 2 | 2 |
| 1,2-Bis(2,4-dichlorophenoxy)ethane (C14H10Cl4O2) | UWGCTYZENHHSID-UHFFFAOYSA-N | C1=CC(=C(C=C1Cl)Cl)OCCOC2=C(C=C(C=C2)Cl)Cl | 5262 | 0 | 1 | 2 |
| Benzamide, 3,5-dichloro-N-(3,4-dichlorophenyl)-2-hydroxy- (C13H7Cl4NO2) | SJQBHPJLLIJASD-UHFFFAOYSA-N | C1=CC(=C(C=C1NC(=O)C2=CC(=CC(=C2O)Cl)Cl)Cl)Cl | 1829 | 30 | 1 | 2 |
| PCB 202 (C12H2Cl8) | JPOPEORRMSDUIP-UHFFFAOYSA-N | C1=C(C(=C(C(=C1Cl)Cl)C2=C(C(=CC(=C2Cl)Cl)Cl)Cl)Cl)Cl | 3286 | 87 | 1 | 2 |
| PCB 197 (C12H2Cl8) | YPDBBDKYNWRFMF-UHFFFAOYSA-N | C1=C(C(=C(C(=C1Cl)Cl)Cl)C2=C(C(=C(C=C2Cl)Cl)Cl)Cl)Cl | 3301 | 62 | 1 | 2 |
| PCB 194 (C12H2Cl8) | DTMRKGRREZAYAP-UHFFFAOYSA-N | C1=C(C(=C(C(=C1Cl)Cl)Cl)Cl)C2=CC(=C(C(=C2Cl)Cl)Cl)Cl | 3591 | 869 | 1 | 1 |
| PCB 201 (C12H2Cl8) | LJQOBQLZTUSEJA-UHFFFAOYSA-N | C1=C(C(=C(C(=C1Cl)Cl)Cl)C2=C(C(=CC(=C2Cl)Cl)Cl)Cl)Cl | 3249 | 125 | 1 | 1 |
| PCB 196 (C12H2Cl8) | BQFCCUSDZLKBJG-UHFFFAOYSA-N | C1=C(C(=C(C(=C1Cl)Cl)Cl)Cl)C2=C(C(=C(C=C2Cl)Cl)Cl)Cl | 3409 | 1065 | 1 | 1 |
| PCB 200 (C12H2Cl8) | HHXNVASVVVNNDG-UHFFFAOYSA-N | C1=CC(=C(C(=C1Cl)C2=C(C(=C(C(=C2Cl)Cl)Cl)Cl)Cl)Cl)Cl | 3215 | 85 | 1 | 2 |
| PCB 199 (C12H2Cl8) | HJBYDWKNARZTMJ-UHFFFAOYSA-N | C1=C(C(=C(C(=C1Cl)Cl)Cl)Cl)C2=C(C(=CC(=C2Cl)Cl)Cl)Cl | 3304 | 86 | 1 | 2 |
| PCB 195 (C12H2Cl8) | JAHJITLFJSDRCG-UHFFFAOYSA-N | C1=CC(=C(C(=C1C2=C(C(=C(C(=C2Cl)Cl)Cl)Cl)Cl)Cl)Cl)Cl | 3486 | 1186 | 1 | 1 |
| PCB 198 (C12H2Cl8) | PJHBSPRZHUOIAS-UHFFFAOYSA-N | C1=C(C=C(C(=C1Cl)Cl)C2=C(C(=C(C(=C2Cl)Cl)Cl)Cl)Cl)Cl | 3421 | 1108 | 1 | 1 |
| PCB 204 (C12H2Cl8) | JDZUWXRNKHXZFE-UHFFFAOYSA-N | C1=C(C=C(C(=C1Cl)C2=C(C(=C(C(=C2Cl)Cl)Cl)Cl)Cl)Cl)Cl | 3322 | 61 | 1 | 2 |
| chloropyramine (C16H20ClN3) | ICKFFNBDFNZJSX-UHFFFAOYSA-N | CN(C)CCN(CC1=CC=C(C=C1)Cl)C2=CC=CC=N2 | 70 | 0 | 2 | 2 |
| N,N'-Di(p-chlorobenzyl)urea (C15H14Cl2N2O) | UQCDPZIXWSDDRU-UHFFFAOYSA-N | C1=CC(=CC=C1CNC(=O)NCC2=CC=C(C=C2)Cl)Cl | 3700 | 0 | 1 | 2 |
| Dichloroacetic acid, 4-benzyloxyphenyl ester (C15H12Cl2O3) | BHDHMFXSIYFMCZ-UHFFFAOYSA-N | C1=CC=C(C=C1)COC2=CC=C(C=C2)OC(=O)C(Cl)Cl | 370 | 0 | 2 | 2 |
| Diclofenac, methyl ester (C15H13Cl2NO2) | VETACGBDFVVKGZ-UHFFFAOYSA-N | COC(=O)CC1=CC=CC=C1NC2=C(C=CC=C2Cl)Cl | 2332 | 0 | 1 | 2 |
| Meclofenamic acid, methyl ester (C15H13Cl2NO2) | IZZWHVDZJOINFQ-UHFFFAOYSA-N | CC1=C(C(=C(C=C1)Cl)NC2=CC=CC=C2C(=O)OC)Cl | 2270 | 140 | 1 | 1 |
| Carbinoxamine (C16H19ClN2O) | OJFSXZCBGQGRNV-UHFFFAOYSA-N | CN(C)CCOC(C1=CC=C(C=C1)Cl)C2=CC=CC=N2 | 0 | 0 | 2 | 2 |
| Bis(p-chlorobenzylidene)ethylene diamine (C16H14Cl2N2) | VMWYCJFPSDIEJB-UHFFFAOYSA-N | C1=CC(=CC=C1C=NCCN=CC2=CC=C(C=C2)Cl)Cl | 27 | 0 | 2 | 2 |
| Benzenemethanol, 2-chloro-α-(4-chlorophenyl)-α-(trichloromethyl)- (C14H9Cl5O) | LUXSISXJGNCOKN-UHFFFAOYSA-N | C1=CC=C(C(=C1)C(C2=CC=C(C=C2)Cl)(C(Cl)(Cl)Cl)O)Cl | 0 | 0 | 2 | 2 |
| Chlophedianol (C17H20ClNO) | WRCHFMBCVFFYEQ-UHFFFAOYSA-N | CN(C)CCC(C1=CC=CC=C1)(C2=CC=CC=C2Cl)O | 20 | 8317 | 2 | 1 |
| Prolan (C15H13Cl2NO2) | JCWVUDIGEJLVPS-UHFFFAOYSA-N | CC(C(C1=CC=C(C=C1)Cl)C2=CC=C(C=C2)Cl)[N+](=O)[O-] | 310 | 0 | 2 | 2 |
| Triadimenol (C14H18ClN3O2) | BAZVSMNPJJMILC-UHFFFAOYSA-N | CC(C)(C)C(C(N1C=NC=N1)OC2=CC=C(C=C2)Cl)O | 16 | 0 | 2 | 2 |
| Triadimefon (C14H16ClN3O2) | WURBVZBTWMNKQT-UHFFFAOYSA-N | CC(C)(C)C(=O)C(N1C=NC=N1)OC2=CC=C(C=C2)Cl | 172 | 59 | 2 | 2 |
| Chloroquine M (des-ethyl) (C16H22ClN3) | MCYUUUTUAAGOOT-UHFFFAOYSA-N | CCNCCCC(C)NC1=C2C=CC(=CC2=NC=C1)Cl | 427 | 0 | 2 | 2 |
| Benzamide, N-(3-chlorophenyl)-4-butyl- (C17H18ClNO) | HFAWWPRHBUTMKX-UHFFFAOYSA-N | CCCCC1=CC=C(C=C1)C(=O)NC2=CC(=CC=C2)Cl | 1081 | 0 | 1 | 2 |
| 4-Butylbenzoic acid, 4-chlorophenyl ester (C17H17ClO2) | KFSDSHJKHABQJV-UHFFFAOYSA-N | CCCCC1=CC=C(C=C1)C(=O)OC2=CC=C(C=C2)Cl | 30 | 0 | 2 | 2 |
| Hexaconazole (C14H17Cl2N3O) | STMIIPIFODONDC-UHFFFAOYSA-N | CCCCC(CN1C=NC=N1)(C2=C(C=C(C=C2)Cl)Cl)O | 10 | 130 | 2 | 1 |
| (2S,3S)-1-(4-Chlorophenyl)-4,4-dimethyl-2-(1,2,4-triazol-1-yl)pentan-3-ol (C15H20ClN3O) | RMOGWMIKYWRTKW-UHFFFAOYSA-N | CC(C)(C)C(C(CC1=CC=C(C=C1)Cl)N2C=NC=N2)O | 10 | 0 | 2 | 2 |
| Perthane (C18H20Cl2) | QFMDFTQOJHFVNR-UHFFFAOYSA-N | CCC1=CC=C(C=C1)C(C2=CC=C(C=C2)CC)C(Cl)Cl | 248 | 57 | 2 | 2 |
| Methoxychlor olefin (DDE-analog) (C16H14Cl2O2) | YCRYSVKEWAWTGI-UHFFFAOYSA-N | COC1=CC=C(C=C1)C(=C(Cl)Cl)C2=CC=C(C=C2)OC | 9999 | 971 | 1 | 1 |
| Uniconazole (C15H18ClN3O) | YNWVFADWVLCOPU-MDWZMJQESA-N | CC(C)(C)C(/C(=C\C1=CC=C(C=C1)Cl)/N2C=NC=N2)O | 20 | 0 | 2 | 2 |
| cis-Captafol (C10H9Cl4NO2S) | JHRWWRDRBPCWTF-OLQVQODUSA-N | C1C=CC[C@H]2[C@@H]1C(=O)N(C2=O)SC(C(Cl)Cl)(Cl)Cl | 40 | 90 | 2 | 2 |
| Dienochlor (C10Cl10) | LWLJUMBEZJHXHV-UHFFFAOYSA-N | C1(=C(C(C(=C1Cl)Cl)(C2(C(=C(C(=C2Cl)Cl)Cl)Cl)Cl)Cl)Cl)Cl | 117 | 0 | 2 | 2 |
| 1,2,3,7,8,9-Hexachlorodibenzo-p-dioxin (C12H2Cl6O2) | LGIRBUBHIWTVCK-UHFFFAOYSA-N | C1=C2C(=C(C(=C1Cl)Cl)Cl)OC3=C(C(=C(C=C3O2)Cl)Cl)Cl | 5180 | 340 | 1 | 1 |
| 1,2,3,4,7,8-Hexachlorodibenzodioxin (C12H2Cl6O2) | WCYYQNSQJHPVMG-UHFFFAOYSA-N | C1=C2C(=CC(=C1Cl)Cl)OC3=C(O2)C(=C(C(=C3Cl)Cl)Cl)Cl | 4974 | 661 | 1 | 1 |
| 1,2,3,6,7,8-Hexachlorodibenzo-p-dioxin (C12H2Cl6O2) | YCLUIPQDHHPDJJ-UHFFFAOYSA-N | C1=C2C(=C(C(=C1Cl)Cl)Cl)OC3=CC(=C(C(=C3O2)Cl)Cl)Cl | 5180 | 650 | 1 | 1 |
| 1,2,3,4,6,7,8-Heptachlorodibenzofuran (C12HCl7O) | WDMKCPIVJOGHBF-UHFFFAOYSA-N | C1=C2C3=C(C(=C(C(=C3Cl)Cl)Cl)Cl)OC2=C(C(=C1Cl)Cl)Cl | 4558 | 25 | 1 | 2 |
| Benzamide, N-(1-naphthyl)-2-chloro- (C17H12ClNO) | VSSOWOYYGCMNBI-UHFFFAOYSA-N | C1=CC=C2C(=C1)C=CC=C2NC(=O)C3=CC=CC=C3Cl | 0 | 310 | 2 | 1 |
| Benzamide, N-(1-naphthyl)-4-chloro- (C17H12ClNO) | IQWRPJFSMOZFIL-UHFFFAOYSA-N | C1=CC=C2C(=C1)C=CC=C2NC(=O)C3=CC=C(C=C3)Cl | 4154 | 0 | 1 | 2 |
| 1-Naphthalenecarboxamide, N-(3-chlorophenyl)- (C17H12ClNO) | MROMMCULOKJDLK-UHFFFAOYSA-N | C1=CC=C2C(=C1)C=CC=C2C(=O)NC3=CC(=CC=C3)Cl | 1702 | 0 | 1 | 2 |
| 4-Chlorobenzoic acid, 2-naphthyl ester (C17H11ClO2) | HFTVSEPAGVIKQA-UHFFFAOYSA-N | C1=CC=C2C=C(C=CC2=C1)OC(=O)C3=CC=C(C=C3)Cl | 2122 | 0 | 1 | 2 |
| 2-Chlorobenzoic acid, 2-naphthyl ester (C17H11ClO2) | SZXKYXASVKDBMN-UHFFFAOYSA-N | C1=CC=C2C=C(C=CC2=C1)OC(=O)C3=CC=CC=C3Cl | 1902 | 0 | 1 | 2 |
| 1-Naphthoic acid, 4-chlorophenyl ester (C17H11ClO2) | ZTNAIEDTVGAUOA-UHFFFAOYSA-N | C1=CC=C2C(=C1)C=CC=C2C(=O)OC3=CC=C(C=C3)Cl | 200 | 0 | 2 | 2 |
| Norclobazam (C15H11ClN2O2) | RRTVVRIFVKKTJK-UHFFFAOYSA-N | C1C(=O)NC2=C(C=C(C=C2)Cl)N(C1=O)C3=CC=CC=C3 | 9999 | 0 | 1 | 2 |
| 7-Aminoclonazepam (C15H12ClN3O) | HEFRPWRJTGLSSV-UHFFFAOYSA-N | C1C(=O)NC2=C(C=C(C=C2)N)C(=N1)C3=CC=CC=C3Cl | 9999 | 2092 | 1 | 1 |
| Oxazepam (C15H12ClN2O2) | ADIMAYPTOBDMTL-UHFFFAOYSA-N | C1=CC=C(C=C1)C2=NC(C(=O)NC3=C2C=C(C=C3)Cl)O | 0 | 0 | 2 | 2 |
| Diazepam (C16H13ClN2O) | AAOVKJBEBIDNHE-UHFFFAOYSA-N | CN1C(=O)CN=C(C2=C1C=CC(=C2)Cl)C3=CC=CC=C3 | 7222 | 643 | 1 | 1 |
| Delorazepam (C15H10Cl2N2O) | CHIFCDOIPRCHCF-UHFFFAOYSA-N | C1C(=O)NC2=C(C=C(C=C2)Cl)C(=N1)C3=CC=CC=C3Cl | 9588 | 9848 | 1 | 1 |
| Adenosine, 2-chloro- (C10H12ClN5O4) | BIXYYZIIJIXVFW-UHFFFAOYSA-N | C1=NC2=C(N1C3C(C(C(O3)CO)O)O)N=C(N=C2N)Cl | 588 | 96 | 2 | 2 |
| Norchlorcyclizine (C17H19ClN2) | UZKBSZSTDQSMDR-UHFFFAOYSA-N | C1CN(CCN1)C(C2=CC=CC=C2)C3=CC=C(C=C3)Cl | 514 | 0 | 2 | 2 |
| 2-[(Cyclopropylmethyl)amino]-5-chlorobenzophenone (C17H16ClNO) | WCRKZICZCPHVAB-UHFFFAOYSA-N | C1CC1CNC2=C(C=C(C=C2)Cl)C(=O)C3=CC=CC=C3 | 9999 | 0 | 1 | 2 |
| Cyclopropanecarbonitrile, 1-(p-chlorophenyl)-2-(p-methoxyphenyl)- (C17H14ClNO) | UKBFLNJUMCRCGX-UHFFFAOYSA-N | COC1=CC=C(C=C1)C2CC2(C#N)C3=CC=C(C=C3)Cl | 9999 | 1802 | 1 | 1 |
| Sertraline (C17H17Cl2N) | VGKDLMBJGBXTGI-UHFFFAOYSA-N | CNC1CCC(C2=CC=CC=C12)C3=CC(=C(C=C3)Cl)Cl | 370 | 30 | 2 | 2 |
| 5-chlorovaleric acid, 2-(1-adamantyl)ethyl ester (C17H27ClO2) | VZIRUWPGJKHWAM-UHFFFAOYSA-N | C1C2CC3CC1CC(C2)(C3)CCOC(=O)CCCCCl | 0 | 0 | 2 | 2 |
| Tetrazepam (C16H17ClN2O) | IQWYAQCHYZHJOS-UHFFFAOYSA-N | CN1C(=O)CN=C(C2=C1C=CC(=C2)Cl)C3=CCCCC3 | 4034 | 9999 | 1 | 1 |
| Endosulfan sulfate (C9H6Cl6O4S) | AAPVQEMYVNZIOO-UHFFFAOYSA-N | C1C2C(COS(=O)(=O)O1)C3(C(=C(C2(C3(Cl)Cl)Cl)Cl)Cl)Cl | 1833 | 5992 | 1 | 1 |
| 1,4:5,8-Dimethanonaphthalen-2-ol, 3,5,6,7,8,9,9-heptachloro-1,2,3,4,4a,5,8,8a-octahydro-, (1α,2α,3β,4α,4aβ,5α,8α,8β)- (C12H9Cl7O) | KAKBPXOZYDVIGI-UHFFFAOYSA-N | C1C2C3C(C1C(C2O)Cl)C4(C(=C(C3(C4(Cl)Cl)Cl)Cl)Cl)Cl | 0 | 130 | 2 | 1 |
| Aldrin (C12H8Cl6) | QBYJBZPUGVGKQQ-SJJAEHHWSA-N | C1[C@@H]2C=C[C@H]1[C@H]3[C@@H]2[C@]4(C(=C([C@@]3(C4(Cl)Cl)Cl)Cl)Cl)Cl | 186 | 770 | 2 | 1 |
| Mazindol (C16H13ClN2O) | ZPXSCAKFGYXMGA-UHFFFAOYSA-N | C1CN2C(=N1)C3=CC=CC=C3C2(C4=CC=C(C=C4)Cl)O | 0 | 0 | 2 | 2 |
| 1-Adamantanecarboxamide, N-(3-chlorophenyl)- (C17H20ClNO) | OWEPCGATVXVIDJ-UHFFFAOYSA-N | C1C2CC3CC1CC(C2)(C3)C(=O)NC4=CC(=CC=C4)Cl | 1401 | 0 | 1 | 2 |
| 1-Adamantanecarboxylic acid, 4-chlorophenyl ester (C17H19ClO2) | BRFGISNTQUMEQO-UHFFFAOYSA-N | C1C2CC3CC1CC(C2)(C3)C(=O)OC4=CC=C(C=C4)Cl | 50 | 0 | 2 | 2 |
| 2-chlorobenzoic acid, 2-adamantyl ester (C17H19ClO2) | HYDIOIONCLEIID-UHFFFAOYSA-N | C1C2CC3CC1CC(C2)C3OC(=O)C4=CC=CC=C4Cl | 861 | 290 | 1 | 1 |
| Succinic acid, octyl 2,2,2-trichloroethyl ester (C14H23Cl3O4) | WZMILCOCAUGKFO-UHFFFAOYSA-N | CCCCCCCCOC(=O)CCC(=O)OCC(Cl)(Cl)Cl | 0 | 0 | 2 | 2 |
| Malonic acid, decyl 2,2-dichloroethyl ester (C15H26Cl2O4) | UAJXIOGUFMDYJU-UHFFFAOYSA-N | CCCCCCCCCCOC(=O)CC(=O)OCC(Cl)Cl | 0 | 70 | 2 | 2 |
| Succinic acid, 2,2-dichloroethyl nonyl ester (C15H26Cl2O4) | TWWRRVZQBHPRIT-UHFFFAOYSA-N | CCCCCCCCCOC(=O)CCC(=O)OCC(Cl)Cl | 0 | 0 | 2 | 2 |
| Succinic acid, 10-chlorodecyl ethyl ester (C16H29ClO4) | KPHAWSOOLTVFOK-UHFFFAOYSA-N | CCOC(=O)CCC(=O)OCCCCCCCCCCCl | 0 | 40 | 2 | 2 |
| Adipic acid, 8-chloroctyl ethyl ester (C16H29ClO4) | GGJPRBXSOZNTSB-UHFFFAOYSA-N | CCOC(=O)CCCCC(=O)OCCCCCCCCCl | 0 | 30 | 2 | 2 |
| Malonic acid, 10-chlorodecyl propyl ester (C16H29ClO4) | COEZQPUULVJOAL-UHFFFAOYSA-N | CCCOC(=O)CC(=O)OCCCCCCCCCCCl | 0 | 0 | 2 | 2 |
| Malonic acid, 8-chlorooctyl pentyl ester (C16H29ClO4) | GHRBRCOZQDGBSO-UHFFFAOYSA-N | CCCCCOC(=O)CC(=O)OCCCCCCCCCl | 0 | 0 | 2 | 2 |
| Succinic acid, butyl 8-chloroctyl ester (C16H29ClO4) | RAEZGNSFQZLPTE-UHFFFAOYSA-N | CCCCOC(=O)CCC(=O)OCCCCCCCCCl | 0 | 30 | 2 | 2 |
| Succinic acid, 8-chloroctyl isobutyl ester (C16H29ClO4) | QRTDRDMVYRYFBY-UHFFFAOYSA-N | CC(C)COC(=O)CCC(=O)OCCCCCCCCCl | 0 | 30 | 2 | 2 |
| D-Alanine, N-(5-chlorovaleryl)-, octyl ester (C16H30ClNO3) | LIOUYMBQEQGVFT-UHFFFAOYSA-N | CCCCCCCCOC(=O)C(C)NC(=O)CCCCCl | 0 | 540 | 2 | 1 |
| Malonic acid, 2-chloropropyl decyl ester (C16H29ClO4) | CYWXSQFLWQPHHS-UHFFFAOYSA-N | CCCCCCCCCCOC(=O)CC(=O)OCC(C)Cl | 0 | 0 | 2 | 2 |
| Succinic acid, 2-chloropropyl nonyl ester (C16H29ClO4) | XKLLPQPPLOBXPN-UHFFFAOYSA-N | CCCCCCCCCOC(=O)CCC(=O)OCC(C)Cl | 0 | 0 | 2 | 2 |
| Trichloroacetamide, N,N-diheptyl- (C16H30Cl3NO) | BVFXHEMDRLSTLE-UHFFFAOYSA-N | CCCCCCCN(CCCCCCC)C(=O)C(Cl)(Cl)Cl | 0 | 1672 | 2 | 1 |
| Dichloroacetamide, N-heptyl-N-octyl- (C17H33Cl2NO) | SOTPHTCBQMPAOA-UHFFFAOYSA-N | CCCCCCCCN(CCCCCCC)C(=O)C(Cl)Cl | 0 | 3353 | 2 | 1 |
| Tetradecyl trichloroacetate (C16H29Cl3O2) | CAUJGQDJKZXWDE-UHFFFAOYSA-N | CCCCCCCCCCCCCCOC(=O)C(Cl)(Cl)Cl | 0 | 0 | 2 | 2 |
| 1-chloroeicosane (C20H41Cl) | AFGNVSCTEXUEJE-UHFFFAOYSA-N | CCCCCCCCCCCCCCCCCCCCCl | 539 | 30 | 2 | 2 |
| Hexadecyl chloroacetate (C18H35ClO2) | XQHTWCIWGQDGOI-UHFFFAOYSA-N | CCCCCCCCCCCCCCCCOC(=O)CCl | 0 | 0 | 2 | 2 |
| 2-chloroethyl hexadecanoate (C18H35ClO2) | CPFFARIYTPCNJA-UHFFFAOYSA-N | CCCCCCCCCCCCCCCC(=O)OCCCl | 750 | 0 | 2 | 2 |
| 5-Chlorovaleric acid, tridecyl ester (C18H35ClO2) | QUAVOGDQRCBTGN-UHFFFAOYSA-N | CCCCCCCCCCCCCOC(=O)CCCCCl | 0 | 320 | 2 | 1 |
| 2-Chloroheptadecanoic acid, methyl ester (C18H35ClO2) | ZZHLIYKVBVITFY-UHFFFAOYSA-N | CCCCCCCCCCCCCCCC(C(=O)OC)Cl | 2672 | 1551 | 1 | 1 |
| 2- Chloropropionic acid, pentadecyl ester (C18H35ClO2) | YKUVAHVSNGLRHB-UHFFFAOYSA-N | CCCCCCCCCCCCCCCOC(=O)C(C)Cl | 0 | 0 | 2 | 2 |
| 5-Chlorovaleric acid, 2-tridecyl ester (C18H35ClO2) | CMCIREZBJQDCSX-UHFFFAOYSA-N | CCCCCCCCCCCC(C)OC(=O)CCCCCl | 0 | 0 | 2 | 2 |
| 5-Chlorovaleric acid, 3-tridecyl ester (C18H35ClO2) | YGWIZQGUCQDCRD-UHFFFAOYSA-N | CCCCCCCCCCC(CC)OC(=O)CCCCCl | 0 | 0 | 2 | 2 |
| 5-Chlorovaleric acid, 4-tridecyl ester (C18H35ClO2) | GXICQKKBZBKOSP-UHFFFAOYSA-N | CCCCCCCCCC(CCC)OC(=O)CCCCCl | 0 | 0 | 2 | 2 |
| Chloroacetamide, N,N-dioctyl- (C18H36ClNO) | ASYPQLBYSSYEBE-UHFFFAOYSA-N | CCCCCCCCN(CCCCCCCC)C(=O)CCl | 50 | 5765 | 2 | 1 |
| Propanamide, N-heptyl-N-octyl-3-chloro- (C18H36ClNO) | BGTYIRFGMJZZQI-UHFFFAOYSA-N | CCCCCCCCN(CCCCCCC)C(=O)CCCl | 140 | 9999 | 2 | 1 |
| Hexanamide, N,N-dihexyl-6-chloro- (C18H36ClNO) | UNTCHXLMOYTQJB-UHFFFAOYSA-N | CCCCCCN(CCCCCC)C(=O)CCCCCCl | 150 | 1271 | 2 | 1 |
| 5-Chlorovaleric acid, 5-tridecyl ester (C18H35ClO2) | MEVZAAUZXNKQIB-UHFFFAOYSA-N | CCCCCCCCC(CCCC)OC(=O)CCCCCl | 0 | 0 | 2 | 2 |
| Propanamide, N-heptyl-N-octyl-2-chloro- (C18H36ClNO) | QBZXHNDGBKWEQP-UHFFFAOYSA-N | CCCCCCCCN(CCCCCCC)C(=O)C(C)Cl | 230 | 9999 | 2 | 1 |
| Chloroacetamide, N,N-bis(2-ethylhexyl)- (C18H36ClNO) | GTLDINRZFOGPQE-UHFFFAOYSA-N | CCCCC(CC)CN(CC(CC)CCCC)C(=O)CCl | 0 | 781 | 2 | 1 |
| 5-chlorovaleric acid, tridec-2-ynyl ester (C18H31ClO2) | IWBWGAOOVXOGLS-UHFFFAOYSA-N | CCCCCCCCCCC#CCOC(=O)CCCCCl | 0 | 0 | 2 | 2 |
| Fumaric acid, octyl 2,2,2-trichloroethyl ester (C14H21Cl3O4) | ZKKSUBLUEFJBSP-CMDGGOBGSA-N | CCCCCCCCOC(=O)/C=C/C(=O)OCC(Cl)(Cl)Cl | 0 | 1241 | 2 | 1 |
| Fumaric acid, 2,2-dichloroethyl nonyl ester (C15H24Cl2O4) | NPEAWWXVPHOFTF-MDZDMXLPSA-N | CCCCCCCCCOC(=O)/C=C/C(=O)OCC(Cl)Cl | 0 | 240 | 2 | 1 |
| Fumaric acid, 10-chlorodecyl ethyl ester (C16H27ClO4) | YWJKCIKKRUGLIM-VAWYXSNFSA-N | CCOC(=O)/C=C/C(=O)OCCCCCCCCCCCl | 0 | 20 | 2 | 2 |
| Fumaric acid, butyl 8-chlorooctyl ester (C16H27ClO4) | PARYJXJJOSFHMQ-ZHACJKMWSA-N | CCCCOC(=O)/C=C/C(=O)OCCCCCCCCCl | 0 | 30 | 2 | 2 |
| Fumaric acid, 8-chlorooctyl isobutyl ester (C16H27ClO4) | MRHRYQMLUYULBY-MDZDMXLPSA-N | CC(C)COC(=O)/C=C/C(=O)OCCCCCCCCCl | 0 | 0 | 2 | 2 |
| Fumaric acid, 2-chloropropyl nonyl ester (C16H27ClO4) | CPJZMLBWUCJYLQ-ZHACJKMWSA-N | CCCCCCCCCOC(=O)/C=C/C(=O)OCC(C)Cl | 0 | 60 | 2 | 2 |
| 2,4,5-T Butoxyethyl ester (C14H17Cl3O4) | GLDWASBMYWLQGG-UHFFFAOYSA-N | CCCCOCCOC(=O)COC1=CC(=C(C=C1Cl)Cl)Cl | 1763 | 0 | 1 | 2 |
| Succinic acid, butyl 2,3,5-trichlorophenyl ester (C14H15Cl3O4) | IFEDMJNUTFOCNH-UHFFFAOYSA-N | CCCCOC(=O)CCC(=O)OC1=CC(=CC(=C1Cl)Cl)Cl | 0 | 0 | 2 | 2 |
| Succinic acid, butyl 2,3,6-trichlorophenyl ester (C14H15Cl3O4) | YKMTZAHFYZTRHM-UHFFFAOYSA-N | CCCCOC(=O)CCC(=O)OC1=C(C=CC(=C1Cl)Cl)Cl | 0 | 0 | 2 | 2 |
| Succinic acid, isobutyl 2,3,5-trichlorophenyl ester (C14H15Cl3O4) | VSZGGQBPIKEDPE-UHFFFAOYSA-N | CC(C)COC(=O)CCC(=O)OC1=CC(=CC(=C1Cl)Cl)Cl | 0 | 0 | 2 | 2 |
| Succinic acid, isobutyl 2,3,6-trichlorophenyl ester (C14H15Cl3O4) | DLAVXKZVZGRPFU-UHFFFAOYSA-N | CC(C)COC(=O)CCC(=O)OC1=C(C=CC(=C1Cl)Cl)Cl | 0 | 0 | 2 | 2 |
| Sarcosine, N-(2-chlorobenzoyl)-, hexyl ester (C16H22ClNO3) | CCRNYVLCQLBOBV-UHFFFAOYSA-N | CCCCCCOC(=O)CN(C)C(=O)C1=CC=CC=C1Cl | 1561 | 110 | 1 | 1 |
| Sarcosine, N-(4-chlorobenzoyl)-, hexyl ester (C16H22ClNO3) | WGPRKIYPKZLFAA-UHFFFAOYSA-N | CCCCCCOC(=O)CN(C)C(=O)C1=CC=C(C=C1)Cl | 1161 | 0 | 1 | 2 |
| Sarcosine, N-(4-chlorobenzoyl)-, isohexyl ester (C16H22ClNO3) | QKNRAHQLPYVFFY-UHFFFAOYSA-N | CC(C)CCCOC(=O)CN(C)C(=O)C1=CC=C(C=C1)Cl | 1141 | 0 | 1 | 2 |
| Diethatyl-ethyl (C16H22ClNO3) | WFKSADNZWSKCRZ-UHFFFAOYSA-N | CCC1=C(C(=CC=C1)CC)N(CC(=O)OCC)C(=O)CCl | 3267 | 0 | 1 | 2 |
| Aramite (C15H23ClO4S) | YKFRAOGHWKADFJ-UHFFFAOYSA-N | CC(COC1=CC=C(C=C1)C(C)(C)C)OS(=O)OCCCl | 450 | 0 | 2 | 2 |
| Succinic acid, 3,5-dichlorophenyl pentyl ester (C15H18Cl2O4) | TXRLZUNUCPQBIJ-UHFFFAOYSA-N | CCCCCOC(=O)CCC(=O)OC1=CC(=CC(=C1)Cl)Cl | 0 | 0 | 2 | 2 |
| Propanamide, N-(1-cyano-1,2-dimethylpropyl)-2-(2,4-dichlorophenoxy)- (C15H18Cl2N2O2) | IUOKJNROJISWRO-UHFFFAOYSA-N | CC(C)C(C)(C#N)NC(=O)C(C)OC1=C(C=C(C=C1)Cl)Cl | 1371 | 5695 | 1 | 1 |
| β-Alanine, N-(2-chlorobenzoyl)-,hexyl ester (C16H22ClNO3) | UNIHSLUFSWZHBU-UHFFFAOYSA-N | CCCCCCOC(=O)CCNC(=O)C1=CC=CC=C1Cl | 671 | 40 | 2 | 2 |
| β-Alanine, N-(4-chlorobenzoyl)-, hexyl ester (C16H22ClNO3) | DOVGEOHEEVJQGX-UHFFFAOYSA-N | CCCCCCOC(=O)CCNC(=O)C1=CC=C(C=C1)Cl | 961 | 0 | 1 | 2 |
| Pretilachlor (C17H26ClNO2) | YLPGTOIOYRQOHV-UHFFFAOYSA-N | CCCOCCN(C1=C(C=CC=C1CC)CC)C(=O)CCl | 360 | 10 | 2 | 2 |
| Butachlor (C17H26ClNO2) | HKPHPIREJKHECO-UHFFFAOYSA-N | CCCCOCN(C1=C(C=CC=C1CC)CC)C(=O)CCl | 809 | 883 | 1 | 1 |
| Benzamide, N-decyl-N-methyl-4-chloro- (C18H28ClNO) | ORXPNBVUTWVQKS-UHFFFAOYSA-N | CCCCCCCCCCN(C)C(=O)C1=CC=C(C=C1)Cl | 661 | 0 | 2 | 2 |
| Undecyl 4-chlorobenzoate (C18H27ClO2) | MEWVVEWZGJOCOB-UHFFFAOYSA-N | CCCCCCCCCCCOC(=O)C1=CC=C(C=C1)Cl | 0 | 0 | 2 | 2 |
| Benzoic acid, 2-chloro, undecyl ester (C18H27ClO2) | LZZUEDTUAJZZBG-UHFFFAOYSA-N | CCCCCCCCCCCOC(=O)C1=CC=CC=C1Cl | 30 | 0 | 2 | 2 |
| Fumaric acid, ethyl pentachlorophenyl ester (C12H7Cl5O4) | MSJGEMUIWSXWBP-ONEGZZNKSA-N | CCOC(=O)/C=C/C(=O)OC1=C(C(=C(C(=C1Cl)Cl)Cl)Cl)Cl | 60 | 0 | 2 | 2 |
| Fumaric acid, propyl 2,3,4,6-tetrachlorophenyl ester (C13H10Cl4O4) | YZQJZHDNIAAPNN-ONEGZZNKSA-N | CCCOC(=O)/C=C/C(=O)OC1=C(C(=C(C=C1Cl)Cl)Cl)Cl | 60 | 0 | 2 | 2 |
| Fumaric acid, propyl 2,3,4,5-tetrachlorophenyl ester (C13H10Cl4O4) | AEDTXMHHILJJGC-ONEGZZNKSA-N | CCCOC(=O)/C=C/C(=O)OC1=CC(=C(C(=C1Cl)Cl)Cl)Cl | 20 | 0 | 2 | 2 |
| Fumaric acid, propyl 2,3,5,6-tetrachlorophenyl ester (C13H10Cl4O4) | VIWFZOSRCAUXGT-ONEGZZNKSA-N | CCCOC(=O)/C=C/C(=O)OC1=C(C(=CC(=C1Cl)Cl)Cl)Cl | 90 | 0 | 2 | 2 |
| Fumaric acid, butyl 2,4,5-trichlorophenyl ester (C14H13Cl3O4) | JCQGTZRZRUGTAR-SNAWJCMRSA-N | CCCCOC(=O)/C=C/C(=O)OC1=CC(=C(C=C1Cl)Cl)Cl | 40 | 0 | 2 | 2 |
| Fumaric acid, butyl 2,3,5-trichlorophenyl ester (C14H13Cl3O4) | DGZMRDPDZYPNKU-SNAWJCMRSA-N | CCCCOC(=O)/C=C/C(=O)OC1=CC(=CC(=C1Cl)Cl)Cl | 40 | 0 | 2 | 2 |
| Fumaric acid, butyl 3,4,5-trichlorophenyl ester (C14H13Cl3O4) | DMECDNPDAOOMEY-SNAWJCMRSA-N | CCCCOC(=O)/C=C/C(=O)OC1=CC(=C(C(=C1)Cl)Cl)Cl | 50 | 0 | 2 | 2 |
| Fumaric acid, butyl 2,3,6-trichlorophenyl ester (C14H13Cl3O4) | QWMHMVNSYUNLFG-VOTSOKGWSA-N | CCCCOC(=O)/C=C/C(=O)OC1=C(C=CC(=C1Cl)Cl)Cl | 90 | 0 | 2 | 2 |
| Fumaric acid, butyl 2,4,6-trichlorophenyl ester (C14H13Cl3O4) | FDIOBAMAUYBXQM-SNAWJCMRSA-N | CCCCOC(=O)/C=C/C(=O)OC1=C(C=C(C=C1Cl)Cl)Cl | 40 | 0 | 2 | 2 |
| Fumaric acid, isobutyl 2,4,5-trichlorophenyl ester (C14H13Cl3O4) | MDKWGDBAERFMIR-ONEGZZNKSA-N | CC(C)COC(=O)/C=C/C(=O)OC1=CC(=C(C=C1Cl)Cl)Cl | 60 | 0 | 2 | 2 |
| Fumaric acid, isobutyl 2,3,5-trichlorophenyl ester (C14H13Cl3O4) | LEPGKNXBRAIHJE-ONEGZZNKSA-N | CC(C)COC(=O)/C=C/C(=O)OC1=CC(=CC(=C1Cl)Cl)Cl | 70 | 0 | 2 | 2 |
| Fumaric acid, isobutyl 3,4,5-trichlorophenyl ester (C14H13Cl3O4) | ZYNNZTCANZASBH-ONEGZZNKSA-N | CC(C)COC(=O)/C=C/C(=O)OC1=CC(=C(C(=C1)Cl)Cl)Cl | 60 | 0 | 2 | 2 |
| Fumaric acid, isobutyl 2,3,6-trichlorophenyl ester (C14H13Cl3O4) | JLSCRORRFNICEV-AATRIKPKSA-N | CC(C)COC(=O)/C=C/C(=O)OC1=C(C=CC(=C1Cl)Cl)Cl | 140 | 0 | 2 | 2 |
| Fumaric acid, isobutyl 2,4,6-trichlorophenyl ester (C14H13Cl3O4) | JJOFXKGOYZAVLR-ONEGZZNKSA-N | CC(C)COC(=O)/C=C/C(=O)OC1=C(C=C(C=C1Cl)Cl)Cl | 50 | 0 | 2 | 2 |
| Fumaric acid, 3,5-dichlorophenyl pentyl ester (C15H16Cl2O4) | RXVXYTPKGNCKMZ-AATRIKPKSA-N | CCCCCOC(=O)/C=C/C(=O)OC1=CC(=CC(=C1)Cl)Cl | 20 | 0 | 2 | 2 |
| Fumaric acid, 2-chloro-5-methylphenyl pentyl ester (C16H19ClO4) | LRSWQFVWJUANNJ-CMDGGOBGSA-N | CCCCCOC(=O)/C=C/C(=O)OC1=C(C=CC(=C1)C)Cl | 230 | 90 | 2 | 2 |
| 2-Chlorobenzoic acid, 2,6-dimethylnon-1-en-3-yn-5-yl ester (C18H21ClO2) | ZONSAPGHXXNOMW-UHFFFAOYSA-N | CCCC(C)C(C#CC(=C)C)OC(=O)C1=CC=CC=C1Cl | 0 | 0 | 2 | 2 |
| 4-Chlorobenzoic acid, 2,6-dimethylnon-1-en-3-yn-5-yl ester (C18H21ClO2) | VSKMPLKDSXMUOU-UHFFFAOYSA-N | CCCC(C)C(C#CC(=C)C)OC(=O)C1=CC=C(C=C1)Cl | 30 | 0 | 2 | 2 |
| 2-Chlorobenzoic acid, undec-2-enyl ester (C18H25ClO2) | UYDLFEDYUOXNMO-FMIVXFBMSA-N | CCCCCCCC/C=C/COC(=O)C1=CC=CC=C1Cl | 30 | 0 | 2 | 2 |
| 4-Chlorobenzoic acid, undec-2-enyl ester (C18H25ClO2) | OECIAGVCGLHDIB-MDZDMXLPSA-N | CCCCCCCC/C=C/COC(=O)C1=CC=C(C=C1)Cl | 0 | 0 | 2 | 2 |
| Hydrochlorothiazide, tetramethyl derivative (C11H16ClN3O4S2) | QPVCMENCMBSJLZ-UHFFFAOYSA-N | CN1CN(S(=O)(=O)C2=CC(=C(C=C21)Cl)S(=O)(=O)N(C)C)C | 4810 | 0 | 1 | 2 |
| Bis(4-Chlorophenylsulfonyl)methane (C13H10Cl2O4S2) | UQLKRAFFXYZVBU-UHFFFAOYSA-N | C1=CC(=CC=C1S(=O)(=O)CS(=O)(=O)C2=CC=C(C=C2)Cl)Cl | 166 | 0 | 2 | 2 |
| Bis(3-chlorophenylsulphonyl)methane (C13H10Cl2O4S2) | OWITURUKFUPLIO-UHFFFAOYSA-N | C1=CC(=CC(=C1)Cl)S(=O)(=O)CS(=O)(=O)C2=CC(=CC=C2)Cl | 410 | 0 | 2 | 2 |
| PCB 206 (C12HCl9) | JFIMDKGRGPNPRQ-UHFFFAOYSA-N | C1=C(C(=C(C(=C1Cl)Cl)Cl)Cl)C2=C(C(=C(C(=C2Cl)Cl)Cl)Cl)Cl | 0 | 873 | 2 | 1 |
| PCB 208 (C12HCl9) | XIFFTDRFWYFAPO-UHFFFAOYSA-N | C1=C(C(=C(C(=C1Cl)Cl)C2=C(C(=C(C(=C2Cl)Cl)Cl)Cl)Cl)Cl)Cl | 2829 | 774 | 1 | 1 |
| PCB 207 (C12HCl9) | YGDPIDTZOQGPAX-UHFFFAOYSA-N | C1=C(C(=C(C(=C1Cl)Cl)Cl)C2=C(C(=C(C(=C2Cl)Cl)Cl)Cl)Cl)Cl | 0 | 0 | 2 | 2 |
| phosalone (C12H15ClNO4S2) | IOUNQDKNJZEDEP-UHFFFAOYSA-N | CCOP(=S)(OCC)SCN1C2=C(C=C(C=C2)Cl)OC1=O | 1757 | 0 | 1 | 2 |
| 4,4'-Methylenebis(3-chlorophenyl)diisothiocyanate (C15H8Cl2N2S2) | GDOSNEJTWBTBOC-UHFFFAOYSA-N | C1=CC(=C(C=C1CC2=CC(=C(C=C2)N=C=S)Cl)Cl)N=C=S | 9999 | 6663 | 1 | 1 |
| Diclofop (C15H12Cl2O4) | OOLBCHYXZDXLDS-UHFFFAOYSA-N | CC(C(=O)O)OC1=CC=C(C=C1)OC2=C(C=C(C=C2)Cl)Cl | 6229 | 30 | 1 | 2 |
| Hexachlorophene (C13H6Cl6O2) | ACGUYXCXAPNIKK-UHFFFAOYSA-N | C1=C(C(=C(C(=C1Cl)Cl)CC2=C(C(=CC(=C2Cl)Cl)Cl)O)O)Cl | 959 | 349 | 1 | 1 |
| Iprodione (C13H13Cl2N3O3) | ONUFESLQCSAYKA-UHFFFAOYSA-N | CC(C)NC(=O)N1CC(=O)N(C1=O)C2=CC(=CC(=C2)Cl)Cl | 179 | 0 | 2 | 2 |
| Rotoxamine (C16H19ClN2O) | OJFSXZCBGQGRNV-MRXNPFEDSA-N | CN(C)CCO[C@H](C1=CC=C(C=C1)Cl)C2=CC=CC=N2 | 0 | 0 | 2 | 2 |
| o,p'-Methoxychlor (C16H15Cl3O2) | KNLLPAOBVIKLDE-UHFFFAOYSA-N | COC1=CC=C(C=C1)C(C2=CC=CC=C2OC)C(Cl)(Cl)Cl | 410 | 90 | 2 | 2 |
| Methoxychlor (C16H15Cl3O2) | IAKOZHOLGAGEJT-UHFFFAOYSA-N | COC1=CC=C(C=C1)C(C2=CC=C(C=C2)OC)C(Cl)(Cl)Cl | 230 | 0 | 2 | 2 |
| Phenoxybenzamine (C18H22ClNO) | QZVCTJOXCFMACW-UHFFFAOYSA-N | CC(COC1=CC=CC=C1)N(CCCl)CC2=CC=CC=C2 | 0 | 60 | 2 | 2 |
| Chlorphenoxamine (C18H22ClNO) | KKHPNPMTPORSQE-UHFFFAOYSA-N | CC(C1=CC=CC=C1)(C2=CC=C(C=C2)Cl)OCCN(C)C | 130 | 0 | 2 | 2 |
| Chlorobenzilate (C16H14Cl2O3) | RAPBNVDSDCTNRC-UHFFFAOYSA-N | CCOC(=O)C(C1=CC=C(C=C1)Cl)(C2=CC=C(C=C2)Cl)O | 0 | 0 | 2 | 2 |
| Phenol, 4,4'-(1-methylethylidene)bis[2,6-dichloro- (C15H12Cl4O2) | KYPYTERUKNKOLP-UHFFFAOYSA-N | CC(C)(C1=CC(=C(C(=C1)Cl)O)Cl)C2=CC(=C(C(=C2)Cl)O)Cl | 2389 | 90 | 1 | 2 |
| Bulan (C16H15Cl2NO2) | SXLBEAVIHFLZQB-UHFFFAOYSA-N | CCC(C(C1=CC=C(C=C1)Cl)C2=CC=C(C=C2)Cl)[N+](=O)[O-] | 80 | 0 | 2 | 2 |
| 4-Butylbenzoic acid, 3,4-dichlorophenyl ester (C17H16Cl2O2) | CJIWMGIVONTUCE-UHFFFAOYSA-N | CCCCC1=CC=C(C=C1)C(=O)OC2=CC(=C(C=C2)Cl)Cl | 0 | 0 | 2 | 2 |
| Diclobutrazole (C15H19Cl2N3O) | URDNHJIVMYZFRT-UHFFFAOYSA-N | CC(C)(C)C(C(CC1=C(C=C(C=C1)Cl)Cl)N2C=NC=N2)O | 0 | 358 | 2 | 1 |
| tebuconazole (C16H22ClN3O) | PXMNMQRDXWABCY-UHFFFAOYSA-N | CC(C)(C)C(CCC1=CC=C(C=C1)Cl)(CN2C=NC=N2)O | 150 | 0 | 2 | 2 |
| Dibenzofuran, octachloro- (C12Cl8O) | RHIROFAGUQOFLU-UHFFFAOYSA-N | C12=C(C(=C(C(=C1Cl)Cl)Cl)Cl)OC3=C2C(=C(C(=C3Cl)Cl)Cl)Cl | 3650 | 139 | 1 | 1 |
| 1-Naphthoic acid, 3,4-dichlorophenyl ester (C17H10Cl2O2) | OMRXSGMGDYFBMY-UHFFFAOYSA-N | C1=CC=C2C(=C1)C=CC=C2C(=O)OC3=CC(=C(C=C3)Cl)Cl | 100 | 0 | 2 | 2 |
| Clobazam (C16H13ClN2O2) | CXOXHMZGEKVPMT-UHFFFAOYSA-N | CN1C(=O)CC(=O)N(C2=C1C=CC(=C2)Cl)C3=CC=CC=C3 | 9999 | 0 | 1 | 2 |
| Chlorpromazine (C17H19ClN2S) | ZPEIMTDSQAKGNT-UHFFFAOYSA-N | CN(C)CCCN1C2=CC=CC=C2SC3=C1C=C(C=C3)Cl | 1530 | 0 | 1 | 2 |
| Chlordiazepoxide (C16H14ClN3O) | ANTSCNMPPGJYLG-UHFFFAOYSA-N | CN=C1CN(C(=C2C=C(C=CC2=N1)Cl)C3=CC=CC=C3)O | 3493 | 190 | 1 | 1 |
| Lorazepam (C15H11Cl2N2O2) | DIWRORZWFLOCLC-UHFFFAOYSA-N | C1=CC=C(C(=C1)C2=NC(C(=O)NC3=C2C=C(C=C3)Cl)O)Cl | 0 | 0 | 2 | 2 |
| Chlorcyclizine (C18H21ClN2) | WFNAKBGANONZEQ-UHFFFAOYSA-N | CN1CCN(CC1)C(C2=CC=CC=C2)C3=CC=C(C=C3)Cl | 1361 | 0 | 1 | 2 |
| Benoxaprofen (C16H12ClNO3) | MITFXPHMIHQXPI-UHFFFAOYSA-N | CC(C1=CC2=C(C=C1)OC(=N2)C3=CC=C(C=C3)Cl)C(=O)O | 4974 | 0 | 1 | 2 |
| 1H-1,2,4-Triazole, 1-[[2-(2,4-dichlorophenyl)-4-ethyl-1,3-dioxolan-2-yl]methyl]- (C14H15Cl2N3O2) | DWRKFAJEBUWTQM-UHFFFAOYSA-N | CCC1COC(O1)(CN2C=NC=N2)C3=C(C=C(C=C3)Cl)Cl | 0 | 0 | 2 | 2 |
| Desmethylclomipramine (C18H21ClN2) | VPIXQGUBUKFLRF-UHFFFAOYSA-N | CNCCCN1C2=CC=CC=C2CCC3=C1C=C(C=C3)Cl | 2292 | 0 | 1 | 2 |
| Chlorprothixene (C18H18ClS) | WSPOMRSOLSGNFJ-VGOFMYFVSA-N | CN(C)CC/C=C/1\C2=CC=CC=C2SC3=C1C=C(C=C3)Cl | 80 | 0 | 2 | 2 |
| Endosulfan II (C9H6Cl6O3S) | RDYMFSUJUZBWLH-MDBBVBRHSA-N | C1[C@@H]2[C@H](COS(=O)O1)C3(C(=C(C2(C3(Cl)Cl)Cl)Cl)Cl)Cl | 471 | 0 | 2 | 2 |
| Endosulfan I (C9H6Cl6O3S) | RDYMFSUJUZBWLH-AMHWMVONSA-N | C1[C@@H]2[C@H](COS(=O)O1)[C@]3(C(=C([C@]2(C3(Cl)Cl)Cl)Cl)Cl)Cl | 149 | 146 | 2 | 1 |
| trans-Nonachlor (C10H5Cl9) | OCHOKXCPKDPNQU-BBXWSCHTSA-N | [C@@H]12[C@@H](C(C(C1Cl)Cl)Cl)C3(C(=C(C2(C3(Cl)Cl)Cl)Cl)Cl)Cl | 80 | 3620 | 2 | 1 |
| cis-Nonachlor (C10H5Cl9) | OCHOKXCPKDPNQU-BBXWSCHTSA-N | [C@@H]12[C@@H](C(C(C1Cl)Cl)Cl)C3(C(=C(C2(C3(Cl)Cl)Cl)Cl)Cl)Cl | 120 | 3323 | 2 | 1 |
| Clotiazepam (C16H15ClN2OS) | CHBRHODLKOZEPZ-UHFFFAOYSA-N | CCC1=CC2=C(S1)N(C(=O)CN=C2C3=CC=CC=C3Cl)C | 7676 | 1581 | 1 | 1 |
| Estazolam (C16H11ClN4) | CDCHDCWJMGXXRH-UHFFFAOYSA-N | C1C2=NN=CN2C3=C(C=C(C=C3)Cl)C(=N1)C4=CC=CC=C4 | 6696 | 9999 | 1 | 1 |
| 1-Adamantanecarboxylic acid, 3,4-dichlorophenyl ester (C17H18Cl2O2) | BQWIKWHHKZSZRP-UHFFFAOYSA-N | C1C2CC3CC1CC(C2)(C3)C(=O)OC4=CC(=C(C=C4)Cl)Cl | 0 | 0 | 2 | 2 |
| 2-chlorobenzoic acid, 1-adamantylmethyl ester (C18H21ClO2) | KYXXVMRHEPJPPY-UHFFFAOYSA-N | C1C2CC3CC1CC(C2)(C3)COC(=O)C4=CC=CC=C4Cl | 170 | 0 | 2 | 2 |
| Kepone (C10Cl10O) | LHHGDZSESBACKH-UHFFFAOYSA-N | C1(=O)C2(C3(C4(C1(C5(C2(C3(C(C45Cl)(Cl)Cl)Cl)Cl)Cl)Cl)Cl)Cl)Cl | 63 | 489 | 2 | 1 |
| L-Cysteine, N,S-bis(5-chlorovaleryl)-, methyl ester (C14H23Cl2NO4S) | ICIWTKWNAHYPRV-UHFFFAOYSA-N | COC(=O)C(CSC(=O)CCCCCl)NC(=O)CCCCCl | 0 | 120 | 2 | 1 |
| L-Seine, N,O-bis(5-chlorovaleyl), methyl ester (C14H23Cl2NO5) | AFGCSGILQIJBKA-UHFFFAOYSA-N | COC(=O)C(COC(=O)CCCCCl)NC(=O)CCCCCl | 50 | 2432 | 2 | 1 |
| Succinic acid, nonyl 2,2,2-trichloroethyl ester (C15H25Cl3O4) | LQWIIORAGPTYOQ-UHFFFAOYSA-N | CCCCCCCCCOC(=O)CCC(=O)OCC(Cl)(Cl)Cl | 0 | 0 | 2 | 2 |
| Succinic acid, decyl 2,2-dichloroethyl ester (C16H28Cl2O4) | SMHJYHBHJCREHK-UHFFFAOYSA-N | CCCCCCCCCCOC(=O)CCC(=O)OCC(Cl)Cl | 0 | 0 | 2 | 2 |
| Succinic acid, 10-chlorodecyl propyl ester (C17H31ClO4) | TWAYUEDEMTZMQZ-UHFFFAOYSA-N | CCCOC(=O)CCC(=O)OCCCCCCCCCCCl | 0 | 40 | 2 | 2 |
| Adipic acid, 8-chloroctyl propyl ester (C17H31ClO4) | ININBCUUYLOIJC-UHFFFAOYSA-N | CCCOC(=O)CCCCC(=O)OCCCCCCCCCl | 0 | 50 | 2 | 2 |
| Malonic acid, butyl 10-chlorodecyl ester (C17H31ClO4) | BKYAULOETAIPKU-UHFFFAOYSA-N | CCCCOC(=O)CC(=O)OCCCCCCCCCCCl | 0 | 0 | 2 | 2 |
| Succinic acid, 8-chloroctyl pentyl ester (C17H31ClO4) | ARIFJQNMMCFGDA-UHFFFAOYSA-N | CCCCCOC(=O)CCC(=O)OCCCCCCCCCl | 0 | 10 | 2 | 2 |
| Malonic acid, 10-chlorodecyl isobutyl ester (C17H31ClO4) | IJONSIOAQZZMJJ-UHFFFAOYSA-N | CC(C)COC(=O)CC(=O)OCCCCCCCCCCCl | 0 | 0 | 2 | 2 |
| Malonic acid, 2-chloropropyl undecyl ester (C17H31ClO4) | XNRXLJXCPKUKHW-UHFFFAOYSA-N | CCCCCCCCCCCOC(=O)CC(=O)OCC(C)Cl | 0 | 0 | 2 | 2 |
| Succinic acid, 2-chloropropyl decyl ester (C17H31ClO4) | LWOMMMFGFUGIJW-UHFFFAOYSA-N | CCCCCCCCCCOC(=O)CCC(=O)OCC(C)Cl | 0 | 0 | 2 | 2 |
| Propanoic acid, 3-chloro, hexadecyl ester (C19H37ClO2) | HKMCSNOMIKTPRU-UHFFFAOYSA-N | CCCCCCCCCCCCCCCCOC(=O)CCCl | 0 | 180 | 2 | 1 |
| 5-Chlorovaleric acid, tetradecyl ester (C19H37ClO2) | RUNXJNORIBCNLQ-UHFFFAOYSA-N | CCCCCCCCCCCCCCOC(=O)CCCCCl | 0 | 170 | 2 | 1 |
| 2-Chlorooctadecanoic acid, methyl ester (C19H37ClO2) | ZCHHUNXPPDBGNO-UHFFFAOYSA-N | CCCCCCCCCCCCCCCCC(C(=O)OC)Cl | 280 | 581 | 2 | 1 |
| Propanoic acid, 2-chloro, hexadecyl ester (C19H37ClO2) | UHVQFSUSOVXCEO-UHFFFAOYSA-N | CCCCCCCCCCCCCCCCOC(=O)C(C)Cl | 30 | 0 | 2 | 2 |
| 5-Chlorovaleric acid, 2-tetradecyl ester (C19H37ClO2) | QYENIOAFQYFFAH-UHFFFAOYSA-N | CCCCCCCCCCCCC(C)OC(=O)CCCCCl | 0 | 0 | 2 | 2 |
| 5-Chlorovaleric acid, 3-tetradecyl ester (C19H37ClO2) | VEWBJVOGYRZNCA-UHFFFAOYSA-N | CCCCCCCCCCCC(CC)OC(=O)CCCCCl | 0 | 0 | 2 | 2 |
| 5-Chlorovaleric acid, 4-tetradecyl ester (C19H37ClO2) | GWCIFSFFXLJPKW-UHFFFAOYSA-N | CCCCCCCCCCC(CCC)OC(=O)CCCCCl | 0 | 0 | 2 | 2 |
| Propanamide, N,N-dioctyl-3-chloro- (C19H38ClNO) | SLCJNBBZBLGJLW-UHFFFAOYSA-N | CCCCCCCCN(CCCCCCCC)C(=O)CCCl | 60 | 4964 | 2 | 1 |
| 5-Chlorovaleric acid, 5-tetradecyl ester (C19H37ClO2) | DHPVLVLEERAWKG-UHFFFAOYSA-N | CCCCCCCCCC(CCCC)OC(=O)CCCCCl | 0 | 0 | 2 | 2 |
| Propanamide, N,N-dioctyl-2-chloro- (C19H38ClNO) | SFIDWMGWNSNDKS-UHFFFAOYSA-N | CCCCCCCCN(CCCCCCCC)C(=O)C(C)Cl | 220 | 9999 | 2 | 1 |
| Propanamide, N,N-bis(2-ethylhexyl)-3-chloro- (C19H38ClNO) | LJNYXXQVVFXPTJ-UHFFFAOYSA-N | CCCCC(CC)CN(CC(CC)CCCC)C(=O)CCCl | 60 | 1511 | 2 | 1 |
| Propanamide, N,N-bis(2-ethylhexyl)-2-chloro- (C19H38ClNO) | LUKYKNLMCMZSGG-UHFFFAOYSA-N | CCCCC(CC)CN(CC(CC)CCCC)C(=O)C(C)Cl | 50 | 1531 | 2 | 1 |
| Dichloroacetamide, N,N-dioctyl- (C18H35Cl2NO) | AIUSXCDXRWTYKI-UHFFFAOYSA-N | CCCCCCCCN(CCCCCCCC)C(=O)C(Cl)Cl | 0 | 4724 | 2 | 1 |
| Pentadecyl trichloroacetate (C17H31Cl3O2) | NKIOTTYRINVYJZ-UHFFFAOYSA-N | CCCCCCCCCCCCCCCOC(=O)C(Cl)(Cl)Cl | 0 | 0 | 2 | 2 |
| Fumaric acid, nonyl 2,2,2-trichloroethyl ester (C15H23Cl3O4) | JRFRJYDZGXWGQR-MDZDMXLPSA-N | CCCCCCCCCOC(=O)/C=C/C(=O)OCC(Cl)(Cl)Cl | 0 | 1361 | 2 | 1 |
| Fumaric acid, decyl 2,2-dichloroethyl ester (C16H26Cl2O4) | LAROUSPUHKQQBD-ZHACJKMWSA-N | CCCCCCCCCCOC(=O)/C=C/C(=O)OCC(Cl)Cl | 90 | 240 | 2 | 1 |
| Fumaric acid, 8-chlorooctyl pentyl ester (C17H29ClO4) | YQMHYKJAOJMLTC-VAWYXSNFSA-N | CCCCCOC(=O)/C=C/C(=O)OCCCCCCCCCl | 0 | 20 | 2 | 2 |
| Fumaric acid, 2-chloropropyl decyl ester (C17H29ClO4) | JKNGOSKOOJMAKL-VAWYXSNFSA-N | CCCCCCCCCCOC(=O)/C=C/C(=O)OCC(C)Cl | 0 | 80 | 2 | 2 |
| Succinic acid, butyl 2,3,4,6-tetrachlorophenyl ester (C14H14Cl4O4) | XFVCSOSLMXVDEQ-UHFFFAOYSA-N | CCCCOC(=O)CCC(=O)OC1=C(C(=C(C=C1Cl)Cl)Cl)Cl | 0 | 0 | 2 | 2 |
| Succinic acid, isobutyl 2,3,4,6-tetrachlorophenyl ester (C14H14Cl4O4) | OGMMOLFVSPGGKN-UHFFFAOYSA-N | CC(C)COC(=O)CCC(=O)OC1=C(C(=C(C=C1Cl)Cl)Cl)Cl | 0 | 0 | 2 | 2 |
| Succinic acid, pentyl 2,3,5-trichlorophenyl ester (C15H17Cl3O4) | QASMQLBMTYVURC-UHFFFAOYSA-N | CCCCCOC(=O)CCC(=O)OC1=CC(=CC(=C1Cl)Cl)Cl | 0 | 0 | 2 | 2 |
| Succinic acid, pentyl 2,3,6-trichlorophenyl ester (C15H17Cl3O4) | IWUYEKOIKZVOLM-UHFFFAOYSA-N | CCCCCOC(=O)CCC(=O)OC1=C(C=CC(=C1Cl)Cl)Cl | 0 | 0 | 2 | 2 |
| Sarcosine, N-(2-chlorobenzoyl)-, heptyl ester (C17H24ClNO3) | DSQPSAANSXWBOD-UHFFFAOYSA-N | CCCCCCCOC(=O)CN(C)C(=O)C1=CC=CC=C1Cl | 861 | 70 | 1 | 2 |
| Sarcosine, N-(4-chlorobenzoyl)-, heptyl ester (C17H24ClNO3) | CWCMESYKQQFSHA-UHFFFAOYSA-N | CCCCCCCOC(=O)CN(C)C(=O)C1=CC=C(C=C1)Cl | 1071 | 0 | 1 | 2 |
| Succinic acid, 3,5-dichlorophenyl hexyl ester (C16H20Cl2O4) | OIHQIRJUMDOMDU-UHFFFAOYSA-N | CCCCCCOC(=O)CCC(=O)OC1=CC(=CC(=C1)Cl)Cl | 0 | 0 | 2 | 2 |
| Succinic acid, 3,5-dichlorophenyl isohexyl ester (C16H20Cl2O4) | QCPJJVBYOYWVMV-UHFFFAOYSA-N | CC(C)CCCOC(=O)CCC(=O)OC1=CC(=CC(=C1)Cl)Cl | 0 | 0 | 2 | 2 |
| β-Alanine, N-(2-chlorobenzoyl)-, heptyl ester (C17H24ClNO3) | PRLIOWSYVIEWKX-UHFFFAOYSA-N | CCCCCCCOC(=O)CCNC(=O)C1=CC=CC=C1Cl | 911 | 50 | 1 | 2 |
| β-Alanine, N-(4-chlorobenzoyl)-, heptyl ester (C17H24ClNO3) | UWBPWJOATPCNSY-UHFFFAOYSA-N | CCCCCCCOC(=O)CCNC(=O)C1=CC=C(C=C1)Cl | 721 | 0 | 2 | 2 |
| 2-chlorobenzoic acid, dodec-9-ynyl ester (C19H25ClO2) | SUDQXLLPAJDXDC-UHFFFAOYSA-N | CCC#CCCCCCCCCOC(=O)C1=CC=CC=C1Cl | 0 | 0 | 2 | 2 |
| Dodecyl 4-chlorobenzoate (C19H29ClO2) | AEOLFEGHEDCXAW-UHFFFAOYSA-N | CCCCCCCCCCCCOC(=O)C1=CC=C(C=C1)Cl | 30 | 0 | 2 | 2 |
| Fumaric acid, pentachlorophenyl propyl ester (C13H9Cl5O4) | SZMBLVSWIKXQFX-ONEGZZNKSA-N | CCCOC(=O)/C=C/C(=O)OC1=C(C(=C(C(=C1Cl)Cl)Cl)Cl)Cl | 40 | 0 | 2 | 2 |
| Fumaric acid, butyl 2,3,4,6-tetrachlorophenyl ester (C14H12Cl4O4) | BPFAPIRCWGJTKJ-SNAWJCMRSA-N | CCCCOC(=O)/C=C/C(=O)OC1=C(C(=C(C=C1Cl)Cl)Cl)Cl | 40 | 0 | 2 | 2 |
| Fumaric acid, butyl 2,3,4,5-tetrachlorophenyl ester (C14H12Cl4O4) | MKDPKNSLVYULAO-SNAWJCMRSA-N | CCCCOC(=O)/C=C/C(=O)OC1=CC(=C(C(=C1Cl)Cl)Cl)Cl | 20 | 0 | 2 | 2 |
| Fumaric acid, butyl 2,3,5,6-tetrachlorophenyl ester (C14H12Cl4O4) | ZGANIIKWTLMKME-SNAWJCMRSA-N | CCCCOC(=O)/C=C/C(=O)OC1=C(C(=CC(=C1Cl)Cl)Cl)Cl | 70 | 0 | 2 | 2 |
| Fumaric acid, isobutyl 2,3,4,6-tetrachlorophenyl ester (C14H12Cl4O4) | URUVAKSJVJOFGY-ONEGZZNKSA-N | CC(C)COC(=O)/C=C/C(=O)OC1=C(C(=C(C=C1Cl)Cl)Cl)Cl | 50 | 0 | 2 | 2 |
| Fumaric acid, isobutyl 2,3,4,5-tetrachlorophenyl ester (C14H12Cl4O4) | LSWQEYJKXOOGPU-ONEGZZNKSA-N | CC(C)COC(=O)/C=C/C(=O)OC1=CC(=C(C(=C1Cl)Cl)Cl)Cl | 20 | 0 | 2 | 2 |
| Fumaric acid, isobutyl 2,3,5,6-tetrachlorophenyl ester (C14H12Cl4O4) | IRHRNSOQRLRWJR-ONEGZZNKSA-N | CC(C)COC(=O)/C=C/C(=O)OC1=C(C(=CC(=C1Cl)Cl)Cl)Cl | 80 | 0 | 2 | 2 |
| Fumaric acid, pentyl 2,4,5-trichlorophenyl ester (C15H15Cl3O4) | DQZQYEZXJUHJHZ-AATRIKPKSA-N | CCCCCOC(=O)/C=C/C(=O)OC1=CC(=C(C=C1Cl)Cl)Cl | 40 | 0 | 2 | 2 |
| Fumaric acid, pentyl 2,3,5-trichlorophenyl ester (C15H15Cl3O4) | HNNVLCVOFXJZLA-AATRIKPKSA-N | CCCCCOC(=O)/C=C/C(=O)OC1=CC(=CC(=C1Cl)Cl)Cl | 30 | 0 | 2 | 2 |
| Fumaric acid, pentyl 3,4,5-trichlorophenyl ester (C15H15Cl3O4) | ALDAXBNYLCOBDS-AATRIKPKSA-N | CCCCCOC(=O)/C=C/C(=O)OC1=CC(=C(C(=C1)Cl)Cl)Cl | 30 | 0 | 2 | 2 |
| Fumaric acid, pentyl 2,3,6-trichlorophenyl ester (C15H15Cl3O4) | XANIAYLQQMRBCA-BQYQJAHWSA-N | CCCCCOC(=O)/C=C/C(=O)OC1=C(C=CC(=C1Cl)Cl)Cl | 90 | 0 | 2 | 2 |
| Fumaric acid, pentyl 2,4,6-trichlorophenyl ester (C15H15Cl3O4) | KQBJGULELVKICC-AATRIKPKSA-N | CCCCCOC(=O)/C=C/C(=O)OC1=C(C=C(C=C1Cl)Cl)Cl | 20 | 0 | 2 | 2 |
| Fumaric acid, 3,5-dichlorophenyl hexyl ester (C16H18Cl2O4) | HKPGBKSEDUOXAX-VOTSOKGWSA-N | CCCCCCOC(=O)/C=C/C(=O)OC1=CC(=CC(=C1)Cl)Cl | 20 | 0 | 2 | 2 |
| Fumaric acid, 3,5-dichlorophenyl isohexyl ester (C16H18Cl2O4) | OHLCSBOWTPMGNR-AATRIKPKSA-N | CC(C)CCCOC(=O)/C=C/C(=O)OC1=CC(=CC(=C1)Cl)Cl | 20 | 0 | 2 | 2 |
| Fumaric acid, 2-chloro-5-methylphenyl hexyl ester (C17H21ClO4) | FAOAWVAOBJOJEV-MDZDMXLPSA-N | CCCCCCOC(=O)/C=C/C(=O)OC1=C(C=CC(=C1)C)Cl | 250 | 120 | 2 | 1 |
| Fumaric acid, 2-chloro-5-methylphenyl isohexyl ester (C17H21ClO4) | TYLIIEZFLRWKOL-CMDGGOBGSA-N | CC1=CC(=C(C=C1)Cl)OC(=O)/C=C/C(=O)OCCCC(C)C | 340 | 150 | 2 | 1 |
| Bifenox (C14H9Cl2NO3) | SUSRORUBZHMPCO-UHFFFAOYSA-N | COC(=O)C1=C(C=CC(=C1)OC2=C(C=C(C=C2)Cl)Cl)[N+](=O)[O-] | 9999 | 0 | 1 | 2 |
| Decachlorobiphenyl (C12Cl10) | ONXPZLFXDMAPRO-UHFFFAOYSA-N | C1(=C(C(=C(C(=C1Cl)Cl)Cl)Cl)Cl)C2=C(C(=C(C(=C2Cl)Cl)Cl)Cl)Cl | 2163 | 0 | 1 | 2 |
| Diclofop-methyl (C16H14Cl2O3) | BACHBFVBHLGWSL-UHFFFAOYSA-N | CC(C(=O)OC)OC1=CC=C(C=C1)OC2=C(C=C(C=C2)Cl)Cl | 8009 | 0 | 1 | 2 |
| L-Phenylalanine, N-(2-chlorobenzoyl)-, methyl ester (C17H16ClNO3) | SBBIHHYZJMQAMT-UHFFFAOYSA-N | COC(=O)C(CC1=CC=CC=C1)NC(=O)C2=CC=CC=C2Cl | 40 | 0 | 2 | 2 |
| 5-Chlorovaleric acid, 4-benzyloxyphenyl ester (C18H19ClO3) | WXPUTBOXHOZGMQ-UHFFFAOYSA-N | C1=CC=C(C=C1)COC2=CC=C(C=C2)OC(=O)CCCCCl | 220 | 0 | 2 | 2 |
| Chloropropylate (C17H16Cl2O3) | AXGUBXVWZBFQGA-UHFFFAOYSA-N | CC(C)OC(=O)C(C1=CC=C(C=C1)Cl)(C2=CC=C(C=C2)Cl)O | 0 | 0 | 2 | 2 |
| Oxadiazon (C15H18Cl2N2O3) | CHNUNORXWHYHNE-UHFFFAOYSA-N | CC(C)OC1=C(C=C(C(=C1)N2C(=O)OC(=N2)C(C)(C)C)Cl)Cl | 1707 | 0 | 1 | 2 |
| Chloroquine (C18H26ClN3) | WHTVZRBIWZFKQO-UHFFFAOYSA-N | CCN(CC)CCCC(C)NC1=C2C=CC(=CC2=NC=C1)Cl | 630 | 0 | 2 | 2 |
| Coumaphos O-analog (C14H16ClO6P) | FDYMERLIFOUIRZ-UHFFFAOYSA-N | CCOP(=O)(OCC)OC1=CC2=C(C=C1)C(=C(C(=O)O2)Cl)C | 1571 | 0 | 1 | 2 |
| Coumaphos (C14H16ClO5PS) | BXNANOICGRISHX-UHFFFAOYSA-N | CCOP(=S)(OCC)OC1=CC2=C(C=C1)C(=C(C(=O)O2)Cl)C | 7819 | 0 | 1 | 2 |
| Dibenzo[b,e][1,4]dioxin, octachloro- (C12Cl8O2) | FOIBFBMSLDGNHL-UHFFFAOYSA-N | C12=C(C(=C(C(=C1Cl)Cl)Cl)Cl)OC3=C(O2)C(=C(C(=C3Cl)Cl)Cl)Cl | 2800 | 200 | 1 | 1 |
| Tris(4-chlorophenyl)phosphine (C18H12Cl3P) | IQKSLJOIKWOGIZ-UHFFFAOYSA-N | C1=CC(=CC=C1P(C2=CC=C(C=C2)Cl)C3=CC=C(C=C3)Cl)Cl | 9549 | 222 | 1 | 1 |
| Tris(3-chlorophenyl)phosphine (C18H12Cl3P) | QAPGHLJQIVDTPT-UHFFFAOYSA-N | C1=CC(=CC(=C1)Cl)P(C2=CC(=CC=C2)Cl)C3=CC(=CC=C3)Cl | 9999 | 330 | 1 | 1 |
| Clonazepam (C15H10ClN3O3) | DGBIGWXXNGSACT-UHFFFAOYSA-N | C1C(=O)NC2=C(C=C(C=C2)[N+](=O)[O-])C(=N1)C3=CC=CC=C3Cl | 5725 | 9999 | 1 | 1 |
| Chlorthalidone (C14H11ClN2O4S) | JIVPVXMEBJLZRO-UHFFFAOYSA-N | C1=CC=C2C(=C1)C(=O)NC2(C3=CC(=C(C=C3)Cl)S(=O)(=O)N)O | 1231 | 0 | 1 | 2 |
| Lormetazepam (C16H12Cl2N2O2) | FJIKWRGCXUCUIG-UHFFFAOYSA-N | CN1C2=C(C=C(C=C2)Cl)C(=NC(C1=O)O)C3=CC=CC=C3Cl | 970 | 220 | 1 | 1 |
| Fenarimol (C17H12Cl2N2O) | NHOWDZOIZKMVAI-UHFFFAOYSA-N | C1=CC=C(C(=C1)C(C2=CC=C(C=C2)Cl)(C3=CN=CN=C3)O)Cl | 1800 | 790 | 1 | 1 |
| p-Anisylchlorodiphenylmethane (C20H17ClO) | OBOHMJWDFPBPKD-UHFFFAOYSA-N | COC1=CC=C(C=C1)C(C2=CC=CC=C2)(C3=CC=CC=C3)Cl | 10 | 9999 | 2 | 1 |
| Pinazepam (C18H13ClN2O) | MFZOSKPPVCIFMT-UHFFFAOYSA-N | C#CCN1C(=O)CN=C(C2=C1C=CC(=C2)Cl)C3=CC=CC=C3 | 9700 | 450 | 1 | 1 |
| Clomipramine (C19H23ClN2) | GDLIGKIOYRNHDA-UHFFFAOYSA-N | CN(C)CCCN1C2=CC=CC=C2CCC3=C1C=C(C=C3)Cl | 741 | 10 | 2 | 2 |
| Pyrrobutamine (C20H22ClN) | WDYYVNNRTDZKAZ-UNOMPAQXSA-N | C1CCN(C1)C/C=C(/CC2=CC=C(C=C2)Cl)\C3=CC=CC=C3 | 220 | 0 | 2 | 2 |
| Amoxapine (C17H16ClN3O) | QWGDMFLQWFTERH-UHFFFAOYSA-N | C1CN(CCN1)C2=NC3=CC=CC=C3OC4=C2C=C(C=C4)Cl | 581 | 10 | 2 | 2 |
| Alprazolam (C17H13ClN4) | VREFGVBLTWBCJP-UHFFFAOYSA-N | CC1=NN=C2N1C3=C(C=C(C=C3)Cl)C(=NC2)C4=CC=CC=C4 | 7326 | 5795 | 1 | 1 |
| 2-chlorobenzoic acid, 2-(1-adamantyl)ethyl ester (C19H23ClO2) | DUPQEGSBYNXUSQ-UHFFFAOYSA-N | C1C2CC3CC1CC(C2)(C3)CCOC(=O)C4=CC=CC=C4Cl | 0 | 0 | 2 | 2 |
| Isodrin (C12H8Cl6) | QBYJBZPUGVGKQQ-DIFDVCDBSA-N | C1[C@@H]2C=C[C@H]1[C@@H]3[C@H]2[C@@]4(C(=C([C@]3(C4(Cl)Cl)Cl)Cl)Cl)Cl | 1231 | 991 | 1 | 1 |
| Mirex (C10Cl12) | GVYLCNUFSHDAAW-UHFFFAOYSA-N | C12(C3(C4(C5(C3(C(C1(C5(C2(C4(Cl)Cl)Cl)Cl)Cl)(Cl)Cl)Cl)Cl)Cl)Cl)Cl | 0 | 0 | 2 | 2 |
| Succinic acid, decyl 2,2,2-trichloroethyl ester (C16H27Cl3O4) | FAIYXLQCQHTADH-UHFFFAOYSA-N | CCCCCCCCCCOC(=O)CCC(=O)OCC(Cl)(Cl)Cl | 0 | 0 | 2 | 2 |
| Succinic acid, 2,2-dichloroethyl undecyl ester (C17H30Cl2O4) | SATXPBVLRSFJFY-UHFFFAOYSA-N | CCCCCCCCCCCOC(=O)CCC(=O)OCC(Cl)Cl | 0 | 0 | 2 | 2 |
| 1-chlorodocosane (C22H45Cl) | OACXFSZVCDOBKF-UHFFFAOYSA-N | CCCCCCCCCCCCCCCCCCCCCCCl | 499 | 20 | 2 | 2 |
| 5-Chlorovaleric acid, pentadecyl ester (C20H39ClO2) | FGQMDMNLZQATFL-UHFFFAOYSA-N | CCCCCCCCCCCCCCCOC(=O)CCCCCl | 0 | 150 | 2 | 1 |
| Malonic acid, 10-chlorodecyl pentyl ester (C18H33ClO4) | LIVKUMQJJABWOO-UHFFFAOYSA-N | CCCCCOC(=O)CC(=O)OCCCCCCCCCCCl | 0 | 0 | 2 | 2 |
| Succinic acid, butyl 10-chlorodecyl ester (C18H33ClO4) | LMHBNPLNRUVHBM-UHFFFAOYSA-N | CCCCOC(=O)CCC(=O)OCCCCCCCCCCCl | 0 | 40 | 2 | 2 |
| Succinic acid, 8-chloroctyl hexyl ester (C18H33ClO4) | OSANRUGTBBHCED-UHFFFAOYSA-N | CCCCCCOC(=O)CCC(=O)OCCCCCCCCCl | 0 | 30 | 2 | 2 |
| Adipic acid, butyl 8-chloroctyl ester (C18H33ClO4) | HETXEZMDDSWKRG-UHFFFAOYSA-N | CCCCOC(=O)CCCCC(=O)OCCCCCCCCCl | 0 | 40 | 2 | 2 |
| 5-Chlorovaleric acid, 2-pentadecyl ester (C20H39ClO2) | SYONAWJMYCBGLY-UHFFFAOYSA-N | CCCCCCCCCCCCCC(C)OC(=O)CCCCCl | 0 | 0 | 2 | 2 |
| 5-Chlorovaleric acid, 3-pentadecyl ester (C20H39ClO2) | KMJZPWWNSUGDJP-UHFFFAOYSA-N | CCCCCCCCCCCCC(CC)OC(=O)CCCCCl | 0 | 0 | 2 | 2 |
| 5-Chlorovaleric acid, 4-pentadecyl ester (C20H39ClO2) | QIWXHHYNGMQWJI-UHFFFAOYSA-N | CCCCCCCCCCCC(CCC)OC(=O)CCCCCl | 0 | 0 | 2 | 2 |
| Chloroacetamide, N,N-dinonyl- (C20H40ClNO) | MPFKIPYWOBSVSB-UHFFFAOYSA-N | CCCCCCCCCN(CCCCCCCCC)C(=O)CCl | 40 | 5275 | 2 | 1 |
| 5-Chlorovaleric acid, 5-pentadecyl ester (C20H39ClO2) | WRDBNJHXOVIVGG-UHFFFAOYSA-N | CCCCCCCCCCC(CCCC)OC(=O)CCCCCl | 0 | 0 | 2 | 2 |
| Succinic acid, 10-chlorodecyl isobutyl ester (C18H33ClO4) | JLLUCWADVCLWOF-UHFFFAOYSA-N | CC(C)COC(=O)CCC(=O)OCCCCCCCCCCCl | 0 | 50 | 2 | 2 |
| Adipic acid, 8-chloroctyl isobutyl ester (C18H33ClO4) | MNXUVEYWINNEBR-UHFFFAOYSA-N | CC(C)COC(=O)CCCCC(=O)OCCCCCCCCCl | 0 | 40 | 2 | 2 |
| Malonic acid, 2-chloropropyl dodecyl ester (C18H33ClO4) | OBRFKQKHEAIOND-UHFFFAOYSA-N | CCCCCCCCCCCCOC(=O)CC(=O)OCC(C)Cl | 0 | 20 | 2 | 2 |
| Succinic acid, 2-chloropropyl undecyl ester (C18H33ClO4) | FPAQEJPTAVGUEC-UHFFFAOYSA-N | CCCCCCCCCCCOC(=O)CCC(=O)OCC(C)Cl | 0 | 0 | 2 | 2 |
| Methyl 9,10-dichlorooctadecanoate (C19H36Cl2O2) | KYPMPURXQCFWCJ-UHFFFAOYSA-N | CCCCCCCCC(C(CCCCCCCC(=O)OC)Cl)Cl | 14 | 10 | 2 | 2 |
| Hexadecyl trichloroacetate (C18H33Cl3O2) | CCFJXGUHYQBXDT-UHFFFAOYSA-N | CCCCCCCCCCCCCCCCOC(=O)C(Cl)(Cl)Cl | 0 | 0 | 2 | 2 |
| Trichloroacetamide, N,N-bis(2-ethylhexyl)- (C18H34Cl3NO) | AQTKUBSOCLDIJW-UHFFFAOYSA-N | CCCCC(CC)CN(CC(CC)CCCC)C(=O)C(Cl)(Cl)Cl | 0 | 90 | 2 | 2 |
| Fumaric acid, decyl 2,2,2-trichloroethyl ester (C16H25Cl3O4) | KZHVAYZESXTNDB-ZHACJKMWSA-N | CCCCCCCCCCOC(=O)/C=C/C(=O)OCC(Cl)(Cl)Cl | 0 | 901 | 2 | 1 |
| Fumaric acid, 2,2-dichloroethyl undecyl ester (C17H28Cl2O4) | QRWWZUHTBJNFPP-VAWYXSNFSA-N | CCCCCCCCCCCOC(=O)/C=C/C(=O)OCC(Cl)Cl | 0 | 260 | 2 | 1 |
| Fumaric acid, butyl 10-chlorodecyl ester (C18H31ClO4) | LFWSBNSBIUWVAS-OUKQBFOZSA-N | CCCCOC(=O)/C=C/C(=O)OCCCCCCCCCCCl | 0 | 30 | 2 | 2 |
| Fumaric acid, 8-chlorooctyl hexyl ester (C18H31ClO4) | FXMZBPMEEOHGHL-OUKQBFOZSA-N | CCCCCCOC(=O)/C=C/C(=O)OCCCCCCCCCl | 0 | 20 | 2 | 2 |
| Fumaric acid, 10-chlorodecyl isobutyl ester (C18H31ClO4) | PEYWTQAYPGMLGO-VAWYXSNFSA-N | CC(C)COC(=O)/C=C/C(=O)OCCCCCCCCCCCl | 0 | 0 | 2 | 2 |
| Fumaric acid, 8-chlorooctyl isohexyl ester (C18H31ClO4) | NBCAIVJHQAZWID-VAWYXSNFSA-N | CC(C)CCCOC(=O)/C=C/C(=O)OCCCCCCCCCl | 0 | 0 | 2 | 2 |
| Fumaric acid, 2-chloropropyl undecyl ester (C18H31ClO4) | PJJXMPHMXBTGDX-OUKQBFOZSA-N | CCCCCCCCCCCOC(=O)/C=C/C(=O)OCC(C)Cl | 0 | 70 | 2 | 2 |
| Succinic acid, pentyl 2,3,4,6-tetrachlorophenyl ester (C15H16Cl4O4) | HOZYPLZLDHHARN-UHFFFAOYSA-N | CCCCCOC(=O)CCC(=O)OC1=C(C(=C(C=C1Cl)Cl)Cl)Cl | 0 | 0 | 2 | 2 |
| Succinic acid, hexyl 2,3,5-trichlorophenyl ester (C16H19Cl3O4) | WGARQZCLZJLLFU-UHFFFAOYSA-N | CCCCCCOC(=O)CCC(=O)OC1=CC(=CC(=C1Cl)Cl)Cl | 0 | 0 | 2 | 2 |
| Succinic acid, hexyl 2,3,6-trichlorophenyl ester (C16H19Cl3O4) | WJUQYRSXVMOFHU-UHFFFAOYSA-N | CCCCCCOC(=O)CCC(=O)OC1=C(C=CC(=C1Cl)Cl)Cl | 0 | 0 | 2 | 2 |
| Succinic acid, isohexyl 2,3,5-trichlorophenyl ester (C16H19Cl3O4) | QBLWHUHQTUEOND-UHFFFAOYSA-N | CC(C)CCCOC(=O)CCC(=O)OC1=CC(=CC(=C1Cl)Cl)Cl | 0 | 0 | 2 | 2 |
| Succinic acid, isohexyl 2,3,6-trichlorophenyl ester (C16H19Cl3O4) | YYGDLSDNSCQSND-UHFFFAOYSA-N | CC(C)CCCOC(=O)CCC(=O)OC1=C(C=CC(=C1Cl)Cl)Cl | 0 | 0 | 2 | 2 |
| Sarcosine, N-(2-chlorobenzoyl)-, octyl ester (C18H26ClNO3) | CEZWRROFJUFQGM-UHFFFAOYSA-N | CCCCCCCCOC(=O)CN(C)C(=O)C1=CC=CC=C1Cl | 1682 | 130 | 1 | 1 |
| Sarcosine, N-(4-chlorobenzoyl)-, octyl ester (C18H26ClNO3) | DRFOMTJWBVHUCF-UHFFFAOYSA-N | CCCCCCCCOC(=O)CN(C)C(=O)C1=CC=C(C=C1)Cl | 1101 | 0 | 1 | 2 |
| Succinic acid, 3,5-dichlorophenyl heptyl ester (C17H22Cl2O4) | HJVKPOCPRXIFDN-UHFFFAOYSA-N | CCCCCCCOC(=O)CCC(=O)OC1=CC(=CC(=C1)Cl)Cl | 0 | 0 | 2 | 2 |
| β-Alanine, N-(2-chlorobenzoyl)-, octyl ester (C18H26ClNO3) | KFPBBCBFEHVJHC-UHFFFAOYSA-N | CCCCCCCCOC(=O)CCNC(=O)C1=CC=CC=C1Cl | 901 | 70 | 1 | 2 |
| β-Alanine, N-(4-chlorobenzoyl)-, octyl ester (C18H26ClNO3) | BTYTZIBBNKDRCF-UHFFFAOYSA-N | CCCCCCCCOC(=O)CCNC(=O)C1=CC=C(C=C1)Cl | 280 | 0 | 2 | 2 |
| 2-Chlorobenzoic acid, tridec-2-ynyl ester (C20H27ClO2) | RDJRHBFOTCUIHH-UHFFFAOYSA-N | CCCCCCCCCCC#CCOC(=O)C1=CC=CC=C1Cl | 0 | 0 | 2 | 2 |
| 4-Chlorobenzoic acid, tridec-2-ynyl ester (C20H27ClO2) | NZIKKCRTTFBRTC-UHFFFAOYSA-N | CCCCCCCCCCC#CCOC(=O)C1=CC=C(C=C1)Cl | 0 | 0 | 2 | 2 |
| 2-Chlorobenzoic acid, tridecyl ester (C20H31ClO2) | RIBWAZGMFLYEHT-UHFFFAOYSA-N | CCCCCCCCCCCCCOC(=O)C1=CC=CC=C1Cl | 40 | 0 | 2 | 2 |
| 2-Chlorobenzoic acid, 2-tridecyl ester (C20H31ClO2) | DPTUHKPBRAEVRJ-UHFFFAOYSA-N | CCCCCCCCCCCC(C)OC(=O)C1=CC=CC=C1Cl | 0 | 0 | 2 | 2 |
| 2-Chlorobenzoic acid, 3-tridecyl ester (C20H31ClO2) | STPUHZZTPBQLLR-UHFFFAOYSA-N | CCCCCCCCCCC(CC)OC(=O)C1=CC=CC=C1Cl | 0 | 0 | 2 | 2 |
| 2-Chlorobenzoic acid, 4-tridecyl ester (C20H31ClO2) | AJBVRWQRFTWIJO-UHFFFAOYSA-N | CCCCCCCCCC(CCC)OC(=O)C1=CC=CC=C1Cl | 0 | 0 | 2 | 2 |
| Fumaric acid, butyl pentachlorophenyl ester (C14H11Cl5O4) | FWAPVTJQGLXHOP-SNAWJCMRSA-N | CCCCOC(=O)/C=C/C(=O)OC1=C(C(=C(C(=C1Cl)Cl)Cl)Cl)Cl | 20 | 0 | 2 | 2 |
| Fumaric acid, isobutyl pentachlorophenyl ester (C14H11Cl5O4) | RRORQPBYPGTCSN-ONEGZZNKSA-N | CC(C)COC(=O)/C=C/C(=O)OC1=C(C(=C(C(=C1Cl)Cl)Cl)Cl)Cl | 30 | 0 | 2 | 2 |
| Fumaric acid, pentyl 2,3,4,6-tetrachlorophenyl ester (C15H14Cl4O4) | JHGVKYBIGKRNCD-AATRIKPKSA-N | CCCCCOC(=O)/C=C/C(=O)OC1=C(C(=C(C=C1Cl)Cl)Cl)Cl | 30 | 0 | 2 | 2 |
| Fumaric acid, pentyl 2,3,4,5-tetrachlorophenyl ester (C15H14Cl4O4) | DCJDIWNEHHPQNL-AATRIKPKSA-N | CCCCCOC(=O)/C=C/C(=O)OC1=CC(=C(C(=C1Cl)Cl)Cl)Cl | 0 | 0 | 2 | 2 |
| Fumaric acid, pentyl 2,3,5,6-tetrachlorophenyl ester (C15H14Cl4O4) | SPGQBUMQGUSKMU-AATRIKPKSA-N | CCCCCOC(=O)/C=C/C(=O)OC1=C(C(=CC(=C1Cl)Cl)Cl)Cl | 40 | 0 | 2 | 2 |
| Fumaric acid, hexyl 2,4,5-trichlorophenyl ester (C16H17Cl3O4) | GUROSZTYDBBLBY-VOTSOKGWSA-N | CCCCCCOC(=O)/C=C/C(=O)OC1=CC(=C(C=C1Cl)Cl)Cl | 30 | 0 | 2 | 2 |
| Fumaric acid, hexyl 2,3,5-trichlorophenyl ester (C16H17Cl3O4) | KTRYCANXHJSDCB-VOTSOKGWSA-N | CCCCCCOC(=O)/C=C/C(=O)OC1=CC(=CC(=C1Cl)Cl)Cl | 30 | 0 | 2 | 2 |
| Fumaric acid, hexyl 3,4,5-trichlorophenyl ester (C16H17Cl3O4) | AEMQNCQCPLKIPB-VOTSOKGWSA-N | CCCCCCOC(=O)/C=C/C(=O)OC1=CC(=C(C(=C1)Cl)Cl)Cl | 30 | 0 | 2 | 2 |
| Fumaric acid, hexyl 2,3,6-trichlorophenyl ester (C16H17Cl3O4) | VUMBEVNGFWMEEA-CMDGGOBGSA-N | CCCCCCOC(=O)/C=C/C(=O)OC1=C(C=CC(=C1Cl)Cl)Cl | 90 | 0 | 2 | 2 |
| Fumaric acid, hexyl 2,4,6-trichlorophenyl ester (C16H17Cl3O4) | DJNHYRDGHUYGCX-VOTSOKGWSA-N | CCCCCCOC(=O)/C=C/C(=O)OC1=C(C=C(C=C1Cl)Cl)Cl | 20 | 0 | 2 | 2 |
| Fumaric acid, isohexyl 2,4,5-trichlorophenyl ester (C16H17Cl3O4) | OCSRHJAISVNNIV-AATRIKPKSA-N | CC(C)CCCOC(=O)/C=C/C(=O)OC1=CC(=C(C=C1Cl)Cl)Cl | 40 | 0 | 2 | 2 |
| Fumaric acid, isohexyl 2,3,5-trichlorophenyl ester (C16H17Cl3O4) | ZJEUAPZXNZRZAM-AATRIKPKSA-N | CC(C)CCCOC(=O)/C=C/C(=O)OC1=CC(=CC(=C1Cl)Cl)Cl | 30 | 0 | 2 | 2 |
| Fumaric acid, isohexyl 3,4,5-trichlorophenyl ester (C16H17Cl3O4) | HDCIXOJTYKFIFX-AATRIKPKSA-N | CC(C)CCCOC(=O)/C=C/C(=O)OC1=CC(=C(C(=C1)Cl)Cl)Cl | 30 | 0 | 2 | 2 |
| Fumaric acid, isohexyl 2,3,6-trichlorophenyl ester (C16H17Cl3O4) | JVPKAVVZKCYBTO-BQYQJAHWSA-N | CC(C)CCCOC(=O)/C=C/C(=O)OC1=C(C=CC(=C1Cl)Cl)Cl | 100 | 0 | 2 | 2 |
| Fumaric acid, isohexyl 2,4,6-trichlorophenyl ester (C16H17Cl3O4) | VJWIRWKRNBZDAC-AATRIKPKSA-N | CC(C)CCCOC(=O)/C=C/C(=O)OC1=C(C=C(C=C1Cl)Cl)Cl | 20 | 0 | 2 | 2 |
| Fumaric acid, 3,5-dichlorophenyl heptyl ester (C17H20Cl2O4) | PKNSQHHAFHSLMA-BQYQJAHWSA-N | CCCCCCCOC(=O)/C=C/C(=O)OC1=CC(=CC(=C1)Cl)Cl | 0 | 0 | 2 | 2 |
| Chlorsulfuron (C12H12ClN5O4S) | VJYIFXVZLXQVHO-UHFFFAOYSA-N | CC1=NC(=NC(=N1)OC)NC(=O)NS(=O)(=O)C2=CC=CC=C2Cl | 79 | 286 | 2 | 1 |
| N-(1,1-Bis(4-chlorophenyl)-2,2,2-trichloroethyl)acetamide (C16H12Cl5NO) | MCSLBZGLEIGWTI-UHFFFAOYSA-N | CC(=O)NC(C1=CC=C(C=C1)Cl)(C2=CC=C(C=C2)Cl)C(Cl)(Cl)Cl | 60 | 2869 | 2 | 1 |
| Dialifor (C14H17ClNO4PS2) | MUMQYXACQUZOFP-UHFFFAOYSA-N | CCOP(=S)(OCC)SC(CCl)N1C(=O)C2=CC=CC=C2C1=O | 0 | 0 | 2 | 2 |
| Hydroxychloroquine (C18H26ClN3O) | XXSMGPRMXLTPCZ-UHFFFAOYSA-N | CCN(CCCC(C)NC1=C2C=CC(=CC2=NC=C1)Cl)CCO | 180 | 0 | 2 | 2 |
| N-propyl-N-[2-(2,4,6-trichlorophenoxy)ethyl]-1H-imidazole-1-carboxamide (C15H16Cl3N3O2) | TVLSRXXIMLFWEO-UHFFFAOYSA-N | CCCN(CCOC1=C(C=C(C=C1Cl)Cl)Cl)C(=O)N2C=CN=C2 | 0 | 0 | 2 | 2 |
| 9H-Purine-9-acetic acid, 6-[(p-chlorophenyl)amino)-, ethyl ester (C15H14ClN5O2) | GRZHGZVUENNXNQ-UHFFFAOYSA-N | CCOC(=O)CN1C=NC2=C1N=CN=C2NC3=CC=C(C=C3)Cl | 9999 | 140 | 1 | 1 |
| Pencycuron (C19H21ClN2O) | OGYFATSSENRIKG-UHFFFAOYSA-N | C1CCC(C1)N(CC2=CC=C(C=C2)Cl)C(=O)NC3=CC=CC=C3 | 2142 | 6 | 1 | 2 |
| Cloperastine (C20H24ClNO) | FLNXBVJLPJNOSI-UHFFFAOYSA-N | C1CCN(CC1)CCOC(C2=CC=CC=C2)C3=CC=C(C=C3)Cl | 0 | 0 | 2 | 2 |
| 6-Chloro-N,N'-(cycloheptyl)-[1,3,5]triazine-2,4-diamine (C17H28ClN5) | NDHBZABQERFFOL-UHFFFAOYSA-N | C1CCCC(CC1)NC2=NC(=NC(=N2)Cl)NC3CCCCCC3 | 4978 | 3450 | 1 | 1 |
| Amoxapine M (7-hydroxy) (C17H16ClN3O2) | MEUGUMOVYNSGEW-UHFFFAOYSA-N | C1CN(CCN1)C2=NC3=C(C=C(C=C3)O)OC4=C2C=C(C=C4)Cl | 1121 | 0 | 1 | 2 |
| Clothiapine (C18H18ClN3S) | KAAZGXDPUNNEFN-UHFFFAOYSA-N | CN1CCN(CC1)C2=NC3=CC=CC=C3SC4=C2C=C(C=C4)Cl | 821 | 0 | 1 | 2 |
| Clozapine (C18H19ClN4) | QZUDBNBUXVUHMW-UHFFFAOYSA-N | CN1CCN(CC1)C2=C3C=CC=CC3=NC4=C(N2)C=C(C=C4)Cl | 1311 | 0 | 1 | 2 |
| Loxapine (C18H18ClN3O) | XJGVXQDUIWGIRW-UHFFFAOYSA-N | CN1CCN(CC1)C2=NC3=CC=CC=C3OC4=C2C=C(C=C4)Cl | 1161 | 0 | 1 | 2 |
| Cloxazolam (C17H14Cl2N2O2) | ZIXNZOBDFKSQTC-UHFFFAOYSA-N | C1COC2(N1CC(=O)NC3=C2C=C(C=C3)Cl)C4=CC=CC=C4Cl | 0 | 0 | 2 | 2 |
| Triazolam (C17H12Cl2N4) | JOFWLTCLBGQGBO-UHFFFAOYSA-N | CC1=NN=C2N1C3=C(C=C(C=C3)Cl)C(=NC2)C4=CC=CC=C4Cl | 4864 | 761 | 1 | 1 |
| Clemizole (C19H20ClN3) | CJXAEXPPLWQRFR-UHFFFAOYSA-N | C1CCN(C1)CC2=NC3=CC=CC=C3N2CC4=CC=C(C=C4)Cl | 0 | 0 | 2 | 2 |
| Oxazolam (C18H17ClN2O2) | VCCZBYPHZRWKFY-UHFFFAOYSA-N | CC1CN2CC(=O)NC3=C(C2(O1)C4=CC=CC=C4)C=C(C=C3)Cl | 70 | 0 | 2 | 2 |
| Prazepam (C19H17ClN2O) | MWQCHHACWWAQLJ-UHFFFAOYSA-N | C1CC1CN2C(=O)CN=C(C3=C2C=CC(=C3)Cl)C4=CC=CC=C4 | 5314 | 200 | 1 | 1 |
| Succinic acid, 2,2,2-trichloroethyl undecyl ester (C17H29Cl3O4) | IDHHVODZKZKOPI-UHFFFAOYSA-N | CCCCCCCCCCCOC(=O)CCC(=O)OCC(Cl)(Cl)Cl | 0 | 0 | 2 | 2 |
| Propanoic acid, 3-chloro, octadecyl ester (C21H41ClO2) | IWNDNJGFFCNPKN-UHFFFAOYSA-N | CCCCCCCCCCCCCCCCCCOC(=O)CCCl | 0 | 180 | 2 | 1 |
| 5-Chlorovaleric acid, hexadecyl ester (C21H41ClO2) | HJPJQMNZOQVLCN-UHFFFAOYSA-N | CCCCCCCCCCCCCCCCOC(=O)CCCCCl | 0 | 120 | 2 | 1 |
| Succinic acid, 10-chlorodecyl pentyl ester (C19H35ClO4) | JSJJXPMDQXRCDP-UHFFFAOYSA-N | CCCCCOC(=O)CCC(=O)OCCCCCCCCCCCl | 0 | 40 | 2 | 2 |
| Succinic acid, 8-chloroctyl heptyl ester (C19H35ClO4) | KOXUVSWOTSRYLK-UHFFFAOYSA-N | CCCCCCCOC(=O)CCC(=O)OCCCCCCCCCl | 0 | 20 | 2 | 2 |
| Adipic acid, 8-chloroctyl pentyl ester (C19H35ClO4) | IIDRUMFLPGVZJE-UHFFFAOYSA-N | CCCCCOC(=O)CCCCC(=O)OCCCCCCCCCl | 0 | 30 | 2 | 2 |
| Propanoic acid, 2-chloro, octadecyl ester (C21H41ClO2) | YQCZLISEEYFBDQ-UHFFFAOYSA-N | CCCCCCCCCCCCCCCCCCOC(=O)C(C)Cl | 30 | 0 | 2 | 2 |
| 5-chlorovaleric acid, 4-hexadecyl ester (C21H41ClO2) | XTQZSUCQERUSLW-UHFFFAOYSA-N | CCCCCCCCCCCCC(CCC)OC(=O)CCCCCl | 0 | 0 | 2 | 2 |
| Propanamide, N,N-dinonyl-3-chloro- (C21H42ClNO) | IXOQRAVVQUMNPF-UHFFFAOYSA-N | CCCCCCCCCN(CCCCCCCCC)C(=O)CCCl | 50 | 5375 | 2 | 1 |
| Malonic acid, 2-chloropropyl tridecyl ester (C19H35ClO4) | XQULABOIZJGPFB-UHFFFAOYSA-N | CCCCCCCCCCCCCOC(=O)CC(=O)OCC(C)Cl | 0 | 0 | 2 | 2 |
| Succinic acid, 2-chloropropyl dodecyl ester (C19H35ClO4) | KOLBIKZQRXLBCH-UHFFFAOYSA-N | CCCCCCCCCCCCOC(=O)CCC(=O)OCC(C)Cl | 0 | 0 | 2 | 2 |
| Propanamide, N,N-dinonyl-2-chloro- (C21H42ClNO) | CAYVAIPTSFKUIT-UHFFFAOYSA-N | CCCCCCCCCN(CCCCCCCCC)C(=O)C(C)Cl | 200 | 9248 | 2 | 1 |
| Succinic acid, 2,2-dichloroethyl dodecyl ester (C18H32Cl2O4) | XPSAWCRUDRNORV-UHFFFAOYSA-N | CCCCCCCCCCCCOC(=O)CCC(=O)OCC(Cl)Cl | 0 | 0 | 2 | 2 |
| Dichloroacetamide, N,N-dinonyl- (C20H39Cl2NO) | HRQKKFLEURNPRM-UHFFFAOYSA-N | CCCCCCCCCN(CCCCCCCCC)C(=O)C(Cl)Cl | 0 | 3153 | 2 | 1 |
| Fumaric acid, 2,2,2-trichloroethyl undecyl ester (C17H27Cl3O4) | QNUKHDVSHBDXON-VAWYXSNFSA-N | CCCCCCCCCCCOC(=O)/C=C/C(=O)OCC(Cl)(Cl)Cl | 0 | 971 | 2 | 1 |
| Fumaric acid, 2,2-dichloroethyl dodecyl ester (C18H30Cl2O4) | IDWSWXWSQPMUQF-OUKQBFOZSA-N | CCCCCCCCCCCCOC(=O)/C=C/C(=O)OCC(Cl)Cl | 0 | 220 | 2 | 1 |
| Fumaric acid, 10-chlorodecyl pentyl ester (C19H33ClO4) | AHHWRJVIUFWBFV-BUHFOSPRSA-N | CCCCCOC(=O)/C=C/C(=O)OCCCCCCCCCCCl | 0 | 20 | 2 | 2 |
| Fumaric acid, 8-chlorooctyl heptyl ester (C19H33ClO4) | FBIIAROAPWHMQC-BUHFOSPRSA-N | CCCCCCCOC(=O)/C=C/C(=O)OCCCCCCCCCl | 0 | 30 | 2 | 2 |
| Fumaric acid, 2-chloropropyl dodecyl ester (C19H33ClO4) | KBMFXTYTNXLVHL-BUHFOSPRSA-N | CCCCCCCCCCCCOC(=O)/C=C/C(=O)OCC(C)Cl | 40 | 110 | 2 | 1 |
| Succinic acid, hexyl 2,3,4,6-tetrachlorophenyl ester (C16H18Cl4O4) | UWAQLHSZIULLMW-UHFFFAOYSA-N | CCCCCCOC(=O)CCC(=O)OC1=C(C(=C(C=C1Cl)Cl)Cl)Cl | 0 | 0 | 2 | 2 |
| Succinic acid, isohexyl 2,3,4,6-tetrachlorophenyl ester (C16H18Cl4O4) | OKWVSPIPIFLQOU-UHFFFAOYSA-N | CC(C)CCCOC(=O)CCC(=O)OC1=C(C(=C(C=C1Cl)Cl)Cl)Cl | 0 | 0 | 2 | 2 |
| Succinic acid, heptyl 2,3,5-trichlorophenyl ester (C17H21Cl3O4) | XOIGLVMSEUBMHM-UHFFFAOYSA-N | CCCCCCCOC(=O)CCC(=O)OC1=CC(=CC(=C1Cl)Cl)Cl | 0 | 0 | 2 | 2 |
| Succinic acid, heptyl 2,3,6-trichlorophenyl ester (C17H21Cl3O4) | HCCNIZQNNWYAGN-UHFFFAOYSA-N | CCCCCCCOC(=O)CCC(=O)OC1=C(C=CC(=C1Cl)Cl)Cl | 0 | 0 | 2 | 2 |
| Sarcosine, N-(2-chlorobenzoyl)-, nonyl ester (C19H28ClNO3) | KLARXPGAVTVVDQ-UHFFFAOYSA-N | CCCCCCCCCOC(=O)CN(C)C(=O)C1=CC=CC=C1Cl | 1421 | 110 | 1 | 1 |
| Sarcosine, N-(4-chlorobenzoyl)-, nonyl ester (C19H28ClNO3) | VNSOENDCYXSYBU-UHFFFAOYSA-N | CCCCCCCCCOC(=O)CN(C)C(=O)C1=CC=C(C=C1)Cl | 1231 | 0 | 1 | 2 |
| Succinic acid, 3,5-dichlorophenyl octyl ester (C18H24Cl2O4) | OXDMKGDASFYIQI-UHFFFAOYSA-N | CCCCCCCCOC(=O)CCC(=O)OC1=CC(=CC(=C1)Cl)Cl | 0 | 0 | 2 | 2 |
| β-Alanine, N-(2-chlorobenzoyl)-, nonyl ester (C19H28ClNO3) | YFRBSXGZLPUOQN-UHFFFAOYSA-N | CCCCCCCCCOC(=O)CCNC(=O)C1=CC=CC=C1Cl | 901 | 80 | 1 | 2 |
| β-Alanine, N-(4-chlorobenzoyl)-, nonyl ester (C19H28ClNO3) | LZFACYYJLOZBFV-UHFFFAOYSA-N | CCCCCCCCCOC(=O)CCNC(=O)C1=CC=C(C=C1)Cl | 591 | 50 | 2 | 2 |
| Benzamide, N,N-diheptyl-4-chloro- (C21H34ClNO) | WLIIKSWZVDTDSF-UHFFFAOYSA-N | CCCCCCCN(CCCCCCC)C(=O)C1=CC=C(C=C1)Cl | 691 | 0 | 2 | 2 |
| 2-Chlorobenzoic acid, tetradecyl ester (C21H33ClO2) | NATDFLAPWQOQKI-UHFFFAOYSA-N | CCCCCCCCCCCCCCOC(=O)C1=CC=CC=C1Cl | 50 | 0 | 2 | 2 |
| 2-Chlorobenzoic acid, 2-tetradecyl ester (C21H33ClO2) | BPLSFDFFHWQJEC-UHFFFAOYSA-N | CCCCCCCCCCCCC(C)OC(=O)C1=CC=CC=C1Cl | 20 | 0 | 2 | 2 |
| 2-Chlorobenzoic acid, 3-tetradecyl ester (C21H33ClO2) | XVAHMORCDPFYJM-UHFFFAOYSA-N | CCCCCCCCCCCC(CC)OC(=O)C1=CC=CC=C1Cl | 0 | 0 | 2 | 2 |
| 2-Chlorobenzoic acid, 4-tetradecyl ester (C21H33ClO2) | YCRAQMYJTQYHQF-UHFFFAOYSA-N | CCCCCCCCCCC(CCC)OC(=O)C1=CC=CC=C1Cl | 0 | 0 | 2 | 2 |
| 2-Chlorobenzoic acid, 5-tetradecyl ester (C21H33ClO2) | HBSHCUYTTUNVEU-UHFFFAOYSA-N | CCCCCCCCCC(CCCC)OC(=O)C1=CC=CC=C1Cl | 0 | 0 | 2 | 2 |
| Fumaric acid, pentachlorophenyl pentyl ester (C15H13Cl5O4) | VLYJINNDRWZADO-AATRIKPKSA-N | CCCCCOC(=O)/C=C/C(=O)OC1=C(C(=C(C(=C1Cl)Cl)Cl)Cl)Cl | 20 | 0 | 2 | 2 |
| Fumaric acid, hexyl 2,3,4,6-tetrachlorophenyl ester (C16H16Cl4O4) | CAUPBJYBDQEJAL-VOTSOKGWSA-N | CCCCCCOC(=O)/C=C/C(=O)OC1=C(C(=C(C=C1Cl)Cl)Cl)Cl | 20 | 0 | 2 | 2 |
| Fumaric acid, hexyl 2,3,4,5-tetrachlorophenyl ester (C16H16Cl4O4) | DRNXHSLSDLPUCA-VOTSOKGWSA-N | CCCCCCOC(=O)/C=C/C(=O)OC1=CC(=C(C(=C1Cl)Cl)Cl)Cl | 0 | 0 | 2 | 2 |
| Fumaric acid, hexyl 2,3,5,6-tetrachlorophenyl ester (C16H16Cl4O4) | OFPNLEPZXPVEHX-VOTSOKGWSA-N | CCCCCCOC(=O)/C=C/C(=O)OC1=C(C(=CC(=C1Cl)Cl)Cl)Cl | 30 | 0 | 2 | 2 |
| Fumaric acid, isohexyl 2,3,4,6-tetrachlorophenyl ester (C16H16Cl4O4) | GIRQPJZMYSGNMT-AATRIKPKSA-N | CC(C)CCCOC(=O)/C=C/C(=O)OC1=C(C(=C(C=C1Cl)Cl)Cl)Cl | 30 | 0 | 2 | 2 |
| Fumaric acid, isohexyl 2,3,4,5-tetrachlorophenyl ester (C16H16Cl4O4) | ZIJTYAMLAPSFSL-AATRIKPKSA-N | CC(C)CCCOC(=O)/C=C/C(=O)OC1=CC(=C(C(=C1Cl)Cl)Cl)Cl | 0 | 0 | 2 | 2 |
| Fumaric acid, isohexyl 2,3,5,6-tetrachlorophenyl ester (C16H16Cl4O4) | ISIZDRAVGGKUKL-AATRIKPKSA-N | CC(C)CCCOC(=O)/C=C/C(=O)OC1=C(C(=CC(=C1Cl)Cl)Cl)Cl | 50 | 0 | 2 | 2 |
| Fumaric acid, heptyl 2,4,5-trichlorophenyl ester (C17H19Cl3O4) | IIUMMUYUNAGBGY-BQYQJAHWSA-N | CCCCCCCOC(=O)/C=C/C(=O)OC1=CC(=C(C=C1Cl)Cl)Cl | 0 | 0 | 2 | 2 |
| Fumaric acid, heptyl 2,3,5-trichlorophenyl ester (C17H19Cl3O4) | APWDLSBWGYTNIR-BQYQJAHWSA-N | CCCCCCCOC(=O)/C=C/C(=O)OC1=CC(=CC(=C1Cl)Cl)Cl | 30 | 0 | 2 | 2 |
| Fumaric acid, heptyl 2,3,6-trichlorophenyl ester (C17H19Cl3O4) | DUQKLYISVMGEDJ-MDZDMXLPSA-N | CCCCCCCOC(=O)/C=C/C(=O)OC1=C(C=CC(=C1Cl)Cl)Cl | 90 | 0 | 2 | 2 |
| Fumaric acid, heptyl 2,4,6-trichlorophenyl ester (C17H19Cl3O4) | OGEQKOYZNQRTMW-BQYQJAHWSA-N | CCCCCCCOC(=O)/C=C/C(=O)OC1=C(C=C(C=C1Cl)Cl)Cl | 20 | 0 | 2 | 2 |
| Succinic acid, di(3,5-dichlorophenyl) ester (C16H10Cl4O4) | VCIGYAXEHAIUNO-UHFFFAOYSA-N | C1=C(C=C(C=C1Cl)Cl)OC(=O)CCC(=O)OC2=CC(=CC(=C2)Cl)Cl | 0 | 0 | 2 | 2 |
| Benzoylprop ethyl (C18H17Cl2NO3) | SLCGUGMPSUYJAY-UHFFFAOYSA-N | CCOC(=O)C(C)N(C1=CC(=C(C=C1)Cl)Cl)C(=O)C2=CC=CC=C2 | 210 | 0 | 2 | 2 |
| Mefenpyr-diethyl (C16H18Cl2N2O4) | OPGCOAPTHCZZIW-UHFFFAOYSA-N | CCOC(=O)C1=NN(C(C1)(C)C(=O)OCC)C2=C(C=C(C=C2)Cl)Cl | 2422 | 0 | 1 | 2 |
| Fumaric acid, di(3,5-dichlorophenyl) ester (C16H8Cl4O4) | ZFRLJURZYHEHAA-OWOJBTEDSA-N | C1=C(C=C(C=C1Cl)Cl)OC(=O)/C=C/C(=O)OC2=CC(=CC(=C2)Cl)Cl | 240 | 0 | 2 | 2 |
| Fumaric acid, di(2-chloro-5-methylphenyl) ester (C18H14Cl2O4) | IPULWJRXVZFERK-BQYQJAHWSA-N | CC1=CC(=C(C=C1)Cl)OC(=O)/C=C/C(=O)OC2=C(C=CC(=C2)C)Cl | 480 | 80 | 2 | 2 |
| Pyridaben (C19H25ClN2OS) | DWFZBUWUXWZWKD-UHFFFAOYSA-N | CC(C)(C)C1=CC=C(C=C1)CSC2=C(C(=O)N(N=C2)C(C)(C)C)Cl | 540 | 0 | 2 | 2 |
| Furosemide, trimethyl (C15H17ClN2O5S) | OBJFIAVSHHYZPK-UHFFFAOYSA-N | CNS(=O)(=O)C1=C(C=C(C(=C1)C(=O)OC)N(C)CC2=CC=CO2)Cl | 1301 | 0 | 1 | 2 |
| 2-Chlorobenzoic acid, 4-benzyloxyphenyl ester (C20H15ClO3) | UQSRIVDYEKEWHD-UHFFFAOYSA-N | C1=CC=C(C=C1)COC2=CC=C(C=C2)OC(=O)C3=CC=CC=C3Cl | 2722 | 0 | 1 | 2 |
| Metolazone (C16H16ClN3O3S) | AQCHWTWZEMGIFD-UHFFFAOYSA-N | CC1NC2=CC(=C(C=C2C(=O)N1C3=CC=CC=C3C)S(=O)(=O)N)Cl | 780 | 0 | 2 | 2 |
| Clemastine (C21H26ClNO) | YNNUSGIPVFPVBX-UHFFFAOYSA-N | CC(C1=CC=CC=C1)(C2=CC=C(C=C2)Cl)OCCC3CCCN3C | 0 | 0 | 2 | 2 |
| Coumachlor (C19H15ClO4) | DEKWZWCFHUABHE-UHFFFAOYSA-N | CC(=O)CC(C1=CC=C(C=C1)Cl)C2=C(C3=CC=CC=C3OC2=O)O | 2739 | 0 | 1 | 2 |
| Griseofulvin (C17H17ClO6) | DDUHZTYCFQRHIY-UHFFFAOYSA-N | CC1CC(=O)C=C(C12C(=O)C3=C(O2)C(=C(C=C3OC)OC)Cl)OC | 9999 | 179 | 1 | 1 |
| Cyclothiazide (C14H16ClN3O4S2) | BOCUKUHCLICSIY-UHFFFAOYSA-N | C1C2CC(C1C=C2)C3NC4=CC(=C(C=C4S(=O)(=O)N3)S(=O)(=O)N)Cl | 500 | 0 | 2 | 2 |
| 5-chlorovaleric acid, heptadecyl ester (C22H43ClO2) | DBESYZFSKKRNPR-UHFFFAOYSA-N | CCCCCCCCCCCCCCCCCOC(=O)CCCCCl | 0 | 100 | 2 | 2 |
| Succinic acid, 10-chlorodecyl hexyl ester (C20H37ClO4) | GZBIUVHJGBCYNE-UHFFFAOYSA-N | CCCCCCOC(=O)CCC(=O)OCCCCCCCCCCCl | 0 | 40 | 2 | 2 |
| Adipic acid, 8-chloroctyl hexyl ester (C20H37ClO4) | QOKXDWWCPGIUMU-UHFFFAOYSA-N | CCCCCCOC(=O)CCCCC(=O)OCCCCCCCCCl | 0 | 40 | 2 | 2 |
| Chloroacetamide, N,N-didecyl- (C22H44ClNO) | YPGJDQWTBAWREX-UHFFFAOYSA-N | CCCCCCCCCCN(CCCCCCCCCC)C(=O)CCl | 40 | 4804 | 2 | 1 |
| Hexanamide, N,N-dioctyl-6-chloro- (C22H44ClNO) | YCAKAOFECFRADB-UHFFFAOYSA-N | CCCCCCCCN(CCCCCCCC)C(=O)CCCCCCl | 40 | 480 | 2 | 1 |
| Succinic acid, 10-chlorodecyl isohexyl ester (C20H37ClO4) | BZKVKBXJIOJRKG-UHFFFAOYSA-N | CC(C)CCCOC(=O)CCC(=O)OCCCCCCCCCCCl | 0 | 30 | 2 | 2 |
| Adipic acid, 8-chloroctyl isohexyl ester (C20H37ClO4) | FMZUTOFLYCLHDY-UHFFFAOYSA-N | CC(C)CCCOC(=O)CCCCC(=O)OCCCCCCCCCl | 0 | 40 | 2 | 2 |
| Hexanamide, N,N-bis(2-ethylhexyl)-6-chloro- (C22H44ClNO) | QIAXMLYRWAMPLX-UHFFFAOYSA-N | CCCCC(CC)CN(CC(CC)CCCC)C(=O)CCCCCCl | 0 | 40 | 2 | 2 |
| Malonic acid, di(8-chlorooctyl) ester (C19H34Cl2O4) | XPFJQQXPWSKDES-UHFFFAOYSA-N | C(CCCCCl)CCCOC(=O)CC(=O)OCCCCCCCCCl | 0 | 0 | 2 | 2 |
| Succinic acid, 2,2-dichloroethyl tridecyl ester (C19H34Cl2O4) | YNGSYZCAMGOLIM-UHFFFAOYSA-N | CCCCCCCCCCCCCOC(=O)CCC(=O)OCC(Cl)Cl | 0 | 0 | 2 | 2 |
| Succinic acid, dodecyl 2,2,2-trichloroethyl ester (C18H31Cl3O4) | BBLDVAZHTUMKSA-UHFFFAOYSA-N | CCCCCCCCCCCCOC(=O)CCC(=O)OCC(Cl)(Cl)Cl | 0 | 0 | 2 | 2 |
| Fumaric acid, dodecyl 2,2,2-trichloroethyl ester (C18H29Cl3O4) | IFPVIGYQBLXYMK-OUKQBFOZSA-N | CCCCCCCCCCCCOC(=O)/C=C/C(=O)OCC(Cl)(Cl)Cl | 0 | 1081 | 2 | 1 |
| Fumaric acid, 10-chlorodecyl hexyl ester (C20H35ClO4) | LZFDJKVRRLCZMI-CCEZHUSRSA-N | CCCCCCOC(=O)/C=C/C(=O)OCCCCCCCCCCCl | 0 | 30 | 2 | 2 |
| Fumaric acid, 8-chlorooctyl octyl ester (C20H35ClO4) | RLEXIXXJESQPHT-CCEZHUSRSA-N | CCCCCCCCOC(=O)/C=C/C(=O)OCCCCCCCCCl | 0 | 20 | 2 | 2 |
| Fumaric acid, 10-chlorodecyl isohexyl ester (C20H35ClO4) | ITEOWUDKTFDNPT-BUHFOSPRSA-N | CC(C)CCCOC(=O)/C=C/C(=O)OCCCCCCCCCCCl | 0 | 0 | 2 | 2 |
| Fumaric acid, 2-chloropropyl tridecyl ester (C20H35ClO4) | QLRKWJUGPGGUET-CCEZHUSRSA-N | CCCCCCCCCCCCCOC(=O)/C=C/C(=O)OCC(C)Cl | 40 | 70 | 2 | 2 |
| Fumaric acid, 2,2-dichloroethyl tridecyl ester (C19H32Cl2O4) | XEUKCOQEWZJXBR-BUHFOSPRSA-N | CCCCCCCCCCCCCOC(=O)/C=C/C(=O)OCC(Cl)Cl | 0 | 200 | 2 | 1 |
| Succinic acid, heptyl 2,3,4,6-tetrachlorophenyl ester (C17H20Cl4O4) | PNUZRGUWPVKHQH-UHFFFAOYSA-N | CCCCCCCOC(=O)CCC(=O)OC1=C(C(=C(C=C1Cl)Cl)Cl)Cl | 0 | 0 | 2 | 2 |
| Succinic acid, octyl 2,3,5-trichlorophenyl ester (C18H23Cl3O4) | ZRSCNPMSOLQJDU-UHFFFAOYSA-N | CCCCCCCCOC(=O)CCC(=O)OC1=CC(=CC(=C1Cl)Cl)Cl | 0 | 0 | 2 | 2 |
| Succinic acid, octyl 2,3,6-trichlorophenyl ester (C18H23Cl3O4) | XEYNTLMQFHCOLE-UHFFFAOYSA-N | CCCCCCCCOC(=O)CCC(=O)OC1=C(C=CC(=C1Cl)Cl)Cl | 0 | 0 | 2 | 2 |
| Sarcosine, N-(2-chlorobenzoyl)-, decyl ester (C20H30ClNO3) | ZNCTXOPPEHUQQW-UHFFFAOYSA-N | CCCCCCCCCCOC(=O)CN(C)C(=O)C1=CC=CC=C1Cl | 1441 | 120 | 1 | 1 |
| Sarcosine, N-(4-chlorobenzoyl)-, decyl ester (C20H30ClNO3) | UPRSSGWYPBBRLK-UHFFFAOYSA-N | CCCCCCCCCCOC(=O)CN(C)C(=O)C1=CC=C(C=C1)Cl | 1021 | 0 | 1 | 2 |
| Succinic acid, 3,5-dichlorophenyl nonyl ester (C19H26Cl2O4) | DNKFFCYLMKTWGF-UHFFFAOYSA-N | CCCCCCCCCOC(=O)CCC(=O)OC1=CC(=CC(=C1)Cl)Cl | 0 | 0 | 2 | 2 |
| β-Alanine, N-(2-chlorobenzoyl)-, decyl ester (C20H30ClNO3) | QYWPBIFYAZPZMP-UHFFFAOYSA-N | CCCCCCCCCCOC(=O)CCNC(=O)C1=CC=CC=C1Cl | 841 | 70 | 1 | 2 |
| β-Alanine, N-(4-chlorobenzoyl)-, decyl ester (C20H30ClNO3) | YCYJYQKVEYADGA-UHFFFAOYSA-N | CCCCCCCCCCOC(=O)CCNC(=O)C1=CC=C(C=C1)Cl | 631 | 100 | 2 | 2 |
| Benzamide, N-heptyl-N-octyl-4-chloro- (C22H36ClNO) | YNGWSACUMIXQBU-UHFFFAOYSA-N | CCCCCCCCN(CCCCCCC)C(=O)C1=CC=C(C=C1)Cl | 470 | 0 | 2 | 2 |
| 2-Chlorobenzoic acid, pentadecyl ester (C22H35ClO2) | KHLHBSPALROSMG-UHFFFAOYSA-N | CCCCCCCCCCCCCCCOC(=O)C1=CC=CC=C1Cl | 40 | 0 | 2 | 2 |
| 2-Chlorobenzoic acid, 2-pentadecyl ester (C22H35ClO2) | ACBTZZPWYCGTSU-UHFFFAOYSA-N | CCCCCCCCCCCCCC(C)OC(=O)C1=CC=CC=C1Cl | 0 | 0 | 2 | 2 |
| 2-Chlorobenzoic acid, 3-pentadecyl ester (C22H35ClO2) | LIPZCBXPWHSJGI-UHFFFAOYSA-N | CCCCCCCCCCCCC(CC)OC(=O)C1=CC=CC=C1Cl | 0 | 0 | 2 | 2 |
| 2-Chlorobenzoic acid, 4-pentadecyl ester (C22H35ClO2) | LFJPOYLNCYQVMI-UHFFFAOYSA-N | CCCCCCCCCCCC(CCC)OC(=O)C1=CC=CC=C1Cl | 20 | 0 | 2 | 2 |
| 2-Chlorobenzoic acid, 5-pentadecyl ester (C22H35ClO2) | ONZWJIRDMUYGJF-UHFFFAOYSA-N | CCCCCCCCCCC(CCCC)OC(=O)C1=CC=CC=C1Cl | 20 | 0 | 2 | 2 |
| 2-Chlorobenzoic acid, 6-pentadecyl ester (C22H35ClO2) | LAECTIQLWWUWOK-UHFFFAOYSA-N | CCCCCCCCCC(CCCCC)OC(=O)C1=CC=CC=C1Cl | 0 | 0 | 2 | 2 |
| 2-Chlorobenzoic acid, 7-pentadecyl ester (C22H35ClO2) | JATVRVHMGHMRDP-UHFFFAOYSA-N | CCCCCCCCC(CCCCCC)OC(=O)C1=CC=CC=C1Cl | 0 | 0 | 2 | 2 |
| 2-Chlorobenzoic acid, 8-pentadecyl ester (C22H35ClO2) | NFAWPWNROYXQCJ-UHFFFAOYSA-N | CCCCCCCC(CCCCCCC)OC(=O)C1=CC=CC=C1Cl | 0 | 0 | 2 | 2 |
| Fumaric acid, hexyl pentachlorophenyl ester (C16H15Cl5O4) | HIPBUHXRIHIIST-VOTSOKGWSA-N | CCCCCCOC(=O)/C=C/C(=O)OC1=C(C(=C(C(=C1Cl)Cl)Cl)Cl)Cl | 0 | 0 | 2 | 2 |
| Fumaric acid, isohexyl pentachlorophenyl ester (C16H15Cl5O4) | BYHQDWGIFGHXMT-AATRIKPKSA-N | CC(C)CCCOC(=O)/C=C/C(=O)OC1=C(C(=C(C(=C1Cl)Cl)Cl)Cl)Cl | 0 | 0 | 2 | 2 |
| Fumaric acid, heptyl 2,3,4,6-tetrachlorophenyl ester (C17H18Cl4O4) | GOGVZHKCOHWPGO-BQYQJAHWSA-N | CCCCCCCOC(=O)/C=C/C(=O)OC1=C(C(=C(C=C1Cl)Cl)Cl)Cl | 20 | 0 | 2 | 2 |
| Fumaric acid, heptyl 2,3,5,6-tetrachlorophenyl ester (C17H18Cl4O4) | YUUNIIFPLKPIJX-BQYQJAHWSA-N | CCCCCCCOC(=O)/C=C/C(=O)OC1=C(C(=CC(=C1Cl)Cl)Cl)Cl | 20 | 0 | 2 | 2 |
| Fumaric acid, octyl 2,3,5-trichlorophenyl ester (C18H21Cl3O4) | JJPOXJAPPIWZEW-CMDGGOBGSA-N | CCCCCCCCOC(=O)/C=C/C(=O)OC1=CC(=CC(=C1Cl)Cl)Cl | 40 | 0 | 2 | 2 |
| Fumaric acid, octyl 2,3,6-trichlorophenyl ester (C18H21Cl3O4) | IRUIEEPNBKTVAT-ZHACJKMWSA-N | CCCCCCCCOC(=O)/C=C/C(=O)OC1=C(C=CC(=C1Cl)Cl)Cl | 90 | 0 | 2 | 2 |
| Fumaric acid, octyl 2,4,6-trichlorophenyl ester (C18H21Cl3O4) | CEIGQFJBTNZSFB-CMDGGOBGSA-N | CCCCCCCCOC(=O)/C=C/C(=O)OC1=C(C=C(C=C1Cl)Cl)Cl | 20 | 0 | 2 | 2 |
| Fumaric acid, 2-chloro-5-methylphenyl nonyl ester (C20H27ClO4) | FGYSIMUZJAJGCJ-OUKQBFOZSA-N | CCCCCCCCCOC(=O)/C=C/C(=O)OC1=C(C=CC(=C1)C)Cl | 310 | 280 | 2 | 1 |
| Benzoximate (C18H18ClNO5) | BZMIHNKNQJJVRO-UHFFFAOYSA-N | CCON=C(C1=C(C=CC(=C1OC)Cl)OC)OC(=O)C2=CC=CC=C2 | 0 | 0 | 2 | 2 |
| pyridate (C19H23ClN2O2S) | JTZCTMAVMHRNTR-UHFFFAOYSA-N | CCCCCCCCSC(=O)OC1=CC(=NN=C1C2=CC=CC=C2)Cl | 140 | 60 | 2 | 2 |
| Clofoctol, methyl ether (C22H28Cl2O) | WCOGVAKQAAUJCO-UHFFFAOYSA-N | CC(C)(C)CC(C)(C)C1=CC(=C(C=C1)OC)CC2=C(C=C(C=C2)Cl)Cl | 440 | 0 | 2 | 2 |
| Felodipine (C18H19Cl2NO4) | RZTAMFZIAATZDJ-UHFFFAOYSA-N | CCOC(=O)C1=C(NC(=C(C1C2=C(C(=CC=C2)Cl)Cl)C(=O)OC)C)C | 240 | 80 | 2 | 2 |
| 2-Naphthalenecarboxamide, N-(5-chloro-2,4-dimethoxyphenyl)-3-hydroxy- (C19H16ClNO4) | XDWATWCCUTYUDE-UHFFFAOYSA-N | COC1=CC(=C(C=C1NC(=O)C2=CC3=CC=CC=C3C=C2O)Cl)OC | 727 | 0 | 2 | 2 |
| 2-Chloro-1,4-dibenzamido benzene (C20H15ClN2O2) | AUCDGWBCUYSRAV-UHFFFAOYSA-N | C1=CC=C(C=C1)C(=O)NC2=CC(=C(C=C2)NC(=O)C3=CC=CC=C3)Cl | 1383 | 1105 | 1 | 1 |
| Amodiaquine (C20H22ClN3O) | OVCDSSHSILBFBN-UHFFFAOYSA-N | CCN(CC)CC1=C(C=CC(=C1)NC2=C3C=CC(=CC3=NC=C2)Cl)O | 3203 | 0 | 1 | 2 |
| Temazepam propanoate (C19H17ClN2O3) | XLLIZHAJTGQVQR-UHFFFAOYSA-N | CCC(=O)OC1C(=O)N(C2=C(C=C(C=C2)Cl)C(=N1)C3=CC=CC=C3)C | 470 | 0 | 2 | 2 |
| Miconazole (C18H14Cl4N2O) | BYBLEWFAAKGYCD-UHFFFAOYSA-N | C1=CC(=C(C=C1Cl)Cl)COC(CN2C=CN=C2)C3=C(C=C(C=C3)Cl)Cl | 110 | 0 | 2 | 2 |
| Indomethacin (C19H16ClNO4) | CGIGDMFJXJATDK-UHFFFAOYSA-N | CC1=C(C2=C(N1C(=O)C3=CC=C(C=C3)Cl)C=CC(=C2)OC)CC(=O)O | 2600 | 0 | 1 | 2 |
| Cyclohexanone, 2-(3-chloro-2-butenyl)-2-methyl-6,6-diphenyl- (C23H25ClO) | TWBAHMRTJVSXOP-NBVRZTHBSA-N | C/C(=C\CC1(CCCC(C1=O)(C2=CC=CC=C2)C3=CC=CC=C3)C)/Cl | 3410 | 0 | 1 | 2 |
| Prochlorperazine (C20H24ClN3S) | WIKYUJGCLQQFNW-UHFFFAOYSA-N | CN1CCN(CC1)CCCN2C3=CC=CC=C3SC4=C2C=C(C=C4)Cl | 3930 | 90 | 1 | 2 |
| 5-Chlorovaleric acid, octadecyl ester (C23H45ClO2) | NIFZBQWHRAITQZ-UHFFFAOYSA-N | CCCCCCCCCCCCCCCCCCOC(=O)CCCCCl | 30 | 90 | 2 | 2 |
| Succinic acid, 10-chlorodecyl heptyl ester (C21H39ClO4) | LWABOUYGQGFCAR-UHFFFAOYSA-N | CCCCCCCOC(=O)CCC(=O)OCCCCCCCCCCCl | 0 | 40 | 2 | 2 |
| Succinic acid, 8-chloroctyl nonyl ester (C21H39ClO4) | KGENSJOMPYRJIV-UHFFFAOYSA-N | CCCCCCCCCOC(=O)CCC(=O)OCCCCCCCCCl | 0 | 40 | 2 | 2 |
| Adipic acid, 8-chloroctyl heptyl ester (C21H39ClO4) | MARYDTQHJDPBRX-UHFFFAOYSA-N | CCCCCCCOC(=O)CCCCC(=O)OCCCCCCCCCl | 0 | 40 | 2 | 2 |
| Propanamide, N,N-didecyl-3-chloro- (C23H46ClNO) | GLHLCNPQGMVKTL-UHFFFAOYSA-N | CCCCCCCCCCN(CCCCCCCCCC)C(=O)CCCl | 110 | 9999 | 2 | 1 |
| Malonic acid, 2-chloropropyl pentadecyl ester (C21H39ClO4) | DQWVDNYIQVEEMR-UHFFFAOYSA-N | CCCCCCCCCCCCCCCOC(=O)CC(=O)OCC(C)Cl | 0 | 0 | 2 | 2 |
| Propanamide, N,N-didecyl-2-chloro- (C23H46ClNO) | RSNTYEXEJHDLHX-UHFFFAOYSA-N | CCCCCCCCCCN(CCCCCCCCCC)C(=O)C(C)Cl | 190 | 9769 | 2 | 1 |
| Succinic acid, di(8-chloroctyl) ester (C20H36Cl2O4) | WSNMNGSFXRGKBL-UHFFFAOYSA-N | C(CCCCCl)CCCOC(=O)CCC(=O)OCCCCCCCCCl | 0 | 50 | 2 | 2 |
| Succinic acid, 2,2-dichloroethyl tetradecyl ester (C20H36Cl2O4) | NFGHDBZFEYGCGM-UHFFFAOYSA-N | CCCCCCCCCCCCCCOC(=O)CCC(=O)OCC(Cl)Cl | 0 | 0 | 2 | 2 |
| Dichloroacetamide, N,N-didecyl- (C22H43Cl2NO) | FJRVDNKXGHJTHQ-UHFFFAOYSA-N | CCCCCCCCCCN(CCCCCCCCCC)C(=O)C(Cl)Cl | 0 | 3513 | 2 | 1 |
| Succinic acid, 2,2,2-trichloroethyl tridecyl ester (C19H33Cl3O4) | GSNGKIWQLUMYKR-UHFFFAOYSA-N | CCCCCCCCCCCCCOC(=O)CCC(=O)OCC(Cl)(Cl)Cl | 0 | 0 | 2 | 2 |
| Fumaric acid, 10-chlorodecyl heptyl ester (C21H37ClO4) | ZZURKLXZZZMMGT-FOCLMDBBSA-N | CCCCCCCOC(=O)/C=C/C(=O)OCCCCCCCCCCCl | 0 | 20 | 2 | 2 |
| Fumaric acid, 8-chlorooctyl nonyl ester (C21H37ClO4) | WKCQJAPPHZEXMS-FOCLMDBBSA-N | CCCCCCCCCOC(=O)/C=C/C(=O)OCCCCCCCCCl | 0 | 0 | 2 | 2 |
| Fumaric acid, di(8-chlorooctyl) ester (C20H34Cl2O4) | QMQZGFHUZRFAIY-BUHFOSPRSA-N | C(CCCCCl)CCCOC(=O)/C=C/C(=O)OCCCCCCCCCl | 0 | 40 | 2 | 2 |
| Fumaric acid, 2,2,2-trichloroethyl tridecyl ester (C19H31Cl3O4) | ZTXCYTUNNUJFBB-BUHFOSPRSA-N | CCCCCCCCCCCCCOC(=O)/C=C/C(=O)OCC(Cl)(Cl)Cl | 0 | 1742 | 2 | 1 |
| Succinic acid, octyl 2,3,4,6-tetrachlorophenyl ester (C18H22Cl4O4) | ZPBDZIQNGPKYBE-UHFFFAOYSA-N | CCCCCCCCOC(=O)CCC(=O)OC1=C(C(=C(C=C1Cl)Cl)Cl)Cl | 0 | 0 | 2 | 2 |
| Succinic acid, nonyl 2,3,5-trichlorophenyl ester (C19H25Cl3O4) | XNLYKNBTHYMUIQ-UHFFFAOYSA-N | CCCCCCCCCOC(=O)CCC(=O)OC1=CC(=CC(=C1Cl)Cl)Cl | 0 | 0 | 2 | 2 |
| Succinic acid, nonyl 2,3,6-trichlorophenyl ester (C19H25Cl3O4) | DMLVDCHTUFDYJD-UHFFFAOYSA-N | CCCCCCCCCOC(=O)CCC(=O)OC1=C(C=CC(=C1Cl)Cl)Cl | 0 | 0 | 2 | 2 |
| Sarcosine, N-(4-chlorobenzoyl)-, undecyl ester (C21H32ClNO3) | GJYSOEJLPGTJJA-UHFFFAOYSA-N | CCCCCCCCCCCOC(=O)CN(C)C(=O)C1=CC=C(C=C1)Cl | 1111 | 0 | 1 | 2 |
| Succinic acid, decyl 3,5-dichlorophenyl ester (C20H28Cl2O4) | HHAIGROYTVHYQS-UHFFFAOYSA-N | CCCCCCCCCCOC(=O)CCC(=O)OC1=CC(=CC(=C1)Cl)Cl | 0 | 0 | 2 | 2 |
| β-Alanine, N-(2-chlorobenzoyl)-, undecyl ester (C21H32ClNO3) | OGLQUWIGLHDZMG-UHFFFAOYSA-N | CCCCCCCCCCCOC(=O)CCNC(=O)C1=CC=CC=C1Cl | 821 | 80 | 1 | 2 |
| β-Alanine, N-(4-chlorobenzoyl)-, undecyl ester (C21H32ClNO3) | IRWQMPMLJWACMD-UHFFFAOYSA-N | CCCCCCCCCCCOC(=O)CCNC(=O)C1=CC=C(C=C1)Cl | 611 | 140 | 2 | 1 |
| 2-Chlorobenzoic acid, hexadecyl ester (C23H37ClO2) | LWTORIKGZQIVSM-UHFFFAOYSA-N | CCCCCCCCCCCCCCCCOC(=O)C1=CC=CC=C1Cl | 50 | 0 | 2 | 2 |
| 2-chlorobenzoic acid, 4-hexadecyl ester (C23H37ClO2) | RLHBBBCVZFMPQH-UHFFFAOYSA-N | CCCCCCCCCCCCC(CCC)OC(=O)C1=CC=CC=C1Cl | 0 | 0 | 2 | 2 |
| Benzamide, N,N-dioctyl-4-chloro- (C23H38ClNO) | MLJYWQWBEVWXDK-UHFFFAOYSA-N | CCCCCCCCN(CCCCCCCC)C(=O)C1=CC=C(C=C1)Cl | 1021 | 0 | 1 | 2 |
| Benzamide, N,N-bis(2-ethylhexyl)-4-chloro- (C23H38ClNO) | MMHNMAOTACRUMR-UHFFFAOYSA-N | CCCCC(CC)CN(CC(CC)CCCC)C(=O)C1=CC=C(C=C1)Cl | 50 | 0 | 2 | 2 |
| Fumaric acid, octyl 2,3,4,6-tetrachlorophenyl ester (C18H20Cl4O4) | FBMUHQGBYTWMGK-CMDGGOBGSA-N | CCCCCCCCOC(=O)/C=C/C(=O)OC1=C(C(=C(C=C1Cl)Cl)Cl)Cl | 0 | 0 | 2 | 2 |
| Fumaric acid, nonyl 2,3,6-trichlorophenyl ester (C19H23Cl3O4) | KTDJRTVQFGUUHP-VAWYXSNFSA-N | CCCCCCCCCOC(=O)/C=C/C(=O)OC1=C(C=CC(=C1Cl)Cl)Cl | 80 | 0 | 2 | 2 |
| Fumaric acid, nonyl 2,4,6-trichlorophenyl ester (C19H23Cl3O4) | IKTKSBWABKVBNL-MDZDMXLPSA-N | CCCCCCCCCOC(=O)/C=C/C(=O)OC1=C(C=C(C=C1Cl)Cl)Cl | 20 | 0 | 2 | 2 |
| Fumaric acid, nonyl 2,3,5-trichlorophenyl ester (C19H23Cl3O4) | WBRWKWLWCRLKMK-MDZDMXLPSA-N | CCCCCCCCCOC(=O)/C=C/C(=O)OC1=CC(=CC(=C1Cl)Cl)Cl | 20 | 0 | 2 | 2 |
| Succinic acid, di(2,3,5-trichlorophenyl) ester (C16H8Cl6O4) | POGRXWJDWDDOOU-UHFFFAOYSA-N | C1=C(C=C(C(=C1Cl)Cl)OC(=O)CCC(=O)OC2=CC(=CC(=C2Cl)Cl)Cl)Cl | 0 | 0 | 2 | 2 |
| Succinic acid, di(2,3,6-trichlorophenyl) ester (C16H8Cl6O4) | ATCHWTIBPUOJAN-UHFFFAOYSA-N | C1=CC(=C(C(=C1Cl)OC(=O)CCC(=O)OC2=C(C=CC(=C2Cl)Cl)Cl)Cl)Cl | 0 | 0 | 2 | 2 |
| L-Cysteine,N,O-bis(2-chlorobenzoyl)-, methyl ester (C18H15Cl2NO4S) | FLHMCEAYKRLRKK-UHFFFAOYSA-N | COC(=O)C(CSC(=O)C1=CC=CC=C1Cl)NC(=O)C2=CC=CC=C2Cl | 0 | 0 | 2 | 2 |
| L-Serine, N,O-bis(2-chlorobenzoyl)-, methyl ester (C18H15Cl2NO5) | XKVADCVCQJNALI-UHFFFAOYSA-N | COC(=O)C(COC(=O)C1=CC=CC=C1Cl)NC(=O)C2=CC=CC=C2Cl | 0 | 0 | 2 | 2 |
| Fumaric acid, di(3,4,5-trichlorophenyl) ester (C16H6Cl6O4) | YMFSLFGSHZYXLA-OWOJBTEDSA-N | C1=C(C=C(C(=C1Cl)Cl)Cl)OC(=O)/C=C/C(=O)OC2=CC(=C(C(=C2)Cl)Cl)Cl | 230 | 0 | 2 | 2 |
| Fumaric acid, di(2,3,6-trichlorophenyl) ester (C16H6Cl6O4) | LGRPINVRSGDPDU-AATRIKPKSA-N | C1=CC(=C(C(=C1Cl)OC(=O)/C=C/C(=O)OC2=C(C=CC(=C2Cl)Cl)Cl)Cl)Cl | 250 | 0 | 2 | 2 |
| Fumaric acid, di(2,4,6-trichlorophenyl) ester (C16H6Cl6O4) | PVAYWDBKLFNXLA-OWOJBTEDSA-N | C1=C(C=C(C(=C1Cl)OC(=O)/C=C/C(=O)OC2=C(C=C(C=C2Cl)Cl)Cl)Cl)Cl | 90 | 0 | 2 | 2 |
| Fumaric acid, di(2,3,5-trichlorophenyl) ester (C16H6Cl6O4) | QJKRQIKEUMJKOO-OWOJBTEDSA-N | C1=C(C=C(C(=C1Cl)Cl)OC(=O)/C=C/C(=O)OC2=CC(=CC(=C2Cl)Cl)Cl)Cl | 220 | 0 | 2 | 2 |
| Benzthiazide (C15H14ClN3O4S3) | NDTSRXAMMQDVSW-UHFFFAOYSA-N | C1=CC=C(C=C1)CSCC2=NS(=O)(=O)C3=CC(=C(C=C3N2)Cl)S(=O)(=O)N | 190 | 0 | 2 | 2 |
| Camazepam (C19H18ClN3O3) | PXBVEXGRHZFEOF-UHFFFAOYSA-N | CN1C2=C(C=C(C=C2)Cl)C(=NC(C1=O)OC(=O)N(C)C)C3=CC=CC=C3 | 330 | 0 | 2 | 2 |
| Quizalofop-P-ethyl (C19H17ClN2O4) | OSUHJPCHFDQAIT-UHFFFAOYSA-N | CCOC(=O)C(C)OC1=CC=C(C=C1)OC2=CN=C3C=C(C=CC3=N2)Cl | 8858 | 20 | 1 | 2 |
| Propanoic acid, 2-[4-[(6-chloro-2-quinoxalinyl)oxy]phenoxy]-, ethyl ester (C19H17ClN2O4) | OSUHJPCHFDQAIT-UHFFFAOYSA-N | CCOC(=O)C(C)OC1=CC=C(C=C1)OC2=CN=C3C=C(C=CC3=N2)Cl | 7779 | 10 | 1 | 2 |
| Hydroxyzine (C21H27ClN2O2) | ZQDWXGKKHFNSQK-UHFFFAOYSA-N | C1CN(CCN1CCOCCO)C(C2=CC=CC=C2)C3=CC=C(C=C3)Cl | 640 | 0 | 2 | 2 |
| Lorcainide (C22H27ClN2O) | XHOJAWVAWFHGHL-UHFFFAOYSA-N | CC(C)N1CCC(CC1)N(C2=CC=C(C=C2)Cl)C(=O)CC3=CC=CC=C3 | 1000 | 0 | 1 | 2 |
| Indomethacin methyl ester (C20H18ClNO4) | OKHORWCUMZIORR-UHFFFAOYSA-N | CC1=C(C2=C(N1C(=O)C3=CC=C(C=C3)Cl)C=CC(=C2)OC)CC(=O)OC | 1601 | 0 | 1 | 2 |
| trans-Permethrin (C21H20Cl2O3) | RLLPVAHGXHCWKJ-MJGOQNOKSA-N | CC1([C@@H]([C@H]1C(=O)OCC2=CC(=CC=C2)OC3=CC=CC=C3)C=C(Cl)Cl)C | 60 | 20 | 2 | 2 |
| Permethrin (C21H20Cl2O3) | RLLPVAHGXHCWKJ-HKUYNNGSSA-N | CC1([C@H]([C@H]1C(=O)OCC2=CC(=CC=C2)OC3=CC=CC=C3)C=C(Cl)Cl)C | 205 | 74 | 2 | 2 |
| cis-Permethrin (C21H20Cl2O3) | RLLPVAHGXHCWKJ-HKUYNNGSSA-N | CC1([C@H]([C@H]1C(=O)OCC2=CC(=CC=C2)OC3=CC=CC=C3)C=C(Cl)Cl)C | 60 | 20 | 2 | 2 |
| Benzamide, N-(4-chloro-9,10-dihydro-9,10-dioxo-1-anthracenyl)- (C21H12ClNO3) | FNCVZYRPXOZNSM-UHFFFAOYSA-N | C1=CC=C(C=C1)C(=O)NC2=C3C(=C(C=C2)Cl)C(=O)C4=CC=CC=C4C3=O | 1017 | 2 | 1 | 2 |
| Trazodone (C19H22ClN5O) | PHLBKPHSAVXXEF-UHFFFAOYSA-N | C1CN(CCN1CCCN2C(=O)N3C=CC=CC3=N2)C4=CC(=CC=C4)Cl | 550 | 0 | 2 | 2 |
| Succinic acid, 10-chlorodecyl octyl ester (C22H41ClO4) | DQOQXOJSIDJDSP-UHFFFAOYSA-N | CCCCCCCCOC(=O)CCC(=O)OCCCCCCCCCCCl | 0 | 50 | 2 | 2 |
| Succinic acid, 8-chloroctyl decyl ester (C22H41ClO4) | CWSIPAVSNOPMPI-UHFFFAOYSA-N | CCCCCCCCCCOC(=O)CCC(=O)OCCCCCCCCCl | 0 | 20 | 2 | 2 |
| Adipic acid, 8-chloroctyl octyl ester (C22H41ClO4) | PNWBSXSNFVCEKW-UHFFFAOYSA-N | CCCCCCCCOC(=O)CCCCC(=O)OCCCCCCCCCl | 0 | 30 | 2 | 2 |
| Chloroacetamide, N,N-diundecyl- (C24H48ClNO) | OHKMCMGARDHSJI-UHFFFAOYSA-N | CCCCCCCCCCCN(CCCCCCCCCCC)C(=O)CCl | 40 | 4434 | 2 | 1 |
| Hexanamide, N,N-dinonyl-6-chloro- (C24H48ClNO) | ARRDNVVGNRQBES-UHFFFAOYSA-N | CCCCCCCCCN(CCCCCCCCC)C(=O)CCCCCCl | 90 | 961 | 2 | 1 |
| Succinic acid, 2,2-dichloroethyl pentadecyl ester (C21H38Cl2O4) | SLAPQLXXZFVIOI-UHFFFAOYSA-N | CCCCCCCCCCCCCCCOC(=O)CCC(=O)OCC(Cl)Cl | 0 | 0 | 2 | 2 |
| Succinic acid, tetradecyl 2,2,2-trichloroethyl ester (C20H35Cl3O4) | GDSXXLDWZMMLAH-UHFFFAOYSA-N | CCCCCCCCCCCCCCOC(=O)CCC(=O)OCC(Cl)(Cl)Cl | 0 | 0 | 2 | 2 |
| Fumaric acid, 10-chlorodecyl octyl ester (C22H39ClO4) | LCSYCOIHYYRUDF-WUKNDPDISA-N | CCCCCCCCOC(=O)/C=C/C(=O)OCCCCCCCCCCCl | 0 | 30 | 2 | 2 |
| Fumaric acid, 8-chlorooctyl decyl ester (C22H39ClO4) | LZFVNKBKTMUJRA-WUKNDPDISA-N | CCCCCCCCCCOC(=O)/C=C/C(=O)OCCCCCCCCCl | 0 | 0 | 2 | 2 |
| Fumaric acid, 2-chloropropyl pentadecyl ester (C22H39ClO4) | MQORINRNGKZFOW-WUKNDPDISA-N | CCCCCCCCCCCCCCCOC(=O)/C=C/C(=O)OCC(C)Cl | 60 | 80 | 2 | 2 |
| Fumaric acid, 2,2-dichloroethyl pentadecyl ester (C21H36Cl2O4) | MWXYEQDJMAXBGQ-FOCLMDBBSA-N | CCCCCCCCCCCCCCCOC(=O)/C=C/C(=O)OCC(Cl)Cl | 30 | 130 | 2 | 1 |
| Succinic acid, nonyl 2,3,4,6-tetrachlorophenyl ester (C19H24Cl4O4) | FRUHYSIQIICQSO-UHFFFAOYSA-N | CCCCCCCCCOC(=O)CCC(=O)OC1=C(C(=C(C=C1Cl)Cl)Cl)Cl | 0 | 0 | 2 | 2 |
| Succinic acid, decyl 2,3,5-trichlorophenyl ester (C20H27Cl3O4) | LQMDAUQYGDNOKV-UHFFFAOYSA-N | CCCCCCCCCCOC(=O)CCC(=O)OC1=CC(=CC(=C1Cl)Cl)Cl | 0 | 0 | 2 | 2 |
| Succinic acid, decyl 2,3,6-trichlorophenyl ester (C20H27Cl3O4) | YRPFYQAPLPILAM-UHFFFAOYSA-N | CCCCCCCCCCOC(=O)CCC(=O)OC1=C(C=CC(=C1Cl)Cl)Cl | 0 | 0 | 2 | 2 |
| Sarcosine, N-(2-chlorobenzoyl)-, dodecyl ester (C22H34ClNO3) | QNPJVNNCUBIYBF-UHFFFAOYSA-N | CCCCCCCCCCCCOC(=O)CN(C)C(=O)C1=CC=CC=C1Cl | 941 | 90 | 1 | 2 |
| Sarcosine, N-(4-chlorobenzoyl)-, dodecyl ester (C22H34ClNO3) | APPPTNVALNXEJO-UHFFFAOYSA-N | CCCCCCCCCCCCOC(=O)CN(C)C(=O)C1=CC=C(C=C1)Cl | 1131 | 0 | 1 | 2 |
| β-Alanine, N-(2-chlorobenzoyl)-, dodecyl ester (C22H34ClNO3) | HEZFSHWAKAXNQU-UHFFFAOYSA-N | CCCCCCCCCCCCOC(=O)CCNC(=O)C1=CC=CC=C1Cl | 821 | 70 | 1 | 2 |
| β-Alanine, N-(4-chlorobenzoyl)-, dodecyl ester (C22H34ClNO3) | CIEGTMHJJZHPCN-UHFFFAOYSA-N | CCCCCCCCCCCCOC(=O)CCNC(=O)C1=CC=C(C=C1)Cl | 691 | 230 | 2 | 1 |
| 2-chlorobenzoic acid, heptadecyl ester (C24H39ClO2) | BODQEIRZCIRWTL-UHFFFAOYSA-N | CCCCCCCCCCCCCCCCCOC(=O)C1=CC=CC=C1Cl | 0 | 0 | 2 | 2 |
| Fumaric acid, nonyl 2,3,4,6-tetrachlorophenyl ester (C19H22Cl4O4) | HXFNNNWNGSGHRR-MDZDMXLPSA-N | CCCCCCCCCOC(=O)/C=C/C(=O)OC1=C(C(=C(C=C1Cl)Cl)Cl)Cl | 20 | 0 | 2 | 2 |
| Fumaric acid, decyl 2,3,6-trichlorophenyl ester (C20H25Cl3O4) | UKPVUYCTLVSBPN-OUKQBFOZSA-N | CCCCCCCCCCOC(=O)/C=C/C(=O)OC1=C(C=CC(=C1Cl)Cl)Cl | 70 | 0 | 2 | 2 |
| Fumaric acid, decyl 2,3,5-trichlorophenyl ester (C20H25Cl3O4) | LWPBKWZRDHPSKM-ZHACJKMWSA-N | CCCCCCCCCCOC(=O)/C=C/C(=O)OC1=CC(=CC(=C1Cl)Cl)Cl | 0 | 0 | 2 | 2 |
| Fumaric acid, 2-chloro-5-methylphenyl undecyl ester (C22H31ClO4) | VGDMPBSGYSHRRP-CCEZHUSRSA-N | CCCCCCCCCCCOC(=O)/C=C/C(=O)OC1=C(C=CC(=C1)C)Cl | 180 | 260 | 2 | 1 |
| Bicyclo[2.2.1]hept-5-ene-2,3-dicarboxylic acid, 1,4,5,6,7,7-hexachloro-, dibutyl ester (C17H20Cl6O4) | UJAHPBDUQZFDLA-UHFFFAOYSA-N | CCCCOC(=O)C1C(C2(C(=C(C1(C2(Cl)Cl)Cl)Cl)Cl)Cl)C(=O)OCCCC | 0 | 0 | 2 | 2 |
| Pyraclostrobin (C19H18ClN3O4) | HZRSNVGNWUDEFX-UHFFFAOYSA-N | COC(=O)N(C1=CC=CC=C1COC2=NN(C=C2)C3=CC=C(C=C3)Cl)OC | 0 | 0 | 2 | 2 |
| Chlorotrianisene (C23H21ClO3) | BFPSDSIWYFKGBC-UHFFFAOYSA-N | COC1=CC=C(C=C1)C(=C(C2=CC=C(C=C2)OC)Cl)C3=CC=C(C=C3)OC | 9999 | 2452 | 1 | 1 |
| Zopiclone (C17H17ClN6O3) | GBBSUAFBMRNDJC-UHFFFAOYSA-N | CN1CCN(CC1)C(=O)OC2C3=NC=CN=C3C(=O)N2C4=NC=C(C=C4)Cl | 0 | 0 | 2 | 2 |
| difenaconazole (C19H17Cl2N3O3) | BQYJATMQXGBDHF-UHFFFAOYSA-N | CC1COC(O1)(CN2C=NC=N2)C3=C(C=C(C=C3)OC4=CC=C(C=C4)Cl)Cl | 0 | 30 | 2 | 2 |
| Perphenazine (C21H26ClN3OS) | RGCVKNLCSQQDEP-UHFFFAOYSA-N | C1CN(CCN1CCCN2C3=CC=CC=C3SC4=C2C=C(C=C4)Cl)CCO | 3483 | 110 | 1 | 1 |
| Chlorophacinon (C23H15ClO3) | UDHXJZHVNHGCEC-UHFFFAOYSA-N | C1=CC=C(C=C1)C(C2=CC=C(C=C2)Cl)C(=O)C3C(=O)C4=CC=CC=C4C3=O | 1899 | 0 | 1 | 2 |
| Zuclopenthixol (C22H25ClN2OS) | WFPIAZLQTJBIFN-BLLMUTORSA-N | C1CN(CCN1CC/C=C/2\C3=CC=CC=C3SC4=C2C=C(C=C4)Cl)CCO | 40 | 0 | 2 | 2 |
| Endrin (C12H8Cl6O) | DFBKLUNHFCTMDC-GKRDHZSOSA-N | C1[C@@H]2[C@@H]3[C@H]([C@H]1[C@H]4[C@@H]2O4)[C@@]5(C(=C([C@]3(C5(Cl)Cl)Cl)Cl)Cl)Cl | 70 | 1169 | 2 | 1 |
| Succinic acid, 10-chlorodecyl nonyl ester (C23H43ClO4) | LKVQTKOCGFMGTP-UHFFFAOYSA-N | CCCCCCCCCOC(=O)CCC(=O)OCCCCCCCCCCCl | 0 | 40 | 2 | 2 |
[truncated: 12,711 more chars]
