# Supplementary material for: The use of fast molecular descriptors and artificial neural networks approach in organochlorine compounds electron ionization mass spectra classification
Source: Environ Sci Pollut Res Int. 2019 Jul 30;26(27):28188–201. doi: 10.1007/s11356-019-05968-4 (PMC6791912; doi:10.1007/s11356-019-05968-4)
Supplement: Supplementary file 2 — (DOCX 364 kb) [file 11356_2019_5968_MOESM2_ESM.docx]

**Supplementary Material S2**

**The use of fast molecular descriptors and artificial neural networks approach** **in organochlorine compounds electron ionization mass spectra classification**

Maciej Przybyłek^a^, Waldemar Studziński^b^, Alicja Gackowska^b^ and Jerzy Gaca^b^

^a^*Chair and Department of Physical Chemistry, Pharmacy Faculty, Collegium Medicum of Bydgoszcz, Nicolaus Copernicus University in Toruń, Kurpińskiego 5, 85-950 Bydgoszcz, Poland,*

^b^*Faculty of Chemical Technology and Engineering, University of Technology and Life Science, Seminaryjna 3, 85-326 Bydgoszcz, Poland*

Table of Contents

[1. Criterion I-based ANN classification models in PMML format 2](#_Toc528425999)

[1.1. MLP 100-19-2 2](#_Toc528426000)

[1.2. MLP 100-23-2 12](#_Toc528426001)

[1.3. MLP 100-15-2 23](#_Toc528426002)

[1.4. MLP 100-25-2 32](#_Toc528426003)

[1.5. MLP 100-21-2 44](#_Toc528426004)

[2. Criterion II-based ANN classification models in PMML format 55](#_Toc528426005)

[2.1. MLP 100-25-2 55](#_Toc528426006)

[2.2. MLP 100-22-2 (BFGS 73) 67](#_Toc528426007)

[2.3. MLP 100-22-2 (BFGS 72) 78](#_Toc528426008)

[2.4. MLP 100-22-2 (BFGS 48) 89](#_Toc528426009)

[2.5. MLP 100-24-2 100](#_Toc528426010)

1. Criterion I-based ANN classification models in PMML format

## 1.1. MLP 100-19-2

<?xml version="1.0" encoding="UTF-8"?>

<PMML version="3.0"><Header copyright="Copyright (c) StatSoft, Inc. All Rights Reserved."><Application name="STATISTICA Automated Neural Networks (SANN)" version="2.0"/></Header><DataDictionary numberOfFields="101"><DataField name="[M] class" optype="categorical"><Value value="1"/><Value value="2"/></DataField><DataField name="nN" optype="continuous"/><DataField name="AATS6i" optype="continuous"/><DataField name="AATS7i" optype="continuous"/><DataField name="AATS8i" optype="continuous"/><DataField name="ATSC3c" optype="continuous"/><DataField name="ATSC0m" optype="continuous"/><DataField name="ATSC3e" optype="continuous"/><DataField name="AATSC0e" optype="continuous"/><DataField name="AATSC0i" optype="continuous"/><DataField name="MATS1c" optype="continuous"/><DataField name="MATS2s" optype="continuous"/><DataField name="GATS1c" optype="continuous"/><DataField name="GATS3c" optype="continuous"/><DataField name="GATS4e" optype="continuous"/><DataField name="GATS4i" optype="continuous"/><DataField name="GATS2s" optype="continuous"/><DataField name="GATS3s" optype="continuous"/><DataField name="GATS4s" optype="continuous"/><DataField name="VR2_Dzs" optype="continuous"/><DataField name="nBase" optype="continuous"/><DataField name="BCUTw-1l" optype="continuous"/><DataField name="BCUTc-1l" optype="continuous"/><DataField name="BCUTc-1h" optype="continuous"/><DataField name="C1SP2" optype="continuous"/><DataField name="C2SP2" optype="continuous"/><DataField name="C3SP2" optype="continuous"/><DataField name="C1SP3" optype="continuous"/><DataField name="C4SP3" optype="continuous"/><DataField name="VE1_Dt" optype="continuous"/><DataField name="nHCsatu" optype="continuous"/><DataField name="nsCH3" optype="continuous"/><DataField name="nsssN" optype="continuous"/><DataField name="ndS" optype="continuous"/><DataField name="nssS" optype="continuous"/><DataField name="SHCsatu" optype="continuous"/><DataField name="minHBd" optype="continuous"/><DataField name="minwHBa" optype="continuous"/><DataField name="minHBint2" optype="continuous"/><DataField name="minHssNH" optype="continuous"/><DataField name="minHdsCH" optype="continuous"/><DataField name="minHCsats" optype="continuous"/><DataField name="minHCsatu" optype="continuous"/><DataField name="minHother" optype="continuous"/><DataField name="minssCH2" optype="continuous"/><DataField name="minaaCH" optype="continuous"/><DataField name="minaasC" optype="continuous"/><DataField name="minsNH2" optype="continuous"/><DataField name="minsssN" optype="continuous"/><DataField name="mindO" optype="continuous"/><DataField name="minssO" optype="continuous"/><DataField name="maxHBd" optype="continuous"/><DataField name="maxHBa" optype="continuous"/><DataField name="maxwHBa" optype="continuous"/><DataField name="maxHBint2" optype="continuous"/><DataField name="maxHssNH" optype="continuous"/><DataField name="maxHdsCH" optype="continuous"/><DataField name="maxHCsats" optype="continuous"/><DataField name="maxHCsatu" optype="continuous"/><DataField name="maxHother" optype="continuous"/><DataField name="maxsCH3" optype="continuous"/><DataField name="maxaaCH" optype="continuous"/><DataField name="maxsssCH" optype="continuous"/><DataField name="maxdssC" optype="continuous"/><DataField name="maxaasC" optype="continuous"/><DataField name="maxssssC" optype="continuous"/><DataField name="maxsNH2" optype="continuous"/><DataField name="maxsssN" optype="continuous"/><DataField name="maxdO" optype="continuous"/><DataField name="maxssO" optype="continuous"/><DataField name="maxsCl" optype="continuous"/><DataField name="gmax" optype="continuous"/><DataField name="MAXDN2" optype="continuous"/><DataField name="MAXDP2" optype="continuous"/><DataField name="ETA_Shape_P" optype="continuous"/><DataField name="ETA_BetaP" optype="continuous"/><DataField name="ETA_BetaP_ns" optype="continuous"/><DataField name="ETA_BetaP_ns_d" optype="continuous"/><DataField name="FMF" optype="continuous"/><DataField name="nHBAcc" optype="continuous"/><DataField name="SIC5" optype="continuous"/><DataField name="BIC5" optype="continuous"/><DataField name="nAtomP" optype="continuous"/><DataField name="nAtomLAC" optype="continuous"/><DataField name="MDEO-12" optype="continuous"/><DataField name="MLFER_S" optype="continuous"/><DataField name="MLFER_E" optype="continuous"/><DataField name="piPC4" optype="continuous"/><DataField name="piPC5" optype="continuous"/><DataField name="piPC6" optype="continuous"/><DataField name="piPC8" optype="continuous"/><DataField name="piPC9" optype="continuous"/><DataField name="piPC10" optype="continuous"/><DataField name="n6Ring" optype="continuous"/><DataField name="RotBFrac" optype="continuous"/><DataField name="RotBtFrac" optype="continuous"/><DataField name="LipinskiFailures" optype="continuous"/><DataField name="GGI6" optype="continuous"/><DataField name="GGI8" optype="continuous"/><DataField name="JGT" optype="continuous"/><DataField name="WTPT-5" optype="continuous"/></DataDictionary><NeuralNetwork modelName="Ark_MLP 100-19-2" functionName="classification"><MiningSchema><MiningField name="[M] class" usageType="predicted"/><MiningField name="nN" lowValue="0.000000" highValue="6.000000"/><MiningField name="AATS6i" lowValue="0.000000" highValue="197.783615"/><MiningField name="AATS7i" lowValue="0.000000" highValue="197.641130"/><MiningField name="AATS8i" lowValue="0.000000" highValue="197.641130"/><MiningField name="ATSC3c" lowValue="-0.709297" highValue="0.477175"/><MiningField name="ATSC0m" lowValue="337.724329" highValue="5532.361122"/><MiningField name="ATSC3e" lowValue="-4.603184" highValue="4.952578"/><MiningField name="AATSC0e" lowValue="0.016140" highValue="0.218137"/><MiningField name="AATSC0i" lowValue="0.466396" highValue="2.091475"/><MiningField name="MATS1c" lowValue="-1.042399" highValue="0.161674"/><MiningField name="MATS2s" lowValue="-1.343888" highValue="0.732539"/><MiningField name="GATS1c" lowValue="0.611983" highValue="2.500000"/><MiningField name="GATS3c" lowValue="0.000000" highValue="2.472448"/><MiningField name="GATS4e" lowValue="0.000000" highValue="3.344208"/><MiningField name="GATS4i" lowValue="0.000000" highValue="2.045863"/><MiningField name="GATS2s" lowValue="0.000000" highValue="2.123355"/><MiningField name="GATS3s" lowValue="0.000000" highValue="3.206156"/><MiningField name="GATS4s" lowValue="0.000000" highValue="4.782779"/><MiningField name="VR2_Dzs" lowValue="1.304698" highValue="800000.000000"/><MiningField name="nBase" lowValue="0.000000" highValue="6.000000"/><MiningField name="BCUTw-1l" lowValue="11.690000" highValue="12.000000"/><MiningField name="BCUTc-1l" lowValue="-0.419857" highValue="-0.118299"/><MiningField name="BCUTc-1h" lowValue="0.049763" highValue="0.530105"/><MiningField name="C1SP2" lowValue="0.000000" highValue="5.000000"/><MiningField name="C2SP2" lowValue="0.000000" highValue="17.000000"/><MiningField name="C3SP2" lowValue="0.000000" highValue="6.000000"/><MiningField name="C1SP3" lowValue="0.000000" highValue="9.000000"/><MiningField name="C4SP3" lowValue="0.000000" highValue="2.000000"/><MiningField name="VE1_Dt" lowValue="0.000000" highValue="0.484850"/><MiningField name="nHCsatu" lowValue="0.000000" highValue="4.000000"/><MiningField name="nsCH3" lowValue="0.000000" highValue="6.000000"/><MiningField name="nsssN" lowValue="0.000000" highValue="4.000000"/><MiningField name="ndS" lowValue="0.000000" highValue="1.000000"/><MiningField name="nssS" lowValue="0.000000" highValue="3.000000"/><MiningField name="SHCsatu" lowValue="0.000000" highValue="2.382460"/><MiningField name="minHBd" lowValue="0.000000" highValue="0.869082"/><MiningField name="minwHBa" lowValue="-1.399568" highValue="3.235284"/><MiningField name="minHBint2" lowValue="-0.511389" highValue="8.289887"/><MiningField name="minHssNH" lowValue="0.000000" highValue="0.677623"/><MiningField name="minHdsCH" lowValue="0.000000" highValue="0.757925"/><MiningField name="minHCsats" lowValue="0.000000" highValue="0.936911"/><MiningField name="minHCsatu" lowValue="0.000000" highValue="1.041473"/><MiningField name="minHother" lowValue="0.000000" highValue="0.760540"/><MiningField name="minssCH2" lowValue="-0.673675" highValue="1.727730"/><MiningField name="minaaCH" lowValue="0.000000" highValue="2.620150"/><MiningField name="minaasC" lowValue="-0.589846" highValue="1.951475"/><MiningField name="minsNH2" lowValue="0.000000" highValue="6.075669"/><MiningField name="minsssN" lowValue="0.000000" highValue="2.664352"/><MiningField name="mindO" lowValue="0.000000" highValue="13.824902"/><MiningField name="minssO" lowValue="0.000000" highValue="6.570147"/><MiningField name="maxHBd" lowValue="0.000000" highValue="0.869082"/><MiningField name="maxHBa" lowValue="0.531293" highValue="13.824902"/><MiningField name="maxwHBa" lowValue="0.000000" highValue="5.432183"/><MiningField name="maxHBint2" lowValue="0.000000" highValue="8.289887"/><MiningField name="maxHssNH" lowValue="0.000000" highValue="0.677623"/><MiningField name="maxHdsCH" lowValue="0.000000" highValue="0.757925"/><MiningField name="maxHCsats" lowValue="0.000000" highValue="1.011564"/><MiningField name="maxHCsatu" lowValue="0.000000" highValue="1.147376"/><MiningField name="maxHother" lowValue="0.000000" highValue="0.786998"/><MiningField name="maxsCH3" lowValue="0.000000" highValue="2.473813"/><MiningField name="maxaaCH" lowValue="0.000000" highValue="2.636307"/><MiningField name="maxsssCH" lowValue="0.000000" highValue="1.366427"/><MiningField name="maxdssC" lowValue="0.000000" highValue="2.211231"/><MiningField name="maxaasC" lowValue="0.000000" highValue="2.077332"/><MiningField name="maxssssC" lowValue="0.000000" highValue="1.365569"/><MiningField name="maxsNH2" lowValue="0.000000" highValue="6.075669"/><MiningField name="maxsssN" lowValue="0.000000" highValue="2.664352"/><MiningField name="maxdO" lowValue="0.000000" highValue="13.824902"/><MiningField name="maxssO" lowValue="0.000000" highValue="6.574487"/><MiningField name="maxsCl" lowValue="0.000000" highValue="1.588820"/><MiningField name="gmax" lowValue="1.350137" highValue="13.824902"/><MiningField name="MAXDN2" lowValue="0.420972" highValue="5.264383"/><MiningField name="MAXDP2" lowValue="0.415123" highValue="6.810793"/><MiningField name="ETA_Shape_P" lowValue="0.062580" highValue="0.851060"/><MiningField name="ETA_BetaP" lowValue="0.437500" highValue="1.750000"/><MiningField name="ETA_BetaP_ns" lowValue="0.000000" highValue="1.192310"/><MiningField name="ETA_BetaP_ns_d" lowValue="0.000000" highValue="0.250000"/><MiningField name="FMF" lowValue="0.000000" highValue="0.642857"/><MiningField name="nHBAcc" lowValue="0.000000" highValue="9.000000"/><MiningField name="SIC5" lowValue="0.000000" highValue="1.000000"/><MiningField name="BIC5" lowValue="0.000000" highValue="0.933062"/><MiningField name="nAtomP" lowValue="0.000000" highValue="25.000000"/><MiningField name="nAtomLAC" lowValue="0.000000" highValue="20.000000"/><MiningField name="MDEO-12" lowValue="0.000000" highValue="2.149140"/><MiningField name="MLFER_S" lowValue="0.166000" highValue="4.011000"/><MiningField name="MLFER_E" lowValue="0.131000" highValue="2.888000"/><MiningField name="piPC4" lowValue="0.000000" highValue="5.794156"/><MiningField name="piPC5" lowValue="0.000000" highValue="6.557754"/><MiningField name="piPC6" lowValue="0.000000" highValue="7.071600"/><MiningField name="piPC8" lowValue="0.000000" highValue="8.204505"/><MiningField name="piPC9" lowValue="0.000000" highValue="8.773305"/><MiningField name="piPC10" lowValue="0.000000" highValue="9.038376"/><MiningField name="n6Ring" lowValue="0.000000" highValue="4.000000"/><MiningField name="RotBFrac" lowValue="0.000000" highValue="0.900000"/><MiningField name="RotBtFrac" lowValue="0.000000" highValue="1.000000"/><MiningField name="LipinskiFailures" lowValue="0.000000" highValue="2.000000"/><MiningField name="GGI6" lowValue="0.000000" highValue="1.144580"/><MiningField name="GGI8" lowValue="0.000000" highValue="0.454775"/><MiningField name="JGT" lowValue="0.075666" highValue="0.926282"/><MiningField name="WTPT-5" lowValue="0.000000" highValue="19.461628"/></MiningSchema><NeuralInputs numberOfInputs="100"><NeuralInput id="0"><DerivedField><NormContinuous field="nN" shift="-0.00000000000000e+000" scale="1.66666666666667e-001"><LinearNorm orig="0.00000000000000e+000" norm="0.000000"/><LinearNorm orig="6.00000000000000e+000" norm="1.000000"/></NormContinuous></DerivedField></NeuralInput><NeuralInput id="1"><DerivedField><NormContinuous field="AATS6i" shift="-0.00000000000000e+000" scale="5.05603053971999e-003"><LinearNorm orig="0.00000000000000e+000" norm="0.000000"/><LinearNorm orig="1.97783615455650e+002" norm="1.000000"/></NormContinuous></DerivedField></NeuralInput><NeuralInput id="2"><DerivedField><NormContinuous field="AATS7i" shift="-0.00000000000000e+000" scale="5.05967557433139e-003"><LinearNorm orig="0.00000000000000e+000" norm="0.000000"/><LinearNorm orig="1.97641130406300e+002" norm="1.000000"/></NormContinuous></DerivedField></NeuralInput><NeuralInput id="3"><DerivedField><NormContinuous field="AATS8i" shift="-0.00000000000000e+000" scale="5.05967557433139e-003"><LinearNorm orig="0.00000000000000e+000" norm="0.000000"/><LinearNorm orig="1.97641130406300e+002" norm="1.000000"/></NormContinuous></DerivedField></NeuralInput><NeuralInput id="4"><DerivedField><NormContinuous field="ATSC3c" shift="5.97820440549968e-001" scale="8.42835077233181e-001"><LinearNorm orig="-7.09297057868622e-001" norm="0.000000"/><LinearNorm orig="4.77174681398273e-001" norm="1.000000"/></NormContinuous></DerivedField></NeuralInput><NeuralInput id="5"><DerivedField><NormContinuous field="ATSC0m" shift="-6.50140408461115e-002" scale="1.92506240526961e-004"><LinearNorm orig="3.37724328666666e+002" norm="0.000000"/><LinearNorm orig="5.53236112206428e+003" norm="1.000000"/></NormContinuous></DerivedField></NeuralInput><NeuralInput id="6"><DerivedField><NormContinuous field="ATSC3e" shift="4.81718150076887e-001" scale="1.04648892923010e-001"><LinearNorm orig="-4.60318438754325e+000" norm="0.000000"/><LinearNorm orig="4.95257843104379e+000" norm="1.000000"/></NormContinuous></DerivedField></NeuralInput><NeuralInput id="7"><DerivedField><NormContinuous field="AATSC0e" shift="-7.99034931570863e-002" scale="4.95056802602201e+000"><LinearNorm orig="1.61402676899063e-002" norm="0.000000"/><LinearNorm orig="2.18137289999999e-001" norm="1.000000"/></NormContinuous></DerivedField></NeuralInput><NeuralInput id="8"><DerivedField><NormContinuous field="AATSC0i" shift="-2.86998985772790e-001" scale="6.15354578529142e-001"><LinearNorm orig="4.66396116623999e-001" norm="0.000000"/><LinearNorm orig="2.09147543656708e+000" norm="1.000000"/></NormContinuous></DerivedField></NeuralInput><NeuralInput id="9"><DerivedField><NormContinuous field="MATS1c" shift="8.65727166044417e-001" scale="8.30513877894299e-001"><LinearNorm orig="-1.04239939763487e+000" norm="0.000000"/><LinearNorm orig="1.61674401270718e-001" norm="1.000000"/></NormContinuous></DerivedField></NeuralInput><NeuralInput id="10"><DerivedField><NormContinuous field="MATS2s" shift="6.47211803411582e-001" scale="4.81596712187758e-001"><LinearNorm orig="-1.34388750386497e+000" norm="0.000000"/><LinearNorm orig="7.32538631723213e-001" norm="1.000000"/></NormContinuous></DerivedField></NeuralInput><NeuralInput id="11"><DerivedField><NormContinuous field="GATS1c" shift="-3.24140792326252e-001" scale="5.29656316930503e-001"><LinearNorm orig="6.11983246428047e-001" norm="0.000000"/><LinearNorm orig="2.49999999999999e+000" norm="1.000000"/></NormContinuous></DerivedField></NeuralInput><NeuralInput id="12"><DerivedField><NormContinuous field="GATS3c" shift="-0.00000000000000e+000" scale="4.04457445713418e-001"><LinearNorm orig="0.00000000000000e+000" norm="0.000000"/><LinearNorm orig="2.47244799322735e+000" norm="1.000000"/></NormContinuous></DerivedField></NeuralInput><NeuralInput id="13"><DerivedField><NormContinuous field="GATS4e" shift="-0.00000000000000e+000" scale="2.99024461462626e-001"><LinearNorm orig="0.00000000000000e+000" norm="0.000000"/><LinearNorm orig="3.34420801264443e+000" norm="1.000000"/></NormContinuous></DerivedField></NeuralInput><NeuralInput id="14"><DerivedField><NormContinuous field="GATS4i" shift="-0.00000000000000e+000" scale="4.88791247683768e-001"><LinearNorm orig="0.00000000000000e+000" norm="0.000000"/><LinearNorm orig="2.04586314656552e+000" norm="1.000000"/></NormContinuous></DerivedField></NeuralInput><NeuralInput id="15"><DerivedField><NormContinuous field="GATS2s" shift="-0.00000000000000e+000" scale="4.70952705983061e-001"><LinearNorm orig="0.00000000000000e+000" norm="0.000000"/><LinearNorm orig="2.12335546074125e+000" norm="1.000000"/></NormContinuous></DerivedField></NeuralInput><NeuralInput id="16"><DerivedField><NormContinuous field="GATS3s" shift="-0.00000000000000e+000" scale="3.11899935981828e-001"><LinearNorm orig="0.00000000000000e+000" norm="0.000000"/><LinearNorm orig="3.20615647724359e+000" norm="1.000000"/></NormContinuous></DerivedField></NeuralInput><NeuralInput id="17"><DerivedField><NormContinuous field="GATS4s" shift="-0.00000000000000e+000" scale="2.09083445127794e-001"><LinearNorm orig="0.00000000000000e+000" norm="0.000000"/><LinearNorm orig="4.78277942755720e+000" norm="1.000000"/></NormContinuous></DerivedField></NeuralInput><NeuralInput id="18"><DerivedField><NormContinuous field="VR2_Dzs" shift="-1.63087485462995e-006" scale="1.25000203859357e-006"><LinearNorm orig="1.30469775590520e+000" norm="0.000000"/><LinearNorm orig="8.00000000000000e+005" norm="1.000000"/></NormContinuous></DerivedField></NeuralInput><NeuralInput id="19"><DerivedField><NormContinuous field="nBase" shift="-0.00000000000000e+000" scale="1.66666666666667e-001"><LinearNorm orig="0.00000000000000e+000" norm="0.000000"/><LinearNorm orig="6.00000000000000e+000" norm="1.000000"/></NormContinuous></DerivedField></NeuralInput><NeuralInput id="20"><DerivedField><NormContinuous field="BCUTw-1l" shift="-3.77097046318940e+001" scale="3.22580877946057e+000"><LinearNorm orig="1.16900000000000e+001" norm="0.000000"/><LinearNorm orig="1.19999997762940e+001" norm="1.000000"/></NormContinuous></DerivedField></NeuralInput><NeuralInput id="21"><DerivedField><NormContinuous field="BCUTc-1l" shift="1.39229140590098e+000" scale="3.31610617756096e+000"><LinearNorm orig="-4.19857305933739e-001" norm="0.000000"/><LinearNorm orig="-1.18298807364941e-001" norm="1.000000"/></NormContinuous></DerivedField></NeuralInput><NeuralInput id="22"><DerivedField><NormContinuous field="BCUTc-1h" shift="-1.03599084775896e-001" scale="2.08184959336469e+000"><LinearNorm orig="4.97630016626027e-002" norm="0.000000"/><LinearNorm orig="5.30105098991448e-001" norm="1.000000"/></NormContinuous></DerivedField></NeuralInput><NeuralInput id="23"><DerivedField><NormContinuous field="C1SP2" shift="-0.00000000000000e+000" scale="2.00000000000000e-001"><LinearNorm orig="0.00000000000000e+000" norm="0.000000"/><LinearNorm orig="5.00000000000000e+000" norm="1.000000"/></NormContinuous></DerivedField></NeuralInput><NeuralInput id="24"><DerivedField><NormContinuous field="C2SP2" shift="-0.00000000000000e+000" scale="5.88235294117647e-002"><LinearNorm orig="0.00000000000000e+000" norm="0.000000"/><LinearNorm orig="1.70000000000000e+001" norm="1.000000"/></NormContinuous></DerivedField></NeuralInput><NeuralInput id="25"><DerivedField><NormContinuous field="C3SP2" shift="-0.00000000000000e+000" scale="1.66666666666667e-001"><LinearNorm orig="0.00000000000000e+000" norm="0.000000"/><LinearNorm orig="6.00000000000000e+000" norm="1.000000"/></NormContinuous></DerivedField></NeuralInput><NeuralInput id="26"><DerivedField><NormContinuous field="C1SP3" shift="-0.00000000000000e+000" scale="1.11111111111111e-001"><LinearNorm orig="0.00000000000000e+000" norm="0.000000"/><LinearNorm orig="9.00000000000000e+000" norm="1.000000"/></NormContinuous></DerivedField></NeuralInput><NeuralInput id="27"><DerivedField><NormContinuous field="C4SP3" shift="-0.00000000000000e+000" scale="5.00000000000000e-001"><LinearNorm orig="0.00000000000000e+000" norm="0.000000"/><LinearNorm orig="2.00000000000000e+000" norm="1.000000"/></NormContinuous></DerivedField></NeuralInput><NeuralInput id="28"><DerivedField><NormContinuous field="VE1_Dt" shift="-0.00000000000000e+000" scale="2.06249354028337e+000"><LinearNorm orig="0.00000000000000e+000" norm="0.000000"/><LinearNorm orig="4.84850003390852e-001" norm="1.000000"/></NormContinuous></DerivedField></NeuralInput><NeuralInput id="29"><DerivedField><NormContinuous field="nHCsatu" shift="-0.00000000000000e+000" scale="2.50000000000000e-001"><LinearNorm orig="0.00000000000000e+000" norm="0.000000"/><LinearNorm orig="4.00000000000000e+000" norm="1.000000"/></NormContinuous></DerivedField></NeuralInput><NeuralInput id="30"><DerivedField><NormContinuous field="nsCH3" shift="-0.00000000000000e+000" scale="1.66666666666667e-001"><LinearNorm orig="0.00000000000000e+000" norm="0.000000"/><LinearNorm orig="6.00000000000000e+000" norm="1.000000"/></NormContinuous></DerivedField></NeuralInput><NeuralInput id="31"><DerivedField><NormContinuous field="nsssN" shift="-0.00000000000000e+000" scale="2.50000000000000e-001"><LinearNorm orig="0.00000000000000e+000" norm="0.000000"/><LinearNorm orig="4.00000000000000e+000" norm="1.000000"/></NormContinuous></DerivedField></NeuralInput><NeuralInput id="32"><DerivedField><NormContinuous field="ndS" shift="-0.00000000000000e+000" scale="1.00000000000000e+000"><LinearNorm orig="0.00000000000000e+000" norm="0.000000"/><LinearNorm orig="1.00000000000000e+000" norm="1.000000"/></NormContinuous></DerivedField></NeuralInput><NeuralInput id="33"><DerivedField><NormContinuous field="nssS" shift="-0.00000000000000e+000" scale="3.33333333333333e-001"><LinearNorm orig="0.00000000000000e+000" norm="0.000000"/><LinearNorm orig="3.00000000000000e+000" norm="1.000000"/></NormContinuous></DerivedField></NeuralInput><NeuralInput id="34"><DerivedField><NormContinuous field="SHCsatu" shift="-0.00000000000000e+000" scale="4.19734199013930e-001"><LinearNorm orig="0.00000000000000e+000" norm="0.000000"/><LinearNorm orig="2.38246014346525e+000" norm="1.000000"/></NormContinuous></DerivedField></NeuralInput><NeuralInput id="35"><DerivedField><NormContinuous field="minHBd" shift="-0.00000000000000e+000" scale="1.15063968819085e+000"><LinearNorm orig="0.00000000000000e+000" norm="0.000000"/><LinearNorm orig="8.69081790123456e-001" norm="1.000000"/></NormContinuous></DerivedField></NeuralInput><NeuralInput id="36"><DerivedField><NormContinuous field="minwHBa" shift="3.01966043162451e-001" scale="2.15756622380441e-001"><LinearNorm orig="-1.39956790123456e+000" norm="0.000000"/><LinearNorm orig="3.23528403965610e+000" norm="1.000000"/></NormContinuous></DerivedField></NeuralInput><NeuralInput id="37"><DerivedField><NormContinuous field="minHBint2" shift="5.81039178269867e-002" scale="1.13619895821466e-001"><LinearNorm orig="-5.11388585660093e-001" norm="0.000000"/><LinearNorm orig="8.28988686676002e+000" norm="1.000000"/></NormContinuous></DerivedField></NeuralInput><NeuralInput id="38"><DerivedField><NormContinuous field="minHssNH" shift="-0.00000000000000e+000" scale="1.47574584377135e+000"><LinearNorm orig="0.00000000000000e+000" norm="0.000000"/><LinearNorm orig="6.77623456790123e-001" norm="1.000000"/></NormContinuous></DerivedField></NeuralInput><NeuralInput id="39"><DerivedField><NormContinuous field="minHdsCH" shift="-0.00000000000000e+000" scale="1.31939134251236e+000"><LinearNorm orig="0.00000000000000e+000" norm="0.000000"/><LinearNorm orig="7.57925240054869e-001" norm="1.000000"/></NormContinuous></DerivedField></NeuralInput><NeuralInput id="40"><DerivedField><NormContinuous field="minHCsats" shift="-0.00000000000000e+000" scale="1.06733668654208e+000"><LinearNorm orig="0.00000000000000e+000" norm="0.000000"/><LinearNorm orig="9.36911485015816e-001" norm="1.000000"/></NormContinuous></DerivedField></NeuralInput><NeuralInput id="41"><DerivedField><NormContinuous field="minHCsatu" shift="-0.00000000000000e+000" scale="9.60178391419969e-001"><LinearNorm orig="0.00000000000000e+000" norm="0.000000"/><LinearNorm orig="1.04147313555051e+000" norm="1.000000"/></NormContinuous></DerivedField></NeuralInput><NeuralInput id="42"><DerivedField><NormContinuous field="minHother" shift="-0.00000000000000e+000" scale="1.31485502100116e+000"><LinearNorm orig="0.00000000000000e+000" norm="0.000000"/><LinearNorm orig="7.60540123456790e-001" norm="1.000000"/></NormContinuous></DerivedField></NeuralInput><NeuralInput id="43"><DerivedField><NormContinuous field="minssCH2" shift="2.80533634802749e-001" scale="4.16422972516244e-001"><LinearNorm orig="-6.73674732946693e-001" norm="0.000000"/><LinearNorm orig="1.72772976680384e+000" norm="1.000000"/></NormContinuous></DerivedField></NeuralInput><NeuralInput id="44"><DerivedField><NormContinuous field="minaaCH" shift="-0.00000000000000e+000" scale="3.81657569375851e-001"><LinearNorm orig="0.00000000000000e+000" norm="0.000000"/><LinearNorm orig="2.62014978933960e+000" norm="1.000000"/></NormContinuous></DerivedField></NeuralInput><NeuralInput id="45"><DerivedField><NormContinuous field="minaasC" shift="2.32102060727429e-001" scale="3.93496246537005e-001"><LinearNorm orig="-5.89845679012345e-001" norm="0.000000"/><LinearNorm orig="1.95147462277091e+000" norm="1.000000"/></NormContinuous></DerivedField></NeuralInput><NeuralInput id="46"><DerivedField><NormContinuous field="minsNH2" shift="-0.00000000000000e+000" scale="1.64590935645283e-001"><LinearNorm orig="0.00000000000000e+000" norm="0.000000"/><LinearNorm orig="6.07566872427983e+000" norm="1.000000"/></NormContinuous></DerivedField></NeuralInput><NeuralInput id="47"><DerivedField><NormContinuous field="minsssN" shift="-0.00000000000000e+000" scale="3.75325803649001e-001"><LinearNorm orig="0.00000000000000e+000" norm="0.000000"/><LinearNorm orig="2.66435185185185e+000" norm="1.000000"/></NormContinuous></DerivedField></NeuralInput><NeuralInput id="48"><DerivedField><NormContinuous field="mindO" shift="-0.00000000000000e+000" scale="7.23332425725208e-002"><LinearNorm orig="0.00000000000000e+000" norm="0.000000"/><LinearNorm orig="1.38249021395302e+001" norm="1.000000"/></NormContinuous></DerivedField></NeuralInput><NeuralInput id="49"><DerivedField><NormContinuous field="minssO" shift="-0.00000000000000e+000" scale="1.52203585344402e-001"><LinearNorm orig="0.00000000000000e+000" norm="0.000000"/><LinearNorm orig="6.57014746227709e+000" norm="1.000000"/></NormContinuous></DerivedField></NeuralInput><NeuralInput id="50"><DerivedField><NormContinuous field="maxHBd" shift="-0.00000000000000e+000" scale="1.15063968819085e+000"><LinearNorm orig="0.00000000000000e+000" norm="0.000000"/><LinearNorm orig="8.69081790123456e-001" norm="1.000000"/></NormContinuous></DerivedField></NeuralInput><NeuralInput id="51"><DerivedField><NormContinuous field="maxHBa" shift="-3.99660360137496e-002" scale="7.52241155501655e-002"><LinearNorm orig="5.31292866941015e-001" norm="0.000000"/><LinearNorm orig="1.38249021395302e+001" norm="1.000000"/></NormContinuous></DerivedField></NeuralInput><NeuralInput id="52"><DerivedField><NormContinuous field="maxwHBa" shift="-0.00000000000000e+000" scale="1.84088037759979e-001"><LinearNorm orig="0.00000000000000e+000" norm="0.000000"/><LinearNorm orig="5.43218349311670e+000" norm="1.000000"/></NormContinuous></DerivedField></NeuralInput><NeuralInput id="53"><DerivedField><NormContinuous field="maxHBint2" shift="-0.00000000000000e+000" scale="1.20628907978190e-001"><LinearNorm orig="0.00000000000000e+000" norm="0.000000"/><LinearNorm orig="8.28988686676002e+000" norm="1.000000"/></NormContinuous></DerivedField></NeuralInput><NeuralInput id="54"><DerivedField><NormContinuous field="maxHssNH" shift="-0.00000000000000e+000" scale="1.47574584377135e+000"><LinearNorm orig="0.00000000000000e+000" norm="0.000000"/><LinearNorm orig="6.77623456790123e-001" norm="1.000000"/></NormContinuous></DerivedField></NeuralInput><NeuralInput id="55"><DerivedField><NormContinuous field="maxHdsCH" shift="-0.00000000000000e+000" scale="1.31939134251236e+000"><LinearNorm orig="0.00000000000000e+000" norm="0.000000"/><LinearNorm orig="7.57925240054869e-001" norm="1.000000"/></NormContinuous></DerivedField></NeuralInput><NeuralInput id="56"><DerivedField><NormContinuous field="maxHCsats" shift="-0.00000000000000e+000" scale="9.88567940546206e-001"><LinearNorm orig="0.00000000000000e+000" norm="0.000000"/><LinearNorm orig="1.01156426279359e+000" norm="1.000000"/></NormContinuous></DerivedField></NeuralInput><NeuralInput id="57"><DerivedField><NormContinuous field="maxHCsatu" shift="-0.00000000000000e+000" scale="8.71553941810767e-001"><LinearNorm orig="0.00000000000000e+000" norm="0.000000"/><LinearNorm orig="1.14737591332829e+000" norm="1.000000"/></NormContinuous></DerivedField></NeuralInput><NeuralInput id="58"><DerivedField><NormContinuous field="maxHother" shift="-0.00000000000000e+000" scale="1.27065052208442e+000"><LinearNorm orig="0.00000000000000e+000" norm="0.000000"/><LinearNorm orig="7.86998456790123e-001" norm="1.000000"/></NormContinuous></DerivedField></NeuralInput><NeuralInput id="59"><DerivedField><NormContinuous field="maxsCH3" shift="-0.00000000000000e+000" scale="4.04234200764108e-001"><LinearNorm orig="0.00000000000000e+000" norm="0.000000"/><LinearNorm orig="2.47381344307270e+000" norm="1.000000"/></NormContinuous></DerivedField></NeuralInput><NeuralInput id="60"><DerivedField><NormContinuous field="maxaaCH" shift="-0.00000000000000e+000" scale="3.79318553438257e-001"><LinearNorm orig="0.00000000000000e+000" norm="0.000000"/><LinearNorm orig="2.63630658436214e+000" norm="1.000000"/></NormContinuous></DerivedField></NeuralInput><NeuralInput id="61"><DerivedField><NormContinuous field="maxsssCH" shift="-0.00000000000000e+000" scale="7.31835863972896e-001"><LinearNorm orig="0.00000000000000e+000" norm="0.000000"/><LinearNorm orig="1.36642661179698e+000" norm="1.000000"/></NormContinuous></DerivedField></NeuralInput><NeuralInput id="62"><DerivedField><NormContinuous field="maxdssC" shift="-0.00000000000000e+000" scale="4.52236757419025e-001"><LinearNorm orig="0.00000000000000e+000" norm="0.000000"/><LinearNorm orig="2.21123113854595e+000" norm="1.000000"/></NormContinuous></DerivedField></NeuralInput><NeuralInput id="63"><DerivedField><NormContinuous field="maxaasC" shift="-0.00000000000000e+000" scale="4.81386710689228e-001"><LinearNorm orig="0.00000000000000e+000" norm="0.000000"/><LinearNorm orig="2.07733196159122e+000" norm="1.000000"/></NormContinuous></DerivedField></NeuralInput><NeuralInput id="64"><DerivedField><NormContinuous field="maxssssC" shift="-0.00000000000000e+000" scale="7.32295328980412e-001"><LinearNorm orig="0.00000000000000e+000" norm="0.000000"/><LinearNorm orig="1.36556927297668e+000" norm="1.000000"/></NormContinuous></DerivedField></NeuralInput><NeuralInput id="65"><DerivedField><NormContinuous field="maxsNH2" shift="-0.00000000000000e+000" scale="1.64590935645283e-001"><LinearNorm orig="0.00000000000000e+000" norm="0.000000"/><LinearNorm orig="6.07566872427983e+000" norm="1.000000"/></NormContinuous></DerivedField></NeuralInput><NeuralInput id="66"><DerivedField><NormContinuous field="maxsssN" shift="-0.00000000000000e+000" scale="3.75325803649001e-001"><LinearNorm orig="0.00000000000000e+000" norm="0.000000"/><LinearNorm orig="2.66435185185185e+000" norm="1.000000"/></NormContinuous></DerivedField></NeuralInput><NeuralInput id="67"><DerivedField><NormContinuous field="maxdO" shift="-0.00000000000000e+000" scale="7.23332425725208e-002"><LinearNorm orig="0.00000000000000e+000" norm="0.000000"/><LinearNorm orig="1.38249021395302e+001" norm="1.000000"/></NormContinuous></DerivedField></NeuralInput><NeuralInput id="68"><DerivedField><NormContinuous field="maxssO" shift="-0.00000000000000e+000" scale="1.52103115062408e-001"><LinearNorm orig="0.00000000000000e+000" norm="0.000000"/><LinearNorm orig="6.57448731138545e+000" norm="1.000000"/></NormContinuous></DerivedField></NeuralInput><NeuralInput id="69"><DerivedField><NormContinuous field="maxsCl" shift="-0.00000000000000e+000" scale="6.29397798402765e-001"><LinearNorm orig="0.00000000000000e+000" norm="0.000000"/><LinearNorm orig="1.58882030178326e+000" norm="1.000000"/></NormContinuous></DerivedField></NeuralInput><NeuralInput id="70"><DerivedField><NormContinuous field="gmax" shift="-1.08229467886950e-001" scale="8.01618309266824e-002"><LinearNorm orig="1.35013717421124e+000" norm="0.000000"/><LinearNorm orig="1.38249021395302e+001" norm="1.000000"/></NormContinuous></DerivedField></NeuralInput><NeuralInput id="71"><DerivedField><NormContinuous field="MAXDN2" shift="-8.69164757836136e-002" scale="2.06466059268234e-001"><LinearNorm orig="4.20972222222222e-001" norm="0.000000"/><LinearNorm orig="5.26438330656336e+000" norm="1.000000"/></NormContinuous></DerivedField></NeuralInput><NeuralInput id="72"><DerivedField><NormContinuous field="MAXDP2" shift="-6.49069604801291e-002" scale="1.56355800710497e-001"><LinearNorm orig="4.15123456790123e-001" norm="0.000000"/><LinearNorm orig="6.81079279208754e+000" norm="1.000000"/></NormContinuous></DerivedField></NeuralInput><NeuralInput id="73"><DerivedField><NormContinuous field="ETA_Shape_P" shift="-7.93678977272727e-002" scale="1.26826298701299e+000"><LinearNorm orig="6.25800000000000e-002" norm="0.000000"/><LinearNorm orig="8.51060000000000e-001" norm="1.000000"/></NormContinuous></DerivedField></NeuralInput><NeuralInput id="74"><DerivedField><NormContinuous field="ETA_BetaP" shift="-3.33333333333333e-001" scale="7.61904761904762e-001"><LinearNorm orig="4.37500000000000e-001" norm="0.000000"/><LinearNorm orig="1.75000000000000e+000" norm="1.000000"/></NormContinuous></DerivedField></NeuralInput><NeuralInput id="75"><DerivedField><NormContinuous field="ETA_BetaP_ns" shift="-0.00000000000000e+000" scale="8.38708054113444e-001"><LinearNorm orig="0.00000000000000e+000" norm="0.000000"/><LinearNorm orig="1.19231000000000e+000" norm="1.000000"/></NormContinuous></DerivedField></NeuralInput><NeuralInput id="76"><DerivedField><NormContinuous field="ETA_BetaP_ns_d" shift="-0.00000000000000e+000" scale="4.00000000000000e+000"><LinearNorm orig="0.00000000000000e+000" norm="0.000000"/><LinearNorm orig="2.50000000000000e-001" norm="1.000000"/></NormContinuous></DerivedField></NeuralInput><NeuralInput id="77"><DerivedField><NormContinuous field="FMF" shift="-0.00000000000000e+000" scale="1.55555555555556e+000"><LinearNorm orig="0.00000000000000e+000" norm="0.000000"/><LinearNorm orig="6.42857142857142e-001" norm="1.000000"/></NormContinuous></DerivedField></NeuralInput><NeuralInput id="78"><DerivedField><NormContinuous field="nHBAcc" shift="-0.00000000000000e+000" scale="1.11111111111111e-001"><LinearNorm orig="0.00000000000000e+000" norm="0.000000"/><LinearNorm orig="9.00000000000000e+000" norm="1.000000"/></NormContinuous></DerivedField></NeuralInput><NeuralInput id="79"><DerivedField><NormContinuous field="SIC5" shift="-0.00000000000000e+000" scale="1.00000000000000e+000"><LinearNorm orig="0.00000000000000e+000" norm="0.000000"/><LinearNorm orig="9.99999999999999e-001" norm="1.000000"/></NormContinuous></DerivedField></NeuralInput><NeuralInput id="80"><DerivedField><NormContinuous field="BIC5" shift="-0.00000000000000e+000" scale="1.07173999864482e+000"><LinearNorm orig="0.00000000000000e+000" norm="0.000000"/><LinearNorm orig="9.33062124455990e-001" norm="1.000000"/></NormContinuous></DerivedField></NeuralInput><NeuralInput id="81"><DerivedField><NormContinuous field="nAtomP" shift="-0.00000000000000e+000" scale="4.00000000000000e-002"><LinearNorm orig="0.00000000000000e+000" norm="0.000000"/><LinearNorm orig="2.50000000000000e+001" norm="1.000000"/></NormContinuous></DerivedField></NeuralInput><NeuralInput id="82"><DerivedField><NormContinuous field="nAtomLAC" shift="-0.00000000000000e+000" scale="5.00000000000000e-002"><LinearNorm orig="0.00000000000000e+000" norm="0.000000"/><LinearNorm orig="2.00000000000000e+001" norm="1.000000"/></NormContinuous></DerivedField></NeuralInput><NeuralInput id="83"><DerivedField><NormContinuous field="MDEO-12" shift="-0.00000000000000e+000" scale="4.65302429551051e-001"><LinearNorm orig="0.00000000000000e+000" norm="0.000000"/><LinearNorm orig="2.14913986364708e+000" norm="1.000000"/></NormContinuous></DerivedField></NeuralInput><NeuralInput id="84"><DerivedField><NormContinuous field="MLFER_S" shift="-4.31729518855657e-002" scale="2.60078023407022e-001"><LinearNorm orig="1.66000000000000e-001" norm="0.000000"/><LinearNorm orig="4.01100000000000e+000" norm="1.000000"/></NormContinuous></DerivedField></NeuralInput><NeuralInput id="85"><DerivedField><NormContinuous field="MLFER_E" shift="-4.75154153064926e-002" scale="3.62713093942691e-001"><LinearNorm orig="1.31000000000000e-001" norm="0.000000"/><LinearNorm orig="2.88800000000000e+000" norm="1.000000"/></NormContinuous></DerivedField></NeuralInput><NeuralInput id="86"><DerivedField><NormContinuous field="piPC4" shift="-0.00000000000000e+000" scale="1.72587682692228e-001"><LinearNorm orig="0.00000000000000e+000" norm="0.000000"/><LinearNorm orig="5.79415624800570e+000" norm="1.000000"/></NormContinuous></DerivedField></NeuralInput><NeuralInput id="87"><DerivedField><NormContinuous field="piPC5" shift="-0.00000000000000e+000" scale="1.52491223760283e-001"><LinearNorm orig="0.00000000000000e+000" norm="0.000000"/><LinearNorm orig="6.55775444213109e+000" norm="1.000000"/></NormContinuous></DerivedField></NeuralInput><NeuralInput id="88"><DerivedField><NormContinuous field="piPC6" shift="-0.00000000000000e+000" scale="1.41410715437847e-001"><LinearNorm orig="0.00000000000000e+000" norm="0.000000"/><LinearNorm orig="7.07159989187325e+000" norm="1.000000"/></NormContinuous></DerivedField></NeuralInput><NeuralInput id="89"><DerivedField><NormContinuous field="piPC8" shift="-0.00000000000000e+000" scale="1.21884254101035e-001"><LinearNorm orig="0.00000000000000e+000" norm="0.000000"/><LinearNorm orig="8.20450522814091e+000" norm="1.000000"/></NormContinuous></DerivedField></NeuralInput><NeuralInput id="90"><DerivedField><NormContinuous field="piPC9" shift="-0.00000000000000e+000" scale="1.13982133938273e-001"><LinearNorm orig="0.00000000000000e+000" norm="0.000000"/><LinearNorm orig="8.77330477547955e+000" norm="1.000000"/></NormContinuous></DerivedField></NeuralInput><NeuralInput id="91"><DerivedField><NormContinuous field="piPC10" shift="-0.00000000000000e+000" scale="1.10639339150100e-001"><LinearNorm orig="0.00000000000000e+000" norm="0.000000"/><LinearNorm orig="9.03837647333864e+000" norm="1.000000"/></NormContinuous></DerivedField></NeuralInput><NeuralInput id="92"><DerivedField><NormContinuous field="n6Ring" shift="-0.00000000000000e+000" scale="2.50000000000000e-001"><LinearNorm orig="0.00000000000000e+000" norm="0.000000"/><LinearNorm orig="4.00000000000000e+000" norm="1.000000"/></NormContinuous></DerivedField></NeuralInput><NeuralInput id="93"><DerivedField><NormContinuous field="RotBFrac" shift="-0.00000000000000e+000" scale="1.11111111111111e+000"><LinearNorm orig="0.00000000000000e+000" norm="0.000000"/><LinearNorm orig="9.00000000000000e-001" norm="1.000000"/></NormContinuous></DerivedField></NeuralInput><NeuralInput id="94"><DerivedField><NormContinuous field="RotBtFrac" shift="-0.00000000000000e+000" scale="1.00000000000000e+000"><LinearNorm orig="0.00000000000000e+000" norm="0.000000"/><LinearNorm orig="1.00000000000000e+000" norm="1.000000"/></NormContinuous></DerivedField></NeuralInput><NeuralInput id="95"><DerivedField><NormContinuous field="LipinskiFailures" shift="-0.00000000000000e+000" scale="5.00000000000000e-001"><LinearNorm orig="0.00000000000000e+000" norm="0.000000"/><LinearNorm orig="2.00000000000000e+000" norm="1.000000"/></NormContinuous></DerivedField></NeuralInput><NeuralInput id="96"><DerivedField><NormContinuous field="GGI6" shift="-0.00000000000000e+000" scale="8.73682542198275e-001"><LinearNorm orig="0.00000000000000e+000" norm="0.000000"/><LinearNorm orig="1.14458049886621e+000" norm="1.000000"/></NormContinuous></DerivedField></NeuralInput><NeuralInput id="97"><DerivedField><NormContinuous field="GGI8" shift="-0.00000000000000e+000" scale="2.19889196675901e+000"><LinearNorm orig="0.00000000000000e+000" norm="0.000000"/><LinearNorm orig="4.54774502393549e-001" norm="1.000000"/></NormContinuous></DerivedField></NeuralInput><NeuralInput id="98"><DerivedField><NormContinuous field="JGT" shift="-8.89538164333661e-002" scale="1.17561796099381e+000"><LinearNorm orig="7.56655813238589e-002" norm="0.000000"/><LinearNorm orig="9.26282051282051e-001" norm="1.000000"/></NormContinuous></DerivedField></NeuralInput><NeuralInput id="99"><DerivedField><NormContinuous field="WTPT-5" shift="-0.00000000000000e+000" scale="5.13831627777234e-002"><LinearNorm orig="0.00000000000000e+000" norm="0.000000"/><LinearNorm orig="1.94616280108304e+001" norm="1.000000"/></NormContinuous></DerivedField></NeuralInput></NeuralInputs><NeuralLayer numberOfNeurons="19" activationFunction="exponential"><Neuron id="100" bias="-1.73055872597543e+000"><Con from="0" weight="1.55168179206592e-001"/><Con from="1" weight="9.92539055774750e-002"/><Con from="2" weight="-3.38523734671106e-001"/><Con from="3" weight="2.46850391083489e-001"/><Con from="4" weight="-1.37262526955182e+000"/><Con from="5" weight="-1.14042594069930e+000"/><Con from="6" weight="7.68675420456099e-001"/><Con from="7" weight="4.34030239367145e-001"/><Con from="8" weight="-1.32175168759158e-001"/><Con from="9" weight="6.44348477429696e-001"/><Con from="10" weight="-1.27625694438283e+000"/><Con from="11" weight="-1.22969528912223e+000"/><Con from="12" weight="-4.30526698637164e-004"/><Con from="13" weight="-1.01571672065083e+000"/><Con from="14" weight="-5.12726262166726e-001"/><Con from="15" weight="-1.49544518564179e+000"/><Con from="16" weight="-6.08649470614665e-001"/><Con from="17" weight="-9.60320699262634e-002"/><Con from="18" weight="-1.74883254124408e-001"/><Con from="19" weight="-2.71989883306159e+000"/><Con from="20" weight="3.47872474665339e-001"/><Con from="21" weight="1.14060182142266e+000"/><Con from="22" weight="8.61975170623528e-002"/><Con from="23" weight="2.16228120625553e-001"/><Con from="24" weight="-1.03208549848894e+000"/><Con from="25" weight="-1.45795453171082e+000"/><Con from="26" weight="3.42587346120951e-001"/><Con from="27" weight="-5.47425090013160e-002"/><Con from="28" weight="-2.55374055742429e-001"/><Con from="29" weight="-1.59600938995238e-001"/><Con from="30" weight="-4.94416378387726e-001"/><Con from="31" weight="2.21149981088048e-001"/><Con from="32" weight="1.91836114895981e-001"/><Con from="33" weight="-4.23243352201570e-002"/><Con from="34" weight="-2.86120714061446e-001"/><Con from="35" weight="-5.10337424874294e-001"/><Con from="36" weight="-4.67055848172611e-001"/><Con from="37" weight="6.89018006747267e-001"/><Con from="38" weight="3.23412929348431e-002"/><Con from="39" weight="-2.86962291386930e-001"/><Con from="40" weight="-6.98418894900991e-002"/><Con from="41" weight="1.43799835692619e-001"/><Con from="42" weight="7.04405304943604e-001"/><Con from="43" weight="3.10280455783518e-001"/><Con from="44" weight="1.45909228139455e+000"/><Con from="45" weight="5.65684289568581e-001"/><Con from="46" weight="4.22996912839961e-001"/><Con from="47" weight="5.98397311182651e-001"/><Con from="48" weight="7.32820422620476e-001"/><Con from="49" weight="-1.58591919693225e-001"/><Con from="50" weight="-8.98133460943557e-001"/><Con from="51" weight="9.04106399180460e-001"/><Con from="52" weight="-9.42161022843504e-002"/><Con from="53" weight="-1.33740107108613e+000"/><Con from="54" weight="1.16476605577345e-001"/><Con from="55" weight="-3.10035755185458e-001"/><Con from="56" weight="2.00838509261374e+000"/><Con from="57" weight="3.67244276973171e-001"/><Con from="58" weight="1.83323159061600e-001"/><Con from="59" weight="9.47906588595774e-001"/><Con from="60" weight="8.95310012528188e-001"/><Con from="61" weight="-2.26342652278260e+000"/><Con from="62" weight="8.92432216232806e-001"/><Con from="63" weight="-4.00270529508634e-001"/><Con from="64" weight="4.68497838978643e-001"/><Con from="65" weight="4.42936852572242e-001"/><Con from="66" weight="-3.66878267180973e-001"/><Con from="67" weight="1.03980485896404e+000"/><Con from="68" weight="6.23492942999495e-001"/><Con from="69" weight="-6.71280366724324e-001"/><Con from="70" weight="-1.69193573388916e+000"/><Con from="71" weight="1.50330312999919e+000"/><Con from="72" weight="-1.39328108549554e+000"/><Con from="73" weight="5.46796871224910e-001"/><Con from="74" weight="-8.83516945838659e-001"/><Con from="75" weight="-1.82455683911804e+000"/><Con from="76" weight="-7.48670560473179e-001"/><Con from="77" weight="-1.24280346818521e+000"/><Con from="78" weight="5.39263708676234e-001"/><Con from="79" weight="6.01753526233463e-001"/><Con from="80" weight="3.02722732013659e-001"/><Con from="81" weight="1.13700262062153e+000"/><Con from="82" weight="-8.42576733615390e-001"/><Con from="83" weight="1.76929741789943e+000"/><Con from="84" weight="-7.01629882387278e-001"/><Con from="85" weight="-1.24926942350477e+000"/><Con from="86" weight="4.82787740953485e-001"/><Con from="87" weight="5.57470185038392e-002"/><Con from="88" weight="1.37857392323460e+000"/><Con from="89" weight="1.60147018792835e+000"/><Con from="90" weight="-1.82912583342853e+000"/><Con from="91" weight="-9.00174419329786e-001"/><Con from="92" weight="-1.33484625130952e+000"/><Con from="93" weight="-1.52257434630492e+000"/><Con from="94" weight="-1.14107371162913e+000"/><Con from="95" weight="-2.23632857038512e+000"/><Con from="96" weight="1.41304905590678e+000"/><Con from="97" weight="-5.89069317316591e-001"/><Con from="98" weight="1.90531773731100e-002"/><Con from="99" weight="4.15193344167699e-001"/></Neuron><Neuron id="101" bias="9.78701917270008e-002"><Con from="0" weight="2.06828349881174e-001"/><Con from="1" weight="-2.57098204176593e+000"/><Con from="2" weight="8.50379245564813e-001"/><Con from="3" weight="-7.73499113204121e-001"/><Con from="4" weight="5.17268403532334e-002"/><Con from="5" weight="-1.33805445049105e+000"/><Con from="6" weight="3.55137457919887e-001"/><Con from="7" weight="2.49820472877384e-001"/><Con from="8" weight="-5.54387918061061e-001"/><Con from="9" weight="4.74661444225422e-002"/><Con from="10" weight="9.02091177480542e-001"/><Con from="11" weight="-1.48424838348581e+000"/><Con from="12" weight="-1.97730314080746e+000"/><Con from="13" weight="-1.75025329257637e+000"/><Con from="14" weight="-1.06743862455085e-001"/><Con from="15" weight="-6.56710120800216e-001"/><Con from="16" weight="-5.51993136776763e-001"/><Con from="17" weight="1.04357239474440e+000"/><Con from="18" weight="-3.17165816486626e-001"/><Con from="19" weight="-2.38200038184988e-001"/><Con from="20" weight="-1.41293009289743e+000"/><Con from="21" weight="-9.36904050344984e-001"/><Con from="22" weight="-1.50310311168979e+000"/><Con from="23" weight="-1.32074371471345e+000"/><Con from="24" weight="-1.19686978094081e+000"/><Con from="25" weight="-1.86338101040097e-001"/><Con from="26" weight="-3.28519232586486e-001"/><Con from="27" weight="1.11828366415062e+000"/><Con from="28" weight="1.45437046137957e+000"/><Con from="29" weight="-2.99538422404423e-001"/><Con from="30" weight="-7.35322232832347e-001"/><Con from="31" weight="-7.61128841149960e-001"/><Con from="32" weight="3.47050536905685e-001"/><Con from="33" weight="-2.20520793420847e-002"/><Con from="34" weight="9.44738841039874e-001"/><Con from="35" weight="8.17285550356014e-001"/><Con from="36" weight="4.09310251500158e-001"/><Con from="37" weight="-1.08113971599400e-001"/><Con from="38" weight="6.03451027704019e-001"/><Con from="39" weight="5.46849577467835e-001"/><Con from="40" weight="-1.84624186173166e+000"/><Con from="41" weight="1.18672307711915e+000"/><Con from="42" weight="-1.42601656938359e+000"/><Con from="43" weight="-1.55953161735951e-001"/><Con from="44" weight="3.91507008559989e-001"/><Con from="45" weight="7.43598827506055e-001"/><Con from="46" weight="-6.27792228994868e-001"/><Con from="47" weight="-1.02893237752630e+000"/><Con from="48" weight="2.22249752270516e-001"/><Con from="49" weight="-1.14539855446896e+000"/><Con from="50" weight="1.82620119126291e-001"/><Con from="51" weight="-2.99796257823568e-001"/><Con from="52" weight="1.16869432173317e-001"/><Con from="53" weight="-2.80801318896561e-002"/><Con from="54" weight="5.78076994852436e-001"/><Con from="55" weight="7.16469272417970e-001"/><Con from="56" weight="-2.24795878344957e+000"/><Con from="57" weight="2.40195737492270e-001"/><Con from="58" weight="-1.94464170839157e-001"/><Con from="59" weight="-1.45664172504626e+000"/><Con from="60" weight="7.49968792873303e-001"/><Con from="61" weight="1.22692034676404e-001"/><Con from="62" weight="-1.26972513745890e+000"/><Con from="63" weight="-2.76626251914840e-002"/><Con from="64" weight="-1.23580801977230e+000"/><Con from="65" weight="-5.74060203478101e-001"/><Con from="66" weight="-1.12899465499937e+000"/><Con from="67" weight="-1.91961579394333e-002"/><Con from="68" weight="-1.10740641614123e+000"/><Con from="69" weight="8.84096259087358e-001"/><Con from="70" weight="-7.84640362508549e-001"/><Con from="71" weight="1.19788964759881e+000"/><Con from="72" weight="-3.13109832318302e-001"/><Con from="73" weight="-1.29430272836680e-001"/><Con from="74" weight="1.90703941713828e-001"/><Con from="75" weight="-7.80333414205269e-002"/><Con from="76" weight="5.77039127003731e-001"/><Con from="77" weight="1.02562083982904e-001"/><Con from="78" weight="5.75066973730741e-001"/><Con from="79" weight="1.84522586615520e+000"/><Con from="80" weight="1.58418294769845e+000"/><Con from="81" weight="-2.60252474063166e-001"/><Con from="82" weight="-1.34217648539333e+000"/><Con from="83" weight="5.13408752163917e-002"/><Con from="84" weight="-1.12252806164795e+000"/><Con from="85" weight="1.08602441664847e+000"/><Con from="86" weight="4.38919781944575e-001"/><Con from="87" weight="6.92400526314856e-001"/><Con from="88" weight="7.71219837843470e-001"/><Con from="89" weight="-1.60038807860959e+000"/><Con from="90" weight="1.91614978774361e+000"/><Con from="91" weight="2.75184449736472e+000"/><Con from="92" weight="-4.73874151219856e-001"/><Con from="93" weight="-1.91588745317141e+000"/><Con from="94" weight="-1.03521338867262e+000"/><Con from="95" weight="-6.10113748669472e-001"/><Con from="96" weight="-4.14800002016903e-001"/><Con from="97" weight="-7.50644674671302e-001"/><Con from="98" weight="-1.19285406372346e+000"/><Con from="99" weight="5.48892104611197e-002"/></Neuron><Neuron id="102" bias="-2.56570803601904e-001"><Con from="0" weight="-5.89704543843619e-001"/><Con from="1" weight="9.75524478154699e-001"/><Con from="2" weight="1.72056317259806e-001"/><Con from="3" weight="-1.13207949700311e-001"/><Con from="4" weight="-1.87127968493311e+000"/><Con from="5" weight="3.59336133542408e-001"/><Con from="6" weight="-5.65964859042486e-001"/><Con from="7" weight="-9.08771865724256e-002"/><Con from="8" weight="9.72422763879767e-001"/><Con from="9" weight="-9.92945522408690e-001"/><Con from="10" weight="-1.58944810393841e-002"/><Con from="11" weight="-1.29899606142096e+000"/><Con from="12" weight="-1.41828748425744e+000"/><Con from="13" weight="9.28979940321048e-001"/><Con from="14" weight="-2.57016555134703e-001"/><Con from="15" weight="7.75286486664475e-001"/><Con from="16" weight="-1.06896296622482e+000"/><Con from="17" weight="-7.53049654497155e-001"/><Con from="18" weight="-4.35705569846958e-001"/><Con from="19" weight="1.33260604946056e-001"/><Con from="20" weight="1.13534968015083e-001"/><Con from="21" weight="-1.05677563181844e+000"/><Con from="22" weight="-1.09950989265819e+000"/><Con from="23" weight="2.16249106772267e-001"/><Con from="24" weight="-7.55584158342181e-001"/><Con from="25" weight="7.01376578431262e-002"/><Con from="26" weight="4.86587605115619e-001"/><Con from="27" weight="9.01459245775352e-001"/><Con from="28" weight="8.95230431366058e-001"/><Con from="29" weight="1.18788241589453e+000"/><Con from="30" weight="-1.13609694232807e+000"/><Con from="31" weight="1.00769054554241e+000"/><Con from="32" weight="1.07703789497601e+000"/><Con from="33" weight="-1.20508444161004e+000"/><Con from="34" weight="1.14902195727584e+000"/><Con from="35" weight="-7.70379075077803e-001"/><Con from="36" weight="7.31995210732421e-001"/><Con from="37" weight="-9.28209528733587e-002"/><Con from="38" weight="-5.40786679612299e-001"/><Con from="39" weight="4.35741466719277e-001"/><Con from="40" weight="1.96005779324747e+000"/><Con from="41" weight="-1.75801801564926e+000"/><Con from="42" weight="-3.18920808000190e-001"/><Con from="43" weight="2.30988119326431e-001"/><Con from="44" weight="8.57957531932732e-001"/><Con from="45" weight="-2.06776241013720e-001"/><Con from="46" weight="-5.62805710082439e-001"/><Con from="47" weight="-5.66311670976791e-001"/><Con from="48" weight="-1.85787745331489e-001"/><Con from="49" weight="3.58146507633567e-001"/><Con from="50" weight="-8.55995828532873e-001"/><Con from="51" weight="1.09023971039392e+000"/><Con from="52" weight="-1.68849578373197e-001"/><Con from="53" weight="4.53660724051726e-001"/><Con from="54" weight="-2.44394895680133e-001"/><Con from="55" weight="3.25868230105116e-001"/><Con from="56" weight="3.77728633253096e-001"/><Con from="57" weight="-1.13943261192946e+000"/><Con from="58" weight="-7.57540217668276e-001"/><Con from="59" weight="-4.05037905491469e-001"/><Con from="60" weight="9.09775633110879e-001"/><Con from="61" weight="4.08856790310929e-001"/><Con from="62" weight="-4.64662415159525e-001"/><Con from="63" weight="-6.72391179394283e-001"/><Con from="64" weight="-9.69057774592326e-001"/><Con from="65" weight="-5.30335672404572e-001"/><Con from="66" weight="-6.98732349562133e-001"/><Con from="67" weight="-5.77001251416680e-001"/><Con from="68" weight="-3.85392178497441e-001"/><Con from="69" weight="5.04106158323574e-001"/><Con from="70" weight="-1.01150106746096e+000"/><Con from="71" weight="2.03818581942483e+000"/><Con from="72" weight="-2.97146906528239e-001"/><Con from="73" weight="7.25083820197981e-001"/><Con from="74" weight="1.25511362980622e-001"/><Con from="75" weight="6.75375318673470e-002"/><Con from="76" weight="-1.57253026165148e+000"/><Con from="77" weight="-5.36492423020329e-001"/><Con from="78" weight="3.70047148150061e-001"/><Con from="79" weight="2.72303340429584e-001"/><Con from="80" weight="2.41251094942593e-002"/><Con from="81" weight="-7.05519505931641e-001"/><Con from="82" weight="-5.32162538762210e-001"/><Con from="83" weight="1.05933615449870e+000"/><Con from="84" weight="-6.31588348923555e-001"/><Con from="85" weight="5.03082855717981e-001"/><Con from="86" weight="3.59694620065284e-002"/><Con from="87" weight="5.29873046327161e-002"/><Con from="88" weight="3.17491380027378e-001"/><Con from="89" weight="2.75283049352382e-001"/><Con from="90" weight="1.00616270111437e+000"/><Con from="91" weight="2.83114349338210e-001"/><Con from="92" weight="1.42189302293770e+000"/><Con from="93" weight="-2.20350429362739e-001"/><Con from="94" weight="5.54924869390664e-001"/><Con from="95" weight="-1.79137595614703e+000"/><Con from="96" weight="-6.13383881042726e-001"/><Con from="97" weight="6.73361565316862e-002"/><Con from="98" weight="-3.05844464412737e-001"/><Con from="99" weight="-1.10302429823897e-001"/></Neuron><Neuron id="103" bias="-1.47430162036083e-001"><Con from="0" weight="1.30113486462371e+000"/><Con from="1" weight="1.54424591279846e+000"/><Con from="2" weight="-2.25060747937528e-002"/><Con from="3" weight="-2.42054418837657e-001"/><Con from="4" weight="-4.93567735755736e-002"/><Con from="5" weight="1.91832329789171e+000"/><Con from="6" weight="-3.57360838209329e+000"/><Con from="7" weight="1.49980192355842e-001"/><Con from="8" weight="-4.41443137783659e+000"/><Con from="9" weight="1.54773264026075e+000"/><Con from="10" weight="7.24797012208529e-004"/><Con from="11" weight="3.23795023668290e-001"/><Con from="12" weight="1.21221824717210e+000"/><Con from="13" weight="1.86461245492172e+000"/><Con from="14" weight="-1.57599218897842e+000"/><Con from="15" weight="8.38521572789592e-001"/><Con from="16" weight="-1.62963950473142e+000"/><Con from="17" weight="-3.43886628722142e+000"/><Con from="18" weight="-6.27880842728697e-001"/><Con from="19" weight="2.93218857246400e+000"/><Con from="20" weight="8.88396418187825e-001"/><Con from="21" weight="-9.44597681026091e-001"/><Con from="22" weight="1.89783317519393e-001"/><Con from="23" weight="5.02860612130861e-001"/><Con from="24" weight="7.30407236926777e-001"/><Con from="25" weight="2.17775445708116e+000"/><Con from="26" weight="3.13412506872775e-001"/><Con from="27" weight="1.29034508157318e-001"/><Con from="28" weight="1.68220847196377e+000"/><Con from="29" weight="1.10850264042160e+000"/><Con from="30" weight="3.07997466342309e-001"/><Con from="31" weight="-2.39462527203954e+000"/><Con from="32" weight="2.27375695413629e+000"/><Con from="33" weight="-5.27807822130246e-001"/><Con from="34" weight="-1.66730971737199e+000"/><Con from="35" weight="-1.51466740711250e+000"/><Con from="36" weight="-1.50494617891055e+000"/><Con from="37" weight="-1.15057563639312e+000"/><Con from="38" weight="1.84738681802573e-001"/><Con from="39" weight="1.36261440186297e-001"/><Con from="40" weight="7.13595088556227e-001"/><Con from="41" weight="-1.56232696796612e-001"/><Con from="42" weight="5.49110515282293e-001"/><Con from="43" weight="-1.22776859856717e-001"/><Con from="44" weight="1.04798146725547e+000"/><Con from="45" weight="8.56706902989333e-001"/><Con from="46" weight="3.70396317321759e-001"/><Con from="47" weight="-8.23144151014227e-001"/><Con from="48" weight="-8.28323013717403e-001"/><Con from="49" weight="2.52948615996164e-001"/><Con from="50" weight="2.79608910870745e-001"/><Con from="51" weight="-2.65612626215844e-001"/><Con from="52" weight="-5.93537999470429e-001"/><Con from="53" weight="-5.95555487102629e-001"/><Con from="54" weight="2.30931708712175e-001"/><Con from="55" weight="-2.70840758747841e-001"/><Con from="56" weight="7.58486865008294e-001"/><Con from="57" weight="2.97219073989197e-001"/><Con from="58" weight="-4.46236418419302e-001"/><Con from="59" weight="-3.51265944122686e-001"/><Con from="60" weight="6.67041702652225e-001"/><Con from="61" weight="7.27827997425670e-002"/><Con from="62" weight="-9.43261492350185e-001"/><Con from="63" weight="-1.47871437197474e+000"/><Con from="64" weight="9.31200942297978e-001"/><Con from="65" weight="2.35098980346832e-001"/><Con from="66" weight="1.13645227792756e+000"/><Con from="67" weight="4.33451689773466e-001"/><Con from="68" weight="-2.99371738665683e-001"/><Con from="69" weight="-1.98079976458313e+000"/><Con from="70" weight="1.56483809822162e+000"/><Con from="71" weight="-8.79586719020101e-001"/><Con from="72" weight="-4.16538226249602e-002"/><Con from="73" weight="-2.08314416682510e-001"/><Con from="74" weight="-1.20473853782459e+000"/><Con from="75" weight="1.08680048842522e+000"/><Con from="76" weight="-3.89118663035426e+000"/><Con from="77" weight="1.88048688268542e+000"/><Con from="78" weight="-5.47890954365792e-001"/><Con from="79" weight="-5.00242727435495e-001"/><Con from="80" weight="6.03341762671015e-001"/><Con from="81" weight="-3.09388998209357e+000"/><Con from="82" weight="2.89489228284241e+000"/><Con from="83" weight="-1.44842365290878e+000"/><Con from="84" weight="-1.54194195423740e+000"/><Con from="85" weight="-2.84603156074614e-003"/><Con from="86" weight="5.36439918789903e-002"/><Con from="87" weight="9.19264536953253e-001"/><Con from="88" weight="-5.27331539780537e-001"/><Con from="89" weight="3.87381991832297e-001"/><Con from="90" weight="-4.71922820670494e-001"/><Con from="91" weight="5.57766598690430e-003"/><Con from="92" weight="-1.23496643728995e+000"/><Con from="93" weight="3.31582754901519e+000"/><Con from="94" weight="-6.98018976178836e-001"/><Con from="95" weight="-1.32235114256918e+000"/><Con from="96" weight="9.57151927644531e-001"/><Con from="97" weight="7.96836777243301e-001"/><Con from="98" weight="2.44041855511467e+000"/><Con from="99" weight="6.73337868251770e-001"/></Neuron><Neuron id="104" bias="-1.44383509890353e+000"><Con from="0" weight="1.03514317153623e+000"/><Con from="1" weight="-8.75250908816351e-001"/><Con from="2" weight="1.42075603844231e+000"/><Con from="3" weight="-9.51882692145532e-001"/><Con from="4" weight="1.61288746630074e-001"/><Con from="5" weight="1.82750930369393e+000"/><Con from="6" weight="-9.03551363804281e-001"/><Con from="7" weight="7.73349318207471e-003"/><Con from="8" weight="-6.05526323204858e-001"/><Con from="9" weight="-1.12226297519950e+000"/><Con from="10" weight="-3.17323151666226e-001"/><Con from="11" weight="6.71889904101648e-002"/><Con from="12" weight="-8.31408360336448e-001"/><Con from="13" weight="1.54689898030135e+000"/><Con from="14" weight="-1.41097980621678e+000"/><Con from="15" weight="-7.81900075010703e-001"/><Con from="16" weight="-1.01005655758349e+000"/><Con from="17" weight="-1.87248769376063e-001"/><Con from="18" weight="-2.25847755823897e-001"/><Con from="19" weight="1.00023744240486e+000"/><Con from="20" weight="-1.53821119398322e+000"/><Con from="21" weight="8.00969362294478e-003"/><Con from="22" weight="1.85679155281363e+000"/><Con from="23" weight="2.29114689741818e-002"/><Con from="24" weight="-1.41064328934187e+000"/><Con from="25" weight="5.09992957198377e-001"/><Con from="26" weight="-2.05990511990792e+000"/><Con from="27" weight="-1.70634532965513e-001"/><Con from="28" weight="8.83346653218216e-001"/><Con from="29" weight="-2.04715778239374e-001"/><Con from="30" weight="-7.39321567813252e-001"/><Con from="31" weight="-2.04301157302589e-001"/><Con from="32" weight="5.86400277211724e-002"/><Con from="33" weight="-3.70734309340430e-001"/><Con from="34" weight="-2.22967372142554e-001"/><Con from="35" weight="-5.44598363594323e-001"/><Con from="36" weight="-1.88960543192820e+000"/><Con from="37" weight="-1.48117216309329e+000"/><Con from="38" weight="6.96728620609843e-001"/><Con from="39" weight="6.28448637560700e-001"/><Con from="40" weight="1.27848562952572e+000"/><Con from="41" weight="2.23741675967058e+000"/><Con from="42" weight="-1.91514266209902e-001"/><Con from="43" weight="4.71240778716252e-001"/><Con from="44" weight="3.08284752921722e-001"/><Con from="45" weight="-6.58179385224616e-001"/><Con from="46" weight="2.36640643216909e-001"/><Con from="47" weight="-1.13690138166493e+000"/><Con from="48" weight="1.17986065192832e+000"/><Con from="49" weight="-6.25206862182890e-002"/><Con from="50" weight="-6.67678140136260e-001"/><Con from="51" weight="1.06899234275239e+000"/><Con from="52" weight="6.97757848928933e-001"/><Con from="53" weight="-6.85937318036455e-001"/><Con from="54" weight="6.71217260225386e-001"/><Con from="55" weight="9.85373012974858e-001"/><Con from="56" weight="8.84258732874698e-001"/><Con from="57" weight="2.49840748109035e+000"/><Con from="58" weight="-2.01270729896903e-001"/><Con from="59" weight="-8.51679203644004e-001"/><Con from="60" weight="2.49639138460565e-001"/><Con from="61" weight="8.41512044552934e-001"/><Con from="62" weight="-8.83196041864641e-001"/><Con from="63" weight="-5.93952107099252e-001"/><Con from="64" weight="-9.87882972140326e-001"/><Con from="65" weight="2.45269158635593e-001"/><Con from="66" weight="-9.45373072240086e-001"/><Con from="67" weight="1.21672425054948e+000"/><Con from="68" weight="-5.80761552439072e-001"/><Con from="69" weight="-2.36523793557415e-001"/><Con from="70" weight="1.40907027810127e+000"/><Con from="71" weight="1.03834572334018e+000"/><Con from="72" weight="1.33924203800817e+000"/><Con from="73" weight="2.43957542330698e-001"/><Con from="74" weight="1.35490117556087e-001"/><Con from="75" weight="4.45601924696887e-001"/><Con from="76" weight="-1.34123712276215e+000"/><Con from="77" weight="-7.70732448535071e-001"/><Con from="78" weight="9.72508959241114e-001"/><Con from="79" weight="-1.64555454132589e+000"/><Con from="80" weight="-1.51259280879936e+000"/><Con from="81" weight="-4.27618027666119e-001"/><Con from="82" weight="1.86329190138827e-001"/><Con from="83" weight="-8.62390533341561e-001"/><Con from="84" weight="-8.04228731264556e-002"/><Con from="85" weight="-8.17240214264778e-001"/><Con from="86" weight="7.95683257816758e-001"/><Con from="87" weight="-6.79250805785716e-001"/><Con from="88" weight="-7.45690756319527e-001"/><Con from="89" weight="2.52572989249155e-001"/><Con from="90" weight="-5.91519298372893e-001"/><Con from="91" weight="7.99446323516126e-001"/><Con from="92" weight="-1.68140015612792e+000"/><Con from="93" weight="-8.77271568461724e-001"/><Con from="94" weight="-1.15797495037300e+000"/><Con from="95" weight="-4.42846274742833e-001"/><Con from="96" weight="-2.85007799010305e-001"/><Con from="97" weight="5.45654008013221e-001"/><Con from="98" weight="1.64847582597334e+000"/><Con from="99" weight="7.98296343631730e-001"/></Neuron><Neuron id="105" bias="-7.46285805164720e-001"><Con from="0" weight="-2.10469397663116e-001"/><Con from="1" weight="-1.00402135177759e+000"/><Con from="2" weight="9.57277224851605e-001"/><Con from="3" weight="1.21391263783715e+000"/><Con from="4" weight="-1.12977402878036e+000"/><Con from="5" weight="1.42903640052567e+000"/><Con from="6" weight="-8.85901759129002e-001"/><Con from="7" weight="-8.04126133787252e-001"/><Con from="8" weight="-1.66349070580090e+000"/><Con from="9" weight="-7.95581791158799e-001"/><Con from="10" weight="3.79544857873442e-001"/><Con from="11" weight="-1.56530707861432e-001"/><Con from="12" weight="-5.60331218552823e-001"/><Con from="13" weight="6.37423092653092e-001"/><Con from="14" weight="-1.05455339769775e+000"/><Con from="15" weight="-1.52606413134949e-001"/><Con from="16" weight="-2.60904045209976e-001"/><Con from="17" weight="1.19199382057077e+000"/><Con from="18" weight="-1.22483798625964e+000"/><Con from="19" weight="1.16094647832334e+000"/><Con from="20" weight="-1.77764342975342e+000"/><Con from="21" weight="1.99265995083299e+000"/><Con from="22" weight="1.51666434059761e+000"/><Con from="23" weight="-3.53145163332048e-001"/><Con from="24" weight="-2.44297444009853e-003"/><Con from="25" weight="5.76828866403291e-002"/><Con from="26" weight="-1.11757070664191e+000"/><Con from="27" weight="1.07325915058261e-001"/><Con from="28" weight="9.06853833715569e-001"/><Con from="29" weight="5.37593137377175e-001"/><Con from="30" weight="-2.27964016637091e-001"/><Con from="31" weight="2.25529091711524e-002"/><Con from="32" weight="-6.33838448883402e-001"/><Con from="33" weight="-6.94444203839659e-001"/><Con from="34" weight="1.75811797567091e-001"/><Con from="35" weight="-1.07752152850262e+000"/><Con from="36" weight="-2.83726308990041e+000"/><Con from="37" weight="-1.19593639335868e+000"/><Con from="38" weight="-1.85959852654689e-001"/><Con from="39" weight="2.43222996792078e-001"/><Con from="40" weight="2.51552634548577e-001"/><Con from="41" weight="7.45197226662590e-001"/><Con from="42" weight="5.11324434124824e-001"/><Con from="43" weight="1.31301938035959e+000"/><Con from="44" weight="-2.88429783356423e-001"/><Con from="45" weight="-6.38471591850131e-001"/><Con from="46" weight="-7.10506465883079e-001"/><Con from="47" weight="1.63839861534552e+000"/><Con from="48" weight="-4.28341441147871e-001"/><Con from="49" weight="6.47826641960725e-001"/><Con from="50" weight="-8.32608588598389e-001"/><Con from="51" weight="-3.74861370921941e-001"/><Con from="52" weight="-7.23274242911831e-001"/><Con from="53" weight="9.43206979526420e-001"/><Con from="54" weight="-3.08060272430609e-001"/><Con from="55" weight="1.13584481236434e-001"/><Con from="56" weight="-1.73425989006443e+000"/><Con from="57" weight="8.46877756020414e-001"/><Con from="58" weight="-3.43189158481053e-001"/><Con from="59" weight="3.02351506796951e-001"/><Con from="60" weight="-1.10811685797041e+000"/><Con from="61" weight="7.58819545400525e-001"/><Con from="62" weight="-9.57642124852019e-002"/><Con from="63" weight="-2.61293097008375e-001"/><Con from="64" weight="-2.18817516311382e+000"/><Con from="65" weight="-6.90575646716209e-001"/><Con from="66" weight="1.96501467204351e+000"/><Con from="67" weight="3.16275988111602e-001"/><Con from="68" weight="4.69929830688845e-001"/><Con from="69" weight="-2.05372080476230e-001"/><Con from="70" weight="-1.21275875048678e-002"/><Con from="71" weight="-9.97491198361375e-001"/><Con from="72" weight="2.71165960415283e+000"/><Con from="73" weight="6.78717050360154e-001"/><Con from="74" weight="3.22722949371594e-001"/><Con from="75" weight="1.15771729550249e+000"/><Con from="76" weight="1.45986955671985e+000"/><Con from="77" weight="-3.23529835595777e-002"/><Con from="78" weight="-1.33775263354534e+000"/><Con from="79" weight="8.08085624712911e-001"/><Con from="80" weight="1.02763457846112e+000"/><Con from="81" weight="7.76379857799507e-001"/><Con from="82" weight="-5.12096116237275e-001"/><Con from="83" weight="7.87448484755200e-001"/><Con from="84" weight="-1.42573236024242e+000"/><Con from="85" weight="-1.14140630970310e+000"/><Con from="86" weight="-3.28397709994876e-001"/><Con from="87" weight="-4.15551294560228e-001"/><Con from="88" weight="-9.53621342224624e-001"/><Con from="89" weight="-2.03634057428565e+000"/><Con from="90" weight="-1.56313398653834e+000"/><Con from="91" weight="1.87877467624833e+000"/><Con from="92" weight="1.12634851029176e+000"/><Con from="93" weight="7.28460772985316e-001"/><Con from="94" weight="3.74452179744198e-001"/><Con from="95" weight="2.60985022312328e-001"/><Con from="96" weight="-1.11648993767521e+000"/><Con from="97" weight="3.66960129745967e+000"/><Con from="98" weight="4.15690188869898e-001"/><Con from="99" weight="-5.99334544696982e-001"/></Neuron><Neuron id="106" bias="-1.50267338187038e+000"><Con from="0" weight="-3.23121028347368e-001"/><Con from="1" weight="2.19447801225538e-002"/><Con from="2" weight="-6.53410603802865e-001"/><Con from="3" weight="2.94175564655287e-001"/><Con from="4" weight="-1.39635069359913e+000"/><Con from="5" weight="6.58503183775248e-001"/><Con from="6" weight="-9.77274815480922e-001"/><Con from="7" weight="4.95615899838338e-001"/><Con from="8" weight="-2.02518637148685e+000"/><Con from="9" weight="-1.54725680740307e+000"/><Con from="10" weight="1.45613656965929e-001"/><Con from="11" weight="-1.03981358665067e+000"/><Con from="12" weight="1.70316154926452e+000"/><Con from="13" weight="6.56255163472798e-001"/><Con from="14" weight="-1.52225005139598e-001"/><Con from="15" weight="-1.37301695503072e+000"/><Con from="16" weight="-1.76567148706186e+000"/><Con from="17" weight="2.33580434784839e-001"/><Con from="18" weight="-4.94980658338747e-001"/><Con from="19" weight="7.64730029524837e-001"/><Con from="20" weight="-2.18150741372439e+000"/><Con from="21" weight="1.33046359946313e+000"/><Con from="22" weight="8.41356369265782e-001"/><Con from="23" weight="4.46374894281263e-001"/><Con from="24" weight="2.42013413226740e-001"/><Con from="25" weight="-1.59484610819927e+000"/><Con from="26" weight="-3.02167970045770e-001"/><Con from="27" weight="6.69877670818693e-001"/><Con from="28" weight="-9.63326904150157e-002"/><Con from="29" weight="1.49024289138027e+000"/><Con from="30" weight="5.46253702768479e-001"/><Con from="31" weight="-1.06846028549898e+000"/><Con from="32" weight="6.84260042299217e-001"/><Con from="33" weight="2.77548127291028e-001"/><Con from="34" weight="5.98519248569200e-001"/><Con from="35" weight="-1.23288319264867e+000"/><Con from="36" weight="-1.55111876603936e+000"/><Con from="37" weight="-8.64805160239393e-001"/><Con from="38" weight="4.11953107757106e-001"/><Con from="39" weight="-5.53581176590133e-001"/><Con from="40" weight="-1.77124674910169e+000"/><Con from="41" weight="-2.61757970280257e-001"/><Con from="42" weight="2.90982857096161e+000"/><Con from="43" weight="-2.46571744635661e-002"/><Con from="44" weight="1.53915912831243e+000"/><Con from="45" weight="1.12932664665484e-001"/><Con from="46" weight="-7.35355624650265e-001"/><Con from="47" weight="-1.86177200554124e+000"/><Con from="48" weight="2.65429030125896e-001"/><Con from="49" weight="-6.12886761553292e-001"/><Con from="50" weight="-1.07407056842633e+000"/><Con from="51" weight="6.72884228344197e-001"/><Con from="52" weight="-1.98798299744789e+000"/><Con from="53" weight="-1.22574337909177e+000"/><Con from="54" weight="4.23760466658959e-001"/><Con from="55" weight="-9.37565423682210e-001"/><Con from="56" weight="-6.70697764723174e-001"/><Con from="57" weight="-4.87644546610321e-001"/><Con from="58" weight="4.46670261088143e-001"/><Con from="59" weight="5.08779767238797e-001"/><Con from="60" weight="-2.88324581658531e-002"/><Con from="61" weight="6.04162137815041e-001"/><Con from="62" weight="-5.07285653549419e-001"/><Con from="63" weight="7.83400993274358e-001"/><Con from="64" weight="-2.68728942255857e-002"/><Con from="65" weight="-7.82758244866914e-001"/><Con from="66" weight="-1.80178215783254e+000"/><Con from="67" weight="1.28562392618278e+000"/><Con from="68" weight="-1.92073982670969e+000"/><Con from="69" weight="-1.81912543060160e-001"/><Con from="70" weight="3.56035994731549e-001"/><Con from="71" weight="-1.57556337566001e+000"/><Con from="72" weight="1.78058735899709e+000"/><Con from="73" weight="-1.74167222081772e-001"/><Con from="74" weight="-1.44091091772806e+000"/><Con from="75" weight="-2.44523959992520e-001"/><Con from="76" weight="-7.69312915754121e-002"/><Con from="77" weight="8.09064187751996e-001"/><Con from="78" weight="-1.85690314132475e+000"/><Con from="79" weight="5.44560062020009e-001"/><Con from="80" weight="8.95070036739233e-001"/><Con from="81" weight="2.26680499113096e+000"/><Con from="82" weight="5.38206963236462e-001"/><Con from="83" weight="-9.74349260458684e-001"/><Con from="84" weight="1.27885445046806e+000"/><Con from="85" weight="-1.39358153646333e+000"/><Con from="86" weight="-1.42561061048478e+000"/><Con from="87" weight="-6.67073081039468e-001"/><Con from="88" weight="-6.68555232622407e-001"/><Con from="89" weight="1.53025076488221e+000"/><Con from="90" weight="1.09632628637689e+000"/><Con from="91" weight="-4.61371931735188e-001"/><Con from="92" weight="1.45560606920299e-001"/><Con from="93" weight="6.87313541387708e-001"/><Con from="94" weight="3.27747746701053e-001"/><Con from="95" weight="-3.47429332730290e-001"/><Con from="96" weight="-1.97904521093573e-001"/><Con from="97" weight="-2.00962128214962e+000"/><Con from="98" weight="4.10798869635094e-002"/><Con from="99" weight="-4.59031539127387e-001"/></Neuron><Neuron id="107" bias="-8.97171425253951e-002"><Con from="0" weight="-5.44434710844658e-001"/><Con from="1" weight="2.03071195449145e+000"/><Con from="2" weight="1.53583522238310e+000"/><Con from="3" weight="7.37819355569384e-001"/><Con from="4" weight="-1.01110785875100e+000"/><Con from="5" weight="-2.92909437387187e+000"/><Con from="6" weight="1.26414673785684e-001"/><Con from="7" weight="-1.43933671094994e+000"/><Con from="8" weight="7.31932475127527e-001"/><Con from="9" weight="-8.49253894619057e-001"/><Con from="10" weight="-1.72440603099162e+000"/><Con from="11" weight="2.23596801029937e+000"/><Con from="12" weight="2.47941467091958e+000"/><Con from="13" weight="1.60492104608651e+000"/><Con from="14" weight="-3.29624301109645e-002"/><Con from="15" weight="5.00981373216000e-001"/><Con from="16" weight="-8.76260192307780e-001"/><Con from="17" weight="6.03637543399976e-001"/><Con from="18" weight="-7.61960219125451e-001"/><Con from="19" weight="-2.21800937454113e+000"/><Con from="20" weight="-3.04632711586565e+000"/><Con from="21" weight="-5.19211553032154e-001"/><Con from="22" weight="-5.97649807448879e-001"/><Con from="23" weight="7.72889770174433e-001"/><Con from="24" weight="1.27024956069953e+000"/><Con from="25" weight="-6.10489723530148e-001"/><Con from="26" weight="-1.79970476128170e+000"/><Con from="27" weight="-2.89325526853758e+000"/><Con from="28" weight="1.94282709027860e+000"/><Con from="29" weight="-1.25292347575253e+000"/><Con from="30" weight="4.13698551511912e-001"/><Con from="31" weight="2.38837269004516e+000"/><Con from="32" weight="1.67313755318126e+000"/><Con from="33" weight="3.10809928673401e+000"/><Con from="34" weight="-1.35571468272494e+000"/><Con from="35" weight="-9.04120069436751e-001"/><Con from="36" weight="-2.89799356127450e+000"/><Con from="37" weight="-1.80766103944782e-001"/><Con from="38" weight="-6.19000853641857e-001"/><Con from="39" weight="-2.24115417509797e-001"/><Con from="40" weight="-9.89157523306226e-001"/><Con from="41" weight="3.00808601497528e+000"/><Con from="42" weight="2.31669225002686e-001"/><Con from="43" weight="-1.72342714564097e+000"/><Con from="44" weight="9.61060172442859e-001"/><Con from="45" weight="-3.27260486366162e-001"/><Con from="46" weight="-2.75869146873173e-001"/><Con from="47" weight="3.07922508962928e-001"/><Con from="48" weight="1.26083906671267e+000"/><Con from="49" weight="7.42588816655871e-001"/><Con from="50" weight="-1.14889585948174e+000"/><Con from="51" weight="6.36955313509538e-001"/><Con from="52" weight="-1.23644352277577e-001"/><Con from="53" weight="-5.90528296745610e-001"/><Con from="54" weight="-6.18540575892611e-001"/><Con from="55" weight="6.27701284839989e-001"/><Con from="56" weight="1.15408841695962e-001"/><Con from="57" weight="3.79242170483532e-001"/><Con from="58" weight="4.82001340249618e-001"/><Con from="59" weight="-9.26600159827948e-001"/><Con from="60" weight="9.05057182802194e-001"/><Con from="61" weight="5.42830896158759e-001"/><Con from="62" weight="-1.55483291743984e+000"/><Con from="63" weight="1.06942709514267e+000"/><Con from="64" weight="2.76313444961711e+000"/><Con from="65" weight="-2.59881314038408e-001"/><Con from="66" weight="2.38867497323030e-001"/><Con from="67" weight="1.62815715665578e+000"/><Con from="68" weight="1.21563936342057e+000"/><Con from="69" weight="-1.43688825703097e+000"/><Con from="70" weight="-7.46969273287392e-001"/><Con from="71" weight="-1.11567356842438e+000"/><Con from="72" weight="-4.56836384757042e-001"/><Con from="73" weight="-5.01232302419756e-001"/><Con from="74" weight="-1.86806899428455e+000"/><Con from="75" weight="-2.18628355548615e+000"/><Con from="76" weight="-1.26623194848435e+000"/><Con from="77" weight="-2.69701410160093e+000"/><Con from="78" weight="-1.78203146698456e+000"/><Con from="79" weight="-1.64800414733112e+000"/><Con from="80" weight="-1.70840251252125e+000"/><Con from="81" weight="-2.62599800164942e+000"/><Con from="82" weight="-5.51254756228037e-001"/><Con from="83" weight="-3.33706871056990e+000"/><Con from="84" weight="1.46775009921305e+000"/><Con from="85" weight="-3.32056981867989e-001"/><Con from="86" weight="-5.36994933952728e-001"/><Con from="87" weight="-1.28304452021830e+000"/><Con from="88" weight="-1.30811069289927e+000"/><Con from="89" weight="2.99030157382744e+000"/><Con from="90" weight="4.62978144917543e-002"/><Con from="91" weight="-5.94287855199590e-001"/><Con from="92" weight="1.44717542052603e+000"/><Con from="93" weight="7.97118876706287e-001"/><Con from="94" weight="1.16460851925638e+000"/><Con from="95" weight="-2.29035682958310e-001"/><Con from="96" weight="1.37576492067616e+000"/><Con from="97" weight="8.50454066085218e-001"/><Con from="98" weight="2.65757190067360e+000"/><Con from="99" weight="-6.17195248275383e-001"/></Neuron><Neuron id="108" bias="-5.92571966952557e-001"><Con from="0" weight="4.21210532766121e-001"/><Con from="1" weight="-5.58917444144386e-001"/><Con from="2" weight="1.43490510183902e-001"/><Con from="3" weight="-4.20702670380020e-001"/><Con from="4" weight="3.58106087483357e-001"/><Con from="5" weight="-6.81722664305009e-001"/><Con from="6" weight="-4.47776371845087e-002"/><Con from="7" weight="-1.10759070099785e-001"/><Con from="8" weight="-6.98565389787787e-001"/><Con from="9" weight="3.73368684619340e-001"/><Con from="10" weight="-7.54034827339345e-001"/><Con from="11" weight="-9.83134134483126e-001"/><Con from="12" weight="-4.80516635457829e-001"/><Con from="13" weight="4.46959420571457e-001"/><Con from="14" weight="1.44793814358027e+000"/><Con from="15" weight="-4.53412198798511e-001"/><Con from="16" weight="-3.62351884815668e-001"/><Con from="17" weight="-8.11300887863677e-001"/><Con from="18" weight="3.24806424452700e-001"/><Con from="19" weight="-2.49729905251392e-002"/><Con from="20" weight="3.65763357320672e-001"/><Con from="21" weight="-1.61084312149181e-001"/><Con from="22" weight="-1.08586018921247e+000"/><Con from="23" weight="-1.51724887054022e+000"/><Con from="24" weight="1.40005752209888e+000"/><Con from="25" weight="8.17075609549595e-001"/><Con from="26" weight="1.97402086934334e-001"/><Con from="27" weight="-2.44494235216593e-001"/><Con from="28" weight="-7.03079777004287e-001"/><Con from="29" weight="-1.14574520836615e+000"/><Con from="30" weight="-5.70582045318211e-001"/><Con from="31" weight="-5.49096386704244e-001"/><Con from="32" weight="-1.49503502584012e+000"/><Con from="33" weight="-4.90038157003540e-001"/><Con from="34" weight="-8.75273058704509e-001"/><Con from="35" weight="3.74143870583438e-001"/><Con from="36" weight="4.50451595830552e-001"/><Con from="37" weight="2.34472860045737e-001"/><Con from="38" weight="6.28212185289575e-001"/><Con from="39" weight="1.50643625778891e-001"/><Con from="40" weight="-6.76896397157607e-001"/><Con from="41" weight="-1.41651669516336e+000"/><Con from="42" weight="4.17757739683471e-001"/><Con from="43" weight="9.36031227516796e-001"/><Con from="44" weight="5.80521416120754e-001"/><Con from="45" weight="-2.08861458873258e-001"/><Con from="46" weight="-4.18637179104670e-001"/><Con from="47" weight="-5.58126650833098e-001"/><Con from="48" weight="-7.85976186514950e-001"/><Con from="49" weight="-5.10240493164641e-001"/><Con from="50" weight="6.04138581551389e-002"/><Con from="51" weight="-1.03641594757060e+000"/><Con from="52" weight="1.35131571180922e-001"/><Con from="53" weight="-8.90869174943298e-002"/><Con from="54" weight="5.86961447111105e-001"/><Con from="55" weight="1.37841470215529e-001"/><Con from="56" weight="-4.02270784755585e-001"/><Con from="57" weight="-1.28957928380218e+000"/><Con from="58" weight="-9.29446483962563e-002"/><Con from="59" weight="-8.66224625862151e-002"/><Con from="60" weight="3.10967407109488e-001"/><Con from="61" weight="8.16540227673399e-001"/><Con from="62" weight="-1.99448888868225e-001"/><Con from="63" weight="-1.48832541068970e-001"/><Con from="64" weight="-8.95667400079132e-001"/><Con from="65" weight="-4.35109162146858e-001"/><Con from="66" weight="-7.00558007416118e-001"/><Con from="67" weight="-6.45508092205001e-001"/><Con from="68" weight="-5.16940764455345e-001"/><Con from="69" weight="4.44235532503984e-002"/><Con from="70" weight="4.75459472974788e-001"/><Con from="71" weight="-5.21458895664242e-001"/><Con from="72" weight="-4.37589101862584e-001"/><Con from="73" weight="-2.84649973303231e-001"/><Con from="74" weight="5.34625729604784e-001"/><Con from="75" weight="7.36802334286271e-001"/><Con from="76" weight="-6.04967654709851e-001"/><Con from="77" weight="1.26791904594164e-001"/><Con from="78" weight="-2.30844011762806e-001"/><Con from="79" weight="-5.68491506479081e-001"/><Con from="80" weight="-6.09278231139368e-001"/><Con from="81" weight="-2.79557983129992e-001"/><Con from="82" weight="-3.65743499775859e-001"/><Con from="83" weight="-5.87026693255935e-001"/><Con from="84" weight="-3.73818764831765e-001"/><Con from="85" weight="-3.99340515299844e-001"/><Con from="86" weight="-4.73437399467426e-001"/><Con from="87" weight="-5.99317888109723e-002"/><Con from="88" weight="6.28172299992669e-001"/><Con from="89" weight="-2.98662929813214e-001"/><Con from="90" weight="8.14264491113276e-001"/><Con from="91" weight="9.31505891944285e-001"/><Con from="92" weight="3.77199472641660e-001"/><Con from="93" weight="-3.79982841000575e-001"/><Con from="94" weight="-7.34943396670982e-001"/><Con from="95" weight="-6.37033736679339e-001"/><Con from="96" weight="-3.45714378418229e-001"/><Con from="97" weight="1.50516616622297e-001"/><Con from="98" weight="-6.59055360033229e-002"/><Con from="99" weight="3.50177655091496e-001"/></Neuron><Neuron id="109" bias="-3.55993398250784e-001"><Con from="0" weight="-1.14488665130300e+000"/><Con from="1" weight="-3.32097314189281e-001"/><Con from="2" weight="-8.24159592215163e-002"/><Con from="3" weight="3.62531206091317e-001"/><Con from="4" weight="9.07400560818193e-001"/><Con from="5" weight="-1.30857014400243e+000"/><Con from="6" weight="1.01768595769417e-001"/><Con from="7" weight="4.57742274993231e-001"/><Con from="8" weight="6.10806641947498e-001"/><Con from="9" weight="3.28871222488734e-001"/><Con from="10" weight="3.91419885239146e-001"/><Con from="11" weight="-1.15202682831477e+000"/><Con from="12" weight="-1.03409080367601e+000"/><Con from="13" weight="-1.51912659530941e+000"/><Con from="14" weight="-1.28600225221655e+000"/><Con from="15" weight="-1.39914381257617e-001"/><Con from="16" weight="-1.19864956941225e-001"/><Con from="17" weight="1.68780169260005e+000"/><Con from="18" weight="6.66778930436376e-001"/><Con from="19" weight="1.53465177494451e+000"/><Con from="20" weight="-2.25281027137650e-001"/><Con from="21" weight="-1.12303491052071e-001"/><Con from="22" weight="3.00405949209152e-001"/><Con from="23" weight="-1.43034525562875e+000"/><Con from="24" weight="-6.03225403995072e-001"/><Con from="25" weight="6.55161889628342e-002"/><Con from="26" weight="6.03208322845244e-001"/><Con from="27" weight="-1.03239793268981e+000"/><Con from="28" weight="7.28655880076045e-001"/><Con from="29" weight="-6.95979620319534e-001"/><Con from="30" weight="2.32344054264386e+000"/><Con from="31" weight="7.84886967238264e-001"/><Con from="32" weight="3.54930837991947e-001"/><Con from="33" weight="1.44505213165513e+000"/><Con from="34" weight="-2.85870429049767e-001"/><Con from="35" weight="1.05279147763906e-001"/><Con from="36" weight="1.38438726070831e+000"/><Con from="37" weight="-8.64477290152170e-001"/><Con from="38" weight="6.21695975920625e-001"/><Con from="39" weight="8.08067172136760e-001"/><Con from="40" weight="1.36005201780229e+000"/><Con from="41" weight="2.92613644131454e+000"/><Con from="42" weight="-7.13309876402562e-001"/><Con from="43" weight="1.88465264194485e-002"/><Con from="44" weight="2.58707624728835e-001"/><Con from="45" weight="-1.12794072765318e+000"/><Con from="46" weight="8.64214921299881e-001"/><Con from="47" weight="-1.65685604684831e-001"/><Con from="48" weight="-7.87827963307684e-002"/><Con from="49" weight="5.67577353633473e-002"/><Con from="50" weight="3.96832569895103e-001"/><Con from="51" weight="-3.60165546813484e-001"/><Con from="52" weight="3.42290480094768e-001"/><Con from="53" weight="9.72362998171486e-001"/><Con from="54" weight="2.20358888355824e-001"/><Con from="55" weight="6.83046666375656e-001"/><Con from="56" weight="-1.15229870808037e+000"/><Con from="57" weight="1.49276575620846e-001"/><Con from="58" weight="-4.70131189793092e-001"/><Con from="59" weight="-6.11091703101714e-001"/><Con from="60" weight="4.72030429476363e-001"/><Con from="61" weight="-1.60726762029750e-001"/><Con from="62" weight="5.75232864993463e-001"/><Con from="63" weight="6.78293163830754e-001"/><Con from="64" weight="-6.25080569896985e-001"/><Con from="65" weight="8.53364786848253e-001"/><Con from="66" weight="4.93142172362493e-001"/><Con from="67" weight="-4.49249130458133e-002"/><Con from="68" weight="6.44435145964568e-002"/><Con from="69" weight="-4.45200199509532e-001"/><Con from="70" weight="-4.66337195208476e-001"/><Con from="71" weight="1.21746574762380e+000"/><Con from="72" weight="8.43872360338554e-001"/><Con from="73" weight="4.87516093918418e-002"/><Con from="74" weight="-7.50313968327454e-001"/><Con from="75" weight="9.22973687437201e-002"/><Con from="76" weight="-1.20668638379223e-001"/><Con from="77" weight="-2.20946394305897e-001"/><Con from="78" weight="-3.79339958134504e-002"/><Con from="79" weight="-5.49345806806409e-001"/><Con from="80" weight="-3.84842854303746e-001"/><Con from="81" weight="-4.75828683387103e-002"/><Con from="82" weight="-1.74779371548237e+000"/><Con from="83" weight="-1.99518069428699e-001"/><Con from="84" weight="-1.71812220267397e+000"/><Con from="85" weight="3.30695047861505e-001"/><Con from="86" weight="1.93127835895334e-002"/><Con from="87" weight="9.04267854528954e-001"/><Con from="88" weight="1.98919177369551e-001"/><Con from="89" weight="9.98025611073920e-001"/><Con from="90" weight="-4.38888960189815e-001"/><Con from="91" weight="9.09993855034476e-001"/><Con from="92" weight="-6.72591280233071e-001"/><Con from="93" weight="-3.56746714853989e-001"/><Con from="94" weight="1.95146738948797e-001"/><Con from="95" weight="-6.39766031451245e-001"/><Con from="96" weight="-4.19509303155417e-001"/><Con from="97" weight="-6.16943133594814e-001"/><Con from="98" weight="-1.88269254665904e+000"/><Con from="99" weight="-1.07285513625212e+000"/></Neuron><Neuron id="110" bias="-1.39869011432797e+000"><Con from="0" weight="-7.72231124090771e-001"/><Con from="1" weight="-2.87742889933126e-002"/><Con from="2" weight="5.53946923007552e-001"/><Con from="3" weight="-6.57064821733434e-001"/><Con from="4" weight="-5.31890703385849e-001"/><Con from="5" weight="7.59870015960225e-001"/><Con from="6" weight="-1.64504134358799e+000"/><Con from="7" weight="-3.03349357818858e-001"/><Con from="8" weight="1.25975020132796e-001"/><Con from="9" weight="-2.99821184515735e-001"/><Con from="10" weight="-6.74922218721327e-001"/><Con from="11" weight="-1.74789876056858e+000"/><Con from="12" weight="-1.12305437920485e+000"/><Con from="13" weight="5.96863063379591e-001"/><Con from="14" weight="7.01062975629835e-001"/><Con from="15" weight="-2.02112274757177e-001"/><Con from="16" weight="-1.14375076902859e+000"/><Con from="17" weight="1.65497770877074e+000"/><Con from="18" weight="9.93718195245044e-001"/><Con from="19" weight="-7.42886614930595e-001"/><Con from="20" weight="-9.76619703589816e-001"/><Con from="21" weight="5.24919566492059e-001"/><Con from="22" weight="-2.02177547318984e-001"/><Con from="23" weight="-1.79751220390739e+000"/><Con from="24" weight="2.70531010413417e-001"/><Con from="25" weight="1.44326747844521e+000"/><Con from="26" weight="-8.85261729812041e-001"/><Con from="27" weight="-7.87840785075469e-001"/><Con from="28" weight="1.63083607814664e+000"/><Con from="29" weight="-1.57711246349827e+000"/><Con from="30" weight="-1.79391272138936e+000"/><Con from="31" weight="-9.76030273425562e-001"/><Con from="32" weight="8.52501052736711e-002"/><Con from="33" weight="-5.22785389986936e-001"/><Con from="34" weight="-6.62852724348314e-001"/><Con from="35" weight="-1.30778801263792e+000"/><Con from="36" weight="1.41061160200771e+000"/><Con from="37" weight="-1.54771486710200e+000"/><Con from="38" weight="2.96423497689760e-001"/><Con from="39" weight="2.52498272530182e-001"/><Con from="40" weight="1.18305693174549e+000"/><Con from="41" weight="1.51765522148419e+000"/><Con from="42" weight="8.70618366017996e-001"/><Con from="43" weight="-6.54729957361089e-001"/><Con from="44" weight="-4.07457217995388e-001"/><Con from="45" weight="4.77766169989891e-002"/><Con from="46" weight="1.21278937619263e-001"/><Con from="47" weight="-2.54778192147277e+000"/><Con from="48" weight="4.09700432532929e-001"/><Con from="49" weight="1.50462971052058e-001"/><Con from="50" weight="2.10102078737711e-001"/><Con from="51" weight="4.98845099324947e-001"/><Con from="52" weight="1.46715401603075e+000"/><Con from="53" weight="1.28404512210873e+000"/><Con from="54" weight="3.57552613100979e-001"/><Con from="55" weight="3.14226175460441e-001"/><Con from="56" weight="-1.08007179402096e+000"/><Con from="57" weight="1.12414137980531e+000"/><Con from="58" weight="3.43246690010462e-001"/><Con from="59" weight="5.91411939383665e-001"/><Con from="60" weight="1.13001524060376e-001"/><Con from="61" weight="6.20965573427671e-001"/><Con from="62" weight="-4.68639579446723e-001"/><Con from="63" weight="-1.03251979714426e+000"/><Con from="64" weight="-1.43470176312940e+000"/><Con from="65" weight="1.11712460936439e-001"/><Con from="66" weight="-2.55662735472216e+000"/><Con from="67" weight="-1.07594326504610e-001"/><Con from="68" weight="-5.47487278105806e-001"/><Con from="69" weight="3.83853663630564e-001"/><Con from="70" weight="1.30292892513424e+000"/><Con from="71" weight="7.50621197384724e-001"/><Con from="72" weight="-2.51627426681894e-001"/><Con from="73" weight="1.36184576680749e-001"/><Con from="74" weight="6.91266249940224e-002"/><Con from="75" weight="4.97494032142349e-001"/><Con from="76" weight="2.33241019992555e-001"/><Con from="77" weight="3.92843890354421e-001"/><Con from="78" weight="-7.78629807549431e-001"/><Con from="79" weight="7.02366107529743e-001"/><Con from="80" weight="4.66675650484988e-001"/><Con from="81" weight="-2.00558089511878e-001"/><Con from="82" weight="-2.98014812507648e-001"/><Con from="83" weight="5.76826545588744e-001"/><Con from="84" weight="1.75885159682549e-001"/><Con from="85" weight="-3.56754303061501e-001"/><Con from="86" weight="-8.74958801801799e-001"/><Con from="87" weight="-1.09200852935401e+000"/><Con from="88" weight="-1.06025440353169e+000"/><Con from="89" weight="7.47624790959909e-001"/><Con from="90" weight="4.71074921666511e-001"/><Con from="91" weight="1.33698801877934e+000"/><Con from="92" weight="-1.86677337356393e+000"/><Con from="93" weight="-3.75539485374366e-001"/><Con from="94" weight="-5.57904472662212e-001"/><Con from="95" weight="-2.14590020410021e-001"/><Con from="96" weight="1.01382387086658e+000"/><Con from="97" weight="-6.33257218945926e-001"/><Con from="98" weight="3.19747830006357e-001"/><Con from="99" weight="-3.17999050232721e-001"/></Neuron><Neuron id="111" bias="1.08649006053071e+000"><Con from="0" weight="8.27039146628675e-002"/><Con from="1" weight="-4.05814387150889e-001"/><Con from="2" weight="-1.75010953072741e-001"/><Con from="3" weight="9.89758045083907e-001"/><Con from="4" weight="-1.20595900530004e+000"/><Con from="5" weight="-3.92407503479259e-001"/><Con from="6" weight="-1.10829954276809e+000"/><Con from="7" weight="-9.22758609819582e-001"/><Con from="8" weight="8.92771023404027e-001"/><Con from="9" weight="4.30903403092202e-001"/><Con from="10" weight="6.22622400433469e-001"/><Con from="11" weight="-6.07488652083441e-001"/><Con from="12" weight="4.56961815191938e-001"/><Con from="13" weight="1.00203827309561e+000"/><Con from="14" weight="-3.52899045422861e-001"/><Con from="15" weight="-7.16218454171683e-001"/><Con from="16" weight="-8.65398556410661e-001"/><Con from="17" weight="9.26057315429962e-001"/><Con from="18" weight="7.79397486808253e-002"/><Con from="19" weight="6.13638724817938e-001"/><Con from="20" weight="-2.91857005762035e-001"/><Con from="21" weight="-2.91049383096913e-001"/><Con from="22" weight="-1.37340885168908e-001"/><Con from="23" weight="-5.03200398742114e-001"/><Con from="24" weight="-1.04783608411224e+000"/><Con from="25" weight="1.10335847608264e+000"/><Con from="26" weight="1.75549539443139e-001"/><Con from="27" weight="1.20977676376502e+000"/><Con from="28" weight="-1.05316053819143e+000"/><Con from="29" weight="4.06580541802347e-001"/><Con from="30" weight="1.07892458744957e+000"/><Con from="31" weight="-5.47630051477662e-001"/><Con from="32" weight="-9.66070149882549e-001"/><Con from="33" weight="2.08910030372848e-001"/><Con from="34" weight="3.51683401111141e-001"/><Con from="35" weight="3.79788807694906e-001"/><Con from="36" weight="1.68616090426685e-001"/><Con from="37" weight="4.16783806013951e-001"/><Con from="38" weight="6.99486686673975e-002"/><Con from="39" weight="5.35517656716754e-001"/><Con from="40" weight="-2.71109490035319e-002"/><Con from="41" weight="-8.43426978824810e-003"/><Con from="42" weight="-4.70921661513653e-001"/><Con from="43" weight="9.46436428816341e-001"/><Con from="44" weight="1.15259581928580e-001"/><Con from="45" weight="3.10713007133655e-001"/><Con from="46" weight="4.53300860494376e-001"/><Con from="47" weight="9.37830774469698e-003"/><Con from="48" weight="3.68266004110811e-001"/><Con from="49" weight="-6.63945130576726e-001"/><Con from="50" weight="6.85316033241100e-001"/><Con from="51" weight="-1.27676532364560e+000"/><Con from="52" weight="7.65044232874746e-001"/><Con from="53" weight="1.99633444290964e-001"/><Con from="54" weight="1.41281912872665e-001"/><Con from="55" weight="5.04007947294284e-001"/><Con from="56" weight="-7.74196955346681e-001"/><Con from="57" weight="1.32380401566657e-001"/><Con from="58" weight="-5.50952877091634e-001"/><Con from="59" weight="4.79062457152160e-001"/><Con from="60" weight="1.04018378903368e-001"/><Con from="61" weight="-5.00682280820881e-002"/><Con from="62" weight="-1.51428599404046e+000"/><Con from="63" weight="2.00289852647737e-001"/><Con from="64" weight="-8.74843359516341e-001"/><Con from="65" weight="5.07121886351604e-001"/><Con from="66" weight="-2.09612486763410e-001"/><Con from="67" weight="-2.57855048654596e-002"/><Con from="68" weight="-6.22498195560604e-001"/><Con from="69" weight="1.80310780209957e-001"/><Con from="70" weight="7.98191419840918e-001"/><Con from="71" weight="-1.71037783440305e+000"/><Con from="72" weight="-1.30126513132988e-001"/><Con from="73" weight="2.88558945472784e-001"/><Con from="74" weight="1.13148732777509e+000"/><Con from="75" weight="1.20006818976182e+000"/><Con from="76" weight="1.63301147094086e+000"/><Con from="77" weight="4.75928840777268e-001"/><Con from="78" weight="1.66366880159963e-004"/><Con from="79" weight="-7.24785624247024e-001"/><Con from="80" weight="-8.89587611346992e-001"/><Con from="81" weight="3.52628231453026e-001"/><Con from="82" weight="-1.54718093099890e+000"/><Con from="83" weight="7.43331700807966e-002"/><Con from="84" weight="3.02573640171998e-001"/><Con from="85" weight="-6.25987041136125e-001"/><Con from="86" weight="3.86816318983888e-001"/><Con from="87" weight="-2.78273764467612e-001"/><Con from="88" weight="-4.63036274070083e-001"/><Con from="89" weight="7.03999753651026e-001"/><Con from="90" weight="6.90928523412672e-001"/><Con from="91" weight="1.42631609292007e+000"/><Con from="92" weight="1.45463236697569e+000"/><Con from="93" weight="-1.03608896720363e+000"/><Con from="94" weight="-3.93441809519940e-001"/><Con from="95" weight="-3.17385936455163e-001"/><Con from="96" weight="-9.84230413164771e-001"/><Con from="97" weight="-2.07212044954774e+000"/><Con from="98" weight="4.04815623778144e-001"/><Con from="99" weight="-2.12630589047393e-001"/></Neuron><Neuron id="112" bias="3.33817197478297e-001"><Con from="0" weight="2.65061506489551e-001"/><Con from="1" weight="-2.83494128730646e-001"/><Con from="2" weight="1.77490546260353e-001"/><Con from="3" weight="-9.00824103963179e-002"/><Con from="4" weight="2.14769430742405e-001"/><Con from="5" weight="-1.06326988310025e+000"/><Con from="6" weight="5.53769009009880e-001"/><Con from="7" weight="-5.41408451707828e-002"/><Con from="8" weight="-1.74409631122118e-001"/><Con from="9" weight="3.12401269236721e-001"/><Con from="10" weight="-2.68286413789389e-002"/><Con from="11" weight="-2.92958017922642e-001"/><Con from="12" weight="2.75924588712839e-001"/><Con from="13" weight="-1.37293578460134e+000"/><Con from="14" weight="-1.38603626405173e+000"/><Con from="15" weight="-2.14307095611536e-001"/><Con from="16" weight="2.03915228478155e+000"/><Con from="17" weight="-3.50310983390882e-001"/><Con from="18" weight="2.59054408803817e-001"/><Con from="19" weight="-7.83680951481106e-001"/><Con from="20" weight="-8.58073132492273e-001"/><Con from="21" weight="-7.36424486189759e-002"/><Con from="22" weight="-4.61543515692793e-001"/><Con from="23" weight="-1.06159746744345e+000"/><Con from="24" weight="6.63651112616816e-001"/><Con from="25" weight="-2.85123401206937e-001"/><Con from="26" weight="4.77850206294888e-001"/><Con from="27" weight="3.38379595696834e-001"/><Con from="28" weight="3.64878830726162e-001"/><Con from="29" weight="-1.17817576918641e-001"/><Con from="30" weight="-8.35437108303939e-001"/><Con from="31" weight="-4.18949902919965e-002"/><Con from="32" weight="-4.03699293983565e-001"/><Con from="33" weight="-8.33911372057524e-001"/><Con from="34" weight="1.05685361115810e-001"/><Con from="35" weight="1.28829755061295e-001"/><Con from="36" weight="-5.33593867620757e-001"/><Con from="37" weight="1.79764029192863e-002"/><Con from="38" weight="3.62006661034120e-001"/><Con from="39" weight="7.61683363246448e-001"/><Con from="40" weight="1.69602318669072e-003"/><Con from="41" weight="3.88930888800970e-001"/><Con from="42" weight="5.65991156926726e-001"/><Con from="43" weight="1.76696575723590e+000"/><Con from="44" weight="1.49634453118792e-001"/><Con from="45" weight="1.76332707918535e+000"/><Con from="46" weight="2.36073870856538e-001"/><Con from="47" weight="4.54015186850724e-001"/><Con from="48" weight="8.06971579852586e-001"/><Con from="49" weight="-8.41918104073294e-001"/><Con from="50" weight="2.66250246810484e-001"/><Con from="51" weight="3.26649759520475e-001"/><Con from="52" weight="-1.10473247470101e+000"/><Con from="53" weight="-2.22759511869566e-002"/><Con from="54" weight="4.62056858509774e-001"/><Con from="55" weight="8.16638690346843e-001"/><Con from="56" weight="2.46298067921570e-001"/><Con from="57" weight="-4.58369480237669e-003"/><Con from="58" weight="4.33689074954155e-001"/><Con from="59" weight="-5.72458091747480e-001"/><Con from="60" weight="1.09935052813503e-001"/><Con from="61" weight="1.42294799390478e+000"/><Con from="62" weight="-1.18179805065268e+000"/><Con from="63" weight="3.35177924388163e-001"/><Con from="64" weight="-7.26878129130992e-001"/><Con from="65" weight="2.24411097158317e-001"/><Con from="66" weight="2.79796613341140e-001"/><Con from="67" weight="8.04305844073930e-001"/><Con from="68" weight="-7.62091958272988e-001"/><Con from="69" weight="2.74689097068992e-001"/><Con from="70" weight="-7.89578846015222e-002"/><Con from="71" weight="-8.20880129600821e-001"/><Con from="72" weight="-2.32842046322389e-001"/><Con from="73" weight="-3.30691429914911e-001"/><Con from="74" weight="-2.06705547448625e-001"/><Con from="75" weight="-3.26301876531711e-001"/><Con from="76" weight="-4.30266095855733e-001"/><Con from="77" weight="-4.01593624545118e-001"/><Con from="78" weight="4.20203106282855e-001"/><Con from="79" weight="-3.57191659435489e-001"/><Con from="80" weight="-3.66790643305588e-001"/><Con from="81" weight="4.01321199939419e-001"/><Con from="82" weight="-1.90435051855097e-001"/><Con from="83" weight="-1.13797084724133e-001"/><Con from="84" weight="3.93132184935859e-001"/><Con from="85" weight="-3.55993509755307e-001"/><Con from="86" weight="-1.41031811851295e+000"/><Con from="87" weight="-4.24558073182495e-001"/><Con from="88" weight="-7.26124460565800e-001"/><Con from="89" weight="-4.51381708750071e-001"/><Con from="90" weight="4.92840649495246e-001"/><Con from="91" weight="2.91422558854363e-001"/><Con from="92" weight="-1.08321067775162e-001"/><Con from="93" weight="7.68431001846838e-001"/><Con from="94" weight="-5.90600506165635e-002"/><Con from="95" weight="8.44821808793288e-002"/><Con from="96" weight="2.54718976413403e-001"/><Con from="97" weight="7.56754023676564e-002"/><Con from="98" weight="-1.58295164280097e+000"/><Con from="99" weight="2.90082420882708e-001"/></Neuron><Neuron id="113" bias="-1.60647302741902e+000"><Con from="0" weight="1.59351073351033e-001"/><Con from="1" weight="-6.35923589237643e-003"/><Con from="2" weight="1.09760518303154e-001"/><Con from="3" weight="-1.07137544624258e+000"/><Con from="4" weight="5.09698023787362e-001"/><Con from="5" weight="1.42709283200249e+000"/><Con from="6" weight="9.42931597748971e-001"/><Con from="7" weight="7.42554295808259e-001"/><Con from="8" weight="-1.06675806044929e+000"/><Con from="9" weight="-8.60487603287631e-001"/><Con from="10" weight="-2.78068799626350e-001"/><Con from="11" weight="-6.49372547303157e-001"/><Con from="12" weight="-1.62044464871735e-001"/><Con from="13" weight="5.15051796984003e-001"/><Con from="14" weight="2.17452132203704e-001"/><Con from="15" weight="-1.82661482848046e-002"/><Con from="16" weight="-8.02432536272739e-001"/><Con from="17" weight="1.48765888503649e+000"/><Con from="18" weight="2.86810430681786e-001"/><Con from="19" weight="-8.47967018456547e-003"/><Con from="20" weight="-4.74860821495777e-001"/><Con from="21" weight="5.85061052734702e-001"/><Con from="22" weight="-8.01992750483101e-001"/><Con from="23" weight="3.04173252297690e-001"/><Con from="24" weight="-9.00245340714146e-001"/><Con from="25" weight="3.46601427164128e-001"/><Con from="26" weight="-6.42236067345820e-001"/><Con from="27" weight="-1.04158669833917e-001"/><Con from="28" weight="1.00133755024527e+000"/><Con from="29" weight="-1.21564214798394e+000"/><Con from="30" weight="-2.15845327930538e-001"/><Con from="31" weight="1.63280644767764e-001"/><Con from="32" weight="9.79603443523383e-001"/><Con from="33" weight="-1.03105650288222e-001"/><Con from="34" weight="-1.07966219290034e+000"/><Con from="35" weight="-4.44274015639178e-001"/><Con from="36" weight="2.72704183932897e-001"/><Con from="37" weight="5.35222722830557e-001"/><Con from="38" weight="6.19855781083009e-002"/><Con from="39" weight="9.52011594422237e-002"/><Con from="40" weight="1.05457511689361e+000"/><Con from="41" weight="8.25571674222918e-002"/><Con from="42" weight="5.21438946143317e-001"/><Con from="43" weight="5.77094108545325e-001"/><Con from="44" weight="8.78961280926023e-001"/><Con from="45" weight="3.55649359182746e-001"/><Con from="46" weight="3.11379783760911e-001"/><Con from="47" weight="-3.54257712595992e-001"/><Con from="48" weight="-2.00084478741386e-001"/><Con from="49" weight="6.36174473379346e-002"/><Con from="50" weight="-9.15795302632051e-001"/><Con from="51" weight="-1.19293015184753e-001"/><Con from="52" weight="-7.15481719409862e-001"/><Con from="53" weight="2.61874495455252e-001"/><Con from="54" weight="-1.88215389457562e-001"/><Con from="55" weight="4.72603651991302e-001"/><Con from="56" weight="1.01979688340906e+000"/><Con from="57" weight="-5.23123250925749e-001"/><Con from="58" weight="3.83229011390613e-001"/><Con from="59" weight="2.83917997175051e-001"/><Con from="60" weight="6.27905590326132e-002"/><Con from="61" weight="-2.74766565129518e-001"/><Con from="62" weight="-1.55351355876504e+000"/><Con from="63" weight="4.11342214693210e-001"/><Con from="64" weight="-7.20090413349467e-001"/><Con from="65" weight="2.16889946152622e-001"/><Con from="66" weight="3.48582635811973e-001"/><Con from="67" weight="1.26523565731989e-001"/><Con from="68" weight="6.38552179776824e-001"/><Con from="69" weight="-8.04257317946832e-001"/><Con from="70" weight="3.02675296623443e-002"/><Con from="71" weight="8.85561662130216e-001"/><Con from="72" weight="6.52792512503839e-001"/><Con from="73" weight="-4.78487120497515e-001"/><Con from="74" weight="-1.71347593678502e+000"/><Con from="75" weight="-1.28359863695872e+000"/><Con from="76" weight="7.30811056378175e-002"/><Con from="77" weight="3.30203298156147e-001"/><Con from="78" weight="1.00258214781465e+000"/><Con from="79" weight="6.25703885903087e-001"/><Con from="80" weight="1.01751232141808e+000"/><Con from="81" weight="-3.87260898217163e-001"/><Con from="82" weight="7.35296592892443e-002"/><Con from="83" weight="-1.05828607037106e+000"/><Con from="84" weight="1.54206290317813e+000"/><Con from="85" weight="4.45444804586084e-001"/><Con from="86" weight="-1.25898255747967e+000"/><Con from="87" weight="-5.83759379560868e-002"/><Con from="88" weight="-8.62663886134914e-001"/><Con from="89" weight="6.50669079807494e-001"/><Con from="90" weight="4.00184076766356e-001"/><Con from="91" weight="5.03318889239606e-002"/><Con from="92" weight="3.80872625105141e-001"/><Con from="93" weight="-3.63391694572776e-001"/><Con from="94" weight="-4.60929479740022e-001"/><Con from="95" weight="-1.23198909318894e+000"/><Con from="96" weight="2.31191895386309e-001"/><Con from="97" weight="-9.45695542962320e-001"/><Con from="98" weight="-1.29727917599532e+000"/><Con from="99" weight="2.24353451322430e-001"/></Neuron><Neuron id="114" bias="3.67758436954758e-001"><Con from="0" weight="2.46129435735257e-001"/><Con from="1" weight="-6.24759053373281e-001"/><Con from="2" weight="-7.37185272170408e-001"/><Con from="3" weight="-1.55216029706790e+000"/><Con from="4" weight="-1.48675182537160e-001"/><Con from="5" weight="-5.74149969957421e-001"/><Con from="6" weight="-4.44551670273232e-002"/><Con from="7" weight="4.06217283876517e-001"/><Con from="8" weight="-1.77984081369220e-001"/><Con from="9" weight="-6.37113028579813e-001"/><Con from="10" weight="-4.97268764985691e-001"/><Con from="11" weight="5.27137945986811e-001"/><Con from="12" weight="4.12306746360308e-001"/><Con from="13" weight="4.58953292658447e-001"/><Con from="14" weight="-1.48720172331235e+000"/><Con from="15" weight="6.73244274456647e-001"/><Con from="16" weight="-7.95931945249581e-001"/><Con from="17" weight="8.36485912444263e-001"/><Con from="18" weight="4.49824765214487e-002"/><Con from="19" weight="-1.94460376304547e+000"/><Con from="20" weight="-5.69735142745779e-001"/><Con from="21" weight="2.90726063913944e-001"/><Con from="22" weight="-7.17393688173188e-001"/><Con from="23" weight="-4.70679557067077e-002"/><Con from="24" weight="1.92879174058743e-001"/><Con from="25" weight="-6.25627410579016e-001"/><Con from="26" weight="4.13814881008127e-001"/><Con from="27" weight="-3.14103305369640e-001"/><Con from="28" weight="-4.57000771923005e-001"/><Con from="29" weight="-2.25029591039605e-001"/><Con from="30" weight="-3.23176052701738e-001"/><Con from="31" weight="7.19461621521467e-001"/><Con from="32" weight="-4.59848003921723e-001"/><Con from="33" weight="-2.62184779622617e-001"/><Con from="34" weight="3.09101837951474e-001"/><Con from="35" weight="-3.37058629049050e-001"/><Con from="36" weight="-5.14466404573974e-001"/><Con from="37" weight="2.39456027241827e-001"/><Con from="38" weight="-2.16029222123882e-001"/><Con from="39" weight="-9.84799072059916e-001"/><Con from="40" weight="-9.99522720843687e-001"/><Con from="41" weight="4.54660504075332e-001"/><Con from="42" weight="3.12529576835383e-001"/><Con from="43" weight="4.75033334687311e-001"/><Con from="44" weight="4.43903842025492e-001"/><Con from="45" weight="1.61183705410316e+000"/><Con from="46" weight="-4.28653036414160e-001"/><Con from="47" weight="1.69366638462311e+000"/><Con from="48" weight="1.07083245227403e+000"/><Con from="49" weight="7.81160931166353e-001"/><Con from="50" weight="-3.60676498969016e-001"/><Con from="51" weight="9.09785631811984e-001"/><Con from="52" weight="2.99654259458529e-002"/><Con from="53" weight="-7.65711122400668e-001"/><Con from="54" weight="-1.50407552010335e-001"/><Con from="55" weight="-1.24953552883272e+000"/><Con from="56" weight="4.64182709363356e-001"/><Con from="57" weight="2.31728340425014e-001"/><Con from="58" weight="-3.35582690626023e-002"/><Con from="59" weight="-1.47104071564823e-001"/><Con from="60" weight="2.73305060803720e-001"/><Con from="61" weight="2.26807750824807e+000"/><Con from="62" weight="-9.12381868524936e-002"/><Con from="63" weight="2.25319572300339e-001"/><Con from="64" weight="-7.74206006551753e-001"/><Con from="65" weight="-4.32478905335901e-001"/><Con from="66" weight="1.19567297502889e+000"/><Con from="67" weight="1.09444939969228e+000"/><Con from="68" weight="8.68347044275467e-001"/><Con from="69" weight="4.23538502498414e-002"/><Con from="70" weight="-4.19281576241952e-001"/><Con from="71" weight="-3.77950874676989e-001"/><Con from="72" weight="-7.85841096861544e-002"/><Con from="73" weight="-5.91683151783158e-001"/><Con from="74" weight="-3.32596592066520e-001"/><Con from="75" weight="-1.12041255452191e+000"/><Con from="76" weight="-5.63123701425695e-001"/><Con from="77" weight="-3.25263538959949e-001"/><Con from="78" weight="6.12110677613024e-001"/><Con from="79" weight="1.60894250342479e-001"/><Con from="80" weight="1.29524339589041e-001"/><Con from="81" weight="8.17330659231963e-001"/><Con from="82" weight="-1.17365339303673e-001"/><Con from="83" weight="-4.80249274618297e-001"/><Con from="84" weight="1.06173256962065e+000"/><Con from="85" weight="-4.54485228656583e-001"/><Con from="86" weight="-5.96894461879445e-001"/><Con from="87" weight="-5.18128373315215e-001"/><Con from="88" weight="1.03489610381217e-001"/><Con from="89" weight="-1.42461870350224e+000"/><Con from="90" weight="-8.32483892046582e-001"/><Con from="91" weight="-4.80791600546947e-001"/><Con from="92" weight="-1.64791027742590e-001"/><Con from="93" weight="1.28679320705635e-001"/><Con from="94" weight="-6.47849736548444e-001"/><Con from="95" weight="3.59831034461993e-001"/><Con from="96" weight="-1.82388804884027e-002"/><Con from="97" weight="5.82203603118722e-001"/><Con from="98" weight="-2.36652251997043e+000"/><Con from="99" weight="3.39034728794208e-001"/></Neuron><Neuron id="115" bias="1.69534392277823e-001"><Con from="0" weight="-2.24195716489902e-001"/><Con from="1" weight="-1.45110600949798e+000"/><Con from="2" weight="-3.31870376074812e-001"/><Con from="3" weight="-2.95694178013890e-001"/><Con from="4" weight="1.90411951943358e+000"/><Con from="5" weight="-1.56554215166636e+000"/><Con from="6" weight="1.63581834907428e-001"/><Con from="7" weight="2.29829124211173e-001"/><Con from="8" weight="-1.11600571472686e+000"/><Con from="9" weight="5.16223870357512e-001"/><Con from="10" weight="1.22017939285336e+000"/><Con from="11" weight="-1.60945581802229e-001"/><Con from="12" weight="-1.61471881263323e+000"/><Con from="13" weight="-2.01356210274206e+000"/><Con from="14" weight="-1.20295023370938e-001"/><Con from="15" weight="9.96825257795712e-001"/><Con from="16" weight="2.10572037265229e+000"/><Con from="17" weight="-1.40668097664415e+000"/><Con from="18" weight="3.42428998436485e-001"/><Con from="19" weight="1.00929673673098e+000"/><Con from="20" weight="2.33324103384802e-001"/><Con from="21" weight="-1.02173605363646e+000"/><Con from="22" weight="1.06686273229314e+000"/><Con from="23" weight="-1.06809495207108e+000"/><Con from="24" weight="-5.67680052554435e-001"/><Con from="25" weight="3.68354765979107e-002"/><Con from="26" weight="1.25733455372911e+000"/><Con from="27" weight="2.09686775353521e-001"/><Con from="28" weight="-4.00930256547700e-001"/><Con from="29" weight="-5.15920692278520e-002"/><Con from="30" weight="1.51071293086518e+000"/><Con from="31" weight="-3.48110837197188e-001"/><Con from="32" weight="-1.74985673674790e+000"/><Con from="33" weight="-1.38224623013505e+000"/><Con from="34" weight="-2.37253387208667e-001"/><Con from="35" weight="-2.48309024139525e-001"/><Con from="36" weight="-6.18998639586489e-001"/><Con from="37" weight="-2.07456834413555e-002"/><Con from="38" weight="-1.88180535019035e-001"/><Con from="39" weight="8.95435516709748e-001"/><Con from="40" weight="9.72317792730094e-001"/><Con from="41" weight="1.38261919383314e+000"/><Con from="42" weight="-1.08464379610637e+000"/><Con from="43" weight="-1.95873689906021e+000"/><Con from="44" weight="-7.15131009766811e-001"/><Con from="45" weight="-5.71135491934247e-001"/><Con from="46" weight="9.79367541042142e-002"/><Con from="47" weight="3.28656999657694e-001"/><Con from="48" weight="1.98535161615611e-001"/><Con from="49" weight="1.01063436917928e+000"/><Con from="50" weight="-2.94629807800105e-001"/><Con from="51" weight="-7.37680804887439e-001"/><Con from="52" weight="-1.33678591674954e+000"/><Con from="53" weight="6.86565187722259e-001"/><Con from="54" weight="-2.12465603879676e-001"/><Con from="55" weight="9.08682106752623e-001"/><Con from="56" weight="1.31398253821376e+000"/><Con from="57" weight="6.20980485422936e-001"/><Con from="58" weight="-1.10037271831032e+000"/><Con from="59" weight="-6.32696415772565e-001"/><Con from="60" weight="-7.30010348721776e-001"/><Con from="61" weight="-2.27588917002237e+000"/><Con from="62" weight="-1.79475432310671e+000"/><Con from="63" weight="-1.04701624716305e+000"/><Con from="64" weight="-8.90298533410775e-001"/><Con from="65" weight="6.82088297593729e-002"/><Con from="66" weight="3.53798816865969e-001"/><Con from="67" weight="1.66408323939017e-001"/><Con from="68" weight="1.18472210059447e+000"/><Con from="69" weight="-3.21742506305955e-001"/><Con from="70" weight="8.69201302697300e-002"/><Con from="71" weight="6.37046826885573e-001"/><Con from="72" weight="-2.72323707869575e-001"/><Con from="73" weight="4.46442007933004e-002"/><Con from="74" weight="-3.79101234933146e-001"/><Con from="75" weight="-4.42204263123008e-001"/><Con from="76" weight="-5.04772917646124e-001"/><Con from="77" weight="-2.38159319616188e-001"/><Con from="78" weight="9.07377012174935e-001"/><Con from="79" weight="3.55036870234642e-001"/><Con from="80" weight="5.58820100710276e-001"/><Con from="81" weight="-6.26299930453214e-001"/><Con from="82" weight="1.07918007107395e-001"/><Con from="83" weight="6.46661136174207e-001"/><Con from="84" weight="-4.97053177777839e-001"/><Con from="85" weight="-1.33172602092313e+000"/><Con from="86" weight="-5.64112375319234e-001"/><Con from="87" weight="-3.57992233552372e-001"/><Con from="88" weight="6.38696479794041e-002"/><Con from="89" weight="7.02680719927350e-001"/><Con from="90" weight="-7.35716250707038e-001"/><Con from="91" weight="-6.12401393922398e-001"/><Con from="92" weight="-9.73792300469360e-001"/><Con from="93" weight="-2.03673020152490e-001"/><Con from="94" weight="6.40347852276890e-001"/><Con from="95" weight="-4.80698939638815e-001"/><Con from="96" weight="-3.25840760190380e-001"/><Con from="97" weight="-4.12561589424240e-001"/><Con from="98" weight="1.59089480165058e-001"/><Con from="99" weight="-3.22547643131533e-001"/></Neuron><Neuron id="116" bias="-1.00227329360565e+000"><Con from="0" weight="-5.99102599121099e-001"/><Con from="1" weight="4.12360635716477e-001"/><Con from="2" weight="-4.33643834401800e-001"/><Con from="3" weight="-8.13932039306266e-002"/><Con from="4" weight="5.97223480828637e-001"/><Con from="5" weight="-2.48004043688242e+000"/><Con from="6" weight="5.40089285873248e+000"/><Con from="7" weight="2.00392197862036e-001"/><Con from="8" weight="3.84236719733323e+000"/><Con from="9" weight="-2.63665130699808e+000"/><Con from="10" weight="-1.96426273537369e+000"/><Con from="11" weight="-1.34273356877583e+000"/><Con from="12" weight="-9.23590018601671e-001"/><Con from="13" weight="-4.26577125090165e+000"/><Con from="14" weight="1.84075893107274e+000"/><Con from="15" weight="-1.69116693184790e+000"/><Con from="16" weight="-1.54961426097313e-001"/><Con from="17" weight="2.51989215115216e+000"/><Con from="18" weight="-6.06663321985063e-002"/><Con from="19" weight="-5.63473609696116e+000"/><Con from="20" weight="8.80768478998916e-001"/><Con from="21" weight="-1.63934875491430e-001"/><Con from="22" weight="-4.93211909386281e+000"/><Con from="23" weight="-2.95208853159499e-002"/><Con from="24" weight="3.89288371228647e-001"/><Con from="25" weight="-3.03448309635977e+000"/><Con from="26" weight="1.41557951566808e+000"/><Con from="27" weight="-2.57720244175008e-001"/><Con from="28" weight="-2.29846633741985e-001"/><Con from="29" weight="2.50335075443034e-002"/><Con from="30" weight="7.72818017107860e-001"/><Con from="31" weight="3.39686254857772e+000"/><Con from="32" weight="-1.39941816458164e+000"/><Con from="33" weight="1.38865591311563e+000"/><Con from="34" weight="-1.89076911850009e+000"/><Con from="35" weight="3.32676621908236e-001"/><Con from="36" weight="1.17393506668947e+000"/><Con from="37" weight="2.05745689943132e+000"/><Con from="38" weight="2.19152629841555e-001"/><Con from="39" weight="3.13448582760845e-001"/><Con from="40" weight="5.74994635313715e-001"/><Con from="41" weight="1.78895307207036e+000"/><Con from="42" weight="1.01567263452798e+000"/><Con from="43" weight="2.37367361759522e-001"/><Con from="44" weight="-3.23971911289932e-001"/><Con from="45" weight="-1.65679351867682e+000"/><Con from="46" weight="-2.86403625892625e-001"/><Con from="47" weight="-1.06702592958485e+000"/><Con from="48" weight="1.10302101672243e+000"/><Con from="49" weight="-3.69197809252201e-001"/><Con from="50" weight="-4.24179953551794e-001"/><Con from="51" weight="1.44068334238198e+000"/><Con from="52" weight="1.76811790217876e+000"/><Con from="53" weight="3.54768061455853e-002"/><Con from="54" weight="5.63473499884012e-001"/><Con from="55" weight="-4.47107490165843e-001"/><Con from="56" weight="-8.08905053340862e-002"/><Con from="57" weight="3.16527283398940e-001"/><Con from="58" weight="1.29347402423423e-001"/><Con from="59" weight="4.13484035534984e-001"/><Con from="60" weight="2.62304727178955e-003"/><Con from="61" weight="-1.76365684201258e+000"/><Con from="62" weight="2.59739580396395e+000"/><Con from="63" weight="1.48543739557020e+000"/><Con from="64" weight="-2.74623359341342e+000"/><Con from="65" weight="-1.45048815774627e-001"/><Con from="66" weight="-8.21621965127665e-001"/><Con from="67" weight="-5.03605838010237e-001"/><Con from="68" weight="5.15252341832121e-001"/><Con from="69" weight="2.41745604189311e+000"/><Con from="70" weight="-3.79461221298586e+000"/><Con from="71" weight="3.27603175929856e+000"/><Con from="72" weight="-3.35667015717409e+000"/><Con from="73" weight="1.45623497492732e+000"/><Con from="74" weight="7.89608193715238e-001"/><Con from="75" weight="-3.61382250014396e-001"/><Con from="76" weight="1.03840358352108e+000"/><Con from="77" weight="-9.87028656298234e-001"/><Con from="78" weight="6.79891693713923e-001"/><Con from="79" weight="2.24839098476391e-001"/><Con from="80" weight="-1.02628464321448e+000"/><Con from="81" weight="3.64443609324652e+000"/><Con from="82" weight="-4.94010711495356e+000"/><Con from="83" weight="3.50113594136746e+000"/><Con from="84" weight="2.16578542079045e-001"/><Con from="85" weight="1.55548206038408e+000"/><Con from="86" weight="-8.03356665048662e-001"/><Con from="87" weight="1.39255600123893e+000"/><Con from="88" weight="7.19615623107937e-001"/><Con from="89" weight="4.66773757799268e-001"/><Con from="90" weight="5.01692465357222e-001"/><Con from="91" weight="-2.44096031544572e+000"/><Con from="92" weight="1.88699523300223e+000"/><Con from="93" weight="-3.52215960971289e+000"/><Con from="94" weight="4.60230696416244e-001"/><Con from="95" weight="-2.55762862352944e-001"/><Con from="96" weight="-8.99342690889586e-001"/><Con from="97" weight="-3.96323063314023e+000"/><Con from="98" weight="-4.56755944549273e+000"/><Con from="99" weight="-5.81000841591689e-001"/></Neuron><Neuron id="117" bias="-6.87151197283290e-001"><Con from="0" weight="4.14904812437058e-001"/><Con from="1" weight="-2.23963867434900e-001"/><Con from="2" weight="-1.08842633985613e+000"/><Con from="3" weight="3.01197168434728e-001"/><Con from="4" weight="-1.22096634712692e+000"/><Con from="5" weight="1.66107247274005e+000"/><Con from="6" weight="-2.84139297465956e+000"/><Con from="7" weight="-4.71276075156651e-001"/><Con from="8" weight="-5.41988144399425e-001"/><Con from="9" weight="1.97304516590661e+000"/><Con from="10" weight="-1.26015404789053e+000"/><Con from="11" weight="-2.65773816632445e-001"/><Con from="12" weight="7.90737556727714e-002"/><Con from="13" weight="2.83458468538171e+000"/><Con from="14" weight="-3.90559576132539e-001"/><Con from="15" weight="1.49304395251792e+000"/><Con from="16" weight="-9.23723823465293e-001"/><Con from="17" weight="-5.40778090573724e-001"/><Con from="18" weight="1.34392780399337e+000"/><Con from="19" weight="2.27255286848452e+000"/><Con from="20" weight="-2.77200000756270e-001"/><Con from="21" weight="-1.00994104896084e-001"/><Con from="22" weight="1.82059316668197e+000"/><Con from="23" weight="-2.65901213326976e+000"/><Con from="24" weight="-4.22728206942353e-001"/><Con from="25" weight="5.55345934935120e-002"/><Con from="26" weight="-3.43753725528679e+000"/><Con from="27" weight="-2.55707681615546e+000"/><Con from="28" weight="9.25212080412223e-001"/><Con from="29" weight="-9.12248087680432e-001"/><Con from="30" weight="-6.23225799946993e-001"/><Con from="31" weight="-1.72439467418494e+000"/><Con from="32" weight="-3.57155192556099e-001"/><Con from="33" weight="-1.06801358316419e+000"/><Con from="34" weight="-5.03692392516747e-001"/><Con from="35" weight="3.77544071974682e-001"/><Con from="36" weight="-3.53620241729470e-001"/><Con from="37" weight="1.93886237714136e-002"/><Con from="38" weight="-1.38888201406088e+000"/><Con from="39" weight="4.79512682385008e-001"/><Con from="40" weight="3.31707848904736e-001"/><Con from="41" weight="-1.29853524150078e+000"/><Con from="42" weight="-2.01213016077969e+000"/><Con from="43" weight="-2.64191496695202e-003"/><Con from="44" weight="1.65193962956134e+000"/><Con from="45" weight="-1.72990898700708e-001"/><Con from="46" weight="3.82767909101476e-001"/><Con from="47" weight="-3.51734276168424e+000"/><Con from="48" weight="3.22426314721563e-001"/><Con from="49" weight="-4.90293402447152e-002"/><Con from="50" weight="5.27561490830389e-001"/><Con from="51" weight="-8.55906315514225e-001"/><Con from="52" weight="2.41584369756634e+000"/><Con from="53" weight="-3.65047908412524e-001"/><Con from="54" weight="-1.42311750697531e+000"/><Con from="55" weight="1.37119473490947e+000"/><Con from="56" weight="1.20162761531452e+000"/><Con from="57" weight="-6.69475737626572e-001"/><Con from="58" weight="-4.75245353674565e-001"/><Con from="59" weight="1.57419074718451e-001"/><Con from="60" weight="1.71152198713661e+000"/><Con from="61" weight="1.81143036978350e+000"/><Con from="62" weight="-2.10426381365173e+000"/><Con from="63" weight="-1.35067653057831e+000"/><Con from="64" weight="2.20219812454151e+000"/><Con from="65" weight="3.34090385790886e-001"/><Con from="66" weight="-3.34320994335296e+000"/><Con from="67" weight="3.90455875757937e-001"/><Con from="68" weight="1.07497352519166e-001"/><Con from="69" weight="-3.27351865688465e-001"/><Con from="70" weight="8.26851011131738e-001"/><Con from="71" weight="7.61453990734480e-001"/><Con from="72" weight="8.59887014932490e-001"/><Con from="73" weight="-8.70876985440309e-001"/><Con from="74" weight="-1.19334220983073e+000"/><Con from="75" weight="-7.69051706276814e-001"/><Con from="76" weight="-2.14182747819179e+000"/><Con from="77" weight="-1.03528286526029e+000"/><Con from="78" weight="-1.26371955256540e-002"/><Con from="79" weight="-7.33942567272765e-001"/><Con from="80" weight="-3.87936950076679e-001"/><Con from="81" weight="-2.01577017165969e+000"/><Con from="82" weight="3.00854005520766e+000"/><Con from="83" weight="-1.46712080550149e-001"/><Con from="84" weight="1.31513574371466e+000"/><Con from="85" weight="-1.92324506165020e+000"/><Con from="86" weight="1.75652946726247e+000"/><Con from="87" weight="-9.71908233936449e-001"/><Con from="88" weight="-9.05500667469803e-001"/><Con from="89" weight="6.27244322953121e-001"/><Con from="90" weight="7.31002573150788e-001"/><Con from="91" weight="1.99331831871143e+000"/><Con from="92" weight="2.53154603916184e-001"/><Con from="93" weight="1.86587411169108e+000"/><Con from="94" weight="2.47985638399687e-001"/><Con from="95" weight="-8.22437127522139e-001"/><Con from="96" weight="1.23223435126400e+000"/><Con from="97" weight="3.50450240994781e-001"/><Con from="98" weight="1.32509123251715e+000"/><Con from="99" weight="5.71829014768051e-004"/></Neuron><Neuron id="118" bias="-1.41833287835409e+000"><Con from="0" weight="1.70342734792926e+000"/><Con from="1" weight="5.47769721435258e-001"/><Con from="2" weight="-8.73684705275206e-001"/><Con from="3" weight="-3.09394574411709e-001"/><Con from="4" weight="8.23167397468183e-002"/><Con from="5" weight="-1.39532146529523e+000"/><Con from="6" weight="-1.51053137187418e+000"/><Con from="7" weight="-1.04809667675199e+000"/><Con from="8" weight="-2.14681663729969e-001"/><Con from="9" weight="1.32383013368486e-001"/><Con from="10" weight="-7.86196602510735e-001"/><Con from="11" weight="-7.17195437855648e-001"/><Con from="12" weight="1.59197214874728e-001"/><Con from="13" weight="-6.14304880328844e-002"/><Con from="14" weight="3.77816063917861e-001"/><Con from="15" weight="5.02354376788765e-001"/><Con from="16" weight="-1.20392100408493e+000"/><Con from="17" weight="4.71803188436140e-001"/><Con from="18" weight="-6.97071857239629e-002"/><Con from="19" weight="-5.37911130345011e-001"/><Con from="20" weight="6.08362734372195e-001"/><Con from="21" weight="-1.27228449415450e+000"/><Con from="22" weight="-1.54030446712986e+000"/><Con from="23" weight="1.13036516484392e+000"/><Con from="24" weight="7.75348275969143e-001"/><Con from="25" weight="-4.01983775313523e-001"/><Con from="26" weight="8.55953115986847e-002"/><Con from="27" weight="-6.78951622622419e-001"/><Con from="28" weight="2.04140342483128e-002"/><Con from="29" weight="-1.72909949093543e-001"/><Con from="30" weight="7.14521783251474e-001"/><Con from="31" weight="9.30533639602304e-002"/><Con from="32" weight="-9.80165047787625e-001"/><Con from="33" weight="-4.53494836133726e-001"/><Con from="34" weight="1.34819718425836e-001"/><Con from="35" weight="7.27859095473796e-001"/><Con from="36" weight="8.01904918625291e-001"/><Con from="37" weight="-3.91376338806677e-001"/><Con from="38" weight="1.46542108293489e-001"/><Con from="39" weight="4.31755836529030e-001"/><Con from="40" weight="-4.93260754591857e-002"/><Con from="41" weight="4.44251839196108e-001"/><Con from="42" weight="5.05584210835981e-001"/><Con from="43" weight="9.59824237842468e-001"/><Con from="44" weight="1.29224439211463e+000"/><Con from="45" weight="-2.51023017979426e-001"/><Con from="46" weight="2.59109099370649e-001"/><Con from="47" weight="-4.41255327107809e-001"/><Con from="48" weight="-5.15795473567987e-001"/><Con from="49" weight="8.61975371890670e-002"/><Con from="50" weight="3.52127705406557e-001"/><Con from="51" weight="-2.03992515361149e+000"/><Con from="52" weight="5.27028549317646e-001"/><Con from="53" weight="-5.69792003158126e-001"/><Con from="54" weight="1.90407286786198e-001"/><Con from="55" weight="5.16840283997683e-001"/><Con from="56" weight="2.35109704136282e-001"/><Con from="57" weight="4.01284950972984e-001"/><Con from="58" weight="4.42160081804744e-001"/><Con from="59" weight="4.49497633246614e-001"/><Con from="60" weight="1.05766532695871e+000"/><Con from="61" weight="-1.23063392265841e+000"/><Con from="62" weight="1.05342259336774e+000"/><Con from="63" weight="-3.75517325806983e-001"/><Con from="64" weight="4.37862911555408e-001"/><Con from="65" weight="1.89113737305088e-001"/><Con from="66" weight="-1.89265246337672e-001"/><Con from="67" weight="-2.73888098903502e-001"/><Con from="68" weight="1.19418390839981e-001"/><Con from="69" weight="-7.89481106400554e-001"/><Con from="70" weight="-6.36856662076753e-001"/><Con from="71" weight="-1.02321424904080e+000"/><Con from="72" weight="-3.23877521066779e-001"/><Con from="73" weight="-1.77393904046980e-001"/><Con from="74" weight="-2.79856035074532e-001"/><Con from="75" weight="-1.47153332416690e-001"/><Con from="76" weight="1.41476763168602e-001"/><Con from="77" weight="-1.94196191075050e-001"/><Con from="78" weight="-4.83817137755141e-002"/><Con from="79" weight="-4.55595458608200e-001"/><Con from="80" weight="-2.19421483836371e-001"/><Con from="81" weight="1.41547209888783e-001"/><Con from="82" weight="-3.25415061922349e-001"/><Con from="83" weight="-6.46257820186798e-001"/><Con from="84" weight="-4.36511828792279e-001"/><Con from="85" weight="7.23454480545321e-002"/><Con from="86" weight="4.71569500310241e-001"/><Con from="87" weight="2.67705145897647e-001"/><Con from="88" weight="2.36463234939506e-001"/><Con from="89" weight="4.09798787699460e-001"/><Con from="90" weight="-7.86576907297973e-001"/><Con from="91" weight="-2.81210683685142e+000"/><Con from="92" weight="8.47298799185609e-001"/><Con from="93" weight="1.31356197700226e-002"/><Con from="94" weight="-3.57044790955140e-001"/><Con from="95" weight="-1.37392485823133e-001"/><Con from="96" weight="-8.88682985440580e-001"/><Con from="97" weight="-3.20218711109330e-001"/><Con from="98" weight="8.95487221619550e-001"/><Con from="99" weight="1.67417633880828e+000"/></Neuron></NeuralLayer><NeuralLayer numberOfNeurons="2" activationFunction="exponential"><Neuron id="119" bias="2.42384718930389e-002"><Con from="100" weight="2.01317709619637e+000"/><Con from="101" weight="-7.64561209785197e-002"/><Con from="102" weight="6.62426607250352e-001"/><Con from="103" weight="-1.29526182492849e+000"/><Con from="104" weight="-2.32974880019959e+000"/><Con from="105" weight="-1.23472560937565e+000"/><Con from="106" weight="-1.73808174977928e+000"/><Con from="107" weight="3.96642874547339e+000"/><Con from="108" weight="-1.00154576394521e-001"/><Con from="109" weight="-7.65441802627678e-002"/><Con from="110" weight="1.20432273766182e+000"/><Con from="111" weight="9.66198764624965e-004"/><Con from="112" weight="2.24521269362687e-001"/><Con from="113" weight="-1.00466371042050e+000"/><Con from="114" weight="4.51137067879949e-001"/><Con from="115" weight="-3.34734146107152e-001"/><Con from="116" weight="-2.77412273450054e-003"/><Con from="117" weight="-1.69631320197821e+000"/><Con from="118" weight="1.60858559274275e-002"/></Neuron><Neuron id="120" bias="-1.27192946850012e-001"><Con from="100" weight="-2.51448172279428e-001"/><Con from="101" weight="-4.46642653774233e+000"/><Con from="102" weight="-1.18611757561983e-001"/><Con from="103" weight="1.36382126289548e-002"/><Con from="104" weight="8.94368471346068e-003"/><Con from="105" weight="3.84767593407379e-002"/><Con from="106" weight="2.47948317594928e+000"/><Con from="107" weight="-2.31914336314990e+000"/><Con from="108" weight="2.06178084408298e+000"/><Con from="109" weight="1.21888143927256e+000"/><Con from="110" weight="7.67314199230823e-001"/><Con from="111" weight="-8.37218236490709e-002"/><Con from="112" weight="-9.76028165104404e-002"/><Con from="113" weight="3.02911264786948e-001"/><Con from="114" weight="-1.73337256197557e-001"/><Con from="115" weight="7.48909390715133e-003"/><Con from="116" weight="-3.01141240762705e+000"/><Con from="117" weight="2.07610136434869e-003"/><Con from="118" weight="4.21128341899114e+000"/></Neuron></NeuralLayer><NeuralOutputs numberOfOutputs="2"><NeuralOutput outputNeuron="119"><DerivedField optype="categorical"><NormDiscrete field="[M] class" value="1"/></DerivedField></NeuralOutput><NeuralOutput outputNeuron="120"><DerivedField optype="categorical"><NormDiscrete field="[M] class" value="2"/></DerivedField></NeuralOutput></NeuralOutputs></NeuralNetwork></PMML>

## 1.2. MLP 100-23-2

<?xml version="1.0" encoding="UTF-8"?>

<PMML version="3.0"><Header copyright="Copyright (c) StatSoft, Inc. All Rights Reserved."><Application name="STATISTICA Automated Neural Networks (SANN)" version="2.0"/></Header><DataDictionary numberOfFields="101"><DataField name="[M] class" optype="categorical"><Value value="1"/><Value value="2"/></DataField><DataField name="nN" optype="continuous"/><DataField name="AATS6i" optype="continuous"/><DataField name="AATS7i" optype="continuous"/><DataField name="AATS8i" optype="continuous"/><DataField name="ATSC3c" optype="continuous"/><DataField name="ATSC0m" optype="continuous"/><DataField name="ATSC3e" optype="continuous"/><DataField name="AATSC0e" optype="continuous"/><DataField name="AATSC0i" optype="continuous"/><DataField name="MATS1c" optype="continuous"/><DataField name="MATS2s" optype="continuous"/><DataField name="GATS1c" optype="continuous"/><DataField name="GATS3c" optype="continuous"/><DataField name="GATS4e" optype="continuous"/><DataField name="GATS4i" optype="continuous"/><DataField name="GATS2s" optype="continuous"/><DataField name="GATS3s" optype="continuous"/><DataField name="GATS4s" optype="continuous"/><DataField name="VR2_Dzs" optype="continuous"/><DataField name="nBase" optype="continuous"/><DataField name="BCUTw-1l" optype="continuous"/><DataField name="BCUTc-1l" optype="continuous"/><DataField name="BCUTc-1h" optype="continuous"/><DataField name="C1SP2" optype="continuous"/><DataField name="C2SP2" optype="continuous"/><DataField name="C3SP2" optype="continuous"/><DataField name="C1SP3" optype="continuous"/><DataField name="C4SP3" optype="continuous"/><DataField name="VE1_Dt" optype="continuous"/><DataField name="nHCsatu" optype="continuous"/><DataField name="nsCH3" optype="continuous"/><DataField name="nsssN" optype="continuous"/><DataField name="ndS" optype="continuous"/><DataField name="nssS" optype="continuous"/><DataField name="SHCsatu" optype="continuous"/><DataField name="minHBd" optype="continuous"/><DataField name="minwHBa" optype="continuous"/><DataField name="minHBint2" optype="continuous"/><DataField name="minHssNH" optype="continuous"/><DataField name="minHdsCH" optype="continuous"/><DataField name="minHCsats" optype="continuous"/><DataField name="minHCsatu" optype="continuous"/><DataField name="minHother" optype="continuous"/><DataField name="minssCH2" optype="continuous"/><DataField name="minaaCH" optype="continuous"/><DataField name="minaasC" optype="continuous"/><DataField name="minsNH2" optype="continuous"/><DataField name="minsssN" optype="continuous"/><DataField name="mindO" optype="continuous"/><DataField name="minssO" optype="continuous"/><DataField name="maxHBd" optype="continuous"/><DataField name="maxHBa" optype="continuous"/><DataField name="maxwHBa" optype="continuous"/><DataField name="maxHBint2" optype="continuous"/><DataField name="maxHssNH" optype="continuous"/><DataField name="maxHdsCH" optype="continuous"/><DataField name="maxHCsats" optype="continuous"/><DataField name="maxHCsatu" optype="continuous"/><DataField name="maxHother" optype="continuous"/><DataField name="maxsCH3" optype="continuous"/><DataField name="maxaaCH" optype="continuous"/><DataField name="maxsssCH" optype="continuous"/><DataField name="maxdssC" optype="continuous"/><DataField name="maxaasC" optype="continuous"/><DataField name="maxssssC" optype="continuous"/><DataField name="maxsNH2" optype="continuous"/><DataField name="maxsssN" optype="continuous"/><DataField name="maxdO" optype="continuous"/><DataField name="maxssO" optype="continuous"/><DataField name="maxsCl" optype="continuous"/><DataField name="gmax" optype="continuous"/><DataField name="MAXDN2" optype="continuous"/><DataField name="MAXDP2" optype="continuous"/><DataField name="ETA_Shape_P" optype="continuous"/><DataField name="ETA_BetaP" optype="continuous"/><DataField name="ETA_BetaP_ns" optype="continuous"/><DataField name="ETA_BetaP_ns_d" optype="continuous"/><DataField name="FMF" optype="continuous"/><DataField name="nHBAcc" optype="continuous"/><DataField name="SIC5" optype="continuous"/><DataField name="BIC5" optype="continuous"/><DataField name="nAtomP" optype="continuous"/><DataField name="nAtomLAC" optype="continuous"/><DataField name="MDEO-12" optype="continuous"/><DataField name="MLFER_S" optype="continuous"/><DataField name="MLFER_E" optype="continuous"/><DataField name="piPC4" optype="continuous"/><DataField name="piPC5" optype="continuous"/><DataField name="piPC6" optype="continuous"/><DataField name="piPC8" optype="continuous"/><DataField name="piPC9" optype="continuous"/><DataField name="piPC10" optype="continuous"/><DataField name="n6Ring" optype="continuous"/><DataField name="RotBFrac" optype="continuous"/><DataField name="RotBtFrac" optype="continuous"/><DataField name="LipinskiFailures" optype="continuous"/><DataField name="GGI6" optype="continuous"/><DataField name="GGI8" optype="continuous"/><DataField name="JGT" optype="continuous"/><DataField name="WTPT-5" optype="continuous"/></DataDictionary><NeuralNetwork modelName="Ark_MLP 100-23-2" functionName="classification"><MiningSchema><MiningField name="[M] class" usageType="predicted"/><MiningField name="nN" lowValue="0.000000" highValue="6.000000"/><MiningField name="AATS6i" lowValue="0.000000" highValue="197.783615"/><MiningField name="AATS7i" lowValue="0.000000" highValue="197.641130"/><MiningField name="AATS8i" lowValue="0.000000" highValue="197.641130"/><MiningField name="ATSC3c" lowValue="-0.709297" highValue="0.477175"/><MiningField name="ATSC0m" lowValue="337.724329" highValue="5532.361122"/><MiningField name="ATSC3e" lowValue="-4.603184" highValue="4.952578"/><MiningField name="AATSC0e" lowValue="0.016140" highValue="0.218137"/><MiningField name="AATSC0i" lowValue="0.466396" highValue="2.091475"/><MiningField name="MATS1c" lowValue="-1.042399" highValue="0.161674"/><MiningField name="MATS2s" lowValue="-1.343888" highValue="0.732539"/><MiningField name="GATS1c" lowValue="0.611983" highValue="2.500000"/><MiningField name="GATS3c" lowValue="0.000000" highValue="2.472448"/><MiningField name="GATS4e" lowValue="0.000000" highValue="3.344208"/><MiningField name="GATS4i" lowValue="0.000000" highValue="2.045863"/><MiningField name="GATS2s" lowValue="0.000000" highValue="2.123355"/><MiningField name="GATS3s" lowValue="0.000000" highValue="3.206156"/><MiningField name="GATS4s" lowValue="0.000000" highValue="4.782779"/><MiningField name="VR2_Dzs" lowValue="1.304698" highValue="800000.000000"/><MiningField name="nBase" lowValue="0.000000" highValue="6.000000"/><MiningField name="BCUTw-1l" lowValue="11.690000" highValue="12.000000"/><MiningField name="BCUTc-1l" lowValue="-0.419857" highValue="-0.118299"/><MiningField name="BCUTc-1h" lowValue="0.049763" highValue="0.530105"/><MiningField name="C1SP2" lowValue="0.000000" highValue="5.000000"/><MiningField name="C2SP2" lowValue="0.000000" highValue="17.000000"/><MiningField name="C3SP2" lowValue="0.000000" highValue="6.000000"/><MiningField name="C1SP3" lowValue="0.000000" highValue="9.000000"/><MiningField name="C4SP3" lowValue="0.000000" highValue="2.000000"/><MiningField name="VE1_Dt" lowValue="0.000000" highValue="0.484850"/><MiningField name="nHCsatu" lowValue="0.000000" highValue="4.000000"/><MiningField name="nsCH3" lowValue="0.000000" highValue="6.000000"/><MiningField name="nsssN" lowValue="0.000000" highValue="4.000000"/><MiningField name="ndS" lowValue="0.000000" highValue="1.000000"/><MiningField name="nssS" lowValue="0.000000" highValue="3.000000"/><MiningField name="SHCsatu" lowValue="0.000000" highValue="2.382460"/><MiningField name="minHBd" lowValue="0.000000" highValue="0.869082"/><MiningField name="minwHBa" lowValue="-1.399568" highValue="3.235284"/><MiningField name="minHBint2" lowValue="-0.511389" highValue="8.289887"/><MiningField name="minHssNH" lowValue="0.000000" highValue="0.677623"/><MiningField name="minHdsCH" lowValue="0.000000" highValue="0.757925"/><MiningField name="minHCsats" lowValue="0.000000" highValue="0.936911"/><MiningField name="minHCsatu" lowValue="0.000000" highValue="1.041473"/><MiningField name="minHother" lowValue="0.000000" highValue="0.760540"/><MiningField name="minssCH2" lowValue="-0.673675" highValue="1.727730"/><MiningField name="minaaCH" lowValue="0.000000" highValue="2.620150"/><MiningField name="minaasC" lowValue="-0.589846" highValue="1.951475"/><MiningField name="minsNH2" lowValue="0.000000" highValue="6.075669"/><MiningField name="minsssN" lowValue="0.000000" highValue="2.664352"/><MiningField name="mindO" lowValue="0.000000" highValue="13.824902"/><MiningField name="minssO" lowValue="0.000000" highValue="6.570147"/><MiningField name="maxHBd" lowValue="0.000000" highValue="0.869082"/><MiningField name="maxHBa" lowValue="0.531293" highValue="13.824902"/><MiningField name="maxwHBa" lowValue="0.000000" highValue="5.432183"/><MiningField name="maxHBint2" lowValue="0.000000" highValue="8.289887"/><MiningField name="maxHssNH" lowValue="0.000000" highValue="0.677623"/><MiningField name="maxHdsCH" lowValue="0.000000" highValue="0.757925"/><MiningField name="maxHCsats" lowValue="0.000000" highValue="1.011564"/><MiningField name="maxHCsatu" lowValue="0.000000" highValue="1.147376"/><MiningField name="maxHother" lowValue="0.000000" highValue="0.786998"/><MiningField name="maxsCH3" lowValue="0.000000" highValue="2.473813"/><MiningField name="maxaaCH" lowValue="0.000000" highValue="2.636307"/><MiningField name="maxsssCH" lowValue="0.000000" highValue="1.366427"/><MiningField name="maxdssC" lowValue="0.000000" highValue="2.211231"/><MiningField name="maxaasC" lowValue="0.000000" highValue="2.077332"/><MiningField name="maxssssC" lowValue="0.000000" highValue="1.365569"/><MiningField name="maxsNH2" lowValue="0.000000" highValue="6.075669"/><MiningField name="maxsssN" lowValue="0.000000" highValue="2.664352"/><MiningField name="maxdO" lowValue="0.000000" highValue="13.824902"/><MiningField name="maxssO" lowValue="0.000000" highValue="6.574487"/><MiningField name="maxsCl" lowValue="0.000000" highValue="1.588820"/><MiningField name="gmax" lowValue="1.350137" highValue="13.824902"/><MiningField name="MAXDN2" lowValue="0.420972" highValue="5.264383"/><MiningField name="MAXDP2" lowValue="0.415123" highValue="6.810793"/><MiningField name="ETA_Shape_P" lowValue="0.062580" highValue="0.851060"/><MiningField name="ETA_BetaP" lowValue="0.437500" highValue="1.750000"/><MiningField name="ETA_BetaP_ns" lowValue="0.000000" highValue="1.192310"/><MiningField name="ETA_BetaP_ns_d" lowValue="0.000000" highValue="0.250000"/><MiningField name="FMF" lowValue="0.000000" highValue="0.642857"/><MiningField name="nHBAcc" lowValue="0.000000" highValue="9.000000"/><MiningField name="SIC5" lowValue="0.000000" highValue="1.000000"/><MiningField name="BIC5" lowValue="0.000000" highValue="0.933062"/><MiningField name="nAtomP" lowValue="0.000000" highValue="25.000000"/><MiningField name="nAtomLAC" lowValue="0.000000" highValue="20.000000"/><MiningField name="MDEO-12" lowValue="0.000000" highValue="2.149140"/><MiningField name="MLFER_S" lowValue="0.166000" highValue="4.011000"/><MiningField name="MLFER_E" lowValue="0.131000" highValue="2.888000"/><MiningField name="piPC4" lowValue="0.000000" highValue="5.794156"/><MiningField name="piPC5" lowValue="0.000000" highValue="6.557754"/><MiningField name="piPC6" lowValue="0.000000" highValue="7.071600"/><MiningField name="piPC8" lowValue="0.000000" highValue="8.204505"/><MiningField name="piPC9" lowValue="0.000000" highValue="8.773305"/><MiningField name="piPC10" lowValue="0.000000" highValue="9.038376"/><MiningField name="n6Ring" lowValue="0.000000" highValue="4.000000"/><MiningField name="RotBFrac" lowValue="0.000000" highValue="0.900000"/><MiningField name="RotBtFrac" lowValue="0.000000" highValue="1.000000"/><MiningField name="LipinskiFailures" lowValue="0.000000" highValue="2.000000"/><MiningField name="GGI6" lowValue="0.000000" highValue="1.144580"/><MiningField name="GGI8" lowValue="0.000000" highValue="0.454775"/><MiningField name="JGT" lowValue="0.075666" highValue="0.926282"/><MiningField name="WTPT-5" lowValue="0.000000" highValue="19.461628"/></MiningSchema><NeuralInputs numberOfInputs="100"><NeuralInput id="0"><DerivedField><NormContinuous field="nN" shift="-0.00000000000000e+000" scale="1.66666666666667e-001"><LinearNorm orig="0.00000000000000e+000" norm="0.000000"/><LinearNorm orig="6.00000000000000e+000" norm="1.000000"/></NormContinuous></DerivedField></NeuralInput><NeuralInput id="1"><DerivedField><NormContinuous field="AATS6i" shift="-0.00000000000000e+000" scale="5.05603053971999e-003"><LinearNorm orig="0.00000000000000e+000" norm="0.000000"/><LinearNorm orig="1.97783615455650e+002" norm="1.000000"/></NormContinuous></DerivedField></NeuralInput><NeuralInput id="2"><DerivedField><NormContinuous field="AATS7i" shift="-0.00000000000000e+000" scale="5.05967557433139e-003"><LinearNorm orig="0.00000000000000e+000" norm="0.000000"/><LinearNorm orig="1.97641130406300e+002" norm="1.000000"/></NormContinuous></DerivedField></NeuralInput><NeuralInput id="3"><DerivedField><NormContinuous field="AATS8i" shift="-0.00000000000000e+000" scale="5.05967557433139e-003"><LinearNorm orig="0.00000000000000e+000" norm="0.000000"/><LinearNorm orig="1.97641130406300e+002" norm="1.000000"/></NormContinuous></DerivedField></NeuralInput><NeuralInput id="4"><DerivedField><NormContinuous field="ATSC3c" shift="5.97820440549968e-001" scale="8.42835077233181e-001"><LinearNorm orig="-7.09297057868622e-001" norm="0.000000"/><LinearNorm orig="4.77174681398273e-001" norm="1.000000"/></NormContinuous></DerivedField></NeuralInput><NeuralInput id="5"><DerivedField><NormContinuous field="ATSC0m" shift="-6.50140408461115e-002" scale="1.92506240526961e-004"><LinearNorm orig="3.37724328666666e+002" norm="0.000000"/><LinearNorm orig="5.53236112206428e+003" norm="1.000000"/></NormContinuous></DerivedField></NeuralInput><NeuralInput id="6"><DerivedField><NormContinuous field="ATSC3e" shift="4.81718150076887e-001" scale="1.04648892923010e-001"><LinearNorm orig="-4.60318438754325e+000" norm="0.000000"/><LinearNorm orig="4.95257843104379e+000" norm="1.000000"/></NormContinuous></DerivedField></NeuralInput><NeuralInput id="7"><DerivedField><NormContinuous field="AATSC0e" shift="-7.99034931570863e-002" scale="4.95056802602201e+000"><LinearNorm orig="1.61402676899063e-002" norm="0.000000"/><LinearNorm orig="2.18137289999999e-001" norm="1.000000"/></NormContinuous></DerivedField></NeuralInput><NeuralInput id="8"><DerivedField><NormContinuous field="AATSC0i" shift="-2.86998985772790e-001" scale="6.15354578529142e-001"><LinearNorm orig="4.66396116623999e-001" norm="0.000000"/><LinearNorm orig="2.09147543656708e+000" norm="1.000000"/></NormContinuous></DerivedField></NeuralInput><NeuralInput id="9"><DerivedField><NormContinuous field="MATS1c" shift="8.65727166044417e-001" scale="8.30513877894299e-001"><LinearNorm orig="-1.04239939763487e+000" norm="0.000000"/><LinearNorm orig="1.61674401270718e-001" norm="1.000000"/></NormContinuous></DerivedField></NeuralInput><NeuralInput id="10"><DerivedField><NormContinuous field="MATS2s" shift="6.47211803411582e-001" scale="4.81596712187758e-001"><LinearNorm orig="-1.34388750386497e+000" norm="0.000000"/><LinearNorm orig="7.32538631723213e-001" norm="1.000000"/></NormContinuous></DerivedField></NeuralInput><NeuralInput id="11"><DerivedField><NormContinuous field="GATS1c" shift="-3.24140792326252e-001" scale="5.29656316930503e-001"><LinearNorm orig="6.11983246428047e-001" norm="0.000000"/><LinearNorm orig="2.49999999999999e+000" norm="1.000000"/></NormContinuous></DerivedField></NeuralInput><NeuralInput id="12"><DerivedField><NormContinuous field="GATS3c" shift="-0.00000000000000e+000" scale="4.04457445713418e-001"><LinearNorm orig="0.00000000000000e+000" norm="0.000000"/><LinearNorm orig="2.47244799322735e+000" norm="1.000000"/></NormContinuous></DerivedField></NeuralInput><NeuralInput id="13"><DerivedField><NormContinuous field="GATS4e" shift="-0.00000000000000e+000" scale="2.99024461462626e-001"><LinearNorm orig="0.00000000000000e+000" norm="0.000000"/><LinearNorm orig="3.34420801264443e+000" norm="1.000000"/></NormContinuous></DerivedField></NeuralInput><NeuralInput id="14"><DerivedField><NormContinuous field="GATS4i" shift="-0.00000000000000e+000" scale="4.88791247683768e-001"><LinearNorm orig="0.00000000000000e+000" norm="0.000000"/><LinearNorm orig="2.04586314656552e+000" norm="1.000000"/></NormContinuous></DerivedField></NeuralInput><NeuralInput id="15"><DerivedField><NormContinuous field="GATS2s" shift="-0.00000000000000e+000" scale="4.70952705983061e-001"><LinearNorm orig="0.00000000000000e+000" norm="0.000000"/><LinearNorm orig="2.12335546074125e+000" norm="1.000000"/></NormContinuous></DerivedField></NeuralInput><NeuralInput id="16"><DerivedField><NormContinuous field="GATS3s" shift="-0.00000000000000e+000" scale="3.11899935981828e-001"><LinearNorm orig="0.00000000000000e+000" norm="0.000000"/><LinearNorm orig="3.20615647724359e+000" norm="1.000000"/></NormContinuous></DerivedField></NeuralInput><NeuralInput id="17"><DerivedField><NormContinuous field="GATS4s" shift="-0.00000000000000e+000" scale="2.09083445127794e-001"><LinearNorm orig="0.00000000000000e+000" norm="0.000000"/><LinearNorm orig="4.78277942755720e+000" norm="1.000000"/></NormContinuous></DerivedField></NeuralInput><NeuralInput id="18"><DerivedField><NormContinuous field="VR2_Dzs" shift="-1.63087485462995e-006" scale="1.25000203859357e-006"><LinearNorm orig="1.30469775590520e+000" norm="0.000000"/><LinearNorm orig="8.00000000000000e+005" norm="1.000000"/></NormContinuous></DerivedField></NeuralInput><NeuralInput id="19"><DerivedField><NormContinuous field="nBase" shift="-0.00000000000000e+000" scale="1.66666666666667e-001"><LinearNorm orig="0.00000000000000e+000" norm="0.000000"/><LinearNorm orig="6.00000000000000e+000" norm="1.000000"/></NormContinuous></DerivedField></NeuralInput><NeuralInput id="20"><DerivedField><NormContinuous field="BCUTw-1l" shift="-3.77097046318940e+001" scale="3.22580877946057e+000"><LinearNorm orig="1.16900000000000e+001" norm="0.000000"/><LinearNorm orig="1.19999997762940e+001" norm="1.000000"/></NormContinuous></DerivedField></NeuralInput><NeuralInput id="21"><DerivedField><NormContinuous field="BCUTc-1l" shift="1.39229140590098e+000" scale="3.31610617756096e+000"><LinearNorm orig="-4.19857305933739e-001" norm="0.000000"/><LinearNorm orig="-1.18298807364941e-001" norm="1.000000"/></NormContinuous></DerivedField></NeuralInput><NeuralInput id="22"><DerivedField><NormContinuous field="BCUTc-1h" shift="-1.03599084775896e-001" scale="2.08184959336469e+000"><LinearNorm orig="4.97630016626027e-002" norm="0.000000"/><LinearNorm orig="5.30105098991448e-001" norm="1.000000"/></NormContinuous></DerivedField></NeuralInput><NeuralInput id="23"><DerivedField><NormContinuous field="C1SP2" shift="-0.00000000000000e+000" scale="2.00000000000000e-001"><LinearNorm orig="0.00000000000000e+000" norm="0.000000"/><LinearNorm orig="5.00000000000000e+000" norm="1.000000"/></NormContinuous></DerivedField></NeuralInput><NeuralInput id="24"><DerivedField><NormContinuous field="C2SP2" shift="-0.00000000000000e+000" scale="5.88235294117647e-002"><LinearNorm orig="0.00000000000000e+000" norm="0.000000"/><LinearNorm orig="1.70000000000000e+001" norm="1.000000"/></NormContinuous></DerivedField></NeuralInput><NeuralInput id="25"><DerivedField><NormContinuous field="C3SP2" shift="-0.00000000000000e+000" scale="1.66666666666667e-001"><LinearNorm orig="0.00000000000000e+000" norm="0.000000"/><LinearNorm orig="6.00000000000000e+000" norm="1.000000"/></NormContinuous></DerivedField></NeuralInput><NeuralInput id="26"><DerivedField><NormContinuous field="C1SP3" shift="-0.00000000000000e+000" scale="1.11111111111111e-001"><LinearNorm orig="0.00000000000000e+000" norm="0.000000"/><LinearNorm orig="9.00000000000000e+000" norm="1.000000"/></NormContinuous></DerivedField></NeuralInput><NeuralInput id="27"><DerivedField><NormContinuous field="C4SP3" shift="-0.00000000000000e+000" scale="5.00000000000000e-001"><LinearNorm orig="0.00000000000000e+000" norm="0.000000"/><LinearNorm orig="2.00000000000000e+000" norm="1.000000"/></NormContinuous></DerivedField></NeuralInput><NeuralInput id="28"><DerivedField><NormContinuous field="VE1_Dt" shift="-0.00000000000000e+000" scale="2.06249354028337e+000"><LinearNorm orig="0.00000000000000e+000" norm="0.000000"/><LinearNorm orig="4.84850003390852e-001" norm="1.000000"/></NormContinuous></DerivedField></NeuralInput><NeuralInput id="29"><DerivedField><NormContinuous field="nHCsatu" shift="-0.00000000000000e+000" scale="2.50000000000000e-001"><LinearNorm orig="0.00000000000000e+000" norm="0.000000"/><LinearNorm orig="4.00000000000000e+000" norm="1.000000"/></NormContinuous></DerivedField></NeuralInput><NeuralInput id="30"><DerivedField><NormContinuous field="nsCH3" shift="-0.00000000000000e+000" scale="1.66666666666667e-001"><LinearNorm orig="0.00000000000000e+000" norm="0.000000"/><LinearNorm orig="6.00000000000000e+000" norm="1.000000"/></NormContinuous></DerivedField></NeuralInput><NeuralInput id="31"><DerivedField><NormContinuous field="nsssN" shift="-0.00000000000000e+000" scale="2.50000000000000e-001"><LinearNorm orig="0.00000000000000e+000" norm="0.000000"/><LinearNorm orig="4.00000000000000e+000" norm="1.000000"/></NormContinuous></DerivedField></NeuralInput><NeuralInput id="32"><DerivedField><NormContinuous field="ndS" shift="-0.00000000000000e+000" scale="1.00000000000000e+000"><LinearNorm orig="0.00000000000000e+000" norm="0.000000"/><LinearNorm orig="1.00000000000000e+000" norm="1.000000"/></NormContinuous></DerivedField></NeuralInput><NeuralInput id="33"><DerivedField><NormContinuous field="nssS" shift="-0.00000000000000e+000" scale="3.33333333333333e-001"><LinearNorm orig="0.00000000000000e+000" norm="0.000000"/><LinearNorm orig="3.00000000000000e+000" norm="1.000000"/></NormContinuous></DerivedField></NeuralInput><NeuralInput id="34"><DerivedField><NormContinuous field="SHCsatu" shift="-0.00000000000000e+000" scale="4.19734199013930e-001"><LinearNorm orig="0.00000000000000e+000" norm="0.000000"/><LinearNorm orig="2.38246014346525e+000" norm="1.000000"/></NormContinuous></DerivedField></NeuralInput><NeuralInput id="35"><DerivedField><NormContinuous field="minHBd" shift="-0.00000000000000e+000" scale="1.15063968819085e+000"><LinearNorm orig="0.00000000000000e+000" norm="0.000000"/><LinearNorm orig="8.69081790123456e-001" norm="1.000000"/></NormContinuous></DerivedField></NeuralInput><NeuralInput id="36"><DerivedField><NormContinuous field="minwHBa" shift="3.01966043162451e-001" scale="2.15756622380441e-001"><LinearNorm orig="-1.39956790123456e+000" norm="0.000000"/><LinearNorm orig="3.23528403965610e+000" norm="1.000000"/></NormContinuous></DerivedField></NeuralInput><NeuralInput id="37"><DerivedField><NormContinuous field="minHBint2" shift="5.81039178269867e-002" scale="1.13619895821466e-001"><LinearNorm orig="-5.11388585660093e-001" norm="0.000000"/><LinearNorm orig="8.28988686676002e+000" norm="1.000000"/></NormContinuous></DerivedField></NeuralInput><NeuralInput id="38"><DerivedField><NormContinuous field="minHssNH" shift="-0.00000000000000e+000" scale="1.47574584377135e+000"><LinearNorm orig="0.00000000000000e+000" norm="0.000000"/><LinearNorm orig="6.77623456790123e-001" norm="1.000000"/></NormContinuous></DerivedField></NeuralInput><NeuralInput id="39"><DerivedField><NormContinuous field="minHdsCH" shift="-0.00000000000000e+000" scale="1.31939134251236e+000"><LinearNorm orig="0.00000000000000e+000" norm="0.000000"/><LinearNorm orig="7.57925240054869e-001" norm="1.000000"/></NormContinuous></DerivedField></NeuralInput><NeuralInput id="40"><DerivedField><NormContinuous field="minHCsats" shift="-0.00000000000000e+000" scale="1.06733668654208e+000"><LinearNorm orig="0.00000000000000e+000" norm="0.000000"/><LinearNorm orig="9.36911485015816e-001" norm="1.000000"/></NormContinuous></DerivedField></NeuralInput><NeuralInput id="41"><DerivedField><NormContinuous field="minHCsatu" shift="-0.00000000000000e+000" scale="9.60178391419969e-001"><LinearNorm orig="0.00000000000000e+000" norm="0.000000"/><LinearNorm orig="1.04147313555051e+000" norm="1.000000"/></NormContinuous></DerivedField></NeuralInput><NeuralInput id="42"><DerivedField><NormContinuous field="minHother" shift="-0.00000000000000e+000" scale="1.31485502100116e+000"><LinearNorm orig="0.00000000000000e+000" norm="0.000000"/><LinearNorm orig="7.60540123456790e-001" norm="1.000000"/></NormContinuous></DerivedField></NeuralInput><NeuralInput id="43"><DerivedField><NormContinuous field="minssCH2" shift="2.80533634802749e-001" scale="4.16422972516244e-001"><LinearNorm orig="-6.73674732946693e-001" norm="0.000000"/><LinearNorm orig="1.72772976680384e+000" norm="1.000000"/></NormContinuous></DerivedField></NeuralInput><NeuralInput id="44"><DerivedField><NormContinuous field="minaaCH" shift="-0.00000000000000e+000" scale="3.81657569375851e-001"><LinearNorm orig="0.00000000000000e+000" norm="0.000000"/><LinearNorm orig="2.62014978933960e+000" norm="1.000000"/></NormContinuous></DerivedField></NeuralInput><NeuralInput id="45"><DerivedField><NormContinuous field="minaasC" shift="2.32102060727429e-001" scale="3.93496246537005e-001"><LinearNorm orig="-5.89845679012345e-001" norm="0.000000"/><LinearNorm orig="1.95147462277091e+000" norm="1.000000"/></NormContinuous></DerivedField></NeuralInput><NeuralInput id="46"><DerivedField><NormContinuous field="minsNH2" shift="-0.00000000000000e+000" scale="1.64590935645283e-001"><LinearNorm orig="0.00000000000000e+000" norm="0.000000"/><LinearNorm orig="6.07566872427983e+000" norm="1.000000"/></NormContinuous></DerivedField></NeuralInput><NeuralInput id="47"><DerivedField><NormContinuous field="minsssN" shift="-0.00000000000000e+000" scale="3.75325803649001e-001"><LinearNorm orig="0.00000000000000e+000" norm="0.000000"/><LinearNorm orig="2.66435185185185e+000" norm="1.000000"/></NormContinuous></DerivedField></NeuralInput><NeuralInput id="48"><DerivedField><NormContinuous field="mindO" shift="-0.00000000000000e+000" scale="7.23332425725208e-002"><LinearNorm orig="0.00000000000000e+000" norm="0.000000"/><LinearNorm orig="1.38249021395302e+001" norm="1.000000"/></NormContinuous></DerivedField></NeuralInput><NeuralInput id="49"><DerivedField><NormContinuous field="minssO" shift="-0.00000000000000e+000" scale="1.52203585344402e-001"><LinearNorm orig="0.00000000000000e+000" norm="0.000000"/><LinearNorm orig="6.57014746227709e+000" norm="1.000000"/></NormContinuous></DerivedField></NeuralInput><NeuralInput id="50"><DerivedField><NormContinuous field="maxHBd" shift="-0.00000000000000e+000" scale="1.15063968819085e+000"><LinearNorm orig="0.00000000000000e+000" norm="0.000000"/><LinearNorm orig="8.69081790123456e-001" norm="1.000000"/></NormContinuous></DerivedField></NeuralInput><NeuralInput id="51"><DerivedField><NormContinuous field="maxHBa" shift="-3.99660360137496e-002" scale="7.52241155501655e-002"><LinearNorm orig="5.31292866941015e-001" norm="0.000000"/><LinearNorm orig="1.38249021395302e+001" norm="1.000000"/></NormContinuous></DerivedField></NeuralInput><NeuralInput id="52"><DerivedField><NormContinuous field="maxwHBa" shift="-0.00000000000000e+000" scale="1.84088037759979e-001"><LinearNorm orig="0.00000000000000e+000" norm="0.000000"/><LinearNorm orig="5.43218349311670e+000" norm="1.000000"/></NormContinuous></DerivedField></NeuralInput><NeuralInput id="53"><DerivedField><NormContinuous field="maxHBint2" shift="-0.00000000000000e+000" scale="1.20628907978190e-001"><LinearNorm orig="0.00000000000000e+000" norm="0.000000"/><LinearNorm orig="8.28988686676002e+000" norm="1.000000"/></NormContinuous></DerivedField></NeuralInput><NeuralInput id="54"><DerivedField><NormContinuous field="maxHssNH" shift="-0.00000000000000e+000" scale="1.47574584377135e+000"><LinearNorm orig="0.00000000000000e+000" norm="0.000000"/><LinearNorm orig="6.77623456790123e-001" norm="1.000000"/></NormContinuous></DerivedField></NeuralInput><NeuralInput id="55"><DerivedField><NormContinuous field="maxHdsCH" shift="-0.00000000000000e+000" scale="1.31939134251236e+000"><LinearNorm orig="0.00000000000000e+000" norm="0.000000"/><LinearNorm orig="7.57925240054869e-001" norm="1.000000"/></NormContinuous></DerivedField></NeuralInput><NeuralInput id="56"><DerivedField><NormContinuous field="maxHCsats" shift="-0.00000000000000e+000" scale="9.88567940546206e-001"><LinearNorm orig="0.00000000000000e+000" norm="0.000000"/><LinearNorm orig="1.01156426279359e+000" norm="1.000000"/></NormContinuous></DerivedField></NeuralInput><NeuralInput id="57"><DerivedField><NormContinuous field="maxHCsatu" shift="-0.00000000000000e+000" scale="8.71553941810767e-001"><LinearNorm orig="0.00000000000000e+000" norm="0.000000"/><LinearNorm orig="1.14737591332829e+000" norm="1.000000"/></NormContinuous></DerivedField></NeuralInput><NeuralInput id="58"><DerivedField><NormContinuous field="maxHother" shift="-0.00000000000000e+000" scale="1.27065052208442e+000"><LinearNorm orig="0.00000000000000e+000" norm="0.000000"/><LinearNorm orig="7.86998456790123e-001" norm="1.000000"/></NormContinuous></DerivedField></NeuralInput><NeuralInput id="59"><DerivedField><NormContinuous field="maxsCH3" shift="-0.00000000000000e+000" scale="4.04234200764108e-001"><LinearNorm orig="0.00000000000000e+000" norm="0.000000"/><LinearNorm orig="2.47381344307270e+000" norm="1.000000"/></NormContinuous></DerivedField></NeuralInput><NeuralInput id="60"><DerivedField><NormContinuous field="maxaaCH" shift="-0.00000000000000e+000" scale="3.79318553438257e-001"><LinearNorm orig="0.00000000000000e+000" norm="0.000000"/><LinearNorm orig="2.63630658436214e+000" norm="1.000000"/></NormContinuous></DerivedField></NeuralInput><NeuralInput id="61"><DerivedField><NormContinuous field="maxsssCH" shift="-0.00000000000000e+000" scale="7.31835863972896e-001"><LinearNorm orig="0.00000000000000e+000" norm="0.000000"/><LinearNorm orig="1.36642661179698e+000" norm="1.000000"/></NormContinuous></DerivedField></NeuralInput><NeuralInput id="62"><DerivedField><NormContinuous field="maxdssC" shift="-0.00000000000000e+000" scale="4.52236757419025e-001"><LinearNorm orig="0.00000000000000e+000" norm="0.000000"/><LinearNorm orig="2.21123113854595e+000" norm="1.000000"/></NormContinuous></DerivedField></NeuralInput><NeuralInput id="63"><DerivedField><NormContinuous field="maxaasC" shift="-0.00000000000000e+000" scale="4.81386710689228e-001"><LinearNorm orig="0.00000000000000e+000" norm="0.000000"/><LinearNorm orig="2.07733196159122e+000" norm="1.000000"/></NormContinuous></DerivedField></NeuralInput><NeuralInput id="64"><DerivedField><NormContinuous field="maxssssC" shift="-0.00000000000000e+000" scale="7.32295328980412e-001"><LinearNorm orig="0.00000000000000e+000" norm="0.000000"/><LinearNorm orig="1.36556927297668e+000" norm="1.000000"/></NormContinuous></DerivedField></NeuralInput><NeuralInput id="65"><DerivedField><NormContinuous field="maxsNH2" shift="-0.00000000000000e+000" scale="1.64590935645283e-001"><LinearNorm orig="0.00000000000000e+000" norm="0.000000"/><LinearNorm orig="6.07566872427983e+000" norm="1.000000"/></NormContinuous></DerivedField></NeuralInput><NeuralInput id="66"><DerivedField><NormContinuous field="maxsssN" shift="-0.00000000000000e+000" scale="3.75325803649001e-001"><LinearNorm orig="0.00000000000000e+000" norm="0.000000"/><LinearNorm orig="2.66435185185185e+000" norm="1.000000"/></NormContinuous></DerivedField></NeuralInput><NeuralInput id="67"><DerivedField><NormContinuous field="maxdO" shift="-0.00000000000000e+000" scale="7.23332425725208e-002"><LinearNorm orig="0.00000000000000e+000" norm="0.000000"/><LinearNorm orig="1.38249021395302e+001" norm="1.000000"/></NormContinuous></DerivedField></NeuralInput><NeuralInput id="68"><DerivedField><NormContinuous field="maxssO" shift="-0.00000000000000e+000" scale="1.52103115062408e-001"><LinearNorm orig="0.00000000000000e+000" norm="0.000000"/><LinearNorm orig="6.57448731138545e+000" norm="1.000000"/></NormContinuous></DerivedField></NeuralInput><NeuralInput id="69"><DerivedField><NormContinuous field="maxsCl" shift="-0.00000000000000e+000" scale="6.29397798402765e-001"><LinearNorm orig="0.00000000000000e+000" norm="0.000000"/><LinearNorm orig="1.58882030178326e+000" norm="1.000000"/></NormContinuous></DerivedField></NeuralInput><NeuralInput id="70"><DerivedField><NormContinuous field="gmax" shift="-1.08229467886950e-001" scale="8.01618309266824e-002"><LinearNorm orig="1.35013717421124e+000" norm="0.000000"/><LinearNorm orig="1.38249021395302e+001" norm="1.000000"/></NormContinuous></DerivedField></NeuralInput><NeuralInput id="71"><DerivedField><NormContinuous field="MAXDN2" shift="-8.69164757836136e-002" scale="2.06466059268234e-001"><LinearNorm orig="4.20972222222222e-001" norm="0.000000"/><LinearNorm orig="5.26438330656336e+000" norm="1.000000"/></NormContinuous></DerivedField></NeuralInput><NeuralInput id="72"><DerivedField><NormContinuous field="MAXDP2" shift="-6.49069604801291e-002" scale="1.56355800710497e-001"><LinearNorm orig="4.15123456790123e-001" norm="0.000000"/><LinearNorm orig="6.81079279208754e+000" norm="1.000000"/></NormContinuous></DerivedField></NeuralInput><NeuralInput id="73"><DerivedField><NormContinuous field="ETA_Shape_P" shift="-7.93678977272727e-002" scale="1.26826298701299e+000"><LinearNorm orig="6.25800000000000e-002" norm="0.000000"/><LinearNorm orig="8.51060000000000e-001" norm="1.000000"/></NormContinuous></DerivedField></NeuralInput><NeuralInput id="74"><DerivedField><NormContinuous field="ETA_BetaP" shift="-3.33333333333333e-001" scale="7.61904761904762e-001"><LinearNorm orig="4.37500000000000e-001" norm="0.000000"/><LinearNorm orig="1.75000000000000e+000" norm="1.000000"/></NormContinuous></DerivedField></NeuralInput><NeuralInput id="75"><DerivedField><NormContinuous field="ETA_BetaP_ns" shift="-0.00000000000000e+000" scale="8.38708054113444e-001"><LinearNorm orig="0.00000000000000e+000" norm="0.000000"/><LinearNorm orig="1.19231000000000e+000" norm="1.000000"/></NormContinuous></DerivedField></NeuralInput><NeuralInput id="76"><DerivedField><NormContinuous field="ETA_BetaP_ns_d" shift="-0.00000000000000e+000" scale="4.00000000000000e+000"><LinearNorm orig="0.00000000000000e+000" norm="0.000000"/><LinearNorm orig="2.50000000000000e-001" norm="1.000000"/></NormContinuous></DerivedField></NeuralInput><NeuralInput id="77"><DerivedField><NormContinuous field="FMF" shift="-0.00000000000000e+000" scale="1.55555555555556e+000"><LinearNorm orig="0.00000000000000e+000" norm="0.000000"/><LinearNorm orig="6.42857142857142e-001" norm="1.000000"/></NormContinuous></DerivedField></NeuralInput><NeuralInput id="78"><DerivedField><NormContinuous field="nHBAcc" shift="-0.00000000000000e+000" scale="1.11111111111111e-001"><LinearNorm orig="0.00000000000000e+000" norm="0.000000"/><LinearNorm orig="9.00000000000000e+000" norm="1.000000"/></NormContinuous></DerivedField></NeuralInput><NeuralInput id="79"><DerivedField><NormContinuous field="SIC5" shift="-0.00000000000000e+000" scale="1.00000000000000e+000"><LinearNorm orig="0.00000000000000e+000" norm="0.000000"/><LinearNorm orig="9.99999999999999e-001" norm="1.000000"/></NormContinuous></DerivedField></NeuralInput><NeuralInput id="80"><DerivedField><NormContinuous field="BIC5" shift="-0.00000000000000e+000" scale="1.07173999864482e+000"><LinearNorm orig="0.00000000000000e+000" norm="0.000000"/><LinearNorm orig="9.33062124455990e-001" norm="1.000000"/></NormContinuous></DerivedField></NeuralInput><NeuralInput id="81"><DerivedField><NormContinuous field="nAtomP" shift="-0.00000000000000e+000" scale="4.00000000000000e-002"><LinearNorm orig="0.00000000000000e+000" norm="0.000000"/><LinearNorm orig="2.50000000000000e+001" norm="1.000000"/></NormContinuous></DerivedField></NeuralInput><NeuralInput id="82"><DerivedField><NormContinuous field="nAtomLAC" shift="-0.00000000000000e+000" scale="5.00000000000000e-002"><LinearNorm orig="0.00000000000000e+000" norm="0.000000"/><LinearNorm orig="2.00000000000000e+001" norm="1.000000"/></NormContinuous></DerivedField></NeuralInput><NeuralInput id="83"><DerivedField><NormContinuous field="MDEO-12" shift="-0.00000000000000e+000" scale="4.65302429551051e-001"><LinearNorm orig="0.00000000000000e+000" norm="0.000000"/><LinearNorm orig="2.14913986364708e+000" norm="1.000000"/></NormContinuous></DerivedField></NeuralInput><NeuralInput id="84"><DerivedField><NormContinuous field="MLFER_S" shift="-4.31729518855657e-002" scale="2.60078023407022e-001"><LinearNorm orig="1.66000000000000e-001" norm="0.000000"/><LinearNorm orig="4.01100000000000e+000" norm="1.000000"/></NormContinuous></DerivedField></NeuralInput><NeuralInput id="85"><DerivedField><NormContinuous field="MLFER_E" shift="-4.75154153064926e-002" scale="3.62713093942691e-001"><LinearNorm orig="1.31000000000000e-001" norm="0.000000"/><LinearNorm orig="2.88800000000000e+000" norm="1.000000"/></NormContinuous></DerivedField></NeuralInput><NeuralInput id="86"><DerivedField><NormContinuous field="piPC4" shift="-0.00000000000000e+000" scale="1.72587682692228e-001"><LinearNorm orig="0.00000000000000e+000" norm="0.000000"/><LinearNorm orig="5.79415624800570e+000" norm="1.000000"/></NormContinuous></DerivedField></NeuralInput><NeuralInput id="87"><DerivedField><NormContinuous field="piPC5" shift="-0.00000000000000e+000" scale="1.52491223760283e-001"><LinearNorm orig="0.00000000000000e+000" norm="0.000000"/><LinearNorm orig="6.55775444213109e+000" norm="1.000000"/></NormContinuous></DerivedField></NeuralInput><NeuralInput id="88"><DerivedField><NormContinuous field="piPC6" shift="-0.00000000000000e+000" scale="1.41410715437847e-001"><LinearNorm orig="0.00000000000000e+000" norm="0.000000"/><LinearNorm orig="7.07159989187325e+000" norm="1.000000"/></NormContinuous></DerivedField></NeuralInput><NeuralInput id="89"><DerivedField><NormContinuous field="piPC8" shift="-0.00000000000000e+000" scale="1.21884254101035e-001"><LinearNorm orig="0.00000000000000e+000" norm="0.000000"/><LinearNorm orig="8.20450522814091e+000" norm="1.000000"/></NormContinuous></DerivedField></NeuralInput><NeuralInput id="90"><DerivedField><NormContinuous field="piPC9" shift="-0.00000000000000e+000" scale="1.13982133938273e-001"><LinearNorm orig="0.00000000000000e+000" norm="0.000000"/><LinearNorm orig="8.77330477547955e+000" norm="1.000000"/></NormContinuous></DerivedField></NeuralInput><NeuralInput id="91"><DerivedField><NormContinuous field="piPC10" shift="-0.00000000000000e+000" scale="1.10639339150100e-001"><LinearNorm orig="0.00000000000000e+000" norm="0.000000"/><LinearNorm orig="9.03837647333864e+000" norm="1.000000"/></NormContinuous></DerivedField></NeuralInput><NeuralInput id="92"><DerivedField><NormContinuous field="n6Ring" shift="-0.00000000000000e+000" scale="2.50000000000000e-001"><LinearNorm orig="0.00000000000000e+000" norm="0.000000"/><LinearNorm orig="4.00000000000000e+000" norm="1.000000"/></NormContinuous></DerivedField></NeuralInput><NeuralInput id="93"><DerivedField><NormContinuous field="RotBFrac" shift="-0.00000000000000e+000" scale="1.11111111111111e+000"><LinearNorm orig="0.00000000000000e+000" norm="0.000000"/><LinearNorm orig="9.00000000000000e-001" norm="1.000000"/></NormContinuous></DerivedField></NeuralInput><NeuralInput id="94"><DerivedField><NormContinuous field="RotBtFrac" shift="-0.00000000000000e+000" scale="1.00000000000000e+000"><LinearNorm orig="0.00000000000000e+000" norm="0.000000"/><LinearNorm orig="1.00000000000000e+000" norm="1.000000"/></NormContinuous></DerivedField></NeuralInput><NeuralInput id="95"><DerivedField><NormContinuous field="LipinskiFailures" shift="-0.00000000000000e+000" scale="5.00000000000000e-001"><LinearNorm orig="0.00000000000000e+000" norm="0.000000"/><LinearNorm orig="2.00000000000000e+000" norm="1.000000"/></NormContinuous></DerivedField></NeuralInput><NeuralInput id="96"><DerivedField><NormContinuous field="GGI6" shift="-0.00000000000000e+000" scale="8.73682542198275e-001"><LinearNorm orig="0.00000000000000e+000" norm="0.000000"/><LinearNorm orig="1.14458049886621e+000" norm="1.000000"/></NormContinuous></DerivedField></NeuralInput><NeuralInput id="97"><DerivedField><NormContinuous field="GGI8" shift="-0.00000000000000e+000" scale="2.19889196675901e+000"><LinearNorm orig="0.00000000000000e+000" norm="0.000000"/><LinearNorm orig="4.54774502393549e-001" norm="1.000000"/></NormContinuous></DerivedField></NeuralInput><NeuralInput id="98"><DerivedField><NormContinuous field="JGT" shift="-8.89538164333661e-002" scale="1.17561796099381e+000"><LinearNorm orig="7.56655813238589e-002" norm="0.000000"/><LinearNorm orig="9.26282051282051e-001" norm="1.000000"/></NormContinuous></DerivedField></NeuralInput><NeuralInput id="99"><DerivedField><NormContinuous field="WTPT-5" shift="-0.00000000000000e+000" scale="5.13831627777234e-002"><LinearNorm orig="0.00000000000000e+000" norm="0.000000"/><LinearNorm orig="1.94616280108304e+001" norm="1.000000"/></NormContinuous></DerivedField></NeuralInput></NeuralInputs><NeuralLayer numberOfNeurons="23" activationFunction="exponential"><Neuron id="100" bias="-1.69847990002530e-001"><Con from="0" weight="-4.93683052372377e-001"/><Con from="1" weight="2.59493695108294e-001"/><Con from="2" weight="-7.75091596505783e-001"/><Con from="3" weight="-3.81146216280091e-001"/><Con from="4" weight="2.88784057208004e-001"/><Con from="5" weight="-9.42743160347462e-002"/><Con from="6" weight="1.16991148989553e-001"/><Con from="7" weight="-3.64994111826390e-001"/><Con from="8" weight="-2.02861146519564e-001"/><Con from="9" weight="3.57170713531251e-003"/><Con from="10" weight="6.06958704984985e-002"/><Con from="11" weight="-3.86786136794281e-001"/><Con from="12" weight="-1.01367359185262e+000"/><Con from="13" weight="-6.73609563662292e-001"/><Con from="14" weight="9.83591024359651e-001"/><Con from="15" weight="-2.00249329390637e-001"/><Con from="16" weight="4.49757420683036e-003"/><Con from="17" weight="-2.14275342410790e-002"/><Con from="18" weight="5.83308782605098e-001"/><Con from="19" weight="5.60304905636578e-001"/><Con from="20" weight="-3.97246438414985e-001"/><Con from="21" weight="2.97574323544166e-001"/><Con from="22" weight="1.38666170144259e-001"/><Con from="23" weight="-2.11376691416147e-001"/><Con from="24" weight="-3.13471603485333e-001"/><Con from="25" weight="9.17849564212212e-002"/><Con from="26" weight="-5.30911821187872e-001"/><Con from="27" weight="-4.37849894866415e-001"/><Con from="28" weight="9.49547866571895e-001"/><Con from="29" weight="-8.83661821156635e-001"/><Con from="30" weight="-1.98151322678365e-001"/><Con from="31" weight="-6.10248910639283e-001"/><Con from="32" weight="-2.06737017908202e-001"/><Con from="33" weight="-9.02134275240779e-001"/><Con from="34" weight="-3.93124686792913e-001"/><Con from="35" weight="-7.71274497011163e-002"/><Con from="36" weight="-5.22769464292384e-001"/><Con from="37" weight="5.49887271506873e-001"/><Con from="38" weight="-1.15576773698866e-001"/><Con from="39" weight="2.15373336431637e-001"/><Con from="40" weight="7.85799389800400e-001"/><Con from="41" weight="8.28571423434006e-002"/><Con from="42" weight="-4.99211596535982e-001"/><Con from="43" weight="-3.13593815694026e-001"/><Con from="44" weight="3.15859221394071e-001"/><Con from="45" weight="-2.24711819176966e-001"/><Con from="46" weight="1.00974872176746e-001"/><Con from="47" weight="-1.04968066582803e+000"/><Con from="48" weight="1.45852740884303e-001"/><Con from="49" weight="-3.82082089097495e-001"/><Con from="50" weight="2.53077344871476e-001"/><Con from="51" weight="-7.96730326213287e-001"/><Con from="52" weight="-1.26875667687384e-001"/><Con from="53" weight="7.24677810391320e-001"/><Con from="54" weight="-1.33782820663428e-001"/><Con from="55" weight="3.21013393172943e-001"/><Con from="56" weight="2.00231837804001e-001"/><Con from="57" weight="1.68425652647937e-001"/><Con from="58" weight="-1.08617836839714e-001"/><Con from="59" weight="-6.54849936017239e-002"/><Con from="60" weight="3.59358677498432e-001"/><Con from="61" weight="-7.65090435041485e-002"/><Con from="62" weight="-1.14261376151733e+000"/><Con from="63" weight="-5.08301585254075e-001"/><Con from="64" weight="-9.03138342689386e-001"/><Con from="65" weight="9.49286217108608e-002"/><Con from="66" weight="-1.01686179764190e+000"/><Con from="67" weight="2.27333446419143e-001"/><Con from="68" weight="-5.05106766361352e-001"/><Con from="69" weight="-9.80106137735890e-001"/><Con from="70" weight="-1.36158084769217e-001"/><Con from="71" weight="4.47737200237128e-001"/><Con from="72" weight="9.12247306452677e-002"/><Con from="73" weight="-5.13326938167869e-002"/><Con from="74" weight="-6.54665573050146e-002"/><Con from="75" weight="1.91091035194145e-001"/><Con from="76" weight="-1.02349109974403e+000"/><Con from="77" weight="5.37622515070127e-001"/><Con from="78" weight="3.44643558219322e-001"/><Con from="79" weight="-3.43899542053917e-001"/><Con from="80" weight="-2.51357485450977e-001"/><Con from="81" weight="-3.10615253184703e-001"/><Con from="82" weight="-3.24799790825961e-001"/><Con from="83" weight="1.63202633501886e-001"/><Con from="84" weight="-5.02630591943694e-001"/><Con from="85" weight="-8.97136787089501e-001"/><Con from="86" weight="-4.38345023327212e-001"/><Con from="87" weight="9.69903203060374e-002"/><Con from="88" weight="-3.44764271985435e-003"/><Con from="89" weight="3.14984578880917e-001"/><Con from="90" weight="-7.82233531704425e-002"/><Con from="91" weight="-1.65060844917528e-001"/><Con from="92" weight="3.72020451181609e-001"/><Con from="93" weight="-8.00883666963981e-001"/><Con from="94" weight="-1.31897784674903e+000"/><Con from="95" weight="-1.19771330031628e+000"/><Con from="96" weight="-2.27968365187360e-001"/><Con from="97" weight="3.55656601099544e-002"/><Con from="98" weight="3.45877068941969e-002"/><Con from="99" weight="-5.01532564458675e-001"/></Neuron><Neuron id="101" bias="-2.45502751357756e-001"><Con from="0" weight="6.43247432970538e-002"/><Con from="1" weight="-3.10566222985976e-001"/><Con from="2" weight="-1.55159283685158e-001"/><Con from="3" weight="5.06539766139034e-001"/><Con from="4" weight="4.06961392244871e-001"/><Con from="5" weight="-5.73952841658752e-003"/><Con from="6" weight="4.07106655552075e-001"/><Con from="7" weight="-1.13408157060710e-001"/><Con from="8" weight="-4.97558176572767e-001"/><Con from="9" weight="-4.84583237504862e-001"/><Con from="10" weight="-3.81742414988131e-002"/><Con from="11" weight="1.32001445375242e-001"/><Con from="12" weight="-4.66180080979001e-001"/><Con from="13" weight="1.31864766240526e-001"/><Con from="14" weight="-7.56569046531807e-001"/><Con from="15" weight="1.93045301644617e-001"/><Con from="16" weight="5.02322619671932e-001"/><Con from="17" weight="4.36562008997379e-001"/><Con from="18" weight="5.13167497871364e-001"/><Con from="19" weight="-1.76219214841340e-001"/><Con from="20" weight="7.94632167064914e-001"/><Con from="21" weight="-5.47502830760180e-001"/><Con from="22" weight="2.06793283404388e-001"/><Con from="23" weight="-2.20569747724953e-001"/><Con from="24" weight="-3.91211233584272e-001"/><Con from="25" weight="-1.72262170261106e-001"/><Con from="26" weight="5.36853427264329e-001"/><Con from="27" weight="3.10986045822108e-002"/><Con from="28" weight="1.68036239344386e-001"/><Con from="29" weight="2.90804147068269e-001"/><Con from="30" weight="1.50698988519437e-001"/><Con from="31" weight="2.15861168184047e-001"/><Con from="32" weight="-8.13666736710715e-001"/><Con from="33" weight="-1.90576236085632e-001"/><Con from="34" weight="3.39138026671130e-001"/><Con from="35" weight="6.84618287600662e-001"/><Con from="36" weight="-3.08973072081234e-001"/><Con from="37" weight="3.14281163942738e-001"/><Con from="38" weight="5.84771160377687e-002"/><Con from="39" weight="4.36710546571556e-001"/><Con from="40" weight="6.08739915803272e-002"/><Con from="41" weight="5.49404790713224e-001"/><Con from="42" weight="9.56701736609005e-002"/><Con from="43" weight="3.25039936298085e-001"/><Con from="44" weight="-7.11834963021449e-001"/><Con from="45" weight="-4.11646101044018e-001"/><Con from="46" weight="1.51916810562129e-001"/><Con from="47" weight="4.71981314190732e-001"/><Con from="48" weight="2.69926292466144e-001"/><Con from="49" weight="6.31113342476428e-001"/><Con from="50" weight="7.19449570876999e-001"/><Con from="51" weight="1.88839692983604e-001"/><Con from="52" weight="8.47883876823922e-002"/><Con from="53" weight="5.16747225066628e-001"/><Con from="54" weight="7.59446991542407e-002"/><Con from="55" weight="4.91030242530478e-001"/><Con from="56" weight="-3.35778724796464e-001"/><Con from="57" weight="1.94875065272369e-001"/><Con from="58" weight="-1.53496096696652e-002"/><Con from="59" weight="-1.32612634884314e+000"/><Con from="60" weight="-7.77370949502339e-001"/><Con from="61" weight="-1.14075670397931e+000"/><Con from="62" weight="-2.78942234552358e-001"/><Con from="63" weight="-6.47034236377454e-001"/><Con from="64" weight="6.53050884715339e-001"/><Con from="65" weight="1.53068164373110e-001"/><Con from="66" weight="4.45963516150146e-001"/><Con from="67" weight="2.59118566780059e-001"/><Con from="68" weight="6.42258673168390e-001"/><Con from="69" weight="-2.12971629346320e-001"/><Con from="70" weight="-2.20456357575861e-001"/><Con from="71" weight="3.58064563913740e-002"/><Con from="72" weight="-3.97364783536693e-001"/><Con from="73" weight="7.42816227994238e-002"/><Con from="74" weight="-6.35307546707738e-001"/><Con from="75" weight="-7.15942650283012e-001"/><Con from="76" weight="-2.36693548848078e-001"/><Con from="77" weight="-4.88629002759004e-001"/><Con from="78" weight="1.03976484181743e-001"/><Con from="79" weight="-2.58753753624304e-001"/><Con from="80" weight="-2.43534424006365e-001"/><Con from="81" weight="-1.44250183993275e-001"/><Con from="82" weight="1.57552851701996e-001"/><Con from="83" weight="-4.07919723100969e-001"/><Con from="84" weight="-4.24960521353928e-001"/><Con from="85" weight="-3.83929079195134e-001"/><Con from="86" weight="-7.98251033001747e-001"/><Con from="87" weight="-2.11400291125268e-001"/><Con from="88" weight="1.72583160884989e-001"/><Con from="89" weight="1.85007799461214e-001"/><Con from="90" weight="-2.47101740914580e-001"/><Con from="91" weight="-5.01065185654392e-001"/><Con from="92" weight="-4.17996692006138e-001"/><Con from="93" weight="3.22258963597218e-001"/><Con from="94" weight="3.58002940584037e-001"/><Con from="95" weight="-2.27625906634359e-001"/><Con from="96" weight="3.49756848776313e-001"/><Con from="97" weight="6.83825362632739e-002"/><Con from="98" weight="-7.05009989619413e-001"/><Con from="99" weight="7.76684642919773e-002"/></Neuron><Neuron id="102" bias="-1.32136998011024e+000"><Con from="0" weight="-6.05886149432908e-002"/><Con from="1" weight="2.96894713523120e-001"/><Con from="2" weight="-3.73172344539003e-001"/><Con from="3" weight="3.94080571442551e-001"/><Con from="4" weight="-2.66917931605741e-001"/><Con from="5" weight="-6.26527156125798e-001"/><Con from="6" weight="3.68092774588462e-001"/><Con from="7" weight="5.57053853452452e-001"/><Con from="8" weight="5.07725306694630e-002"/><Con from="9" weight="-1.24842420098215e+000"/><Con from="10" weight="-1.20539940615860e+000"/><Con from="11" weight="-3.45064507071670e-001"/><Con from="12" weight="-4.57471123487873e-001"/><Con from="13" weight="-1.12020724738020e-001"/><Con from="14" weight="-1.02734595112455e+000"/><Con from="15" weight="-1.47149116689401e-001"/><Con from="16" weight="5.63793630205577e-001"/><Con from="17" weight="-4.29536359165624e-001"/><Con from="18" weight="-5.33349914234311e-001"/><Con from="19" weight="-1.41425042610326e+000"/><Con from="20" weight="1.47161749373049e+000"/><Con from="21" weight="-1.06669102792904e+000"/><Con from="22" weight="-4.55004376658729e-001"/><Con from="23" weight="2.95033944267283e-001"/><Con from="24" weight="1.80597534602898e-001"/><Con from="25" weight="2.22997354280941e-001"/><Con from="26" weight="6.09287669040711e-001"/><Con from="27" weight="5.12924360612659e-001"/><Con from="28" weight="-7.29392173905813e-001"/><Con from="29" weight="8.08999421428524e-001"/><Con from="30" weight="-3.62894153613289e-001"/><Con from="31" weight="2.71421192296628e-001"/><Con from="32" weight="4.21515306622538e-001"/><Con from="33" weight="8.12433691343609e-003"/><Con from="34" weight="-2.52305470835805e-001"/><Con from="35" weight="-1.65867117236073e-001"/><Con from="36" weight="-4.29994665498780e-001"/><Con from="37" weight="1.69898513750955e-001"/><Con from="38" weight="8.51373459402116e-004"/><Con from="39" weight="-5.30117808830148e-001"/><Con from="40" weight="8.92018255329245e-001"/><Con from="41" weight="-2.16048679046882e-001"/><Con from="42" weight="-7.94977242892319e-002"/><Con from="43" weight="8.59840631199604e-001"/><Con from="44" weight="-1.61692824157969e-002"/><Con from="45" weight="-8.58095452743764e-001"/><Con from="46" weight="1.08092777030669e-001"/><Con from="47" weight="-1.45613386611051e+000"/><Con from="48" weight="5.10210359636869e-001"/><Con from="49" weight="-5.60313428686742e-001"/><Con from="50" weight="-5.69801370349883e-001"/><Con from="51" weight="5.72347851026669e-001"/><Con from="52" weight="-2.12397937974061e-001"/><Con from="53" weight="-4.16619964209984e-001"/><Con from="54" weight="-7.69618539112188e-002"/><Con from="55" weight="-8.95785966842464e-001"/><Con from="56" weight="1.00768014589710e+000"/><Con from="57" weight="-4.69780443966110e-001"/><Con from="58" weight="-4.14113491165538e-001"/><Con from="59" weight="4.00136293586877e-001"/><Con from="60" weight="1.27425988728439e-001"/><Con from="61" weight="9.35095534773265e-001"/><Con from="62" weight="3.95938833598592e-001"/><Con from="63" weight="5.28396967911160e-002"/><Con from="64" weight="5.27964664042307e-001"/><Con from="65" weight="1.34455880183299e-001"/><Con from="66" weight="-1.66880229623347e+000"/><Con from="67" weight="3.77425334072756e-001"/><Con from="68" weight="-1.25220480069974e-001"/><Con from="69" weight="1.17281599512933e+000"/><Con from="70" weight="-5.30779574498801e-001"/><Con from="71" weight="7.38853874545780e-001"/><Con from="72" weight="-6.92420024197090e-001"/><Con from="73" weight="2.03166707084030e-001"/><Con from="74" weight="-3.96952759971083e-001"/><Con from="75" weight="-8.72340036181314e-001"/><Con from="76" weight="-1.45217390043173e-001"/><Con from="77" weight="-1.28363650781626e+000"/><Con from="78" weight="-1.13002929672070e-001"/><Con from="79" weight="9.10884105429417e-001"/><Con from="80" weight="6.07201396093007e-001"/><Con from="81" weight="6.78715938104667e-001"/><Con from="82" weight="-7.08230523038565e-001"/><Con from="83" weight="5.81757258739097e-001"/><Con from="84" weight="-2.96632133770511e-001"/><Con from="85" weight="-4.93507281791315e-001"/><Con from="86" weight="3.28796129978761e-001"/><Con from="87" weight="2.84410436520825e-001"/><Con from="88" weight="2.62941257249194e-001"/><Con from="89" weight="-7.09181146801345e-001"/><Con from="90" weight="-7.73860046505376e-002"/><Con from="91" weight="7.03279958529254e-001"/><Con from="92" weight="-2.11492758641171e-001"/><Con from="93" weight="-5.79724319804179e-002"/><Con from="94" weight="2.45179774646941e-001"/><Con from="95" weight="-1.28298160619269e+000"/><Con from="96" weight="-7.82581476917244e-001"/><Con from="97" weight="-5.04542457914654e-001"/><Con from="98" weight="-2.88521202163532e-001"/><Con from="99" weight="2.55682925477819e-002"/></Neuron><Neuron id="103" bias="-3.34042426287947e-002"><Con from="0" weight="-5.28737512896728e-001"/><Con from="1" weight="-1.14167978405503e+000"/><Con from="2" weight="7.77328369641225e-002"/><Con from="3" weight="1.35290139747321e-001"/><Con from="4" weight="-1.84076420767128e-002"/><Con from="5" weight="-2.86742593964111e-001"/><Con from="6" weight="-2.54131909657168e-001"/><Con from="7" weight="7.11546899688003e-001"/><Con from="8" weight="-4.93577082233397e-001"/><Con from="9" weight="-3.46903649098146e-001"/><Con from="10" weight="2.36386056359366e-001"/><Con from="11" weight="2.99042520322813e-001"/><Con from="12" weight="-7.64308222937399e-001"/><Con from="13" weight="-7.60705599875864e-001"/><Con from="14" weight="2.44697241526250e-002"/><Con from="15" weight="-3.30914547528973e-001"/><Con from="16" weight="-5.66223880108525e-002"/><Con from="17" weight="-1.13635023623294e-001"/><Con from="18" weight="1.93561039176037e-001"/><Con from="19" weight="1.01586550627377e-001"/><Con from="20" weight="-3.20845330898214e-001"/><Con from="21" weight="-1.92222797934267e-001"/><Con from="22" weight="7.36338734321050e-001"/><Con from="23" weight="5.31809181647732e-001"/><Con from="24" weight="-6.12970009702931e-001"/><Con from="25" weight="-2.44482541793871e-001"/><Con from="26" weight="4.95970458323975e-002"/><Con from="27" weight="-1.27872463421334e-001"/><Con from="28" weight="-3.50151774594361e-001"/><Con from="29" weight="-1.83143419279898e-001"/><Con from="30" weight="3.26713438670056e-001"/><Con from="31" weight="-2.86052227974140e-001"/><Con from="32" weight="-1.12266202152832e-001"/><Con from="33" weight="-1.51186065336818e-001"/><Con from="34" weight="-8.45144317122077e-002"/><Con from="35" weight="2.34826037549292e-001"/><Con from="36" weight="-9.35368104443185e-001"/><Con from="37" weight="1.21625480388879e-001"/><Con from="38" weight="-3.85847051648989e-001"/><Con from="39" weight="1.38890108740034e-001"/><Con from="40" weight="7.84068061511383e-001"/><Con from="41" weight="9.11526455173420e-001"/><Con from="42" weight="-7.93175674088761e-001"/><Con from="43" weight="-1.70108711279185e-001"/><Con from="44" weight="-9.30980619346495e-001"/><Con from="45" weight="-5.79355796372952e-001"/><Con from="46" weight="-5.63421237671174e-001"/><Con from="47" weight="-5.51577994446144e-001"/><Con from="48" weight="7.98062381967607e-001"/><Con from="49" weight="5.50250383864939e-001"/><Con from="50" weight="2.31828517893924e-001"/><Con from="51" weight="5.96991953363876e-001"/><Con from="52" weight="-9.49260162151131e-001"/><Con from="53" weight="2.37890742173788e-001"/><Con from="54" weight="-3.78742131103080e-001"/><Con from="55" weight="2.21124524647151e-001"/><Con from="56" weight="8.18645339161087e-001"/><Con from="57" weight="7.74134784357864e-001"/><Con from="58" weight="-6.96990351540668e-001"/><Con from="59" weight="7.51245199518115e-001"/><Con from="60" weight="-9.84136450230433e-001"/><Con from="61" weight="2.54308852851328e-002"/><Con from="62" weight="-5.21174449885569e-001"/><Con from="63" weight="-9.13499178390602e-001"/><Con from="64" weight="3.76151170598589e-001"/><Con from="65" weight="-5.70789536757335e-001"/><Con from="66" weight="-5.43406409925753e-001"/><Con from="67" weight="8.34341920396779e-001"/><Con from="68" weight="5.66625709048834e-001"/><Con from="69" weight="-7.11386212069305e-001"/><Con from="70" weight="5.20442106050646e-001"/><Con from="71" weight="4.52821372143176e-001"/><Con from="72" weight="3.56001717817580e-002"/><Con from="73" weight="5.49683670479652e-001"/><Con from="74" weight="-7.28312834475713e-001"/><Con from="75" weight="-6.52668823012121e-001"/><Con from="76" weight="-6.90294098302416e-001"/><Con from="77" weight="-1.11303455584172e+000"/><Con from="78" weight="1.93867873382861e-004"/><Con from="79" weight="-3.19877692825822e-001"/><Con from="80" weight="-2.06045424847036e-001"/><Con from="81" weight="-4.25643287271681e-001"/><Con from="82" weight="5.20548189306375e-001"/><Con from="83" weight="2.17263908799480e-001"/><Con from="84" weight="-4.92029056119855e-001"/><Con from="85" weight="-6.90939905270237e-001"/><Con from="86" weight="-5.25637782496241e-001"/><Con from="87" weight="-8.11473199833306e-001"/><Con from="88" weight="-7.65376970144121e-001"/><Con from="89" weight="-4.38023066542382e-001"/><Con from="90" weight="-3.47761888862684e-001"/><Con from="91" weight="-2.02890680606791e-001"/><Con from="92" weight="-5.46759523165037e-001"/><Con from="93" weight="3.61214141303409e-001"/><Con from="94" weight="6.84797329449423e-001"/><Con from="95" weight="-2.68253432284154e-001"/><Con from="96" weight="-1.59692725779570e-001"/><Con from="97" weight="2.50858350969099e-001"/><Con from="98" weight="5.39356568012025e-001"/><Con from="99" weight="-4.50118621284315e-001"/></Neuron><Neuron id="104" bias="3.41714717566671e-001"><Con from="0" weight="1.02666608561035e-001"/><Con from="1" weight="4.96373035599548e-001"/><Con from="2" weight="-3.89897783721824e-001"/><Con from="3" weight="-1.81288979243162e-002"/><Con from="4" weight="-5.54646615479156e-001"/><Con from="5" weight="1.86958289050943e+000"/><Con from="6" weight="-1.91025678711377e+000"/><Con from="7" weight="-1.45907035948413e-001"/><Con from="8" weight="-9.06806095397900e-001"/><Con from="9" weight="8.91918658749248e-001"/><Con from="10" weight="-3.13815588869218e-001"/><Con from="11" weight="-9.86983178893007e-002"/><Con from="12" weight="3.90902248162125e-001"/><Con from="13" weight="8.31944998233595e-001"/><Con from="14" weight="-5.26307334249539e-001"/><Con from="15" weight="1.30734323786406e+000"/><Con from="16" weight="-9.43414531253142e-001"/><Con from="17" weight="-8.14564325105127e-001"/><Con from="18" weight="8.79091765808898e-002"/><Con from="19" weight="7.51837867556330e-001"/><Con from="20" weight="9.77650552099913e-001"/><Con from="21" weight="-8.16240813397367e-001"/><Con from="22" weight="1.54450757682462e-001"/><Con from="23" weight="3.88118526611774e-001"/><Con from="24" weight="-5.03812689020135e-001"/><Con from="25" weight="-7.30502982840850e-001"/><Con from="26" weight="8.49296346149860e-001"/><Con from="27" weight="-4.17406960058869e-001"/><Con from="28" weight="6.31237394051808e-001"/><Con from="29" weight="1.09112512931824e-001"/><Con from="30" weight="7.89333934925676e-001"/><Con from="31" weight="-9.06784698403572e-001"/><Con from="32" weight="9.38040234904217e-001"/><Con from="33" weight="-6.56391392628936e-001"/><Con from="34" weight="-5.24037571954165e-001"/><Con from="35" weight="-1.34442021518098e+000"/><Con from="36" weight="-1.66604050805529e-001"/><Con from="37" weight="-9.05642186344266e-001"/><Con from="38" weight="-4.18794283481527e-001"/><Con from="39" weight="-1.04456662180157e+000"/><Con from="40" weight="5.65278036029656e-001"/><Con from="41" weight="-6.82891267104048e-001"/><Con from="42" weight="6.95920265492964e-002"/><Con from="43" weight="-7.33031507346804e-001"/><Con from="44" weight="3.33570629982278e-001"/><Con from="45" weight="-6.51762289691446e-001"/><Con from="46" weight="4.20533910303653e-001"/><Con from="47" weight="-1.58456413834371e+000"/><Con from="48" weight="1.72460865560464e-001"/><Con from="49" weight="8.91990931303869e-002"/><Con from="50" weight="-5.40307544104274e-001"/><Con from="51" weight="-9.90325505268201e-002"/><Con from="52" weight="2.48618383107804e-001"/><Con from="53" weight="4.96110920294085e-001"/><Con from="54" weight="-2.60755502547103e-001"/><Con from="55" weight="-7.75064620456487e-001"/><Con from="56" weight="7.59768740286227e-001"/><Con from="57" weight="-3.14214798140691e-001"/><Con from="58" weight="5.51930373663620e-001"/><Con from="59" weight="4.08992476617639e-001"/><Con from="60" weight="3.65908972842642e-001"/><Con from="61" weight="1.02890549088109e+000"/><Con from="62" weight="-1.63965299318585e-001"/><Con from="63" weight="-5.28903536455961e-001"/><Con from="64" weight="7.76542088976830e-001"/><Con from="65" weight="3.93511391967519e-001"/><Con from="66" weight="-9.38306693713832e-001"/><Con from="67" weight="9.02479980907923e-001"/><Con from="68" weight="-1.01864681055423e+000"/><Con from="69" weight="-4.54615154273438e-001"/><Con from="70" weight="2.42593580756023e-001"/><Con from="71" weight="-6.13368124958589e-001"/><Con from="72" weight="1.36780819629654e+000"/><Con from="73" weight="-6.57625885902264e-001"/><Con from="74" weight="-1.75771320335948e+000"/><Con from="75" weight="-9.13137430963381e-001"/><Con from="76" weight="-1.82316003021299e+000"/><Con from="77" weight="1.37592059471761e+000"/><Con from="78" weight="1.87507172360026e-001"/><Con from="79" weight="-4.77718918468778e-001"/><Con from="80" weight="2.74241855609455e-001"/><Con from="81" weight="-1.27918731081352e+000"/><Con from="82" weight="2.30095544776968e+000"/><Con from="83" weight="-2.03222716228971e+000"/><Con from="84" weight="1.13943433234560e+000"/><Con from="85" weight="-2.24183842472010e-001"/><Con from="86" weight="6.25203364106878e-001"/><Con from="87" weight="2.79742560835679e-002"/><Con from="88" weight="-5.78123299609735e-001"/><Con from="89" weight="7.00582475746165e-001"/><Con from="90" weight="-1.37058649385213e-001"/><Con from="91" weight="-5.03624968890211e-001"/><Con from="92" weight="1.05125815063738e+000"/><Con from="93" weight="9.62114906749562e-001"/><Con from="94" weight="-1.05415769591938e-001"/><Con from="95" weight="-1.01741882780394e+000"/><Con from="96" weight="8.13310382257416e-001"/><Con from="97" weight="-6.94419155940637e-001"/><Con from="98" weight="3.91619166257386e-001"/><Con from="99" weight="4.67539119304291e-001"/></Neuron><Neuron id="105" bias="1.68117814356826e-001"><Con from="0" weight="1.94646692695792e-001"/><Con from="1" weight="1.92987458148265e-001"/><Con from="2" weight="3.82324750523847e-001"/><Con from="3" weight="-8.73569098581521e-001"/><Con from="4" weight="-4.81250069822418e-001"/><Con from="5" weight="-7.56008703968534e-001"/><Con from="6" weight="4.60447662476103e-002"/><Con from="7" weight="1.01436002961255e-001"/><Con from="8" weight="-5.43214554724137e-002"/><Con from="9" weight="-1.09404082332438e-001"/><Con from="10" weight="-1.55394539821077e-001"/><Con from="11" weight="5.45250918082565e-001"/><Con from="12" weight="8.64017505795342e-001"/><Con from="13" weight="-3.57436593648998e-001"/><Con from="14" weight="-3.77277584296986e-001"/><Con from="15" weight="1.75410820831256e-001"/><Con from="16" weight="1.36009642529253e-001"/><Con from="17" weight="-9.38785192892529e-001"/><Con from="18" weight="3.82387216553547e-001"/><Con from="19" weight="-2.76246087546290e-001"/><Con from="20" weight="-1.53235858992863e+000"/><Con from="21" weight="-3.49164626672255e-001"/><Con from="22" weight="-4.72672360223571e-001"/><Con from="23" weight="1.29970396789491e-001"/><Con from="24" weight="-1.01500169505933e-001"/><Con from="25" weight="-1.66472594007479e-002"/><Con from="26" weight="-9.30951886612174e-001"/><Con from="27" weight="1.31296763690753e-001"/><Con from="28" weight="5.29634371086090e-001"/><Con from="29" weight="2.35696127151688e-001"/><Con from="30" weight="-3.79325019382799e-001"/><Con from="31" weight="3.34855879241971e-001"/><Con from="32" weight="1.36433344922594e+000"/><Con from="33" weight="-4.90150356046957e-001"/><Con from="34" weight="1.55928003035342e-001"/><Con from="35" weight="-6.57600334972199e-001"/><Con from="36" weight="-7.64319157743775e-001"/><Con from="37" weight="2.13567152669217e-001"/><Con from="38" weight="5.09481196208945e-001"/><Con from="39" weight="6.75228301504851e-001"/><Con from="40" weight="-3.10056666250576e-001"/><Con from="41" weight="-7.77776474145331e-001"/><Con from="42" weight="-4.20219066347517e-001"/><Con from="43" weight="1.00979389415382e+000"/><Con from="44" weight="-1.79233908768891e-001"/><Con from="45" weight="4.55335446924759e-002"/><Con from="46" weight="5.24773691192421e-002"/><Con from="47" weight="5.25914395615384e-002"/><Con from="48" weight="9.17420800653065e-001"/><Con from="49" weight="2.59546031612981e-001"/><Con from="50" weight="-8.21815071533992e-001"/><Con from="51" weight="1.01287357263340e+000"/><Con from="52" weight="-4.99802794995195e-001"/><Con from="53" weight="-1.31748271328533e-001"/><Con from="54" weight="4.78656534815309e-001"/><Con from="55" weight="4.46560770384898e-001"/><Con from="56" weight="1.27700254799849e+000"/><Con from="57" weight="2.14058702364438e-001"/><Con from="58" weight="-5.31057978946580e-001"/><Con from="59" weight="9.10147536146800e-002"/><Con from="60" weight="-3.04269696341703e-001"/><Con from="61" weight="6.53381073885884e-001"/><Con from="62" weight="-9.45869446685994e-001"/><Con from="63" weight="-5.69714786505406e-002"/><Con from="64" weight="-3.63454306823224e-002"/><Con from="65" weight="3.59323315911408e-002"/><Con from="66" weight="-1.62520191430348e-002"/><Con from="67" weight="1.03387460075459e+000"/><Con from="68" weight="4.43337516333901e-001"/><Con from="69" weight="-6.82283339169888e-001"/><Con from="70" weight="5.76486529047135e-001"/><Con from="71" weight="-2.72675313916776e-001"/><Con from="72" weight="8.61611787396435e-001"/><Con from="73" weight="-2.49190483422794e-001"/><Con from="74" weight="-7.79166832605215e-001"/><Con from="75" weight="-1.03941107217212e+000"/><Con from="76" weight="-6.97092550559151e-001"/><Con from="77" weight="-1.05138737822500e+000"/><Con from="78" weight="4.46427704777796e-001"/><Con from="79" weight="-1.16002998731968e+000"/><Con from="80" weight="-1.14146887161154e+000"/><Con from="81" weight="-2.37078805228747e-001"/><Con from="82" weight="7.97926261591322e-001"/><Con from="83" weight="1.52464098721055e-001"/><Con from="84" weight="5.56409174849572e-001"/><Con from="85" weight="-4.11818117299106e-001"/><Con from="86" weight="-1.74225052194624e-001"/><Con from="87" weight="-8.90576034669536e-001"/><Con from="88" weight="-7.99323689016430e-001"/><Con from="89" weight="-9.39021432606261e-002"/><Con from="90" weight="6.48386283635685e-001"/><Con from="91" weight="6.50987726255635e-001"/><Con from="92" weight="1.34165647828852e-001"/><Con from="93" weight="1.20291263713332e+000"/><Con from="94" weight="7.57792648296548e-001"/><Con from="95" weight="2.31263253503710e-001"/><Con from="96" weight="-4.27477597184546e-001"/><Con from="97" weight="1.46026035027711e-002"/><Con from="98" weight="-2.81671544140413e-001"/><Con from="99" weight="1.42791499735301e-001"/></Neuron><Neuron id="106" bias="-2.60694312586898e-001"><Con from="0" weight="-2.36884291691504e-001"/><Con from="1" weight="6.07652798835884e-001"/><Con from="2" weight="-5.74323698086268e-001"/><Con from="3" weight="5.73426942558950e-001"/><Con from="4" weight="5.29568510442528e-002"/><Con from="5" weight="-4.52095218516896e-001"/><Con from="6" weight="7.87114100075749e-001"/><Con from="7" weight="-2.72087055013421e-001"/><Con from="8" weight="-3.18810789799859e-001"/><Con from="9" weight="-2.05906937226635e-001"/><Con from="10" weight="-2.53595569859077e-001"/><Con from="11" weight="-1.04890369673661e-001"/><Con from="12" weight="-3.68537599306534e-001"/><Con from="13" weight="-1.08348693830792e-001"/><Con from="14" weight="-6.00998723416018e-001"/><Con from="15" weight="-3.10910885329900e-001"/><Con from="16" weight="-2.85123762478109e-001"/><Con from="17" weight="1.77732635932294e+000"/><Con from="18" weight="2.93602097374590e-002"/><Con from="19" weight="-9.31591312362495e-001"/><Con from="20" weight="4.07919914078071e-001"/><Con from="21" weight="-7.83490814953657e-002"/><Con from="22" weight="7.86405930009674e-001"/><Con from="23" weight="9.41393753388226e-003"/><Con from="24" weight="-2.53154648538824e-001"/><Con from="25" weight="-1.32004064032086e-001"/><Con from="26" weight="2.29245197990208e-001"/><Con from="27" weight="-6.10128326516713e-001"/><Con from="28" weight="1.34199075477668e-002"/><Con from="29" weight="-8.71344699146504e-001"/><Con from="30" weight="5.42509187749833e-001"/><Con from="31" weight="6.07523360656290e-001"/><Con from="32" weight="3.53870606981775e-002"/><Con from="33" weight="-3.48934174124560e-001"/><Con from="34" weight="-4.74154352539209e-001"/><Con from="35" weight="-1.09284210368950e-001"/><Con from="36" weight="-5.36345214059121e-001"/><Con from="37" weight="-2.94528097640604e-001"/><Con from="38" weight="-9.06559690661522e-001"/><Con from="39" weight="3.18189473982362e-001"/><Con from="40" weight="9.12190388341624e-001"/><Con from="41" weight="4.55756412176507e-001"/><Con from="42" weight="-3.71843711539618e-001"/><Con from="43" weight="-1.20209047613457e+000"/><Con from="44" weight="4.82399110230333e-001"/><Con from="45" weight="7.27663759544014e-002"/><Con from="46" weight="1.37975523972340e-001"/><Con from="47" weight="2.57177565322488e-001"/><Con from="48" weight="3.91553099822819e-001"/><Con from="49" weight="-5.02501876292381e-002"/><Con from="50" weight="-2.25239153923066e-001"/><Con from="51" weight="3.50960188794785e-001"/><Con from="52" weight="-2.17109640013198e-001"/><Con from="53" weight="-7.52615365609325e-001"/><Con from="54" weight="-8.78146669882533e-001"/><Con from="55" weight="1.81356757895799e-001"/><Con from="56" weight="1.52346691253737e+000"/><Con from="57" weight="-9.95511808940347e-002"/><Con from="58" weight="-6.72768783289315e-001"/><Con from="59" weight="1.42938264771981e-001"/><Con from="60" weight="4.75209982419683e-001"/><Con from="61" weight="5.12446334823563e-002"/><Con from="62" weight="3.82615440100007e-001"/><Con from="63" weight="-1.00125364072629e-001"/><Con from="64" weight="7.25324566483277e-001"/><Con from="65" weight="1.09583378036804e-001"/><Con from="66" weight="7.13790776064631e-002"/><Con from="67" weight="4.68925091598320e-001"/><Con from="68" weight="3.05025269171950e-001"/><Con from="69" weight="-6.73732090927043e-001"/><Con from="70" weight="2.45816622276318e-001"/><Con from="71" weight="5.62801890193451e-001"/><Con from="72" weight="-4.07927317524836e-001"/><Con from="73" weight="3.33752424161847e-001"/><Con from="74" weight="-6.20035816259156e-001"/><Con from="75" weight="-7.21680841602748e-001"/><Con from="76" weight="-5.45906327607088e-001"/><Con from="77" weight="-8.67886907566062e-001"/><Con from="78" weight="8.10073746777381e-001"/><Con from="79" weight="-1.59925087262331e-001"/><Con from="80" weight="-3.23967994960786e-001"/><Con from="81" weight="7.29037840522706e-002"/><Con from="82" weight="9.61323084711319e-001"/><Con from="83" weight="1.61160599451317e-001"/><Con from="84" weight="-1.04782198804992e-002"/><Con from="85" weight="-8.56556187840936e-001"/><Con from="86" weight="-6.38900358448629e-001"/><Con from="87" weight="-3.95394225774333e-002"/><Con from="88" weight="3.97279783300116e-001"/><Con from="89" weight="2.07481372858475e-001"/><Con from="90" weight="-2.26217544666690e-001"/><Con from="91" weight="-6.45313402027984e-001"/><Con from="92" weight="-6.73024220594902e-001"/><Con from="93" weight="1.03503799565765e-001"/><Con from="94" weight="2.45552861306452e-001"/><Con from="95" weight="-3.06682682793801e-001"/><Con from="96" weight="4.22150590104153e-001"/><Con from="97" weight="3.29413021812756e-001"/><Con from="98" weight="-4.49351050920022e-001"/><Con from="99" weight="-2.91115948082916e-001"/></Neuron><Neuron id="107" bias="3.94127099815481e-001"><Con from="0" weight="-4.22964607975213e-001"/><Con from="1" weight="-6.34803371367642e-001"/><Con from="2" weight="-2.81775185424461e-001"/><Con from="3" weight="-3.58257579054769e-001"/><Con from="4" weight="-7.36620592960860e-003"/><Con from="5" weight="-1.06880985869168e+000"/><Con from="6" weight="-4.46246212443526e-001"/><Con from="7" weight="-1.65709355091796e-001"/><Con from="8" weight="3.32462007686083e-001"/><Con from="9" weight="3.59906050116890e-001"/><Con from="10" weight="-4.24380779489853e-001"/><Con from="11" weight="-3.49976018946285e-001"/><Con from="12" weight="-9.85538048693130e-001"/><Con from="13" weight="-9.70343339617808e-001"/><Con from="14" weight="-8.61804510782393e-001"/><Con from="15" weight="6.77359020076254e-001"/><Con from="16" weight="8.26513719502133e-002"/><Con from="17" weight="-7.98990174543073e-001"/><Con from="18" weight="-4.42502922785747e-001"/><Con from="19" weight="-1.66458891850210e+000"/><Con from="20" weight="-2.22342634858945e+000"/><Con from="21" weight="-1.25034373404012e+000"/><Con from="22" weight="-4.70962101555461e-001"/><Con from="23" weight="2.55170050475697e-001"/><Con from="24" weight="2.12992166260257e-001"/><Con from="25" weight="6.37168735000468e-001"/><Con from="26" weight="-1.36541042367951e-001"/><Con from="27" weight="7.53768435576615e-001"/><Con from="28" weight="2.16688125486953e-001"/><Con from="29" weight="-1.17432041181002e-002"/><Con from="30" weight="-2.41913608620731e-001"/><Con from="31" weight="4.02382710540255e-001"/><Con from="32" weight="-3.94696301568849e-001"/><Con from="33" weight="-2.51999336355408e-001"/><Con from="34" weight="7.98325927918145e-001"/><Con from="35" weight="-3.91870437119184e-001"/><Con from="36" weight="9.72336974663607e-001"/><Con from="37" weight="-1.03801253703511e+000"/><Con from="38" weight="4.24651405206984e-001"/><Con from="39" weight="2.25601818487551e-001"/><Con from="40" weight="-9.25837780691257e-001"/><Con from="41" weight="-3.61381465108351e-001"/><Con from="42" weight="-7.71924200393901e-001"/><Con from="43" weight="4.41165556271979e-001"/><Con from="44" weight="-7.58711800289942e-002"/><Con from="45" weight="1.93233484603573e-001"/><Con from="46" weight="3.97201895198170e-001"/><Con from="47" weight="6.65380524125755e-001"/><Con from="48" weight="1.51358695182827e+000"/><Con from="49" weight="-5.06185221444104e-001"/><Con from="50" weight="-6.83896673757388e-001"/><Con from="51" weight="4.43550687063503e-001"/><Con from="52" weight="-8.97174381021255e-001"/><Con from="53" weight="-5.84211390997985e-001"/><Con from="54" weight="3.89277563146447e-001"/><Con from="55" weight="1.52891019446701e-001"/><Con from="56" weight="-1.63189360375593e+000"/><Con from="57" weight="-6.90213226332047e-001"/><Con from="58" weight="-2.57283491512926e-001"/><Con from="59" weight="-1.26461935858910e+000"/><Con from="60" weight="1.03017673340498e+000"/><Con from="61" weight="-9.01670700466963e-001"/><Con from="62" weight="1.20823782487522e-002"/><Con from="63" weight="-7.30580424822142e-001"/><Con from="64" weight="-8.18638664814959e-001"/><Con from="65" weight="3.94360264272521e-001"/><Con from="66" weight="9.41797484898198e-002"/><Con from="67" weight="1.04794755208669e+000"/><Con from="68" weight="-2.66118060923763e-001"/><Con from="69" weight="1.74229036757480e-001"/><Con from="70" weight="1.06745825864831e+000"/><Con from="71" weight="8.17489663711758e-002"/><Con from="72" weight="-8.06857819567986e-001"/><Con from="73" weight="-6.72128409801377e-001"/><Con from="74" weight="4.57950912604753e-001"/><Con from="75" weight="3.12523498996925e-002"/><Con from="76" weight="-1.07718940583874e+000"/><Con from="77" weight="5.73715529237571e-001"/><Con from="78" weight="4.81838224669662e-001"/><Con from="79" weight="7.36450525149538e-001"/><Con from="80" weight="4.04325959987084e-001"/><Con from="81" weight="-8.61611072280011e-001"/><Con from="82" weight="-9.90862827406143e-001"/><Con from="83" weight="7.39240785195390e-001"/><Con from="84" weight="-1.09558592496723e+000"/><Con from="85" weight="4.28334570050504e-001"/><Con from="86" weight="5.22748526669943e-001"/><Con from="87" weight="9.46887170880653e-001"/><Con from="88" weight="1.11118043171383e+000"/><Con from="89" weight="-1.11837210318848e+000"/><Con from="90" weight="5.38280419675983e-002"/><Con from="91" weight="-1.61622954334401e-001"/><Con from="92" weight="9.81678974271951e-001"/><Con from="93" weight="-5.05563070255368e-001"/><Con from="94" weight="-1.35576815802283e-001"/><Con from="95" weight="6.98521270505141e-001"/><Con from="96" weight="7.02598523145500e-001"/><Con from="97" weight="-9.05973366815350e-001"/><Con from="98" weight="9.70994339609895e-002"/><Con from="99" weight="-3.51762324073311e-001"/></Neuron><Neuron id="108" bias="-1.66491922422418e+000"><Con from="0" weight="5.34164190792255e-001"/><Con from="1" weight="2.99266518347416e-001"/><Con from="2" weight="-2.12923141284748e-001"/><Con from="3" weight="-3.32847869496704e-002"/><Con from="4" weight="-2.90244028190619e-002"/><Con from="5" weight="-4.87678598692986e-001"/><Con from="6" weight="-1.16673515989422e+000"/><Con from="7" weight="4.26961597220830e-001"/><Con from="8" weight="-5.31815084187113e-001"/><Con from="9" weight="4.26921502578012e-001"/><Con from="10" weight="-2.34610163696122e-001"/><Con from="11" weight="-2.10515038088490e-001"/><Con from="12" weight="1.70635793752022e-001"/><Con from="13" weight="8.37697248839590e-001"/><Con from="14" weight="1.49183421477329e+000"/><Con from="15" weight="5.58426396191836e-002"/><Con from="16" weight="-1.52049710935039e+000"/><Con from="17" weight="-1.55131168657045e+000"/><Con from="18" weight="-4.18855377669093e-003"/><Con from="19" weight="5.64784475819826e-001"/><Con from="20" weight="2.15061806643687e-001"/><Con from="21" weight="-3.46132296224915e-001"/><Con from="22" weight="-1.19190755350654e+000"/><Con from="23" weight="-1.75037170657925e-001"/><Con from="24" weight="3.49599977328562e-001"/><Con from="25" weight="-4.45037956967145e-002"/><Con from="26" weight="-2.14089160507700e-001"/><Con from="27" weight="-3.81729432805112e-001"/><Con from="28" weight="-3.47907857490330e-001"/><Con from="29" weight="1.18014387744056e+000"/><Con from="30" weight="-2.98906077947488e-001"/><Con from="31" weight="-1.20442704069286e+000"/><Con from="32" weight="8.04438686950720e-001"/><Con from="33" weight="5.85632496207135e-001"/><Con from="34" weight="-8.45461128504108e-001"/><Con from="35" weight="-2.29719553048282e-001"/><Con from="36" weight="-1.12191542109022e+000"/><Con from="37" weight="-3.17381880009840e-001"/><Con from="38" weight="-1.36348775071368e-001"/><Con from="39" weight="-4.80950520354839e-001"/><Con from="40" weight="-7.43254736471071e-001"/><Con from="41" weight="-7.96063031297271e-001"/><Con from="42" weight="1.94700069889345e+000"/><Con from="43" weight="1.37018308173711e-001"/><Con from="44" weight="1.24232539183031e+000"/><Con from="45" weight="-5.82684006247600e-001"/><Con from="46" weight="1.72331619993693e-001"/><Con from="47" weight="-1.06381107909112e-001"/><Con from="48" weight="-2.75750872601403e-001"/><Con from="49" weight="-2.77893821967717e-001"/><Con from="50" weight="-6.91394101229515e-002"/><Con from="51" weight="-1.06952787975631e+000"/><Con from="52" weight="1.46636605990217e+000"/><Con from="53" weight="-5.26257401604810e-001"/><Con from="54" weight="3.68255285737748e-002"/><Con from="55" weight="-8.50154159546933e-002"/><Con from="56" weight="-1.00591643311094e+000"/><Con from="57" weight="-6.14048504608084e-002"/><Con from="58" weight="1.17275910129349e-002"/><Con from="59" weight="1.32615095645338e-001"/><Con from="60" weight="2.35901642395358e-001"/><Con from="61" weight="-1.07789999075575e-001"/><Con from="62" weight="1.41355044465299e+000"/><Con from="63" weight="1.87606506247788e-001"/><Con from="64" weight="1.87738941880827e-001"/><Con from="65" weight="1.21215591290224e-002"/><Con from="66" weight="6.42516068463300e-001"/><Con from="67" weight="1.04282991502668e+000"/><Con from="68" weight="-5.06912851584562e-001"/><Con from="69" weight="1.09117459829700e+000"/><Con from="70" weight="-6.54951390745358e-001"/><Con from="71" weight="4.14778049449786e-002"/><Con from="72" weight="1.72654578260464e-001"/><Con from="73" weight="-3.12148779736541e-001"/><Con from="74" weight="-6.79494792378397e-001"/><Con from="75" weight="2.89811146626215e-002"/><Con from="76" weight="-6.11809702689466e-001"/><Con from="77" weight="9.69792014552779e-001"/><Con from="78" weight="-1.00129651621395e+000"/><Con from="79" weight="7.92520736920535e-002"/><Con from="80" weight="8.00803603181051e-001"/><Con from="81" weight="-1.33623957836143e-001"/><Con from="82" weight="-1.34556578336710e-001"/><Con from="83" weight="-1.59953783000444e+000"/><Con from="84" weight="3.37662953812576e-001"/><Con from="85" weight="-1.57089927843455e-002"/><Con from="86" weight="2.92414369322542e-001"/><Con from="87" weight="-4.72257960743332e-001"/><Con from="88" weight="-4.55369504411675e-001"/><Con from="89" weight="2.84032385127169e-001"/><Con from="90" weight="1.44815211044826e-001"/><Con from="91" weight="-5.43031814758935e-001"/><Con from="92" weight="9.40377265734907e-001"/><Con from="93" weight="8.85239492736393e-001"/><Con from="94" weight="-9.17923086485103e-001"/><Con from="95" weight="-9.06474266634852e-001"/><Con from="96" weight="-4.43703765237378e-001"/><Con from="97" weight="-2.59972553422304e-001"/><Con from="98" weight="7.25212670502722e-001"/><Con from="99" weight="5.81611533182053e-001"/></Neuron><Neuron id="109" bias="-2.31418360584492e-001"><Con from="0" weight="-8.14563976888092e-001"/><Con from="1" weight="3.52845329724822e-001"/><Con from="2" weight="2.35240189941568e-001"/><Con from="3" weight="-3.17389430937364e-001"/><Con from="4" weight="-6.71121613937310e-001"/><Con from="5" weight="-4.29658190428287e-003"/><Con from="6" weight="-1.79780494586844e-001"/><Con from="7" weight="8.88661951656350e-001"/><Con from="8" weight="4.67273790083295e-001"/><Con from="9" weight="1.35732937317772e-001"/><Con from="10" weight="-1.13657789740066e+000"/><Con from="11" weight="-1.19828491570688e+000"/><Con from="12" weight="1.41336714654973e-001"/><Con from="13" weight="3.10609500832237e-001"/><Con from="14" weight="-3.53685078257951e-001"/><Con from="15" weight="9.40942788752518e-001"/><Con from="16" weight="-5.71884486561770e-001"/><Con from="17" weight="-1.84220848781733e+000"/><Con from="18" weight="-4.96973056353177e-001"/><Con from="19" weight="-5.75322899111209e-001"/><Con from="20" weight="-4.49743337230710e-001"/><Con from="21" weight="-2.62876922336773e-001"/><Con from="22" weight="-1.50363114014343e-001"/><Con from="23" weight="-4.23979428262690e-001"/><Con from="24" weight="3.40735487773907e-001"/><Con from="25" weight="-2.13185531243990e-001"/><Con from="26" weight="7.94494587344018e-001"/><Con from="27" weight="6.17042524492271e-001"/><Con from="28" weight="-5.12657425433594e-001"/><Con from="29" weight="4.40388233756575e-001"/><Con from="30" weight="-9.92787816154653e-001"/><Con from="31" weight="1.11911700899505e+000"/><Con from="32" weight="1.21139822607335e+000"/><Con from="33" weight="6.43494638661180e-002"/><Con from="34" weight="4.38763128743111e-001"/><Con from="35" weight="-7.23505138621437e-001"/><Con from="36" weight="1.42398326761354e+000"/><Con from="37" weight="4.01844717559986e-002"/><Con from="38" weight="-7.76214569247695e-001"/><Con from="39" weight="4.85087784710134e-001"/><Con from="40" weight="9.42456195709815e-001"/><Con from="41" weight="-2.07786194776150e-001"/><Con from="42" weight="5.88016999764503e-001"/><Con from="43" weight="5.56168873820947e-001"/><Con from="44" weight="4.52959403099377e-001"/><Con from="45" weight="4.94746659021899e-001"/><Con from="46" weight="-1.10004150672226e+000"/><Con from="47" weight="5.58495376639364e-001"/><Con from="48" weight="2.41881303033169e-001"/><Con from="49" weight="-1.74709643631699e-001"/><Con from="50" weight="-9.58457290210437e-001"/><Con from="51" weight="1.75407060187086e-002"/><Con from="52" weight="1.13440471275615e-003"/><Con from="53" weight="-4.43883502764361e-001"/><Con from="54" weight="-7.58015920406423e-001"/><Con from="55" weight="2.92332880930144e-001"/><Con from="56" weight="1.96169380176033e-001"/><Con from="57" weight="-5.09440323919891e-001"/><Con from="58" weight="3.00171743187045e-001"/><Con from="59" weight="-9.33074575445332e-001"/><Con from="60" weight="5.93840006248658e-001"/><Con from="61" weight="4.54228764388556e-001"/><Con from="62" weight="-3.17314509243901e-001"/><Con from="63" weight="8.07775796697406e-001"/><Con from="64" weight="-1.50664106478791e+000"/><Con from="65" weight="-1.06310209879502e+000"/><Con from="66" weight="3.85450788442343e-001"/><Con from="67" weight="3.92232204232850e-002"/><Con from="68" weight="-1.72595864227389e-001"/><Con from="69" weight="-1.31069548359604e-001"/><Con from="70" weight="-8.28469897639254e-001"/><Con from="71" weight="-4.13101526063850e-001"/><Con from="72" weight="-8.29434428752156e-001"/><Con from="73" weight="-1.74413803876051e-001"/><Con from="74" weight="2.40566396140202e-001"/><Con from="75" weight="7.29953561836494e-002"/><Con from="76" weight="1.33033367984658e-002"/><Con from="77" weight="-3.06622907802366e-001"/><Con from="78" weight="-4.23433316732620e-001"/><Con from="79" weight="8.17272481969120e-001"/><Con from="80" weight="5.58043410758376e-001"/><Con from="81" weight="2.27983911191254e-001"/><Con from="82" weight="-7.24560247925506e-001"/><Con from="83" weight="1.33946032777747e-002"/><Con from="84" weight="-2.18995596393658e-001"/><Con from="85" weight="2.37767915605381e-001"/><Con from="86" weight="-5.57936946701279e-001"/><Con from="87" weight="-4.96223864308194e-002"/><Con from="88" weight="-6.30248059612490e-001"/><Con from="89" weight="3.71501719781263e-001"/><Con from="90" weight="1.34744734930660e-001"/><Con from="91" weight="-4.85834273450484e-001"/><Con from="92" weight="2.69021581607807e-001"/><Con from="93" weight="-4.92514818872993e-001"/><Con from="94" weight="6.14122573554422e-002"/><Con from="95" weight="-8.89112885878112e-001"/><Con from="96" weight="-2.77415010528079e-001"/><Con from="97" weight="-6.57340277546737e-001"/><Con from="98" weight="-1.18498578870024e+000"/><Con from="99" weight="-6.06934624678475e-001"/></Neuron><Neuron id="110" bias="-3.31889146500088e-001"><Con from="0" weight="4.39818146728352e-002"/><Con from="1" weight="-2.68585260892522e-001"/><Con from="2" weight="-3.25394021488874e-001"/><Con from="3" weight="9.57956430931503e-002"/><Con from="4" weight="1.17114746897479e-001"/><Con from="5" weight="7.72751436895406e-002"/><Con from="6" weight="1.03647564238880e-001"/><Con from="7" weight="-4.16261355321049e-001"/><Con from="8" weight="-4.95525708982680e-001"/><Con from="9" weight="-4.02176464972651e-001"/><Con from="10" weight="-1.62457929853619e-001"/><Con from="11" weight="2.55688279975655e-002"/><Con from="12" weight="-3.22322794457682e-001"/><Con from="13" weight="2.60097789742673e-004"/><Con from="14" weight="-4.78914006941238e-001"/><Con from="15" weight="3.73717563113921e-002"/><Con from="16" weight="2.03009955119101e-001"/><Con from="17" weight="9.09105869276684e-002"/><Con from="18" weight="2.16345966718973e-001"/><Con from="19" weight="-5.39468584143890e-002"/><Con from="20" weight="5.18245270654575e-001"/><Con from="21" weight="-5.23655819482650e-001"/><Con from="22" weight="1.08483983720123e-001"/><Con from="23" weight="-1.60295059756309e-001"/><Con from="24" weight="-3.04766782117923e-001"/><Con from="25" weight="-1.08407474824604e-001"/><Con from="26" weight="5.90919466641081e-001"/><Con from="27" weight="-9.94755281330474e-002"/><Con from="28" weight="-2.36527577893777e-001"/><Con from="29" weight="-7.83339233594301e-002"/><Con from="30" weight="1.84051432743696e-001"/><Con from="31" weight="3.02712155596449e-001"/><Con from="32" weight="-5.26007520157847e-001"/><Con from="33" weight="-2.23762900902932e-001"/><Con from="34" weight="-5.75529812955487e-002"/><Con from="35" weight="-5.43760323909085e-002"/><Con from="36" weight="-2.16849300485102e-001"/><Con from="37" weight="-2.64806668290786e-002"/><Con from="38" weight="-2.02408194988981e-001"/><Con from="39" weight="-1.52949259035243e-002"/><Con from="40" weight="1.61025300417272e-001"/><Con from="41" weight="3.57476749974403e-001"/><Con from="42" weight="-3.29004194872196e-001"/><Con from="43" weight="1.79274000992966e-002"/><Con from="44" weight="-4.64992151500617e-001"/><Con from="45" weight="-3.44340168016503e-001"/><Con from="46" weight="-4.60733744564257e-002"/><Con from="47" weight="7.49055517563838e-001"/><Con from="48" weight="2.06955264944809e-001"/><Con from="49" weight="6.94536868805439e-001"/><Con from="50" weight="-4.11686181126311e-002"/><Con from="51" weight="1.16809215756793e-001"/><Con from="52" weight="-2.55045427651908e-001"/><Con from="53" weight="3.05076140238829e-002"/><Con from="54" weight="-2.21007440100262e-001"/><Con from="55" weight="2.19699778627616e-002"/><Con from="56" weight="1.62190934876895e-002"/><Con from="57" weight="1.02191716561548e-002"/><Con from="58" weight="-3.80074189708333e-001"/><Con from="59" weight="-2.78079457438029e-001"/><Con from="60" weight="-5.00279776566985e-001"/><Con from="61" weight="-6.52763362076886e-001"/><Con from="62" weight="3.16281048691600e-002"/><Con from="63" weight="-4.15450187675359e-001"/><Con from="64" weight="1.49520267476284e-001"/><Con from="65" weight="-3.41464303072326e-002"/><Con from="66" weight="7.49795221367272e-001"/><Con from="67" weight="1.81475671078332e-001"/><Con from="68" weight="7.28843400614445e-001"/><Con from="69" weight="-2.32726551928889e-001"/><Con from="70" weight="-1.84697596316984e-002"/><Con from="71" weight="-1.19445118458361e-001"/><Con from="72" weight="-1.42116171386299e-001"/><Con from="73" weight="-1.28126974368523e-001"/><Con from="74" weight="-7.51319748012834e-001"/><Con from="75" weight="-7.75884131765706e-001"/><Con from="76" weight="-3.24366092353512e-001"/><Con from="77" weight="-6.41995163305467e-001"/><Con from="78" weight="1.10934370953989e-001"/><Con from="79" weight="-4.38113916976990e-001"/><Con from="80" weight="-3.97057430289220e-001"/><Con from="81" weight="-1.53559326196078e-001"/><Con from="82" weight="4.00316261980655e-001"/><Con from="83" weight="-1.52814019802660e-001"/><Con from="84" weight="-2.55656321372684e-001"/><Con from="85" weight="-4.28277784099683e-001"/><Con from="86" weight="-7.33761344801280e-001"/><Con from="87" weight="-3.09304925807714e-001"/><Con from="88" weight="-1.13051017648692e-001"/><Con from="89" weight="1.17378417450247e-001"/><Con from="90" weight="-9.66573472471572e-003"/><Con from="91" weight="-1.29235929305574e-001"/><Con from="92" weight="-3.11881581676439e-001"/><Con from="93" weight="5.05882384117281e-001"/><Con from="94" weight="4.34973392655353e-001"/><Con from="95" weight="8.22538890629351e-001"/><Con from="96" weight="2.81012244192362e-001"/><Con from="97" weight="3.77693308918751e-001"/><Con from="98" weight="-8.13528382043944e-001"/><Con from="99" weight="7.37589734477186e-002"/></Neuron><Neuron id="111" bias="-2.39100342038567e-001"><Con from="0" weight="7.45568672465798e-002"/><Con from="1" weight="4.22820469484023e-001"/><Con from="2" weight="2.74587132503354e-001"/><Con from="3" weight="-9.62127237448217e-002"/><Con from="4" weight="9.15094075815595e-001"/><Con from="5" weight="-4.70398948839898e-001"/><Con from="6" weight="9.87641994314351e-001"/><Con from="7" weight="5.21368614707790e-001"/><Con from="8" weight="-1.95102294744459e-001"/><Con from="9" weight="-4.30097852271260e-001"/><Con from="10" weight="-4.39822983194264e-001"/><Con from="11" weight="5.51032937988264e-001"/><Con from="12" weight="-5.30750510600730e-001"/><Con from="13" weight="-5.20563985877332e-001"/><Con from="14" weight="5.98282324945359e-001"/><Con from="15" weight="8.78147861648956e-002"/><Con from="16" weight="-1.22763874504530e+000"/><Con from="17" weight="2.23841124638800e-001"/><Con from="18" weight="-5.51875239424481e-001"/><Con from="19" weight="-3.48481745618263e-001"/><Con from="20" weight="-3.96621619819630e-001"/><Con from="21" weight="-4.23221938719456e-001"/><Con from="22" weight="7.71822996023731e-001"/><Con from="23" weight="-6.34229394127698e-001"/><Con from="24" weight="-9.73595420085395e-002"/><Con from="25" weight="1.77580083599883e-001"/><Con from="26" weight="8.68605985698804e-001"/><Con from="27" weight="-1.83637601816635e-001"/><Con from="28" weight="-5.41728341933869e-001"/><Con from="29" weight="-2.52616007356680e-001"/><Con from="30" weight="-3.04612503109306e-001"/><Con from="31" weight="1.08715038436370e-001"/><Con from="32" weight="-1.19248363196590e+000"/><Con from="33" weight="4.38380547019856e-001"/><Con from="34" weight="3.26783030948392e-001"/><Con from="35" weight="2.79178249614901e-002"/><Con from="36" weight="-8.93548156474222e-002"/><Con from="37" weight="2.45973357418948e-001"/><Con from="38" weight="5.34650902003309e-001"/><Con from="39" weight="-5.69141815077625e-001"/><Con from="40" weight="-1.02507956885502e+000"/><Con from="41" weight="1.15923358423517e+000"/><Con from="42" weight="4.81466419648890e-001"/><Con from="43" weight="-9.79689579504677e-001"/><Con from="44" weight="4.70131658521225e-001"/><Con from="45" weight="-2.63312674061377e-002"/><Con from="46" weight="-5.92400807798976e-001"/><Con from="47" weight="8.73883633469546e-001"/><Con from="48" weight="3.94194613193352e-001"/><Con from="49" weight="-5.45566052952283e-001"/><Con from="50" weight="-4.08341677277743e-001"/><Con from="51" weight="-2.04258207424772e-001"/><Con from="52" weight="1.69609900960035e-001"/><Con from="53" weight="-3.87467651391272e-001"/><Con from="54" weight="5.71811866527438e-001"/><Con from="55" weight="-5.74054144966040e-001"/><Con from="56" weight="-4.45350196156525e-001"/><Con from="57" weight="8.32909557574078e-001"/><Con from="58" weight="3.11649998975338e-001"/><Con from="59" weight="1.97003233778650e-002"/><Con from="60" weight="4.75873176642603e-002"/><Con from="61" weight="-7.30419033621176e-001"/><Con from="62" weight="-1.25199692586052e-001"/><Con from="63" weight="5.27339519499693e-001"/><Con from="64" weight="1.40610173445584e+000"/><Con from="65" weight="-5.60874311966030e-001"/><Con from="66" weight="7.19645610469579e-001"/><Con from="67" weight="3.90576632889074e-001"/><Con from="68" weight="-3.41475531513948e-001"/><Con from="69" weight="-1.17142947865758e+000"/><Con from="70" weight="-9.92253368327159e-001"/><Con from="71" weight="7.38056121333389e-001"/><Con from="72" weight="-5.66962499499589e-001"/><Con from="73" weight="-5.96266745672668e-002"/><Con from="74" weight="-1.03492585763955e-001"/><Con from="75" weight="-3.13476122279122e-001"/><Con from="76" weight="3.90393439236352e-001"/><Con from="77" weight="-5.01944387192286e-001"/><Con from="78" weight="1.05238574046460e-001"/><Con from="79" weight="-4.53812679280641e-001"/><Con from="80" weight="-4.99968555755669e-001"/><Con from="81" weight="6.65282631554318e-001"/><Con from="82" weight="-3.29816861201842e-001"/><Con from="83" weight="-3.02607320957231e-002"/><Con from="84" weight="-5.26820936255275e-001"/><Con from="85" weight="1.02080184203635e+000"/><Con from="86" weight="-1.29648638487770e-001"/><Con from="87" weight="9.87680864491892e-002"/><Con from="88" weight="3.26945322934165e-001"/><Con from="89" weight="7.52650697246785e-001"/><Con from="90" weight="-9.92096168235835e-002"/><Con from="91" weight="-7.79829663862805e-001"/><Con from="92" weight="4.35442886472853e-001"/><Con from="93" weight="-1.47509790871750e-001"/><Con from="94" weight="4.51027331298086e-005"/><Con from="95" weight="6.99230767525976e-001"/><Con from="96" weight="-6.60807312125653e-002"/><Con from="97" weight="-8.16609417414213e-001"/><Con from="98" weight="-1.06899975692250e+000"/><Con from="99" weight="3.69480622734888e-002"/></Neuron><Neuron id="112" bias="-7.43865958939074e-001"><Con from="0" weight="-2.78747175313687e-001"/><Con from="1" weight="-1.71859987023400e-001"/><Con from="2" weight="-2.56073752027593e-001"/><Con from="3" weight="5.28191339104926e-001"/><Con from="4" weight="-5.11648666972532e-001"/><Con from="5" weight="1.81436354312269e+000"/><Con from="6" weight="-1.27509990964632e+000"/><Con from="7" weight="-4.54093108464290e-001"/><Con from="8" weight="-8.31977344678085e-001"/><Con from="9" weight="7.06055799017131e-001"/><Con from="10" weight="1.95095864529866e-001"/><Con from="11" weight="5.64218504342755e-001"/><Con from="12" weight="-1.83424686402756e+000"/><Con from="13" weight="1.16704639553633e+000"/><Con from="14" weight="-1.40083058429961e+000"/><Con from="15" weight="2.16742508314849e-002"/><Con from="16" weight="1.62690236230874e-001"/><Con from="17" weight="-4.51360224804871e-001"/><Con from="18" weight="4.65390183355298e-002"/><Con from="19" weight="2.54422290789715e+000"/><Con from="20" weight="-5.55452169058104e-002"/><Con from="21" weight="-9.50336650018817e-001"/><Con from="22" weight="1.87382524920615e+000"/><Con from="23" weight="-4.05815324235482e-002"/><Con from="24" weight="-2.14616998735830e-001"/><Con from="25" weight="9.01978268218708e-001"/><Con from="26" weight="-7.51881786372362e-001"/><Con from="27" weight="9.91476351069039e-002"/><Con from="28" weight="5.56067526653068e-001"/><Con from="29" weight="1.01437626396299e-001"/><Con from="30" weight="2.13285305540909e-001"/><Con from="31" weight="-1.21284870664380e+000"/><Con from="32" weight="-4.35943647270056e-001"/><Con from="33" weight="-1.09328377073260e+000"/><Con from="34" weight="2.31505774024271e-001"/><Con from="35" weight="-1.35796718726735e-001"/><Con from="36" weight="-9.03806724846108e-001"/><Con from="37" weight="-9.71271477745854e-001"/><Con from="38" weight="1.00887202423132e-002"/><Con from="39" weight="2.32816440866987e-001"/><Con from="40" weight="1.16086957690017e+000"/><Con from="41" weight="8.36892267895968e-002"/><Con from="42" weight="-1.48334947571436e+000"/><Con from="43" weight="9.50076334705200e-001"/><Con from="44" weight="-2.45115236492445e-001"/><Con from="45" weight="-1.49794953681972e-001"/><Con from="46" weight="4.40733328310641e-001"/><Con from="47" weight="1.22670720449246e-001"/><Con from="48" weight="-2.65022212599102e-001"/><Con from="49" weight="6.42869970394836e-001"/><Con from="50" weight="2.58287722414510e-001"/><Con from="51" weight="6.48154899482277e-002"/><Con from="52" weight="-3.40031698967397e-001"/><Con from="53" weight="-2.66428970769982e-001"/><Con from="54" weight="-1.03820569107495e-001"/><Con from="55" weight="7.14019835253088e-001"/><Con from="56" weight="6.08929142299251e-001"/><Con from="57" weight="3.27573904734690e-001"/><Con from="58" weight="-3.44833271952910e-002"/><Con from="59" weight="-3.32130646760103e-001"/><Con from="60" weight="-1.89494445152357e-001"/><Con from="61" weight="1.57478686954502e-001"/><Con from="62" weight="-6.58801127684923e-001"/><Con from="63" weight="-6.83043926518182e-002"/><Con from="64" weight="-1.17451077983444e+000"/><Con from="65" weight="4.56384276578395e-001"/><Con from="66" weight="5.15104346821213e-001"/><Con from="67" weight="-3.97290113660941e-001"/><Con from="68" weight="-2.15882017255448e-001"/><Con from="69" weight="-7.29047886165407e-001"/><Con from="70" weight="1.26976862800650e+000"/><Con from="71" weight="6.18553109259293e-001"/><Con from="72" weight="1.08531782849211e+000"/><Con from="73" weight="2.65712864393968e-002"/><Con from="74" weight="1.85481897931803e-001"/><Con from="75" weight="7.23091471814177e-001"/><Con from="76" weight="-1.07913226483753e+000"/><Con from="77" weight="-1.66513182046437e-001"/><Con from="78" weight="-5.05339934058258e-001"/><Con from="79" weight="1.00301076379028e-001"/><Con from="80" weight="4.96161308842309e-001"/><Con from="81" weight="-1.20812681141488e-001"/><Con from="82" weight="4.81353838688199e-001"/><Con from="83" weight="-1.14377138309031e-002"/><Con from="84" weight="-1.61708071106776e+000"/><Con from="85" weight="-8.85069711245706e-001"/><Con from="86" weight="8.93932247373652e-001"/><Con from="87" weight="-1.43863121750715e-001"/><Con from="88" weight="-1.13695701044343e+000"/><Con from="89" weight="-1.13340463834385e+000"/><Con from="90" weight="-4.16640121044019e-001"/><Con from="91" weight="1.26905519408929e+000"/><Con from="92" weight="-1.39644630085592e+000"/><Con from="93" weight="1.69889069617529e+000"/><Con from="94" weight="7.56817435613878e-001"/><Con from="95" weight="-3.51083002414237e-001"/><Con from="96" weight="-4.55078886684932e-001"/><Con from="97" weight="1.31189443369107e+000"/><Con from="98" weight="9.89496033341937e-001"/><Con from="99" weight="-3.03860682817078e-001"/></Neuron><Neuron id="113" bias="-5.42282584463940e-001"><Con from="0" weight="-3.70054537486579e-001"/><Con from="1" weight="1.04344971344161e+000"/><Con from="2" weight="-5.58041901707160e-001"/><Con from="3" weight="1.70202058217540e-001"/><Con from="4" weight="3.71271972874815e-001"/><Con from="5" weight="-1.73236402434718e+000"/><Con from="6" weight="6.91906777255909e-001"/><Con from="7" weight="6.23513548721271e-002"/><Con from="8" weight="-4.79535336282159e-001"/><Con from="9" weight="-6.87284146975778e-001"/><Con from="10" weight="-6.99504901264331e-001"/><Con from="11" weight="1.93668666900118e+000"/><Con from="12" weight="1.08488200799031e+000"/><Con from="13" weight="4.57087332748175e-002"/><Con from="14" weight="9.01564067541663e-001"/><Con from="15" weight="-1.66699564380939e+000"/><Con from="16" weight="-1.56219255981581e+000"/><Con from="17" weight="2.76262796931569e+000"/><Con from="18" weight="-6.93140077053717e-001"/><Con from="19" weight="-2.56126429166228e+000"/><Con from="20" weight="-6.31729902146992e-001"/><Con from="21" weight="-5.53865171863368e-001"/><Con from="22" weight="-8.01440835497358e-001"/><Con from="23" weight="3.26655539980683e-002"/><Con from="24" weight="9.39654699683826e-001"/><Con from="25" weight="-2.65661169950784e-001"/><Con from="26" weight="3.68453798256535e-001"/><Con from="27" weight="-3.47554482798354e+000"/><Con from="28" weight="7.37547380726296e-001"/><Con from="29" weight="-1.12650262485981e+000"/><Con from="30" weight="-8.11381821753240e-001"/><Con from="31" weight="1.75141855669669e+000"/><Con from="32" weight="9.01985519884134e-001"/><Con from="33" weight="-4.50565622122600e-001"/><Con from="34" weight="1.77168132218325e-001"/><Con from="35" weight="-4.10451219553229e-001"/><Con from="36" weight="-1.87490842649989e+000"/><Con from="37" weight="5.25945422263260e-001"/><Con from="38" weight="-3.42778777724741e-001"/><Con from="39" weight="-3.08471134345239e-001"/><Con from="40" weight="-1.06302585831603e+000"/><Con from="41" weight="5.45922392238492e-001"/><Con from="42" weight="-1.86727352361715e-001"/><Con from="43" weight="-1.45194874975274e+000"/><Con from="44" weight="4.70034973758802e-001"/><Con from="45" weight="-3.43993734265052e-001"/><Con from="46" weight="3.72745973351293e-001"/><Con from="47" weight="9.29567389270488e-001"/><Con from="48" weight="1.32924071594064e+000"/><Con from="49" weight="-5.23162153165350e-001"/><Con from="50" weight="-1.09688824062410e+000"/><Con from="51" weight="1.11124600849916e+000"/><Con from="52" weight="7.21273938223959e-001"/><Con from="53" weight="1.18865056580615e-001"/><Con from="54" weight="-3.67448370931408e-001"/><Con from="55" weight="-7.46865392279031e-001"/><Con from="56" weight="2.16516061961681e-001"/><Con from="57" weight="6.86280376142483e-001"/><Con from="58" weight="5.19957313190169e-001"/><Con from="59" weight="4.88653744603726e-001"/><Con from="60" weight="-3.43373402569315e-002"/><Con from="61" weight="-2.84751872218111e-001"/><Con from="62" weight="1.86426304897717e-001"/><Con from="63" weight="1.22178427892871e-001"/><Con from="64" weight="1.78968709132359e-001"/><Con from="65" weight="3.69490923735151e-001"/><Con from="66" weight="1.82095207916023e-001"/><Con from="67" weight="1.41036378919169e+000"/><Con from="68" weight="1.73351934226693e+000"/><Con from="69" weight="-1.19606725592767e+000"/><Con from="70" weight="1.30694312136414e-001"/><Con from="71" weight="-1.37383573032896e+000"/><Con from="72" weight="-6.92826917886348e-001"/><Con from="73" weight="-2.84245715271715e-001"/><Con from="74" weight="-6.56735606595473e-001"/><Con from="75" weight="-1.75467907592385e+000"/><Con from="76" weight="5.56126892631737e-001"/><Con from="77" weight="3.26710136216457e-001"/><Con from="78" weight="-1.93651228759473e+000"/><Con from="79" weight="-8.64032461958360e-001"/><Con from="80" weight="-1.14290556090601e+000"/><Con from="81" weight="6.97790543863385e-002"/><Con from="82" weight="-7.17622870605551e-001"/><Con from="83" weight="-6.32778229828254e-001"/><Con from="84" weight="2.19173246415710e+000"/><Con from="85" weight="-1.83409509163884e+000"/><Con from="86" weight="-8.90772474876147e-002"/><Con from="87" weight="-8.46911253686667e-001"/><Con from="88" weight="-2.49550377005568e-001"/><Con from="89" weight="2.62277618204618e+000"/><Con from="90" weight="3.16908114584616e-002"/><Con from="91" weight="-1.71565477007604e+000"/><Con from="92" weight="1.86615664672752e+000"/><Con from="93" weight="8.05750819106280e-002"/><Con from="94" weight="-4.54357319890840e-001"/><Con from="95" weight="1.20032425051737e-001"/><Con from="96" weight="1.00231359547658e+000"/><Con from="97" weight="4.70763817809003e-002"/><Con from="98" weight="1.70950620413303e+000"/><Con from="99" weight="-8.77172580828187e-001"/></Neuron><Neuron id="114" bias="-1.47180401922134e-001"><Con from="0" weight="-1.49272367821693e-001"/><Con from="1" weight="-1.03879827549275e-002"/><Con from="2" weight="1.02576091428881e-001"/><Con from="3" weight="-7.96165393450260e-002"/><Con from="4" weight="-2.45252233572217e-001"/><Con from="5" weight="2.57097125942028e-001"/><Con from="6" weight="-3.19764138224623e-001"/><Con from="7" weight="1.56339705998165e-001"/><Con from="8" weight="-3.41463296228709e-001"/><Con from="9" weight="1.03919137291034e-002"/><Con from="10" weight="-3.19121561869206e-001"/><Con from="11" weight="4.53249701495775e-002"/><Con from="12" weight="1.44052423095086e-001"/><Con from="13" weight="-4.94821868991267e-002"/><Con from="14" weight="-3.03945765480624e-001"/><Con from="15" weight="4.39733137781706e-002"/><Con from="16" weight="-2.25116185581894e-001"/><Con from="17" weight="-3.79136596842604e-001"/><Con from="18" weight="-1.96338535704063e-001"/><Con from="19" weight="8.41267760390449e-002"/><Con from="20" weight="1.12324389221497e-001"/><Con from="21" weight="-3.17678362159404e-001"/><Con from="22" weight="1.57541743671805e-001"/><Con from="23" weight="2.63393553445102e-001"/><Con from="24" weight="4.85292694127160e-003"/><Con from="25" weight="1.15930205423645e-001"/><Con from="26" weight="3.68652642530853e-002"/><Con from="27" weight="1.10508307027523e-001"/><Con from="28" weight="-1.67386158719619e-001"/><Con from="29" weight="1.29222363703889e-001"/><Con from="30" weight="-1.40388175798284e-001"/><Con from="31" weight="-2.24635032568600e-002"/><Con from="32" weight="2.06364994648175e-003"/><Con from="33" weight="-1.20127791454347e-001"/><Con from="34" weight="1.67637663764102e-001"/><Con from="35" weight="-5.03858316032052e-001"/><Con from="36" weight="-1.52325487458933e-001"/><Con from="37" weight="-9.13918867784216e-002"/><Con from="38" weight="-1.41051708386089e-001"/><Con from="39" weight="-3.80192305291596e-001"/><Con from="40" weight="5.48045014673806e-002"/><Con from="41" weight="-4.72339730415486e-002"/><Con from="42" weight="-2.16621273214291e-001"/><Con from="43" weight="-1.31655942389909e-001"/><Con from="44" weight="9.99098606507652e-003"/><Con from="45" weight="1.93937416011618e-001"/><Con from="46" weight="-1.44104099880758e-001"/><Con from="47" weight="4.04186188484203e-001"/><Con from="48" weight="1.51350837813866e-002"/><Con from="49" weight="4.08668351097334e-001"/><Con from="50" weight="-4.96781705301763e-001"/><Con from="51" weight="1.46459703776428e-001"/><Con from="52" weight="-3.02483527315599e-001"/><Con from="53" weight="-1.48880767986956e-001"/><Con from="54" weight="-1.36978148216825e-001"/><Con from="55" weight="-3.79569359477795e-001"/><Con from="56" weight="3.05026432235277e-001"/><Con from="57" weight="4.89318771408754e-002"/><Con from="58" weight="-1.56814680374626e-001"/><Con from="59" weight="3.65585967825847e-004"/><Con from="60" weight="-2.85086433666522e-002"/><Con from="61" weight="1.90076229187442e-001"/><Con from="62" weight="-4.95816570528398e-002"/><Con from="63" weight="2.13058740435713e-001"/><Con from="64" weight="5.38888161808631e-001"/><Con from="65" weight="-1.56105371867040e-001"/><Con from="66" weight="3.02726270396757e-001"/><Con from="67" weight="4.31419015717490e-002"/><Con from="68" weight="4.36689434353456e-001"/><Con from="69" weight="-3.00731934878952e-001"/><Con from="70" weight="7.29326273437117e-003"/><Con from="71" weight="-1.04367839792537e-001"/><Con from="72" weight="-7.74690373910381e-003"/><Con from="73" weight="-2.14192244715402e-003"/><Con from="74" weight="-2.76037771887831e-001"/><Con from="75" weight="-2.84796022691394e-001"/><Con from="76" weight="-1.23268848237862e-001"/><Con from="77" weight="-1.17233537403664e-001"/><Con from="78" weight="-1.69741743659591e-001"/><Con from="79" weight="-1.68999564599234e-001"/><Con from="80" weight="-1.24751088746867e-001"/><Con from="81" weight="-1.70557688875905e-001"/><Con from="82" weight="3.76855436483868e-001"/><Con from="83" weight="7.36906467764085e-002"/><Con from="84" weight="-8.32091593406201e-002"/><Con from="85" weight="2.33977523941933e-002"/><Con from="86" weight="-1.03547032940448e-001"/><Con from="87" weight="-3.54935661057300e-002"/><Con from="88" weight="2.16846976239232e-002"/><Con from="89" weight="3.33316277245052e-001"/><Con from="90" weight="-1.50305434501403e-001"/><Con from="91" weight="-2.74833641737882e-001"/><Con from="92" weight="-2.35976797085966e-002"/><Con from="93" weight="1.84722886378045e-001"/><Con from="94" weight="4.57170057650326e-003"/><Con from="95" weight="4.01121196037879e-001"/><Con from="96" weight="2.19549823299573e-001"/><Con from="97" weight="4.04271362226850e-001"/><Con from="98" weight="6.48756126120081e-002"/><Con from="99" weight="-1.18472352796064e-001"/></Neuron><Neuron id="115" bias="-3.33188390804820e-001"><Con from="0" weight="3.18737645875709e-001"/><Con from="1" weight="5.26831702564909e-001"/><Con from="2" weight="-2.02140619236292e-001"/><Con from="3" weight="-5.22910696843769e-001"/><Con from="4" weight="-4.81534459331690e-001"/><Con from="5" weight="8.77050029464800e-001"/><Con from="6" weight="-9.01671835250507e-001"/><Con from="7" weight="-2.88382951514500e-001"/><Con from="8" weight="1.27342136466264e-001"/><Con from="9" weight="-2.07543513306728e-002"/><Con from="10" weight="-2.05042842232083e-001"/><Con from="11" weight="-8.42116672048424e-001"/><Con from="12" weight="-8.77132506859552e-002"/><Con from="13" weight="3.28090476859641e-002"/><Con from="14" weight="9.66501386369173e-001"/><Con from="15" weight="5.22768320830191e-001"/><Con from="16" weight="-9.61774931963143e-002"/><Con from="17" weight="1.66025309120794e-001"/><Con from="18" weight="-2.13204771969656e-001"/><Con from="19" weight="1.23370380591661e+000"/><Con from="20" weight="9.00522104600844e-001"/><Con from="21" weight="3.38906654097728e-001"/><Con from="22" weight="-8.34881245140491e-001"/><Con from="23" weight="1.47922225240825e-002"/><Con from="24" weight="4.42703169348641e-001"/><Con from="25" weight="5.35167462776388e-001"/><Con from="26" weight="-8.47069457980993e-002"/><Con from="27" weight="2.79081304699133e-001"/><Con from="28" weight="1.31763776973245e-001"/><Con from="29" weight="-7.79453436649912e-001"/><Con from="30" weight="-2.12152907290745e-001"/><Con from="31" weight="-2.96007633072515e-001"/><Con from="32" weight="-2.49212901902692e-001"/><Con from="33" weight="3.17784324115772e-001"/><Con from="34" weight="-4.55818442353731e-001"/><Con from="35" weight="-8.02853108670618e-001"/><Con from="36" weight="1.26794722269316e-001"/><Con from="37" weight="-5.92846608878873e-001"/><Con from="38" weight="4.83313127534244e-001"/><Con from="39" weight="-5.87685728445898e-001"/><Con from="40" weight="2.67421354395476e-001"/><Con from="41" weight="7.39481906565519e-001"/><Con from="42" weight="9.49761061581928e-001"/><Con from="43" weight="-3.61280980913213e-001"/><Con from="44" weight="-3.62988638764401e-001"/><Con from="45" weight="4.44002361603200e-002"/><Con from="46" weight="8.27852984630085e-002"/><Con from="47" weight="-1.67468972019856e+000"/><Con from="48" weight="1.17851014148606e-002"/><Con from="49" weight="3.75303109280461e-003"/><Con from="50" weight="1.12732615647488e-001"/><Con from="51" weight="6.58709160120748e-001"/><Con from="52" weight="6.65894944042532e-001"/><Con from="53" weight="-1.80515761011921e-001"/><Con from="54" weight="4.36381457544540e-001"/><Con from="55" weight="-7.34193381217888e-001"/><Con from="56" weight="-8.53838949358837e-001"/><Con from="57" weight="2.90906251499356e-001"/><Con from="58" weight="9.57806059609066e-001"/><Con from="59" weight="2.51231196962196e-001"/><Con from="60" weight="4.30070957392803e-001"/><Con from="61" weight="-4.74481820971822e-001"/><Con from="62" weight="9.01715621200614e-001"/><Con from="63" weight="-5.77657827074286e-001"/><Con from="64" weight="-1.69392780318234e+000"/><Con from="65" weight="5.95695319808419e-002"/><Con from="66" weight="-1.64118557685610e+000"/><Con from="67" weight="-4.03935227361997e-001"/><Con from="68" weight="-2.12560218795564e-001"/><Con from="69" weight="4.21838040674281e-001"/><Con from="70" weight="1.43753430577241e-001"/><Con from="71" weight="4.80355798238010e-001"/><Con from="72" weight="-2.03518705751777e-001"/><Con from="73" weight="-4.14388208187213e-001"/><Con from="74" weight="-8.87947358833899e-002"/><Con from="75" weight="-6.57567194439278e-002"/><Con from="76" weight="-3.63501870546536e-001"/><Con from="77" weight="-2.80237275500012e-001"/><Con from="78" weight="1.68017359806002e-001"/><Con from="79" weight="-2.79209350367577e-001"/><Con from="80" weight="-2.66847796115140e-001"/><Con from="81" weight="-4.90500840859605e-001"/><Con from="82" weight="-4.32836786245194e-001"/><Con from="83" weight="-1.42712472520257e-001"/><Con from="84" weight="6.48389089672859e-001"/><Con from="85" weight="1.44551381368241e-001"/><Con from="86" weight="6.60298470143884e-001"/><Con from="87" weight="3.23826228425455e-001"/><Con from="88" weight="-4.76259069006338e-001"/><Con from="89" weight="7.76784690011153e-002"/><Con from="90" weight="-5.23085049459025e-001"/><Con from="91" weight="7.69941916288680e-001"/><Con from="92" weight="7.29245117724490e-002"/><Con from="93" weight="-5.28193850390344e-001"/><Con from="94" weight="-1.64221477389417e-001"/><Con from="95" weight="-9.55243319286462e-001"/><Con from="96" weight="-2.01514207068832e-001"/><Con from="97" weight="9.90140091691823e-002"/><Con from="98" weight="4.18779811912344e-001"/><Con from="99" weight="3.67641460258592e-001"/></Neuron><Neuron id="116" bias="-8.93897129495638e-001"><Con from="0" weight="5.08865940945944e-001"/><Con from="1" weight="-2.04987716957917e-002"/><Con from="2" weight="-3.32217589495828e-001"/><Con from="3" weight="3.96190384292755e-001"/><Con from="4" weight="1.28671852266005e-001"/><Con from="5" weight="-1.46318523799489e+000"/><Con from="6" weight="1.64718230085046e+000"/><Con from="7" weight="7.56873045670305e-001"/><Con from="8" weight="1.45084579410699e+000"/><Con from="9" weight="-2.19180079684805e+000"/><Con from="10" weight="-5.91553867486407e-001"/><Con from="11" weight="-1.28980313894935e+000"/><Con from="12" weight="9.49910625147376e-002"/><Con from="13" weight="-1.52220153133393e+000"/><Con from="14" weight="1.64882721318657e+000"/><Con from="15" weight="-9.42835036267845e-001"/><Con from="16" weight="2.70431229951183e-001"/><Con from="17" weight="1.54972860601144e+000"/><Con from="18" weight="-2.60674665696257e-001"/><Con from="19" weight="-1.13739633261631e+000"/><Con from="20" weight="8.03520272652572e-001"/><Con from="21" weight="4.77715510090556e-001"/><Con from="22" weight="-2.94073762702989e+000"/><Con from="23" weight="-8.86432035091240e-001"/><Con from="24" weight="4.04181240651739e-002"/><Con from="25" weight="-1.51053331667381e-002"/><Con from="26" weight="-3.03369909005340e-001"/><Con from="27" weight="3.54683239974187e-001"/><Con from="28" weight="-1.26035333959837e+000"/><Con from="29" weight="3.79151088680599e-002"/><Con from="30" weight="-4.57164404604382e-001"/><Con from="31" weight="1.21384019477145e+000"/><Con from="32" weight="-3.11973149867258e-001"/><Con from="33" weight="1.87470064874441e+000"/><Con from="34" weight="-2.59941096902627e-001"/><Con from="35" weight="6.45333879140430e-001"/><Con from="36" weight="8.82974256224490e-001"/><Con from="37" weight="1.56439629656145e+000"/><Con from="38" weight="7.57323499242581e-002"/><Con from="39" weight="5.60391120367425e-002"/><Con from="40" weight="-3.57107923635598e-001"/><Con from="41" weight="6.62694482399219e-001"/><Con from="42" weight="1.21591180955693e+000"/><Con from="43" weight="9.20324759314580e-002"/><Con from="44" weight="-7.04653866171061e-002"/><Con from="45" weight="-1.00018370824304e+000"/><Con from="46" weight="-4.22062239848028e-001"/><Con from="47" weight="-5.70019101639775e-001"/><Con from="48" weight="4.42413326288088e-001"/><Con from="49" weight="-2.58636007326293e-001"/><Con from="50" weight="4.26112898969097e-002"/><Con from="51" weight="3.36642954778408e-001"/><Con from="52" weight="1.87993089997314e-001"/><Con from="53" weight="2.44342754316858e-001"/><Con from="54" weight="1.40973614737021e-001"/><Con from="55" weight="-6.08133276117152e-001"/><Con from="56" weight="-8.65316707969866e-001"/><Con from="57" weight="-2.62312393208494e-001"/><Con from="58" weight="5.91014869246039e-001"/><Con from="59" weight="8.61577162477111e-001"/><Con from="60" weight="-5.19824317526826e-001"/><Con from="61" weight="-9.61901659236148e-001"/><Con from="62" weight="2.15844874966172e+000"/><Con from="63" weight="1.23483641855225e+000"/><Con from="64" weight="-3.54703637764496e-001"/><Con from="65" weight="-2.92148818867213e-001"/><Con from="66" weight="-8.79152074433048e-001"/><Con from="67" weight="-5.07696040512875e-001"/><Con from="68" weight="-8.64017060241814e-002"/><Con from="69" weight="2.32503552567556e+000"/><Con from="70" weight="-1.11685119847153e+000"/><Con from="71" weight="1.37545388570452e+000"/><Con from="72" weight="-1.49652038848829e+000"/><Con from="73" weight="-4.33421544332614e-001"/><Con from="74" weight="3.88979502520809e-001"/><Con from="75" weight="-3.50011509712487e-001"/><Con from="76" weight="1.46520129384403e+000"/><Con from="77" weight="-6.54022229187468e-001"/><Con from="78" weight="3.11585438971845e-001"/><Con from="79" weight="2.62201636094328e-001"/><Con from="80" weight="-4.59060742960592e-001"/><Con from="81" weight="1.48103695824770e+000"/><Con from="82" weight="-2.98849001726724e+000"/><Con from="83" weight="1.89685738323851e+000"/><Con from="84" weight="2.84970971753675e-001"/><Con from="85" weight="2.05911550209741e+000"/><Con from="86" weight="-1.92389196222614e+000"/><Con from="87" weight="8.06958605013072e-001"/><Con from="88" weight="8.22975710547554e-001"/><Con from="89" weight="-3.92987756311106e-001"/><Con from="90" weight="4.31704797874919e-001"/><Con from="91" weight="-8.61177566094402e-001"/><Con from="92" weight="6.29239327214131e-001"/><Con from="93" weight="-1.57414635118276e+000"/><Con from="94" weight="-4.03076223348895e-001"/><Con from="95" weight="-1.01794513214470e+000"/><Con from="96" weight="-1.53721109751405e+000"/><Con from="97" weight="-1.94226453127807e+000"/><Con from="98" weight="-1.76558099914619e+000"/><Con from="99" weight="5.67114140404239e-001"/></Neuron><Neuron id="117" bias="-4.94264237313820e-001"><Con from="0" weight="1.60013390889078e-001"/><Con from="1" weight="6.56924323075936e-002"/><Con from="2" weight="-2.84979259771674e-001"/><Con from="3" weight="-3.12416681841724e-001"/><Con from="4" weight="-5.94349000854599e-001"/><Con from="5" weight="-5.88209469009270e-001"/><Con from="6" weight="1.51516536333960e-001"/><Con from="7" weight="-2.77460298923667e-001"/><Con from="8" weight="5.29231667294251e-001"/><Con from="9" weight="3.75082807219290e-001"/><Con from="10" weight="-1.24483346361962e-001"/><Con from="11" weight="-5.31897694610279e-001"/><Con from="12" weight="-2.51182064306566e-001"/><Con from="13" weight="-5.68752754659269e-001"/><Con from="14" weight="1.50600200224623e-001"/><Con from="15" weight="-6.63637601020090e-001"/><Con from="16" weight="-9.71730505834051e-001"/><Con from="17" weight="-6.78624275259620e-001"/><Con from="18" weight="-3.37728599453835e-001"/><Con from="19" weight="6.03532930027478e-001"/><Con from="20" weight="1.46720423469053e-001"/><Con from="21" weight="3.05362740207470e-001"/><Con from="22" weight="-8.99926776155790e-002"/><Con from="23" weight="-1.11225509086344e+000"/><Con from="24" weight="3.09787997875387e-001"/><Con from="25" weight="-1.32996478574751e+000"/><Con from="26" weight="-5.07024553550153e-001"/><Con from="27" weight="1.40345351238776e+000"/><Con from="28" weight="-2.22224803707109e-001"/><Con from="29" weight="-3.26431492496676e-001"/><Con from="30" weight="-1.33763389425083e-001"/><Con from="31" weight="1.22495804856467e+000"/><Con from="32" weight="5.48699010887777e-001"/><Con from="33" weight="1.63492029730020e-001"/><Con from="34" weight="2.10858565526479e-002"/><Con from="35" weight="-2.14246380288145e-001"/><Con from="36" weight="-2.00134909419955e-001"/><Con from="37" weight="3.34049529973043e-001"/><Con from="38" weight="-4.82124130297996e-002"/><Con from="39" weight="-2.11749135374400e-001"/><Con from="40" weight="6.44372879300309e-001"/><Con from="41" weight="-9.05772782308237e-002"/><Con from="42" weight="5.88272654689781e-001"/><Con from="43" weight="-6.34751536026970e-001"/><Con from="44" weight="1.13538602667155e-001"/><Con from="45" weight="-1.35145165808096e-001"/><Con from="46" weight="-6.15904639597055e-001"/><Con from="47" weight="8.10274103352409e-001"/><Con from="48" weight="-1.83287592567449e-001"/><Con from="49" weight="9.93416103736342e-002"/><Con from="50" weight="-1.55507335869724e-001"/><Con from="51" weight="5.08223744976169e-001"/><Con from="52" weight="3.07696354702133e-001"/><Con from="53" weight="-1.43733390789848e-001"/><Con from="54" weight="-8.57991848160088e-002"/><Con from="55" weight="-4.41967779236827e-001"/><Con from="56" weight="1.12873187383881e-001"/><Con from="57" weight="-3.49546784502116e-001"/><Con from="58" weight="1.03442170137130e-001"/><Con from="59" weight="-1.07974718494946e-001"/><Con from="60" weight="4.90786652481278e-001"/><Con from="61" weight="1.40785404156615e+000"/><Con from="62" weight="3.99711170540830e-001"/><Con from="63" weight="-2.57933737938954e-001"/><Con from="64" weight="-1.51194201638302e+000"/><Con from="65" weight="-6.11144955910430e-001"/><Con from="66" weight="6.03899866506836e-001"/><Con from="67" weight="-5.98619865225502e-001"/><Con from="68" weight="5.21524251581601e-002"/><Con from="69" weight="-4.49570355514923e-001"/><Con from="70" weight="-1.78913731092708e-001"/><Con from="71" weight="8.81138274012345e-001"/><Con from="72" weight="-7.51153684941640e-001"/><Con from="73" weight="-1.19162003175049e-001"/><Con from="74" weight="2.66601999706258e-001"/><Con from="75" weight="-3.17855368534675e-003"/><Con from="76" weight="2.53094900814773e-001"/><Con from="77" weight="-5.34509836345710e-001"/><Con from="78" weight="1.98723895792347e-001"/><Con from="79" weight="4.82923387104258e-001"/><Con from="80" weight="1.52179964893955e-001"/><Con from="81" weight="7.48101999242409e-001"/><Con from="82" weight="-7.73062155729647e-001"/><Con from="83" weight="8.36158116294666e-001"/><Con from="84" weight="3.62523284794049e-001"/><Con from="85" weight="8.82054849008303e-002"/><Con from="86" weight="2.11581826657764e-001"/><Con from="87" weight="1.21395807165666e-001"/><Con from="88" weight="2.39938444200837e-001"/><Con from="89" weight="4.01316337325890e-001"/><Con from="90" weight="2.07279406398042e-001"/><Con from="91" weight="-4.14910852341416e-001"/><Con from="92" weight="-5.66298803657431e-001"/><Con from="93" weight="-9.19817460569645e-001"/><Con from="94" weight="-7.18533084984583e-001"/><Con from="95" weight="-1.44009732173198e-001"/><Con from="96" weight="2.94264425419288e-001"/><Con from="97" weight="2.62392212887709e-001"/><Con from="98" weight="4.52099227622096e-001"/><Con from="99" weight="9.12403263561538e-002"/></Neuron><Neuron id="118" bias="-3.11045657387673e-001"><Con from="0" weight="2.61156389401993e-001"/><Con from="1" weight="3.89451108291468e-001"/><Con from="2" weight="-1.65125853519848e-001"/><Con from="3" weight="-6.96634555200092e-001"/><Con from="4" weight="-8.75378874385166e-001"/><Con from="5" weight="-1.22918214514080e-001"/><Con from="6" weight="-7.46592316803515e-001"/><Con from="7" weight="-2.22662448498442e-001"/><Con from="8" weight="-8.41590825595495e-002"/><Con from="9" weight="-2.56003636123042e-001"/><Con from="10" weight="-2.86764419584236e-001"/><Con from="11" weight="-6.78491361966411e-001"/><Con from="12" weight="4.73226803273406e-001"/><Con from="13" weight="5.80160501211094e-002"/><Con from="14" weight="1.96180277408735e-002"/><Con from="15" weight="-1.84616043771826e-001"/><Con from="16" weight="-9.57354123523335e-001"/><Con from="17" weight="-3.53859392509768e-001"/><Con from="18" weight="-5.69007160153071e-001"/><Con from="19" weight="-6.71761864265429e-002"/><Con from="20" weight="1.05411082341995e-001"/><Con from="21" weight="-2.88120117260672e-001"/><Con from="22" weight="-7.29532311484227e-001"/><Con from="23" weight="5.92246194432345e-001"/><Con from="24" weight="4.16035415904627e-001"/><Con from="25" weight="5.35973770632312e-001"/><Con from="26" weight="-4.76189680918277e-001"/><Con from="27" weight="-3.39714111130616e-002"/><Con from="28" weight="-1.49367498866359e-001"/><Con from="29" weight="-6.26498354452612e-002"/><Con from="30" weight="1.38331501009859e-001"/><Con from="31" weight="-3.03116982563190e-002"/><Con from="32" weight="5.43893890775568e-001"/><Con from="33" weight="2.14170444092089e-002"/><Con from="34" weight="-7.55443045102577e-002"/><Con from="35" weight="2.20671224966028e-001"/><Con from="36" weight="4.39066461373380e-002"/><Con from="37" weight="1.02269246469496e-001"/><Con from="38" weight="1.99408826903427e-001"/><Con from="39" weight="6.80779225342384e-001"/><Con from="40" weight="-1.12370131435388e+000"/><Con from="41" weight="3.07036338632298e-002"/><Con from="42" weight="7.83392619018113e-001"/><Con from="43" weight="-1.21932978860966e+000"/><Con from="44" weight="6.01122126583297e-001"/><Con from="45" weight="-1.56601572782330e-001"/><Con from="46" weight="-5.84006247288341e-002"/><Con from="47" weight="-5.90847511795461e-002"/><Con from="48" weight="-9.48139001860926e-001"/><Con from="49" weight="-9.33429815974084e-001"/><Con from="50" weight="1.54936458273301e-001"/><Con from="51" weight="-7.51200588912642e-001"/><Con from="52" weight="6.02997832061852e-001"/><Con from="53" weight="1.04509746250646e-001"/><Con from="54" weight="1.86214280087787e-001"/><Con from="55" weight="6.48407307139132e-001"/><Con from="56" weight="-1.58818722408303e+000"/><Con from="57" weight="-1.08792556059450e-002"/><Con from="58" weight="7.33792636891430e-001"/><Con from="59" weight="-1.31554843389182e-001"/><Con from="60" weight="6.97920489269929e-001"/><Con from="61" weight="-1.09017603293190e+000"/><Con from="62" weight="8.28305513063128e-001"/><Con from="63" weight="5.97585210059975e-001"/><Con from="64" weight="-1.45311530144815e+000"/><Con from="65" weight="-5.29632103773511e-002"/><Con from="66" weight="-8.85991557947916e-002"/><Con from="67" weight="-1.00055104588025e+000"/><Con from="68" weight="-9.46730070498037e-001"/><Con from="69" weight="-7.83297790946032e-001"/><Con from="70" weight="-4.20953936136341e-001"/><Con from="71" weight="-6.31377297224657e-001"/><Con from="72" weight="-3.83264575300669e-001"/><Con from="73" weight="-1.38673066009550e-001"/><Con from="74" weight="7.87409857550679e-001"/><Con from="75" weight="1.03069104471748e+000"/><Con from="76" weight="6.20882748587121e-001"/><Con from="77" weight="1.67266590797584e-001"/><Con from="78" weight="-3.15611365022293e-002"/><Con from="79" weight="-4.34613222595051e-001"/><Con from="80" weight="-5.32828286092732e-001"/><Con from="81" weight="5.65413205911746e-001"/><Con from="82" weight="-5.89338902381479e-001"/><Con from="83" weight="-4.18617022420562e-001"/><Con from="84" weight="2.25670047619204e-001"/><Con from="85" weight="6.99356628032938e-001"/><Con from="86" weight="9.40633072902128e-002"/><Con from="87" weight="3.59227347369313e-003"/><Con from="88" weight="7.35754526843035e-002"/><Con from="89" weight="2.16990859739364e-002"/><Con from="90" weight="-1.97163559432787e-002"/><Con from="91" weight="2.32073237343867e-001"/><Con from="92" weight="2.61382987743225e-001"/><Con from="93" weight="-1.00574443078331e+000"/><Con from="94" weight="-8.44568757111164e-001"/><Con from="95" weight="-8.66141994654648e-001"/><Con from="96" weight="-3.46023632284757e-002"/><Con from="97" weight="-3.83643172228083e-001"/><Con from="98" weight="-1.68214840818838e-001"/><Con from="99" weight="2.03403653046950e-001"/></Neuron><Neuron id="119" bias="1.13958205409453e-001"><Con from="0" weight="8.60937080178085e-002"/><Con from="1" weight="3.79985388204226e-001"/><Con from="2" weight="-1.28359482646639e-001"/><Con from="3" weight="-3.85028766217811e-001"/><Con from="4" weight="-6.20994537884595e-001"/><Con from="5" weight="1.13439963782863e-001"/><Con from="6" weight="-1.43644834320075e+000"/><Con from="7" weight="3.85060111670222e-001"/><Con from="8" weight="-5.75238014300970e-001"/><Con from="9" weight="1.19243743712871e+000"/><Con from="10" weight="-9.33367984199460e-001"/><Con from="11" weight="3.60704908499388e-002"/><Con from="12" weight="1.07578274188108e+000"/><Con from="13" weight="5.66400710643774e-001"/><Con from="14" weight="-1.99315873610248e-001"/><Con from="15" weight="-4.70249979643480e-004"/><Con from="16" weight="-9.72184481599633e-001"/><Con from="17" weight="-1.99287427061306e+000"/><Con from="18" weight="-4.57847210593926e-001"/><Con from="19" weight="3.04737738592017e-001"/><Con from="20" weight="4.64913638201429e-001"/><Con from="21" weight="-1.38667036113697e-001"/><Con from="22" weight="7.47269467265763e-002"/><Con from="23" weight="2.12207161684098e-001"/><Con from="24" weight="8.08049863691342e-001"/><Con from="25" weight="8.69727649786403e-001"/><Con from="26" weight="-5.03133880083789e-001"/><Con from="27" weight="1.05609081781585e-001"/><Con from="28" weight="-4.60211915379405e-001"/><Con from="29" weight="-5.38365984464629e-001"/><Con from="30" weight="-1.44471320120237e-001"/><Con from="31" weight="-1.05926780024690e+000"/><Con from="32" weight="-6.96012017863817e-002"/><Con from="33" weight="-5.23859853265135e-001"/><Con from="34" weight="-5.93449133678407e-001"/><Con from="35" weight="-5.02800132785089e-001"/><Con from="36" weight="-1.01503831889443e+000"/><Con from="37" weight="-3.46921300388223e-001"/><Con from="38" weight="6.81594496628286e-001"/><Con from="39" weight="-2.18517386338241e-001"/><Con from="40" weight="-2.69230990504748e-001"/><Con from="41" weight="-4.55699581948458e-001"/><Con from="42" weight="1.73483957036740e-001"/><Con from="43" weight="4.96906351955055e-002"/><Con from="44" weight="3.04837030466091e-001"/><Con from="45" weight="3.33244051171302e-001"/><Con from="46" weight="3.09649430972614e-002"/><Con from="47" weight="9.78823130474142e-001"/><Con from="48" weight="-7.01785994783801e-001"/><Con from="49" weight="-1.29524931768405e-002"/><Con from="50" weight="-1.72306968068309e-001"/><Con from="51" weight="1.34343677510957e-001"/><Con from="52" weight="-1.11487156336552e+000"/><Con from="53" weight="-4.43858578751263e-001"/><Con from="54" weight="7.00401639078228e-001"/><Con from="55" weight="-2.55398882169471e-001"/><Con from="56" weight="3.89344259425137e-001"/><Con from="57" weight="-1.63793142368304e-001"/><Con from="58" weight="7.34949914077600e-001"/><Con from="59" weight="2.44955296751216e-002"/><Con from="60" weight="3.19302973936859e-001"/><Con from="61" weight="4.49008932241517e-001"/><Con from="62" weight="9.75437883916841e-002"/><Con from="63" weight="-3.34116788696065e-001"/><Con from="64" weight="1.71888852293481e+000"/><Con from="65" weight="3.76001802694741e-002"/><Con from="66" weight="3.88747440423029e-001"/><Con from="67" weight="-3.55626963645024e-001"/><Con from="68" weight="-1.00939512609927e-001"/><Con from="69" weight="-1.39197166267185e+000"/><Con from="70" weight="4.11075179923082e-001"/><Con from="71" weight="-8.93043510495335e-001"/><Con from="72" weight="2.59816483462714e-001"/><Con from="73" weight="-5.20328389937913e-001"/><Con from="74" weight="-5.18942966455472e-002"/><Con from="75" weight="2.51232461728351e-001"/><Con from="76" weight="-7.28975494031250e-001"/><Con from="77" weight="3.03281153843104e-001"/><Con from="78" weight="-1.14323422800797e+000"/><Con from="79" weight="-1.51453625731357e-001"/><Con from="80" weight="4.32365257255608e-002"/><Con from="81" weight="-1.77147890855286e-001"/><Con from="82" weight="1.45273242390489e+000"/><Con from="83" weight="-2.71456673976014e-001"/><Con from="84" weight="-1.26432515349653e-001"/><Con from="85" weight="-5.17888983657990e-002"/><Con from="86" weight="6.72836193748492e-002"/><Con from="87" weight="-5.21265868685710e-001"/><Con from="88" weight="-2.63170025397647e-001"/><Con from="89" weight="3.51331013874628e-001"/><Con from="90" weight="-4.04083564363921e-001"/><Con from="91" weight="-3.21274952209915e-001"/><Con from="92" weight="-3.24052377430833e-001"/><Con from="93" weight="8.62229440583142e-001"/><Con from="94" weight="-3.68313018052389e-001"/><Con from="95" weight="-1.94966992942667e-001"/><Con from="96" weight="4.27836837512044e-001"/><Con from="97" weight="1.23996175523515e+000"/><Con from="98" weight="9.94689932949570e-001"/><Con from="99" weight="-3.10869823185533e-002"/></Neuron><Neuron id="120" bias="-6.24728795782321e-001"><Con from="0" weight="2.03672756964702e-001"/><Con from="1" weight="-2.28944507363533e-001"/><Con from="2" weight="2.64579714803127e-001"/><Con from="3" weight="-1.07381252640577e+000"/><Con from="4" weight="-1.38368055865696e-001"/><Con from="5" weight="-6.81423637345916e-001"/><Con from="6" weight="4.22831940232542e-001"/><Con from="7" weight="4.06574432968801e-001"/><Con from="8" weight="1.63198851844167e-002"/><Con from="9" weight="2.57252980277057e-001"/><Con from="10" weight="-1.55123657247033e-001"/><Con from="11" weight="-7.14418822436596e-001"/><Con from="12" weight="-7.75377979088781e-001"/><Con from="13" weight="-8.62345689290611e-001"/><Con from="14" weight="4.42440288330677e-001"/><Con from="15" weight="-5.08242172487088e-001"/><Con from="16" weight="2.73752376799040e-001"/><Con from="17" weight="6.49945404417969e-001"/><Con from="18" weight="3.22643000185676e-002"/><Con from="19" weight="-4.11896917779485e-001"/><Con from="20" weight="-9.99202018456976e-001"/><Con from="21" weight="3.71315910886904e-001"/><Con from="22" weight="1.05523791389292e-001"/><Con from="23" weight="-8.19611458926183e-001"/><Con from="24" weight="-2.53313543295168e-001"/><Con from="25" weight="-2.95748565912007e-001"/><Con from="26" weight="-6.27196470703932e-001"/><Con from="27" weight="-1.83447341512461e-001"/><Con from="28" weight="1.06016015323966e+000"/><Con from="29" weight="-3.16657145359331e-001"/><Con from="30" weight="-4.57944113412637e-001"/><Con from="31" weight="1.93425800138573e-002"/><Con from="32" weight="8.77198646142207e-001"/><Con from="33" weight="2.91852879319674e-001"/><Con from="34" weight="-3.37130741654298e-001"/><Con from="35" weight="-4.03994738163008e-001"/><Con from="36" weight="3.37194980244326e-003"/><Con from="37" weight="1.66773011740200e-001"/><Con from="38" weight="-1.11524665386342e-001"/><Con from="39" weight="4.54685474109237e-001"/><Con from="40" weight="-4.68600171117325e-001"/><Con from="41" weight="-4.38626496663774e-001"/><Con from="42" weight="1.73690253183118e-001"/><Con from="43" weight="1.58876068454061e-002"/><Con from="44" weight="5.86450453918695e-001"/><Con from="45" weight="2.40860694119859e-001"/><Con from="46" weight="-3.82792725020869e-001"/><Con from="47" weight="7.05725507069081e-002"/><Con from="48" weight="-2.02823026502909e-002"/><Con from="49" weight="-1.06734846013939e+000"/><Con from="50" weight="-4.98417543390727e-001"/><Con from="51" weight="-2.48198315991603e-001"/><Con from="52" weight="3.01681419713603e-001"/><Con from="53" weight="1.78997372448903e-001"/><Con from="54" weight="-1.10052478128379e-001"/><Con from="55" weight="5.47653107134221e-001"/><Con from="56" weight="-9.48266625803757e-001"/><Con from="57" weight="-4.17984372475560e-001"/><Con from="58" weight="3.70198341377156e-001"/><Con from="59" weight="-1.10439316792489e+000"/><Con from="60" weight="3.58349444943231e-001"/><Con from="61" weight="-8.43779282066794e-001"/><Con from="62" weight="-9.34707295778118e-001"/><Con from="63" weight="6.15850583352515e-001"/><Con from="64" weight="4.58230735778961e-001"/><Con from="65" weight="-3.85096332333607e-001"/><Con from="66" weight="4.40151000592757e-002"/><Con from="67" weight="2.62288532777326e-002"/><Con from="68" weight="-1.06280939965008e+000"/><Con from="69" weight="5.50546538056403e-002"/><Con from="70" weight="8.22526540531828e-002"/><Con from="71" weight="1.69681498132114e-001"/><Con from="72" weight="6.68449935939125e-002"/><Con from="73" weight="2.70334272002974e-001"/><Con from="74" weight="4.05875734129905e-001"/><Con from="75" weight="3.07419370550408e-001"/><Con from="76" weight="5.84856409596956e-001"/><Con from="77" weight="5.30007424476939e-001"/><Con from="78" weight="-8.92388174778568e-002"/><Con from="79" weight="3.19872981145886e-001"/><Con from="80" weight="1.16721351410446e-001"/><Con from="81" weight="1.87313576802683e-001"/><Con from="82" weight="-5.62185045209117e-001"/><Con from="83" weight="-5.60172002425012e-001"/><Con from="84" weight="3.72328479756724e-001"/><Con from="85" weight="4.62025266205795e-001"/><Con from="86" weight="-1.36511910402264e-001"/><Con from="87" weight="2.73575651572948e-001"/><Con from="88" weight="5.52563006086390e-001"/><Con from="89" weight="2.24655320423974e-001"/><Con from="90" weight="8.08850402697290e-003"/><Con from="91" weight="4.75889706662852e-001"/><Con from="92" weight="4.71419936217901e-003"/><Con from="93" weight="-6.92892718489907e-001"/><Con from="94" weight="-9.22688326925037e-001"/><Con from="95" weight="-1.34275135434131e+000"/><Con from="96" weight="-9.69792384908169e-002"/><Con from="97" weight="-6.18302211937995e-001"/><Con from="98" weight="-2.97924259968895e-001"/><Con from="99" weight="1.74622476658678e-001"/></Neuron><Neuron id="121" bias="-2.46383860129373e-001"><Con from="0" weight="-4.69681220986668e-001"/><Con from="1" weight="5.70068738751863e-002"/><Con from="2" weight="-5.37556313843852e-001"/><Con from="3" weight="-5.30090980438183e-001"/><Con from="4" weight="1.22160688210522e-001"/><Con from="5" weight="1.03557180895818e-001"/><Con from="6" weight="-1.34258281243535e+000"/><Con from="7" weight="3.55719618100210e-001"/><Con from="8" weight="-2.35232380418294e+000"/><Con from="9" weight="-1.10141856218569e+000"/><Con from="10" weight="8.12219664770083e-001"/><Con from="11" weight="-9.35904282849064e-001"/><Con from="12" weight="4.79980911739286e-001"/><Con from="13" weight="2.45049372792440e+000"/><Con from="14" weight="1.15977718669936e-001"/><Con from="15" weight="-2.66402068853112e-001"/><Con from="16" weight="-2.67087651354881e-001"/><Con from="17" weight="6.33375062666536e-001"/><Con from="18" weight="-2.48591587222212e+000"/><Con from="19" weight="1.40250270864048e+000"/><Con from="20" weight="-1.63799826582562e+000"/><Con from="21" weight="9.16945557319874e-001"/><Con from="22" weight="2.04335679503218e+000"/><Con from="23" weight="-6.17120680188270e-001"/><Con from="24" weight="1.24805059975423e-001"/><Con from="25" weight="8.97340251590768e-001"/><Con from="26" weight="-1.95038252626820e+000"/><Con from="27" weight="5.97378311288001e-001"/><Con from="28" weight="3.68733108398399e-001"/><Con from="29" weight="1.86828584681078e-001"/><Con from="30" weight="3.61236759548923e-001"/><Con from="31" weight="-1.21553754585150e+000"/><Con from="32" weight="1.24952563146308e+000"/><Con from="33" weight="-1.92571587891445e+000"/><Con from="34" weight="-2.20579783943625e-001"/><Con from="35" weight="-8.71295626176157e-001"/><Con from="36" weight="-6.58339773607641e-001"/><Con from="37" weight="-1.44198812548628e-001"/><Con from="38" weight="-4.87406555471794e-001"/><Con from="39" weight="-5.12090452615188e-001"/><Con from="40" weight="-1.59929211600265e+000"/><Con from="41" weight="3.60181601001565e-001"/><Con from="42" weight="1.43256334304512e+000"/><Con from="43" weight="1.01906353440349e-001"/><Con from="44" weight="2.44999194916493e-001"/><Con from="45" weight="4.84199606679223e-001"/><Con from="46" weight="-3.02231677812216e-001"/><Con from="47" weight="1.10364001452802e+000"/><Con from="48" weight="-8.02334976460223e-001"/><Con from="49" weight="1.31349350682293e+000"/><Con from="50" weight="-4.34493365050062e-001"/><Con from="51" weight="7.10220717077864e-001"/><Con from="52" weight="-1.85078934639089e+000"/><Con from="53" weight="4.41454578301135e-001"/><Con from="54" weight="-3.98174028471738e-001"/><Con from="55" weight="-2.51418476507304e-001"/><Con from="56" weight="-5.04141231092224e-001"/><Con from="57" weight="8.74428606027207e-001"/><Con from="58" weight="6.69735850769210e-001"/><Con from="59" weight="4.52143648463090e-001"/><Con from="60" weight="-2.49429103396796e-001"/><Con from="61" weight="4.31992350944368e-001"/><Con from="62" weight="-7.83274088013461e-001"/><Con from="63" weight="2.23003666654750e-001"/><Con from="64" weight="-4.04698319108294e-001"/><Con from="65" weight="-3.55231829755090e-001"/><Con from="66" weight="1.89632476523805e+000"/><Con from="67" weight="-5.92948923800893e-002"/><Con from="68" weight="3.98335072002250e-001"/><Con from="69" weight="-1.03754996104778e+000"/><Con from="70" weight="-8.46540947358701e-002"/><Con from="71" weight="-1.27564021692265e+000"/><Con from="72" weight="2.14465319916909e+000"/><Con from="73" weight="9.54575023478575e-002"/><Con from="74" weight="-6.72345111544927e-001"/><Con from="75" weight="5.75107691729170e-001"/><Con from="76" weight="1.25031866200983e+000"/><Con from="77" weight="2.83683413760143e-001"/><Con from="78" weight="1.89814813452391e-001"/><Con from="79" weight="1.54231266674909e-001"/><Con from="80" weight="6.80331898286017e-001"/><Con from="81" weight="2.05639107433576e+000"/><Con from="82" weight="-6.77780629633904e-001"/><Con from="83" weight="1.43434368033249e-001"/><Con from="84" weight="2.05092759818773e-001"/><Con from="85" weight="-5.59078590798207e-001"/><Con from="86" weight="-9.11622151110556e-001"/><Con from="87" weight="-1.70995230624014e-001"/><Con from="88" weight="-4.70737203484123e-001"/><Con from="89" weight="-7.50263745361718e-001"/><Con from="90" weight="1.49668399763525e+000"/><Con from="91" weight="-3.37856845929162e-001"/><Con from="92" weight="-9.49849517449558e-001"/><Con from="93" weight="2.79916751968850e-001"/><Con from="94" weight="-4.50949076740387e-001"/><Con from="95" weight="2.11399162622364e-001"/><Con from="96" weight="-1.69087867161460e-001"/><Con from="97" weight="5.96516954829906e-001"/><Con from="98" weight="-1.56226195232641e+000"/><Con from="99" weight="-2.79507856969658e-001"/></Neuron><Neuron id="122" bias="-2.22737956628993e-001"><Con from="0" weight="2.20828263085057e-001"/><Con from="1" weight="1.50485307271682e-001"/><Con from="2" weight="-1.29225233716897e-001"/><Con from="3" weight="-4.98283378910344e-001"/><Con from="4" weight="1.88188395322039e-001"/><Con from="5" weight="-1.49411031374113e-001"/><Con from="6" weight="-1.51951273819055e-001"/><Con from="7" weight="-3.06494803233055e-001"/><Con from="8" weight="-1.02197556639152e-001"/><Con from="9" weight="4.17885474099887e-001"/><Con from="10" weight="-4.76937748590399e-001"/><Con from="11" weight="-1.78004313913108e-001"/><Con from="12" weight="-2.73638218706632e-001"/><Con from="13" weight="-4.38155384168605e-001"/><Con from="14" weight="-3.99609815765564e-001"/><Con from="15" weight="-1.96419503879219e-001"/><Con from="16" weight="-6.75637969472010e-002"/><Con from="17" weight="-1.14848457231710e-001"/><Con from="18" weight="1.48667118099519e-001"/><Con from="19" weight="9.53994680608048e-002"/><Con from="20" weight="6.74027517782687e-001"/><Con from="21" weight="-5.35039891418865e-001"/><Con from="22" weight="-2.16972565678864e-001"/><Con from="23" weight="4.88244045804160e-001"/><Con from="24" weight="-4.71703791125851e-002"/><Con from="25" weight="5.89014335379094e-001"/><Con from="26" weight="6.84118261881151e-002"/><Con from="27" weight="-4.55905907947249e-001"/><Con from="28" weight="3.22931287262287e-001"/><Con from="29" weight="-6.10977007739149e-001"/><Con from="30" weight="1.41848924255710e-001"/><Con from="31" weight="-1.07773233839628e-001"/><Con from="32" weight="5.23279523542995e-001"/><Con from="33" weight="-4.28968253932483e-002"/><Con from="34" weight="-5.05675961007927e-001"/><Con from="35" weight="-4.53703728129139e-002"/><Con from="36" weight="2.60148631060190e-001"/><Con from="37" weight="-3.48369005823012e-001"/><Con from="38" weight="3.59256297444783e-001"/><Con from="39" weight="-1.25777535453009e-001"/><Con from="40" weight="7.47464478497776e-001"/><Con from="41" weight="3.72178172025894e-001"/><Con from="42" weight="-1.37402284628175e-001"/><Con from="43" weight="1.24439056285211e-001"/><Con from="44" weight="4.16466271183537e-001"/><Con from="45" weight="-3.68146367709654e-001"/><Con from="46" weight="2.84249433692491e-001"/><Con from="47" weight="6.02739281037991e-001"/><Con from="48" weight="-5.22516178816684e-002"/><Con from="49" weight="2.12641490250264e-001"/><Con from="50" weight="-1.40236824200728e-001"/><Con from="51" weight="-7.75946955778447e-001"/><Con from="52" weight="3.49375139976859e-001"/><Con from="53" weight="-7.48090286184077e-001"/><Con from="54" weight="3.46907453946486e-001"/><Con from="55" weight="-1.22209815090987e-001"/><Con from="56" weight="4.11250144295855e-001"/><Con from="57" weight="2.70786496315759e-001"/><Con from="58" weight="2.76514772463024e-001"/><Con from="59" weight="-6.73335778206173e-001"/><Con from="60" weight="5.00882155734931e-001"/><Con from="61" weight="-3.77868811279690e-001"/><Con from="62" weight="7.85947709918139e-001"/><Con from="63" weight="-1.72533869219393e-001"/><Con from="64" weight="5.34739606323033e-001"/><Con from="65" weight="2.61206481318218e-001"/><Con from="66" weight="8.62529211680479e-001"/><Con from="67" weight="5.55503687500337e-002"/><Con from="68" weight="2.88107073806308e-001"/><Con from="69" weight="-5.48311374895221e-001"/><Con from="70" weight="8.73845748924477e-002"/><Con from="71" weight="6.77339758650822e-002"/><Con from="72" weight="2.60095298589493e-001"/><Con from="73" weight="1.88051337719918e-001"/><Con from="74" weight="-5.07180340905813e-002"/><Con from="75" weight="5.77745479507674e-002"/><Con from="76" weight="-6.30675040138971e-001"/><Con from="77" weight="-6.22680762278959e-002"/><Con from="78" weight="7.95969177145986e-002"/><Con from="79" weight="-2.55173492007485e-001"/><Con from="80" weight="-1.78479249944521e-001"/><Con from="81" weight="-4.51544031568019e-001"/><Con from="82" weight="4.06190843845382e-001"/><Con from="83" weight="2.83913914440603e-001"/><Con from="84" weight="7.74482380076603e-002"/><Con from="85" weight="4.21677358192019e-001"/><Con from="86" weight="1.30371483716372e-001"/><Con from="87" weight="-1.85825526370428e-001"/><Con from="88" weight="1.18992173612899e-001"/><Con from="89" weight="-7.62844678003526e-002"/><Con from="90" weight="-1.94874054202423e-001"/><Con from="91" weight="-6.67317299420621e-002"/><Con from="92" weight="1.58747700050785e-001"/><Con from="93" weight="-1.98946153157516e-001"/><Con from="94" weight="-1.10408825827569e-001"/><Con from="95" weight="-6.65233177565899e-001"/><Con from="96" weight="6.15446060665364e-001"/><Con from="97" weight="5.18510991191934e-001"/><Con from="98" weight="-1.74267187286293e-002"/><Con from="99" weight="2.56523368550998e-001"/></Neuron></NeuralLayer><NeuralLayer numberOfNeurons="2" activationFunction="exponential"><Neuron id="123" bias="1.28019964984233e-001"><Con from="100" weight="-1.59727127303942e-001"/><Con from="101" weight="2.28591850163921e-001"/><Con from="102" weight="1.63747144235991e+000"/><Con from="103" weight="-6.79258896725065e-001"/><Con from="104" weight="-5.47211869068751e-001"/><Con from="105" weight="1.28536449300373e+000"/><Con from="106" weight="5.88484748865727e-001"/><Con from="107" weight="1.66318514264877e-001"/><Con from="108" weight="-1.13033311558108e-001"/><Con from="109" weight="7.20941275255676e-002"/><Con from="110" weight="5.07050641854632e-001"/><Con from="111" weight="-1.90982492968102e-001"/><Con from="112" weight="-1.79125271052625e+000"/><Con from="113" weight="2.02698971435018e-001"/><Con from="114" weight="-1.50153440041037e-001"/><Con from="115" weight="6.94986757043122e-002"/><Con from="116" weight="-2.20621648768413e-002"/><Con from="117" weight="4.50702396178857e-001"/><Con from="118" weight="7.24733348951659e-002"/><Con from="119" weight="-1.48030161373158e+000"/><Con from="120" weight="-3.27021023815682e-002"/><Con from="121" weight="-3.21720613399997e-001"/><Con from="122" weight="-2.76957149811139e-001"/></Neuron><Neuron id="124" bias="1.74761540164572e-001"><Con from="100" weight="3.47599550723229e+000"/><Con from="101" weight="4.89675166941240e-003"/><Con from="102" weight="-3.63490038758269e-002"/><Con from="103" weight="4.21571449610788e-003"/><Con from="104" weight="9.00178343073293e-003"/><Con from="105" weight="-5.40756905836066e-002"/><Con from="106" weight="9.60985841333196e-003"/><Con from="107" weight="-7.41036146951153e-001"/><Con from="108" weight="1.61179938612841e+000"/><Con from="109" weight="-4.63037030278124e-001"/><Con from="110" weight="1.02664751554759e-001"/><Con from="111" weight="-2.20044551251242e-001"/><Con from="112" weight="-5.32651024773179e-003"/><Con from="113" weight="-3.85990265011036e-001"/><Con from="114" weight="3.04029608402191e-002"/><Con from="115" weight="2.27444732643197e-002"/><Con from="116" weight="-2.62364205394164e+000"/><Con from="117" weight="-4.32259129982013e-001"/><Con from="118" weight="-6.80564040513155e-001"/><Con from="119" weight="-7.02640800999147e-001"/><Con from="120" weight="-1.96669154355356e-001"/><Con from="121" weight="3.25070704262197e-001"/><Con from="122" weight="2.77082110839301e-001"/></Neuron></NeuralLayer><NeuralOutputs numberOfOutputs="2"><NeuralOutput outputNeuron="123"><DerivedField optype="categorical"><NormDiscrete field="[M] class" value="1"/></DerivedField></NeuralOutput><NeuralOutput outputNeuron="124"><DerivedField optype="categorical"><NormDiscrete field="[M] class" value="2"/></DerivedField></NeuralOutput></NeuralOutputs></NeuralNetwork></PMML>

## 1.3. MLP 100-15-2

<?xml version="1.0" encoding="UTF-8"?>
[truncated: 1,196,908 more chars]
