# Supplementary material for: The use of fast molecular descriptors and artificial neural networks approach in organochlorine compounds electron ionization mass spectra classification
Source: Environ Sci Pollut Res Int. 2019 Jul 30;26(27):28188–201. doi: 10.1007/s11356-019-05968-4 (PMC6791912; doi:10.1007/s11356-019-05968-4)
Supplement: Supplementary file 3 — (DOCX 39 kb) [file 11356_2019_5968_MOESM3_ESM.docx]

**Supplementary Material S3**

**The use of fast molecular descriptors and artificial neural networks approach** **in organochlorine compounds electron ionization mass spectra classification**

Maciej Przybyłek^a^, Waldemar Studziński^b^, Alicja Gackowska^b^ and Jerzy Gaca^b^

^a^*Chair and Department of Physical Chemistry, Pharmacy Faculty, Collegium Medicum of Bydgoszcz, Nicolaus Copernicus University in Toruń, Kurpińskiego 5, 85-950 Bydgoszcz, Poland,*

^b^*Faculty of Chemical Technology and Engineering, University of Technology and Life Science, Seminaryjna 3, 85-326 Bydgoszcz, Poland*

Table of Contents

[1. Sensitivity analysis for [M] peaks classification models (criterion I) 2](#_Toc529486284)

[2. Sensitivity analysis for [M-35] peaks classification models (criterion II) 5](#_Toc529486285)

# 1. Sensitivity analysis for [M] peaks classification models (criterion I)

**Table S2** List of applied molecular descriptors along with the results of sensitivity analysis performed for created [M] peaks classification models (criterion I). Error values are expressed as cross entropy.

| **Descriptor** | **Error** | | | | | | **Rank** |
| --- | --- | --- | --- | --- | --- | --- | --- |
|  | **MLP**  **100-19-2** | **MLP**  **100-23-2** | **MLP**  **100-15-2** | **MLP**  **100-25-2** | **MLP**  **100-21-2** | **Mean** |  |
| minaasC | 3.14•10^11^ | 1.0694 | 1.0470 | 1.0139 | 1.0184 | 6.28•10^10^ | 1 |
| nsssN | 1.0043 | 5.92•10^7^ | 1.0923 | 1.2983 | 1.1186 | 1.18•10^7^ | 2 |
| maxdO | 0.0040 | 5.17•10^6^ | 1.3198 | 1.2220 | 1.1477 | 1.03•10^6^ | 3 |
| C2SP2 | 1.04•10^5^ | 1.1500 | 1.0884 | 1.0851 | 1.0885 | 2.07•10^4^ | 4 |
| piPC8 | 0.0001 | 4.31•10^4^ | 1.1923 | 1.2692 | 1.2409 | 8627.8722 | 5 |
| piPC9 | 2.85•10^4^ | 3.9590 | 1.1990 | 1.2186 | 1.1542 | 5699.7545 | 6 |
| maxaasC | 1.11•10^4^ | 1.1299 | 1.9081 | 1.2159 | 2.2037 | 2216.4674 | 7 |
| maxsssCH | 1349.3481 | 1.2655 | 1.2599 | 1.0282 | 1.1324 | 270.8068 | 8 |
| maxaaCH | 22.0657 | 1309.7658 | 1.2534 | 1.3347 | 1.2555 | 267.1350 | 9 |
| minaaCH | 37.4814 | 1061.4823 | 1.2984 | 1.1892 | 1.2388 | 220.5380 | 10 |
| mindO | 0.0030 | 279.0569 | 1.5769 | 1.6368 | 1.2678 | 56.7083 | 11 |
| nAtomP | 255.2547 | 2.0460 | 1.2269 | 1.7502 | 1.3450 | 52.3246 | 12 |
| n6Ring | 1.2204 | 122.4817 | 1.1309 | 1.1186 | 1.1012 | 25.4106 | 13 |
| ETA_Shape_P | 84.6648 | 1.4254 | 1.0747 | 1.0547 | 1.0196 | 17.8478 | 14 |
| piPC10 | 46.9719 | 33.9554 | 1.5425 | 1.8350 | 1.6230 | 17.1856 | 15 |
| BCUTc-1h | 0.0026 | 69.1073 | 1.2594 | 1.2546 | 1.2576 | 14.5763 | 16 |
| AATS6i | 0.0006 | 35.3533 | 1.1263 | 1.0868 | 1.1529 | 7.7440 | 17 |
| FMF | 0.0008 | 29.5380 | 1.1291 | 1.4347 | 1.2917 | 6.6789 | 18 |
| ATSC0m | 0.0153 | 26.0519 | 1.6636 | 1.9373 | 1.3265 | 6.1989 | 19 |
| MLFER_S | 24.3525 | 1.9584 | 1.1076 | 1.0674 | 1.0698 | 5.9111 | 20 |
| BCUTw-1l | 23.5955 | 1.3205 | 1.1206 | 1.0948 | 1.1293 | 5.6521 | 21 |
| VE1_Dt | 22.6377 | 1.4533 | 1.2651 | 1.0347 | 1.1318 | 5.5045 | 22 |
| MAXDP2 | 0.0311 | 22.2254 | 1.4122 | 2.3721 | 1.2372 | 5.4556 | 23 |
| minwHBa | 13.4531 | 5.7694 | 1.1746 | 1.2351 | 1.1543 | 4.5573 | 24 |
| maxHBd | 11.1643 | 0.8650 | 1.1053 | 1.1012 | 1.2145 | 3.0901 | 25 |
| maxHCsats | 5.9516 | 5.5515 | 1.1561 | 1.4258 | 1.2576 | 3.0685 | 26 |
| AATS7i | 6.7704 | 3.9879 | 1.0680 | 1.1538 | 1.1119 | 2.8184 | 27 |
| GGI6 | 8.6878 | 1.9349 | 1.1026 | 1.0489 | 1.0855 | 2.7719 | 28 |
| C1SP2 | 8.5303 | 1.6330 | 1.0502 | 1.0531 | 1.0837 | 2.6701 | 29 |
| minHother | 0.0000 | 9.1428 | 1.2914 | 1.3020 | 1.0512 | 2.5575 | 30 |
| RotBFrac | 0.1218 | 6.6532 | 1.2739 | 1.6865 | 1.1984 | 2.1868 | 31 |
| C1SP3 | 6.2157 | 0.8069 | 1.0523 | 1.1748 | 1.0755 | 2.0650 | 32 |
| BCUTc-1l | 0.9598 | 5.9667 | 1.0115 | 1.0389 | 1.0262 | 2.0006 | 33 |
| minsssN | 5.5780 | 1.2159 | 1.0737 | 1.0372 | 1.0377 | 1.9885 | 34 |
| piPC6 | 0.0000 | 5.1968 | 1.0953 | 1.2409 | 1.0964 | 1.7259 | 35 |
| LipinskiFailures | 3.8560 | 1.2544 | 1.1062 | 1.1013 | 1.0805 | 1.6797 | 36 |
| maxHssNH | 4.1171 | 1.0629 | 1.0123 | 1.0106 | 1.0680 | 1.6542 | 37 |
| AATSC0i | 0.6514 | 4.0727 | 1.1291 | 1.2009 | 1.1960 | 1.6500 | 38 |
| nHBAcc | 3.9812 | 1.0198 | 1.0616 | 1.0391 | 1.0410 | 1.6285 | 39 |
| minHBd | 3.6396 | 0.7453 | 1.3027 | 1.0704 | 1.3222 | 1.6160 | 40 |
| AATSC0e | 3.4679 | 1.1126 | 1.0841 | 1.1088 | 1.1112 | 1.5769 | 41 |
| maxsssN | 2.8123 | 1.6134 | 1.0758 | 1.2511 | 1.0329 | 1.5571 | 42 |
| maxHCsatu | 3.5249 | 0.8127 | 1.0627 | 1.1845 | 1.0686 | 1.5307 | 43 |
| maxssO | 0.0000 | 3.2206 | 1.5114 | 1.4879 | 1.2771 | 1.4994 | 44 |
| MLFER_E | 0.0268 | 3.8054 | 1.0518 | 1.3274 | 1.0364 | 1.4496 | 45 |
| minssO | 0.0000 | 3.6732 | 1.1914 | 1.1607 | 1.1459 | 1.4342 | 46 |
| minHssNH | 2.8737 | 1.0549 | 1.0111 | 1.0302 | 1.0648 | 1.4069 | 47 |
| gmax | 1.3805 | 1.3257 | 1.5806 | 1.1855 | 1.4762 | 1.3897 | 48 |
| maxHBa | 0.3433 | 2.0608 | 1.0597 | 2.1801 | 1.1598 | 1.3607 | 49 |
| piPC4 | 0.0000 | 2.9937 | 1.4033 | 1.1183 | 1.1646 | 1.3360 | 50 |
| WTPT-5 | 2.5931 | 1.0468 | 0.9989 | 0.9856 | 0.9980 | 1.3245 | 51 |
| nHCsatu | 2.1549 | 1.1330 | 1.0285 | 1.1447 | 1.0265 | 1.2975 | 52 |
| nN | 2.2576 | 1.1615 | 1.0013 | 0.9876 | 0.9988 | 1.2814 | 53 |
| minHBint2 | 1.5407 | 1.1645 | 1.2464 | 1.1973 | 1.1445 | 1.2587 | 54 |
| GATS2s | 1.8723 | 0.9658 | 1.0201 | 1.1121 | 1.0235 | 1.1988 | 55 |
| AATS8i | 0.3364 | 1.7287 | 1.3371 | 1.1894 | 1.2757 | 1.1735 | 56 |
| JGT | 0.0000 | 2.2653 | 1.2213 | 1.0474 | 1.2335 | 1.1535 | 57 |
| minHCsatu | 0.1397 | 2.0719 | 1.1877 | 1.2085 | 1.1591 | 1.1534 | 58 |
| nAtomLAC | 0.5894 | 1.2636 | 1.3365 | 1.2355 | 1.3308 | 1.1512 | 59 |
| minsNH2 | 1.6166 | 0.9976 | 1.0533 | 1.0066 | 1.0300 | 1.1408 | 60 |
| maxsNH2 | 1.5836 | 0.9899 | 1.0507 | 0.9959 | 1.0266 | 1.1293 | 61 |
| GATS1c | 0.0127 | 2.3630 | 1.0657 | 1.0152 | 1.0305 | 1.0974 | 62 |
| GGI8 | 1.3059 | 0.6385 | 1.2456 | 1.1148 | 1.1805 | 1.0970 | 63 |
| MATS1c | 0.0082 | 2.1940 | 1.0749 | 1.0628 | 1.1386 | 1.0957 | 64 |
| maxsCl | 1.0200 | 0.9672 | 1.2345 | 1.0793 | 1.1180 | 1.0838 | 65 |
| VR2_Dzs | 1.3954 | 0.9416 | 1.0125 | 1.0130 | 1.0075 | 1.0740 | 66 |
| C4SP3 | 1.2150 | 1.0514 | 1.0390 | 1.0370 | 1.0215 | 1.0728 | 67 |
| MDEO-12 | 0.5711 | 1.2620 | 1.0708 | 1.4343 | 1.0038 | 1.0684 | 68 |
| ETA_BetaP | 0.1859 | 1.8237 | 1.0761 | 1.1779 | 1.0783 | 1.0684 | 69 |
| SHCsatu | 0.7146 | 1.3624 | 1.0364 | 1.0986 | 1.0325 | 1.0489 | 70 |
| RotBtFrac | 1.0791 | 0.7325 | 1.0554 | 1.3299 | 1.0401 | 1.0474 | 71 |
| maxHBint2 | 0.9576 | 1.1233 | 1.0542 | 1.0321 | 1.0343 | 1.0403 | 72 |
| ndS | 0.6946 | 1.1642 | 1.0534 | 1.0377 | 1.0528 | 1.0006 | 73 |
| maxHother | 0.0003 | 1.8469 | 1.0524 | 1.0294 | 1.0625 | 0.9983 | 74 |
| ATSC3c | 0.9451 | 0.9957 | 1.0087 | 1.0048 | 1.0052 | 0.9919 | 75 |
| nssS | 0.6430 | 0.9940 | 1.0794 | 1.0403 | 1.1708 | 0.9855 | 76 |
| maxssssC | 0.3744 | 1.0183 | 1.1497 | 1.1917 | 1.1799 | 0.9828 | 77 |
| ATSC3e | 0.6603 | 0.9806 | 1.0674 | 1.0903 | 1.0879 | 0.9773 | 78 |
| maxwHBa | 0.0015 | 1.5581 | 1.0770 | 1.0947 | 1.0935 | 0.9650 | 79 |
| nBase | 0.7053 | 1.0307 | 1.0244 | 1.0209 | 1.0046 | 0.9572 | 80 |
| MATS2s | 0.7197 | 0.9801 | 1.0071 | 1.0644 | 1.0102 | 0.9563 | 81 |
| ETA_BetaP_ns_d | 0.0431 | 1.1678 | 1.0878 | 1.3731 | 1.0787 | 0.9501 | 82 |
| GATS3c | 0.6418 | 0.9028 | 1.0817 | 1.0419 | 1.0646 | 0.9466 | 83 |
| ETA_BetaP_ns | 0.1115 | 0.7536 | 1.1551 | 1.5219 | 1.1536 | 0.9391 | 84 |
| GATS4e | 0.0000 | 1.1961 | 1.1827 | 1.0935 | 1.1557 | 0.9256 | 85 |
| SIC5 | 0.0000 | 1.1824 | 1.0307 | 1.2110 | 1.0554 | 0.8959 | 86 |
| nsCH3 | 0.0021 | 1.0848 | 1.0034 | 1.2183 | 1.0803 | 0.8778 | 87 |
| maxdssC | 0.0661 | 0.9227 | 1.1787 | 1.0915 | 1.1076 | 0.8733 | 88 |
| MAXDN2 | 0.0059 | 1.1369 | 1.0229 | 1.1814 | 0.9983 | 0.8691 | 89 |
| minHCsats | 0.2714 | 0.7227 | 1.0820 | 1.2049 | 1.0631 | 0.8688 | 90 |
| maxsCH3 | 0.0000 | 0.6364 | 1.2649 | 1.1789 | 1.2238 | 0.8608 | 91 |
| minssCH2 | 0.0000 | 1.1147 | 1.0459 | 1.0399 | 1.0297 | 0.8460 | 92 |
| GATS4s | 0.0347 | 0.8635 | 1.0810 | 1.0820 | 1.0891 | 0.8301 | 93 |
| GATS4i | 0.0000 | 1.0166 | 1.0293 | 1.0509 | 1.0395 | 0.8272 | 94 |
| piPC5 | 0.0000 | 0.7640 | 1.0208 | 1.2892 | 1.0501 | 0.8248 | 95 |
| C3SP2 | 0.1670 | 0.6839 | 1.0614 | 1.0657 | 1.0761 | 0.8108 | 96 |
| GATS3s | 0.0000 | 0.8005 | 1.0301 | 1.0049 | 1.0161 | 0.7703 | 97 |
| maxHdsCH | 0.0000 | 0.6476 | 1.0746 | 1.0173 | 1.0444 | 0.7568 | 98 |
| BIC5 | 0.0000 | 0.4420 | 1.0297 | 1.2416 | 1.0230 | 0.7472 | 99 |
| minHdsCH | 0.0000 | 0.5328 | 1.0485 | 1.0230 | 1.0125 | 0.7234 | 100 |

# 2. Sensitivity analysis for [M-35] peaks classification models (criterion II)

**Table S3** List of applied molecular descriptors along with the results of sensitivity analysis performed for created [M-35] peaks classification models (criterion II). Error values are expressed as cross entropy.

| **Descriptor** | **Error** | | | | | | **Rank** |
| --- | --- | --- | --- | --- | --- | --- | --- |
|  | **MLP**  **100-25-2** | **MLP**  **100-22-2**  **(BFGS 73)** | **MLP**  **100-22-2**  **(BFGS 72)** | **MLP**  **100-22-2**  **(BFGS 48)** | **MLP**  **100-24-2** | **Mean** |  |
| maxHaaCH | 1.2286 | 1.3790 | 44.9766 | 1.1496 | 1.2306 | 9.9929 | 1 |
| maxwHBd | 1.0733 | 1.0383 | 39.7840 | 1.0173 | 1.0320 | 8.7890 | 2 |
| maxHCHnX | 1.0724 | 1.0413 | 29.6273 | 1.0125 | 1.0295 | 6.7566 | 3 |
| VE1_Dzm | 1.0114 | 1.0502 | 12.7748 | 1.0045 | 1.0392 | 3.3760 | 4 |
| VE1_DzZ | 1.0111 | 1.0520 | 12.5535 | 1.0044 | 1.0399 | 3.3322 | 5 |
| nHCsatu | 1.0120 | 1.0586 | 10.4278 | 1.0466 | 1.0195 | 2.9129 | 6 |
| ETA_Shape_Y | 1.0731 | 1.4402 | 7.5190 | 1.0557 | 1.2068 | 2.4590 | 7 |
| GGI8 | 1.0745 | 1.1347 | 7.4509 | 1.0500 | 1.1511 | 2.3723 | 8 |
| minHCsats | 1.0282 | 1.2068 | 5.9874 | 1.0136 | 1.1261 | 2.0724 | 9 |
| nHBAcc | 1.0713 | 1.0357 | 5.7839 | 1.0136 | 1.0362 | 1.9881 | 10 |
| minsOH | 1.0624 | 1.0081 | 3.8933 | 1.0082 | 0.9884 | 1.5921 | 11 |
| minsCH3 | 1.3500 | 1.5873 | 1.2694 | 1.2873 | 2.1379 | 1.5264 | 12 |
| VE1_Dzv | 1.0047 | 1.0214 | 3.5748 | 1.0026 | 1.0069 | 1.5221 | 13 |
| maxsCH3 | 1.3387 | 1.6986 | 0.1581 | 1.2756 | 2.1541 | 1.3250 | 14 |
| LipinskiFailures | 1.0259 | 1.0408 | 2.4133 | 1.0151 | 1.0345 | 1.3059 | 15 |
| ATS8v | 1.0212 | 1.0244 | 2.1001 | 1.0045 | 1.0305 | 1.2361 | 16 |
| minHBint2 | 1.1277 | 1.1193 | 1.7477 | 1.0424 | 1.1038 | 1.2282 | 17 |
| VE1_Dzs | 1.0115 | 1.0630 | 1.9242 | 1.0053 | 1.0051 | 1.2018 | 18 |
| minssO | 1.1450 | 1.6878 | 0.5153 | 1.1453 | 1.4511 | 1.1889 | 19 |
| maxaaCH | 1.2638 | 1.2969 | 0.2784 | 1.2752 | 1.7157 | 1.1660 | 20 |
| GATS7i | 1.0209 | 1.0485 | 1.6698 | 1.0017 | 1.0153 | 1.1512 | 21 |
| piPC9 | 1.0416 | 1.2448 | 1.3078 | 1.0064 | 1.1335 | 1.1468 | 22 |
| piPC10 | 1.0679 | 1.1861 | 1.2504 | 1.0169 | 1.1834 | 1.1410 | 23 |
| FMF | 1.1162 | 1.2527 | 1.1448 | 1.0023 | 1.1199 | 1.1272 | 24 |
| maxssCH2 | 1.0337 | 1.4363 | 0.7864 | 1.0311 | 1.2918 | 1.1159 | 25 |
| maxaasC | 1.2141 | 1.5692 | 0.1400 | 1.1847 | 1.4076 | 1.1031 | 26 |
| nAtomLC | 1.0071 | 1.2969 | 1.0272 | 1.0043 | 1.1731 | 1.1017 | 27 |
| maxsOH | 1.0625 | 1.0117 | 1.4146 | 1.0093 | 0.9926 | 1.0981 | 28 |
| maxwHBa | 1.1612 | 1.1725 | 1.0216 | 1.0695 | 1.0472 | 1.0944 | 29 |
| minHother | 1.1581 | 1.3210 | 0.3757 | 1.1149 | 1.3895 | 1.0718 | 30 |
| SpMax2_Bhs | 1.0332 | 1.0378 | 1.2016 | 1.0421 | 1.0244 | 1.0678 | 31 |
| GGI10 | 1.0391 | 1.0142 | 1.2565 | 1.0111 | 1.0135 | 1.0669 | 32 |
| AATS7i | 1.0487 | 1.0918 | 1.1263 | 1.0092 | 1.0543 | 1.0661 | 33 |
| BCUTc-1h | 1.1240 | 1.0334 | 1.0578 | 1.0518 | 1.0485 | 1.0631 | 34 |
| GATS1c | 1.0061 | 1.0332 | 1.2157 | 1.0047 | 1.0492 | 1.0618 | 35 |
| GATS3s | 1.0221 | 1.0181 | 1.1662 | 1.0074 | 1.0086 | 1.0445 | 36 |
| minwHBa | 1.1414 | 1.0315 | 0.9817 | 1.0419 | 1.0210 | 1.0435 | 37 |
| RotBFrac | 1.1051 | 1.2169 | 0.6817 | 1.0366 | 1.1764 | 1.0433 | 38 |
| GATS8m | 1.0101 | 1.0062 | 1.1823 | 1.0038 | 1.0045 | 1.0414 | 39 |
| maxHCsats | 1.0252 | 1.4853 | 0.0943 | 1.0749 | 1.5102 | 1.0380 | 40 |
| GATS4m | 1.0487 | 1.0501 | 1.0550 | 1.0059 | 1.0295 | 1.0379 | 41 |
| SpMAD_Dzp | 1.0474 | 1.1529 | 0.8027 | 1.0684 | 1.1150 | 1.0373 | 42 |
| minHBd | 1.0420 | 1.0797 | 0.9440 | 1.0133 | 1.1042 | 1.0366 | 43 |
| maxHBd | 1.0250 | 1.1391 | 0.9619 | 1.0062 | 1.0403 | 1.0345 | 44 |
| MATS1s | 1.0190 | 1.0060 | 1.0709 | 1.0401 | 1.0343 | 1.0340 | 45 |
| C4SP3 | 1.0172 | 1.0094 | 1.0802 | 1.0105 | 1.0040 | 1.0242 | 46 |
| ATSC7v | 1.0280 | 1.0188 | 1.0622 | 1.0049 | 1.0043 | 1.0236 | 47 |
| AATS8p | 1.0191 | 1.0350 | 1.0128 | 1.0095 | 1.0200 | 1.0193 | 48 |
| SaaN | 1.0014 | 1.0073 | 1.0631 | 1.0016 | 1.0166 | 1.0180 | 49 |
| GATS4s | 1.0473 | 1.0100 | 0.9895 | 1.0216 | 1.0099 | 1.0156 | 50 |
| naaN | 1.0022 | 1.0094 | 1.0579 | 1.0018 | 1.0054 | 1.0153 | 51 |
| nT5HeteroRing | 1.0119 | 1.0022 | 1.0499 | 1.0030 | 0.9711 | 1.0076 | 52 |
| n5HeteroRing | 1.0118 | 1.0022 | 1.0458 | 1.0029 | 0.9710 | 1.0067 | 53 |
| AATS8m | 1.0009 | 1.0052 | 1.0125 | 1.0024 | 1.0057 | 1.0054 | 54 |
| nS | 1.0395 | 1.0363 | 0.8798 | 1.0118 | 1.0148 | 0.9964 | 55 |
| AATS8v | 1.0279 | 1.0191 | 0.8697 | 1.0095 | 1.0049 | 0.9862 | 56 |
| VE1_Dt | 1.1158 | 1.0555 | 0.6869 | 1.0416 | 1.0269 | 0.9854 | 57 |
| maxaaN | 1.0026 | 1.0123 | 0.8920 | 1.0042 | 1.0146 | 0.9851 | 58 |
| maxHsNH2 | 1.0084 | 1.0020 | 0.9047 | 1.0045 | 1.0042 | 0.9848 | 59 |
| ATSC6m | 1.0128 | 1.0017 | 0.9004 | 1.0019 | 1.0055 | 0.9845 | 60 |
| MATS1c | 1.0175 | 1.0687 | 0.7846 | 1.0038 | 1.0465 | 0.9842 | 61 |
| maxsNH2 | 1.0125 | 1.0067 | 0.8849 | 1.0025 | 0.9903 | 0.9794 | 62 |
| nHBint4 | 1.0228 | 1.0320 | 0.8392 | 1.0074 | 0.9885 | 0.9780 | 63 |
| GATS6i | 1.0051 | 1.0129 | 0.8537 | 1.0015 | 1.0070 | 0.9760 | 64 |
| minaaN | 1.0027 | 1.0024 | 0.8523 | 1.0029 | 1.0036 | 0.9728 | 65 |
| nHdsCH | 1.0269 | 1.0336 | 0.7515 | 1.0256 | 1.0186 | 0.9713 | 66 |
| C1SP2 | 1.1039 | 1.0341 | 0.6479 | 1.0234 | 1.0323 | 0.9683 | 67 |
| ndsCH | 1.0267 | 1.0324 | 0.7410 | 1.0252 | 1.0149 | 0.9681 | 68 |
| ETA_BetaP_ns_d | 1.0552 | 1.1944 | 0.4283 | 1.0261 | 1.1347 | 0.9678 | 69 |
| C2SP2 | 1.0576 | 1.2474 | 0.2997 | 1.0244 | 1.1460 | 0.9550 | 70 |
| C3SP2 | 1.0339 | 1.0329 | 0.6749 | 1.0161 | 1.0148 | 0.9545 | 71 |
| ETA_Beta_ns_d | 1.0079 | 1.0696 | 0.5671 | 1.0022 | 1.0685 | 0.9431 | 72 |
| AATS8i | 1.0553 | 1.1050 | 0.5113 | 1.0294 | 1.0133 | 0.9428 | 73 |
| maxssO | 1.1089 | 1.1780 | 0.1564 | 1.0415 | 1.2227 | 0.9415 | 74 |
| maxdO | 1.0929 | 1.1716 | 0.2167 | 1.0603 | 1.1600 | 0.9403 | 75 |
| mindO | 1.0902 | 1.1957 | 0.2173 | 1.0462 | 1.1489 | 0.9396 | 76 |
| maxsssN | 1.0179 | 1.0074 | 0.6644 | 1.0067 | 0.9985 | 0.9390 | 77 |
| SHdsCH | 1.0201 | 1.2678 | 0.3299 | 1.0412 | 1.0268 | 0.9372 | 78 |
| C1SP3 | 1.0186 | 1.0302 | 0.5906 | 1.0038 | 1.0336 | 0.9353 | 79 |
| minHCsatu | 1.0198 | 1.1328 | 0.4489 | 1.0059 | 1.0378 | 0.9290 | 80 |
| maxHCsatu | 1.0429 | 1.2548 | 0.1634 | 1.0895 | 1.0934 | 0.9288 | 81 |
| minssCH2 | 1.0644 | 1.0450 | 0.4165 | 1.0533 | 1.0143 | 0.9187 | 82 |
| piPC8 | 1.0567 | 1.1052 | 0.3907 | 1.0021 | 1.0238 | 0.9157 | 83 |
| AATS8e | 1.0029 | 1.1834 | 0.2613 | 1.0068 | 1.1037 | 0.9116 | 84 |
| MDEO-12 | 1.0134 | 1.1292 | 0.2950 | 1.0129 | 1.1012 | 0.9104 | 85 |
| mindssC | 1.0284 | 1.0042 | 0.4186 | 1.0041 | 1.0076 | 0.8926 | 86 |
| minHdsCH | 1.0607 | 1.0542 | 0.1117 | 1.0370 | 1.1254 | 0.8778 | 87 |
| minHCHnX | 1.0333 | 1.0430 | 0.2711 | 1.0095 | 1.0278 | 0.8769 | 88 |
| MAXDN2 | 1.0676 | 1.0515 | 0.1508 | 1.0168 | 1.0959 | 0.8765 | 89 |
| VE1_Dzp | 1.0195 | 1.0350 | 0.2957 | 1.0178 | 1.0142 | 0.8764 | 90 |
| minwHBd | 1.0328 | 1.0431 | 0.2503 | 1.0118 | 1.0267 | 0.8730 | 91 |
| BCUTw-1l | 1.0049 | 1.0112 | 0.2513 | 1.0029 | 1.0117 | 0.8564 | 92 |
| minsCl | 1.0138 | 1.0307 | 0.2159 | 0.9996 | 1.0046 | 0.8529 | 93 |
| ATS7m | 1.0133 | 1.0517 | 0.0976 | 1.0026 | 1.0769 | 0.8484 | 94 |
| minsssN | 1.0312 | 1.0243 | 0.1345 | 1.0098 | 1.0341 | 0.8468 | 95 |
| BCUTc-1l | 1.0101 | 1.0764 | 0.1260 | 1.0158 | 1.0048 | 0.8466 | 96 |
| GGI9 | 1.0144 | 1.0234 | 0.1522 | 1.0080 | 1.0040 | 0.8404 | 97 |
| MLFER_S | 1.0262 | 1.0323 | 0.0955 | 1.0143 | 1.0323 | 0.8401 | 98 |
| SHCsatu | 1.0044 | 1.0175 | 0.1253 | 1.0090 | 0.9974 | 0.8307 | 99 |
| MLFER_BO | 1.0198 | 1.0087 | 0.0999 | 1.0018 | 1.0062 | 0.8273 | 100 |
