# Supplementary material for: The use of fast molecular descriptors and artificial neural networks approach in organochlorine compounds electron ionization mass spectra classification
Source: Environ Sci Pollut Res Int. 2019 Jul 30;26(27):28188–201. doi: 10.1007/s11356-019-05968-4 (PMC6791912; doi:10.1007/s11356-019-05968-4)
Supplement: Supplementary file 4 — (DOCX 213 kb) [file 11356_2019_5968_MOESM4_ESM.docx]

**Supplementary Material S4**

**The use of fast molecular descriptors and artificial neural networks approach** **in organochlorine compounds electron ionization mass spectra classification**

Maciej Przybyłek^a^, Waldemar Studziński^b^, Alicja Gackowska^b^ and Jerzy Gaca^b^

^a^*Chair and Department of Physical Chemistry, Pharmacy Faculty, Collegium Medicum of Bydgoszcz, Nicolaus Copernicus University in Toruń, Kurpińskiego 5, 85-950 Bydgoszcz, Poland,*

^b^*Faculty of Chemical Technology and Engineering, University of Technology and Life Science, Seminaryjna 3, 85-326 Bydgoszcz, Poland*

Table of Contents

[1. Prediction of test set examples by the classification models 2](#_Toc530852084)

[1.1. Criterion I 2](#_Toc530852085)

[1.2. Criterion II 37](#_Toc530852086)

# 1. Prediction of test set examples by the classification models

## 1.1. Criterion I

**Table S4** The results of [M] peaks classification (criterion I) performed for the test set using MLP 100-19-2 (model 1), MLP 100-23-2 (model 2), MLP 100-15-2 (model 3), MLP 100-25-2 (model 4) and MLP 100-21-2 (model 5). Mass spectra data were obtained from NIST Chemistry WebBook database (http://webbook.nist.gov/chemistry/)

| **Name** | **IUPAC Standard InChIKey** | **SMILES** | **[M]** | **[M] class exp.** | **Model** | | | | |
| --- | --- | --- | --- | --- | --- | --- | --- | --- | --- |
|  |  |  |  |  | **1** | **2** | **3** | **4** | **5** |
| Ethyl Chloride (C2H5Cl) | HRYZWHHZPQKTII-UHFFFAOYSA-N | CCCl | 9999 | 1 | 1 | 1 | 1 | 1 | 1 |
| Thiophosgene (CCl2S) | ZWZVWGITAAIFPS-UHFFFAOYSA-N | C(=S)(Cl)Cl | 2150 | 1 | 1 | 1 | 1 | 1 | 1 |
| Acetaldehyde, chloro- (C2H3ClO) | QSKPIOLLBIHNAC-UHFFFAOYSA-N | C(C=O)Cl | 4040 | 1 | 1 | 1 | 1 | 1 | 1 |
| 1-Chloropropane (C3H7Cl) | SNMVRZFUUCLYTO-UHFFFAOYSA-N | CCCCl | 269 | 2 | 2 | 2 | 2 | 2 | 2 |
| 1-Chloropropene (C3H5Cl) | OWXJKYNZGFSVRC-UHFFFAOYSA-N | CC=CCl | 4249 | 1 | 1 | 1 | 1 | 1 | 1 |
| Methane, chloro- (CH3Cl) | NEHMKBQYUWJMIP-UHFFFAOYSA-N | CCl | 9999 | 1 | 1 | 1 | 1 | 1 | 1 |
| Carbonochloridothioic acid, S-methyl ester (C2H3ClOS) | YPSUCTSXOROPBS-UHFFFAOYSA-N | CSC(=O)Cl | 2400 | 1 | 2 | 1 | 2 | 1 | 1 |
| 1-Chlorobutane (C4H9Cl) | VFWCMGCRMGJXDK-UHFFFAOYSA-N | CCCCCl | 40 | 2 | 2 | 2 | 2 | 2 | 2 |
| 2-Propenenitrile, 2-chloro- (C3H2ClN) | OYUNTGBISCIYPW-UHFFFAOYSA-N | C=C(C#N)Cl | 9169 | 1 | 1 | 1 | 1 | 1 | 2 |
| 2-Propenoyl chloride (C3H3ClO) | HFBMWMNUJJDEQZ-UHFFFAOYSA-N | C=CC(=O)Cl | 50 | 2 | 2 | 2 | 2 | 2 | 2 |
| 1-Butene, 1-chloro-, (Z)- (C4H7Cl) | DUDKKPVINWLFBI-ARJAWSKDSA-N | CC/C=C\Cl | 2988 | 1 | 1 | 1 | 1 | 1 | 1 |
| Chloroprene (C4H5Cl) | YACLQRRMGMJLJV-UHFFFAOYSA-N | C=CC(=C)Cl | 4563 | 1 | 1 | 1 | 1 | 1 | 1 |
| Oxirane, (chloromethyl)-, (R)- (C3H5ClO) | BRLQWZUYTZBJKN-GSVOUGTGSA-N | C1[C@H](O1)CCl | 0 | 2 | 2 | 2 | 2 | 2 | 2 |
| Chloromethyl sulfonylchloride (CH2Cl2O2S) | KQDDQXNVESLJNO-UHFFFAOYSA-N | C(S(=O)(=O)Cl)Cl | 0 | 2 | 2 | 2 | 2 | 2 | 2 |
| 2-Chloro-N-methylacetamide (C3H6ClNO) | HOZLOOPIXHWKCI-UHFFFAOYSA-N | CNC(=O)CCl | 1926 | 1 | 1 | 1 | 1 | 1 | 2 |
| Oxalyl chloride (C2Cl2O2) | CTSLXHKWHWQRSH-UHFFFAOYSA-N | C(=O)(C(=O)Cl)Cl | 90 | 2 | 2 | 2 | 2 | 2 | 2 |
| Dichloroacetyl chloride (C2HCl3O) | FBCCMZVIWNDFMO-UHFFFAOYSA-N | C(C(=O)Cl)(Cl)Cl | 169 | 2 | 2 | 2 | 2 | 2 | 2 |
| 2,3-Dichloro-1-propanol (C3H6Cl2O) | ZXCYIJGIGSDJQQ-UHFFFAOYSA-N | C(C(CCl)Cl)O | 0 | 2 | 2 | 2 | 2 | 2 | 2 |
| 2-Butyne, 1,4-dichloro- (C4H4Cl2) | RCHDLEVSZBOHOS-UHFFFAOYSA-N | C(C#CCCl)Cl | 2369 | 1 | 1 | 1 | 1 | 1 | 1 |
| 2,3-Dichlorobutane (C4H8Cl2) | RMISVOPUIFJTEO-UHFFFAOYSA-N | CC(C(C)Cl)Cl | 179 | 2 | 2 | 2 | 2 | 2 | 2 |
| 2-Chloroethyl vinyl ether (C4H7ClO) | DNJRKFKAFWSXSE-UHFFFAOYSA-N | C=COCCCl | 2729 | 1 | 1 | 1 | 1 | 1 | 1 |
| 1-Pentene, 5-chloro- (C5H9Cl) | UPOBJNRMUDPATE-UHFFFAOYSA-N | C=CCCCCl | 1104 | 1 | 1 | 2 | 1 | 2 | 1 |
| 1-Chloro-3-methyl-2-butene (C5H9Cl) | JKXQKGNGJVZKFA-UHFFFAOYSA-N | CC(=CCCl)C | 3430 | 1 | 1 | 1 | 1 | 1 | 1 |
| 2-Chloro-3-methyl-1-butene (C5H9Cl) | RBSYGFLXVMWYGD-UHFFFAOYSA-N | CC(C)C(=C)Cl | 2068 | 1 | 1 | 1 | 1 | 1 | 1 |
| 2-Chloro-3-methyl-2-butene (C5H9Cl) | WIIKEBDPJPYJHF-UHFFFAOYSA-N | CC(=C(C)Cl)C | 4854 | 1 | 1 | 1 | 1 | 1 | 1 |
| 1H-1,2,4-Triazole, 3-chloro- (C2H2ClN3) | QGOUKZPSCTVYLX-UHFFFAOYSA-N | C1=NNC(=N1)Cl | 9562 | 1 | 1 | 1 | 1 | 1 | 1 |
| Cyclopentene, 1-chloro- (C5H7Cl) | UJUIJZWQFDQKHO-UHFFFAOYSA-N | C1CC=C(C1)Cl | 2450 | 1 | 1 | 1 | 1 | 1 | 1 |
| β-Methoxyethoxymethyl chloride (C4H9ClO2) | BIAAQBNMRITRDV-UHFFFAOYSA-N | COCCOCCl | 0 | 2 | 2 | 2 | 2 | 2 | 2 |
| Chloral Hydrate (C2H3Cl3O2) | RNFNDJAIBTYOQL-UHFFFAOYSA-N | C(C(Cl)(Cl)Cl)(O)O | 0 | 2 | 2 | 2 | 2 | 2 | 2 |
| Acetamide, 2,2,2-trichloro- (C2H2Cl3NO) | UPQQXPKAYZYUKO-UHFFFAOYSA-N | C(=O)(C(Cl)(Cl)Cl)N | 0 | 2 | 2 | 2 | 2 | 2 | 2 |
| Ethyl chloroacetate (C4H7ClO2) | VEUUMBGHMNQHGO-UHFFFAOYSA-N | CCOC(=O)CCl | 70 | 2 | 2 | 2 | 2 | 2 | 2 |
| Propane, 2-chloro-2-nitro- (C3H6ClNO2) | JQYFSFNSNVRUPY-UHFFFAOYSA-N | CC(C)([N+](=O)[O-])Cl | 0 | 2 | 2 | 2 | 2 | 2 | 2 |
| Bis(2-chloroethyl) sulphide (C4H8Cl2S) | QKSKPIVNLNLAAV-UHFFFAOYSA-N | C(CCl)SCCCl | 2382 | 1 | 1 | 1 | 1 | 1 | 1 |
| Bis(2-chloroethyl) ether (C4H8Cl2O) | ZNSMNVMLTJELDZ-UHFFFAOYSA-N | C(CCl)OCCCl | 126 | 2 | 2 | 2 | 2 | 2 | 2 |
| 2,3-dichloropropionyl chloride (C3H3Cl3O) | JQELECXPPAOSTM-UHFFFAOYSA-N | C(C(C(=O)Cl)Cl)Cl | 0 | 2 | 2 | 2 | 2 | 2 | 2 |
| Propane, 1,1,2,2-tetrachloro- (C3H4Cl4) | MDCBRXYTSHYYJE-UHFFFAOYSA-N | CC(C(Cl)Cl)(Cl)Cl | 20 | 2 | 2 | 2 | 2 | 2 | 2 |
| 5-Chlorovaleronitrile (C5H8ClN) | JSAWFGSXRPCFSW-UHFFFAOYSA-N | C(CCCl)CC#N | 3 | 2 | 2 | 2 | 1 | 2 | 2 |
| Butanoyl chloride, 3-methyl- (C5H9ClO) | ISULZYQDGYXDFW-UHFFFAOYSA-N | CC(C)CC(=O)Cl | 0 | 2 | 2 | 2 | 2 | 2 | 2 |
| Ethylmethylacetylchloride (C5H9ClO) | XRPVXVRWIDOORM-UHFFFAOYSA-N | CCC(C)C(=O)Cl | 10 | 2 | 2 | 2 | 2 | 2 | 2 |
| Pivalyl chloride (C5H9ClO) | JVSFQJZRHXAUGT-UHFFFAOYSA-N | CC(C)(C)C(=O)Cl | 0 | 2 | 2 | 2 | 2 | 2 | 2 |
| 1,4-Dichloro-2-methylbutane (C5H10Cl2) | OUSZUUNUORQHDW-UHFFFAOYSA-N | CC(CCCl)CCl | 19 | 2 | 2 | 2 | 2 | 2 | 2 |
| Pentane, 3-chloro-3-methyl- (C6H13Cl) | SGWJUIFOPCZXMR-UHFFFAOYSA-N | CCC(C)(CC)Cl | 17 | 2 | 2 | 2 | 2 | 2 | 2 |
| 1-Propene, 1,2,3,3-tetrachloro- (C3H2Cl4) | JUGQRTGGLWOBPG-UPHRSURJSA-N | C(=C(/C(Cl)Cl)\Cl)\Cl | 1601 | 1 | 1 | 1 | 1 | 1 | 2 |
| 4,5-Dichloroimidazole (C3H2Cl2N2) | CHUPRLGXGZETTE-UHFFFAOYSA-N | C1=NNC(=C1Cl)Cl | 9999 | 1 | 1 | 1 | 1 | 1 | 1 |
| 1-chlorocyclohex-1-ene (C6H9Cl) | BUAKPITZELZWNI-UHFFFAOYSA-N | C1CCC(=CC1)Cl | 2329 | 1 | 1 | 1 | 1 | 1 | 1 |
| 1,1,3,3-Tetrachloroacetone (C3H2Cl4O) | DJWVKJAGMVZYFP-UHFFFAOYSA-N | C(C(=O)C(Cl)Cl)(Cl)Cl | 279 | 2 | 2 | 2 | 2 | 2 | 2 |
| 2-Propanol, 1-chloro-3-ethoxy- (C5H11ClO2) | XHIINWKFCZSGNY-UHFFFAOYSA-N | CCOCC(CCl)O | 4 | 2 | 2 | 2 | 2 | 2 | 2 |
| 3-Chloro-2,4-pentanedione (C5H7ClO2) | VLRGXXKFHVJQOL-UHFFFAOYSA-N | CC(=O)C(C(=O)C)Cl | 6349 | 1 | 2 | 2 | 2 | 2 | 2 |
| Hexane, 3-chloro-3-methyl- (C7H15Cl) | UTKDCNDVTOWUHW-UHFFFAOYSA-N | CCCC(C)(CC)Cl | 0 | 2 | 2 | 2 | 2 | 2 | 2 |
| 2-Chloro-2,4-dimethylpentane (C7H15Cl) | DQOHPSPKVODKLV-UHFFFAOYSA-N | CC(C)CC(C)(C)Cl | 3 | 2 | 2 | 2 | 2 | 2 | 2 |
| 2-Propenoic acid, 2-chloroethyl ester (C5H7ClO2) | WHBAYNMEIXUTJV-UHFFFAOYSA-N | C=CC(=O)OCCCl | 0 | 2 | 2 | 2 | 2 | 2 | 2 |
| 2,6-Dichloropyrazine (C4H2Cl2N2) | LSEAAPGIZCDEEH-UHFFFAOYSA-N | C1=C(N=C(C=N1)Cl)Cl | 9999 | 1 | 1 | 1 | 1 | 1 | 1 |
| 3-Amino-2-chloropyridine (C5H5ClN2) | MEQBJJUWDCYIAB-UHFFFAOYSA-N | C1=CC(=C(N=C1)Cl)N | 9999 | 1 | 1 | 1 | 1 | 1 | 1 |
| 1,3-Dichlorobenzene (C6H4Cl2) | ZPQOPVIELGIULI-UHFFFAOYSA-N | C1=CC(=CC(=C1)Cl)Cl | 9999 | 1 | 1 | 1 | 1 | 1 | 1 |
| Benzyl chloride (C7H7Cl) | KCXMKQUNVWSEMD-UHFFFAOYSA-N | C1=CC=C(C=C1)CCl | 2489 | 1 | 1 | 1 | 1 | 1 | 1 |
| 2,2-Dichlorocyclopropylacetonitrile (C5H5Cl2N) | METYSMJJRFRDPP-UHFFFAOYSA-N | C1C(C1(Cl)Cl)CC#N | 18 | 2 | 2 | 2 | 2 | 2 | 2 |
| Bis(β-chloroethyl) sulfone (C4H8Cl2O2S) | LUYAMNYBNTVQJG-UHFFFAOYSA-N | C(CCl)S(=O)(=O)CCCl | 0 | 2 | 2 | 2 | 2 | 2 | 2 |
| Butanoic acid, 2-chloro-3-oxo-, methyl ester (C5H7ClO3) | GYQRIAVRKLRQKP-UHFFFAOYSA-N | CC(=O)C(C(=O)OC)Cl | 169 | 2 | 2 | 2 | 2 | 2 | 2 |
| 3-chloropropyl chloroacetate (C5H8Cl2O2) | CWZVJVDIQWKNJX-UHFFFAOYSA-N | C(COC(=O)CCl)CCl | 11 | 2 | 2 | 2 | 2 | 2 | 2 |
| 2,3-Dichloropropyl acetate (C5H8Cl2O2) | BVXPMFQVOWRQKD-UHFFFAOYSA-N | CC(=O)OCC(CCl)Cl | 0 | 2 | 2 | 2 | 2 | 2 | 2 |
| 2,4-Dichlorobutanoic acid, methyl ester (C5H8Cl2O2) | MIXXUSQBRXDJHD-UHFFFAOYSA-N | COC(=O)C(CCCl)Cl | 0 | 2 | 2 | 2 | 2 | 2 | 2 |
| 4,4-Dichlorobutanoic acid, methyl ester (C5H8Cl2O2) | FLTUWIKFBYMPIV-UHFFFAOYSA-N | COC(=O)CCC(Cl)Cl | 0 | 2 | 2 | 2 | 2 | 2 | 2 |
| Methyl threo-2,3-dichlorobutanoate (C5H8Cl2O2) | KJZMAXQPXRBYDC-UHFFFAOYSA-N | CC(C(C(=O)OC)Cl)Cl | 0 | 2 | 2 | 2 | 2 | 2 | 2 |
| 2-Propanol, 1-chloro-3-isopropoxy- (C6H13ClO2) | GQPJSBQMFFGZAU-UHFFFAOYSA-N | CC(C)OCC(CCl)O | 0 | 2 | 2 | 2 | 2 | 2 | 2 |
| Chloromethyl pivalate (C6H11ClO2) | GGRHYQCXXYLUTL-UHFFFAOYSA-N | CC(C)(C)C(=O)OCCl | 60 | 2 | 2 | 2 | 2 | 2 | 2 |
| Propane, 1,1'-thiobis[3-chloro- (C6H12Cl2S) | VDTHWBLOSZIMMN-UHFFFAOYSA-N | C(CSCCCCl)CCl | 1090 | 1 | 2 | 2 | 1 | 2 | 2 |
| Propane, 1,1'-oxybis[3-chloro- (C6H12Cl2O) | SMANNJALMIGASX-UHFFFAOYSA-N | C(COCCCCl)CCl | 10 | 2 | 2 | 2 | 2 | 2 | 2 |
| Bis(2-chloropropyl) sulfide (C6H12Cl2S) | AQHTWLYIOSPNMG-UHFFFAOYSA-N | CC(CSCC(C)Cl)Cl | 2913 | 1 | 2 | 1 | 2 | 2 | 2 |
| bis(2-chloro-1-methylethyl) ether (C6H12Cl2O) | QCFYJCYNJLBDRT-UHFFFAOYSA-N | CC(CCl)OC(C)CCl | 0 | 2 | 2 | 2 | 2 | 2 | 2 |
| 2-Butanone, 1,1-dichloro-3,3-dimethyl- (C6H10Cl2O) | UDWZXMQIEHAAQT-UHFFFAOYSA-N | CC(C)(C)C(=O)C(Cl)Cl | 0 | 2 | 2 | 2 | 2 | 2 | 2 |
| 2-Chlorooctane (C8H17Cl) | HKDCIIMOALDWHF-UHFFFAOYSA-N | CCCCCCC(C)Cl | 0 | 2 | 2 | 2 | 2 | 2 | 2 |
| Desethyl-desisopropyl-atrazine (C3H4ClN5) | FVFVNNKYKYZTJU-UHFFFAOYSA-N | C1(=NC(=NC(=N1)Cl)N)N | 9999 | 1 | 1 | 1 | 1 | 1 | 1 |
| Pyridazine, 3-chloro-6-methoxy- (C5H5ClN2O) | XBJLKXOOHLLTPG-UHFFFAOYSA-N | COC1=NN=C(C=C1)Cl | 6058 | 1 | 1 | 1 | 1 | 1 | 1 |
| 3,4,5-Trichloropyridine (C5H2Cl3N) | KKWRVUBDCJQHBZ-UHFFFAOYSA-N | C1=C(C(=C(C=N1)Cl)Cl)Cl | 9999 | 1 | 1 | 1 | 1 | 1 | 1 |
| Benzenamine, 2-chloro-N-methyl- (C7H8ClN) | WGNNILPYHCKCFF-UHFFFAOYSA-N | CNC1=CC=CC=C1Cl | 6906 | 1 | 1 | 1 | 1 | 1 | 1 |
| 3-Chloroanisole (C7H7ClO) | YUKILTJWFRTXGB-UHFFFAOYSA-N | COC1=CC(=CC=C1)Cl | 9999 | 1 | 1 | 1 | 1 | 1 | 1 |
| 4-Chloroanisole (C7H7ClO) | YRGAYAGBVIXNAQ-UHFFFAOYSA-N | COC1=CC=C(C=C1)Cl | 9999 | 1 | 1 | 1 | 1 | 1 | 1 |
| 2,6-Dichloroaniline (C6H5Cl2N) | JDMFXJULNGEPOI-UHFFFAOYSA-N | C1=CC(=C(C(=C1)Cl)N)Cl | 9999 | 1 | 1 | 1 | 1 | 1 | 1 |
| 4-Chlorobenzyl mercaptan (C7H7ClS) | GKQXPTHQTXCXEV-UHFFFAOYSA-N | C1=CC(=CC=C1CS)Cl | 2009 | 1 | 1 | 1 | 1 | 1 | 1 |
| m-Chlorobenzaldehyde (C7H5ClO) | SRWILAKSARHZPR-UHFFFAOYSA-N | C1=CC(=CC(=C1)Cl)C=O | 8219 | 1 | 1 | 1 | 1 | 1 | 1 |
| Phenol, 5-chloro-2-methyl- (C7H7ClO) | KKFPXGXMSBBNJI-UHFFFAOYSA-N | CC1=C(C=C(C=C1)Cl)O | 4812 | 1 | 1 | 1 | 1 | 1 | 1 |
| Phenol, 3-chloro-4-methyl- (C7H7ClO) | VQZRLBWPEHFGCD-UHFFFAOYSA-N | CC1=C(C=C(C=C1)O)Cl | 4165 | 1 | 1 | 1 | 1 | 1 | 1 |
| Benzene, 1-chloro-3-(chloromethyl)- (C7H6Cl2) | DDGRAFHHXYIQQR-UHFFFAOYSA-N | C1=CC(=CC(=C1)Cl)CCl | 2189 | 1 | 1 | 1 | 1 | 1 | 1 |
| 2-Chloro-3,6-dimethylpyrazine (C6H7ClN2) | NNBALVIZMGWZHS-UHFFFAOYSA-N | CC1=CN=C(C(=N1)Cl)C | 9999 | 1 | 1 | 1 | 1 | 1 | 1 |
| Benzene, 1-(chloromethyl)-2-methyl- (C8H9Cl) | VQRBXYBBGHOGFT-UHFFFAOYSA-N | CC1=CC=CC=C1CCl | 2236 | 1 | 1 | 1 | 1 | 1 | 1 |
| Benzene, 1-chloro-3-ethyl- (C8H9Cl) | LOXUEGMPESDGBQ-UHFFFAOYSA-N | CCC1=CC(=CC=C1)Cl | 5215 | 1 | 1 | 1 | 1 | 1 | 1 |
| Benzene, 1-chloro-4-ethyl- (C8H9Cl) | GPOFSFLJOIAMSA-UHFFFAOYSA-N | CCC1=CC=C(C=C1)Cl | 3633 | 1 | 1 | 1 | 1 | 1 | 1 |
| m-Xylene, 2-chloro- (C8H9Cl) | VDXLAYAQGYCQEO-UHFFFAOYSA-N | CC1=C(C(=CC=C1)C)Cl | 4419 | 1 | 1 | 1 | 1 | 1 | 1 |
| Cyclohexanone,2-chloro-2-methyl- (C7H11ClO) | IDRWHLQDVSLCBJ-UHFFFAOYSA-N | CC1(CCCCC1=O)Cl | 1700 | 1 | 2 | 2 | 1 | 2 | 1 |
| 4-chlorostyrene (C8H7Cl) | KTZVZZJJVJQZHV-UHFFFAOYSA-N | C=CC1=CC=C(C=C1)Cl | 9999 | 1 | 1 | 1 | 1 | 1 | 1 |
| Benzene, 1-chloro-3-ethenyl- (C8H7Cl) | BOVQCIDBZXNFEJ-UHFFFAOYSA-N | C=CC1=CC(=CC=C1)Cl | 9999 | 1 | 1 | 1 | 1 | 1 | 1 |
| 5-Chloro-2-thiophenecarboxylic acid (C5H3ClO2S) | QZLSBOVWPHXCLT-UHFFFAOYSA-N | C1=C(SC(=C1)Cl)C(=O)O | 6360 | 1 | 1 | 1 | 1 | 1 | 1 |
| Propanoic acid, 2,2,3,3-tetrachloro, methyl ester (C4H4Cl4O2) | UQYYMXYJSOOLLA-UHFFFAOYSA-N | COC(=O)C(C(Cl)Cl)(Cl)Cl | 0 | 2 | 2 | 2 | 2 | 2 | 2 |
| 1,1,1,2,2,3,3-Heptachloropropane (C3HCl7) | YFIIENAGGCUHIQ-UHFFFAOYSA-N | C(C(C(Cl)(Cl)Cl)(Cl)Cl)(Cl)Cl | 0 | 2 | 2 | 2 | 2 | 2 | 2 |
| Ethanol, 2-[2-(2-chloroethoxy)ethoxy]- (C6H13ClO3) | KECMLGZOQMJIBM-UHFFFAOYSA-N | C(COCCOCCCl)O | 0 | 2 | 2 | 2 | 2 | 2 | 2 |
| Acetic acid, dichloro, isobutyl ester (C6H10Cl2O2) | XLKJPQKUATYFQJ-UHFFFAOYSA-N | CC(C)COC(=O)C(Cl)Cl | 0 | 2 | 2 | 2 | 2 | 2 | 2 |
| Acetic acid, chloro-, 3-methylbutyl ester (C7H13ClO2) | UZQBACINTKFBSX-UHFFFAOYSA-N | CC(C)CCOC(=O)CCl | 0 | 2 | 2 | 2 | 2 | 2 | 2 |
| Propanoic acid, 3-chloro, 1-methylpropyl ester (C7H13ClO2) | XLIOZHNKYDPTAJ-UHFFFAOYSA-N | CCC(C)OC(=O)CCCl | 0 | 2 | 2 | 2 | 2 | 2 | 2 |
| Octanoyl chloride (C8H15ClO) | REEZZSHJLXOIHL-UHFFFAOYSA-N | CCCCCCCC(=O)Cl | 0 | 2 | 2 | 2 | 2 | 2 | 2 |
| 2-ethylhexanoyl chloride (C8H15ClO) | WFSGQBNCVASPMW-UHFFFAOYSA-N | CCCCC(CC)C(=O)Cl | 0 | 2 | 2 | 2 | 2 | 2 | 2 |
| Trichloroacetic acid 2-propenyl ester (C5H5Cl3O2) | LJQCONXCOYBYIE-UHFFFAOYSA-N | C=CCOC(=O)C(Cl)(Cl)Cl | 50 | 2 | 2 | 2 | 2 | 2 | 2 |
| 4-Pyrimidinamine, 6-chloro-2-(methylthio)- (C5H6ClN3S) | ISUXMAHVLFRZQU-UHFFFAOYSA-N | CSC1=NC(=CC(=N1)Cl)N | 9999 | 1 | 1 | 1 | 1 | 1 | 1 |
| Pyrimidine, 4,6-dichloro-2-(methylthio)- (C5H4Cl2N2S) | FCMLONIWOAGZJX-UHFFFAOYSA-N | CSC1=NC(=CC(=N1)Cl)Cl | 9999 | 1 | 1 | 1 | 1 | 1 | 1 |
| 2-Hydroxy-3,5,6-trichloropyridine (C5H2Cl3NO) | WCYYAQFQZQEUEN-UHFFFAOYSA-N | C1=C(C(=O)NC(=C1Cl)Cl)Cl | 9145 | 1 | 1 | 1 | 1 | 1 | 1 |
| 2-Chlorophenyl isothiocyanate (C7H4ClNS) | DASSPOJBUMBXLU-UHFFFAOYSA-N | C1=CC=C(C(=C1)N=C=S)Cl | 9999 | 1 | 1 | 1 | 1 | 1 | 1 |
| 1-Chloro-3-isocyanatobenzene (C7H4ClNO) | HHIRBXHEYVDUAM-UHFFFAOYSA-N | C1=CC(=CC(=C1)Cl)N=C=O | 9999 | 1 | 1 | 1 | 1 | 1 | 1 |
| Benzene, 1-chloro-4-[(chloromethyl)thio]- (C7H6Cl2S) | XPJUCMIJGVAEGF-UHFFFAOYSA-N | C1=CC(=CC=C1SCCl)Cl | 1100 | 1 | 1 | 1 | 1 | 1 | 1 |
| 1,3-Benzenediol, 4,6-dichloro- (C6H4Cl2O2) | GRLQBYQELUWBIO-UHFFFAOYSA-N | C1=C(C(=CC(=C1O)Cl)Cl)O | 9999 | 1 | 1 | 1 | 1 | 1 | 1 |
| Benzene, 1,4-dichloro-2-methoxy- (C7H6Cl2O) | QKMNFFSBZRGHDJ-UHFFFAOYSA-N | COC1=C(C=CC(=C1)Cl)Cl | 9999 | 1 | 1 | 1 | 1 | 1 | 1 |
| 2,4,6-Trichloroaniline (C6H4Cl3N) | NATVSFWWYVJTAZ-UHFFFAOYSA-N | C1=C(C=C(C(=C1Cl)N)Cl)Cl | 9999 | 1 | 1 | 1 | 1 | 1 | 1 |
| 3-Chlorobenzamide (C7H6ClNO) | MJTGQALMWUUPQM-UHFFFAOYSA-N | C1=CC(=CC(=C1)Cl)C(=O)N | 7229 | 1 | 1 | 1 | 1 | 1 | 1 |
| Benzene, 1-(chloromethyl)-2-methoxy- (C8H9ClO) | UAWVMPOAIVZWFQ-UHFFFAOYSA-N | COC1=CC=CC=C1CCl | 2477 | 1 | 1 | 1 | 1 | 1 | 1 |
| Benzene, 1-chloro-2-methyl-4-methoxy (C8H9ClO) | SDGMUBWPXBSKCT-UHFFFAOYSA-N | CC1=C(C=CC(=C1)OC)Cl | 9999 | 1 | 1 | 1 | 1 | 1 | 1 |
| 3-Chloro-4-methoxytoluene (C8H9ClO) | VUZBRBKYGIQXMP-UHFFFAOYSA-N | CC1=CC(=C(C=C1)OC)Cl | 5155 | 1 | 1 | 1 | 1 | 1 | 1 |
| 2,5-Dichlorobenzylamine (C7H7Cl2N) | AKGJLIXNRPNPCH-UHFFFAOYSA-N | C1=CC(=C(C=C1Cl)CN)Cl | 1372 | 1 | 1 | 1 | 1 | 1 | 1 |
| 2,4-Dichlorobenzylamine (C7H7Cl2N) | SJUKJZSTBBSGHF-UHFFFAOYSA-N | C1=CC(=C(C=C1Cl)Cl)CN | 710 | 2 | 1 | 1 | 1 | 1 | 1 |
| 3,4-Dichlorobenzyl alcohol (C7H6Cl2O) | FVJIUQSKXOYFKG-UHFFFAOYSA-N | C1=CC(=C(C=C1CO)Cl)Cl | 6599 | 1 | 1 | 1 | 1 | 1 | 1 |
| Benzaldehyde, 2,6-dichloro- (C7H4Cl2O) | DMIYKWPEFRFTPY-UHFFFAOYSA-N | C1=CC(=C(C(=C1)Cl)C=O)Cl | 6139 | 1 | 1 | 1 | 1 | 1 | 1 |
| α,3,4-Trichlorotoluene (C7H5Cl3) | YZIFVWOCPGPNHB-UHFFFAOYSA-N | C1=CC(=C(C=C1CCl)Cl)Cl | 1869 | 1 | 1 | 1 | 1 | 1 | 1 |
| 3-Chloro-2-methylbenzonitrile (C8H6ClN) | FKFZTNLSUJCIMG-UHFFFAOYSA-N | CC1=C(C=CC=C1Cl)C#N | 4306 | 1 | 1 | 1 | 1 | 1 | 1 |
| Benzoyl chloride, 2-methyl- (C8H7ClO) | GPZXFICWCMCQPF-UHFFFAOYSA-N | CC1=CC=CC=C1C(=O)Cl | 449 | 2 | 2 | 2 | 2 | 2 | 1 |
| Benzene, 1,3-bis(chloromethyl)- (C8H8Cl2) | GRJWOKACBGZOKT-UHFFFAOYSA-N | C1=CC(=CC(=C1)CCl)CCl | 2089 | 1 | 1 | 1 | 1 | 1 | 2 |
| 4-(Chloromethyl)-1-ethylbenzene (C9H11Cl) | DUBCVXSYZVTCOC-UHFFFAOYSA-N | CCC1=CC=C(C=C1)CCl | 3194 | 1 | 1 | 1 | 1 | 1 | 1 |
| 3,5-Dimethylbenzyl chloride (C9H11Cl) | FYNVRRYQTHUESZ-UHFFFAOYSA-N | CC1=CC(=CC(=C1)CCl)C | 2100 | 1 | 1 | 1 | 1 | 1 | 1 |
| Benzene, 2-(chloromethyl)-1,4-dimethyl- (C9H11Cl) | PECXPZGFZFGDRD-UHFFFAOYSA-N | CC1=CC(=C(C=C1)C)CCl | 1942 | 1 | 1 | 1 | 1 | 1 | 1 |
| Benzene, (3-chloroallyl)- (C9H9Cl) | JJTUJRVKTPSEFZ-XBXARRHUSA-N | C1=CC=C(C=C1)C/C=C/Cl | 2482 | 1 | 1 | 1 | 1 | 1 | 1 |
| Cyclopropanecarboxylic acid, 3-chloroprop-2-enyl ester (C7H9ClO2) | QAJSCZWJFSRULT-DAFODLJHSA-N | C1CC1C(=O)OC/C=C/Cl | 0 | 2 | 2 | 2 | 2 | 2 | 2 |
| 6-Chloropurine (C5H3ClN4) | ZKBQDFAWXLTYKS-UHFFFAOYSA-N | C1=NC2=C(N1)C(=NC=N2)Cl | 9999 | 1 | 1 | 1 | 1 | 1 | 1 |
| 2-Chlorobenzimidazole (C7H5ClN2) | AYPSHJCKSDNETA-UHFFFAOYSA-N | C1=CC=C2C(=C1)NC(=N2)Cl | 9999 | 1 | 1 | 1 | 1 | 1 | 1 |
| Phosphonic acid, (2-chloroethyl)-, diethyl ester (C6H14ClO3P) | GMDLEOVIACJWTD-UHFFFAOYSA-N | CCOP(=O)(CCCl)OCC | 0 | 2 | 2 | 2 | 2 | 2 | 2 |
| Trichloroacetic acid, but-3-yn-2-yl ester (C6H5Cl3O2) | KEBRGWUCOLOSMN-UHFFFAOYSA-N | CC(C#C)OC(=O)C(Cl)(Cl)Cl | 0 | 2 | 2 | 2 | 2 | 2 | 2 |
| Butyl trichloroacetate (C6H9Cl3O2) | SECVZLDDYUWJAC-UHFFFAOYSA-N | CCCCOC(=O)C(Cl)(Cl)Cl | 0 | 2 | 2 | 2 | 2 | 2 | 2 |
| 3-Chlorohexanoic acid, chloromethyl ester (C7H12Cl2O2) | FWVZTAJOBMVDDT-UHFFFAOYSA-N | CCCC(CC(=O)OCCl)Cl | 0 | 2 | 2 | 2 | 2 | 2 | 2 |
| 2-Chlorohexanoic acid, chloromethyl ester (C7H12Cl2O2) | SLCVMKYCEWYVKS-UHFFFAOYSA-N | CCCCC(C(=O)OCCl)Cl | 0 | 2 | 2 | 2 | 2 | 2 | 2 |
| Hexyl chloroacetate (C8H15ClO2) | OJGRZJILAIHWIY-UHFFFAOYSA-N | CCCCCCOC(=O)CCl | 0 | 2 | 2 | 2 | 2 | 2 | 2 |
| 3-Chlorodecane (C10H21Cl) | SMVZPOXWOUHGQI-UHFFFAOYSA-N | CCCCCCCC(CC)Cl | 0 | 2 | 2 | 2 | 2 | 2 | 2 |
| Trichloroacetic acid, 3-chloroprop-2-enyl ester (C5H4Cl4O2) | KPJDOQORQYKCEB-OWOJBTEDSA-N | C(/C=C/Cl)OC(=O)C(Cl)(Cl)Cl | 120 | 2 | 2 | 2 | 2 | 2 | 2 |
| Phenol, 2-chloro-4-nitro- (C6H4ClNO3) | BOFRXDMCQRTGII-UHFFFAOYSA-N | C1=CC(=C(C=C1[N+](=O)[O-])Cl)O | 9920 | 1 | 1 | 1 | 1 | 1 | 1 |
| 2-Chloro-4-methoxyphenol, methyl ether (C8H9ClO2) | QMXZSRVFIWACJH-UHFFFAOYSA-N | COC1=CC(=C(C=C1)OC)Cl | 5915 | 1 | 1 | 1 | 1 | 1 | 1 |
| Benzene, 2,4-dichloro-1-nitro- (C6H3Cl2NO2) | QUIMTLZDMCNYGY-UHFFFAOYSA-N | C1=CC(=C(C=C1Cl)Cl)[N+](=O)[O-] | 9199 | 1 | 1 | 1 | 1 | 1 | 1 |
| 3,5-Dichlorophenyl isocyanate (C7H3Cl2NO) | XEFUJGURFLOFAN-UHFFFAOYSA-N | C1=C(C=C(C=C1Cl)Cl)N=C=O | 9999 | 1 | 1 | 1 | 1 | 1 | 1 |
| 2-Methoxy-3,6-dichloro-phenol (C7H6Cl2O2) | OBRQSFBOZCMSTK-UHFFFAOYSA-N | COC1=C(C=CC(=C1O)Cl)Cl | 6605 | 1 | 1 | 1 | 1 | 1 | 1 |
| Benzene, 1,2,4-trichloro-5-methoxy- (C7H5Cl3O) | SXKBHOQOOGRFJF-UHFFFAOYSA-N | COC1=CC(=C(C=C1Cl)Cl)Cl | 9999 | 1 | 1 | 1 | 1 | 1 | 1 |
| Phenol, 2,3,4,6-tetrachloro- (C6H2Cl4O) | VGVRPFIJEJYOFN-UHFFFAOYSA-N | C1=C(C(=C(C(=C1Cl)Cl)Cl)O)Cl | 7877 | 1 | 1 | 1 | 1 | 1 | 1 |
| Desisopropylatrazine (C5H8ClN5) | IVENSCMCQBJAKW-UHFFFAOYSA-N | CCNC1=NC(=NC(=N1)N)Cl | 5005 | 1 | 1 | 1 | 1 | 1 | 1 |
| 1,3,5-Triazin-2-amine, 4,6-dichloro-N-ethyl- (C5H6Cl2N4) | ACAHVXOSWOUZAB-UHFFFAOYSA-N | CCNC1=NC(=NC(=N1)Cl)Cl | 4804 | 1 | 1 | 1 | 1 | 1 | 1 |
| 3-Chlorobenzhydrazide (C7H7ClN2O) | PHRDZSRVSVNQRN-UHFFFAOYSA-N | C1=CC(=CC(=C1)Cl)C(=O)NN | 1571 | 1 | 1 | 1 | 1 | 1 | 1 |
| Benzoic acid, 2-amino-5-chloro- (C7H6ClNO2) | IFXKXCLVKQVVDI-UHFFFAOYSA-N | C1=CC(=C(C=C1Cl)C(=O)O)N | 5989 | 1 | 1 | 1 | 1 | 1 | 1 |
| Acetic acid, 3-chlorophenyl ester (C8H7ClO2) | GQTKYLQYHPTULY-UHFFFAOYSA-N | CC(=O)OC1=CC(=CC=C1)Cl | 2072 | 1 | 1 | 1 | 1 | 1 | 1 |
| Benzoic acid, 2,6-dichloro- (C7H4Cl2O2) | MRUDNSFOFOQZDA-UHFFFAOYSA-N | C1=CC(=C(C(=C1)Cl)C(=O)O)Cl | 5262 | 1 | 1 | 1 | 1 | 1 | 1 |
| 3,5-Dichlorobenzoic acid (C7H4Cl2O2) | CXKCZFDUOYMOOP-UHFFFAOYSA-N | C1=C(C=C(C=C1Cl)Cl)C(=O)O | 9999 | 1 | 1 | 1 | 1 | 1 | 1 |
| 1,3-dichloro-2-(methoxymethyl)benzene (C8H8Cl2O) | QBKBHXIQLAMKOB-UHFFFAOYSA-N | COCC1=C(C=CC=C1Cl)Cl | 1401 | 1 | 1 | 1 | 1 | 2 | 1 |
| 2,4,6-Trichlorobenzonitrile (C7H2Cl3N) | PGODHCIOIPODFE-UHFFFAOYSA-N | C1=C(C=C(C(=C1Cl)C#N)Cl)Cl | 9999 | 1 | 1 | 1 | 1 | 1 | 1 |
| Benzoyl chloride, 3,4-dichloro- (C7H3Cl3O) | VTXNOVCTHUBABW-UHFFFAOYSA-N | C1=CC(=C(C=C1C(=O)Cl)Cl)Cl | 989 | 1 | 1 | 1 | 1 | 1 | 1 |
| m-Chlorophenylacetic acid (C8H7ClO2) | WFPMUFXQDKMVCO-UHFFFAOYSA-N | C1=CC(=CC(=C1)Cl)CC(=O)O | 3929 | 1 | 1 | 1 | 1 | 1 | 1 |
| 4-Methoxy-3-methylbenzyl chloride (C9H11ClO) | BHEHNICAPZVKRH-UHFFFAOYSA-N | CC1=C(C=CC(=C1)CCl)OC | 1572 | 1 | 1 | 1 | 1 | 1 | 1 |
| 2,6-Dichloroacetophenone (C8H6Cl2O) | HYBDSXBLGCQKRE-UHFFFAOYSA-N | CC(=O)C1=C(C=CC=C1Cl)Cl | 1550 | 1 | 1 | 1 | 1 | 1 | 1 |
| Benzene, (2,2,2-trichloroethyl)- (C8H7Cl3) | XFEKIQFBJSDMQB-UHFFFAOYSA-N | C1=CC=C(C=C1)CC(Cl)(Cl)Cl | 611 | 2 | 2 | 2 | 2 | 2 | 2 |
| Dichloroacetic acid, morpholide (C6H9Cl2NO2) | SPDHGKQMFGYCHN-UHFFFAOYSA-N | C1COCCN1C(=O)C(Cl)Cl | 1231 | 1 | 1 | 2 | 2 | 2 | 2 |
| p-(Chlorophenyl)acetone (C9H9ClO) | WEJRYKSUUFKMBC-UHFFFAOYSA-N | CC(=O)CC1=CC=C(C=C1)Cl | 573 | 2 | 2 | 2 | 2 | 2 | 2 |
| Cyclopropane, 1,1-dichloro-2,2,3-triethyl- (C9H16Cl2) | HZZCFNUFGQXPQC-UHFFFAOYSA-N | CCC1C(C1(Cl)Cl)(CC)CC | 300 | 2 | 2 | 2 | 2 | 2 | 2 |
| 6-Chloro-2,4-dihydroxy-1,3-dimethylpyrimidine (C6H7ClN2O2) | VATQPUHLFQHDBD-UHFFFAOYSA-N | CN1C(=CC(=O)N(C1=O)C)Cl | 2592 | 1 | 1 | 1 | 1 | 1 | 1 |
| 8-Chloroquinoline (C9H6ClN) | RUSMDMDNFUYZTM-UHFFFAOYSA-N | C1=CC2=C(C(=C1)Cl)N=CC=C2 | 9999 | 1 | 1 | 1 | 1 | 1 | 1 |
| Bornyl chloride (C10H17Cl) | XXZAOMJCZBZKPV-UHFFFAOYSA-N | CC1(C2CCC1(C(C2)Cl)C)C | 0 | 2 | 1 | 1 | 2 | 1 | 1 |
| Pentyl trichloroacetate (C7H11Cl3O2) | LZJOVWVTJGLPFN-UHFFFAOYSA-N | CCCCCOC(=O)C(Cl)(Cl)Cl | 0 | 2 | 2 | 2 | 2 | 2 | 2 |
| Acetic acid, trichloro-, 3-methylbutyl ester (C7H11Cl3O2) | HBNLTLLITHPMDZ-UHFFFAOYSA-N | CC(C)CCOC(=O)C(Cl)(Cl)Cl | 0 | 2 | 2 | 2 | 2 | 2 | 2 |
| Propanoic acid, 3-chloro, hexyl ester (C9H17ClO2) | PYOAHWWMEFRKLM-UHFFFAOYSA-N | CCCCCCOC(=O)CCCl | 0 | 2 | 2 | 2 | 2 | 2 | 2 |
| 10-Chloro-1-decanol (C10H21ClO) | OTUSESJECXGMIV-UHFFFAOYSA-N | C(CCCCCCl)CCCCO | 0 | 2 | 2 | 2 | 2 | 2 | 2 |
| Formamidine, 3,3-dimethyl-1-(4-chlorophenyl) (C9H11ClN2) | ZPTXBCJETBDOAT-UHFFFAOYSA-N | CN(C)C=NC1=CC=C(C=C1)Cl | 9999 | 1 | 1 | 1 | 1 | 1 | 1 |
| Benzene, 1,2,4-trichloro-5-nitro- (C6H2Cl3NO2) | IBRBMZRLVYKVRF-UHFFFAOYSA-N | C1=C(C(=CC(=C1Cl)Cl)Cl)[N+](=O)[O-] | 9999 | 1 | 1 | 1 | 1 | 1 | 1 |
| 2-(4-Chlorophenoxy)thioacetamide (C8H8ClNOS) | AIOQDHOVIDONNW-UHFFFAOYSA-N | C1=CC(=CC=C1OCC(=S)N)Cl | 1511 | 1 | 1 | 1 | 1 | 1 | 2 |
| 4-Nitrobenzoyl chloride (C7H4ClNO3) | SKDHHIUENRGTHK-UHFFFAOYSA-N | C1=CC(=CC=C1C(=O)Cl)[N+](=O)[O-] | 90 | 2 | 2 | 1 | 2 | 2 | 2 |
| 3',4'-Dichloroacetanilide (C8H7Cl2NO) | SCYGGCAQZFJGRF-UHFFFAOYSA-N | CC(=O)NC1=CC(=C(C=C1)Cl)Cl | 2449 | 1 | 1 | 1 | 1 | 1 | 1 |
| Phenol, 3,4-dichloro-, acetate (C8H6Cl2O2) | OSKGYRIYNMZFSJ-UHFFFAOYSA-N | CC(=O)OC1=CC(=C(C=C1)Cl)Cl | 861 | 1 | 1 | 1 | 1 | 1 | 1 |
| Phenol, 2,5-dichloro-, acetate (C8H6Cl2O2) | XSJDGJRHRDWQOR-UHFFFAOYSA-N | CC(=O)OC1=C(C=CC(=C1)Cl)Cl | 1041 | 1 | 1 | 1 | 1 | 1 | 1 |
| Propanamide, N-(4-chlorophenyl)- (C9H10ClNO) | FFHGCIYKPNISDY-UHFFFAOYSA-N | CCC(=O)NC1=CC=C(C=C1)Cl | 1300 | 1 | 1 | 1 | 1 | 1 | 1 |
| 2-Chloropropionanilide (C9H10ClNO) | VCVUMBWLSGNGFA-UHFFFAOYSA-N | CCC(=O)NC1=CC=CC=C1Cl | 2100 | 1 | 1 | 1 | 1 | 1 | 1 |
| Benzoic acid, 3-chloro-, ethyl ester (C9H9ClO2) | LVFRSNCBCHABAM-UHFFFAOYSA-N | CCOC(=O)C1=CC(=CC=C1)Cl | 1952 | 1 | 1 | 1 | 1 | 1 | 1 |
| Benzene, (4-chlorobutoxy)- (C10H13ClO) | JKXCPAVECBFBOC-UHFFFAOYSA-N | C1=CC=C(C=C1)OCCCCCl | 909 | 1 | 2 | 1 | 1 | 1 | 2 |
| Chlorothymol (C10H13ClO) | KFZXVMNBUMVKLN-UHFFFAOYSA-N | CC1=CC(=C(C=C1Cl)C(C)C)O | 2989 | 1 | 1 | 1 | 1 | 1 | 1 |
| 1-Chloromethyl-3-(1,1-dimethylethyl)benzene (C11H15Cl) | QZWCABOLGIVZCP-UHFFFAOYSA-N | CC(C)(C)C1=CC=CC(=C1)CCl | 1300 | 1 | 1 | 1 | 1 | 1 | 1 |
| β-BHC (C6H6Cl6) | JLYXXMFPNIAWKQ-UHFFFAOYSA-N | C1(C(C(C(C(C1Cl)Cl)Cl)Cl)Cl)Cl | 279 | 2 | 2 | 2 | 2 | 2 | 2 |
| m-Chlorocinnamic acid (C9H7ClO2) | FFKGOJWPSXRALK-SNAWJCMRSA-N | C1=CC(=CC(=C1)Cl)/C=C/C(=O)O | 9999 | 1 | 1 | 1 | 1 | 1 | 1 |
| p-Benzoquinone, 2,3,5,6-tetrachloro- (C6Cl4O2) | UGNWTBMOAKPKBL-UHFFFAOYSA-N | C1(=C(C(=O)C(=C(C1=O)Cl)Cl)Cl)Cl | 4899 | 1 | 1 | 1 | 1 | 1 | 1 |
| Naphthalene, 1,4-dichloro- (C10H6Cl2) | JDPKCYMVSKDOGS-UHFFFAOYSA-N | C1=CC=C2C(=C1)C(=CC=C2Cl)Cl | 9999 | 1 | 1 | 1 | 1 | 1 | 1 |
| 6-Chloropiperonal (C8H5ClO3) | VRNADRCOROWLJC-UHFFFAOYSA-N | C1OC2=C(O1)C=C(C(=C2)C=O)Cl | 7497 | 1 | 1 | 1 | 1 | 1 | 1 |
| 6-Chloro-2-methylquinoline (C10H8ClN) | OCCIBGIEIBQGAJ-UHFFFAOYSA-N | CC1=NC2=C(C=C1)C=C(C=C2)Cl | 9999 | 1 | 1 | 1 | 1 | 1 | 1 |
| Malonic acid, 2-chloropropyl ethyl ester (C8H13ClO4) | LNNJMHLJYAMEAG-UHFFFAOYSA-N | CCOC(=O)CC(=O)OCC(C)Cl | 0 | 2 | 2 | 2 | 2 | 2 | 2 |
| 8-Chlorooctanoic acid, chloromethyl ester (C9H16Cl2O2) | YEDAJMGEJCGDOV-UHFFFAOYSA-N | C(CCCC(=O)OCCl)CCCCl | 0 | 2 | 2 | 2 | 2 | 2 | 2 |
| Heptyl dichloroacetate (C9H16Cl2O2) | DABKLGRTFPUJHU-UHFFFAOYSA-N | CCCCCCCOC(=O)C(Cl)Cl | 0 | 2 | 2 | 2 | 2 | 2 | 2 |
| 5-Chlorovaleric acid, pentyl ester (C10H19ClO2) | LAYFAERBACNODJ-UHFFFAOYSA-N | CCCCCOC(=O)CCCCCl | 0 | 2 | 2 | 2 | 2 | 2 | 2 |
| nonanoic acid, chloromethyl ester (C10H19ClO2) | NNVIRQBZFULPEI-UHFFFAOYSA-N | CCCCCCCCC(=O)OCCl | 120 | 2 | 2 | 2 | 2 | 2 | 2 |
| Benzene, 4-chloro-1,2-dinitro- (C6H3ClN2O4) | QVQSOXMXXFZAKU-UHFFFAOYSA-N | C1=CC(=C(C=C1Cl)[N+](=O)[O-])[N+](=O)[O-] | 9999 | 1 | 1 | 1 | 1 | 1 | 1 |
| 1-Chloro-2,4-dinitrobenzene (C6H3ClN2O4) | VYZAHLCBVHPDDF-UHFFFAOYSA-N | C1=CC(=C(C=C1[N+](=O)[O-])[N+](=O)[O-])Cl | 5469 | 1 | 1 | 1 | 1 | 1 | 1 |
| Monuron (C9H11ClN2O) | BMLIZLVNXIYGCK-UHFFFAOYSA-N | CN(C)C(=O)NC1=CC=C(C=C1)Cl | 2349 | 1 | 1 | 1 | 1 | 1 | 1 |
| Benzene, 3,4,5-trichloro-1,2-dimethoxy (C8H7Cl3O2) | VKNITLPENCJQOP-UHFFFAOYSA-N | COC1=CC(=C(C(=C1OC)Cl)Cl)Cl | 8000 | 1 | 1 | 1 | 1 | 1 | 1 |
| Phenol, 2,3,5,6-tetrachloro-4-methoxy- (C7H4Cl4O2) | XIWJLPHQDBDOAN-UHFFFAOYSA-N | COC1=C(C(=C(C(=C1Cl)Cl)O)Cl)Cl | 4471 | 1 | 1 | 1 | 1 | 1 | 1 |
| 5-Chloro-2-nitrobenzoic acid (C7H4ClNO4) | ZKUYSJHXBFFGPU-UHFFFAOYSA-N | C1=CC(=C(C=C1Cl)C(=O)O)[N+](=O)[O-] | 9999 | 1 | 1 | 1 | 1 | 1 | 1 |
| Carbamic acid, 4-chlorophenyl, ethyl ester (C9H10ClNO2) | WSKXXIMERYQVGJ-UHFFFAOYSA-N | CCOC(=O)NC1=CC=C(C=C1)Cl | 9999 | 1 | 1 | 1 | 1 | 1 | 1 |
| 2-Chloro-4-methoxyphenol, acetate (C9H9ClO3) | OZFXOOSAUSDUCK-UHFFFAOYSA-N | CC(=O)OC1=C(C=C(C=C1)OC)Cl | 350 | 2 | 1 | 1 | 1 | 1 | 1 |
| 2-Chloro-5-nitrobenzoyl chloride (C7H3Cl2NO3) | OGLKKYALUKXVPQ-UHFFFAOYSA-N | C1=CC(=C(C=C1[N+](=O)[O-])C(=O)Cl)Cl | 216 | 2 | 2 | 2 | 2 | 2 | 2 |
| Chloroacetic acid, 3,4-dichlorophenyl ester (C8H5Cl3O2) | CNALHVZXVPTLGY-UHFFFAOYSA-N | C1=CC(=C(C=C1OC(=O)CCl)Cl)Cl | 791 | 2 | 1 | 1 | 1 | 1 | 1 |
| Benzene, 1,2,3,5-tetrachloro-4-ethoxy- (C8H6Cl4O) | OMGPLLBLNOWRAA-UHFFFAOYSA-N | CCOC1=C(C(=C(C=C1Cl)Cl)Cl)Cl | 1348 | 1 | 1 | 1 | 1 | 1 | 1 |
| Chloroacetic acid, 4-cyanophenyl ester (C9H6ClNO2) | APQLVUNYVFVTPC-UHFFFAOYSA-N | C1=CC(=CC=C1C#N)OC(=O)CCl | 901 | 1 | 1 | 1 | 1 | 1 | 1 |
| propanil (C9H9Cl2NO) | LFULEKSKNZEWOE-UHFFFAOYSA-N | CCC(=O)NC1=CC(=C(C=C1)Cl)Cl | 1996 | 1 | 1 | 1 | 1 | 1 | 1 |
| Propanamide, N-(3-methylphenyl)-3-chloro- (C10H12ClNO) | XZPLSUAKDONHQY-UHFFFAOYSA-N | CC1=CC(=CC=C1)NC(=O)CCCl | 2793 | 1 | 1 | 1 | 1 | 1 | 1 |
| Benzoic acid, 4-chloro, propyl ester (C10H11ClO2) | BLEFFSGNRQPNCA-UHFFFAOYSA-N | CCCOC(=O)C1=CC=C(C=C1)Cl | 110 | 2 | 2 | 2 | 2 | 2 | 2 |
| Chloroacetic acid, 3,5-dimethylphenyl ester (C10H11ClO2) | WQVBITVXKZGBJJ-UHFFFAOYSA-N | CC1=CC(=CC(=C1)OC(=O)CCl)C | 1541 | 1 | 1 | 1 | 1 | 1 | 1 |
| Benzoic acid, 2-chloro, 1-methylethyl ester (C10H11ClO2) | ZQVYNQNVFVRHMT-UHFFFAOYSA-N | CC(C)OC(=O)C1=CC=CC=C1Cl | 1051 | 1 | 1 | 2 | 1 | 2 | 1 |
| 2-Methylpropionic acid, 4-chlorophenyl ester (C10H11ClO2) | WPCAYDFRIDVTNI-UHFFFAOYSA-N | CC(C)C(=O)OC1=CC=C(C=C1)Cl | 2442 | 1 | 1 | 1 | 1 | 1 | 1 |
| 4-tert-butylbenzoyl chloride (C11H13ClO) | WNLMYNASWOULQY-UHFFFAOYSA-N | CC(C)(C)C1=CC=C(C=C1)C(=O)Cl | 319 | 2 | 2 | 2 | 2 | 2 | 2 |
| 2,4-Dichloro-ω-nitrostyrene (C8H5Cl2NO2) | LIWIJBBAMBDXME-ONEGZZNKSA-N | C1=CC(=C(C=C1Cl)Cl)/C=C/[N+](=O)[O-] | 2516 | 1 | 1 | 1 | 1 | 1 | 1 |
| Naphthalene, 1,3,7-trichloro- (C10H5Cl3) | CFEUGIGSIREATC-UHFFFAOYSA-N | C1=CC(=CC2=C(C=C(C=C21)Cl)Cl)Cl | 9999 | 1 | 1 | 1 | 1 | 1 | 1 |
| Naphthalene, 1-(chloromethyl)-2-methyl- (C12H11Cl) | STBYRSZXHDPASK-UHFFFAOYSA-N | CC1=C(C2=CC=CC=C2C=C1)CCl | 2521 | 1 | 1 | 1 | 1 | 1 | 1 |
| 2-Chlorobenzo[b]thiophene-3-acetonitrile (C10H6ClNS) | CBWNFQCLFYLDJQ-UHFFFAOYSA-N | C1=CC=C2C(=C1)C(=C(S2)Cl)CC#N | 3937 | 1 | 1 | 1 | 1 | 1 | 1 |
| Succinic acid, 2,2-dichloroethyl ethyl ester (C8H12Cl2O4) | PFNFXPLZQOXXMK-UHFFFAOYSA-N | CCOC(=O)CCC(=O)OCC(Cl)Cl | 0 | 2 | 2 | 2 | 2 | 2 | 2 |
| Malonic acid, 2-chloropropyl propyl ester (C9H15ClO4) | IQLRLUZXLPNYMG-UHFFFAOYSA-N | CCCOC(=O)CC(=O)OCC(C)Cl | 0 | 2 | 2 | 2 | 2 | 2 | 2 |
| Propanamide, N,N-dibutyl-2-chloro- (C11H22ClNO) | AMBSMXGFOZRXNX-UHFFFAOYSA-N | CCCCN(CCCC)C(=O)C(C)Cl | 400 | 2 | 2 | 2 | 2 | 2 | 2 |
| Succinic acid, monochloride, 3,3-dimethylbut-2-yl ester (C10H17ClO3) | PUJBLZZZGPXNRQ-UHFFFAOYSA-N | CC(C(C)(C)C)OC(=O)CCC(=O)Cl | 0 | 2 | 2 | 2 | 2 | 2 | 2 |
| 3-Chlorononanoic acid, chloromethyl ester (C10H18Cl2O2) | QADYLIOQVFEZKX-UHFFFAOYSA-N | CCCCCCC(CC(=O)OCCl)Cl | 0 | 2 | 2 | 2 | 2 | 2 | 2 |
| Monolinuron (C9H11ClN2O2) | LKJPSUCKSLORMF-UHFFFAOYSA-N | CN(C(=O)NC1=CC=C(C=C1)Cl)OC | 1250 | 1 | 1 | 1 | 1 | 1 | 1 |
| Benzene, 2-chloro-5-methyl-1,3-dinitro- (C7H5ClN2O4) | JMDVARRGYWIJGZ-UHFFFAOYSA-N | CC1=CC(=C(C(=C1)[N+](=O)[O-])Cl)[N+](=O)[O-] | 6021 | 1 | 1 | 1 | 1 | 1 | 1 |
| 2,3-Dichlorobenzyl-N-methylcarbamate (C9H9Cl2NO2) | SESJCOBCXSACPH-UHFFFAOYSA-N | CNC(=O)OCC1=C(C(=CC=C1)Cl)Cl | 960 | 1 | 1 | 1 | 2 | 1 | 2 |
| Dichloroacetic acid, 4-methoxyphenyl ester (C9H8Cl2O3) | FYSUBKYBGWIHKT-UHFFFAOYSA-N | COC1=CC=C(C=C1)OC(=O)C(Cl)Cl | 1732 | 1 | 1 | 1 | 1 | 1 | 1 |
| Ethanol, 2-chloro-, 4-methylbenzenesulfonate (C9H11ClO3S) | ZXNMIUJDTOMBPV-UHFFFAOYSA-N | CC1=CC=C(C=C1)S(=O)(=O)OCCCl | 1519 | 1 | 1 | 1 | 2 | 1 | 2 |
| Butyric acid, 3,4-dichlorophenyl ester (C10H10Cl2O2) | GVUWGECOKPZWDC-UHFFFAOYSA-N | CCCC(=O)OC1=CC(=C(C=C1)Cl)Cl | 1121 | 1 | 2 | 2 | 1 | 2 | 2 |
| 4-Chlorobenzoic acid, but-3-yn-2-yl ester (C11H9ClO2) | LRAXWWFDHMSJAF-UHFFFAOYSA-N | CC(C#C)OC(=O)C1=CC=C(C=C1)Cl | 1091 | 1 | 2 | 2 | 2 | 2 | 2 |
| 2-Chloropropionic acid, 3,5-dimethylphenyl ester (C11H13ClO2) | SJELLOBJQJSXPZ-UHFFFAOYSA-N | CC1=CC(=CC(=C1)OC(=O)C(C)Cl)C | 921 | 1 | 1 | 1 | 1 | 1 | 1 |
| Propanamide, N-(3-chlorophenyl)-2,2-dimethyl- (C11H14ClNO) | OGOQXGKPTWSHPS-UHFFFAOYSA-N | CC(C)(C)C(=O)NC1=CC(=CC=C1)Cl | 5485 | 1 | 1 | 1 | 1 | 2 | 2 |
| 1-Propanone, 2-chloro-1-(2,5-dimethylphenyl)-2-methyl- (C12H15ClO) | VXXAHSALZMCFQS-UHFFFAOYSA-N | CC1=CC(=C(C=C1)C)C(=O)C(C)(C)Cl | 70 | 2 | 2 | 2 | 2 | 2 | 2 |
| 2-Chlorobenzoic acid, 3-chloroprop-2-enyl ester (C10H8Cl2O2) | GIMRXOTXLJWYLM-ZZXKWVIFSA-N | C1=CC=C(C(=C1)C(=O)OC/C=C/Cl)Cl | 180 | 2 | 2 | 2 | 2 | 2 | 2 |
| m-toluylic acid, 3-chloroprop-2-enyl ester (C11H11ClO2) | MBSMOYXJNIUOJL-ZZXKWVIFSA-N | CC1=CC=CC(=C1)C(=O)OC/C=C/Cl | 200 | 2 | 2 | 2 | 2 | 2 | 2 |
| Cyclopropanecarboxylic acid, 3-(2,2-dichloroethenyl)-2,2-dimethyl-, ethyl ester (C10H14Cl2O2) | QPTWKDNRYCGMJM-UHFFFAOYSA-N | CCOC(=O)C1C(C1(C)C)C=C(Cl)Cl | 379 | 2 | 2 | 2 | 2 | 2 | 2 |
| 3-cyclopentylpropionic acid, 3-chloroprop-2-enyl ester (C11H17ClO2) | QXWNSXGSUGCIHO-FPYGCLRLSA-N | C1CCC(C1)CCC(=O)OC/C=C/Cl | 190 | 2 | 2 | 2 | 2 | 2 | 2 |
| Naphthalene, 1,3,5,7-tetrachloro- (C10H4Cl4) | OTTCXKPQKOLSJN-UHFFFAOYSA-N | C1=C(C=C(C2=CC(=CC(=C21)Cl)Cl)Cl)Cl | 7837 | 1 | 1 | 1 | 1 | 1 | 1 |
| [1,1'-Biphenyl]-4-ol, 4'-chloro- (C12H9ClO) | ICVFJPSNAUMFCW-UHFFFAOYSA-N | C1=CC(=CC=C1C2=CC=C(C=C2)Cl)O | 9999 | 1 | 1 | 1 | 1 | 1 | 1 |
| PCB 4 (C12H8Cl2) | JAYCNKDKIKZTAF-UHFFFAOYSA-N | C1=CC=C(C(=C1)C2=CC=CC=C2Cl)Cl | 5909 | 1 | 1 | 1 | 1 | 1 | 1 |
| PCB 8 (C12H8Cl2) | UFNIBRDIUNVOMX-UHFFFAOYSA-N | C1=CC=C(C(=C1)C2=CC=C(C=C2)Cl)Cl | 8497 | 1 | 1 | 1 | 1 | 1 | 1 |
| 1,1'-Biphenyl, 4-(chloromethyl)- (C13H11Cl) | HLQZCRVEEQKNMS-UHFFFAOYSA-N | C1=CC=C(C=C1)C2=CC=C(C=C2)CCl | 2709 | 1 | 1 | 1 | 1 | 1 | 1 |
| Succinic acid, 2,2-dichloroethyl propyl ester (C9H14Cl2O4) | FJMPKWLUBAMQEB-UHFFFAOYSA-N | CCCOC(=O)CCC(=O)OCC(Cl)Cl | 0 | 2 | 2 | 2 | 2 | 2 | 2 |
| 7-Chlorodecanoic acid, chloromethyl ester (C11H20Cl2O2) | WOXCLOSQVCNINZ-UHFFFAOYSA-N | CCCC(CCCCCC(=O)OCCl)Cl | 0 | 2 | 2 | 2 | 2 | 2 | 2 |
| 2-Chlorodecanoic acid, chloromethyl ester (C11H20Cl2O2) | ZBWKSSOXFUXWPK-UHFFFAOYSA-N | CCCCCCCCC(C(=O)OCCl)Cl | 0 | 2 | 2 | 2 | 2 | 2 | 2 |
| Nonyl dichloroacetate (C11H20Cl2O2) | XKMMRMJBZDCIHI-UHFFFAOYSA-N | CCCCCCCCCOC(=O)C(Cl)Cl | 0 | 2 | 2 | 2 | 2 | 2 | 2 |
| 3-Chloropropionic acid, 2-methyloct-5-yn-4-yl ester (C12H19ClO2) | UZTJDQYZKQSKGR-UHFFFAOYSA-N | CCC#CC(CC(C)C)OC(=O)CCCl | 50 | 2 | 2 | 2 | 2 | 2 | 2 |
| Ethanimidamide, N-[(6-chloro-3-pyridinyl)methyl]-N'-cyano-N-methyl-, (1E)- (C10H11ClN4) | WCXDHFDTOYPNIE-UHFFFAOYSA-N | CC(=NC#N)N(C)CC1=CN=C(C=C1)Cl | 500 | 2 | 2 | 2 | 1 | 2 | 2 |
| Butanoic acid, 4-(2,4-dichlorophenoxy)- (C10H10Cl2O3) | YIVXMZJTEQBPQO-UHFFFAOYSA-N | C1=CC(=C(C=C1Cl)Cl)OCCCC(=O)O | 589 | 2 | 1 | 1 | 1 | 1 | 2 |
| Rose acetate (C10H9Cl3O2) | JKRWZLOCPLZZEI-UHFFFAOYSA-N | CC(=O)OC(C1=CC=CC=C1)C(Cl)(Cl)Cl | 0 | 2 | 2 | 2 | 2 | 1 | 2 |
| Butanoic acid, 4-(4-chloro-2-methylphenoxy)- (C10H13ClO3) | LLWADFLAOKUBDR-UHFFFAOYSA-N | CC1=C(C=CC(=C1)Cl)OCCCC(=O)O | 1419 | 1 | 1 | 1 | 2 | 1 | 2 |
| 5-Chloropentyl benzoate (C12H15ClO2) | XIOZPPYPTAEPGJ-UHFFFAOYSA-N | C1=CC=C(C=C1)C(=O)OCCCCCCl | 64 | 2 | 2 | 2 | 2 | 2 | 2 |
| Hexanamide, N-(3-chlorophenyl)- (C12H16ClNO) | BAUYPJBSRHYPFK-UHFFFAOYSA-N | CCCCCC(=O)NC1=CC(=CC=C1)Cl | 961 | 1 | 1 | 1 | 1 | 1 | 1 |
| Benzene, 1,1'-oxybis[4-chloro- (C12H8Cl2O) | URUJZHZLCCIILC-UHFFFAOYSA-N | C1=CC(=CC=C1OC2=CC=C(C=C2)Cl)Cl | 9999 | 1 | 1 | 1 | 1 | 1 | 1 |
| 1,1'-Biphenyl,3-chloro-4-methoxy- (C13H11ClO) | SLBYCCHSURAIIK-UHFFFAOYSA-N | COC1=C(C=C(C=C1)C2=CC=CC=C2)Cl | 9139 | 1 | 1 | 1 | 1 | 1 | 1 |
| PCB 33 (C12H7Cl3) | RIMXLXBUOQMDHV-UHFFFAOYSA-N | C1=CC=C(C(=C1)C2=CC(=C(C=C2)Cl)Cl)Cl | 9838 | 1 | 1 | 1 | 1 | 1 | 1 |
| 2-Chlorobenzoic acid, morpholide (C11H12ClNO2) | ZRGHSGNYLSCFJY-UHFFFAOYSA-N | C1COCCN1C(=O)C2=CC=CC=C2Cl | 601 | 2 | 1 | 1 | 2 | 1 | 2 |
| 4-Chlorobenzoic acid, morpholide (C11H12ClNO2) | BGRFQNTYHMHVFJ-UHFFFAOYSA-N | C1COCCN1C(=O)C2=CC=C(C=C2)Cl | 1621 | 1 | 1 | 1 | 2 | 1 | 2 |
| Bibenzyl, 3-chloro- (C14H13Cl) | NWVGKFZMBBPAJL-UHFFFAOYSA-N | C1=CC=C(C=C1)CCC2=CC(=CC=C2)Cl | 1451 | 1 | 1 | 1 | 1 | 1 | 1 |
| Cyclopentanecarboxamide, N-(3-chlorophenyl)- (C12H14ClNO) | YOKCBEZMIXZJTA-UHFFFAOYSA-N | C1CCC(C1)C(=O)NC2=CC(=CC=C2)Cl | 2212 | 1 | 1 | 1 | 1 | 1 | 1 |
| Methyl 4-chloroindolyl-3-acetate (C11H10ClNO2) | SYPGJEURLIGNPE-UHFFFAOYSA-N | COC(=O)CC1=CNC2=C1C(=CC=C2)Cl | 2350 | 1 | 1 | 1 | 1 | 1 | 1 |
| 6,8-Dichlorochromone-3-carboxaldehyde (C10H4Cl2O3) | IHCCHRKNCOFDAJ-UHFFFAOYSA-N | C1=C(C=C2C(=C1Cl)OC=C(C2=O)C=O)Cl | 358 | 2 | 1 | 1 | 2 | 1 | 1 |
| Heptenophos (C9H12ClO4P) | GBAWQJNHVWMTLU-UHFFFAOYSA-N | COP(=O)(OC)OC1=C(C2C1CC=C2)Cl | 940 | 1 | 2 | 2 | 2 | 2 | 2 |
| 5-Chloro-1,10-phenanthroline (C12H7ClN2) | XDUUQOQFSWSZSM-UHFFFAOYSA-N | C1=CC2=CC(=C3C=CC=NC3=C2N=C1)Cl | 9999 | 1 | 1 | 1 | 1 | 1 | 1 |
| Anthracene, 1-chloro- (C14H9Cl) | SRIHSAFSOOUEGL-UHFFFAOYSA-N | C1=CC=C2C=C3C(=CC2=C1)C=CC=C3Cl | 9999 | 1 | 1 | 1 | 1 | 1 | 1 |
| Succinic acid, di(2,2-dichloroethyl) ester (C8H10Cl4O4) | APJYUYGGYUZQET-UHFFFAOYSA-N | C(CC(=O)OCC(Cl)Cl)C(=O)OCC(Cl)Cl | 0 | 2 | 2 | 2 | 2 | 2 | 2 |
| 5-Chlorovaleric acid, octyl ester (C13H25ClO2) | KWLSFBAAGRONDK-UHFFFAOYSA-N | CCCCCCCCOC(=O)CCCCCl | 0 | 2 | 2 | 2 | 2 | 2 | 2 |
| Fumaric acid, 2,2-dichloroethyl isobutyl ester (C10H14Cl2O4) | IKJKPEUGXREZPB-ONEGZZNKSA-N | CC(C)COC(=O)/C=C/C(=O)OCC(Cl)Cl | 0 | 2 | 2 | 2 | 2 | 2 | 2 |
| Dichloroacetic acid, 2,7-dimethyloct-7-en-5-yn-4-yl (C12H16Cl2O2) | VSXCBHKTNKYJJP-UHFFFAOYSA-N | CC(C)CC(C#CC(=C)C)OC(=O)C(Cl)Cl | 330 | 2 | 2 | 2 | 2 | 2 | 2 |
| Diethyl 3-chlorophenyl phosphate (C10H14ClO4P) | OXUXCXDESUJMFE-UHFFFAOYSA-N | CCOP(=O)(OCC)OC1=CC(=CC=C1)Cl | 3493 | 1 | 2 | 1 | 1 | 1 | 1 |
| 2,3-Dichlorophenol, isoBOC (C11H12Cl2O3) | SJOZPBMTTPQALN-UHFFFAOYSA-N | CC(C)COC(=O)OC1=C(C(=CC=C1)Cl)Cl | 0 | 2 | 2 | 2 | 2 | 2 | 2 |
| 5-Chlorovaleric acid, 4-methoxyphenyl ester (C12H15ClO3) | KGAVHPXSHLZLNB-UHFFFAOYSA-N | COC1=CC=C(C=C1)OC(=O)CCCCCl | 280 | 2 | 2 | 2 | 2 | 2 | 2 |
| 4-N,N-Bis(2-chloroethyl)amino-2-tolualdehyde (C12H15Cl2NO) | ZQIAXDULHBLZJE-UHFFFAOYSA-N | CC1=C(C=CC(=C1)N(CCCl)CCCl)C=O | 1144 | 1 | 1 | 1 | 1 | 1 | 1 |
| Propyzamide (C12H11Cl2NO) | PHNUZKMIPFFYSO-UHFFFAOYSA-N | CC(C)(C#C)NC(=O)C1=CC(=CC(=C1)Cl)Cl | 2742 | 1 | 1 | 2 | 1 | 2 | 2 |
| Pentanochlor (C13H18ClNO) | WGVWLKXZBUVUAM-UHFFFAOYSA-N | CCCC(C)C(=O)NC1=CC(=C(C=C1)C)Cl | 1470 | 1 | 2 | 2 | 2 | 2 | 2 |
| 1-Chloromethyl-3,5-bis(1,1-dimethylethyl)benzene (C15H23Cl) | UNRGFCVSCXJGCL-UHFFFAOYSA-N | CC(C)(C)C1=CC(=CC(=C1)CCl)C(C)(C)C | 1700 | 1 | 1 | 1 | 1 | 1 | 2 |
| PCB 66 (C12H6Cl4) | RKLLTEAEZIJBAU-UHFFFAOYSA-N | C1=CC(=C(C=C1C2=C(C=C(C=C2)Cl)Cl)Cl)Cl | 7827 | 1 | 1 | 1 | 1 | 1 | 1 |
| PCB 77 (C12H6Cl4) | UQMGJOKDKOLIDP-UHFFFAOYSA-N | C1=CC(=C(C=C1C2=CC(=C(C=C2)Cl)Cl)Cl)Cl | 7470 | 1 | 1 | 1 | 1 | 1 | 1 |
| PCB 42 (C12H6Cl4) | ALFHIHDQSYXSGP-UHFFFAOYSA-N | C1=CC(=C(C(=C1)Cl)Cl)C2=C(C=C(C=C2)Cl)Cl | 8029 | 1 | 1 | 1 | 1 | 1 | 1 |
| PCB 40 (C12H6Cl4) | VTLYHLREPCPDKX-UHFFFAOYSA-N | C1=CC(=C(C(=C1)Cl)Cl)C2=C(C(=CC=C2)Cl)Cl | 6552 | 1 | 1 | 1 | 1 | 1 | 1 |
| PCB 79 (C12H6Cl4) | QLCTXEMDCZGPCG-UHFFFAOYSA-N | C1=CC(=C(C=C1C2=CC(=CC(=C2)Cl)Cl)Cl)Cl | 8008 | 1 | 1 | 1 | 1 | 1 | 1 |
| 2,4'-Dichlorobenzophenone (C13H8Cl2O) | YXMYPHLWXBXNFF-UHFFFAOYSA-N | C1=CC=C(C(=C1)C(=O)C2=CC=C(C=C2)Cl)Cl | 2139 | 1 | 1 | 1 | 1 | 1 | 1 |
| Diphenylacetyl chloride (C14H11ClO) | MSYLETHDEIJMAF-UHFFFAOYSA-N | C1=CC=C(C=C1)C(C2=CC=CC=C2)C(=O)Cl | 0 | 2 | 2 | 1 | 1 | 1 | 2 |
| Benzamide, N-tetrahydrofurfuryl-4-chloro- (C12H14ClNO2) | ZMMAVVGZVPYQOX-UHFFFAOYSA-N | C1CC(OC1)CNC(=O)C2=CC=C(C=C2)Cl | 130 | 2 | 2 | 1 | 2 | 1 | 2 |
| 1,1-Bis(4-chlorophenyl)ethylene (C14H10Cl2) | IEAUXBMXWDAYID-UHFFFAOYSA-N | C=C(C1=CC=C(C=C1)Cl)C2=CC=C(C=C2)Cl | 3800 | 1 | 1 | 1 | 1 | 1 | 1 |
| 2-Thiophenecarboxylic acid, 3,4-dichlorophenyl ester (C11H6Cl2O2S) | BTWJLTDNAKWSDS-UHFFFAOYSA-N | C1=CSC(=C1)C(=O)OC2=CC(=C(C=C2)Cl)Cl | 380 | 2 | 2 | 2 | 2 | 2 | 2 |
| Acetamide, N-(3-chlorophenyl)-2-(2-thienyl)- (C12H10ClNOS) | RTZYPCPHOITSSY-UHFFFAOYSA-N | C1=CC(=CC(=C1)Cl)NC(=O)CC2=CC=CS2 | 4724 | 1 | 1 | 1 | 1 | 1 | 1 |
| α-Chlordene (C10H6Cl6) | GSNLXLNDMLYEEK-UHFFFAOYSA-N | C1C2C3C(C1Cl)C(=C(C2(C(=C3Cl)Cl)Cl)Cl)Cl | 1879 | 1 | 1 | 1 | 1 | 1 | 1 |
| Chloroacetamide, N,N-dihexyl- (C14H28ClNO) | ZGQVQRKCUZEQLW-UHFFFAOYSA-N | CCCCCCN(CCCCCC)C(=O)CCl | 60 | 2 | 2 | 2 | 2 | 2 | 2 |
| 11-Chlorododecanoic acid, chloromethyl ester (C13H24Cl2O2) | SWJDHWAMRFKAJU-UHFFFAOYSA-N | CC(CCCCCCCCCC(=O)OCCl)Cl | 0 | 2 | 2 | 2 | 2 | 2 | 2 |
| 3-Chlorododecanoic acid, chloromethyl ester (C13H24Cl2O2) | GQHFPKLBEGPYQL-UHFFFAOYSA-N | CCCCCCCCCC(CC(=O)OCCl)Cl | 0 | 2 | 2 | 2 | 2 | 2 | 2 |
| 2-chloroethyl dodecanoate (C14H27ClO2) | PPRUSMUBWUQYRY-UHFFFAOYSA-N | CCCCCCCCCCCC(=O)OCCCl | 450 | 2 | 2 | 2 | 2 | 2 | 2 |
| Propanoic acid, 3-chloro, undecyl ester (C14H27ClO2) | MEBJHQDVCFHKMD-UHFFFAOYSA-N | CCCCCCCCCCCOC(=O)CCCl | 0 | 2 | 2 | 2 | 2 | 2 | 2 |
| Fumaric acid, isobutyl 2,2,2-trichloroethyl ester (C10H13Cl3O4) | VYCVEXWSKPJYKF-ONEGZZNKSA-N | CC(C)COC(=O)/C=C/C(=O)OCC(Cl)(Cl)Cl | 0 | 2 | 2 | 2 | 2 | 2 | 2 |
| Dichloroacetic acid, 2,6-dimethylnon-1-en-3-yn-5-yl ester (C13H18Cl2O2) | WQHIJAZVGMYLTQ-UHFFFAOYSA-N | CCCC(C)C(C#CC(=C)C)OC(=O)C(Cl)Cl | 0 | 2 | 2 | 2 | 2 | 2 | 2 |
| 3-Chloropropionic acid, 2,6-dimethylnon-1-en-3-yn-5-yl ester (C14H21ClO2) | NOIASPFJFXMZDP-UHFFFAOYSA-N | CCCC(C)C(C#CC(=C)C)OC(=O)CCCl | 80 | 2 | 2 | 2 | 2 | 2 | 2 |
| 2,4,5-TB methyl ester (C11H11Cl3O3) | OTBMAATXBNOQHO-UHFFFAOYSA-N | COC(=O)CCCOC1=CC(=C(C=C1Cl)Cl)Cl | 40 | 2 | 2 | 2 | 2 | 2 | 2 |
| 2-Chloro-4-nitro-N,N-dipropylaniline (C12H17ClN2O2) | OYJJPFCGGNTTTO-UHFFFAOYSA-N | CCCN(CCC)C1=C(C=C(C=C1)[N+](=O)[O-])Cl | 900 | 1 | 2 | 1 | 1 | 2 | 1 |
| 6-Chlorohexanoic acid, 4-methoxyphenyl ester (C13H17ClO3) | WIUNAWMQMZCBAR-UHFFFAOYSA-N | COC1=CC=C(C=C1)OC(=O)CCCCCCl | 260 | 2 | 2 | 2 | 2 | 2 | 2 |
| Butanilicaine (C13H19ClN2O) | VWYQKFLLGRBICZ-UHFFFAOYSA-N | CCCCNCC(=O)NC1=C(C=CC=C1Cl)C | 120 | 2 | 2 | 2 | 2 | 2 | 2 |
| Fenson (C12H9ClO2S) | SPJOZZSIXXJYBT-UHFFFAOYSA-N | C1=CC=C(C=C1)S(=O)(=O)OC2=CC=C(C=C2)Cl | 2215 | 1 | 1 | 1 | 1 | 1 | 1 |
| Diazene, bis(4-chlorophenyl)-, 1-oxide (C12H8Cl2N2O) | NMAZIJPSESMWSA-UHFFFAOYSA-N | C1=CC(=CC=C1N=[N+](C2=CC=C(C=C2)Cl)[O-])Cl | 8768 | 1 | 1 | 2 | 1 | 1 | 1 |
| bis(4-chlorophenyl) sulphone (C12H8Cl2O2S) | GPAPPPVRLPGFEQ-UHFFFAOYSA-N | C1=CC(=CC=C1S(=O)(=O)C2=CC=C(C=C2)Cl)Cl | 2132 | 1 | 1 | 1 | 1 | 1 | 1 |
| Tetrasul (C12H6Cl4S) | QUWSDLYBOVGOCW-UHFFFAOYSA-N | C1=CC(=CC=C1SC2=CC(=C(C=C2Cl)Cl)Cl)Cl | 5815 | 1 | 1 | 1 | 1 | 1 | 1 |
| PCB 84 (C12H5Cl5) | QVWUJLANSDKRAH-UHFFFAOYSA-N | C1=CC(=C(C(=C1)Cl)Cl)C2=C(C=CC(=C2Cl)Cl)Cl | 5723 | 1 | 1 | 1 | 1 | 1 | 1 |
| PCB 92 (C12H5Cl5) | CRCBRZBVCDKPGA-UHFFFAOYSA-N | C1=CC(=C(C=C1Cl)C2=CC(=CC(=C2Cl)Cl)Cl)Cl | 5848 | 1 | 1 | 1 | 1 | 1 | 1 |
| PCB 86 (C12H5Cl5) | AIURIRUDHVDRFQ-UHFFFAOYSA-N | C1=CC=C(C(=C1)C2=CC(=C(C(=C2Cl)Cl)Cl)Cl)Cl | 6241 | 1 | 1 | 1 | 1 | 1 | 1 |
| PCB 83 (C12H5Cl5) | SUBRHHYLRGOTHL-UHFFFAOYSA-N | C1=CC(=C(C(=C1)Cl)Cl)C2=CC(=CC(=C2Cl)Cl)Cl | 6341 | 1 | 1 | 1 | 1 | 1 | 1 |
| 1,1'-Biphenyl, 2,2',4,5',6-Pentachloro- (C12H5Cl5) | PQHZWWBJPCNNGI-UHFFFAOYSA-N | C1=CC(=C(C=C1Cl)C2=C(C=C(C=C2Cl)Cl)Cl)Cl | 6387 | 1 | 1 | 1 | 1 | 1 | 1 |
| PCB 114 (C12H5Cl5) | SXZSFWHOSHAKMN-UHFFFAOYSA-N | C1=CC(=CC=C1C2=CC(=C(C(=C2Cl)Cl)Cl)Cl)Cl | 6285 | 1 | 1 | 1 | 1 | 1 | 1 |
| Benzamide, N-(3-chlorophenyl)-4-methyl- (C14H12ClNO) | RUYDNAMXAAGBCA-UHFFFAOYSA-N | CC1=CC=C(C=C1)C(=O)NC2=CC(=CC=C2)Cl | 1692 | 1 | 1 | 1 | 1 | 1 | 1 |
| Benzamide, N-(3-methylphenyl)-2-chloro- (C14H12ClNO) | QMUDBMHUUBUXHG-UHFFFAOYSA-N | CC1=CC(=CC=C1)NC(=O)C2=CC=CC=C2Cl | 3693 | 1 | 1 | 1 | 1 | 1 | 1 |
| Benzhydryl 2-chloroethyl ether (C15H15ClO) | ZNVASENTCOLNJT-UHFFFAOYSA-N | C1=CC=C(C=C1)C(C2=CC=CC=C2)OCCCl | 3275 | 1 | 2 | 1 | 1 | 2 | 1 |
| Benzophenone, 2-methylamino-5-chloro- (C14H12ClNO) | YHSCBLSYYDIOFJ-UHFFFAOYSA-N | C1=CC=C(C=C1)C(=O)C2=C(C=CC(=C2)Cl)CN | 9999 | 1 | 1 | 2 | 1 | 2 | 1 |
| 3-Cyclopentylpropionic acid, 4-chlorophenyl ester (C14H17ClO2) | XVFXNEMYRISWTL-UHFFFAOYSA-N | C1CCC(C1)CCC(=O)OC2=CC=C(C=C2)Cl | 1762 | 1 | 2 | 2 | 1 | 1 | 1 |
| Heptachlor (C10H5Cl7) | FRCCEHPWNOQAEU-UHFFFAOYSA-N | C1=CC(C2C1C3(C(=C(C2(C3(Cl)Cl)Cl)Cl)Cl)Cl)Cl | 342 | 2 | 2 | 2 | 2 | 1 | 2 |
| Succinic acid, pentyl 2,2,2-trichloroethyl ester (C11H17Cl3O4) | SOSGHDULTDVXJY-UHFFFAOYSA-N | CCCCCOC(=O)CCC(=O)OCC(Cl)(Cl)Cl | 0 | 2 | 2 | 2 | 2 | 2 | 2 |
| Succinic acid, 2-chloropropyl isohexyl ester (C13H23ClO4) | WCTQPSDQUVPTMD-UHFFFAOYSA-N | CC(C)CCCOC(=O)CCC(=O)OCC(C)Cl | 0 | 2 | 2 | 2 | 2 | 2 | 2 |
| Propanamide, N,N-dihexyl-2-chloro- (C15H30ClNO) | CPIHTZXGVWMATK-UHFFFAOYSA-N | CCCCCCN(CCCCCC)C(=O)C(C)Cl | 150 | 2 | 2 | 2 | 2 | 2 | 2 |
| 5-Chlorovaleric acid, decyl ester (C15H29ClO2) | GDDOSYXFHTVPTR-UHFFFAOYSA-N | CCCCCCCCCCOC(=O)CCCCCl | 0 | 2 | 2 | 2 | 2 | 2 | 2 |
| Phenol, pentachloro-, trichloroacetate (C8Cl8O2) | WMUFBMFZOHGUPA-UHFFFAOYSA-N | C1(=C(C(=C(C(=C1Cl)Cl)Cl)Cl)Cl)OC(=O)C(Cl)(Cl)Cl | 60 | 2 | 1 | 2 | 2 | 1 | 2 |
| Hexanamide, N-ethyl-N-(3-methylphenyl)-6-chloro- (C15H22ClNO) | XOQPJEQXXHERBD-UHFFFAOYSA-N | CCN(C1=CC=CC(=C1)C)C(=O)CCCCCCl | 751 | 2 | 2 | 2 | 1 | 2 | 2 |
| Benzamide, N,N-dibutyl-4-chloro- (C15H22ClNO) | HBSQBZKONFNLBZ-UHFFFAOYSA-N | CCCCN(CCCC)C(=O)C1=CC=C(C=C1)Cl | 520 | 2 | 2 | 2 | 2 | 2 | 2 |
| Benzamide, N-(3-chlorophenyl)-2-methoxy- (C14H12ClNO2) | RLDGBZWAIDMLAE-UHFFFAOYSA-N | COC1=CC=CC=C1C(=O)NC2=CC(=CC=C2)Cl | 1491 | 1 | 1 | 1 | 1 | 1 | 1 |
| PCB 139 (C12H4Cl6) | SPOPSCCFZQFGDL-UHFFFAOYSA-N | C1=CC(=C(C=C1Cl)Cl)C2=C(C(=C(C=C2Cl)Cl)Cl)Cl | 4919 | 1 | 1 | 1 | 1 | 1 | 1 |
| PCB 147 (C12H4Cl6) | AQONCPKMJSBHQT-UHFFFAOYSA-N | C1=CC(=C(C=C1Cl)Cl)C2=C(C(=CC(=C2Cl)Cl)Cl)Cl | 5343 | 1 | 1 | 1 | 1 | 1 | 1 |
| 4-Chloro-3-nitrobenzophenone (C13H8ClNO3) | YBDBYPQFIMSFJW-UHFFFAOYSA-N | C1=CC=C(C=C1)C(=O)C2=CC(=C(C=C2)Cl)[N+](=O)[O-] | 2856 | 1 | 1 | 1 | 1 | 1 | 1 |
| 2-Phenylethyl 2-chlorobenzoate (C15H13ClO2) | FANZVPUMGJHASY-UHFFFAOYSA-N | C1=CC=C(C=C1)CCOC(=O)C2=CC=CC=C2Cl | 0 | 2 | 2 | 2 | 2 | 2 | 2 |
| o,p'-DDE (C14H8Cl4) | ZDYJWDIWLRZXDB-UHFFFAOYSA-N | C1=CC=C(C(=C1)C(=C(Cl)Cl)C2=CC=C(C=C2)Cl)Cl | 3238 | 1 | 1 | 1 | 1 | 1 | 1 |
| 9,10-Anthracenedione, 1,8-dichloro- (C14H6Cl2O2) | VBQNYYXVDQUKIU-UHFFFAOYSA-N | C1=CC2=C(C(=C1)Cl)C(=O)C3=C(C2=O)C=CC=C3Cl | 9999 | 1 | 1 | 1 | 1 | 1 | 1 |
| Isobenzan (C9H4Cl8O) | LRWHHSXTGZSMSN-UHFFFAOYSA-N | C12C(C(OC1Cl)Cl)C3(C(=C(C2(C3(Cl)Cl)Cl)Cl)Cl)Cl | 103 | 2 | 2 | 2 | 2 | 2 | 2 |
| Succinic acid, 2,2-dichloroethyl heptyl ester (C13H22Cl2O4) | XYHOOJUYXZQYME-UHFFFAOYSA-N | CCCCCCCOC(=O)CCC(=O)OCC(Cl)Cl | 0 | 2 | 2 | 2 | 2 | 2 | 2 |
| Malonic acid, 8-chlorooctyl propyl ester (C14H25ClO4) | CSXNAOIJZYGGFP-UHFFFAOYSA-N | CCCOC(=O)CC(=O)OCCCCCCCCCl | 0 | 2 | 2 | 2 | 2 | 2 | 2 |
| Tetradecyl chloroacetate (C16H31ClO2) | INPWKHSGGJNIIM-UHFFFAOYSA-N | CCCCCCCCCCCCCCOC(=O)CCl | 0 | 2 | 2 | 2 | 2 | 2 | 2 |
| 5-chlorovaleric acid, undec-2-enyl ester (C16H29ClO2) | MMBYQPWSFIURFR-FMIVXFBMSA-N | CCCCCCCC/C=C/COC(=O)CCCCCl | 90 | 2 | 2 | 2 | 2 | 2 | 2 |
| Succinic acid, ethyl 2,3,5-trichlorophenyl ester (C12H11Cl3O4) | DMGJAPZJPKIMQE-UHFFFAOYSA-N | CCOC(=O)CCC(=O)OC1=CC(=CC(=C1Cl)Cl)Cl | 0 | 2 | 2 | 2 | 2 | 2 | 2 |
| Succinic acid, ethyl 2,3,6-trichlorophenyl ester (C12H11Cl3O4) | VQAWDMHUBWVGEG-UHFFFAOYSA-N | CCOC(=O)CCC(=O)OC1=C(C=CC(=C1Cl)Cl)Cl | 0 | 2 | 2 | 2 | 2 | 2 | 2 |
| β-Alanine, N-(2-chlorobenzoyl)-, butyl ester (C14H18ClNO3) | VUQQMELHTPWSLV-UHFFFAOYSA-N | CCCCOC(=O)CCNC(=O)C1=CC=CC=C1Cl | 831 | 1 | 2 | 2 | 2 | 2 | 2 |
| Fumaric acid, ethyl 3,4,5-trichlorophenyl ester (C12H9Cl3O4) | NPYGPOLDZDOIEX-ONEGZZNKSA-N | CCOC(=O)/C=C/C(=O)OC1=CC(=C(C(=C1)Cl)Cl)Cl | 70 | 2 | 2 | 2 | 2 | 2 | 2 |
| Fumaric acid, ethyl 2,4,6-trichlorophenyl ester (C12H9Cl3O4) | ZQUKPRSELNYMAD-ONEGZZNKSA-N | CCOC(=O)/C=C/C(=O)OC1=C(C=C(C=C1Cl)Cl)Cl | 70 | 2 | 2 | 2 | 2 | 2 | 2 |
| PCB 189 (C12H3Cl7) | XUAWBXBYHDRROL-UHFFFAOYSA-N | C1=C(C=C(C(=C1Cl)Cl)Cl)C2=CC(=C(C(=C2Cl)Cl)Cl)Cl | 3585 | 1 | 1 | 1 | 1 | 1 | 1 |
| PCB 178 (C12H3Cl7) | WCIBKXHMIXUQHK-UHFFFAOYSA-N | C1=C(C=C(C(=C1Cl)Cl)C2=C(C(=CC(=C2Cl)Cl)Cl)Cl)Cl | 3938 | 1 | 1 | 1 | 1 | 1 | 1 |
| 2,6-Dichlorobenzyl ether (C14H10Cl4O) | NWYHVMDKERUNLM-UHFFFAOYSA-N | C1=CC(=C(C=C1Cl)Cl)COCC2=C(C=C(C=C2)Cl)Cl | 120 | 2 | 2 | 2 | 2 | 1 | 2 |
| Pyrifenox (C14H12Cl2N2O) | CKPCAYZTYMHQEX-UHFFFAOYSA-N | CON=C(CC1=CN=CC=C1)C2=C(C=C(C=C2)Cl)Cl | 1702 | 1 | 1 | 1 | 1 | 1 | 1 |
| 2-amino-5-chlorobenzophenone, acetylated (C15H12ClNO2) | NHAUKYAYIYDFST-UHFFFAOYSA-N | CC(=O)NC1=C(C=C(C=C1)Cl)C(=O)C2=CC=CC=C2 | 1170 | 1 | 1 | 1 | 1 | 1 | 1 |
| 3-Phenylpropionic acid, 3,4-dichlorophenyl ester (C15H12Cl2O2) | FQOCOCXQBBVPNZ-UHFFFAOYSA-N | C1=CC=C(C=C1)CCC(=O)OC2=CC(=C(C=C2)Cl)Cl | 601 | 2 | 2 | 2 | 1 | 2 | 2 |
| 1,2,4,7,8-Pentachlorodibenzo-p-dioxin (C12H3Cl5O2) | QUPLGUUISJOUPJ-UHFFFAOYSA-N | C1=C2C(=CC(=C1Cl)Cl)OC3=C(O2)C(=CC(=C3Cl)Cl)Cl | 6260 | 1 | 1 | 1 | 1 | 1 | 1 |
| Medazepam (C16H15ClN2) | YLCXGBZIZBEVPZ-UHFFFAOYSA-N | CN1CCN=C(C2=C1C=CC(=C2)Cl)C3=CC=CC=C3 | 3309 | 1 | 1 | 1 | 1 | 1 | 1 |
| Chlorflurenol, methyl ester (C15H11ClO3) | LINPVWIEWJTEEJ-UHFFFAOYSA-N | COC(=O)C1(C2=CC=CC=C2C3=C1C=C(C=C3)Cl)O | 1111 | 1 | 1 | 1 | 1 | 1 | 1 |
| Endosulfan (C9H6Cl6O3S) | RDYMFSUJUZBWLH-UHFFFAOYSA-N | C1C2C(COS(=O)O1)C3(C(=C(C2(C3(Cl)Cl)Cl)Cl)Cl)Cl | 284 | 2 | 2 | 2 | 2 | 2 | 2 |
| Oxychlordane (C10H4Cl8O) | VWGNQYSIWFHEQU-UHFFFAOYSA-N | C12C(C(C3(C1O3)Cl)Cl)C4(C(=C(C2(C4(Cl)Cl)Cl)Cl)Cl)Cl | 0 | 2 | 2 | 2 | 2 | 2 | 2 |
| Tris(1,3-dichioro-2-propyl) phosphate (C9H15Cl6O4P) | ASLWPAWFJZFCKF-UHFFFAOYSA-N | C(C(CCl)OP(=O)(OC(CCl)CCl)OC(CCl)CCl)Cl | 0 | 2 | 2 | 2 | 2 | 2 | 2 |
| Succinic acid, heptyl 2,2,2-trichloroethyl ester (C13H21Cl3O4) | NAQVIHHSOMSWFF-UHFFFAOYSA-N | CCCCCCCOC(=O)CCC(=O)OCC(Cl)(Cl)Cl | 0 | 2 | 2 | 2 | 2 | 2 | 2 |
| Fumaric acid, 8-chlorooctyl propyl ester (C15H25ClO4) | MGHZNNGUVBDAGD-MDZDMXLPSA-N | CCCOC(=O)/C=C/C(=O)OCCCCCCCCCl | 0 | 2 | 2 | 2 | 2 | 2 | 2 |
| Succinic acid, propyl 2,3,6-trichlorophenyl ester (C13H13Cl3O4) | ULGNTESKJOCJGR-UHFFFAOYSA-N | CCCOC(=O)CCC(=O)OC1=C(C=CC(=C1Cl)Cl)Cl | 0 | 2 | 2 | 2 | 2 | 2 | 2 |
| β-Alanine, N-(4-chlorobenzoyl)-, pentyl ester (C15H20ClNO3) | AEPLOPPDIMQBKI-UHFFFAOYSA-N | CCCCCOC(=O)CCNC(=O)C1=CC=C(C=C1)Cl | 1161 | 1 | 2 | 2 | 1 | 1 | 1 |
| Benzoic acid, 2-chloro, decyl ester (C17H25ClO2) | XWNLBSXKHJVMLC-UHFFFAOYSA-N | CCCCCCCCCCOC(=O)C1=CC=CC=C1Cl | 80 | 2 | 2 | 2 | 2 | 2 | 2 |
| Benzamide, N-(2,5-dimethoxyphenyl)-4-chloro- (C15H14ClNO3) | HCVYUDNPZQUVRH-UHFFFAOYSA-N | COC1=CC(=C(C=C1)OC)NC(=O)C2=CC=C(C=C2)Cl | 5225 | 1 | 1 | 1 | 1 | 1 | 1 |
| Dichloroacetic acid, 4-benzyloxyphenyl ester (C15H12Cl2O3) | BHDHMFXSIYFMCZ-UHFFFAOYSA-N | C1=CC=C(C=C1)COC2=CC=C(C=C2)OC(=O)C(Cl)Cl | 370 | 2 | 1 | 1 | 1 | 1 | 1 |
| cis-Captafol (C10H9Cl4NO2S) | JHRWWRDRBPCWTF-OLQVQODUSA-N | C1C=CC[C@H]2[C@@H]1C(=O)N(C2=O)SC(C(Cl)Cl)(Cl)Cl | 40 | 2 | 2 | 2 | 2 | 2 | 2 |
| Dienochlor (C10Cl10) | LWLJUMBEZJHXHV-UHFFFAOYSA-N | C1(=C(C(C(=C1Cl)Cl)(C2(C(=C(C(=C2Cl)Cl)Cl)Cl)Cl)Cl)Cl)Cl | 117 | 2 | 2 | 2 | 1 | 2 | 2 |
| 1-Naphthoic acid, 4-chlorophenyl ester (C17H11ClO2) | ZTNAIEDTVGAUOA-UHFFFAOYSA-N | C1=CC=C2C(=C1)C=CC=C2C(=O)OC3=CC=C(C=C3)Cl | 200 | 2 | 2 | 2 | 1 | 1 | 1 |
| 7-Aminoclonazepam (C15H12ClN3O) | HEFRPWRJTGLSSV-UHFFFAOYSA-N | C1C(=O)NC2=C(C=C(C=C2)N)C(=N1)C3=CC=CC=C3Cl | 9999 | 1 | 1 | 1 | 1 | 1 | 1 |
| Diazepam (C16H13ClN2O) | AAOVKJBEBIDNHE-UHFFFAOYSA-N | CN1C(=O)CN=C(C2=C1C=CC(=C2)Cl)C3=CC=CC=C3 | 7222 | 1 | 1 | 1 | 1 | 1 | 1 |
| Adenosine, 2-chloro- (C10H12ClN5O4) | BIXYYZIIJIXVFW-UHFFFAOYSA-N | C1=NC2=C(N1C3C(C(C(O3)CO)O)O)N=C(N=C2N)Cl | 588 | 2 | 2 | 2 | 2 | 2 | 2 |
| Cyclopropanecarbonitrile, 1-(p-chlorophenyl)-2-(p-methoxyphenyl)- (C17H14ClNO) | UKBFLNJUMCRCGX-UHFFFAOYSA-N | COC1=CC=C(C=C1)C2CC2(C#N)C3=CC=C(C=C3)Cl | 9999 | 1 | 1 | 1 | 2 | 1 | 1 |
| Trichloroacetamide, N,N-diheptyl- (C16H30Cl3NO) | BVFXHEMDRLSTLE-UHFFFAOYSA-N | CCCCCCCN(CCCCCCC)C(=O)C(Cl)(Cl)Cl | 0 | 2 | 2 | 2 | 2 | 2 | 2 |
| 2-chloroethyl hexadecanoate (C18H35ClO2) | CPFFARIYTPCNJA-UHFFFAOYSA-N | CCCCCCCCCCCCCCCC(=O)OCCCl | 750 | 2 | 2 | 2 | 2 | 2 | 2 |
| 5-Chlorovaleric acid, tridecyl ester (C18H35ClO2) | QUAVOGDQRCBTGN-UHFFFAOYSA-N | CCCCCCCCCCCCCOC(=O)CCCCCl | 0 | 2 | 2 | 2 | 2 | 2 | 2 |
| 5-Chlorovaleric acid, 2-tridecyl ester (C18H35ClO2) | CMCIREZBJQDCSX-UHFFFAOYSA-N | CCCCCCCCCCCC(C)OC(=O)CCCCCl | 0 | 2 | 2 | 2 | 2 | 2 | 2 |
| Chloroacetamide, N,N-dioctyl- (C18H36ClNO) | ASYPQLBYSSYEBE-UHFFFAOYSA-N | CCCCCCCCN(CCCCCCCC)C(=O)CCl | 50 | 2 | 2 | 2 | 2 | 2 | 2 |
| 5-Chlorovaleric acid, 5-tridecyl ester (C18H35ClO2) | MEVZAAUZXNKQIB-UHFFFAOYSA-N | CCCCCCCCC(CCCC)OC(=O)CCCCCl | 0 | 2 | 2 | 2 | 2 | 2 | 2 |
| Propanamide, N-heptyl-N-octyl-2-chloro- (C18H36ClNO) | QBZXHNDGBKWEQP-UHFFFAOYSA-N | CCCCCCCCN(CCCCCCC)C(=O)C(C)Cl | 230 | 2 | 2 | 2 | 2 | 2 | 2 |
| 5-chlorovaleric acid, tridec-2-ynyl ester (C18H31ClO2) | IWBWGAOOVXOGLS-UHFFFAOYSA-N | CCCCCCCCCCC#CCOC(=O)CCCCCl | 0 | 2 | 2 | 2 | 2 | 2 | 2 |
| Fumaric acid, 8-chlorooctyl isobutyl ester (C16H27ClO4) | MRHRYQMLUYULBY-MDZDMXLPSA-N | CC(C)COC(=O)/C=C/C(=O)OCCCCCCCCCl | 0 | 2 | 2 | 2 | 2 | 2 | 2 |
| 2,4,5-T Butoxyethyl ester (C14H17Cl3O4) | GLDWASBMYWLQGG-UHFFFAOYSA-N | CCCCOCCOC(=O)COC1=CC(=C(C=C1Cl)Cl)Cl | 1763 | 1 | 1 | 1 | 1 | 1 | 1 |
| Sarcosine, N-(2-chlorobenzoyl)-, hexyl ester (C16H22ClNO3) | CCRNYVLCQLBOBV-UHFFFAOYSA-N | CCCCCCOC(=O)CN(C)C(=O)C1=CC=CC=C1Cl | 1561 | 1 | 1 | 1 | 1 | 1 | 1 |
| Sarcosine, N-(4-chlorobenzoyl)-, isohexyl ester (C16H22ClNO3) | QKNRAHQLPYVFFY-UHFFFAOYSA-N | CC(C)CCCOC(=O)CN(C)C(=O)C1=CC=C(C=C1)Cl | 1141 | 1 | 1 | 1 | 1 | 1 | 1 |
| Aramite (C15H23ClO4S) | YKFRAOGHWKADFJ-UHFFFAOYSA-N | CC(COC1=CC=C(C=C1)C(C)(C)C)OS(=O)OCCCl | 450 | 2 | 1 | 2 | 1 | 2 | 1 |
| Succinic acid, 3,5-dichlorophenyl pentyl ester (C15H18Cl2O4) | TXRLZUNUCPQBIJ-UHFFFAOYSA-N | CCCCCOC(=O)CCC(=O)OC1=CC(=CC(=C1)Cl)Cl | 0 | 2 | 2 | 2 | 2 | 2 | 2 |
| Pretilachlor (C17H26ClNO2) | YLPGTOIOYRQOHV-UHFFFAOYSA-N | CCCOCCN(C1=C(C=CC=C1CC)CC)C(=O)CCl | 360 | 2 | 2 | 2 | 1 | 2 | 2 |
| Fumaric acid, propyl 2,3,4,5-tetrachlorophenyl ester (C13H10Cl4O4) | AEDTXMHHILJJGC-ONEGZZNKSA-N | CCCOC(=O)/C=C/C(=O)OC1=CC(=C(C(=C1Cl)Cl)Cl)Cl | 20 | 2 | 2 | 2 | 2 | 2 | 2 |
| Fumaric acid, isobutyl 3,4,5-trichlorophenyl ester (C14H13Cl3O4) | ZYNNZTCANZASBH-ONEGZZNKSA-N | CC(C)COC(=O)/C=C/C(=O)OC1=CC(=C(C(=C1)Cl)Cl)Cl | 60 | 2 | 2 | 2 | 2 | 2 | 2 |
| Fumaric acid, isobutyl 2,4,6-trichlorophenyl ester (C14H13Cl3O4) | JJOFXKGOYZAVLR-ONEGZZNKSA-N | CC(C)COC(=O)/C=C/C(=O)OC1=C(C=C(C=C1Cl)Cl)Cl | 50 | 2 | 2 | 2 | 2 | 2 | 2 |
| Bis(3-chlorophenylsulphonyl)methane (C13H10Cl2O4S2) | OWITURUKFUPLIO-UHFFFAOYSA-N | C1=CC(=CC(=C1)Cl)S(=O)(=O)CS(=O)(=O)C2=CC(=CC=C2)Cl | 410 | 2 | 2 | 2 | 2 | 2 | 2 |
| PCB 206 (C12HCl9) | JFIMDKGRGPNPRQ-UHFFFAOYSA-N | C1=C(C(=C(C(=C1Cl)Cl)Cl)Cl)C2=C(C(=C(C(=C2Cl)Cl)Cl)Cl)Cl | 0 | 2 | 1 | 1 | 1 | 1 | 1 |
| o,p'-Methoxychlor (C16H15Cl3O2) | KNLLPAOBVIKLDE-UHFFFAOYSA-N | COC1=CC=C(C=C1)C(C2=CC=CC=C2OC)C(Cl)(Cl)Cl | 410 | 2 | 2 | 2 | 2 | 2 | 2 |
| tebuconazole (C16H22ClN3O) | PXMNMQRDXWABCY-UHFFFAOYSA-N | CC(C)(C)C(CCC1=CC=C(C=C1)Cl)(CN2C=NC=N2)O | 150 | 2 | 2 | 2 | 2 | 2 | 2 |
| 1-Naphthoic acid, 3,4-dichlorophenyl ester (C17H10Cl2O2) | OMRXSGMGDYFBMY-UHFFFAOYSA-N | C1=CC=C2C(=C1)C=CC=C2C(=O)OC3=CC(=C(C=C3)Cl)Cl | 100 | 2 | 2 | 2 | 1 | 2 | 1 |
| 1H-1,2,4-Triazole, 1-[[2-(2,4-dichlorophenyl)-4-ethyl-1,3-dioxolan-2-yl]methyl]- (C14H15Cl2N3O2) | DWRKFAJEBUWTQM-UHFFFAOYSA-N | CCC1COC(O1)(CN2C=NC=N2)C3=C(C=C(C=C3)Cl)Cl | 0 | 2 | 2 | 2 | 2 | 2 | 2 |
| trans-Nonachlor (C10H5Cl9) | OCHOKXCPKDPNQU-BBXWSCHTSA-N | [C@@H]12[C@@H](C(C(C1Cl)Cl)Cl)C3(C(=C(C2(C3(Cl)Cl)Cl)Cl)Cl)Cl | 80 | 2 | 2 | 2 | 2 | 2 | 2 |
| 2-chlorobenzoic acid, 1-adamantylmethyl ester (C18H21ClO2) | KYXXVMRHEPJPPY-UHFFFAOYSA-N | C1C2CC3CC1CC(C2)(C3)COC(=O)C4=CC=CC=C4Cl | 170 | 2 | 2 | 2 | 2 | 2 | 2 |
| Succinic acid, decyl 2,2-dichloroethyl ester (C16H28Cl2O4) | SMHJYHBHJCREHK-UHFFFAOYSA-N | CCCCCCCCCCOC(=O)CCC(=O)OCC(Cl)Cl | 0 | 2 | 2 | 2 | 2 | 2 | 2 |
| Malonic acid, 2-chloropropyl undecyl ester (C17H31ClO4) | XNRXLJXCPKUKHW-UHFFFAOYSA-N | CCCCCCCCCCCOC(=O)CC(=O)OCC(C)Cl | 0 | 2 | 2 | 2 | 2 | 2 | 2 |
| Propanamide, N,N-dioctyl-3-chloro- (C19H38ClNO) | SLCJNBBZBLGJLW-UHFFFAOYSA-N | CCCCCCCCN(CCCCCCCC)C(=O)CCCl | 60 | 2 | 2 | 2 | 2 | 2 | 2 |
| Propanamide, N,N-bis(2-ethylhexyl)-3-chloro- (C19H38ClNO) | LJNYXXQVVFXPTJ-UHFFFAOYSA-N | CCCCC(CC)CN(CC(CC)CCCC)C(=O)CCCl | 60 | 2 | 2 | 2 | 2 | 2 | 2 |
| 2-chlorobenzoic acid, dodec-9-ynyl ester (C19H25ClO2) | SUDQXLLPAJDXDC-UHFFFAOYSA-N | CCC#CCCCCCCCCOC(=O)C1=CC=CC=C1Cl | 0 | 2 | 2 | 2 | 2 | 2 | 2 |
| Fumaric acid, isobutyl 2,3,4,6-tetrachlorophenyl ester (C14H12Cl4O4) | URUVAKSJVJOFGY-ONEGZZNKSA-N | CC(C)COC(=O)/C=C/C(=O)OC1=C(C(=C(C=C1Cl)Cl)Cl)Cl | 50 | 2 | 2 | 2 | 2 | 2 | 2 |
| Fumaric acid, isobutyl 2,3,4,5-tetrachlorophenyl ester (C14H12Cl4O4) | LSWQEYJKXOOGPU-ONEGZZNKSA-N | CC(C)COC(=O)/C=C/C(=O)OC1=CC(=C(C(=C1Cl)Cl)Cl)Cl | 20 | 2 | 2 | 2 | 2 | 2 | 2 |
| Fumaric acid, pentyl 3,4,5-trichlorophenyl ester (C15H15Cl3O4) | ALDAXBNYLCOBDS-AATRIKPKSA-N | CCCCCOC(=O)/C=C/C(=O)OC1=CC(=C(C(=C1)Cl)Cl)Cl | 30 | 2 | 2 | 2 | 2 | 2 | 2 |
| Fumaric acid, 3,5-dichlorophenyl isohexyl ester (C16H18Cl2O4) | OHLCSBOWTPMGNR-AATRIKPKSA-N | CC(C)CCCOC(=O)/C=C/C(=O)OC1=CC(=CC(=C1)Cl)Cl | 20 | 2 | 2 | 2 | 2 | 2 | 2 |
| Bifenox (C14H9Cl2NO3) | SUSRORUBZHMPCO-UHFFFAOYSA-N | COC(=O)C1=C(C=CC(=C1)OC2=C(C=C(C=C2)Cl)Cl)[N+](=O)[O-] | 9999 | 1 | 2 | 1 | 1 | 1 | 1 |
| Tris(4-chlorophenyl)phosphine (C18H12Cl3P) | IQKSLJOIKWOGIZ-UHFFFAOYSA-N | C1=CC(=CC=C1P(C2=CC=C(C=C2)Cl)C3=CC=C(C=C3)Cl)Cl | 9549 | 1 | 1 | 1 | 1 | 1 | 1 |
| Alprazolam (C17H13ClN4) | VREFGVBLTWBCJP-UHFFFAOYSA-N | CC1=NN=C2N1C3=C(C=C(C=C3)Cl)C(=NC2)C4=CC=CC=C4 | 7326 | 1 | 1 | 1 | 1 | 1 | 1 |
| Succinic acid, decyl 2,2,2-trichloroethyl ester (C16H27Cl3O4) | FAIYXLQCQHTADH-UHFFFAOYSA-N | CCCCCCCCCCOC(=O)CCC(=O)OCC(Cl)(Cl)Cl | 0 | 2 | 2 | 2 | 2 | 2 | 2 |
| 1-chlorodocosane (C22H45Cl) | OACXFSZVCDOBKF-UHFFFAOYSA-N | CCCCCCCCCCCCCCCCCCCCCCCl | 499 | 2 | 2 | 2 | 2 | 2 | 2 |
| Adipic acid, butyl 8-chloroctyl ester (C18H33ClO4) | HETXEZMDDSWKRG-UHFFFAOYSA-N | CCCCOC(=O)CCCCC(=O)OCCCCCCCCCl | 0 | 2 | 2 | 2 | 2 | 2 | 2 |
| 5-Chlorovaleric acid, 5-pentadecyl ester (C20H39ClO2) | WRDBNJHXOVIVGG-UHFFFAOYSA-N | CCCCCCCCCCC(CCCC)OC(=O)CCCCCl | 0 | 2 | 2 | 2 | 2 | 2 | 2 |
| Succinic acid, 10-chlorodecyl isobutyl ester (C18H33ClO4) | JLLUCWADVCLWOF-UHFFFAOYSA-N | CC(C)COC(=O)CCC(=O)OCCCCCCCCCCCl | 0 | 2 | 2 | 2 | 2 | 2 | 2 |
| Adipic acid, 8-chloroctyl isobutyl ester (C18H33ClO4) | MNXUVEYWINNEBR-UHFFFAOYSA-N | CC(C)COC(=O)CCCCC(=O)OCCCCCCCCCl | 0 | 2 | 2 | 2 | 2 | 2 | 2 |
| Trichloroacetamide, N,N-bis(2-ethylhexyl)- (C18H34Cl3NO) | AQTKUBSOCLDIJW-UHFFFAOYSA-N | CCCCC(CC)CN(CC(CC)CCCC)C(=O)C(Cl)(Cl)Cl | 0 | 2 | 2 | 2 | 2 | 2 | 2 |
| Succinic acid, isohexyl 2,3,5-trichlorophenyl ester (C16H19Cl3O4) | QBLWHUHQTUEOND-UHFFFAOYSA-N | CC(C)CCCOC(=O)CCC(=O)OC1=CC(=CC(=C1Cl)Cl)Cl | 0 | 2 | 2 | 2 | 2 | 2 | 2 |
| 2-Chlorobenzoic acid, tridec-2-ynyl ester (C20H27ClO2) | RDJRHBFOTCUIHH-UHFFFAOYSA-N | CCCCCCCCCCC#CCOC(=O)C1=CC=CC=C1Cl | 0 | 2 | 2 | 2 | 2 | 2 | 2 |
| 2-Chlorobenzoic acid, 3-tridecyl ester (C20H31ClO2) | STPUHZZTPBQLLR-UHFFFAOYSA-N | CCCCCCCCCCC(CC)OC(=O)C1=CC=CC=C1Cl | 0 | 2 | 2 | 2 | 2 | 2 | 2 |
| 2-Chlorobenzoic acid, 4-tridecyl ester (C20H31ClO2) | AJBVRWQRFTWIJO-UHFFFAOYSA-N | CCCCCCCCCC(CCC)OC(=O)C1=CC=CC=C1Cl | 0 | 2 | 2 | 2 | 2 | 2 | 2 |
| Fumaric acid, isobutyl pentachlorophenyl ester (C14H11Cl5O4) | RRORQPBYPGTCSN-ONEGZZNKSA-N | CC(C)COC(=O)/C=C/C(=O)OC1=C(C(=C(C(=C1Cl)Cl)Cl)Cl)Cl | 30 | 2 | 2 | 2 | 2 | 2 | 2 |
| Fumaric acid, isohexyl 3,4,5-trichlorophenyl ester (C16H17Cl3O4) | HDCIXOJTYKFIFX-AATRIKPKSA-N | CC(C)CCCOC(=O)/C=C/C(=O)OC1=CC(=C(C(=C1)Cl)Cl)Cl | 30 | 2 | 2 | 2 | 2 | 2 | 2 |
| Chlorsulfuron (C12H12ClN5O4S) | VJYIFXVZLXQVHO-UHFFFAOYSA-N | CC1=NC(=NC(=N1)OC)NC(=O)NS(=O)(=O)C2=CC=CC=C2Cl | 79 | 2 | 1 | 1 | 1 | 1 | 2 |
| 9H-Purine-9-acetic acid, 6-[(p-chlorophenyl)amino)-, ethyl ester (C15H14ClN5O2) | GRZHGZVUENNXNQ-UHFFFAOYSA-N | CCOC(=O)CN1C=NC2=C1N=CN=C2NC3=CC=C(C=C3)Cl | 9999 | 1 | 1 | 1 | 1 | 1 | 2 |
| 6-Chloro-N,N'-(cycloheptyl)-[1,3,5]triazine-2,4-diamine (C17H28ClN5) | NDHBZABQERFFOL-UHFFFAOYSA-N | C1CCCC(CC1)NC2=NC(=NC(=N2)Cl)NC3CCCCCC3 | 4978 | 1 | 1 | 1 | 1 | 1 | 1 |
| Amoxapine M (7-hydroxy) (C17H16ClN3O2) | MEUGUMOVYNSGEW-UHFFFAOYSA-N | C1CN(CCN1)C2=NC3=C(C=C(C=C3)O)OC4=C2C=C(C=C4)Cl | 1121 | 1 | 2 | 2 | 2 | 1 | 2 |
| Oxazolam (C18H17ClN2O2) | VCCZBYPHZRWKFY-UHFFFAOYSA-N | CC1CN2CC(=O)NC3=C(C2(O1)C4=CC=CC=C4)C=C(C=C3)Cl | 70 | 2 | 2 | 1 | 2 | 2 | 1 |
| Propanoic acid, 3-chloro, octadecyl ester (C21H41ClO2) | IWNDNJGFFCNPKN-UHFFFAOYSA-N | CCCCCCCCCCCCCCCCCCOC(=O)CCCl | 0 | 2 | 2 | 2 | 2 | 2 | 2 |
| Adipic acid, 8-chloroctyl pentyl ester (C19H35ClO4) | IIDRUMFLPGVZJE-UHFFFAOYSA-N | CCCCCOC(=O)CCCCC(=O)OCCCCCCCCCl | 0 | 2 | 2 | 2 | 2 | 2 | 2 |
| Propanamide, N,N-dinonyl-3-chloro- (C21H42ClNO) | IXOQRAVVQUMNPF-UHFFFAOYSA-N | CCCCCCCCCN(CCCCCCCCC)C(=O)CCCl | 50 | 2 | 2 | 2 | 2 | 2 | 2 |
| Malonic acid, 2-chloropropyl tridecyl ester (C19H35ClO4) | XQULABOIZJGPFB-UHFFFAOYSA-N | CCCCCCCCCCCCCOC(=O)CC(=O)OCC(C)Cl | 0 | 2 | 2 | 2 | 2 | 2 | 2 |
| Propanamide, N,N-dinonyl-2-chloro- (C21H42ClNO) | CAYVAIPTSFKUIT-UHFFFAOYSA-N | CCCCCCCCCN(CCCCCCCCC)C(=O)C(C)Cl | 200 | 2 | 2 | 2 | 2 | 2 | 2 |
| Fumaric acid, 2,2-dichloroethyl dodecyl ester (C18H30Cl2O4) | IDWSWXWSQPMUQF-OUKQBFOZSA-N | CCCCCCCCCCCCOC(=O)/C=C/C(=O)OCC(Cl)Cl | 0 | 2 | 2 | 2 | 2 | 2 | 2 |
| Fumaric acid, 10-chlorodecyl pentyl ester (C19H33ClO4) | AHHWRJVIUFWBFV-BUHFOSPRSA-N | CCCCCOC(=O)/C=C/C(=O)OCCCCCCCCCCCl | 0 | 2 | 2 | 2 | 2 | 2 | 2 |
| Fumaric acid, 2-chloropropyl dodecyl ester (C19H33ClO4) | KBMFXTYTNXLVHL-BUHFOSPRSA-N | CCCCCCCCCCCCOC(=O)/C=C/C(=O)OCC(C)Cl | 40 | 2 | 2 | 2 | 2 | 2 | 2 |
| Succinic acid, heptyl 2,3,5-trichlorophenyl ester (C17H21Cl3O4) | XOIGLVMSEUBMHM-UHFFFAOYSA-N | CCCCCCCOC(=O)CCC(=O)OC1=CC(=CC(=C1Cl)Cl)Cl | 0 | 2 | 2 | 2 | 2 | 2 | 2 |
| Fumaric acid, hexyl 2,3,5,6-tetrachlorophenyl ester (C16H16Cl4O4) | OFPNLEPZXPVEHX-VOTSOKGWSA-N | CCCCCCOC(=O)/C=C/C(=O)OC1=C(C(=CC(=C1Cl)Cl)Cl)Cl | 30 | 2 | 2 | 2 | 2 | 2 | 2 |
| Fumaric acid, isohexyl 2,3,5,6-tetrachlorophenyl ester (C16H16Cl4O4) | ISIZDRAVGGKUKL-AATRIKPKSA-N | CC(C)CCCOC(=O)/C=C/C(=O)OC1=C(C(=CC(=C1Cl)Cl)Cl)Cl | 50 | 2 | 2 | 2 | 2 | 2 | 2 |
| Furosemide, trimethyl (C15H17ClN2O5S) | OBJFIAVSHHYZPK-UHFFFAOYSA-N | CNS(=O)(=O)C1=C(C=C(C(=C1)C(=O)OC)N(C)CC2=CC=CO2)Cl | 1301 | 1 | 2 | 2 | 2 | 2 | 2 |
| Coumachlor (C19H15ClO4) | DEKWZWCFHUABHE-UHFFFAOYSA-N | CC(=O)CC(C1=CC=C(C=C1)Cl)C2=C(C3=CC=CC=C3OC2=O)O | 2739 | 1 | 2 | 2 | 1 | 2 | 2 |
| Adipic acid, 8-chloroctyl hexyl ester (C20H37ClO4) | QOKXDWWCPGIUMU-UHFFFAOYSA-N | CCCCCCOC(=O)CCCCC(=O)OCCCCCCCCCl | 0 | 2 | 2 | 2 | 2 | 2 | 2 |
| Succinic acid, 10-chlorodecyl isohexyl ester (C20H37ClO4) | BZKVKBXJIOJRKG-UHFFFAOYSA-N | CC(C)CCCOC(=O)CCC(=O)OCCCCCCCCCCCl | 0 | 2 | 2 | 2 | 2 | 2 | 2 |
| Hexanamide, N,N-bis(2-ethylhexyl)-6-chloro- (C22H44ClNO) | QIAXMLYRWAMPLX-UHFFFAOYSA-N | CCCCC(CC)CN(CC(CC)CCCC)C(=O)CCCCCCl | 0 | 2 | 2 | 2 | 2 | 2 | 2 |
| Succinic acid, dodecyl 2,2,2-trichloroethyl ester (C18H31Cl3O4) | BBLDVAZHTUMKSA-UHFFFAOYSA-N | CCCCCCCCCCCCOC(=O)CCC(=O)OCC(Cl)(Cl)Cl | 0 | 2 | 2 | 2 | 2 | 2 | 2 |
| Fumaric acid, dodecyl 2,2,2-trichloroethyl ester (C18H29Cl3O4) | IFPVIGYQBLXYMK-OUKQBFOZSA-N | CCCCCCCCCCCCOC(=O)/C=C/C(=O)OCC(Cl)(Cl)Cl | 0 | 2 | 2 | 2 | 2 | 2 | 2 |
| Sarcosine, N-(4-chlorobenzoyl)-, decyl ester (C20H30ClNO3) | UPRSSGWYPBBRLK-UHFFFAOYSA-N | CCCCCCCCCCOC(=O)CN(C)C(=O)C1=CC=C(C=C1)Cl | 1021 | 1 | 1 | 1 | 1 | 1 | 1 |
| β-Alanine, N-(2-chlorobenzoyl)-, decyl ester (C20H30ClNO3) | QYWPBIFYAZPZMP-UHFFFAOYSA-N | CCCCCCCCCCOC(=O)CCNC(=O)C1=CC=CC=C1Cl | 841 | 1 | 2 | 2 | 1 | 2 | 1 |
| β-Alanine, N-(4-chlorobenzoyl)-, decyl ester (C20H30ClNO3) | YCYJYQKVEYADGA-UHFFFAOYSA-N | CCCCCCCCCCOC(=O)CCNC(=O)C1=CC=C(C=C1)Cl | 631 | 2 | 2 | 2 | 2 | 2 | 2 |
| 2-Chlorobenzoic acid, 3-pentadecyl ester (C22H35ClO2) | LIPZCBXPWHSJGI-UHFFFAOYSA-N | CCCCCCCCCCCCC(CC)OC(=O)C1=CC=CC=C1Cl | 0 | 2 | 2 | 2 | 2 | 2 | 2 |
| Fumaric acid, heptyl 2,3,4,6-tetrachlorophenyl ester (C17H18Cl4O4) | GOGVZHKCOHWPGO-BQYQJAHWSA-N | CCCCCCCOC(=O)/C=C/C(=O)OC1=C(C(=C(C=C1Cl)Cl)Cl)Cl | 20 | 2 | 2 | 2 | 2 | 2 | 2 |
| Fumaric acid, octyl 2,4,6-trichlorophenyl ester (C18H21Cl3O4) | CEIGQFJBTNZSFB-CMDGGOBGSA-N | CCCCCCCCOC(=O)/C=C/C(=O)OC1=C(C=C(C=C1Cl)Cl)Cl | 20 | 2 | 2 | 2 | 2 | 2 | 2 |
| 2-Naphthalenecarboxamide, N-(5-chloro-2,4-dimethoxyphenyl)-3-hydroxy- (C19H16ClNO4) | XDWATWCCUTYUDE-UHFFFAOYSA-N | COC1=CC(=C(C=C1NC(=O)C2=CC3=CC=CC=C3C=C2O)Cl)OC | 727 | 2 | 1 | 1 | 1 | 1 | 1 |
| Amodiaquine (C20H22ClN3O) | OVCDSSHSILBFBN-UHFFFAOYSA-N | CCN(CC)CC1=C(C=CC(=C1)NC2=C3C=CC(=CC3=NC=C2)Cl)O | 3203 | 1 | 2 | 2 | 2 | 1 | 2 |
| Miconazole (C18H14Cl4N2O) | BYBLEWFAAKGYCD-UHFFFAOYSA-N | C1=CC(=C(C=C1Cl)Cl)COC(CN2C=CN=C2)C3=C(C=C(C=C3)Cl)Cl | 110 | 2 | 2 | 2 | 2 | 2 | 2 |
| Indomethacin (C19H16ClNO4) | CGIGDMFJXJATDK-UHFFFAOYSA-N | CC1=C(C2=C(N1C(=O)C3=CC=C(C=C3)Cl)C=CC(=C2)OC)CC(=O)O | 2600 | 1 | 1 | 1 | 1 | 1 | 1 |
| Cyclohexanone, 2-(3-chloro-2-butenyl)-2-methyl-6,6-diphenyl- (C23H25ClO) | TWBAHMRTJVSXOP-NBVRZTHBSA-N | C/C(=C\CC1(CCCC(C1=O)(C2=CC=CC=C2)C3=CC=CC=C3)C)/Cl | 3410 | 1 | 2 | 2 | 1 | 1 | 1 |
| Succinic acid, di(8-chloroctyl) ester (C20H36Cl2O4) | WSNMNGSFXRGKBL-UHFFFAOYSA-N | C(CCCCCl)CCCOC(=O)CCC(=O)OCCCCCCCCCl | 0 | 2 | 2 | 2 | 2 | 2 | 2 |
| Succinic acid, 2,2,2-trichloroethyl tridecyl ester (C19H33Cl3O4) | GSNGKIWQLUMYKR-UHFFFAOYSA-N | CCCCCCCCCCCCCOC(=O)CCC(=O)OCC(Cl)(Cl)Cl | 0 | 2 | 2 | 2 | 2 | 2 | 2 |
| β-Alanine, N-(2-chlorobenzoyl)-, undecyl ester (C21H32ClNO3) | OGLQUWIGLHDZMG-UHFFFAOYSA-N | CCCCCCCCCCCOC(=O)CCNC(=O)C1=CC=CC=C1Cl | 821 | 1 | 2 | 2 | 1 | 2 | 1 |
| Benzamide, N,N-bis(2-ethylhexyl)-4-chloro- (C23H38ClNO) | MMHNMAOTACRUMR-UHFFFAOYSA-N | CCCCC(CC)CN(CC(CC)CCCC)C(=O)C1=CC=C(C=C1)Cl | 50 | 2 | 2 | 2 | 1 | 2 | 2 |
| Succinic acid, di(2,3,5-trichlorophenyl) ester (C16H8Cl6O4) | POGRXWJDWDDOOU-UHFFFAOYSA-N | C1=C(C=C(C(=C1Cl)Cl)OC(=O)CCC(=O)OC2=CC(=CC(=C2Cl)Cl)Cl)Cl | 0 | 2 | 2 | 2 | 2 | 2 | 2 |
| Benzthiazide (C15H14ClN3O4S3) | NDTSRXAMMQDVSW-UHFFFAOYSA-N | C1=CC=C(C=C1)CSCC2=NS(=O)(=O)C3=CC(=C(C=C3N2)Cl)S(=O)(=O)N | 190 | 2 | 2 | 1 | 1 | 1 | 1 |
| Quizalofop-P-ethyl (C19H17ClN2O4) | OSUHJPCHFDQAIT-UHFFFAOYSA-N | CCOC(=O)C(C)OC1=CC=C(C=C1)OC2=CN=C3C=C(C=CC3=N2)Cl | 8858 | 1 | 1 | 1 | 1 | 1 | 1 |
| Hydroxyzine (C21H27ClN2O2) | ZQDWXGKKHFNSQK-UHFFFAOYSA-N | C1CN(CCN1CCOCCO)C(C2=CC=CC=C2)C3=CC=C(C=C3)Cl | 640 | 2 | 2 | 2 | 1 | 2 | 2 |
| cis-Permethrin (C21H20Cl2O3) | RLLPVAHGXHCWKJ-HKUYNNGSSA-N | CC1([C@H]([C@H]1C(=O)OCC2=CC(=CC=C2)OC3=CC=CC=C3)C=C(Cl)Cl)C | 60 | 2 | 2 | 2 | 2 | 2 | 2 |
| Fumaric acid, 2,2-dichloroethyl pentadecyl ester (C21H36Cl2O4) | MWXYEQDJMAXBGQ-FOCLMDBBSA-N | CCCCCCCCCCCCCCCOC(=O)/C=C/C(=O)OCC(Cl)Cl | 30 | 2 | 2 | 2 | 2 | 2 | 2 |
| Sarcosine, N-(2-chlorobenzoyl)-, dodecyl ester (C22H34ClNO3) | QNPJVNNCUBIYBF-UHFFFAOYSA-N | CCCCCCCCCCCCOC(=O)CN(C)C(=O)C1=CC=CC=C1Cl | 941 | 1 | 1 | 1 | 1 | 1 | 1 |
| Fumaric acid, decyl 2,3,6-trichlorophenyl ester (C20H25Cl3O4) | UKPVUYCTLVSBPN-OUKQBFOZSA-N | CCCCCCCCCCOC(=O)/C=C/C(=O)OC1=C(C=CC(=C1Cl)Cl)Cl | 70 | 2 | 2 | 2 | 2 | 2 | 2 |
| Bicyclo[2.2.1]hept-5-ene-2,3-dicarboxylic acid, 1,4,5,6,7,7-hexachloro-, dibutyl ester (C17H20Cl6O4) | UJAHPBDUQZFDLA-UHFFFAOYSA-N | CCCCOC(=O)C1C(C2(C(=C(C1(C2(Cl)Cl)Cl)Cl)Cl)Cl)C(=O)OCCCC | 0 | 2 | 2 | 2 | 2 | 2 | 2 |
| Endrin (C12H8Cl6O) | DFBKLUNHFCTMDC-GKRDHZSOSA-N | C1[C@@H]2[C@@H]3[C@H]([C@H]1[C@H]4[C@@H]2O4)[C@@]5(C(=C([C@]3(C5(Cl)Cl)Cl)Cl)Cl)Cl | 70 | 2 | 2 | 2 | 2 | 2 | 1 |
| Succinic acid, 2,2-dichloroethyl hexadecyl ester (C22H40Cl2O4) | UOCYXFFWUUTHLW-UHFFFAOYSA-N | CCCCCCCCCCCCCCCCOC(=O)CCC(=O)OCC(Cl)Cl | 0 | 2 | 2 | 2 | 2 | 2 | 2 |
| Fumaric acid, 10-chlorodecyl nonyl ester (C23H41ClO4) | ACJORMVAPZCSEZ-ISLYRVAYSA-N | CCCCCCCCCOC(=O)/C=C/C(=O)OCCCCCCCCCCCl | 0 | 2 | 2 | 2 | 2 | 2 | 2 |
| Succinic acid, 3,5-dichlorophenyl dodecyl ester (C22H32Cl2O4) | VVFZSHOFCJGLEL-UHFFFAOYSA-N | CCCCCCCCCCCCOC(=O)CCC(=O)OC1=CC(=CC(=C1)Cl)Cl | 0 | 2 | 2 | 2 | 2 | 2 | 2 |
| Fumaric acid, 2-chloro-5-methylphenyl dodecyl ester (C23H33ClO4) | PKAHTTPOKHAOIZ-FOCLMDBBSA-N | CCCCCCCCCCCCOC(=O)/C=C/C(=O)OC1=C(C=CC(=C1)C)Cl | 140 | 2 | 2 | 2 | 2 | 2 | 2 |
| Succinic acid, hexadecyl 2,2,2-trichloroethyl ester (C22H39Cl3O4) | GGIKVMZYWDHFFS-UHFFFAOYSA-N | CCCCCCCCCCCCCCCCOC(=O)CCC(=O)OCC(Cl)(Cl)Cl | 0 | 2 | 2 | 2 | 2 | 2 | 2 |
| Succinic acid, dodecyl 2,3,6-trichlorophenyl ester (C22H31Cl3O4) | ZISPEBQCKRKILO-UHFFFAOYSA-N | CCCCCCCCCCCCOC(=O)CCC(=O)OC1=C(C=CC(=C1Cl)Cl)Cl | 0 | 2 | 2 | 2 | 2 | 2 | 2 |
| β-Alanine, N-(2-chlorobenzoyl)-, tetradecyl ester (C24H38ClNO3) | WGEPOKJCBMIFIN-UHFFFAOYSA-N | CCCCCCCCCCCCCCOC(=O)CCNC(=O)C1=CC=CC=C1Cl | 721 | 2 | 2 | 2 | 1 | 2 | 1 |
| Sarcosine, N-(4-chlorobenzoyl)-, tetradecyl ester (C24H38ClNO3) | GVTFKZULRJMDTB-UHFFFAOYSA-N | CCCCCCCCCCCCCCOC(=O)CN(C)C(=O)C1=CC=C(C=C1)Cl | 1171 | 1 | 1 | 1 | 1 | 1 | 1 |
| Fumaric acid, 8-chlorooctyl tridecyl ester (C25H45ClO4) | KXJLNMQRVDAJCN-FMQUCBEESA-N | CCCCCCCCCCCCCOC(=O)/C=C/C(=O)OCCCCCCCCCl | 20 | 2 | 2 | 2 | 2 | 2 | 2 |
| Fumaric acid, 2,4,6-trichlorophenyl tridecyl ester (C23H31Cl3O4) | RFHVSCGCTYDDEL-BUHFOSPRSA-N | CCCCCCCCCCCCCOC(=O)/C=C/C(=O)OC1=C(C=C(C=C1Cl)Cl)Cl | 0 | 2 | 2 | 2 | 2 | 2 | 2 |
| Succinic acid, 2,2-dichloroethyl nonadecyl ester (C25H46Cl2O4) | ZTESYAMDHFJNHK-UHFFFAOYSA-N | CCCCCCCCCCCCCCCCCCCOC(=O)CCC(=O)OCC(Cl)Cl | 0 | 2 | 2 | 2 | 2 | 2 | 2 |
| Succinic acid, octadecyl 2,2,2-trichloroethyl ester (C24H43Cl3O4) | HIGJJEDMWGWLJX-UHFFFAOYSA-N | CCCCCCCCCCCCCCCCCCOC(=O)CCC(=O)OCC(Cl)(Cl)Cl | 0 | 2 | 2 | 2 | 2 | 2 | 2 |
| Succinic acid, 2,3,4,6-tetrachlorophenyl tridecyl ester (C23H32Cl4O4) | FFQOTIQTIMSPCZ-UHFFFAOYSA-N | CCCCCCCCCCCCCOC(=O)CCC(=O)OC1=C(C(=C(C=C1Cl)Cl)Cl)Cl | 0 | 2 | 2 | 2 | 2 | 2 | 2 |
| Adipic acid, 8-chloroctyl tridecyl ester (C27H51ClO4) | KWSLVIWMUBDKCJ-UHFFFAOYSA-N | CCCCCCCCCCCCCOC(=O)CCCCC(=O)OCCCCCCCCCl | 0 | 2 | 2 | 2 | 2 | 2 | 2 |
| Succinic acid, pentadecyl 2,3,6-trichlorophenyl ester (C25H37Cl3O4) | BYEZQRLTMPJVRB-UHFFFAOYSA-N | CCCCCCCCCCCCCCCOC(=O)CCC(=O)OC1=C(C=CC(=C1Cl)Cl)Cl | 0 | 2 | 2 | 2 | 2 | 2 | 2 |
| Adipic acid, 8-chloroctyl tetradecyl ester (C28H53ClO4) | NECYQUIQLJYGQL-UHFFFAOYSA-N | CCCCCCCCCCCCCCOC(=O)CCCCC(=O)OCCCCCCCCCl | 0 | 2 | 2 | 2 | 2 | 2 | 2 |

## 1.2. Criterion II

**Table S5** The results of [M-35] peaks classification (criterion I) performed for the test set using MLP 100-25-2 (model 1), MLP 100-22-2 (model 2), MLP 100-22-2 (BFGS 73) (model 3), MLP 100-22-2 (BFGS 72) (model 4) and MLP 100-24-2 (BFGS 48) (model 5). Mass spectra data were obtained from NIST Chemistry WebBook database (http://webbook.nist.gov/chemistry/)

| **Name** | **IUPAC Standard InChIKey** | **SMILES (PUBCHEM ISOMERIC)** | **[M-Cl]** | **[M-35] class exp.** | **Model** | | | | |
| --- | --- | --- | --- | --- | --- | --- | --- | --- | --- |
|  |  |  |  |  | **1** | **2** | **3** | **4** | **5** |
| Ethyl Chloride (C2H5Cl) | HRYZWHHZPQKTII-UHFFFAOYSA-N | CCCl | 7216 | 1 | 1 | 1 | 1 | 1 | 1 |
| Thiophosgene (CCl2S) | ZWZVWGITAAIFPS-UHFFFAOYSA-N | C(=S)(Cl)Cl | 9999 | 1 | 1 | 1 | 1 | 1 | 1 |
| Acetaldehyde, chloro- (C2H3ClO) | QSKPIOLLBIHNAC-UHFFFAOYSA-N | C(C=O)Cl | 110 | 1 | 1 | 1 | 1 | 1 | 1 |
| 1-Chloropropane (C3H7Cl) | SNMVRZFUUCLYTO-UHFFFAOYSA-N | CCCCl | 1369 | 1 | 1 | 1 | 1 | 1 | 1 |
| 1-Chloropropene (C3H5Cl) | OWXJKYNZGFSVRC-UHFFFAOYSA-N | CC=CCl | 9999 | 1 | 1 | 1 | 1 | 1 | 1 |
| Methane, chloro- (CH3Cl) | NEHMKBQYUWJMIP-UHFFFAOYSA-N | CCl | 7237 | 1 | 1 | 1 | 1 | 1 | 1 |
| Carbonochloridothioic acid, S-methyl ester (C2H3ClOS) | YPSUCTSXOROPBS-UHFFFAOYSA-N | CSC(=O)Cl | 9999 | 1 | 1 | 1 | 1 | 1 | 1 |
| 1-Chlorobutane (C4H9Cl) | VFWCMGCRMGJXDK-UHFFFAOYSA-N | CCCCCl | 570 | 1 | 1 | 1 | 1 | 1 | 1 |
| 2-Propenenitrile, 2-chloro- (C3H2ClN) | OYUNTGBISCIYPW-UHFFFAOYSA-N | C=C(C#N)Cl | 9999 | 1 | 1 | 1 | 1 | 1 | 1 |
| 2-Propenoyl chloride (C3H3ClO) | HFBMWMNUJJDEQZ-UHFFFAOYSA-N | C=CC(=O)Cl | 9999 | 1 | 1 | 1 | 1 | 1 | 1 |
| 1-Butene, 1-chloro-, (Z)- (C4H7Cl) | DUDKKPVINWLFBI-ARJAWSKDSA-N | CC/C=C\Cl | 9999 | 1 | 1 | 1 | 1 | 1 | 1 |
| Chloroprene (C4H5Cl) | YACLQRRMGMJLJV-UHFFFAOYSA-N | C=CC(=C)Cl | 9999 | 1 | 1 | 1 | 1 | 1 | 1 |
| Oxirane, (chloromethyl)-, (R)- (C3H5ClO) | BRLQWZUYTZBJKN-GSVOUGTGSA-N | C1[C@H](O1)CCl | 9999 | 1 | 1 | 1 | 1 | 1 | 1 |
| Chloromethyl sulfonylchloride (CH2Cl2O2S) | KQDDQXNVESLJNO-UHFFFAOYSA-N | C(S(=O)(=O)Cl)Cl | 475 | 1 | 1 | 1 | 1 | 2 | 1 |
| 2-Chloro-N-methylacetamide (C3H6ClNO) | HOZLOOPIXHWKCI-UHFFFAOYSA-N | CNC(=O)CCl | 1445 | 1 | 1 | 1 | 1 | 1 | 1 |
| Oxalyl chloride (C2Cl2O2) | CTSLXHKWHWQRSH-UHFFFAOYSA-N | C(=O)(C(=O)Cl)Cl | 70 | 2 | 1 | 1 | 1 | 1 | 1 |
| Dichloroacetyl chloride (C2HCl3O) | FBCCMZVIWNDFMO-UHFFFAOYSA-N | C(C(=O)Cl)(Cl)Cl | 1009 | 1 | 1 | 1 | 1 | 1 | 1 |
| 2,3-Dichloro-1-propanol (C3H6Cl2O) | ZXCYIJGIGSDJQQ-UHFFFAOYSA-N | C(C(CCl)Cl)O | 50 | 2 | 1 | 1 | 1 | 1 | 1 |
| 2-Butyne, 1,4-dichloro- (C4H4Cl2) | RCHDLEVSZBOHOS-UHFFFAOYSA-N | C(C#CCCl)Cl | 9999 | 1 | 1 | 1 | 1 | 1 | 1 |
| 2,3-Dichlorobutane (C4H8Cl2) | RMISVOPUIFJTEO-UHFFFAOYSA-N | CC(C(C)Cl)Cl | 929 | 1 | 1 | 1 | 1 | 1 | 1 |
| 2-Chloroethyl vinyl ether (C4H7ClO) | DNJRKFKAFWSXSE-UHFFFAOYSA-N | C=COCCCl | 70 | 2 | 1 | 1 | 1 | 1 | 1 |
| 1-Pentene, 5-chloro- (C5H9Cl) | UPOBJNRMUDPATE-UHFFFAOYSA-N | C=CCCCCl | 1749 | 1 | 1 | 1 | 1 | 1 | 1 |
| 1-Chloro-3-methyl-2-butene (C5H9Cl) | JKXQKGNGJVZKFA-UHFFFAOYSA-N | CC(=CCCl)C | 9999 | 1 | 1 | 1 | 1 | 1 | 1 |
| 2-Chloro-3-methyl-1-butene (C5H9Cl) | RBSYGFLXVMWYGD-UHFFFAOYSA-N | CC(C)C(=C)Cl | 9999 | 1 | 1 | 1 | 1 | 1 | 1 |
| 2-Chloro-3-methyl-2-butene (C5H9Cl) | WIIKEBDPJPYJHF-UHFFFAOYSA-N | CC(=C(C)Cl)C | 9560 | 1 | 1 | 1 | 1 | 1 | 1 |
| 1H-1,2,4-Triazole, 3-chloro- (C2H2ClN3) | QGOUKZPSCTVYLX-UHFFFAOYSA-N | C1=NNC(=N1)Cl | 78 | 2 | 1 | 1 | 1 | 1 | 1 |
| Cyclopentene, 1-chloro- (C5H7Cl) | UJUIJZWQFDQKHO-UHFFFAOYSA-N | C1CC=C(C1)Cl | 9999 | 1 | 1 | 1 | 1 | 1 | 1 |
| β-Methoxyethoxymethyl chloride (C4H9ClO2) | BIAAQBNMRITRDV-UHFFFAOYSA-N | COCCOCCl | 1131 | 1 | 2 | 2 | 2 | 2 | 2 |
| Chloral Hydrate (C2H3Cl3O2) | RNFNDJAIBTYOQL-UHFFFAOYSA-N | C(C(Cl)(Cl)Cl)(O)O | 0 | 2 | 1 | 1 | 1 | 1 | 1 |
| Acetamide, 2,2,2-trichloro- (C2H2Cl3NO) | UPQQXPKAYZYUKO-UHFFFAOYSA-N | C(=O)(C(Cl)(Cl)Cl)N | 179 | 1 | 1 | 1 | 1 | 1 | 1 |
| Ethyl chloroacetate (C4H7ClO2) | VEUUMBGHMNQHGO-UHFFFAOYSA-N | CCOC(=O)CCl | 10 | 2 | 2 | 2 | 2 | 2 | 2 |
| Propane, 2-chloro-2-nitro- (C3H6ClNO2) | JQYFSFNSNVRUPY-UHFFFAOYSA-N | CC(C)([N+](=O)[O-])Cl | 0 | 2 | 2 | 2 | 2 | 2 | 2 |
| Bis(2-chloroethyl) sulphide (C4H8Cl2S) | QKSKPIVNLNLAAV-UHFFFAOYSA-N | C(CCl)SCCCl | 310 | 1 | 1 | 1 | 1 | 1 | 1 |
| Bis(2-chloroethyl) ether (C4H8Cl2O) | ZNSMNVMLTJELDZ-UHFFFAOYSA-N | C(CCl)OCCCl | 0 | 2 | 2 | 2 | 2 | 2 | 2 |
| 2,3-dichloropropionyl chloride (C3H3Cl3O) | JQELECXPPAOSTM-UHFFFAOYSA-N | C(C(C(=O)Cl)Cl)Cl | 5195 | 1 | 1 | 1 | 1 | 1 | 1 |
| Propane, 1,1,2,2-tetrachloro- (C3H4Cl4) | MDCBRXYTSHYYJE-UHFFFAOYSA-N | CC(C(Cl)Cl)(Cl)Cl | 2803 | 1 | 1 | 1 | 1 | 1 | 1 |
| 5-Chlorovaleronitrile (C5H8ClN) | JSAWFGSXRPCFSW-UHFFFAOYSA-N | C(CCCl)CC#N | 1275 | 1 | 1 | 1 | 1 | 1 | 1 |
| Butanoyl chloride, 3-methyl- (C5H9ClO) | ISULZYQDGYXDFW-UHFFFAOYSA-N | CC(C)CC(=O)Cl | 5539 | 1 | 1 | 1 | 1 | 1 | 1 |
| Ethylmethylacetylchloride (C5H9ClO) | XRPVXVRWIDOORM-UHFFFAOYSA-N | CCC(C)C(=O)Cl | 1629 | 1 | 1 | 1 | 1 | 1 | 1 |
| Pivalyl chloride (C5H9ClO) | JVSFQJZRHXAUGT-UHFFFAOYSA-N | CC(C)(C)C(=O)Cl | 189 | 1 | 2 | 2 | 2 | 1 | 2 |
| 1,4-Dichloro-2-methylbutane (C5H10Cl2) | OUSZUUNUORQHDW-UHFFFAOYSA-N | CC(CCCl)CCl | 194 | 1 | 1 | 1 | 1 | 1 | 1 |
| Pentane, 3-chloro-3-methyl- (C6H13Cl) | SGWJUIFOPCZXMR-UHFFFAOYSA-N | CCC(C)(CC)Cl | 5148 | 1 | 1 | 1 | 1 | 1 | 1 |
| 1-Propene, 1,2,3,3-tetrachloro- (C3H2Cl4) | JUGQRTGGLWOBPG-UPHRSURJSA-N | C(=C(/C(Cl)Cl)\Cl)\Cl | 9999 | 1 | 1 | 1 | 1 | 1 | 1 |
| 4,5-Dichloroimidazole (C3H2Cl2N2) | CHUPRLGXGZETTE-UHFFFAOYSA-N | C1=NNC(=C1Cl)Cl | 791 | 1 | 1 | 1 | 1 | 1 | 1 |
| 1-chlorocyclohex-1-ene (C6H9Cl) | BUAKPITZELZWNI-UHFFFAOYSA-N | C1CCC(=CC1)Cl | 9999 | 1 | 1 | 1 | 1 | 1 | 1 |
| 1,1,3,3-Tetrachloroacetone (C3H2Cl4O) | DJWVKJAGMVZYFP-UHFFFAOYSA-N | C(C(=O)C(Cl)Cl)(Cl)Cl | 90 | 2 | 2 | 1 | 2 | 2 | 2 |
| 2-Propanol, 1-chloro-3-ethoxy- (C5H11ClO2) | XHIINWKFCZSGNY-UHFFFAOYSA-N | CCOCC(CCl)O | 42 | 2 | 2 | 2 | 2 | 2 | 2 |
| 3-Chloro-2,4-pentanedione (C5H7ClO2) | VLRGXXKFHVJQOL-UHFFFAOYSA-N | CC(=O)C(C(=O)C)Cl | 399 | 1 | 2 | 1 | 1 | 2 | 2 |
| Hexane, 3-chloro-3-methyl- (C7H15Cl) | UTKDCNDVTOWUHW-UHFFFAOYSA-N | CCCC(C)(CC)Cl | 871 | 1 | 1 | 1 | 1 | 1 | 1 |
| 2-Chloro-2,4-dimethylpentane (C7H15Cl) | DQOHPSPKVODKLV-UHFFFAOYSA-N | CC(C)CC(C)(C)Cl | 5451 | 1 | 1 | 1 | 1 | 1 | 1 |
| 2-Propenoic acid, 2-chloroethyl ester (C5H7ClO2) | WHBAYNMEIXUTJV-UHFFFAOYSA-N | C=CC(=O)OCCCl | 1460 | 1 | 1 | 1 | 1 | 1 | 1 |
| 2,6-Dichloropyrazine (C4H2Cl2N2) | LSEAAPGIZCDEEH-UHFFFAOYSA-N | C1=C(N=C(C=N1)Cl)Cl | 6919 | 1 | 1 | 1 | 1 | 1 | 1 |
| 3-Amino-2-chloropyridine (C5H5ClN2) | MEQBJJUWDCYIAB-UHFFFAOYSA-N | C1=CC(=C(N=C1)Cl)N | 1859 | 1 | 1 | 1 | 1 | 1 | 1 |
| 1,3-Dichlorobenzene (C6H4Cl2) | ZPQOPVIELGIULI-UHFFFAOYSA-N | C1=CC(=CC(=C1)Cl)Cl | 4510 | 1 | 1 | 1 | 1 | 1 | 1 |
| Benzyl chloride (C7H7Cl) | KCXMKQUNVWSEMD-UHFFFAOYSA-N | C1=CC=C(C=C1)CCl | 9999 | 1 | 1 | 1 | 1 | 1 | 1 |
| 2,2-Dichlorocyclopropylacetonitrile (C5H5Cl2N) | METYSMJJRFRDPP-UHFFFAOYSA-N | C1C(C1(Cl)Cl)CC#N | 573 | 1 | 1 | 1 | 1 | 1 | 1 |
| Bis(β-chloroethyl) sulfone (C4H8Cl2O2S) | LUYAMNYBNTVQJG-UHFFFAOYSA-N | C(CCl)S(=O)(=O)CCCl | 0 | 2 | 2 | 2 | 2 | 2 | 2 |
| Butanoic acid, 2-chloro-3-oxo-, methyl ester (C5H7ClO3) | GYQRIAVRKLRQKP-UHFFFAOYSA-N | CC(=O)C(C(=O)OC)Cl | 0 | 2 | 2 | 2 | 2 | 2 | 2 |
| 3-chloropropyl chloroacetate (C5H8Cl2O2) | CWZVJVDIQWKNJX-UHFFFAOYSA-N | C(COC(=O)CCl)CCl | 1199 | 1 | 2 | 2 | 2 | 2 | 2 |
| 2,3-Dichloropropyl acetate (C5H8Cl2O2) | BVXPMFQVOWRQKD-UHFFFAOYSA-N | CC(=O)OCC(CCl)Cl | 30 | 2 | 2 | 1 | 1 | 2 | 1 |
| 2,4-Dichlorobutanoic acid, methyl ester (C5H8Cl2O2) | MIXXUSQBRXDJHD-UHFFFAOYSA-N | COC(=O)C(CCCl)Cl | 600 | 1 | 1 | 1 | 1 | 1 | 1 |
| 4,4-Dichlorobutanoic acid, methyl ester (C5H8Cl2O2) | FLTUWIKFBYMPIV-UHFFFAOYSA-N | COC(=O)CCC(Cl)Cl | 600 | 1 | 1 | 1 | 1 | 1 | 1 |
| Methyl threo-2,3-dichlorobutanoate (C5H8Cl2O2) | KJZMAXQPXRBYDC-UHFFFAOYSA-N | CC(C(C(=O)OC)Cl)Cl | 330 | 1 | 1 | 1 | 1 | 1 | 1 |
| 2-Propanol, 1-chloro-3-isopropoxy- (C6H13ClO2) | GQPJSBQMFFGZAU-UHFFFAOYSA-N | CC(C)OCC(CCl)O | 20 | 2 | 2 | 2 | 2 | 2 | 2 |
| Chloromethyl pivalate (C6H11ClO2) | GGRHYQCXXYLUTL-UHFFFAOYSA-N | CC(C)(C)C(=O)OCCl | 0 | 2 | 2 | 2 | 2 | 2 | 2 |
| Propane, 1,1'-thiobis[3-chloro- (C6H12Cl2S) | VDTHWBLOSZIMMN-UHFFFAOYSA-N | C(CSCCCCl)CCl | 115 | 1 | 2 | 1 | 2 | 2 | 1 |
| Propane, 1,1'-oxybis[3-chloro- (C6H12Cl2O) | SMANNJALMIGASX-UHFFFAOYSA-N | C(COCCCCl)CCl | 10 | 2 | 2 | 2 | 2 | 2 | 2 |
| Bis(2-chloropropyl) sulfide (C6H12Cl2S) | AQHTWLYIOSPNMG-UHFFFAOYSA-N | CC(CSCC(C)Cl)Cl | 1381 | 1 | 1 | 1 | 1 | 1 | 1 |
| bis(2-chloro-1-methylethyl) ether (C6H12Cl2O) | QCFYJCYNJLBDRT-UHFFFAOYSA-N | CC(CCl)OC(C)CCl | 10 | 2 | 2 | 2 | 2 | 2 | 2 |
| 2-Butanone, 1,1-dichloro-3,3-dimethyl- (C6H10Cl2O) | UDWZXMQIEHAAQT-UHFFFAOYSA-N | CC(C)(C)C(=O)C(Cl)Cl | 0 | 2 | 2 | 2 | 2 | 2 | 2 |
| 2-Chlorooctane (C8H17Cl) | HKDCIIMOALDWHF-UHFFFAOYSA-N | CCCCCCC(C)Cl | 117 | 1 | 2 | 2 | 2 | 2 | 2 |
| Desethyl-desisopropyl-atrazine (C3H4ClN5) | FVFVNNKYKYZTJU-UHFFFAOYSA-N | C1(=NC(=NC(=N1)Cl)N)N | 3069 | 1 | 1 | 1 | 1 | 1 | 1 |
| Pyridazine, 3-chloro-6-methoxy- (C5H5ClN2O) | XBJLKXOOHLLTPG-UHFFFAOYSA-N | COC1=NN=C(C=C1)Cl | 1756 | 1 | 1 | 1 | 1 | 1 | 1 |
| 3,4,5-Trichloropyridine (C5H2Cl3N) | KKWRVUBDCJQHBZ-UHFFFAOYSA-N | C1=C(C(=C(C=N1)Cl)Cl)Cl | 4994 | 1 | 1 | 1 | 1 | 1 | 1 |
| Benzenamine, 2-chloro-N-methyl- (C7H8ClN) | WGNNILPYHCKCFF-UHFFFAOYSA-N | CNC1=CC=CC=C1Cl | 170 | 1 | 1 | 1 | 1 | 1 | 1 |
| 3-Chloroanisole (C7H7ClO) | YUKILTJWFRTXGB-UHFFFAOYSA-N | COC1=CC(=CC=C1)Cl | 909 | 1 | 1 | 1 | 1 | 1 | 1 |
| 4-Chloroanisole (C7H7ClO) | YRGAYAGBVIXNAQ-UHFFFAOYSA-N | COC1=CC=C(C=C1)Cl | 580 | 1 | 1 | 1 | 1 | 1 | 1 |
| 2,6-Dichloroaniline (C6H5Cl2N) | JDMFXJULNGEPOI-UHFFFAOYSA-N | C1=CC(=C(C(=C1)Cl)N)Cl | 1259 | 1 | 1 | 1 | 1 | 1 | 1 |
| 4-Chlorobenzyl mercaptan (C7H7ClS) | GKQXPTHQTXCXEV-UHFFFAOYSA-N | C1=CC(=CC=C1CS)Cl | 80 | 2 | 1 | 1 | 1 | 1 | 1 |
| m-Chlorobenzaldehyde (C7H5ClO) | SRWILAKSARHZPR-UHFFFAOYSA-N | C1=CC(=CC(=C1)Cl)C=O | 30 | 2 | 2 | 2 | 2 | 2 | 2 |
| Phenol, 5-chloro-2-methyl- (C7H7ClO) | KKFPXGXMSBBNJI-UHFFFAOYSA-N | CC1=C(C=C(C=C1)Cl)O | 9999 | 1 | 1 | 1 | 1 | 1 | 1 |
| Phenol, 3-chloro-4-methyl- (C7H7ClO) | VQZRLBWPEHFGCD-UHFFFAOYSA-N | CC1=C(C=C(C=C1)O)Cl | 9999 | 1 | 1 | 1 | 1 | 1 | 1 |
| Benzene, 1-chloro-3-(chloromethyl)- (C7H6Cl2) | DDGRAFHHXYIQQR-UHFFFAOYSA-N | C1=CC(=CC(=C1)Cl)CCl | 9999 | 1 | 1 | 1 | 1 | 1 | 1 |
| 2-Chloro-3,6-dimethylpyrazine (C6H7ClN2) | NNBALVIZMGWZHS-UHFFFAOYSA-N | CC1=CN=C(C(=N1)Cl)C | 9568 | 1 | 1 | 1 | 1 | 1 | 1 |
| Benzene, 1-(chloromethyl)-2-methyl- (C8H9Cl) | VQRBXYBBGHOGFT-UHFFFAOYSA-N | CC1=CC=CC=C1CCl | 9999 | 1 | 1 | 1 | 1 | 1 | 1 |
| Benzene, 1-chloro-3-ethyl- (C8H9Cl) | LOXUEGMPESDGBQ-UHFFFAOYSA-N | CCC1=CC(=CC=C1)Cl | 8818 | 1 | 1 | 1 | 1 | 1 | 1 |
| Benzene, 1-chloro-4-ethyl- (C8H9Cl) | GPOFSFLJOIAMSA-UHFFFAOYSA-N | CCC1=CC=C(C=C1)Cl | 4694 | 1 | 1 | 1 | 1 | 1 | 1 |
| m-Xylene, 2-chloro- (C8H9Cl) | VDXLAYAQGYCQEO-UHFFFAOYSA-N | CC1=C(C(=CC=C1)C)Cl | 9999 | 1 | 1 | 1 | 1 | 1 | 1 |
| Cyclohexanone,2-chloro-2-methyl- (C7H11ClO) | IDRWHLQDVSLCBJ-UHFFFAOYSA-N | CC1(CCCCC1=O)Cl | 400 | 1 | 1 | 1 | 1 | 1 | 1 |
| 4-chlorostyrene (C8H7Cl) | KTZVZZJJVJQZHV-UHFFFAOYSA-N | C=CC1=CC=C(C=C1)Cl | 4964 | 1 | 1 | 1 | 1 | 1 | 1 |
| Benzene, 1-chloro-3-ethenyl- (C8H7Cl) | BOVQCIDBZXNFEJ-UHFFFAOYSA-N | C=CC1=CC(=CC=C1)Cl | 7529 | 1 | 1 | 1 | 1 | 1 | 1 |
| 5-Chloro-2-thiophenecarboxylic acid (C5H3ClO2S) | QZLSBOVWPHXCLT-UHFFFAOYSA-N | C1=C(SC(=C1)Cl)C(=O)O | 46 | 2 | 1 | 2 | 2 | 2 | 2 |
| Propanoic acid, 2,2,3,3-tetrachloro, methyl ester (C4H4Cl4O2) | UQYYMXYJSOOLLA-UHFFFAOYSA-N | COC(=O)C(C(Cl)Cl)(Cl)Cl | 230 | 1 | 1 | 1 | 1 | 1 | 1 |
| 1,1,1,2,2,3,3-Heptachloropropane (C3HCl7) | YFIIENAGGCUHIQ-UHFFFAOYSA-N | C(C(C(Cl)(Cl)Cl)(Cl)Cl)(Cl)Cl | 189 | 1 | 1 | 1 | 1 | 1 | 1 |
| Ethanol, 2-[2-(2-chloroethoxy)ethoxy]- (C6H13ClO3) | KECMLGZOQMJIBM-UHFFFAOYSA-N | C(COCCOCCCl)O | 0 | 2 | 2 | 2 | 2 | 2 | 2 |
| Acetic acid, dichloro, isobutyl ester (C6H10Cl2O2) | XLKJPQKUATYFQJ-UHFFFAOYSA-N | CC(C)COC(=O)C(Cl)Cl | 0 | 2 | 2 | 2 | 2 | 2 | 2 |
| Acetic acid, chloro-, 3-methylbutyl ester (C7H13ClO2) | UZQBACINTKFBSX-UHFFFAOYSA-N | CC(C)CCOC(=O)CCl | 0 | 2 | 2 | 2 | 2 | 2 | 2 |
| Propanoic acid, 3-chloro, 1-methylpropyl ester (C7H13ClO2) | XLIOZHNKYDPTAJ-UHFFFAOYSA-N | CCC(C)OC(=O)CCCl | 0 | 2 | 2 | 2 | 2 | 2 | 2 |
| Octanoyl chloride (C8H15ClO) | REEZZSHJLXOIHL-UHFFFAOYSA-N | CCCCCCCC(=O)Cl | 3140 | 1 | 1 | 1 | 1 | 1 | 1 |
| 2-ethylhexanoyl chloride (C8H15ClO) | WFSGQBNCVASPMW-UHFFFAOYSA-N | CCCCC(CC)C(=O)Cl | 309 | 1 | 1 | 1 | 1 | 1 | 1 |
| Trichloroacetic acid 2-propenyl ester (C5H5Cl3O2) | LJQCONXCOYBYIE-UHFFFAOYSA-N | C=CCOC(=O)C(Cl)(Cl)Cl | 90 | 2 | 1 | 1 | 1 | 1 | 1 |
| 4-Pyrimidinamine, 6-chloro-2-(methylthio)- (C5H6ClN3S) | ISUXMAHVLFRZQU-UHFFFAOYSA-N | CSC1=NC(=CC(=N1)Cl)N | 289 | 1 | 1 | 1 | 1 | 1 | 1 |
| Pyrimidine, 4,6-dichloro-2-(methylthio)- (C5H4Cl2N2S) | FCMLONIWOAGZJX-UHFFFAOYSA-N | CSC1=NC(=CC(=N1)Cl)Cl | 3049 | 1 | 1 | 1 | 1 | 1 | 1 |
| 2-Hydroxy-3,5,6-trichloropyridine (C5H2Cl3NO) | WCYYAQFQZQEUEN-UHFFFAOYSA-N | C1=C(C(=O)NC(=C1Cl)Cl)Cl | 1505 | 1 | 1 | 1 | 1 | 1 | 1 |
| 2-Chlorophenyl isothiocyanate (C7H4ClNS) | DASSPOJBUMBXLU-UHFFFAOYSA-N | C1=CC=C(C(=C1)N=C=S)Cl | 1696 | 1 | 1 | 1 | 1 | 1 | 1 |
| 1-Chloro-3-isocyanatobenzene (C7H4ClNO) | HHIRBXHEYVDUAM-UHFFFAOYSA-N | C1=CC(=CC(=C1)Cl)N=C=O | 30 | 2 | 2 | 2 | 2 | 2 | 2 |
| Benzene, 1-chloro-4-[(chloromethyl)thio]- (C7H6Cl2S) | XPJUCMIJGVAEGF-UHFFFAOYSA-N | C1=CC(=CC=C1SCCl)Cl | 9999 | 1 | 1 | 1 | 1 | 1 | 1 |
| 1,3-Benzenediol, 4,6-dichloro- (C6H4Cl2O2) | GRLQBYQELUWBIO-UHFFFAOYSA-N | C1=C(C(=CC(=C1O)Cl)Cl)O | 240 | 1 | 1 | 1 | 1 | 1 | 1 |
| Benzene, 1,4-dichloro-2-methoxy- (C7H6Cl2O) | QKMNFFSBZRGHDJ-UHFFFAOYSA-N | COC1=C(C=CC(=C1)Cl)Cl | 470 | 1 | 1 | 1 | 1 | 1 | 1 |
| 2,4,6-Trichloroaniline (C6H4Cl3N) | NATVSFWWYVJTAZ-UHFFFAOYSA-N | C1=C(C=C(C(=C1Cl)N)Cl)Cl | 969 | 1 | 1 | 1 | 1 | 1 | 1 |
| 3-Chlorobenzamide (C7H6ClNO) | MJTGQALMWUUPQM-UHFFFAOYSA-N | C1=CC(=CC(=C1)Cl)C(=O)N | 10 | 2 | 2 | 2 | 2 | 2 | 2 |
| Benzene, 1-(chloromethyl)-2-methoxy- (C8H9ClO) | UAWVMPOAIVZWFQ-UHFFFAOYSA-N | COC1=CC=CC=C1CCl | 8369 | 1 | 1 | 1 | 1 | 1 | 1 |
| Benzene, 1-chloro-2-methyl-4-methoxy (C8H9ClO) | SDGMUBWPXBSKCT-UHFFFAOYSA-N | CC1=C(C=CC(=C1)OC)Cl | 3373 | 1 | 1 | 1 | 1 | 1 | 1 |
| 3-Chloro-4-methoxytoluene (C8H9ClO) | VUZBRBKYGIQXMP-UHFFFAOYSA-N | CC1=CC(=C(C=C1)OC)Cl | 3833 | 1 | 1 | 1 | 1 | 1 | 1 |
| 2,5-Dichlorobenzylamine (C7H7Cl2N) | AKGJLIXNRPNPCH-UHFFFAOYSA-N | C1=CC(=C(C=C1Cl)CN)Cl | 9999 | 1 | 1 | 1 | 1 | 1 | 1 |
| 2,4-Dichlorobenzylamine (C7H7Cl2N) | SJUKJZSTBBSGHF-UHFFFAOYSA-N | C1=CC(=C(C=C1Cl)Cl)CN | 9999 | 1 | 1 | 1 | 1 | 1 | 1 |
| 3,4-Dichlorobenzyl alcohol (C7H6Cl2O) | FVJIUQSKXOYFKG-UHFFFAOYSA-N | C1=CC(=C(C=C1CO)Cl)Cl | 9669 | 1 | 1 | 1 | 1 | 1 | 1 |
| Benzaldehyde, 2,6-dichloro- (C7H4Cl2O) | DMIYKWPEFRFTPY-UHFFFAOYSA-N | C1=CC(=C(C(=C1)Cl)C=O)Cl | 50 | 2 | 2 | 2 | 2 | 2 | 2 |
| α,3,4-Trichlorotoluene (C7H5Cl3) | YZIFVWOCPGPNHB-UHFFFAOYSA-N | C1=CC(=C(C=C1CCl)Cl)Cl | 9999 | 1 | 1 | 1 | 1 | 1 | 1 |
| 3-Chloro-2-methylbenzonitrile (C8H6ClN) | FKFZTNLSUJCIMG-UHFFFAOYSA-N | CC1=C(C=CC=C1Cl)C#N | 9999 | 1 | 1 | 1 | 1 | 1 | 1 |
| Benzoyl chloride, 2-methyl- (C8H7ClO) | GPZXFICWCMCQPF-UHFFFAOYSA-N | CC1=CC=CC=C1C(=O)Cl | 9999 | 1 | 1 | 1 | 1 | 1 | 1 |
| Benzene, 1,3-bis(chloromethyl)- (C8H8Cl2) | GRJWOKACBGZOKT-UHFFFAOYSA-N | C1=CC(=CC(=C1)CCl)CCl | 9999 | 1 | 1 | 1 | 1 | 1 | 1 |
| 4-(Chloromethyl)-1-ethylbenzene (C9H11Cl) | DUBCVXSYZVTCOC-UHFFFAOYSA-N | CCC1=CC=C(C=C1)CCl | 9999 | 1 | 1 | 1 | 1 | 1 | 1 |
| 3,5-Dimethylbenzyl chloride (C9H11Cl) | FYNVRRYQTHUESZ-UHFFFAOYSA-N | CC1=CC(=CC(=C1)CCl)C | 9999 | 1 | 1 | 1 | 1 | 1 | 1 |
| Benzene, 2-(chloromethyl)-1,4-dimethyl- (C9H11Cl) | PECXPZGFZFGDRD-UHFFFAOYSA-N | CC1=CC(=C(C=C1)C)CCl | 9999 | 1 | 1 | 1 | 1 | 1 | 1 |
| Benzene, (3-chloroallyl)- (C9H9Cl) | JJTUJRVKTPSEFZ-XBXARRHUSA-N | C1=CC=C(C=C1)C/C=C/Cl | 9999 | 1 | 1 | 1 | 1 | 1 | 1 |
| Cyclopropanecarboxylic acid, 3-chloroprop-2-enyl ester (C7H9ClO2) | QAJSCZWJFSRULT-DAFODLJHSA-N | C1CC1C(=O)OC/C=C/Cl | 2563 | 1 | 1 | 1 | 1 | 1 | 1 |
| 6-Chloropurine (C5H3ClN4) | ZKBQDFAWXLTYKS-UHFFFAOYSA-N | C1=NC2=C(N1)C(=NC=N2)Cl | 5882 | 1 | 1 | 1 | 1 | 1 | 1 |
| 2-Chlorobenzimidazole (C7H5ClN2) | AYPSHJCKSDNETA-UHFFFAOYSA-N | C1=CC=C2C(=C1)NC(=N2)Cl | 821 | 1 | 1 | 1 | 1 | 1 | 1 |
| Phosphonic acid, (2-chloroethyl)-, diethyl ester (C6H14ClO3P) | GMDLEOVIACJWTD-UHFFFAOYSA-N | CCOP(=O)(CCCl)OCC | 7725 | 1 | 1 | 1 | 1 | 1 | 1 |
| Trichloroacetic acid, but-3-yn-2-yl ester (C6H5Cl3O2) | KEBRGWUCOLOSMN-UHFFFAOYSA-N | CC(C#C)OC(=O)C(Cl)(Cl)Cl | 130 | 1 | 1 | 1 | 1 | 1 | 1 |
| Butyl trichloroacetate (C6H9Cl3O2) | SECVZLDDYUWJAC-UHFFFAOYSA-N | CCCCOC(=O)C(Cl)(Cl)Cl | 0 | 2 | 2 | 2 | 2 | 2 | 2 |
| 3-Chlorohexanoic acid, chloromethyl ester (C7H12Cl2O2) | FWVZTAJOBMVDDT-UHFFFAOYSA-N | CCCC(CC(=O)OCCl)Cl | 290 | 1 | 2 | 2 | 2 | 2 | 2 |
| 2-Chlorohexanoic acid, chloromethyl ester (C7H12Cl2O2) | SLCVMKYCEWYVKS-UHFFFAOYSA-N | CCCCC(C(=O)OCCl)Cl | 0 | 2 | 2 | 2 | 2 | 2 | 2 |
| Hexyl chloroacetate (C8H15ClO2) | OJGRZJILAIHWIY-UHFFFAOYSA-N | CCCCCCOC(=O)CCl | 0 | 2 | 2 | 2 | 2 | 2 | 2 |
| 3-Chlorodecane (C10H21Cl) | SMVZPOXWOUHGQI-UHFFFAOYSA-N | CCCCCCCC(CC)Cl | 622 | 1 | 2 | 2 | 2 | 2 | 2 |
| Trichloroacetic acid, 3-chloroprop-2-enyl ester (C5H4Cl4O2) | KPJDOQORQYKCEB-OWOJBTEDSA-N | C(/C=C/Cl)OC(=O)C(Cl)(Cl)Cl | 240 | 1 | 1 | 1 | 1 | 1 | 1 |
| Phenol, 2-chloro-4-nitro- (C6H4ClNO3) | BOFRXDMCQRTGII-UHFFFAOYSA-N | C1=CC(=C(C=C1[N+](=O)[O-])Cl)O | 30 | 2 | 2 | 2 | 2 | 2 | 2 |
| 2-Chloro-4-methoxyphenol, methyl ether (C8H9ClO2) | QMXZSRVFIWACJH-UHFFFAOYSA-N | COC1=CC(=C(C=C1)OC)Cl | 0 | 2 | 2 | 2 | 2 | 2 | 2 |
| Benzene, 2,4-dichloro-1-nitro- (C6H3Cl2NO2) | QUIMTLZDMCNYGY-UHFFFAOYSA-N | C1=CC(=C(C=C1Cl)Cl)[N+](=O)[O-] | 0 | 2 | 2 | 2 | 2 | 2 | 2 |
| 3,5-Dichlorophenyl isocyanate (C7H3Cl2NO) | XEFUJGURFLOFAN-UHFFFAOYSA-N | C1=C(C=C(C=C1Cl)Cl)N=C=O | 0 | 2 | 2 | 2 | 2 | 2 | 2 |
| 2-Methoxy-3,6-dichloro-phenol (C7H6Cl2O2) | OBRQSFBOZCMSTK-UHFFFAOYSA-N | COC1=C(C=CC(=C1O)Cl)Cl | 250 | 1 | 1 | 1 | 1 | 1 | 2 |
| Benzene, 1,2,4-trichloro-5-methoxy- (C7H5Cl3O) | SXKBHOQOOGRFJF-UHFFFAOYSA-N | COC1=CC(=C(C=C1Cl)Cl)Cl | 240 | 1 | 1 | 1 | 1 | 1 | 1 |
| Phenol, 2,3,4,6-tetrachloro- (C6H2Cl4O) | VGVRPFIJEJYOFN-UHFFFAOYSA-N | C1=C(C(=C(C(=C1Cl)Cl)Cl)O)Cl | 160 | 1 | 1 | 1 | 1 | 1 | 1 |
| Desisopropylatrazine (C5H8ClN5) | IVENSCMCQBJAKW-UHFFFAOYSA-N | CCNC1=NC(=NC(=N1)N)Cl | 0 | 2 | 2 | 2 | 2 | 2 | 2 |
| 1,3,5-Triazin-2-amine, 4,6-dichloro-N-ethyl- (C5H6Cl2N4) | ACAHVXOSWOUZAB-UHFFFAOYSA-N | CCNC1=NC(=NC(=N1)Cl)Cl | 0 | 2 | 2 | 1 | 1 | 1 | 1 |
| 3-Chlorobenzhydrazide (C7H7ClN2O) | PHRDZSRVSVNQRN-UHFFFAOYSA-N | C1=CC(=CC(=C1)Cl)C(=O)NN | 0 | 2 | 2 | 2 | 2 | 2 | 2 |
| Benzoic acid, 2-amino-5-chloro- (C7H6ClNO2) | IFXKXCLVKQVVDI-UHFFFAOYSA-N | C1=CC(=C(C=C1Cl)C(=O)O)N | 60 | 2 | 2 | 2 | 2 | 2 | 2 |
| Acetic acid, 3-chlorophenyl ester (C8H7ClO2) | GQTKYLQYHPTULY-UHFFFAOYSA-N | CC(=O)OC1=CC(=CC=C1)Cl | 0 | 2 | 2 | 2 | 2 | 2 | 2 |
| Benzoic acid, 2,6-dichloro- (C7H4Cl2O2) | MRUDNSFOFOQZDA-UHFFFAOYSA-N | C1=CC(=C(C(=C1)Cl)C(=O)O)Cl | 230 | 1 | 1 | 2 | 2 | 2 | 2 |
| 3,5-Dichlorobenzoic acid (C7H4Cl2O2) | CXKCZFDUOYMOOP-UHFFFAOYSA-N | C1=C(C=C(C=C1Cl)Cl)C(=O)O | 302 | 1 | 2 | 2 | 2 | 2 | 2 |
| 1,3-dichloro-2-(methoxymethyl)benzene (C8H8Cl2O) | QBKBHXIQLAMKOB-UHFFFAOYSA-N | COCC1=C(C=CC=C1Cl)Cl | 9999 | 1 | 1 | 2 | 1 | 1 | 2 |
| 2,4,6-Trichlorobenzonitrile (C7H2Cl3N) | PGODHCIOIPODFE-UHFFFAOYSA-N | C1=C(C=C(C(=C1Cl)C#N)Cl)Cl | 1780 | 1 | 1 | 1 | 1 | 1 | 1 |
| Benzoyl chloride, 3,4-dichloro- (C7H3Cl3O) | VTXNOVCTHUBABW-UHFFFAOYSA-N | C1=CC(=C(C=C1C(=O)Cl)Cl)Cl | 9999 | 1 | 1 | 1 | 1 | 1 | 1 |
| m-Chlorophenylacetic acid (C8H7ClO2) | WFPMUFXQDKMVCO-UHFFFAOYSA-N | C1=CC(=CC(=C1)Cl)CC(=O)O | 30 | 2 | 1 | 2 | 1 | 1 | 2 |
| 4-Methoxy-3-methylbenzyl chloride (C9H11ClO) | BHEHNICAPZVKRH-UHFFFAOYSA-N | CC1=C(C=CC(=C1)CCl)OC | 9999 | 1 | 1 | 1 | 1 | 1 | 1 |
| 2,6-Dichloroacetophenone (C8H6Cl2O) | HYBDSXBLGCQKRE-UHFFFAOYSA-N | CC(=O)C1=C(C=CC=C1Cl)Cl | 0 | 2 | 2 | 2 | 2 | 2 | 2 |
| Benzene, (2,2,2-trichloroethyl)- (C8H7Cl3) | XFEKIQFBJSDMQB-UHFFFAOYSA-N | C1=CC=C(C=C1)CC(Cl)(Cl)Cl | 486 | 1 | 1 | 1 | 1 | 1 | 1 |
| Dichloroacetic acid, morpholide (C6H9Cl2NO2) | SPDHGKQMFGYCHN-UHFFFAOYSA-N | C1COCCN1C(=O)C(Cl)Cl | 851 | 1 | 1 | 1 | 1 | 1 | 1 |
| p-(Chlorophenyl)acetone (C9H9ClO) | WEJRYKSUUFKMBC-UHFFFAOYSA-N | CC(=O)CC1=CC=C(C=C1)Cl | 0 | 2 | 2 | 2 | 2 | 2 | 2 |
| Cyclopropane, 1,1-dichloro-2,2,3-triethyl- (C9H16Cl2) | HZZCFNUFGQXPQC-UHFFFAOYSA-N | CCC1C(C1(Cl)Cl)(CC)CC | 1401 | 1 | 1 | 2 | 1 | 1 | 1 |
| 6-Chloro-2,4-dihydroxy-1,3-dimethylpyrimidine (C6H7ClN2O2) | VATQPUHLFQHDBD-UHFFFAOYSA-N | CN1C(=CC(=O)N(C1=O)C)Cl | 20 | 2 | 1 | 1 | 1 | 1 | 1 |
| 8-Chloroquinoline (C9H6ClN) | RUSMDMDNFUYZTM-UHFFFAOYSA-N | C1=CC2=C(C(=C1)Cl)N=CC=C2 | 2589 | 1 | 1 | 1 | 1 | 1 | 1 |
| Bornyl chloride (C10H17Cl) | XXZAOMJCZBZKPV-UHFFFAOYSA-N | CC1(C2CCC1(C(C2)Cl)C)C | 709 | 1 | 2 | 2 | 2 | 1 | 2 |
| Pentyl trichloroacetate (C7H11Cl3O2) | LZJOVWVTJGLPFN-UHFFFAOYSA-N | CCCCCOC(=O)C(Cl)(Cl)Cl | 0 | 2 | 2 | 2 | 2 | 2 | 2 |
| Acetic acid, trichloro-, 3-methylbutyl ester (C7H11Cl3O2) | HBNLTLLITHPMDZ-UHFFFAOYSA-N | CC(C)CCOC(=O)C(Cl)(Cl)Cl | 40 | 2 | 2 | 2 | 2 | 2 | 2 |
| Propanoic acid, 3-chloro, hexyl ester (C9H17ClO2) | PYOAHWWMEFRKLM-UHFFFAOYSA-N | CCCCCCOC(=O)CCCl | 0 | 2 | 2 | 2 | 2 | 2 | 2 |
| 10-Chloro-1-decanol (C10H21ClO) | OTUSESJECXGMIV-UHFFFAOYSA-N | C(CCCCCCl)CCCCO | 0 | 2 | 2 | 2 | 2 | 2 | 2 |
| Formamidine, 3,3-dimethyl-1-(4-chlorophenyl) (C9H11ClN2) | ZPTXBCJETBDOAT-UHFFFAOYSA-N | CN(C)C=NC1=CC=C(C=C1)Cl | 0 | 2 | 2 | 2 | 2 | 2 | 1 |
| Benzene, 1,2,4-trichloro-5-nitro- (C6H2Cl3NO2) | IBRBMZRLVYKVRF-UHFFFAOYSA-N | C1=C(C(=CC(=C1Cl)Cl)Cl)[N+](=O)[O-] | 20 | 2 | 2 | 2 | 2 | 2 | 2 |
| 2-(4-Chlorophenoxy)thioacetamide (C8H8ClNOS) | AIOQDHOVIDONNW-UHFFFAOYSA-N | C1=CC(=CC=C1OCC(=S)N)Cl | 0 | 2 | 2 | 2 | 2 | 2 | 2 |
| 4-Nitrobenzoyl chloride (C7H4ClNO3) | SKDHHIUENRGTHK-UHFFFAOYSA-N | C1=CC(=CC=C1C(=O)Cl)[N+](=O)[O-] | 9999 | 1 | 1 | 1 | 1 | 1 | 1 |
| 3',4'-Dichloroacetanilide (C8H7Cl2NO) | SCYGGCAQZFJGRF-UHFFFAOYSA-N | CC(=O)NC1=CC(=C(C=C1)Cl)Cl | 0 | 2 | 2 | 2 | 2 | 2 | 2 |
| Phenol, 3,4-dichloro-, acetate (C8H6Cl2O2) | OSKGYRIYNMZFSJ-UHFFFAOYSA-N | CC(=O)OC1=CC(=C(C=C1)Cl)Cl | 0 | 2 | 2 | 2 | 2 | 2 | 2 |
| Phenol, 2,5-dichloro-, acetate (C8H6Cl2O2) | XSJDGJRHRDWQOR-UHFFFAOYSA-N | CC(=O)OC1=C(C=CC(=C1)Cl)Cl | 0 | 2 | 2 | 2 | 2 | 2 | 2 |
| Propanamide, N-(4-chlorophenyl)- (C9H10ClNO) | FFHGCIYKPNISDY-UHFFFAOYSA-N | CCC(=O)NC1=CC=C(C=C1)Cl | 0 | 2 | 1 | 2 | 2 | 2 | 1 |
| 2-Chloropropionanilide (C9H10ClNO) | VCVUMBWLSGNGFA-UHFFFAOYSA-N | CCC(=O)NC1=CC=CC=C1Cl | 2000 | 1 | 1 | 1 | 1 | 1 | 1 |
| Benzoic acid, 3-chloro-, ethyl ester (C9H9ClO2) | LVFRSNCBCHABAM-UHFFFAOYSA-N | CCOC(=O)C1=CC(=CC=C1)Cl | 200 | 1 | 1 | 1 | 1 | 2 | 2 |
| Benzene, (4-chlorobutoxy)- (C10H13ClO) | JKXCPAVECBFBOC-UHFFFAOYSA-N | C1=CC=C(C=C1)OCCCCCl | 10 | 2 | 2 | 2 | 2 | 2 | 2 |
| Chlorothymol (C10H13ClO) | KFZXVMNBUMVKLN-UHFFFAOYSA-N | CC1=CC(=C(C=C1Cl)C(C)C)O | 349 | 1 | 1 | 1 | 1 | 2 | 1 |
| 1-Chloromethyl-3-(1,1-dimethylethyl)benzene (C11H15Cl) | QZWCABOLGIVZCP-UHFFFAOYSA-N | CC(C)(C)C1=CC=CC(=C1)CCl | 470 | 1 | 1 | 2 | 1 | 1 | 2 |
| β-BHC (C6H6Cl6) | JLYXXMFPNIAWKQ-UHFFFAOYSA-N | C1(C(C(C(C(C1Cl)Cl)Cl)Cl)Cl)Cl | 159 | 1 | 1 | 1 | 1 | 1 | 1 |
| m-Chlorocinnamic acid (C9H7ClO2) | FFKGOJWPSXRALK-SNAWJCMRSA-N | C1=CC(=CC(=C1)Cl)/C=C/C(=O)O | 4049 | 1 | 1 | 1 | 1 | 1 | 1 |
| p-Benzoquinone, 2,3,5,6-tetrachloro- (C6Cl4O2) | UGNWTBMOAKPKBL-UHFFFAOYSA-N | C1(=C(C(=O)C(=C(C1=O)Cl)Cl)Cl)Cl | 4569 | 1 | 1 | 1 | 1 | 1 | 1 |
| Naphthalene, 1,4-dichloro- (C10H6Cl2) | JDPKCYMVSKDOGS-UHFFFAOYSA-N | C1=CC=C2C(=C1)C(=CC=C2Cl)Cl | 2019 | 1 | 1 | 1 | 1 | 1 | 1 |
| 6-Chloropiperonal (C8H5ClO3) | VRNADRCOROWLJC-UHFFFAOYSA-N | C1OC2=C(O1)C=C(C(=C2)C=O)Cl | 0 | 2 | 1 | 2 | 1 | 2 | 2 |
| 6-Chloro-2-methylquinoline (C10H8ClN) | OCCIBGIEIBQGAJ-UHFFFAOYSA-N | CC1=NC2=C(C=C1)C=C(C=C2)Cl | 2758 | 1 | 1 | 1 | 1 | 1 | 1 |
| Malonic acid, 2-chloropropyl ethyl ester (C8H13ClO4) | LNNJMHLJYAMEAG-UHFFFAOYSA-N | CCOC(=O)CC(=O)OCC(C)Cl | 50 | 2 | 2 | 2 | 2 | 2 | 2 |
| 8-Chlorooctanoic acid, chloromethyl ester (C9H16Cl2O2) | YEDAJMGEJCGDOV-UHFFFAOYSA-N | C(CCCC(=O)OCCl)CCCCl | 0 | 2 | 2 | 2 | 2 | 2 | 2 |
| Heptyl dichloroacetate (C9H16Cl2O2) | DABKLGRTFPUJHU-UHFFFAOYSA-N | CCCCCCCOC(=O)C(Cl)Cl | 0 | 2 | 2 | 2 | 2 | 2 | 2 |
| 5-Chlorovaleric acid, pentyl ester (C10H19ClO2) | LAYFAERBACNODJ-UHFFFAOYSA-N | CCCCCOC(=O)CCCCCl | 300 | 1 | 1 | 2 | 2 | 2 | 2 |
| nonanoic acid, chloromethyl ester (C10H19ClO2) | NNVIRQBZFULPEI-UHFFFAOYSA-N | CCCCCCCCC(=O)OCCl | 0 | 2 | 2 | 2 | 2 | 2 | 2 |
| Benzene, 4-chloro-1,2-dinitro- (C6H3ClN2O4) | QVQSOXMXXFZAKU-UHFFFAOYSA-N | C1=CC(=C(C=C1Cl)[N+](=O)[O-])[N+](=O)[O-] | 0 | 2 | 2 | 2 | 2 | 2 | 2 |
| 1-Chloro-2,4-dinitrobenzene (C6H3ClN2O4) | VYZAHLCBVHPDDF-UHFFFAOYSA-N | C1=CC(=C(C=C1[N+](=O)[O-])[N+](=O)[O-])Cl | 0 | 2 | 2 | 2 | 2 | 2 | 2 |
| Monuron (C9H11ClN2O) | BMLIZLVNXIYGCK-UHFFFAOYSA-N | CN(C)C(=O)NC1=CC=C(C=C1)Cl | 30 | 2 | 2 | 2 | 2 | 2 | 2 |
| Benzene, 3,4,5-trichloro-1,2-dimethoxy (C8H7Cl3O2) | VKNITLPENCJQOP-UHFFFAOYSA-N | COC1=CC(=C(C(=C1OC)Cl)Cl)Cl | 0 | 2 | 2 | 2 | 2 | 2 | 2 |
| Phenol, 2,3,5,6-tetrachloro-4-methoxy- (C7H4Cl4O2) | XIWJLPHQDBDOAN-UHFFFAOYSA-N | COC1=C(C(=C(C(=C1Cl)Cl)O)Cl)Cl | 97 | 2 | 2 | 2 | 2 | 2 | 2 |
| 5-Chloro-2-nitrobenzoic acid (C7H4ClNO4) | ZKUYSJHXBFFGPU-UHFFFAOYSA-N | C1=CC(=C(C=C1Cl)C(=O)O)[N+](=O)[O-] | 0 | 2 | 2 | 2 | 2 | 2 | 2 |
| Carbamic acid, 4-chlorophenyl, ethyl ester (C9H10ClNO2) | WSKXXIMERYQVGJ-UHFFFAOYSA-N | CCOC(=O)NC1=CC=C(C=C1)Cl | 0 | 2 | 2 | 2 | 2 | 2 | 2 |
| 2-Chloro-4-methoxyphenol, acetate (C9H9ClO3) | OZFXOOSAUSDUCK-UHFFFAOYSA-N | CC(=O)OC1=C(C=C(C=C1)OC)Cl | 20 | 2 | 2 | 2 | 2 | 2 | 2 |
| 2-Chloro-5-nitrobenzoyl chloride (C7H3Cl2NO3) | OGLKKYALUKXVPQ-UHFFFAOYSA-N | C1=CC(=C(C=C1[N+](=O)[O-])C(=O)Cl)Cl | 9999 | 1 | 1 | 1 | 1 | 1 | 1 |
| Chloroacetic acid, 3,4-dichlorophenyl ester (C8H5Cl3O2) | CNALHVZXVPTLGY-UHFFFAOYSA-N | C1=CC(=C(C=C1OC(=O)CCl)Cl)Cl | 0 | 2 | 2 | 2 | 2 | 2 | 2 |
| Benzene, 1,2,3,5-tetrachloro-4-ethoxy- (C8H6Cl4O) | OMGPLLBLNOWRAA-UHFFFAOYSA-N | CCOC1=C(C(=C(C=C1Cl)Cl)Cl)Cl | 0 | 2 | 2 | 2 | 2 | 2 | 2 |
| Chloroacetic acid, 4-cyanophenyl ester (C9H6ClNO2) | APQLVUNYVFVTPC-UHFFFAOYSA-N | C1=CC(=CC=C1C#N)OC(=O)CCl | 0 | 2 | 2 | 2 | 2 | 2 | 2 |
| propanil (C9H9Cl2NO) | LFULEKSKNZEWOE-UHFFFAOYSA-N | CCC(=O)NC1=CC(=C(C=C1)Cl)Cl | 22 | 2 | 2 | 2 | 2 | 2 | 2 |
| Propanamide, N-(3-methylphenyl)-3-chloro- (C10H12ClNO) | XZPLSUAKDONHQY-UHFFFAOYSA-N | CC1=CC(=CC=C1)NC(=O)CCCl | 70 | 2 | 1 | 1 | 1 | 1 | 1 |
| Benzoic acid, 4-chloro, propyl ester (C10H11ClO2) | BLEFFSGNRQPNCA-UHFFFAOYSA-N | CCCOC(=O)C1=CC=C(C=C1)Cl | 0 | 2 | 2 | 2 | 2 | 2 | 2 |
| Chloroacetic acid, 3,5-dimethylphenyl ester (C10H11ClO2) | WQVBITVXKZGBJJ-UHFFFAOYSA-N | CC1=CC(=CC(=C1)OC(=O)CCl)C | 0 | 2 | 1 | 2 | 2 | 1 | 2 |
| Benzoic acid, 2-chloro, 1-methylethyl ester (C10H11ClO2) | ZQVYNQNVFVRHMT-UHFFFAOYSA-N | CC(C)OC(=O)C1=CC=CC=C1Cl | 90 | 2 | 2 | 1 | 2 | 2 | 2 |
| 2-Methylpropionic acid, 4-chlorophenyl ester (C10H11ClO2) | WPCAYDFRIDVTNI-UHFFFAOYSA-N | CC(C)C(=O)OC1=CC=C(C=C1)Cl | 0 | 2 | 2 | 2 | 2 | 2 | 2 |
| 4-tert-butylbenzoyl chloride (C11H13ClO) | WNLMYNASWOULQY-UHFFFAOYSA-N | CC(C)(C)C1=CC=C(C=C1)C(=O)Cl | 9999 | 1 | 1 | 1 | 1 | 1 | 1 |
| 2,4-Dichloro-ω-nitrostyrene (C8H5Cl2NO2) | LIWIJBBAMBDXME-ONEGZZNKSA-N | C1=CC(=C(C=C1Cl)Cl)/C=C/[N+](=O)[O-] | 6603 | 1 | 2 | 2 | 1 | 2 | 1 |
| Naphthalene, 1,3,7-trichloro- (C10H5Cl3) | CFEUGIGSIREATC-UHFFFAOYSA-N | C1=CC(=CC2=C(C=C(C=C21)Cl)Cl)Cl | 1316 | 1 | 1 | 1 | 1 | 1 | 1 |
| Naphthalene, 1-(chloromethyl)-2-methyl- (C12H11Cl) | STBYRSZXHDPASK-UHFFFAOYSA-N | CC1=C(C2=CC=CC=C2C=C1)CCl | 9999 | 1 | 1 | 1 | 1 | 1 | 1 |
| 2-Chlorobenzo[b]thiophene-3-acetonitrile (C10H6ClNS) | CBWNFQCLFYLDJQ-UHFFFAOYSA-N | C1=CC=C2C(=C1)C(=C(S2)Cl)CC#N | 9999 | 1 | 1 | 1 | 1 | 2 | 1 |
| Succinic acid, 2,2-dichloroethyl ethyl ester (C8H12Cl2O4) | PFNFXPLZQOXXMK-UHFFFAOYSA-N | CCOC(=O)CCC(=O)OCC(Cl)Cl | 0 | 2 | 2 | 2 | 2 | 2 | 2 |
| Malonic acid, 2-chloropropyl propyl ester (C9H15ClO4) | IQLRLUZXLPNYMG-UHFFFAOYSA-N | CCCOC(=O)CC(=O)OCC(C)Cl | 30 | 2 | 2 | 2 | 2 | 2 | 2 |
| Propanamide, N,N-dibutyl-2-chloro- (C11H22ClNO) | AMBSMXGFOZRXNX-UHFFFAOYSA-N | CCCCN(CCCC)C(=O)C(C)Cl | 3934 | 1 | 1 | 1 | 1 | 1 | 1 |
| Succinic acid, monochloride, 3,3-dimethylbut-2-yl ester (C10H17ClO3) | PUJBLZZZGPXNRQ-UHFFFAOYSA-N | CC(C(C)(C)C)OC(=O)CCC(=O)Cl | 0 | 2 | 2 | 2 | 2 | 2 | 2 |
| 3-Chlorononanoic acid, chloromethyl ester (C10H18Cl2O2) | QADYLIOQVFEZKX-UHFFFAOYSA-N | CCCCCCC(CC(=O)OCCl)Cl | 3202 | 1 | 2 | 2 | 2 | 2 | 2 |
| Monolinuron (C9H11ClN2O2) | LKJPSUCKSLORMF-UHFFFAOYSA-N | CN(C(=O)NC1=CC=C(C=C1)Cl)OC | 0 | 2 | 2 | 2 | 2 | 2 | 2 |
| Benzene, 2-chloro-5-methyl-1,3-dinitro- (C7H5ClN2O4) | JMDVARRGYWIJGZ-UHFFFAOYSA-N | CC1=CC(=C(C(=C1)[N+](=O)[O-])Cl)[N+](=O)[O-] | 0 | 2 | 2 | 2 | 2 | 2 | 2 |
| 2,3-Dichlorobenzyl-N-methylcarbamate (C9H9Cl2NO2) | SESJCOBCXSACPH-UHFFFAOYSA-N | CNC(=O)OCC1=C(C(=CC=C1)Cl)Cl | 5425 | 1 | 1 | 2 | 2 | 2 | 2 |
| Dichloroacetic acid, 4-methoxyphenyl ester (C9H8Cl2O3) | FYSUBKYBGWIHKT-UHFFFAOYSA-N | COC1=CC=C(C=C1)OC(=O)C(Cl)Cl | 0 | 2 | 2 | 2 | 2 | 2 | 2 |
| Ethanol, 2-chloro-, 4-methylbenzenesulfonate (C9H11ClO3S) | ZXNMIUJDTOMBPV-UHFFFAOYSA-N | CC1=CC=C(C=C1)S(=O)(=O)OCCCl | 0 | 2 | 2 | 2 | 2 | 2 | 1 |
| Butyric acid, 3,4-dichlorophenyl ester (C10H10Cl2O2) | GVUWGECOKPZWDC-UHFFFAOYSA-N | CCCC(=O)OC1=CC(=C(C=C1)Cl)Cl | 0 | 2 | 2 | 2 | 2 | 2 | 2 |
| 4-Chlorobenzoic acid, but-3-yn-2-yl ester (C11H9ClO2) | LRAXWWFDHMSJAF-UHFFFAOYSA-N | CC(C#C)OC(=O)C1=CC=C(C=C1)Cl | 90 | 2 | 1 | 2 | 2 | 2 | 2 |
| 2-Chloropropionic acid, 3,5-dimethylphenyl ester (C11H13ClO2) | SJELLOBJQJSXPZ-UHFFFAOYSA-N | CC1=CC(=CC(=C1)OC(=O)C(C)Cl)C | 0 | 2 | 2 | 2 | 2 | 2 | 2 |
| Propanamide, N-(3-chlorophenyl)-2,2-dimethyl- (C11H14ClNO) | OGOQXGKPTWSHPS-UHFFFAOYSA-N | CC(C)(C)C(=O)NC1=CC(=CC=C1)Cl | 0 | 2 | 2 | 2 | 2 | 2 | 2 |
| 1-Propanone, 2-chloro-1-(2,5-dimethylphenyl)-2-methyl- (C12H15ClO) | VXXAHSALZMCFQS-UHFFFAOYSA-N | CC1=CC(=C(C=C1)C)C(=O)C(C)(C)Cl | 100 | 2 | 1 | 2 | 1 | 1 | 1 |
| 2-Chlorobenzoic acid, 3-chloroprop-2-enyl ester (C10H8Cl2O2) | GIMRXOTXLJWYLM-ZZXKWVIFSA-N | C1=CC=C(C(=C1)C(=O)OC/C=C/Cl)Cl | 1441 | 1 | 1 | 1 | 1 | 1 | 1 |
| m-toluylic acid, 3-chloroprop-2-enyl ester (C11H11ClO2) | MBSMOYXJNIUOJL-ZZXKWVIFSA-N | CC1=CC=CC(=C1)C(=O)OC/C=C/Cl | 2853 | 1 | 1 | 1 | 1 | 1 | 1 |
| Cyclopropanecarboxylic acid, 3-(2,2-dichloroethenyl)-2,2-dimethyl-, ethyl ester (C10H14Cl2O2) | QPTWKDNRYCGMJM-UHFFFAOYSA-N | CCOC(=O)C1C(C1(C)C)C=C(Cl)Cl | 2879 | 1 | 1 | 1 | 1 | 1 | 1 |
| 3-cyclopentylpropionic acid, 3-chloroprop-2-enyl ester (C11H17ClO2) | QXWNSXGSUGCIHO-FPYGCLRLSA-N | C1CCC(C1)CCC(=O)OC/C=C/Cl | 2983 | 1 | 1 | 1 | 1 | 1 | 1 |
| Naphthalene, 1,3,5,7-tetrachloro- (C10H4Cl4) | OTTCXKPQKOLSJN-UHFFFAOYSA-N | C1=C(C=C(C2=CC(=CC(=C21)Cl)Cl)Cl)Cl | 896 | 1 | 1 | 1 | 1 | 1 | 1 |
| [1,1'-Biphenyl]-4-ol, 4'-chloro- (C12H9ClO) | ICVFJPSNAUMFCW-UHFFFAOYSA-N | C1=CC(=CC=C1C2=CC=C(C=C2)Cl)O | 289 | 1 | 1 | 1 | 1 | 1 | 1 |
| PCB 4 (C12H8Cl2) | JAYCNKDKIKZTAF-UHFFFAOYSA-N | C1=CC=C(C(=C1)C2=CC=CC=C2Cl)Cl | 4250 | 1 | 1 | 1 | 1 | 1 | 1 |
| PCB 8 (C12H8Cl2) | UFNIBRDIUNVOMX-UHFFFAOYSA-N | C1=CC=C(C(=C1)C2=CC=C(C=C2)Cl)Cl | 256 | 1 | 1 | 1 | 1 | 1 | 1 |
| 1,1'-Biphenyl, 4-(chloromethyl)- (C13H11Cl) | HLQZCRVEEQKNMS-UHFFFAOYSA-N | C1=CC=C(C=C1)C2=CC=C(C=C2)CCl | 9999 | 1 | 1 | 1 | 1 | 1 | 1 |
| Succinic acid, 2,2-dichloroethyl propyl ester (C9H14Cl2O4) | FJMPKWLUBAMQEB-UHFFFAOYSA-N | CCCOC(=O)CCC(=O)OCC(Cl)Cl | 0 | 2 | 2 | 2 | 2 | 2 | 2 |
| 7-Chlorodecanoic acid, chloromethyl ester (C11H20Cl2O2) | WOXCLOSQVCNINZ-UHFFFAOYSA-N | CCCC(CCCCCC(=O)OCCl)Cl | 0 | 2 | 2 | 2 | 2 | 2 | 2 |
| 2-Chlorodecanoic acid, chloromethyl ester (C11H20Cl2O2) | ZBWKSSOXFUXWPK-UHFFFAOYSA-N | CCCCCCCCC(C(=O)OCCl)Cl | 0 | 2 | 2 | 2 | 2 | 2 | 2 |
| Nonyl dichloroacetate (C11H20Cl2O2) | XKMMRMJBZDCIHI-UHFFFAOYSA-N | CCCCCCCCCOC(=O)C(Cl)Cl | 0 | 2 | 2 | 2 | 2 | 2 | 2 |
| 3-Chloropropionic acid, 2-methyloct-5-yn-4-yl ester (C12H19ClO2) | UZTJDQYZKQSKGR-UHFFFAOYSA-N | CCC#CC(CC(C)C)OC(=O)CCCl | 0 | 2 | 1 | 1 | 1 | 1 | 1 |
| Ethanimidamide, N-[(6-chloro-3-pyridinyl)methyl]-N'-cyano-N-methyl-, (1E)- (C10H11ClN4) | WCXDHFDTOYPNIE-UHFFFAOYSA-N | CC(=NC#N)N(C)CC1=CN=C(C=C1)Cl | 581 | 1 | 1 | 1 | 1 | 1 | 1 |
| Butanoic acid, 4-(2,4-dichlorophenoxy)- (C10H10Cl2O3) | YIVXMZJTEQBPQO-UHFFFAOYSA-N | C1=CC(=C(C=C1Cl)Cl)OCCCC(=O)O | 0 | 2 | 2 | 2 | 2 | 2 | 2 |
| Rose acetate (C10H9Cl3O2) | JKRWZLOCPLZZEI-UHFFFAOYSA-N | CC(=O)OC(C1=CC=CC=C1)C(Cl)(Cl)Cl | 0 | 2 | 2 | 2 | 1 | 2 | 2 |
| Butanoic acid, 4-(4-chloro-2-methylphenoxy)- (C10H13ClO3) | LLWADFLAOKUBDR-UHFFFAOYSA-N | CC1=C(C=CC(=C1)Cl)OCCCC(=O)O | 0 | 2 | 2 | 2 | 2 | 2 | 2 |
| 5-Chloropentyl benzoate (C12H15ClO2) | XIOZPPYPTAEPGJ-UHFFFAOYSA-N | C1=CC=C(C=C1)C(=O)OCCCCCCl | 85 | 2 | 2 | 2 | 2 | 2 | 2 |
| Hexanamide, N-(3-chlorophenyl)- (C12H16ClNO) | BAUYPJBSRHYPFK-UHFFFAOYSA-N | CCCCCC(=O)NC1=CC(=CC=C1)Cl | 0 | 2 | 2 | 2 | 2 | 2 | 2 |
| Benzene, 1,1'-oxybis[4-chloro- (C12H8Cl2O) | URUJZHZLCCIILC-UHFFFAOYSA-N | C1=CC(=CC=C1OC2=CC=C(C=C2)Cl)Cl | 230 | 1 | 1 | 1 | 1 | 1 | 1 |
| 1,1'-Biphenyl,3-chloro-4-methoxy- (C13H11ClO) | SLBYCCHSURAIIK-UHFFFAOYSA-N | COC1=C(C=C(C=C1)C2=CC=CC=C2)Cl | 0 | 2 | 1 | 1 | 2 | 1 | 1 |
| PCB 33 (C12H7Cl3) | RIMXLXBUOQMDHV-UHFFFAOYSA-N | C1=CC=C(C(=C1)C2=CC(=C(C=C2)Cl)Cl)Cl | 195 | 1 | 1 | 1 | 1 | 1 | 1 |
| 2-Chlorobenzoic acid, morpholide (C11H12ClNO2) | ZRGHSGNYLSCFJY-UHFFFAOYSA-N | C1COCCN1C(=O)C2=CC=CC=C2Cl | 530 | 1 | 1 | 1 | 1 | 1 | 1 |
| 4-Chlorobenzoic acid, morpholide (C11H12ClNO2) | BGRFQNTYHMHVFJ-UHFFFAOYSA-N | C1COCCN1C(=O)C2=CC=C(C=C2)Cl | 50 | 2 | 2 | 2 | 2 | 2 | 2 |
| Bibenzyl, 3-chloro- (C14H13Cl) | NWVGKFZMBBPAJL-UHFFFAOYSA-N | C1=CC=C(C=C1)CCC2=CC(=CC=C2)Cl | 0 | 2 | 1 | 2 | 1 | 2 | 1 |
| Cyclopentanecarboxamide, N-(3-chlorophenyl)- (C12H14ClNO) | YOKCBEZMIXZJTA-UHFFFAOYSA-N | C1CCC(C1)C(=O)NC2=CC(=CC=C2)Cl | 0 | 2 | 2 | 2 | 2 | 1 | 2 |
| Methyl 4-chloroindolyl-3-acetate (C11H10ClNO2) | SYPGJEURLIGNPE-UHFFFAOYSA-N | COC(=O)CC1=CNC2=C1C(=CC=C2)Cl | 0 | 2 | 2 | 2 | 2 | 2 | 2 |
| 6,8-Dichlorochromone-3-carboxaldehyde (C10H4Cl2O3) | IHCCHRKNCOFDAJ-UHFFFAOYSA-N | C1=C(C=C2C(=C1Cl)OC=C(C2=O)C=O)Cl | 0 | 2 | 1 | 1 | 1 | 1 | 1 |
| Heptenophos (C9H12ClO4P) | GBAWQJNHVWMTLU-UHFFFAOYSA-N | COP(=O)(OC)OC1=C(C2C1CC=C2)Cl | 1310 | 1 | 1 | 1 | 2 | 1 | 1 |
| 5-Chloro-1,10-phenanthroline (C12H7ClN2) | XDUUQOQFSWSZSM-UHFFFAOYSA-N | C1=CC2=CC(=C3C=CC=NC3=C2N=C1)Cl | 1419 | 1 | 1 | 1 | 1 | 1 | 1 |
| Anthracene, 1-chloro- (C14H9Cl) | SRIHSAFSOOUEGL-UHFFFAOYSA-N | C1=CC=C2C=C3C(=CC2=C1)C=CC=C3Cl | 1129 | 1 | 1 | 1 | 1 | 1 | 1 |
| Succinic acid, di(2,2-dichloroethyl) ester (C8H10Cl4O4) | APJYUYGGYUZQET-UHFFFAOYSA-N | C(CC(=O)OCC(Cl)Cl)C(=O)OCC(Cl)Cl | 0 | 2 | 2 | 2 | 2 | 2 | 2 |
| 5-Chlorovaleric acid, octyl ester (C13H25ClO2) | KWLSFBAAGRONDK-UHFFFAOYSA-N | CCCCCCCCOC(=O)CCCCCl | 210 | 1 | 1 | 1 | 2 | 2 | 2 |
| Fumaric acid, 2,2-dichloroethyl isobutyl ester (C10H14Cl2O4) | IKJKPEUGXREZPB-ONEGZZNKSA-N | CC(C)COC(=O)/C=C/C(=O)OCC(Cl)Cl | 70 | 2 | 2 | 2 | 2 | 2 | 2 |
| Dichloroacetic acid, 2,7-dimethyloct-7-en-5-yn-4-yl (C12H16Cl2O2) | VSXCBHKTNKYJJP-UHFFFAOYSA-N | CC(C)CC(C#CC(=C)C)OC(=O)C(Cl)Cl | 841 | 1 | 2 | 1 | 1 | 1 | 1 |
| Diethyl 3-chlorophenyl phosphate (C10H14ClO4P) | OXUXCXDESUJMFE-UHFFFAOYSA-N | CCOP(=O)(OCC)OC1=CC(=CC=C1)Cl | 0 | 2 | 1 | 1 | 1 | 1 | 1 |
| 2,3-Dichlorophenol, isoBOC (C11H12Cl2O3) | SJOZPBMTTPQALN-UHFFFAOYSA-N | CC(C)COC(=O)OC1=C(C(=CC=C1)Cl)Cl | 0 | 2 | 2 | 2 | 2 | 2 | 2 |
| 5-Chlorovaleric acid, 4-methoxyphenyl ester (C12H15ClO3) | KGAVHPXSHLZLNB-UHFFFAOYSA-N | COC1=CC=C(C=C1)OC(=O)CCCCCl | 0 | 2 | 2 | 2 | 2 | 2 | 2 |
| 4-N,N-Bis(2-chloroethyl)amino-2-tolualdehyde (C12H15Cl2NO) | ZQIAXDULHBLZJE-UHFFFAOYSA-N | CC1=C(C=CC(=C1)N(CCCl)CCCl)C=O | 148 | 1 | 1 | 1 | 1 | 1 | 1 |
| Propyzamide (C12H11Cl2NO) | PHNUZKMIPFFYSO-UHFFFAOYSA-N | CC(C)(C#C)NC(=O)C1=CC(=CC(=C1)Cl)Cl | 0 | 2 | 2 | 1 | 2 | 2 | 2 |
| Pentanochlor (C13H18ClNO) | WGVWLKXZBUVUAM-UHFFFAOYSA-N | CCCC(C)C(=O)NC1=CC(=C(C=C1)C)Cl | 40 | 2 | 2 | 2 | 2 | 2 | 2 |
| 1-Chloromethyl-3,5-bis(1,1-dimethylethyl)benzene (C15H23Cl) | UNRGFCVSCXJGCL-UHFFFAOYSA-N | CC(C)(C)C1=CC(=CC(=C1)CCl)C(C)(C)C | 570 | 1 | 1 | 1 | 1 | 1 | 1 |
| PCB 66 (C12H6Cl4) | RKLLTEAEZIJBAU-UHFFFAOYSA-N | C1=CC(=C(C=C1C2=C(C=C(C=C2)Cl)Cl)Cl)Cl | 78 | 2 | 1 | 1 | 1 | 1 | 1 |
| PCB 77 (C12H6Cl4) | UQMGJOKDKOLIDP-UHFFFAOYSA-N | C1=CC(=C(C=C1C2=CC(=C(C=C2)Cl)Cl)Cl)Cl | 99 | 2 | 1 | 1 | 1 | 1 | 1 |
| PCB 42 (C12H6Cl4) | ALFHIHDQSYXSGP-UHFFFAOYSA-N | C1=CC(=C(C(=C1)Cl)Cl)C2=C(C=C(C=C2)Cl)Cl | 2478 | 1 | 1 | 1 | 1 | 1 | 1 |
| PCB 40 (C12H6Cl4) | VTLYHLREPCPDKX-UHFFFAOYSA-N | C1=CC(=C(C(=C1)Cl)Cl)C2=C(C(=CC=C2)Cl)Cl | 5099 | 1 | 1 | 1 | 1 | 1 | 1 |
| PCB 79 (C12H6Cl4) | QLCTXEMDCZGPCG-UHFFFAOYSA-N | C1=CC(=C(C=C1C2=CC(=CC(=C2)Cl)Cl)Cl)Cl | 54 | 2 | 1 | 1 | 1 | 1 | 1 |
| 2,4'-Dichlorobenzophenone (C13H8Cl2O) | YXMYPHLWXBXNFF-UHFFFAOYSA-N | C1=CC=C(C(=C1)C(=O)C2=CC=C(C=C2)Cl)Cl | 209 | 1 | 1 | 1 | 1 | 2 | 2 |
| Diphenylacetyl chloride (C14H11ClO) | MSYLETHDEIJMAF-UHFFFAOYSA-N | C1=CC=C(C=C1)C(C2=CC=CC=C2)C(=O)Cl | 500 | 1 | 1 | 1 | 1 | 1 | 1 |
| Benzamide, N-tetrahydrofurfuryl-4-chloro- (C12H14ClNO2) | ZMMAVVGZVPYQOX-UHFFFAOYSA-N | C1CC(OC1)CNC(=O)C2=CC=C(C=C2)Cl | 0 | 2 | 2 | 2 | 2 | 2 | 2 |
| 1,1-Bis(4-chlorophenyl)ethylene (C14H10Cl2) | IEAUXBMXWDAYID-UHFFFAOYSA-N | C=C(C1=CC=C(C=C1)Cl)C2=CC=C(C=C2)Cl | 3000 | 1 | 1 | 1 | 1 | 1 | 1 |
| 2-Thiophenecarboxylic acid, 3,4-dichlorophenyl ester (C11H6Cl2O2S) | BTWJLTDNAKWSDS-UHFFFAOYSA-N | C1=CSC(=C1)C(=O)OC2=CC(=C(C=C2)Cl)Cl | 0 | 2 | 2 | 2 | 2 | 2 | 2 |
| Acetamide, N-(3-chlorophenyl)-2-(2-thienyl)- (C12H10ClNOS) | RTZYPCPHOITSSY-UHFFFAOYSA-N | C1=CC(=CC(=C1)Cl)NC(=O)CC2=CC=CS2 | 0 | 2 | 2 | 2 | 2 | 2 | 2 |
| α-Chlordene (C10H6Cl6) | GSNLXLNDMLYEEK-UHFFFAOYSA-N | C1C2C3C(C1Cl)C(=C(C2(C(=C3Cl)Cl)Cl)Cl)Cl | 5941 | 1 | 1 | 1 | 1 | 1 | 1 |
| Chloroacetamide, N,N-dihexyl- (C14H28ClNO) | ZGQVQRKCUZEQLW-UHFFFAOYSA-N | CCCCCCN(CCCCCC)C(=O)CCl | 5305 | 1 | 1 | 1 | 1 | 1 | 1 |
| 11-Chlorododecanoic acid, chloromethyl ester (C13H24Cl2O2) | SWJDHWAMRFKAJU-UHFFFAOYSA-N | CC(CCCCCCCCCC(=O)OCCl)Cl | 0 | 2 | 2 | 2 | 2 | 2 | 2 |
| 3-Chlorododecanoic acid, chloromethyl ester (C13H24Cl2O2) | GQHFPKLBEGPYQL-UHFFFAOYSA-N | CCCCCCCCCC(CC(=O)OCCl)Cl | 5685 | 1 | 2 | 2 | 2 | 2 | 2 |
| 2-chloroethyl dodecanoate (C14H27ClO2) | PPRUSMUBWUQYRY-UHFFFAOYSA-N | CCCCCCCCCCCC(=O)OCCCl | 0 | 2 | 2 | 2 | 2 | 2 | 2 |
| Propanoic acid, 3-chloro, undecyl ester (C14H27ClO2) | MEBJHQDVCFHKMD-UHFFFAOYSA-N | CCCCCCCCCCCOC(=O)CCCl | 480 | 1 | 2 | 2 | 2 | 2 | 2 |
| Fumaric acid, isobutyl 2,2,2-trichloroethyl ester (C10H13Cl3O4) | VYCVEXWSKPJYKF-ONEGZZNKSA-N | CC(C)COC(=O)/C=C/C(=O)OCC(Cl)(Cl)Cl | 1111 | 1 | 2 | 1 | 1 | 1 | 1 |
| Dichloroacetic acid, 2,6-dimethylnon-1-en-3-yn-5-yl ester (C13H18Cl2O2) | WQHIJAZVGMYLTQ-UHFFFAOYSA-N | CCCC(C)C(C#CC(=C)C)OC(=O)C(Cl)Cl | 400 | 1 | 1 | 1 | 1 | 1 | 1 |
| 3-Chloropropionic acid, 2,6-dimethylnon-1-en-3-yn-5-yl ester (C14H21ClO2) | NOIASPFJFXMZDP-UHFFFAOYSA-N | CCCC(C)C(C#CC(=C)C)OC(=O)CCCl | 30 | 2 | 1 | 2 | 1 | 1 | 1 |
| 2,4,5-TB methyl ester (C11H11Cl3O3) | OTBMAATXBNOQHO-UHFFFAOYSA-N | COC(=O)CCCOC1=CC(=C(C=C1Cl)Cl)Cl | 0 | 2 | 2 | 2 | 2 | 2 | 2 |
| 2-Chloro-4-nitro-N,N-dipropylaniline (C12H17ClN2O2) | OYJJPFCGGNTTTO-UHFFFAOYSA-N | CCCN(CCC)C1=C(C=C(C=C1)[N+](=O)[O-])Cl | 15 | 2 | 2 | 2 | 2 | 2 | 2 |
| 6-Chlorohexanoic acid, 4-methoxyphenyl ester (C13H17ClO3) | WIUNAWMQMZCBAR-UHFFFAOYSA-N | COC1=CC=C(C=C1)OC(=O)CCCCCCl | 0 | 2 | 2 | 2 | 2 | 2 | 2 |
| Butanilicaine (C13H19ClN2O) | VWYQKFLLGRBICZ-UHFFFAOYSA-N | CCCCNCC(=O)NC1=C(C=CC=C1Cl)C | 930 | 1 | 2 | 1 | 2 | 2 | 1 |
| Fenson (C12H9ClO2S) | SPJOZZSIXXJYBT-UHFFFAOYSA-N | C1=CC=C(C=C1)S(=O)(=O)OC2=CC=C(C=C2)Cl | 0 | 2 | 2 | 2 | 2 | 2 | 2 |
| Diazene, bis(4-chlorophenyl)-, 1-oxide (C12H8Cl2N2O) | NMAZIJPSESMWSA-UHFFFAOYSA-N | C1=CC(=CC=C1N=[N+](C2=CC=C(C=C2)Cl)[O-])Cl | 0 | 2 | 2 | 2 | 2 | 2 | 2 |
| bis(4-chlorophenyl) sulphone (C12H8Cl2O2S) | GPAPPPVRLPGFEQ-UHFFFAOYSA-N | C1=CC(=CC=C1S(=O)(=O)C2=CC=C(C=C2)Cl)Cl | 10 | 2 | 2 | 2 | 1 | 2 | 2 |
| Tetrasul (C12H6Cl4S) | QUWSDLYBOVGOCW-UHFFFAOYSA-N | C1=CC(=CC=C1SC2=CC(=C(C=C2Cl)Cl)Cl)Cl | 290 | 1 | 1 | 2 | 1 | 2 | 1 |
| PCB 84 (C12H5Cl5) | QVWUJLANSDKRAH-UHFFFAOYSA-N | C1=CC(=C(C(=C1)Cl)Cl)C2=C(C=CC(=C2Cl)Cl)Cl | 2889 | 1 | 1 | 1 | 1 | 1 | 1 |
| PCB 92 (C12H5Cl5) | CRCBRZBVCDKPGA-UHFFFAOYSA-N | C1=CC(=C(C=C1Cl)C2=CC(=CC(=C2Cl)Cl)Cl)Cl | 1470 | 1 | 1 | 1 | 1 | 1 | 1 |
| PCB 86 (C12H5Cl5) | AIURIRUDHVDRFQ-UHFFFAOYSA-N | C1=CC=C(C(=C1)C2=CC(=C(C(=C2Cl)Cl)Cl)Cl)Cl | 2241 | 1 | 1 | 1 | 1 | 1 | 1 |
| PCB 83 (C12H5Cl5) | SUBRHHYLRGOTHL-UHFFFAOYSA-N | C1=CC(=C(C(=C1)Cl)Cl)C2=CC(=CC(=C2Cl)Cl)Cl | 3178 | 1 | 1 | 1 | 1 | 1 | 1 |
| 1,1'-Biphenyl, 2,2',4,5',6-Pentachloro- (C12H5Cl5) | PQHZWWBJPCNNGI-UHFFFAOYSA-N | C1=CC(=C(C=C1Cl)C2=C(C=C(C=C2Cl)Cl)Cl)Cl | 2004 | 1 | 1 | 1 | 1 | 1 | 1 |
| PCB 114 (C12H5Cl5) | SXZSFWHOSHAKMN-UHFFFAOYSA-N | C1=CC(=CC=C1C2=CC(=C(C(=C2Cl)Cl)Cl)Cl)Cl | 34 | 2 | 2 | 1 | 1 | 1 | 1 |
| Benzamide, N-(3-chlorophenyl)-4-methyl- (C14H12ClNO) | RUYDNAMXAAGBCA-UHFFFAOYSA-N | CC1=CC=C(C=C1)C(=O)NC2=CC(=CC=C2)Cl | 0 | 2 | 2 | 1 | 2 | 2 | 2 |
| Benzamide, N-(3-methylphenyl)-2-chloro- (C14H12ClNO) | QMUDBMHUUBUXHG-UHFFFAOYSA-N | CC1=CC(=CC=C1)NC(=O)C2=CC=CC=C2Cl | 450 | 1 | 1 | 1 | 2 | 2 | 2 |
| Benzhydryl 2-chloroethyl ether (C15H15ClO) | ZNVASENTCOLNJT-UHFFFAOYSA-N | C1=CC=C(C=C1)C(C2=CC=CC=C2)OCCCl | 0 | 2 | 1 | 1 | 2 | 1 | 1 |
| Benzophenone, 2-methylamino-5-chloro- (C14H12ClNO) | YHSCBLSYYDIOFJ-UHFFFAOYSA-N | C1=CC=C(C=C1)C(=O)C2=C(C=CC(=C2)Cl)CN | 301 | 1 | 1 | 1 | 1 | 1 | 1 |
| 3-Cyclopentylpropionic acid, 4-chlorophenyl ester (C14H17ClO2) | XVFXNEMYRISWTL-UHFFFAOYSA-N | C1CCC(C1)CCC(=O)OC2=CC=C(C=C2)Cl | 0 | 2 | 2 | 2 | 2 | 2 | 2 |
| Heptachlor (C10H5Cl7) | FRCCEHPWNOQAEU-UHFFFAOYSA-N | C1=CC(C2C1C3(C(=C(C2(C3(Cl)Cl)Cl)Cl)Cl)Cl)Cl | 898 | 1 | 1 | 1 | 1 | 1 | 1 |
| Succinic acid, pentyl 2,2,2-trichloroethyl ester (C11H17Cl3O4) | SOSGHDULTDVXJY-UHFFFAOYSA-N | CCCCCOC(=O)CCC(=O)OCC(Cl)(Cl)Cl | 0 | 2 | 2 | 2 | 2 | 2 | 2 |
| Succinic acid, 2-chloropropyl isohexyl ester (C13H23ClO4) | WCTQPSDQUVPTMD-UHFFFAOYSA-N | CC(C)CCCOC(=O)CCC(=O)OCC(C)Cl | 0 | 2 | 2 | 2 | 2 | 2 | 2 |
| Propanamide, N,N-dihexyl-2-chloro- (C15H30ClNO) | CPIHTZXGVWMATK-UHFFFAOYSA-N | CCCCCCN(CCCCCC)C(=O)C(C)Cl | 5575 | 1 | 1 | 1 | 1 | 1 | 1 |
| 5-Chlorovaleric acid, decyl ester (C15H29ClO2) | GDDOSYXFHTVPTR-UHFFFAOYSA-N | CCCCCCCCCCOC(=O)CCCCCl | 270 | 1 | 2 | 2 | 2 | 2 | 2 |
| Phenol, pentachloro-, trichloroacetate (C8Cl8O2) | WMUFBMFZOHGUPA-UHFFFAOYSA-N | C1(=C(C(=C(C(=C1Cl)Cl)Cl)Cl)Cl)OC(=O)C(Cl)(Cl)Cl | 20 | 2 | 2 | 2 | 2 | 2 | 2 |
| Hexanamide, N-ethyl-N-(3-methylphenyl)-6-chloro- (C15H22ClNO) | XOQPJEQXXHERBD-UHFFFAOYSA-N | CCN(C1=CC=CC(=C1)C)C(=O)CCCCCCl | 671 | 1 | 1 | 1 | 1 | 1 | 1 |
| Benzamide, N,N-dibutyl-4-chloro- (C15H22ClNO) | HBSQBZKONFNLBZ-UHFFFAOYSA-N | CCCCN(CCCC)C(=O)C1=CC=C(C=C1)Cl | 0 | 2 | 2 | 2 | 2 | 2 | 2 |
| Benzamide, N-(3-chlorophenyl)-2-methoxy- (C14H12ClNO2) | RLDGBZWAIDMLAE-UHFFFAOYSA-N | COC1=CC=CC=C1C(=O)NC2=CC(=CC=C2)Cl | 0 | 2 | 2 | 2 | 2 | 2 | 2 |
| PCB 139 (C12H4Cl6) | SPOPSCCFZQFGDL-UHFFFAOYSA-N | C1=CC(=C(C=C1Cl)Cl)C2=C(C(=C(C=C2Cl)Cl)Cl)Cl | 1276 | 1 | 1 | 1 | 1 | 1 | 1 |
| PCB 147 (C12H4Cl6) | AQONCPKMJSBHQT-UHFFFAOYSA-N | C1=CC(=C(C=C1Cl)Cl)C2=C(C(=CC(=C2Cl)Cl)Cl)Cl | 2213 | 1 | 1 | 1 | 1 | 1 | 1 |
| 4-Chloro-3-nitrobenzophenone (C13H8ClNO3) | YBDBYPQFIMSFJW-UHFFFAOYSA-N | C1=CC=C(C=C1)C(=O)C2=CC(=C(C=C2)Cl)[N+](=O)[O-] | 173 | 1 | 2 | 2 | 2 | 2 | 2 |
| 2-Phenylethyl 2-chlorobenzoate (C15H13ClO2) | FANZVPUMGJHASY-UHFFFAOYSA-N | C1=CC=C(C=C1)CCOC(=O)C2=CC=CC=C2Cl | 0 | 2 | 2 | 2 | 2 | 2 | 2 |
| o,p'-DDE (C14H8Cl4) | ZDYJWDIWLRZXDB-UHFFFAOYSA-N | C1=CC=C(C(=C1)C(=C(Cl)Cl)C2=CC=C(C=C2)Cl)Cl | 532 | 1 | 1 | 1 | 1 | 1 | 1 |
| 9,10-Anthracenedione, 1,8-dichloro- (C14H6Cl2O2) | VBQNYYXVDQUKIU-UHFFFAOYSA-N | C1=CC2=C(C(=C1)Cl)C(=O)C3=C(C2=O)C=CC=C3Cl | 4379 | 1 | 1 | 1 | 1 | 1 | 1 |
| Isobenzan (C9H4Cl8O) | LRWHHSXTGZSMSN-UHFFFAOYSA-N | C12C(C(OC1Cl)Cl)C3(C(=C(C2(C3(Cl)Cl)Cl)Cl)Cl)Cl | 747 | 1 | 1 | 1 | 1 | 1 | 1 |
| Succinic acid, 2,2-dichloroethyl heptyl ester (C13H22Cl2O4) | XYHOOJUYXZQYME-UHFFFAOYSA-N | CCCCCCCOC(=O)CCC(=O)OCC(Cl)Cl | 0 | 2 | 2 | 2 | 2 | 2 | 2 |
| Malonic acid, 8-chlorooctyl propyl ester (C14H25ClO4) | CSXNAOIJZYGGFP-UHFFFAOYSA-N | CCCOC(=O)CC(=O)OCCCCCCCCCl | 0 | 2 | 2 | 2 | 2 | 2 | 2 |
| Tetradecyl chloroacetate (C16H31ClO2) | INPWKHSGGJNIIM-UHFFFAOYSA-N | CCCCCCCCCCCCCCOC(=O)CCl | 0 | 2 | 2 | 2 | 2 | 2 | 2 |
| 5-chlorovaleric acid, undec-2-enyl ester (C16H29ClO2) | MMBYQPWSFIURFR-FMIVXFBMSA-N | CCCCCCCC/C=C/COC(=O)CCCCCl | 40 | 2 | 1 | 1 | 2 | 1 | 2 |
| Succinic acid, ethyl 2,3,5-trichlorophenyl ester (C12H11Cl3O4) | DMGJAPZJPKIMQE-UHFFFAOYSA-N | CCOC(=O)CCC(=O)OC1=CC(=CC(=C1Cl)Cl)Cl | 0 | 2 | 2 | 2 | 2 | 2 | 2 |
| Succinic acid, ethyl 2,3,6-trichlorophenyl ester (C12H11Cl3O4) | VQAWDMHUBWVGEG-UHFFFAOYSA-N | CCOC(=O)CCC(=O)OC1=C(C=CC(=C1Cl)Cl)Cl | 0 | 2 | 2 | 2 | 2 | 2 | 2 |
| β-Alanine, N-(2-chlorobenzoyl)-, butyl ester (C14H18ClNO3) | VUQQMELHTPWSLV-UHFFFAOYSA-N | CCCCOC(=O)CCNC(=O)C1=CC=CC=C1Cl | 100 | 2 | 2 | 2 | 2 | 2 | 2 |
| Fumaric acid, ethyl 3,4,5-trichlorophenyl ester (C12H9Cl3O4) | NPYGPOLDZDOIEX-ONEGZZNKSA-N | CCOC(=O)/C=C/C(=O)OC1=CC(=C(C(=C1)Cl)Cl)Cl | 0 | 2 | 2 | 2 | 2 | 2 | 2 |
| Fumaric acid, ethyl 2,4,6-trichlorophenyl ester (C12H9Cl3O4) | ZQUKPRSELNYMAD-ONEGZZNKSA-N | CCOC(=O)/C=C/C(=O)OC1=C(C=C(C=C1Cl)Cl)Cl | 0 | 2 | 2 | 2 | 2 | 2 | 2 |
| PCB 189 (C12H3Cl7) | XUAWBXBYHDRROL-UHFFFAOYSA-N | C1=C(C=C(C(=C1Cl)Cl)Cl)C2=CC(=C(C(=C2Cl)Cl)Cl)Cl | 0 | 2 | 1 | 2 | 2 | 1 | 2 |
| PCB 178 (C12H3Cl7) | WCIBKXHMIXUQHK-UHFFFAOYSA-N | C1=C(C=C(C(=C1Cl)Cl)C2=C(C(=CC(=C2Cl)Cl)Cl)Cl)Cl | 1991 | 1 | 1 | 1 | 1 | 1 | 1 |
| 2,6-Dichlorobenzyl ether (C14H10Cl4O) | NWYHVMDKERUNLM-UHFFFAOYSA-N | C1=CC(=C(C=C1Cl)Cl)COCC2=C(C=C(C=C2)Cl)Cl | 20 | 2 | 2 | 2 | 2 | 1 | 2 |
| Pyrifenox (C14H12Cl2N2O) | CKPCAYZTYMHQEX-UHFFFAOYSA-N | CON=C(CC1=CN=CC=C1)C2=C(C=C(C=C2)Cl)Cl | 981 | 1 | 1 | 1 | 1 | 1 | 1 |
| 2-amino-5-chlorobenzophenone, acetylated (C15H12ClNO2) | NHAUKYAYIYDFST-UHFFFAOYSA-N | CC(=O)NC1=C(C=C(C=C1)Cl)C(=O)C2=CC=CC=C2 | 11 | 2 | 2 | 2 | 2 | 2 | 2 |
| 3-Phenylpropionic acid, 3,4-dichlorophenyl ester (C15H12Cl2O2) | FQOCOCXQBBVPNZ-UHFFFAOYSA-N | C1=CC=C(C=C1)CCC(=O)OC2=CC(=C(C=C2)Cl)Cl | 0 | 2 | 2 | 2 | 2 | 2 | 2 |
| 1,2,4,7,8-Pentachlorodibenzo-p-dioxin (C12H3Cl5O2) | QUPLGUUISJOUPJ-UHFFFAOYSA-N | C1=C2C(=CC(=C1Cl)Cl)OC3=C(O2)C(=CC(=C3Cl)Cl)Cl | 680 | 1 | 1 | 1 | 1 | 1 | 1 |
| Medazepam (C16H15ClN2) | YLCXGBZIZBEVPZ-UHFFFAOYSA-N | CN1CCN=C(C2=C1C=CC(=C2)Cl)C3=CC=CC=C3 | 40 | 2 | 1 | 2 | 1 | 2 | 2 |
| Chlorflurenol, methyl ester (C15H11ClO3) | LINPVWIEWJTEEJ-UHFFFAOYSA-N | COC(=O)C1(C2=CC=CC=C2C3=C1C=C(C=C3)Cl)O | 0 | 2 | 2 | 2 | 2 | 2 | 2 |
| Endosulfan (C9H6Cl6O3S) | RDYMFSUJUZBWLH-UHFFFAOYSA-N | C1C2C(COS(=O)O1)C3(C(=C(C2(C3(Cl)Cl)Cl)Cl)Cl)Cl | 0 | 2 | 1 | 1 | 1 | 1 | 1 |
| Oxychlordane (C10H4Cl8O) | VWGNQYSIWFHEQU-UHFFFAOYSA-N | C12C(C(C3(C1O3)Cl)Cl)C4(C(=C(C2(C4(Cl)Cl)Cl)Cl)Cl)Cl | 2085 | 1 | 1 | 1 | 1 | 1 | 1 |
| Tris(1,3-dichioro-2-propyl) phosphate (C9H15Cl6O4P) | ASLWPAWFJZFCKF-UHFFFAOYSA-N | C(C(CCl)OP(=O)(OC(CCl)CCl)OC(CCl)CCl)Cl | 40 | 2 | 1 | 1 | 1 | 1 | 1 |
| Succinic acid, heptyl 2,2,2-trichloroethyl ester (C13H21Cl3O4) | NAQVIHHSOMSWFF-UHFFFAOYSA-N | CCCCCCCOC(=O)CCC(=O)OCC(Cl)(Cl)Cl | 0 | 2 | 2 | 2 | 2 | 2 | 2 |
| Fumaric acid, 8-chlorooctyl propyl ester (C15H25ClO4) | MGHZNNGUVBDAGD-MDZDMXLPSA-N | CCCOC(=O)/C=C/C(=O)OCCCCCCCCCl | 30 | 2 | 2 | 2 | 2 | 2 | 2 |
| Succinic acid, propyl 2,3,6-trichlorophenyl ester (C13H13Cl3O4) | ULGNTESKJOCJGR-UHFFFAOYSA-N | CCCOC(=O)CCC(=O)OC1=C(C=CC(=C1Cl)Cl)Cl | 0 | 2 | 2 | 2 | 2 | 2 | 2 |
| β-Alanine, N-(4-chlorobenzoyl)-, pentyl ester (C15H20ClNO3) | AEPLOPPDIMQBKI-UHFFFAOYSA-N | CCCCCOC(=O)CCNC(=O)C1=CC=C(C=C1)Cl | 0 | 2 | 2 | 2 | 2 | 2 | 2 |
| Benzoic acid, 2-chloro, decyl ester (C17H25ClO2) | XWNLBSXKHJVMLC-UHFFFAOYSA-N | CCCCCCCCCCOC(=O)C1=CC=CC=C1Cl | 0 | 2 | 2 | 2 | 2 | 2 | 2 |
| Benzamide, N-(2,5-dimethoxyphenyl)-4-chloro- (C15H14ClNO3) | HCVYUDNPZQUVRH-UHFFFAOYSA-N | COC1=CC(=C(C=C1)OC)NC(=O)C2=CC=C(C=C2)Cl | 20 | 2 | 2 | 2 | 2 | 2 | 2 |
| Dichloroacetic acid, 4-benzyloxyphenyl ester (C15H12Cl2O3) | BHDHMFXSIYFMCZ-UHFFFAOYSA-N | C1=CC=C(C=C1)COC2=CC=C(C=C2)OC(=O)C(Cl)Cl | 0 | 2 | 2 | 2 | 2 | 2 | 2 |
| cis-Captafol (C10H9Cl4NO2S) | JHRWWRDRBPCWTF-OLQVQODUSA-N | C1C=CC[C@H]2[C@@H]1C(=O)N(C2=O)SC(C(Cl)Cl)(Cl)Cl | 90 | 2 | 1 | 1 | 1 | 1 | 1 |
| Dienochlor (C10Cl10) | LWLJUMBEZJHXHV-UHFFFAOYSA-N | C1(=C(C(C(=C1Cl)Cl)(C2(C(=C(C(=C2Cl)Cl)Cl)Cl)Cl)Cl)Cl)Cl | 0 | 2 | 1 | 1 | 1 | 1 | 1 |
| 1-Naphthoic acid, 4-chlorophenyl ester (C17H11ClO2) | ZTNAIEDTVGAUOA-UHFFFAOYSA-N | C1=CC=C2C(=C1)C=CC=C2C(=O)OC3=CC=C(C=C3)Cl | 0 | 2 | 2 | 2 | 2 | 2 | 2 |
| 7-Aminoclonazepam (C15H12ClN3O) | HEFRPWRJTGLSSV-UHFFFAOYSA-N | C1C(=O)NC2=C(C=C(C=C2)N)C(=N1)C3=CC=CC=C3Cl | 2092 | 1 | 2 | 2 | 1 | 1 | 2 |
| Diazepam (C16H13ClN2O) | AAOVKJBEBIDNHE-UHFFFAOYSA-N | CN1C(=O)CN=C(C2=C1C=CC(=C2)Cl)C3=CC=CC=C3 | 643 | 1 | 1 | 1 | 1 | 1 | 2 |
| Adenosine, 2-chloro- (C10H12ClN5O4) | BIXYYZIIJIXVFW-UHFFFAOYSA-N | C1=NC2=C(N1C3C(C(C(O3)CO)O)O)N=C(N=C2N)Cl | 96 | 2 | 2 | 2 | 2 | 2 | 2 |
| Cyclopropanecarbonitrile, 1-(p-chlorophenyl)-2-(p-methoxyphenyl)- (C17H14ClNO) | UKBFLNJUMCRCGX-UHFFFAOYSA-N | COC1=CC=C(C=C1)C2CC2(C#N)C3=CC=C(C=C3)Cl | 1802 | 1 | 2 | 2 | 2 | 2 | 2 |
| Trichloroacetamide, N,N-diheptyl- (C16H30Cl3NO) | BVFXHEMDRLSTLE-UHFFFAOYSA-N | CCCCCCCN(CCCCCCC)C(=O)C(Cl)(Cl)Cl | 1672 | 1 | 1 | 1 | 1 | 1 | 1 |
| 2-chloroethyl hexadecanoate (C18H35ClO2) | CPFFARIYTPCNJA-UHFFFAOYSA-N | CCCCCCCCCCCCCCCC(=O)OCCCl | 0 | 2 | 2 | 2 | 2 | 2 | 2 |
| 5-Chlorovaleric acid, tridecyl ester (C18H35ClO2) | QUAVOGDQRCBTGN-UHFFFAOYSA-N | CCCCCCCCCCCCCOC(=O)CCCCCl | 320 | 1 | 2 | 2 | 2 | 2 | 2 |
| 5-Chlorovaleric acid, 2-tridecyl ester (C18H35ClO2) | CMCIREZBJQDCSX-UHFFFAOYSA-N | CCCCCCCCCCCC(C)OC(=O)CCCCCl | 0 | 2 | 2 | 2 | 2 | 2 | 2 |
| Chloroacetamide, N,N-dioctyl- (C18H36ClNO) | ASYPQLBYSSYEBE-UHFFFAOYSA-N | CCCCCCCCN(CCCCCCCC)C(=O)CCl | 5765 | 1 | 1 | 1 | 1 | 1 | 1 |
| 5-Chlorovaleric acid, 5-tridecyl ester (C18H35ClO2) | MEVZAAUZXNKQIB-UHFFFAOYSA-N | CCCCCCCCC(CCCC)OC(=O)CCCCCl | 0 | 2 | 2 | 2 | 2 | 2 | 2 |
| Propanamide, N-heptyl-N-octyl-2-chloro- (C18H36ClNO) | QBZXHNDGBKWEQP-UHFFFAOYSA-N | CCCCCCCCN(CCCCCCC)C(=O)C(C)Cl | 9999 | 1 | 1 | 1 | 1 | 1 | 1 |
| 5-chlorovaleric acid, tridec-2-ynyl ester (C18H31ClO2) | IWBWGAOOVXOGLS-UHFFFAOYSA-N | CCCCCCCCCCC#CCOC(=O)CCCCCl | 0 | 2 | 2 | 1 | 1 | 2 | 2 |
| Fumaric acid, 8-chlorooctyl isobutyl ester (C16H27ClO4) | MRHRYQMLUYULBY-MDZDMXLPSA-N | CC(C)COC(=O)/C=C/C(=O)OCCCCCCCCCl | 0 | 2 | 2 | 2 | 2 | 2 | 2 |
| 2,4,5-T Butoxyethyl ester (C14H17Cl3O4) | GLDWASBMYWLQGG-UHFFFAOYSA-N | CCCCOCCOC(=O)COC1=CC(=C(C=C1Cl)Cl)Cl | 0 | 2 | 2 | 2 | 2 | 2 | 2 |
| Sarcosine, N-(2-chlorobenzoyl)-, hexyl ester (C16H22ClNO3) | CCRNYVLCQLBOBV-UHFFFAOYSA-N | CCCCCCOC(=O)CN(C)C(=O)C1=CC=CC=C1Cl | 110 | 1 | 2 | 2 | 2 | 2 | 2 |
| Sarcosine, N-(4-chlorobenzoyl)-, isohexyl ester (C16H22ClNO3) | QKNRAHQLPYVFFY-UHFFFAOYSA-N | CC(C)CCCOC(=O)CN(C)C(=O)C1=CC=C(C=C1)Cl | 0 | 2 | 2 | 2 | 2 | 2 | 2 |
| Aramite (C15H23ClO4S) | YKFRAOGHWKADFJ-UHFFFAOYSA-N | CC(COC1=CC=C(C=C1)C(C)(C)C)OS(=O)OCCCl | 0 | 2 | 2 | 1 | 2 | 2 | 2 |
| Succinic acid, 3,5-dichlorophenyl pentyl ester (C15H18Cl2O4) | TXRLZUNUCPQBIJ-UHFFFAOYSA-N | CCCCCOC(=O)CCC(=O)OC1=CC(=CC(=C1)Cl)Cl | 0 | 2 | 2 | 2 | 2 | 2 | 2 |
| Pretilachlor (C17H26ClNO2) | YLPGTOIOYRQOHV-UHFFFAOYSA-N | CCCOCCN(C1=C(C=CC=C1CC)CC)C(=O)CCl | 10 | 2 | 2 | 1 | 1 | 1 | 1 |
| Fumaric acid, propyl 2,3,4,5-tetrachlorophenyl ester (C13H10Cl4O4) | AEDTXMHHILJJGC-ONEGZZNKSA-N | CCCOC(=O)/C=C/C(=O)OC1=CC(=C(C(=C1Cl)Cl)Cl)Cl | 0 | 2 | 2 | 2 | 2 | 2 | 2 |
| Fumaric acid, isobutyl 3,4,5-trichlorophenyl ester (C14H13Cl3O4) | ZYNNZTCANZASBH-ONEGZZNKSA-N | CC(C)COC(=O)/C=C/C(=O)OC1=CC(=C(C(=C1)Cl)Cl)Cl | 0 | 2 | 2 | 2 | 2 | 2 | 2 |
| Fumaric acid, isobutyl 2,4,6-trichlorophenyl ester (C14H13Cl3O4) | JJOFXKGOYZAVLR-ONEGZZNKSA-N | CC(C)COC(=O)/C=C/C(=O)OC1=C(C=C(C=C1Cl)Cl)Cl | 0 | 2 | 2 | 2 | 2 | 2 | 2 |
| Bis(3-chlorophenylsulphonyl)methane (C13H10Cl2O4S2) | OWITURUKFUPLIO-UHFFFAOYSA-N | C1=CC(=CC(=C1)Cl)S(=O)(=O)CS(=O)(=O)C2=CC(=CC=C2)Cl | 0 | 2 | 2 | 2 | 2 | 2 | 2 |
| PCB 206 (C12HCl9) | JFIMDKGRGPNPRQ-UHFFFAOYSA-N | C1=C(C(=C(C(=C1Cl)Cl)Cl)Cl)C2=C(C(=C(C(=C2Cl)Cl)Cl)Cl)Cl | 873 | 1 | 1 | 2 | 2 | 1 | 1 |
| o,p'-Methoxychlor (C16H15Cl3O2) | KNLLPAOBVIKLDE-UHFFFAOYSA-N | COC1=CC=C(C=C1)C(C2=CC=CC=C2OC)C(Cl)(Cl)Cl | 90 | 2 | 2 | 1 | 2 | 1 | 1 |
| tebuconazole (C16H22ClN3O) | PXMNMQRDXWABCY-UHFFFAOYSA-N | CC(C)(C)C(CCC1=CC=C(C=C1)Cl)(CN2C=NC=N2)O | 0 | 2 | 2 | 2 | 2 | 2 | 2 |
| 1-Naphthoic acid, 3,4-dichlorophenyl ester (C17H10Cl2O2) | OMRXSGMGDYFBMY-UHFFFAOYSA-N | C1=CC=C2C(=C1)C=CC=C2C(=O)OC3=CC(=C(C=C3)Cl)Cl | 0 | 2 | 2 | 2 | 2 | 2 | 2 |
| 1H-1,2,4-Triazole, 1-[[2-(2,4-dichlorophenyl)-4-ethyl-1,3-dioxolan-2-yl]methyl]- (C14H15Cl2N3O2) | DWRKFAJEBUWTQM-UHFFFAOYSA-N | CCC1COC(O1)(CN2C=NC=N2)C3=C(C=C(C=C3)Cl)Cl | 0 | 2 | 2 | 2 | 2 | 2 | 2 |
| trans-Nonachlor (C10H5Cl9) | OCHOKXCPKDPNQU-BBXWSCHTSA-N | [C@@H]12[C@@H](C(C(C1Cl)Cl)Cl)C3(C(=C(C2(C3(Cl)Cl)Cl)Cl)Cl)Cl | 3620 | 1 | 1 | 1 | 1 | 1 | 1 |
| 2-chlorobenzoic acid, 1-adamantylmethyl ester (C18H21ClO2) | KYXXVMRHEPJPPY-UHFFFAOYSA-N | C1C2CC3CC1CC(C2)(C3)COC(=O)C4=CC=CC=C4Cl | 0 | 2 | 2 | 2 | 2 | 2 | 2 |
| Succinic acid, decyl 2,2-dichloroethyl ester (C16H28Cl2O4) | SMHJYHBHJCREHK-UHFFFAOYSA-N | CCCCCCCCCCOC(=O)CCC(=O)OCC(Cl)Cl | 0 | 2 | 2 | 2 | 2 | 2 | 2 |
| Malonic acid, 2-chloropropyl undecyl ester (C17H31ClO4) | XNRXLJXCPKUKHW-UHFFFAOYSA-N | CCCCCCCCCCCOC(=O)CC(=O)OCC(C)Cl | 0 | 2 | 2 | 2 | 2 | 2 | 2 |
| Propanamide, N,N-dioctyl-3-chloro- (C19H38ClNO) | SLCJNBBZBLGJLW-UHFFFAOYSA-N | CCCCCCCCN(CCCCCCCC)C(=O)CCCl | 4964 | 1 | 1 | 1 | 1 | 1 | 1 |
| Propanamide, N,N-bis(2-ethylhexyl)-3-chloro- (C19H38ClNO) | LJNYXXQVVFXPTJ-UHFFFAOYSA-N | CCCCC(CC)CN(CC(CC)CCCC)C(=O)CCCl | 1511 | 1 | 1 | 1 | 1 | 1 | 1 |
| 2-chlorobenzoic acid, dodec-9-ynyl ester (C19H25ClO2) | SUDQXLLPAJDXDC-UHFFFAOYSA-N | CCC#CCCCCCCCCOC(=O)C1=CC=CC=C1Cl | 0 | 2 | 2 | 2 | 2 | 2 | 2 |
| Fumaric acid, isobutyl 2,3,4,6-tetrachlorophenyl ester (C14H12Cl4O4) | URUVAKSJVJOFGY-ONEGZZNKSA-N | CC(C)COC(=O)/C=C/C(=O)OC1=C(C(=C(C=C1Cl)Cl)Cl)Cl | 0 | 2 | 2 | 2 | 2 | 2 | 2 |
| Fumaric acid, isobutyl 2,3,4,5-tetrachlorophenyl ester (C14H12Cl4O4) | LSWQEYJKXOOGPU-ONEGZZNKSA-N | CC(C)COC(=O)/C=C/C(=O)OC1=CC(=C(C(=C1Cl)Cl)Cl)Cl | 0 | 2 | 2 | 2 | 2 | 2 | 2 |
| Fumaric acid, pentyl 3,4,5-trichlorophenyl ester (C15H15Cl3O4) | ALDAXBNYLCOBDS-AATRIKPKSA-N | CCCCCOC(=O)/C=C/C(=O)OC1=CC(=C(C(=C1)Cl)Cl)Cl | 0 | 2 | 2 | 2 | 2 | 2 | 2 |
| Fumaric acid, 3,5-dichlorophenyl isohexyl ester (C16H18Cl2O4) | OHLCSBOWTPMGNR-AATRIKPKSA-N | CC(C)CCCOC(=O)/C=C/C(=O)OC1=CC(=CC(=C1)Cl)Cl | 0 | 2 | 2 | 2 | 2 | 2 | 2 |
| Bifenox (C14H9Cl2NO3) | SUSRORUBZHMPCO-UHFFFAOYSA-N | COC(=O)C1=C(C=CC(=C1)OC2=C(C=C(C=C2)Cl)Cl)[N+](=O)[O-] | 0 | 2 | 2 | 2 | 2 | 2 | 2 |
| Tris(4-chlorophenyl)phosphine (C18H12Cl3P) | IQKSLJOIKWOGIZ-UHFFFAOYSA-N | C1=CC(=CC=C1P(C2=CC=C(C=C2)Cl)C3=CC=C(C=C3)Cl)Cl | 222 | 1 | 2 | 1 | 1 | 1 | 1 |
| Alprazolam (C17H13ClN4) | VREFGVBLTWBCJP-UHFFFAOYSA-N | CC1=NN=C2N1C3=C(C=C(C=C3)Cl)C(=NC2)C4=CC=CC=C4 | 5795 | 1 | 1 | 1 | 1 | 1 | 1 |
| Succinic acid, decyl 2,2,2-trichloroethyl ester (C16H27Cl3O4) | FAIYXLQCQHTADH-UHFFFAOYSA-N | CCCCCCCCCCOC(=O)CCC(=O)OCC(Cl)(Cl)Cl | 0 | 2 | 2 | 2 | 2 | 2 | 2 |
| 1-chlorodocosane (C22H45Cl) | OACXFSZVCDOBKF-UHFFFAOYSA-N | CCCCCCCCCCCCCCCCCCCCCCCl | 20 | 2 | 2 | 2 | 2 | 2 | 2 |
| Adipic acid, butyl 8-chloroctyl ester (C18H33ClO4) | HETXEZMDDSWKRG-UHFFFAOYSA-N | CCCCOC(=O)CCCCC(=O)OCCCCCCCCCl | 40 | 2 | 2 | 2 | 2 | 2 | 2 |
| 5-Chlorovaleric acid, 5-pentadecyl ester (C20H39ClO2) | WRDBNJHXOVIVGG-UHFFFAOYSA-N | CCCCCCCCCCC(CCCC)OC(=O)CCCCCl | 0 | 2 | 2 | 2 | 2 | 2 | 2 |
| Succinic acid, 10-chlorodecyl isobutyl ester (C18H33ClO4) | JLLUCWADVCLWOF-UHFFFAOYSA-N | CC(C)COC(=O)CCC(=O)OCCCCCCCCCCCl | 50 | 2 | 2 | 2 | 2 | 2 | 2 |
| Adipic acid, 8-chloroctyl isobutyl ester (C18H33ClO4) | MNXUVEYWINNEBR-UHFFFAOYSA-N | CC(C)COC(=O)CCCCC(=O)OCCCCCCCCCl | 40 | 2 | 2 | 2 | 2 | 2 | 2 |
| Trichloroacetamide, N,N-bis(2-ethylhexyl)- (C18H34Cl3NO) | AQTKUBSOCLDIJW-UHFFFAOYSA-N | CCCCC(CC)CN(CC(CC)CCCC)C(=O)C(Cl)(Cl)Cl | 90 | 2 | 1 | 1 | 1 | 1 | 1 |
| Succinic acid, isohexyl 2,3,5-trichlorophenyl ester (C16H19Cl3O4) | QBLWHUHQTUEOND-UHFFFAOYSA-N | CC(C)CCCOC(=O)CCC(=O)OC1=CC(=CC(=C1Cl)Cl)Cl | 0 | 2 | 2 | 2 | 2 | 2 | 2 |
| 2-Chlorobenzoic acid, tridec-2-ynyl ester (C20H27ClO2) | RDJRHBFOTCUIHH-UHFFFAOYSA-N | CCCCCCCCCCC#CCOC(=O)C1=CC=CC=C1Cl | 0 | 2 | 2 | 2 | 2 | 2 | 2 |
| 2-Chlorobenzoic acid, 3-tridecyl ester (C20H31ClO2) | STPUHZZTPBQLLR-UHFFFAOYSA-N | CCCCCCCCCCC(CC)OC(=O)C1=CC=CC=C1Cl | 0 | 2 | 2 | 2 | 2 | 2 | 2 |
| 2-Chlorobenzoic acid, 4-tridecyl ester (C20H31ClO2) | AJBVRWQRFTWIJO-UHFFFAOYSA-N | CCCCCCCCCC(CCC)OC(=O)C1=CC=CC=C1Cl | 0 | 2 | 2 | 2 | 2 | 2 | 2 |
| Fumaric acid, isobutyl pentachlorophenyl ester (C14H11Cl5O4) | RRORQPBYPGTCSN-ONEGZZNKSA-N | CC(C)COC(=O)/C=C/C(=O)OC1=C(C(=C(C(=C1Cl)Cl)Cl)Cl)Cl | 0 | 2 | 2 | 2 | 2 | 2 | 2 |
| Fumaric acid, isohexyl 3,4,5-trichlorophenyl ester (C16H17Cl3O4) | HDCIXOJTYKFIFX-AATRIKPKSA-N | CC(C)CCCOC(=O)/C=C/C(=O)OC1=CC(=C(C(=C1)Cl)Cl)Cl | 0 | 2 | 2 | 2 | 2 | 2 | 2 |
| Chlorsulfuron (C12H12ClN5O4S) | VJYIFXVZLXQVHO-UHFFFAOYSA-N | CC1=NC(=NC(=N1)OC)NC(=O)NS(=O)(=O)C2=CC=CC=C2Cl | 286 | 1 | 2 | 2 | 2 | 2 | 2 |
| 9H-Purine-9-acetic acid, 6-[(p-chlorophenyl)amino)-, ethyl ester (C15H14ClN5O2) | GRZHGZVUENNXNQ-UHFFFAOYSA-N | CCOC(=O)CN1C=NC2=C1N=CN=C2NC3=CC=C(C=C3)Cl | 140 | 1 | 2 | 2 | 2 | 2 | 2 |
| 6-Chloro-N,N'-(cycloheptyl)-[1,3,5]triazine-2,4-diamine (C17H28ClN5) | NDHBZABQERFFOL-UHFFFAOYSA-N | C1CCCC(CC1)NC2=NC(=NC(=N2)Cl)NC3CCCCCC3 | 3450 | 1 | 1 | 1 | 2 | 2 | 1 |
| Amoxapine M (7-hydroxy) (C17H16ClN3O2) | MEUGUMOVYNSGEW-UHFFFAOYSA-N | C1CN(CCN1)C2=NC3=C(C=C(C=C3)O)OC4=C2C=C(C=C4)Cl | 0 | 2 | 2 | 2 | 2 | 2 | 2 |
| Oxazolam (C18H17ClN2O2) | VCCZBYPHZRWKFY-UHFFFAOYSA-N | CC1CN2CC(=O)NC3=C(C2(O1)C4=CC=CC=C4)C=C(C=C3)Cl | 0 | 2 | 2 | 1 | 2 | 2 | 2 |
| Propanoic acid, 3-chloro, octadecyl ester (C21H41ClO2) | IWNDNJGFFCNPKN-UHFFFAOYSA-N | CCCCCCCCCCCCCCCCCCOC(=O)CCCl | 180 | 1 | 2 | 2 | 2 | 2 | 2 |
| Adipic acid, 8-chloroctyl pentyl ester (C19H35ClO4) | IIDRUMFLPGVZJE-UHFFFAOYSA-N | CCCCCOC(=O)CCCCC(=O)OCCCCCCCCCl | 30 | 2 | 2 | 2 | 2 | 2 | 2 |
| Propanamide, N,N-dinonyl-3-chloro- (C21H42ClNO) | IXOQRAVVQUMNPF-UHFFFAOYSA-N | CCCCCCCCCN(CCCCCCCCC)C(=O)CCCl | 5375 | 1 | 1 | 1 | 1 | 1 | 1 |
| Malonic acid, 2-chloropropyl tridecyl ester (C19H35ClO4) | XQULABOIZJGPFB-UHFFFAOYSA-N | CCCCCCCCCCCCCOC(=O)CC(=O)OCC(C)Cl | 0 | 2 | 2 | 2 | 2 | 2 | 2 |
| Propanamide, N,N-dinonyl-2-chloro- (C21H42ClNO) | CAYVAIPTSFKUIT-UHFFFAOYSA-N | CCCCCCCCCN(CCCCCCCCC)C(=O)C(C)Cl | 9248 | 1 | 1 | 1 | 1 | 1 | 1 |
| Fumaric acid, 2,2-dichloroethyl dodecyl ester (C18H30Cl2O4) | IDWSWXWSQPMUQF-OUKQBFOZSA-N | CCCCCCCCCCCCOC(=O)/C=C/C(=O)OCC(Cl)Cl | 220 | 1 | 2 | 1 | 2 | 2 | 2 |
| Fumaric acid, 10-chlorodecyl pentyl ester (C19H33ClO4) | AHHWRJVIUFWBFV-BUHFOSPRSA-N | CCCCCOC(=O)/C=C/C(=O)OCCCCCCCCCCCl | 20 | 2 | 2 | 2 | 2 | 2 | 2 |
| Fumaric acid, 2-chloropropyl dodecyl ester (C19H33ClO4) | KBMFXTYTNXLVHL-BUHFOSPRSA-N | CCCCCCCCCCCCOC(=O)/C=C/C(=O)OCC(C)Cl | 110 | 1 | 2 | 2 | 2 | 2 | 2 |
| Succinic acid, heptyl 2,3,5-trichlorophenyl ester (C17H21Cl3O4) | XOIGLVMSEUBMHM-UHFFFAOYSA-N | CCCCCCCOC(=O)CCC(=O)OC1=CC(=CC(=C1Cl)Cl)Cl | 0 | 2 | 2 | 2 | 2 | 2 | 2 |
| Fumaric acid, hexyl 2,3,5,6-tetrachlorophenyl ester (C16H16Cl4O4) | OFPNLEPZXPVEHX-VOTSOKGWSA-N | CCCCCCOC(=O)/C=C/C(=O)OC1=C(C(=CC(=C1Cl)Cl)Cl)Cl | 0 | 2 | 2 | 2 | 2 | 2 | 2 |
| Fumaric acid, isohexyl 2,3,5,6-tetrachlorophenyl ester (C16H16Cl4O4) | ISIZDRAVGGKUKL-AATRIKPKSA-N | CC(C)CCCOC(=O)/C=C/C(=O)OC1=C(C(=CC(=C1Cl)Cl)Cl)Cl | 0 | 2 | 2 | 2 | 2 | 2 | 2 |
| Furosemide, trimethyl (C15H17ClN2O5S) | OBJFIAVSHHYZPK-UHFFFAOYSA-N | CNS(=O)(=O)C1=C(C=C(C(=C1)C(=O)OC)N(C)CC2=CC=CO2)Cl | 0 | 2 | 2 | 1 | 2 | 2 | 2 |
| Coumachlor (C19H15ClO4) | DEKWZWCFHUABHE-UHFFFAOYSA-N | CC(=O)CC(C1=CC=C(C=C1)Cl)C2=C(C3=CC=CC=C3OC2=O)O | 0 | 2 | 2 | 2 | 2 | 2 | 2 |
| Adipic acid, 8-chloroctyl hexyl ester (C20H37ClO4) | QOKXDWWCPGIUMU-UHFFFAOYSA-N | CCCCCCOC(=O)CCCCC(=O)OCCCCCCCCCl | 40 | 2 | 2 | 2 | 2 | 2 | 2 |
| Succinic acid, 10-chlorodecyl isohexyl ester (C20H37ClO4) | BZKVKBXJIOJRKG-UHFFFAOYSA-N | CC(C)CCCOC(=O)CCC(=O)OCCCCCCCCCCCl | 30 | 2 | 2 | 2 | 2 | 2 | 2 |
| Hexanamide, N,N-bis(2-ethylhexyl)-6-chloro- (C22H44ClNO) | QIAXMLYRWAMPLX-UHFFFAOYSA-N | CCCCC(CC)CN(CC(CC)CCCC)C(=O)CCCCCCl | 40 | 2 | 1 | 1 | 1 | 1 | 1 |
| Succinic acid, dodecyl 2,2,2-trichloroethyl ester (C18H31Cl3O4) | BBLDVAZHTUMKSA-UHFFFAOYSA-N | CCCCCCCCCCCCOC(=O)CCC(=O)OCC(Cl)(Cl)Cl | 0 | 2 | 2 | 2 | 2 | 2 | 2 |
| Fumaric acid, dodecyl 2,2,2-trichloroethyl ester (C18H29Cl3O4) | IFPVIGYQBLXYMK-OUKQBFOZSA-N | CCCCCCCCCCCCOC(=O)/C=C/C(=O)OCC(Cl)(Cl)Cl | 1081 | 1 | 1 | 1 | 1 | 1 | 1 |
| Sarcosine, N-(4-chlorobenzoyl)-, decyl ester (C20H30ClNO3) | UPRSSGWYPBBRLK-UHFFFAOYSA-N | CCCCCCCCCCOC(=O)CN(C)C(=O)C1=CC=C(C=C1)Cl | 0 | 2 | 2 | 2 | 2 | 2 | 2 |
| β-Alanine, N-(2-chlorobenzoyl)-, decyl ester (C20H30ClNO3) | QYWPBIFYAZPZMP-UHFFFAOYSA-N | CCCCCCCCCCOC(=O)CCNC(=O)C1=CC=CC=C1Cl | 70 | 2 | 2 | 2 | 2 | 2 | 2 |
| β-Alanine, N-(4-chlorobenzoyl)-, decyl ester (C20H30ClNO3) | YCYJYQKVEYADGA-UHFFFAOYSA-N | CCCCCCCCCCOC(=O)CCNC(=O)C1=CC=C(C=C1)Cl | 100 | 2 | 2 | 2 | 2 | 2 | 2 |
| 2-Chlorobenzoic acid, 3-pentadecyl ester (C22H35ClO2) | LIPZCBXPWHSJGI-UHFFFAOYSA-N | CCCCCCCCCCCCC(CC)OC(=O)C1=CC=CC=C1Cl | 0 | 2 | 2 | 2 | 2 | 2 | 2 |
| Fumaric acid, heptyl 2,3,4,6-tetrachlorophenyl ester (C17H18Cl4O4) | GOGVZHKCOHWPGO-BQYQJAHWSA-N | CCCCCCCOC(=O)/C=C/C(=O)OC1=C(C(=C(C=C1Cl)Cl)Cl)Cl | 0 | 2 | 2 | 2 | 2 | 2 | 2 |
| Fumaric acid, octyl 2,4,6-trichlorophenyl ester (C18H21Cl3O4) | CEIGQFJBTNZSFB-CMDGGOBGSA-N | CCCCCCCCOC(=O)/C=C/C(=O)OC1=C(C=C(C=C1Cl)Cl)Cl | 0 | 2 | 2 | 2 | 2 | 2 | 2 |
| 2-Naphthalenecarboxamide, N-(5-chloro-2,4-dimethoxyphenyl)-3-hydroxy- (C19H16ClNO4) | XDWATWCCUTYUDE-UHFFFAOYSA-N | COC1=CC(=C(C=C1NC(=O)C2=CC3=CC=CC=C3C=C2O)Cl)OC | 0 | 2 | 2 | 2 | 2 | 2 | 2 |
| Amodiaquine (C20H22ClN3O) | OVCDSSHSILBFBN-UHFFFAOYSA-N | CCN(CC)CC1=C(C=CC(=C1)NC2=C3C=CC(=CC3=NC=C2)Cl)O | 0 | 2 | 2 | 2 | 2 | 2 | 2 |
| Miconazole (C18H14Cl4N2O) | BYBLEWFAAKGYCD-UHFFFAOYSA-N | C1=CC(=C(C=C1Cl)Cl)COC(CN2C=CN=C2)C3=C(C=C(C=C3)Cl)Cl | 0 | 2 | 2 | 2 | 2 | 2 | 2 |
| Indomethacin (C19H16ClNO4) | CGIGDMFJXJATDK-UHFFFAOYSA-N | CC1=C(C2=C(N1C(=O)C3=CC=C(C=C3)Cl)C=CC(=C2)OC)CC(=O)O | 0 | 2 | 2 | 2 | 2 | 2 | 2 |
| Cyclohexanone, 2-(3-chloro-2-butenyl)-2-methyl-6,6-diphenyl- (C23H25ClO) | TWBAHMRTJVSXOP-NBVRZTHBSA-N | C/C(=C\CC1(CCCC(C1=O)(C2=CC=CC=C2)C3=CC=CC=C3)C)/Cl | 0 | 2 | 2 | 2 | 2 | 2 | 2 |
| Succinic acid, di(8-chloroctyl) ester (C20H36Cl2O4) | WSNMNGSFXRGKBL-UHFFFAOYSA-N | C(CCCCCl)CCCOC(=O)CCC(=O)OCCCCCCCCCl | 50 | 2 | 2 | 2 | 2 | 2 | 2 |
| Succinic acid, 2,2,2-trichloroethyl tridecyl ester (C19H33Cl3O4) | GSNGKIWQLUMYKR-UHFFFAOYSA-N | CCCCCCCCCCCCCOC(=O)CCC(=O)OCC(Cl)(Cl)Cl | 0 | 2 | 2 | 2 | 2 | 2 | 2 |
| β-Alanine, N-(2-chlorobenzoyl)-, undecyl ester (C21H32ClNO3) | OGLQUWIGLHDZMG-UHFFFAOYSA-N | CCCCCCCCCCCOC(=O)CCNC(=O)C1=CC=CC=C1Cl | 80 | 2 | 2 | 2 | 2 | 2 | 2 |
| Benzamide, N,N-bis(2-ethylhexyl)-4-chloro- (C23H38ClNO) | MMHNMAOTACRUMR-UHFFFAOYSA-N | CCCCC(CC)CN(CC(CC)CCCC)C(=O)C1=CC=C(C=C1)Cl | 0 | 2 | 2 | 2 | 2 | 2 | 2 |
| Succinic acid, di(2,3,5-trichlorophenyl) ester (C16H8Cl6O4) | POGRXWJDWDDOOU-UHFFFAOYSA-N | C1=C(C=C(C(=C1Cl)Cl)OC(=O)CCC(=O)OC2=CC(=CC(=C2Cl)Cl)Cl)Cl | 0 | 2 | 2 | 2 | 2 | 2 | 2 |
| Benzthiazide (C15H14ClN3O4S3) | NDTSRXAMMQDVSW-UHFFFAOYSA-N | C1=CC=C(C=C1)CSCC2=NS(=O)(=O)C3=CC(=C(C=C3N2)Cl)S(=O)(=O)N | 0 | 2 | 2 | 2 | 2 | 2 | 2 |
| Quizalofop-P-ethyl (C19H17ClN2O4) | OSUHJPCHFDQAIT-UHFFFAOYSA-N | CCOC(=O)C(C)OC1=CC=C(C=C1)OC2=CN=C3C=C(C=CC3=N2)Cl | 20 | 2 | 2 | 2 | 2 | 2 | 2 |
| Hydroxyzine (C21H27ClN2O2) | ZQDWXGKKHFNSQK-UHFFFAOYSA-N | C1CN(CCN1CCOCCO)C(C2=CC=CC=C2)C3=CC=C(C=C3)Cl | 0 | 2 | 2 | 2 | 2 | 2 | 2 |
| cis-Permethrin (C21H20Cl2O3) | RLLPVAHGXHCWKJ-HKUYNNGSSA-N | CC1([C@H]([C@H]1C(=O)OCC2=CC(=CC=C2)OC3=CC=CC=C3)C=C(Cl)Cl)C | 20 | 2 | 2 | 2 | 2 | 2 | 2 |
| Fumaric acid, 2,2-dichloroethyl pentadecyl ester (C21H36Cl2O4) | MWXYEQDJMAXBGQ-FOCLMDBBSA-N | CCCCCCCCCCCCCCCOC(=O)/C=C/C(=O)OCC(Cl)Cl | 130 | 1 | 2 | 1 | 2 | 2 | 2 |
| Sarcosine, N-(2-chlorobenzoyl)-, dodecyl ester (C22H34ClNO3) | QNPJVNNCUBIYBF-UHFFFAOYSA-N | CCCCCCCCCCCCOC(=O)CN(C)C(=O)C1=CC=CC=C1Cl | 90 | 2 | 2 | 2 | 1 | 2 | 1 |
| Fumaric acid, decyl 2,3,6-trichlorophenyl ester (C20H25Cl3O4) | UKPVUYCTLVSBPN-OUKQBFOZSA-N | CCCCCCCCCCOC(=O)/C=C/C(=O)OC1=C(C=CC(=C1Cl)Cl)Cl | 0 | 2 | 2 | 2 | 2 | 2 | 2 |
| Bicyclo[2.2.1]hept-5-ene-2,3-dicarboxylic acid, 1,4,5,6,7,7-hexachloro-, dibutyl ester (C17H20Cl6O4) | UJAHPBDUQZFDLA-UHFFFAOYSA-N | CCCCOC(=O)C1C(C2(C(=C(C1(C2(Cl)Cl)Cl)Cl)Cl)Cl)C(=O)OCCCC | 0 | 2 | 1 | 2 | 2 | 1 | 2 |
| Endrin (C12H8Cl6O) | DFBKLUNHFCTMDC-GKRDHZSOSA-N | C1[C@@H]2[C@@H]3[C@H]([C@H]1[C@H]4[C@@H]2O4)[C@@]5(C(=C([C@]3(C5(Cl)Cl)Cl)Cl)Cl)Cl | 1169 | 1 | 1 | 1 | 1 | 1 | 1 |
| Succinic acid, 2,2-dichloroethyl hexadecyl ester (C22H40Cl2O4) | UOCYXFFWUUTHLW-UHFFFAOYSA-N | CCCCCCCCCCCCCCCCOC(=O)CCC(=O)OCC(Cl)Cl | 0 | 2 | 2 | 2 | 2 | 2 | 2 |
| Fumaric acid, 10-chlorodecyl nonyl ester (C23H41ClO4) | ACJORMVAPZCSEZ-ISLYRVAYSA-N | CCCCCCCCCOC(=O)/C=C/C(=O)OCCCCCCCCCCCl | 30 | 2 | 2 | 2 | 2 | 2 | 2 |
| Succinic acid, 3,5-dichlorophenyl dodecyl ester (C22H32Cl2O4) | VVFZSHOFCJGLEL-UHFFFAOYSA-N | CCCCCCCCCCCCOC(=O)CCC(=O)OC1=CC(=CC(=C1)Cl)Cl | 0 | 2 | 2 | 2 | 2 | 2 | 2 |
| Fumaric acid, 2-chloro-5-methylphenyl dodecyl ester (C23H33ClO4) | PKAHTTPOKHAOIZ-FOCLMDBBSA-N | CCCCCCCCCCCCOC(=O)/C=C/C(=O)OC1=C(C=CC(=C1)C)Cl | 230 | 1 | 1 | 1 | 1 | 1 | 1 |
| Succinic acid, hexadecyl 2,2,2-trichloroethyl ester (C22H39Cl3O4) | GGIKVMZYWDHFFS-UHFFFAOYSA-N | CCCCCCCCCCCCCCCCOC(=O)CCC(=O)OCC(Cl)(Cl)Cl | 0 | 2 | 2 | 2 | 2 | 2 | 2 |
| Succinic acid, dodecyl 2,3,6-trichlorophenyl ester (C22H31Cl3O4) | ZISPEBQCKRKILO-UHFFFAOYSA-N | CCCCCCCCCCCCOC(=O)CCC(=O)OC1=C(C=CC(=C1Cl)Cl)Cl | 0 | 2 | 2 | 2 | 2 | 2 | 2 |
| β-Alanine, N-(2-chlorobenzoyl)-, tetradecyl ester (C24H38ClNO3) | WGEPOKJCBMIFIN-UHFFFAOYSA-N | CCCCCCCCCCCCCCOC(=O)CCNC(=O)C1=CC=CC=C1Cl | 60 | 2 | 2 | 2 | 2 | 2 | 2 |
| Sarcosine, N-(4-chlorobenzoyl)-, tetradecyl ester (C24H38ClNO3) | GVTFKZULRJMDTB-UHFFFAOYSA-N | CCCCCCCCCCCCCCOC(=O)CN(C)C(=O)C1=CC=C(C=C1)Cl | 0 | 2 | 2 | 2 | 2 | 2 | 2 |
| Fumaric acid, 8-chlorooctyl tridecyl ester (C25H45ClO4) | KXJLNMQRVDAJCN-FMQUCBEESA-N | CCCCCCCCCCCCCOC(=O)/C=C/C(=O)OCCCCCCCCCl | 30 | 2 | 2 | 2 | 2 | 2 | 2 |
| Fumaric acid, 2,4,6-trichlorophenyl tridecyl ester (C23H31Cl3O4) | RFHVSCGCTYDDEL-BUHFOSPRSA-N | CCCCCCCCCCCCCOC(=O)/C=C/C(=O)OC1=C(C=C(C=C1Cl)Cl)Cl | 0 | 2 | 2 | 2 | 2 | 2 | 2 |
| Succinic acid, 2,2-dichloroethyl nonadecyl ester (C25H46Cl2O4) | ZTESYAMDHFJNHK-UHFFFAOYSA-N | CCCCCCCCCCCCCCCCCCCOC(=O)CCC(=O)OCC(Cl)Cl | 0 | 2 | 2 | 2 | 2 | 2 | 2 |
| Succinic acid, octadecyl 2,2,2-trichloroethyl ester (C24H43Cl3O4) | HIGJJEDMWGWLJX-UHFFFAOYSA-N | CCCCCCCCCCCCCCCCCCOC(=O)CCC(=O)OCC(Cl)(Cl)Cl | 0 | 2 | 2 | 2 | 2 | 2 | 2 |
| Succinic acid, 2,3,4,6-tetrachlorophenyl tridecyl ester (C23H32Cl4O4) | FFQOTIQTIMSPCZ-UHFFFAOYSA-N | CCCCCCCCCCCCCOC(=O)CCC(=O)OC1=C(C(=C(C=C1Cl)Cl)Cl)Cl | 0 | 2 | 2 | 2 | 2 | 2 | 2 |
| Adipic acid, 8-chloroctyl tridecyl ester (C27H51ClO4) | KWSLVIWMUBDKCJ-UHFFFAOYSA-N | CCCCCCCCCCCCCOC(=O)CCCCC(=O)OCCCCCCCCCl | 20 | 2 | 2 | 2 | 2 | 2 | 2 |
| Succinic acid, pentadecyl 2,3,6-trichlorophenyl ester (C25H37Cl3O4) | BYEZQRLTMPJVRB-UHFFFAOYSA-N | CCCCCCCCCCCCCCCOC(=O)CCC(=O)OC1=C(C=CC(=C1Cl)Cl)Cl | 0 | 2 | 2 | 2 | 2 | 2 | 2 |
| Adipic acid, 8-chloroctyl tetradecyl ester (C28H53ClO4) | NECYQUIQLJYGQL-UHFFFAOYSA-N | CCCCCCCCCCCCCCOC(=O)CCCCC(=O)OCCCCCCCCCl | 20 | 2 | 2 | 2 | 2 | 2 | 2 |
